# Evaluating and clustering retrosynthesis pathways with learned strategy†

**DOI:** 10.1039/d0sc05078d

**Published:** 2020-11-23

**Authors:** Yiming Mo, Yanfei Guan, Pritha Verma, Jiang Guo, Mike E. Fortunato, Zhaohong Lu, Connor W. Coley, Klavs F. Jensen

**Affiliations:** Department of Chemical Engineering, Massachusetts Institute of Technology Cambridge Massachusetts 02139 USA kfjensen@mit.edu; College of Chemical and Biological Engineering, Zhejiang University Hangzhou Zhejiang Province 310007 China; ZJU-Hangzhou Global Scientific and Technological Innovation Center Hangzhou Zhejiang Province 311215 China; Computer Science and Artificial Intelligence Laboratory, Massachusetts Institute of Technology Cambridge Massachusetts 02139 USA; Department of Chemistry, Massachusetts Institute of Technology Cambridge Massachusetts 02139 USA

## Abstract

With recent advances in the computer-aided synthesis planning (CASP) powered by data science and machine learning, modern CASP programs can rapidly identify thousands of potential pathways for a given target molecule. However, the lack of a holistic pathway evaluation mechanism makes it challenging to systematically prioritize strategic pathways except for using some simple heuristics. Herein, we introduce a data-driven approach to evaluate the relative strategic levels of retrosynthesis pathways using a dynamic tree-structured long short-term memory (tree-LSTM) model. We first curated a retrosynthesis pathway database, containing 238k patent-extracted pathways along with ∼55 M artificial pathways generated from an open-source CASP program, ASKCOS. The tree-LSTM model was trained to differentiate patent-extracted and artificial pathways with the same target molecule in order to learn the strategic relationship among single-step reactions within the patent-extracted pathways. The model achieved a top-1 ranking accuracy of 79.1% to recognize patent-extracted pathways. In addition, the trained tree-LSTM model learned to encode pathway-level information into a representative latent vector, which can facilitate clustering similar pathways to help illustrate strategically diverse pathways generated from CASP programs.

## Abstract 2

Tree-structured long short-term memory neural model learns to understand the retrosynthesis design strategies from patent-extracted retrosynthetic pathway data.

## Graphical abstract

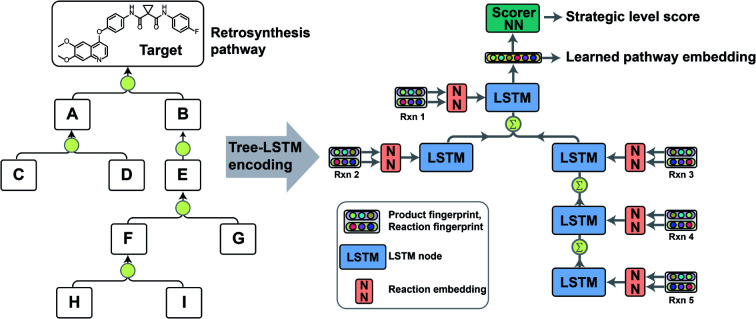

## Introduction

Computer-aided synthesis planning (CASP), initially proposed by Corey,^1^ has recently been extensively investigated and improved with the implementation of data science and machine learning.^2–5^ CASP aims at decomposing the target molecule step by step into commercially available compounds or simple precursors that can be easily synthesized. During this process, single-step retrosynthetic reactions can be proposed using reaction templates (expert-encoded reaction rules^3,6,7^ or machine-extracted retrosynthetic transformations^8–10^) or template-free retrosynthesis models.^4,11–14^ For each intermediate molecule, there could be numerous valid strategies to transform it into corresponding precursors. To avoid the combinatorial explosion during recursive expansion to find viable multistep retrosynthesis pathways, either heuristic rules^15,16^ or data-driven ranking models^2,5,8^ can be implemented to prioritize promising single-step retrosynthetic reactions. Depending on the constraints that users set for the retrosynthesis search, such as search time and number of single-step expansions allowed per intermediate, a successful retrosynthetic search could result in thousands of potential retrosynthesis pathways. For example, the open-source program, ASKCOS,^5,17,18^ gave a total of 1498 different retrosynthesis pathways for hydroxychloroquine with only 30 seconds search time on a 20-core workstation.

Two challenges naturally arise with the large number of pathways proposed by the modern CASP programs:

(1) Prioritizing strategic retrosynthesis pathways. In spite of the effort to improve the quality of the single-step retrosynthetic transformation, the final retrosynthesis pathways found may not be useful even though each single-step reaction is valid and selective. As an intuitive example, protection and deprotection reactions are important steps in the retrosynthesis design; however, without pathway-level guidance during the retrosynthetic search, the program could produce pathways composed of a series of nonproductive protection and deprotection reactions.

(2) Clustering similar retrosynthesis pathways. A majority of the retrosynthesis pathways proposed differ only at a sub-portion level, leaving users overwhelmed by similar pathways, and making it hard to focus on the pathways that are strategically different.

Simple heuristics can be implemented to partially mitigate these two challenges. Sorting retrosynthesis pathways by the number of reaction steps can easily prioritize pathways that contain no or fewer nonproductive steps (*e.g.* a series of protection and deprotection reactions). Schwaller *et al.*^4^ and Lin *et al.*^14^ designed customized scoring functions, which aggregates the single-step reaction likelihood and the degree of molecule simplification, to evaluate candidate retrosynthesis reactions in the tree search. These heuristic scoring functions will guide the tree search towards simple precursors. Alternatively, Badowski *et al.*^7^ excluded protection and deprotection reaction rules during the retrosynthetic search to focus only on the productive disconnections. They treated protection reactions as a mask for the incompatible functional groups. However, this is only possible with their expert-encoded reaction rules that have extensive information about reaction type and functional group tolerance. In addition, application-oriented metrics can also be used to sort pathways. For example, price of the final target is one of key considerations for process chemistry. Badowski *et al.*^19^ developed a price estimator that used recursive formulae to assign cost to individual components along the pathways, and price penalties were applied to strategically similar pathways to ensure diversity in the top-ranking routes. Despite their inclusion of many expert-designed considerations when estimating the price, such as reaction yield and reaction cost composed of labor plus equipment/solvent/purification, target compound price estimation may still remain challenging without accurate prediction of the reaction stoichiometry, reaction concentration, and separation efficiency.

Applying these heuristics during the retrosynthesis search can certainly guide the retrosynthesis search towards more desired pathways. However, retrosynthetic design is often referred to as an art, and these heuristics can also potentially lead to missing “smart” pathway designs that, otherwise, could be found without these heuristics. For example, it can be tactically beneficial to temporarily increase complexity with directing groups or protecting groups for significant structural simplification in the subsequent steps in the retrosynthesis pathway.^20^ Gajewska *et al.* designed an algorithm to enable automatic discovery of new tactical two-step syntheses that involves counterintuitive complexity increase in the first step,^21^ highlighting that such tactical synthetic strategies are often ignored by retrosynthesis programs with the current implementation of the expert-enforced heuristics, *i.e.* preferring simple and short pathways.

Thus, it remains of interest to develop a methodology to evaluate CASP retrosynthesis pathways based on their strategic viability and to cluster similar pathways after they are generated. In this work, we address these two challenges *via* a data-driven approach, which has the potential to avoid any bias introduced by expert-designed rules. First, we curate a retrosynthesis pathway database containing pathways extracted from a commercial patent reaction database, Pistachio, and machine-designed pathways using the ASKCOS program.^5^ Due to the lack of readily available models to encode information of the whole pathway,^14^ we built a dynamic tree-structured LSTM model to encode pathways with various structures into a latent vector. The pathway encoder was trained on the curated database to differentiate between patent-extracted and machine-designed pathways with the purpose of understanding the relative strategic level of different pathways. This learned latent vector aggregates the pathway-level information that can be used for either ranking different pathways with the same target molecule, or clustering strategically similar pathways.

## Results and discussion

### Curating the retrosynthesis pathway database

Previous efforts on reaction prediction and single-step retrosynthesis planning have relied on public or proprietary single-step reaction databases, such as Reaxys,^22^ USPTO,^23^ and Pistachio.^24^ In contrast, an accessible and well-curated retrosynthesis pathway database is not available. One exception is Drug Future, which offers a public Drug Preparation Database containing retrosynthesis pathway information of 7000 commercial or investigational drugs.^25^ However, the data is provided as either images or texts, which require substantial effort to make them machine-readable. This challenge motivated us to extract and build a machine-readable database of multistep retrosynthesis pathways from single-step reaction databases.

**Fig. 1:**
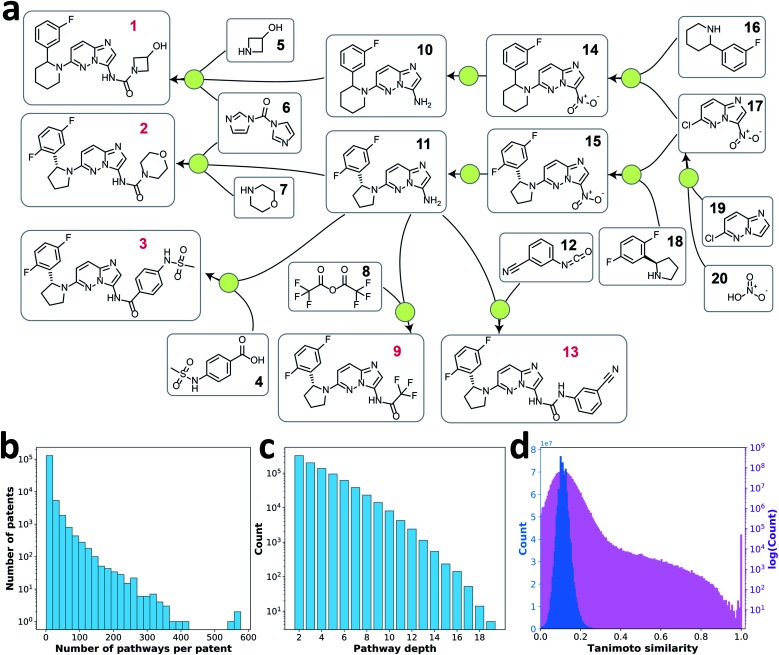
(a) A reaction network extracted from patent US10011604B2. Each green dot represents a reaction node connecting product to its reactants, and reagents in each reaction are omitted. The compounds with red labels are root nodes. Retrosynthesis pathways that can be extracted from this reaction network include: (1) [1] → [5, 6, 10] → [14] → [16, 17] → [19, 20]; (2) [2] → [6, 7, 11] → [15] → [17, 18] → [19, 20]; (3) [3] → [4, 11] → [15] → [17, 18] → [19, 20]; (4) [9] → [8, 11] → [15] → [17, 18] → [19, 20]; (5) [13] → [11, 12] → [15] → [17, 18] → [19, 20]. (b) Histogram of number of retrosynthesis pathways extracted per patent. (c) Histogram of depth of extracted retrosynthesis pathways. (d) Distribution of pairwise Tanimoto similarities between pairs of 50 000 randomly selected target molecules in retrosynthesis pathways.

Converting single-step reactions into a reaction network (*i.e.* a directed graph) can help to identify pathways in the network. However, a reaction network of the whole database will contain single-step reactions from various literature sources, where the roles of products and reactants could be reversed creating the possibility of cyclic reaction paths. As a consequence, it could be difficult to define a meaningful retrosynthesis pathway algorithmically. Considering that drug or fine chemical patents are typically preparation-oriented, single-step reactions extracted from a single patent would be highly related with fewer cyclic patterns. As the example shown in Fig. 1, a reaction network was constructed from a recent patent (US10011604B2). Starting from root nodes, *i.e.* compounds only appearing as products and not as reactants, traversing through the network with a complete depth-first search (DFS) algorithm will give all the retrosynthesis pathways embedded in the network. Reagents were omitted from the network to make the neural model focus on assessing the retrosynthesis design strategy, *i.e.* how a target molecule is decomposed step by step towards commercially available precursors, rather than on minor differences in reagent choices for a particular transformation. To improve data quality, we implemented the state-of-the-art atom mapping algorithm, RXNmapper,^26^ for reaction validation and accurate differentiation between reactants and reagents.

With this pathway curation algorithm, we extracted 907 209 retrosynthesis pathways with a depth of 2–20 from the single-step reaction patent database, Pistachio.^24^ The extraction process would work similarly on other single-step reaction databases that contain reaction source identifiers (*e.g.* USPTO^23^ database with patent numbers and Reaxys^22^ database with literature identifiers). 85% of patents provided fewer than 10 pathways each (Fig. 1b). The distribution of pathway depth is shown in Fig. 1c. Because the goal of this work was to learn the design strategies of multistep retrosynthesis pathways, we focused on the pathways of depth 4 to 10, excluding very short pathways (depth of 2 and 3) that seldom reflect strategic design information, as well as lengthy pathways (depth >10), typically undesired in practice. Using these pathways, we examined the target compounds' similarity to ensure the diversity of the retrosynthesis pathways curated. Fig. 1d shows the pairwise Tanimoto similarity of 50 000 randomly selected target compounds, where 98% of the molecule pairs show a similarity between 0 and 0.2, indicating diverse target molecules of the retrosynthesis pathway data were explored.

Next, for each patent-extracted pathway, we used the ASKCOS program^5^ to generate a set of artificial retrosynthesis pathways with the same target compound as the corresponding patent-extracted pathway. Up to 300 artificial pathways were randomly selected from top 3000 pathways generated from ASKCOS. Ultimately, 238 379 patent pathways with depth between 4 and 10 were curated, and each pathway had 5–300 artificial pathways. This pathway database was randomly split into 80% training, 10% validation, and 10% testing data for the following study while ensuring that no pathways belonging to the same patent ended up in two different data groups.

### Tree-structured LSTM model

Linear or branched retrosynthesis pathways can be viewed as tree-structured data. For example, convergent synthesis contains multiple branches in order to reduce the maximum pathway depth for improved overall synthesis yield. Considering that there are no retrosynthesis pathway encoders readily available, we decided to implement the tree-structured long short-term memory network (tree-LSTM) model to encode the overall pathway information. The tree-LSTM algorithm was initially proposed for tasks such as semantic relatedness of two sentences and sentiment classification.^27^ It has recently been used to encode an organic molecule by converting atoms into tree nodes and bonds into tree connections.^28^ The encoded pathway is represented by a latent numeric vector, which will be further processed for two tasks as discussed above, ranking pathways based the relative strategic level and clustering similar pathways.

**Fig. 2:**
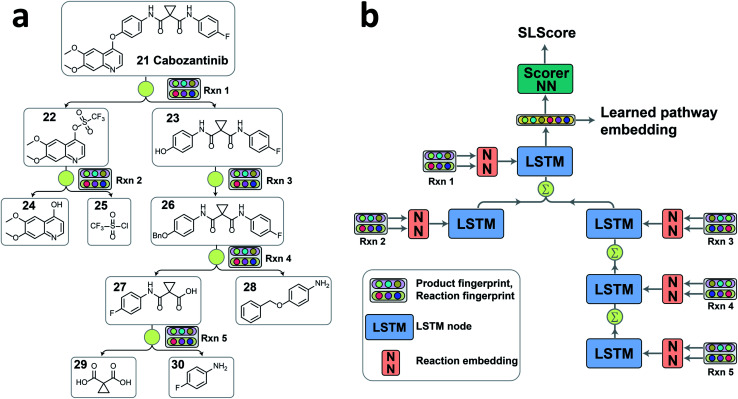
(a) A representative convergent synthesis pathway of cabozantinib 21 extracted from patent US20140155396A1. Each reaction and its corresponding product are converted to 2048 bit Morgan fingerprints with a radius of 2 as inputs for tree-LSTM model. (b) The structure and workflow of the tree-LSTM network. Each reaction node information passes through a feed-forward neural network (FFNN) to embed the reaction information into a latent vector as the input of LSTM node. Calculation starts from leaf nodes (Rxn 2 and Rxn 5) on the tree, and propagates following the tree connections towards the root node (Rxn 1). When a node has multiple child nodes, the information of child nodes is aggregated *via* a direct sum of hidden states and a weighted sum of cell states with forget gates. The hidden state of the root node is a latent vector of the pathway containing the overall pathway information. The latent vector can be passed through a scorer neural network to give the strategic level score (SLScore) representing the design strategy of a pathway, or directly used as a numerical representation of the pathway for clustering purpose.

Since each retrosynthesis pathway has a different tree structure, the tree-LSTM structure is constructed on the fly accordingly (Fig. 2). The tree-LSTM model is designed to understand the design strategies of multistep reactions, and thus, each reaction in the pathway is considered as a node, and the reaction nodes are connected *via* intermediate compounds as the edges. The Morgan fingerprints of products^29^ and reactions^30^ with 2048 bits and a radius of 2 as implemented in RDKit^31^ were used to encode the reaction node information.^2,29,30^ Using both reaction fingerprint and product fingerprint as inputs gives the model a complete picture of the reaction core and the unchanged fragments. This encoded reaction representation was then fed into a reaction embedding neural network. The structure of the tree-LSTM network is identical to the structure of the pathway tree, and each LSTM node takes in the corresponding learned reaction node embedding as input (Fig. 2b). Unlike linear-chained LSTM models, where the calculation propagates from the start to the end of the sequence or in the reversed direction, the tree-LSTM model evaluates child nodes first and then traverses the information back to their parent nodes *via* a direct sum of hidden states and a weighted sum of cell states with forget gates (see ESI for detailed descriptions†). The hidden state of the root node is the output of the tree-LSTM model, which is a latent vector representation of all reactions in the entire pathway. This latent vector can either be passed through a feedforward neural network (FFNN) scorer to give a relative strategic level score (SLScore) for comparing pathways with the same target molecule, or *via* unsupervised learning algorithms, it can be used to cluster pathways with the same target into subgroups with similar retrosynthesis designs.

### Pathway ranking based on strategic level

With the tree-LSTM model, we sought to train the model to understand the pathway-level information. The first task was ranking pathways based on their strategic level, which considers various aspects of the pathway design, such as whether there are nonproductive sequences of reactions, the complexity of the pathway design, and the commonality of decomposing the molecule in a certain way. In brief, the strategic level measures the likelihood of pathways to be carried out by chemists in practice. Each patent pathway has up to 300 artificial pathways with the same target compound as the patent pathway. The patent pathways were designed by chemists and evaluated in practice, while the quality of pathways proposed by current CASP programs varies wildly because current state-of-the-art retrosynthesis programs still only examine single-step plausibility without evaluating pathway-level design strategy. Thus, we assumed that patent pathways are more likely to be more strategic than the artificial pathways of the same targets. Although this assumption doesn't always hold since pathways proposed by ASKCOS have been demonstrated experimentally with successful syntheses of drug molecules,^5^ the consequence of having artificial pathways to be as strategic as or better than the patent pathways would only make it hard for the model to differentiate between those pathways. With this assumption, we aimed at training the tree-LSTM model to give a higher strategic level score (SLScore) for the patent pathway compared to its accompanied artificial pathways. With this training procedure, the SLScore is interpreted as a relative quantity that is only used for comparing pathways with the same target molecule. To be noted, the SLScore absolute value of an individual pathway has little meaningful information itself, or when comparing across pathways with different targets. The trained tree-LSTM pathway ranking model gave a top-1 accuracy of 79.1% on the testing dataset described above (Table 1).

**Table 1:** Overall top-*k* accuracy in pathway ranking tested using on the held-out testing dataset. Top-*k* accuracy denotes the percentage of data where patent-extracted pathway is ranked in the top-*k* scored pathways

Model	Depth^a^ (%)	SCScore (%)	Hybrid (%)	Tree-LSTM (%)
Top 1	13.9 (54.9)	33.5	39.6	79.1
Top 5	21.9 (63.0)	48.0	55.0	88.6
Top 10	29.0 (70.2)	58.0	64.3	92.6
Top 30	55.2 (85.6)	76.2	80.7	97.5
Top 50	72.0 (92.1)	83.6	87.0	98.7
Top 100	90.8 (97.7)	92.0	93.8	99.6

a Pathways with the same depth were given a unique ranking position. The worst-case and best-case scenario accuracy were reported outside and inside the parenthesis, respectively.

To facilitate the understanding of how the developed tree-LSTM model was capable of differentiating the patent pathways and artificial pathways, we implemented the following three baseline models that utilized heuristic metrics to rank pathways.

#### Depth baseline model

Pathway depth is often the first metric to consider since a short and simple retrosynthesis design is always preferred due to its reduced synthesis effort in practice. However, relying on this metric alone does not give a full picture of pathways' strategic level, resulting in 13.9% (54.9%) top-1 accuracy.

#### SCScore baseline model

A portion of the non-strategic pathways given by the retrosynthesis programs contain nonproductive sequence of reactions, leading to non-decreasing molecular complexity along the pathway. This pattern could be captured by the complexity change of intermediate compounds. To represent the evolution of complexity through the pathway, the second baseline model starts with linearizing the tree-structured retrosynthesis pathway into individual linear pathways *via* splitting at branching nodes, and then tracks the intermediates' complexity flow through each linear pathway with a complexity vector. We used SCScore developed by Coley *et al.*^20^ to quantify the complexity of each compound. For multi-reactant reactions, the most complex compound was selected to represent the complexity. The constructed complexity vector was then passed through a FFNN to generate a score for each linear pathway, followed by min-pooling to aggregate the scores of all linear pathways belonging to the same retrosynthesis pathway as its score. The min-pooling was used since the strategic level assessment of retrosynthesis pathways should be dominated by the least strategic linear pathway (see ESI for detailed model description†). Because the presence of the nonproductive sequences of reactions will lead to increasing the pathway depth, the improvement using the SCScore baseline over the depth baseline was only marginal (top-1 accuracy of 31%).

#### Hybrid baseline model

Next, we developed a third baseline model that used hybrid descriptors of the pathway. In addition to SCScore, this model also includes pathway depth, the number of linear pathways within a retrosynthesis tree, number of nodes and leaves, and maximum number of child nodes. To describe the intermediates' complexity evolution through the pathway without linearization as the previous SCScore baseline model, the complexity descriptors used in this hybrid baseline model are the maximum SCScore for leaf nodes, the minimum and the maximum SCScore for intermediates, the minimum and the maximum SCScore difference for each reaction, and the SCScore for the target compound. Similarly, the constructed vector of hybrid descriptors was passed through a FFNN to generate the strategic level score for each pathway (see ESI for detailed model description†). This hybrid model provided an improved ranking accuracy on the testing dataset, indicating that the strategic level is partially reflected among these descriptors.

The tree-LSTM model significantly outperformed baseline models in distinguishing the patent pathways from artificial ones (Table 1). As mentioned in the Introduction section, a strategic retrosynthetic design can be considered as an art indicating the difficulty to standardize the evaluation of a newly designed pathway. Using human-designed metrics similar to the three baseline models described above shows a low-to-medium level of success, and it is expected that adding more descriptors to the hybrid model will further improve the accuracy. On the other hand, directly learning from data with tree-LSTM model avoids bias introduced by the human-designed metrics.

**Fig. 3:**
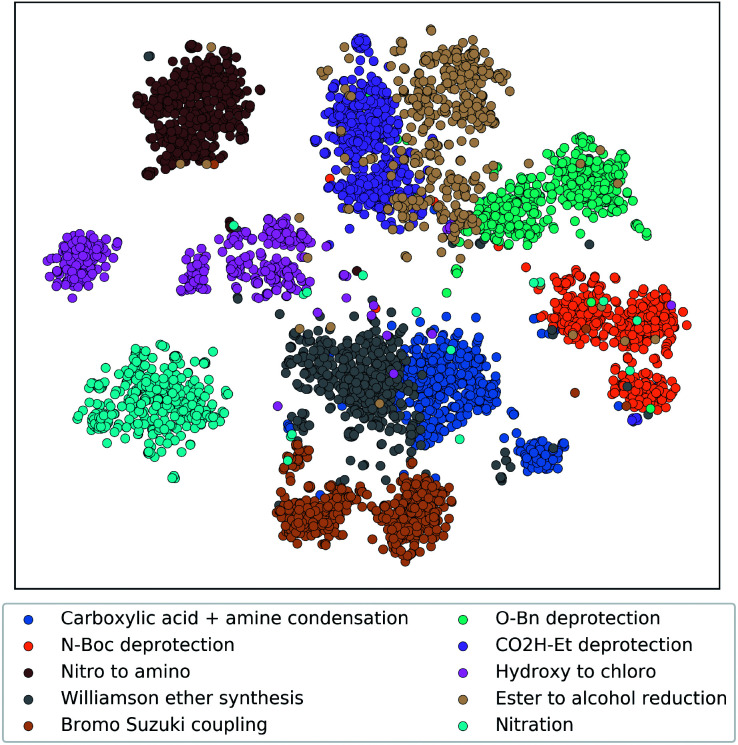
Embedding of single-step reactions from ten representative reaction classes projected to a two-dimension space using t-SNE. The embedding was generated by passing the single-step reaction features (product fingerprint and reaction fingerprint) through the trained reaction encoder. Each reaction class contains 600 randomly selected reaction records from the testing dataset. Reaction classes were assigned in the Pistachio database using the NameRxn tool.^33^

To demonstrate that the tree-LSTM model captures the overall single-step reaction relationship in the pathway, we examined the output of the reaction node embedding NN (*i.e.* the input to the LSTM node). 6000 randomly selected single-step reactions from the testing dataset belonging to 10 different frequently used reaction types were embedded using the trained reaction node embedding NN from the tree-LSTM model, giving a vector representation of each single-step reaction. These 6000 vector representations were projected to a two-dimensional space using t-Distributed Stochastic Neighbor Embedding (t-SNE) method^32^ (Fig. 3). Reactions of different types were clustered in groups, indicating that the trained reaction node embedding understands what type of reaction is performed at each reaction node. Then, the tree-LSTM model incorporates all single-step reactions and uses the characteristics of their interconnections to rank strategic pathways higher than non-strategic ones.

**Fig. 4:**
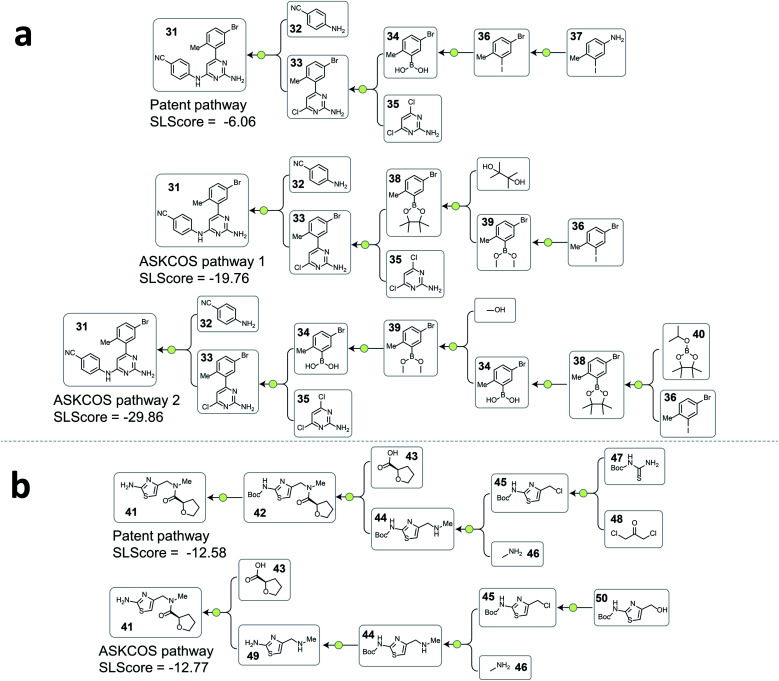
Examples from the testing dataset where tree-LSTM model scored the patent pathways higher than the ASKCOS pathways. (a) Example pathways from patent US07419984B2. (b) Example pathways from patent US20120015941A1.

**Fig. 5:**
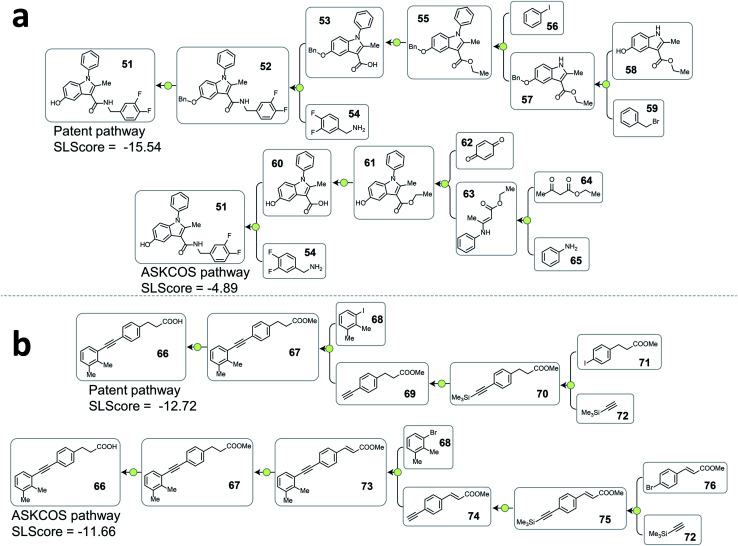
Examples from the testing dataset where tree-LSTM model scored the ASKCOS pathways higher than the patent pathways. (a) Example pathways from patent US07737173. (b) Example pathways from patent US08586607B2.

Fig. 4 and 5 depict several representative pathway ranking examples from the testing dataset, and additional examples can be found in the ESI.†

A consequence of not having pathway-level guidance when searching viable synthetic routes is the generation of nonproductive sequences of reactions despite each single-step reaction being feasible. In Fig. 4a. ASKCOS pathway 1 uses an indirect two-step approach for the synthesis of the boronic ester 38 from the aryl iodide 36, while it could be synthesized in a single step from 36 directly. Thus, despite that the ASKCOS pathway 1 has the same step count as the patent pathway, the tree-LSTM model gives it a slightly lower SLScore since the reaction sequence [35, 38] → [39] → [36] can be simplified with a single reaction. Furthermore, in the ASKCOS pathway 2, the unnecessary manipulation of the aryl boron reagents led to an extremely low SLScore. In addition to recognizing nonproductive reaction sequences, the tree-LSTM model is also able to capture pathways with functional group incompatibility issues, especially as it pertains to the strategic use of protecting groups. For example, the ASKCOS pathway in Fig. 4b, compared to the patent pathway, involves a reversed order of the Boc group deprotection step and the amide formation step. The potential site-selectivity issue arising in the amide bond formation step is captured effectively by the tree-LSTM model that assigns a lower SLScore to the ASKCOS pathway.

Analyzing the cases where the model failed help reveal the underlying reasons that the rest 20.9% of testing patent pathways were considered less strategic than some artificial pathways. In Fig. 5a, the high scoring ASKCOS pathway involved Nenitzescu indole synthesis as a key step that significantly reduces the complexity of the intermediates 61, leading to the usage of simpler starting materials and a shorter synthetic route compared to the patent pathway. This example echoes our previous assumption and demonstrates that, despite having artificial pathways that are more strategic than the patent pathways, the model was still able to learn to recognize good retrosynthetic designs proposed by ASKCOS. Nevertheless, training the tree-LSTM model as a ranking task, to some extent, limits model's capability besides understanding the relationship of single-step reactions. For example, the artificial pathway in Fig. 5b was given a slightly higher score than the patent pathway even though it unnecessarily utilizes an unsaturated ester containing starting material that is later reduced, thus introducing an additional step in the synthesis. This example demonstrates that the current tree-LSTM model is unable to evaluate pathways out of the scope of the given pathway information, *e.g.*, knowing that there are more desirable precursors to improve the retrosynthetic design.

### Clustering similar pathways

As demonstrated above, the tree-LSTM model was trained to capture the relationship among single-step reactions within a pathway, and the latent vector output from the root node is a learned embedding of the pathway. This pathway-level representation encodes both single-step reactions and their connectivity. Intuitively, this representation can be used to analyze the similarity between two pathways with the same target compound. Thus, we decided to use this learned pathway embedding to cluster retrosynthesis pathways given by the current ASKCOS program to tackle the challenges in organizing numerous retrosynthesis pathways found and only providing meaningfully different pathways for users to examine. The pathway embeddings were clustered with hierarchical density-based spatial clustering algorithm (HDBSCAN)^34^ to group pathways with similar strategies.

**Fig. 6:**
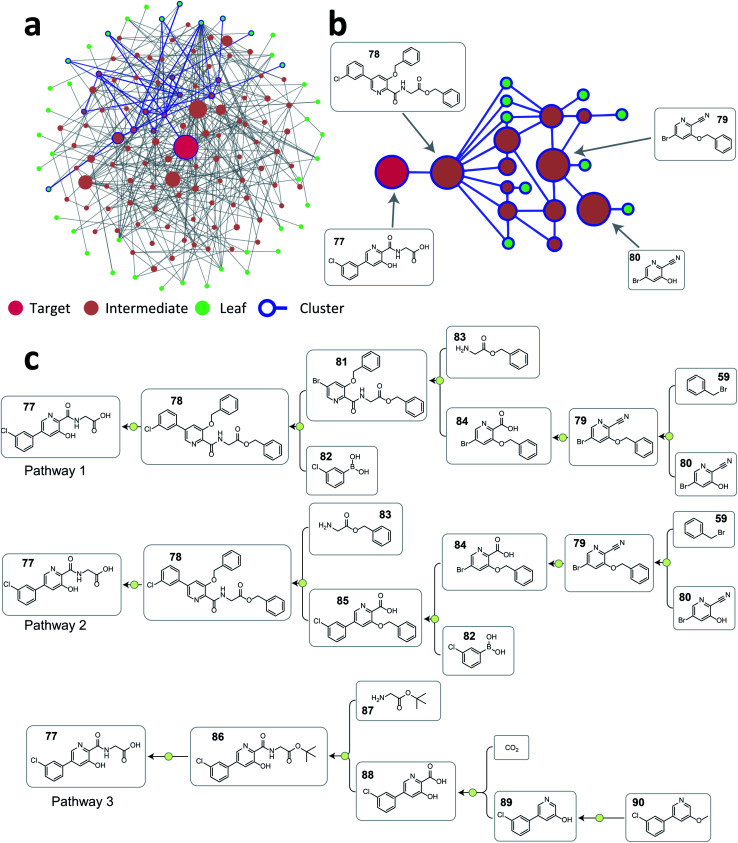
(a) The reaction network graph of 2000 retrosynthesis pathways of vadadustat 77 generated from ASKCOS. Each circle node represents a unique compound, and the node size is linearly correlated with its appearance counts among the 2000 pathways. Compounds and connections from one example cluster are highlighted with blue color. (b) The reaction network subgraph of the highlighted example cluster. The node size is linearly correlated with its appearance counts among this cluster. (c) Three representative pathways chosen from the 2000 pathways. Pathway 1 and 2 are from the example cluster shown in Fig. 6b, and pathway 3 is from a different cluster.

To illustrate how this approach can help organize a large number of pathways generated, we selected vadadustat 77 as the target molecule. After searching pathways for 45 seconds using ASKCOS, we selected the top 2000 pathways found for the following analysis (current ASKCOS ranks pathways based on pathway depth and plausibility of all single-step reactions). Fig. 6a shows the reaction network graph of these 2000 pathways, with each node and edge representing a unique compound and a reaction connection, respectively. Despite having 2000 pathways, there are only 142 unique compounds in total, indicating that many pathways share common intermediates. After clustering, the blue-color highlighted nodes and edges in Fig. 6a exemplifies a pathway cluster, and Fig. 6b zooms in this cluster showing that three major intermediates compounds are shared within this cluster. We picked two pathways from this cluster (pathway 1 and 2 in Fig. 6c), and they are strategically similar only with a reversed order of the amide formation reaction and Suzuki–Miyaura C–C coupling reaction. In contrast, the pathway from a different cluster (pathway 3 in Fig. 6c) is a fundamentally different retrosynthetic design, that installs the carboxylic acid group with a Kolbe–Schmitt reaction on the phenylpyridine precursor instead of constructing this biaryl structure using a Suzuki–Miyaura reaction in pathway 1 and 2 shown in Fig. 6c. This demonstrates that the tree-LSTM model, despite being trained for pathway ranking, can encode pathways from a retrosynthetic design perspective giving the opportunity to use this learned pathway encoding for clustering purpose.

### Limitations and frontiers

The tree-LSTM model was demonstrated to understand strategic retrosynthesis design and cluster strategically similar pathways. Nevertheless, due to limitations in data labelling, the tree-LSTM model was trained to differentiate patent-extracted pathway and artificial pathways, with the assumption that patent-extracted pathways should be considered more strategic than artificial pathways. Thus, the model, to a certain extent, will ignore creative artificial pathways with comparable or improved strategic levels compared to the patent-extracted pathways. In addition, since the sources of patent-extracted and artificial pathways are different, certain data discrepancy (*e.g.* appearance frequency of different reaction types) may exist, biasing the model towards patterns that appear more frequently in patent-extracted pathways than in artificial pathways. Looking forward, these limitations can be mitigated by (1) having multiple patent-extracted pathways (currently only one) for the same target molecule, (2) having more accurate and richer labelling of different pathway designs, and (3) having more examples with tactical retrosynthesis designs (*e.g.* the use of directing groups).

The current tree-LSTM model does not explicitly evaluate the plausibility or selectivity of each single-step reaction. However, there have been many models developed for examining single-step reactions,^35–38^ and the pathways fed into the tree-LSTM model can be pre-evaluated with those models. Thus, we decided to omit single-step evaluation and only focus on overall strategic relationship of all singe-step reactions in the pathway.

Furthermore, this work relied on the Pistachio patent dataset that was extracted using natural language processing algorithm (NLP) by Nextmove. Despite that data was deeply cleaned and curated with the state-of-the-art atom mapping algorithm, the potential data quality issue may still mislead the tree-LSTM model to using some minor features that have never appeared in the artificial pathways for ranking. Thus, using high-quality or even human-curated pathway dataset can further refine the model's ability of understanding the retrosynthesis design strategies.

## Conclusions

This work implemented a tree-LSTM neutral network structure to encode pathway-level retrosynthesis design information. In order to facilitate learning how chemists design synthetic routes in practice, we curated a retrosynthesis pathway database from the single-step patent reaction database. For each target molecule in the pathway, 5–300 artificial pathways were generated by the ASKCOS program. The tree-LSTM model was trained to understand the strategic level of the retrosynthesis pathways *via* ranking patent-extracted retrosynthesis pathways higher than the artificial ones. The model was able to achieve a top-1 ranking accuracy of 79.1%, which significantly outperformed the other three heuristic baseline models. Case studies on the correctly and incorrectly ranked results showed that tree-LSTM model was indeed able to recognize strategic synthesis designs and penalize nonproductive or non-selective reaction sequences. The trained tree-LSTM model can also serve as a tool to cluster pathways with strategically similar designs by encoding the pathway into a learned pathway embedding, so that users can focus on strategically different pathways proposed by the retrosynthesis program.

## Methods and data

The reaction database used in this work is the Pistachio patent database from NextMove (v3.0 released in June 2019). All scripts were written in Python 3.7. RDKit^31^ was used for molecule/reaction parsing, molecular fingerprint conversion, and various cheminformatics calculations. PyTorch 1.4 (ref. 39) was used for building the machine learning architectures. See ESI† for detailed model structures and training procedures. All code used in this work can be found on GitHub.^40^ The patent-extracted pathway dataset can be provided upon request with a valid Pistachio license. The pathway dataset generated by ASKCOS is available on Figshare.^41^

## Conflicts of interest

There are no conflicts to declare.

## Supplementary Material

SC-012-D0SC05078D-s001

SC-012-D0SC05078D-s002

SC-012-D0SC05078D-s003
