# Supplementary material for: Evaluating and clustering retrosynthesis pathways with learned strategy
Source: Chem Sci. 2020 Nov 23;12(4):1469–78. doi: 10.1039/d0sc05078d (PMC8179211; doi:10.1039/d0sc05078d)
Supplement: SC-012-D0SC05078D-s001 [file SC-012-D0SC05078D-s001.pdf]

Electronic Supplementary Material (ESI) for Chemical Science.
This journal is © The Royal Society of Chemistry 2020

Supporting Information

Evaluating and clustering retrosynthesis pathways with learned strategy

Yiming Mo,‡[a, b, c] Yanfei Guan,‡[a] Pritha Verma,[a] Jiang Guo,[d] Mike E. Fortunato,[a] Zhaohong Lu,[e] Connor W. Coley,[a] and Klavs F. Jensen*[a]

[a]Department of Chemical Engineering, Massachusetts Institute of Technology, Cambridge, Massachusetts 02139, United States

[b]College of Chemical and Biological Engineering, Zhejiang University, Hangzhou, Zhejiang Province 310007, China.

[c]ZJU-Hangzhou Global Scientific and Technological Innovation Center, Hangzhou, Zhejiang Province 311215, China.

[d]Computer Science and Artificial Intelligence Laboratory, Massachusetts Institute of Technology, Cambridge, Massachusetts 02139, United States

[e]Department of Chemistry, Massachusetts Institute of Technology, Cambridge, Massachusetts 02139, United States

Table of contents

1. General information: data and code 2
2. Detailed tree-LSTM model structure and training 4
3. Baseline model details 8
4. Reference 13
5. Appendix 14

1. General information: data and code

The reaction database used this work is Pistachio patent database from NextMove (released in June 2019)[1]. All scripts were written in Python 3.7. RDKit[2] was used for molecule/reaction parsing, molecular fingerprint conversion, and various cheminformatics calculations. PyTorch 1.4[3] was used for building the machine learning architectures. The ASKCOS used in this work was an in-house development version, however, the open-source version[4] can also be used.

1.1 Pistachio patent pathway extraction process

As discussed in the manuscript, single-step reactions were grouped by patent number. Each group only contains single-step reactions from the same patent, and then, connecting single-step reactions via matching the canonical SMILES strings of products and reactants to construct the reaction network. The reagents (compounds that do not contribute atoms to the products) are neglected in the network. The example network is shown in Fig. 1a in the main text. Within this network, compounds that only appeared as product and not appeared as reactants were first identified as the root nodes. And then with a complete depth-first search (DFS) algorithm, traversing through the network starting from the root nodes will give all the retrosynthesis pathways embedded in the reaction network. However, when cyclic patterns (appearing infrequently when grouping single-step reactions within a single patent) exist in the reaction network, pathways with infinite depth can be found, and a maximum depth of 20 was set to avoid finding cyclic pathways.

1.2 ASKCOS pathway generation process

ASKCOS retrosynthesis program uses template-based method to recursively apply single-step reaction templates until all precursors can be purchased. A Monte Carlo tree search (MCTS) algorithm is implemented during this tree search to improve success rate. At each step, multiple templates will be applied, and priority of templates for further exploration will depend on the trained ASKCOS retrosynthesis model and MCTS algorithm. Even for compounds that have appeared in the database, ASKCOS can still give a variety of pathway designs that are significantly different from those presented in the dataset.

We used the ASKCOS program to generate a set of artificial retrosynthesis pathways for each target compound. The maximum depth allowed for the ASKCOS generated pathways for one specific target molecule is two plus the depth of its patent pathway to avoid generating unrealistically long pathways (e.g. the depth of the pathway (1) found in Fig. 1a in the main text is 4, and the maximum depth allowed when searching pathways using ASKCOS was 6). We randomly selected 300 artificial pathways from top 3,000 pathways outputted from ASKCOS. (If the number of output pathways was less than 300, all the generated pathways were used. For each target compound, there were at least 5 artificial pathways, otherwise the target compound was dropped from the final pathway database.)

Pathways extracted from Pistachio patent database do not always end with buyable precursors. To keep consistent comparison between Pistachio pathways and ASKCOS-generated pathways, two stop criteria were used when searching retrosynthesis pathways using ASKCOS:

(1) When all the precursors of the pistachio pathway are buyable, the stop criterion is finding pathways with buyable precursors.

(2) When one or more precursors of the Pistachio pathway are not buyable, the stop criterion is finding pathways with precursors that are equally or less complex compared to the most complex precursor in the Pistachio pathway. The complexity of the precursor is measured by number of each type of atoms in the molecule (hydrogen atoms are excluded). For example, $C_6H_5NO_2$ is less complex than $C_7H_5NO_4$.

Additional settings for ASKCOS pathway generation are shown in Table S1.

Table S1. Settings for ASKCOS pathway generation.

**Settings**	**Values**
Searching time	30 s
Maximum branches each node	25
Maximum depth	Depth of Pistachio pathway + 2
Single-step plausibility*	0.75
Minimum non-chiral template frequency§	40
Minimum chiral template frequency§	20
Maximum number of templates used each node†	1000
Maximum cumulative template probability†	0.9999

*Single-step reaction plausibility was evaluated by in-scope filter[5]. §The reaction template frequency counts used in published literatures and patents were updated in 2017. †These settings are for template relevance model[6].

2. Detailed tree-LSTM model structure and training

Model structure

As illustrated in Figure 2B in the manuscript, the tree-LSTM model is a dynamic model structure, and the structure is determined by the structure of the retrosynthesis pathway. Each node in the tree is a reaction in the pathway, and the reaction is encoded using product fingerprint and reaction fingerprint. The reaction fingerprint is calculated using the following formula[7]:

$$reactionFP = w\left(\sum_i ProductFP_i - \sum_i ReactantFP_i\right) + (1-w)\sum_i AgentFP_i$$

Since the model focuses on the retrosynthesis strategy instead of the single-step reaction plausibility, the reagent information is ignored in the reaction fingerprint. Thus, w was set to 1, and the reaction fingerprint was calculated as follows:

$$reactionFP = \sum_i ProductFP_i - \sum_i ReactantFP_i$$

The fingerprints for products and reactants are 2048-bit Morgan circular fingerprints (equivalent to ECFP4[8]) with a radius of 2. The sum operation of fingerprints will be followed by a compression operation to compress positive values on bits to one (zero bits will remain as zero). The reason for using product fingerprints and reaction fingerprints as the input is making the model aware of the structural information of each intermediate and its corresponding reaction performed. This information is critical in understanding the strategic connections of single-step reaction in the pathway.

The reaction encoder model structure is shown in Figure S1. Product fingerprint and reaction fingerprint passed through its own 2-layer neural network, and then the two outputs were aggregated using an element-wise multiplication operation to the reaction embedding of the singe-step reaction.

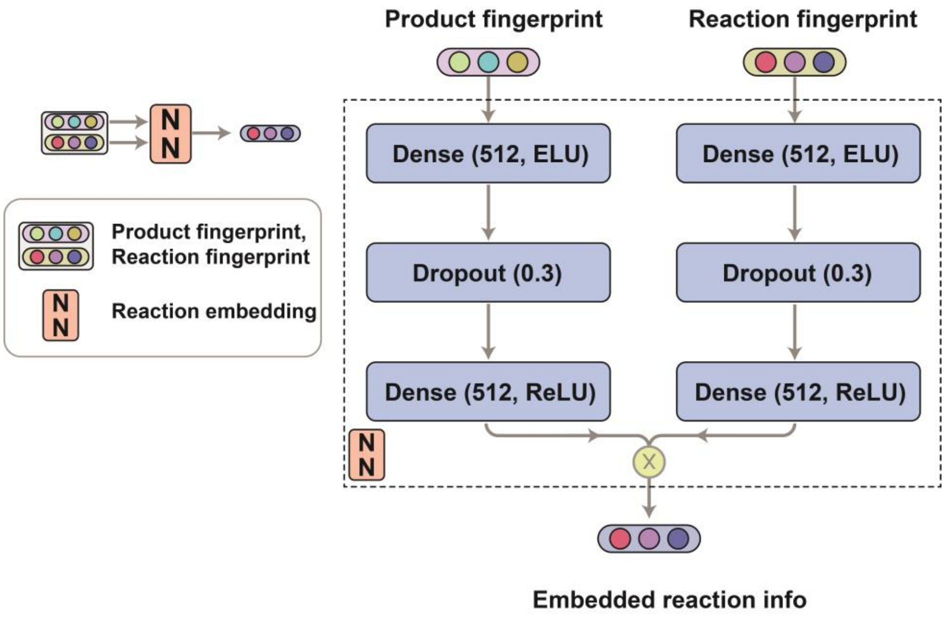

Figure S1. The structure of the reaction encoding model.

The LSTM node uses the following formula to process the input and hidden states from the child nodes[9]:

$$\tilde{h}_t = \sum_{k \in C(j)} h_k$$

$$i_t = \sigma(W^{(i)} x_t + U^{(i)} \tilde{h}_t + b^{(i)})$$

$$f_{tk} = \sigma(W^{(f)} x_t + U^{(f)} h_k + b^{(f)})$$

$$o_t = \sigma(W^{(o)} x_t + U^{(o)} \tilde{h}_t + b^{(o)})$$

$$u_t = tanh(W^{(u)} x_t + U^{(u)} \tilde{h}_t + b^{(u)})$$

$$c_t = i_t \odot u_t + \sum_{k \in C(j)} f_{tk} \odot c_k$$

$$h_t = o_t \odot \tanh(c_t)$$

where x_t is the input at the current node, σ denotes logistic sigmoid function, and $\odot$ denotes elementwise multiplication. Additionally, we defined i_t as the input gate, f_t as the forget gate, o_t as the output gate, c_t as memory cell state, and h_t as the hidden state. $\tilde{h}_t$ is a sum of hidden states from the child nodes. The tree-LSTM unit contains one forget gate f_{tk} for each child k. W, U and b are weights and biases for each transition, respectively.

The tree-LSTM model was adopted from this GitHub repository[10] for accelerated computation and training speed compared to the original publication[9].

The hidden state of the root node was used to represent the retrosynthesis pathway since this latent vector contains all information of the whole pathway. This latent vector was then passed through a scorer network to give a strategic level score for this pathway. The scorer network structure is shown in Figure S2.

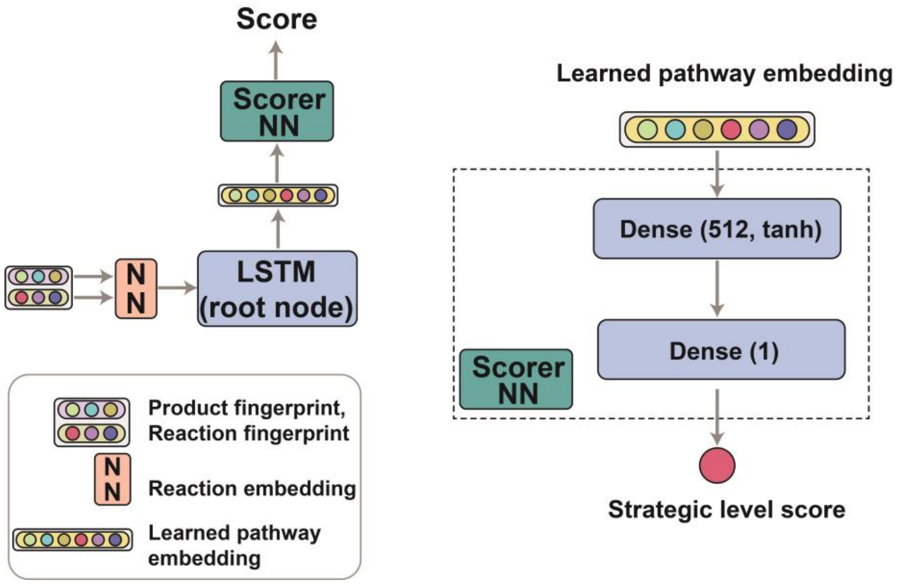

Figure S2. The structure of the reaction encoding model.

Model training procedure

As discussed in the main text, the score given by the tree-LSTM model is a relative score that is used compare pathways with the same target compound. The model parameters are trained to maximize the ranking accuracy when comparing the Pistachio pathway and its corresponding ASKCOS-generated pathways. Thus, the loss function is a cross-entropy loss using the probability distribution from softmax activation of the scores. For each Pistachio pathway and its corresponding ASKCOS pathways, the loss can be calculated as follows:

$$y_{Pistachio} = \frac{\exp(Score_{Pistachio})}{\exp(Score_{Pistachio}) + \sum \exp(Score_{ASKCOS,i})}$$

$$loss = -\log(y_{Pistachio})$$

Training was conducted on Nvidia GeForce RTX 2080 Ti Graphics Card. The reaction fingerprints and product fingerprints were precomputed for improved training speed. During training, batches of Pistachio pathways and ASKCOS pathways are fed into model with batch size of 32 (32 is the suitable for 11 GB GPU memory size, and batch size can be bigger if larger GPU memory is available). The loss of the batch is an average of the individual loss of each Pistachio pathway and its corresponding ASKCOS pathways. With 80%, 10%, 10% split of the pathway dataset, the training generally took ~10 hours each epoch for 199,358 records of pathways. We trained the model for 5 epochs with a semi-shuffle of training data (only shuffled the order of batches fed into the model). The evolution of training and validation loss is shown in Figure S3, and evolution of the top-k accuracy during training is shown in Figure S4.

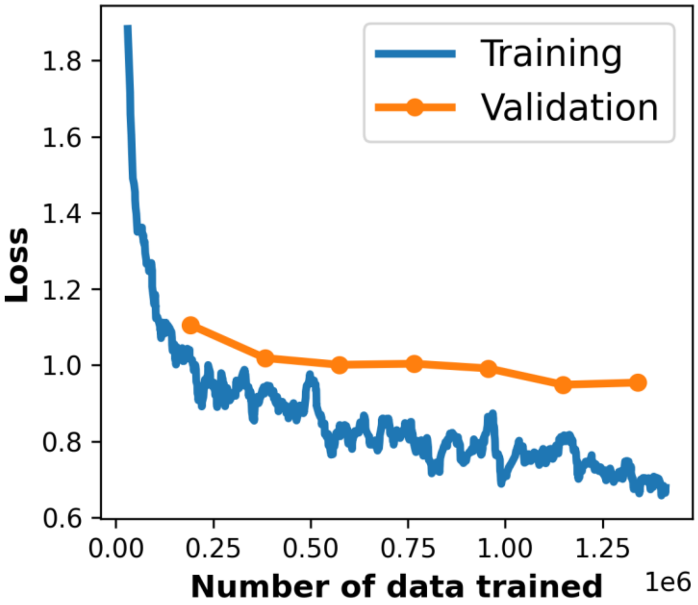

Figure S3. The evolution of training and validation loss during training.
(The x axis is the number of data records that have been fed into the model.)

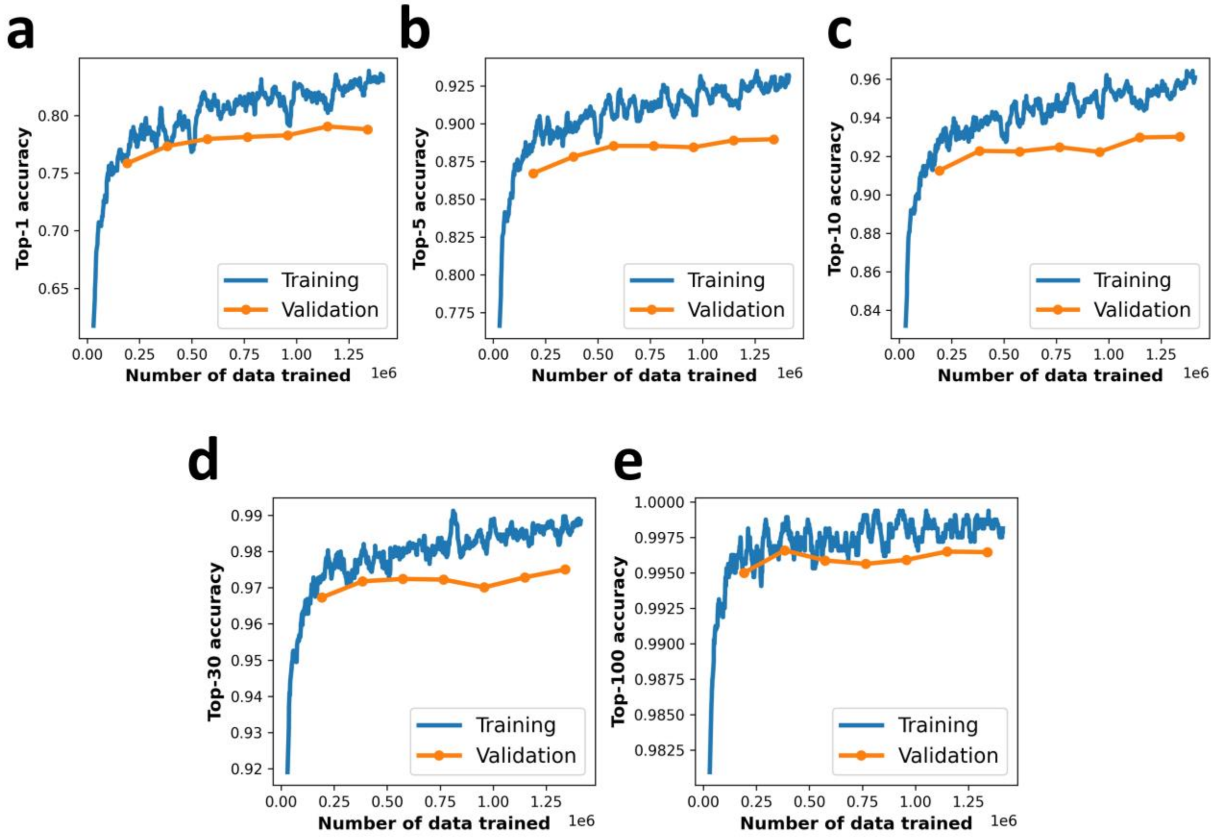

Figure S4. The evolution of training and validation top-k accuracy during training. (a) Top-1 accuracy; (b) Top-5 accuracy; (c) Top-10 accuracy; (d) Top-30 accuracy; (e) Top-100 accuracy. (The x axis is the number of data records that have been fed into the model.)

3. Baseline model details

3. 1 SCScore baseline model

The SCScore baseline model uses the evolution of molecular complexity of intermediates through a retrosynthesis pathway to evaluate its strategic level score (SLScore). Since the pathway can have multiple branches, the model first linearizes the tree-structured retrosynthesis pathway into individual linear pathways via splitting at each branching intermediate node.

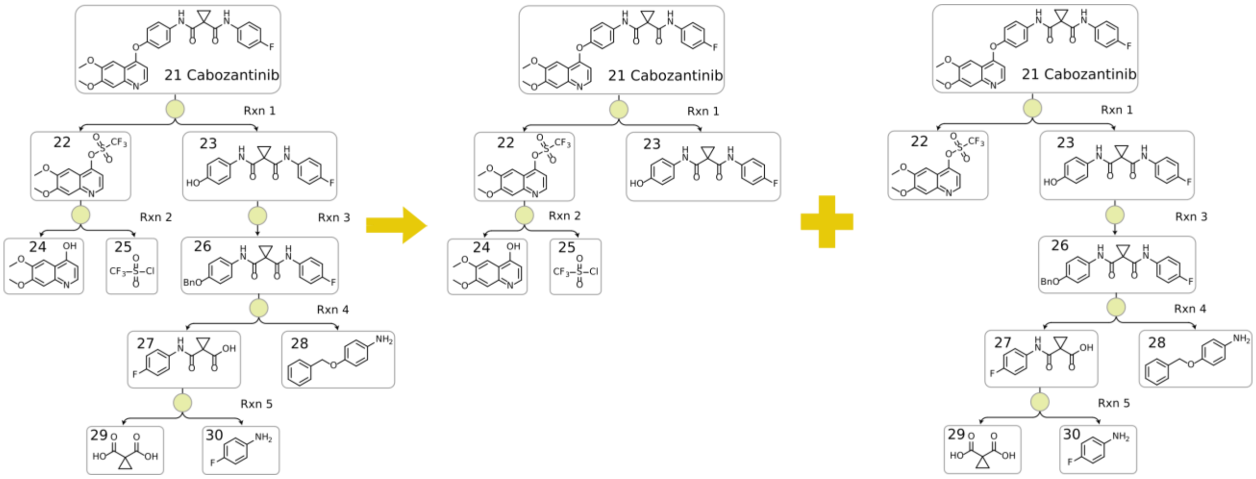

Figure S5. Linearizing the tree-structured retrosynthesis pathway into individual linear pathways. As an example, the tree-structured pathway for cabozantinib **21** synthesis was split into two linear paths at Rxn 1.

For example, the Figure S5 shows the linearization process. Cabozantinib **21** has two precursors, **22** and **23**, and neither **22** nor **23** is a leaf compound. In this case, the tree was split into two individual linear routes at Rxn 1, and the resulting linear routes have only one intermediate compound that is further decomposed for each layer. For each layer, the highest SCScore of the intermediates belonging to this layer was selected to represent complexity of this layer. For example, for the layer 4 of path 2, the SCScore of **27** is 2.26, while the SCScore of **28** is 1.55. Then 2.26 is selected as the complexity index for this layer. The calculated SCScores for each linear path were then put in a vector of length 13 that is used as the input to a neural network. The input vector size of 13 is selected as the maximum depth of paths in the curated database. If the path is shorter than 13, the remaining elements of the input vector will then be capped with 0.

The predicted scores of all linear paths will then be pooled by selecting the minimum score. The training process, including loss function, optimizer, learning rate schedular, are the same as training the tree-LSTM model.

The architecture of the SCScore model is shown in Figure S6. The evolution of training and validation loss is shown in Figure S7, and evolution of the top-k accuracy during training is shown in Figure S8.

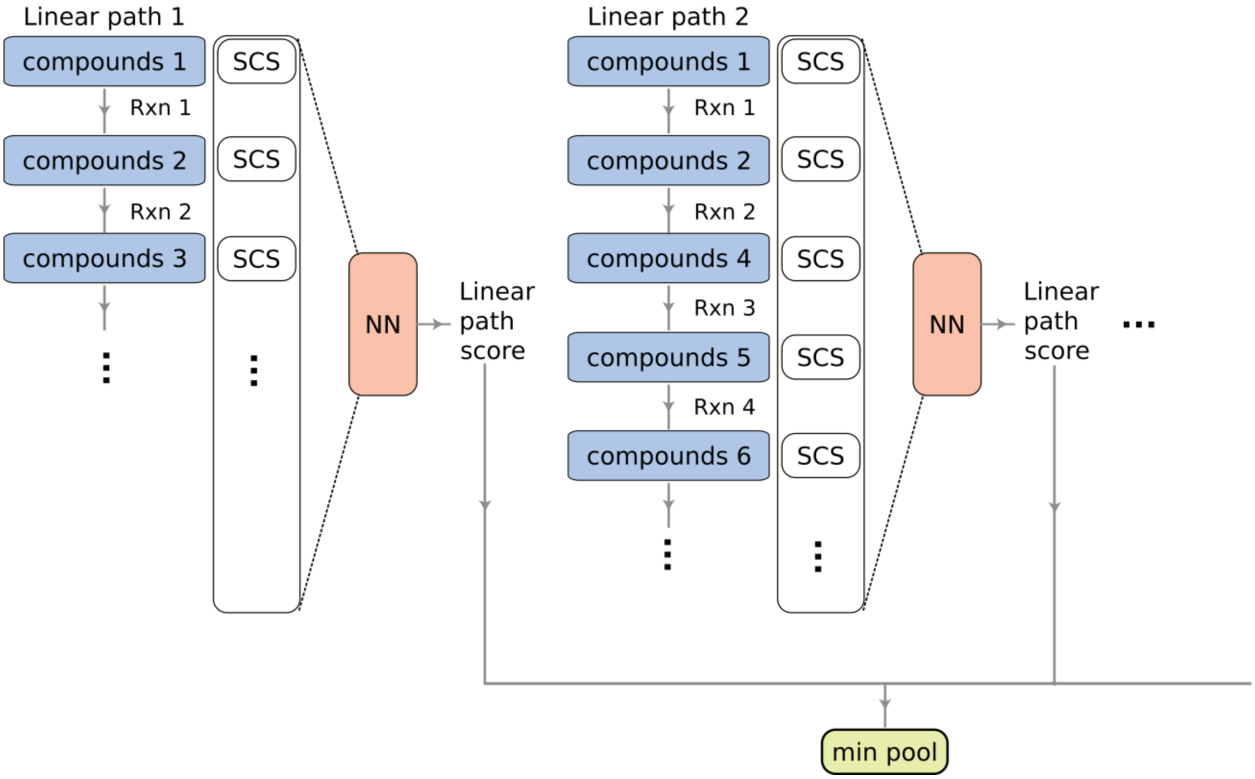

Figure S6. SCScore baseline model architecture.

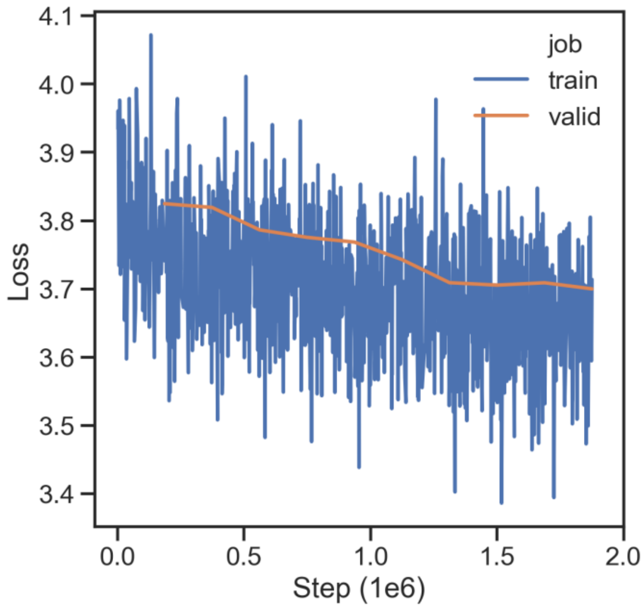

Figure S7. The evolution of training and validation loss during training of SCScore model. (The x axis is the number of data records that have been fed into the model.)

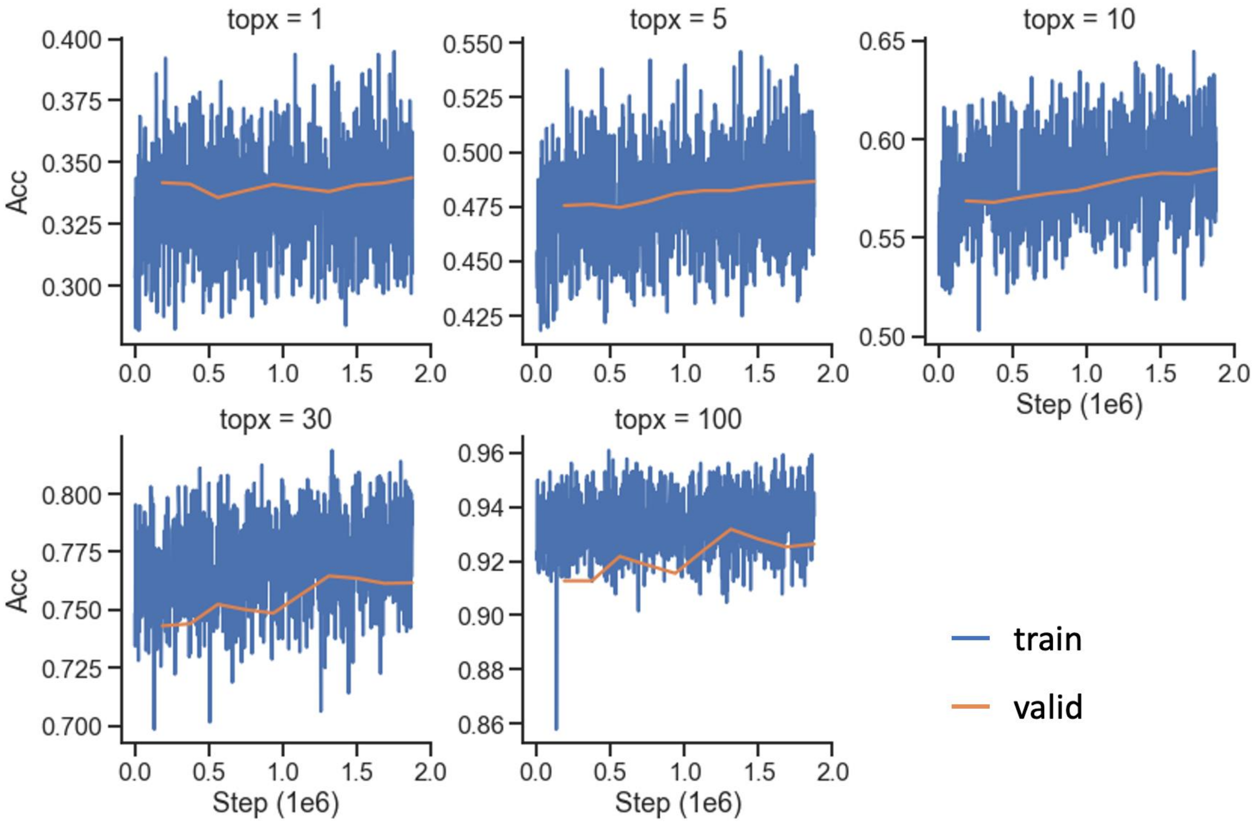

Figure S8. The evolution of training and validation top-k accuracy during training for the SCScore model

3.2 Hybrid model

The Hybrid model uses a set of descriptors depicting the structure of the retrosynthesis tree. The selected descriptors are listed and described in Table S2.

Table S2. Descriptors used for the hybrid baseline model.

Descriptors	Descriptions	Type
Depth	Lenth of the longest linear path	Int
Width	The number of linear pathways composing the retrosynthesis tree path	Int
Number of nodes	Number of intermediate compounds	Int
Number of leaf	Number of leaf compounds	Int
Maximum forks	Maximum number of child nodes appended after the parent node	Int
Target SCScore	SCScore for the target compound	Float [1,5]
Maximum leaf SCScore	Maximum SCScore for leaf comopunds	Float [1,5]
Maximum node SCScore	Maximum SCScore for intermediate compounds	Float [1,5]
Minimum node SCScore	Minimum SCScore for intermediate compounds	Float [1,5]
Maximum SCScore differnece	Maximum difference between the SCScore of product and reactatns for all single step reactions in a tree	Float [1,5]
Minimum SCScore difference	Minimum difference between the SCScore of product and reactatns for all single step reactions in a tree	Float [1,5]

A vector composing the descriptors above are then pass through a neural network to give the final prediction. The training process is the similar to that of tree-LSTM model and SCScore baseline model.

The evolution of training and validation loss is shown in Figure S9, and evolution of the top-k accuracy during training is shown in Figure S10.

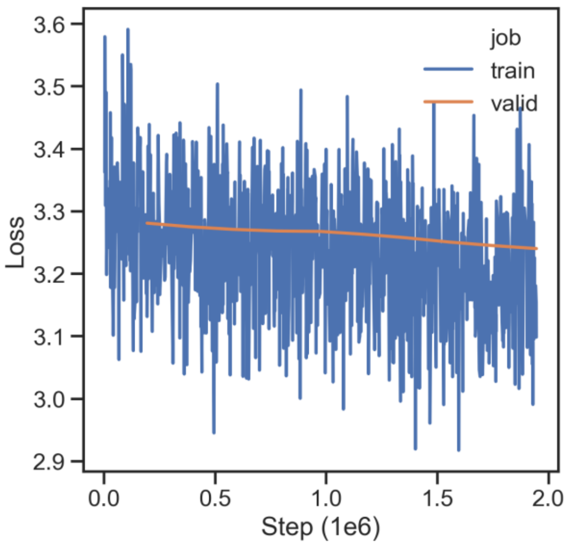

Figure S9. The evolution of training and validation loss during training of hybrid model. (The x axis is the number of data records that have been fed into the model.)

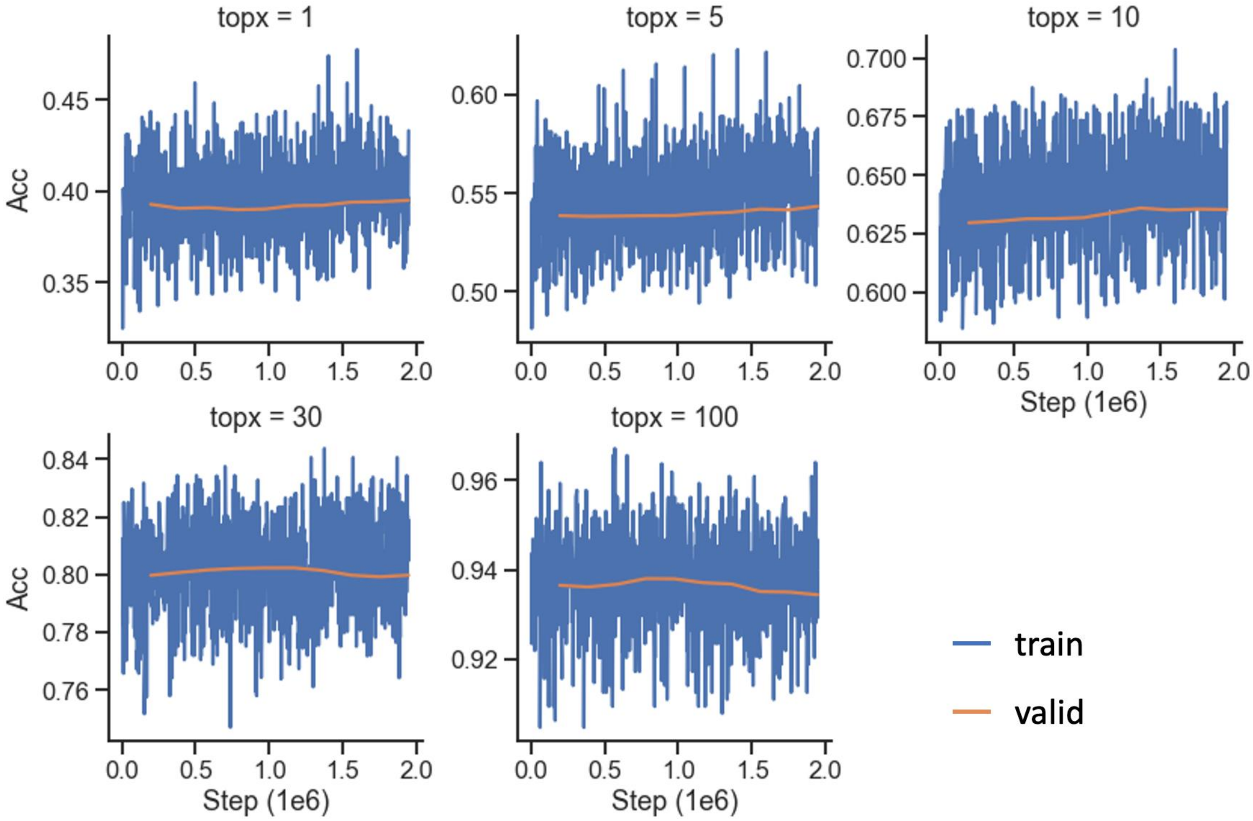

Figure S10. The evolution of training and validation top-k accuracy during training for the hybrid model.

4. Reference

(1) Pistachio (NextMove Software) https://www.nextmovesoftware.com/pistachio.html (accessed Apr 5, 2020).
(2) RDKit http://www.rdkit.org/ (accessed Mar 1, 2020).
(3) PyTorch https://www.pytorch.org (accessed Apr 20, 2020).
(4) ASKCOS https://github.com/connorcoley/ASKCOS (accessed Apr 20, 2020).
(5) Segler, M. H. S.; Preuss, M.; Waller, M. P. Planning Chemical Syntheses with Deep Neural Networks and Symbolic AI. *Nature* **2018**, *555* (7698), 604–610. https://doi.org/10.1038/nature25978.
(6) Segler, M. H. S.; Waller, M. P. Neural-Symbolic Machine Learning for Retrosynthesis and Reaction Prediction. *Chem. – Eur. J.* **2017**, *23* (25), 5966–5971. https://doi.org/10.1002/chem.201605499.
(7) Schneider, N.; Lowe, D. M.; Sayle, R. A.; Landrum, G. A. Development of a Novel Fingerprint for Chemical Reactions and Its Application to Large-Scale Reaction Classification and Similarity. *J. Chem. Inf. Model.* **2015**, *55* (1), 39–53. https://doi.org/10.1021/ci5006614.
(8) Rogers, D.; Hahn, M. Extended-Connectivity Fingerprints. *J. Chem. Inf. Model.* **2010**, *50* (5), 742–754. https://doi.org/10.1021/ci100050t.
(9) Tai, K. S.; Socher, R.; Manning, C. D. Improved Semantic Representations From Tree-Structured Long Short-Term Memory Networks. *ArXiv150300075 Cs* **2015**.
(10) Pytorch implementation of the child-sum Tree-LSTM model https://github.com/unbounce/pytorch-tree-lstm (accessed Apr 20, 2020).

5. Appendix

Randomly selected examples of pathway ranking can be found in the "*SI pathway ranking examples.pdf*" file in the supplementary information. For illustration purpose, ASKCOS pathways were first clustered and then the pathway with highest score in the cluster was displayed (maximum 10 clusters are plotted). The machine-readable format of these examples was also provided in the supplementary information.
