# Supplementary material for: Evaluating and clustering retrosynthesis pathways with learned strategy
Source: Chem Sci. 2020 Nov 23;12(4):1469–78. doi: 10.1039/d0sc05078d (PMC8179211; doi:10.1039/d0sc05078d)
Supplement: SC-012-D0SC05078D-s002 [file SC-012-D0SC05078D-s002.pdf]

Electronic Supplementary Material (ESI) for Chemical Science.
This journal is © The Royal Society of Chemistry 2020

Model ranks patent pathway as top-1: Example 1

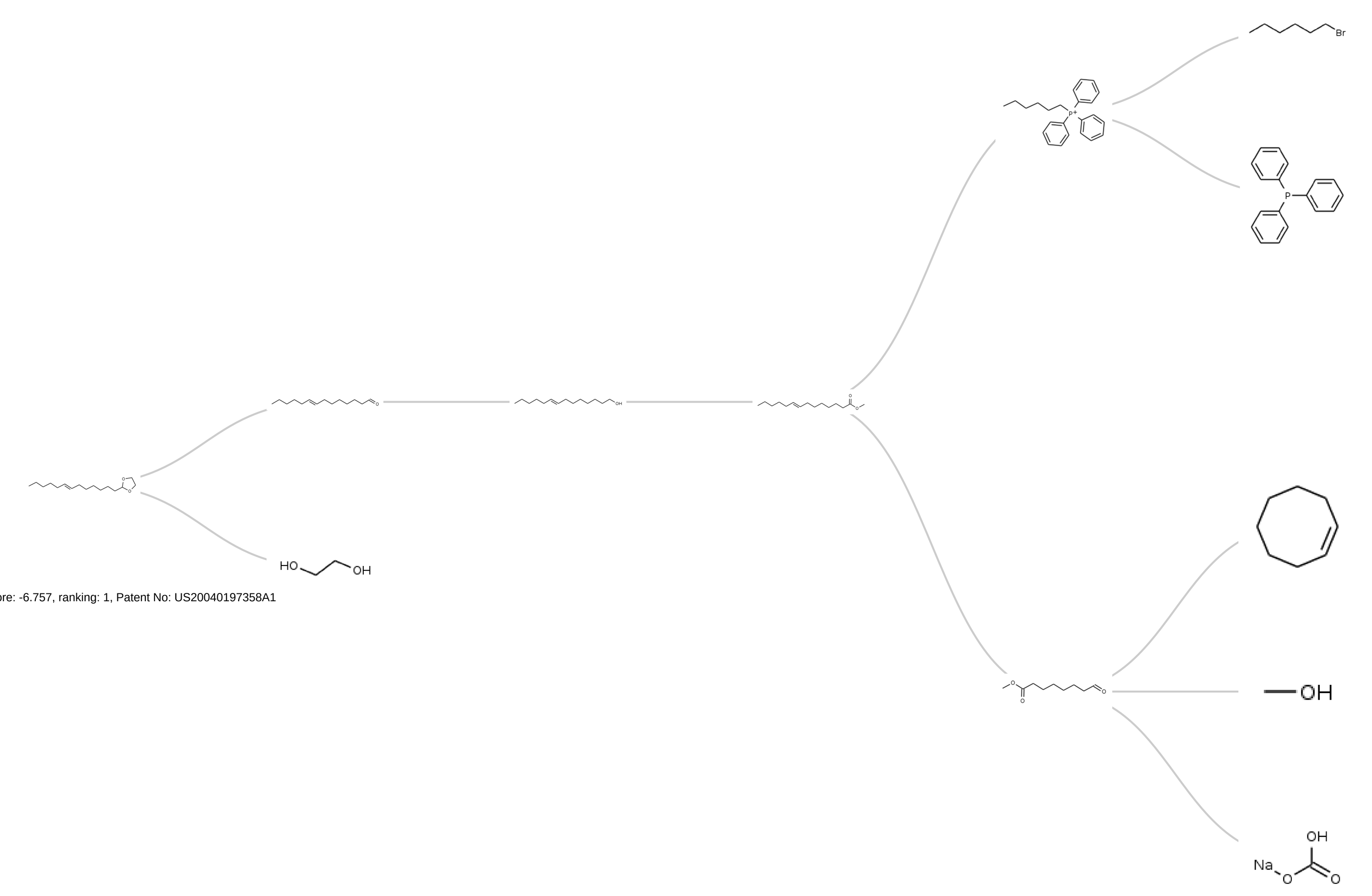

Piatachio pathway, score: -6.757, ranking: 1, Patent No: US20040197358A1

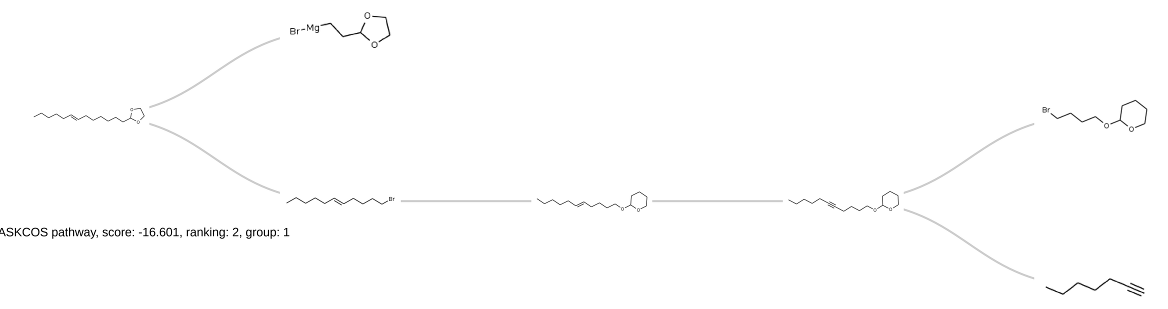

ASKCOS pathway, score: -16.601, ranking: 2, group: 1

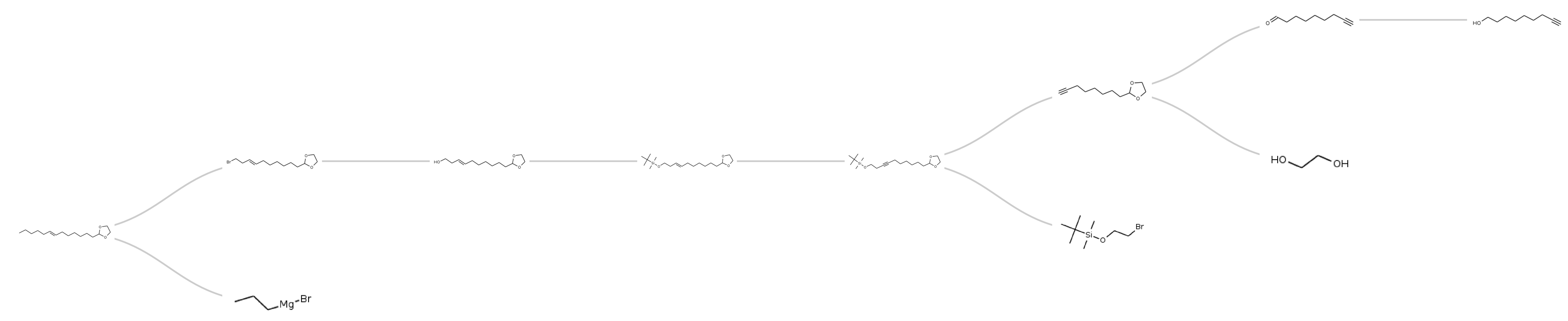

ASKCOS pathway, score: -17.069, ranking: 3, group: 2

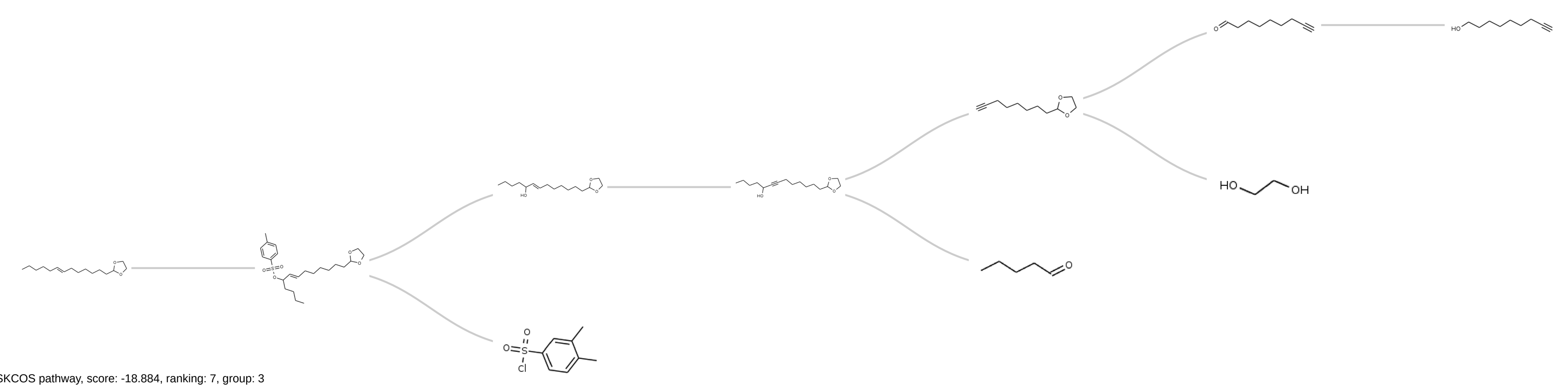

ASKCOS pathway, score: -18.884, ranking: 7, group: 3

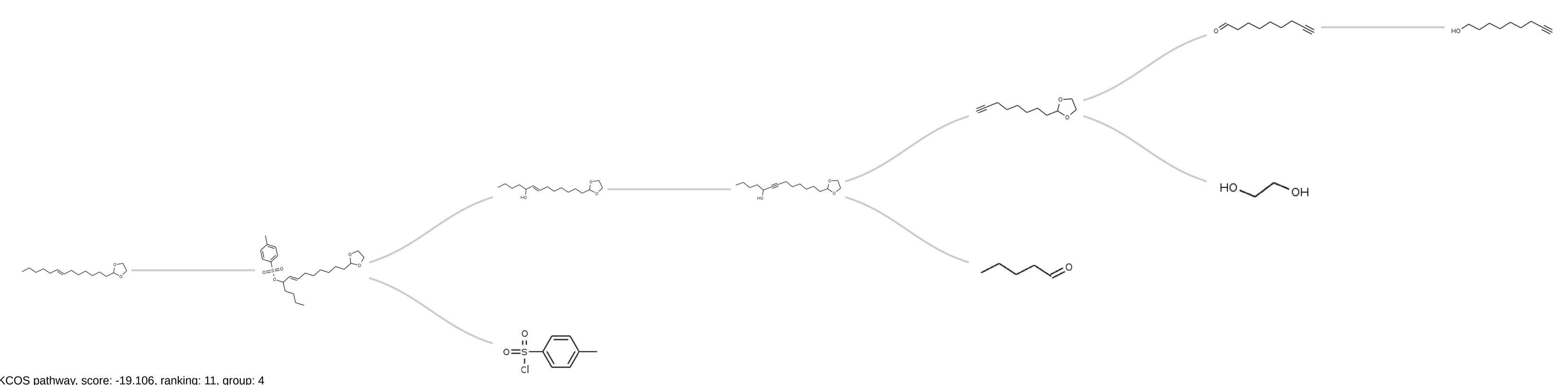

ASKCOS pathway, score: -19.106, ranking: 11, group: 4

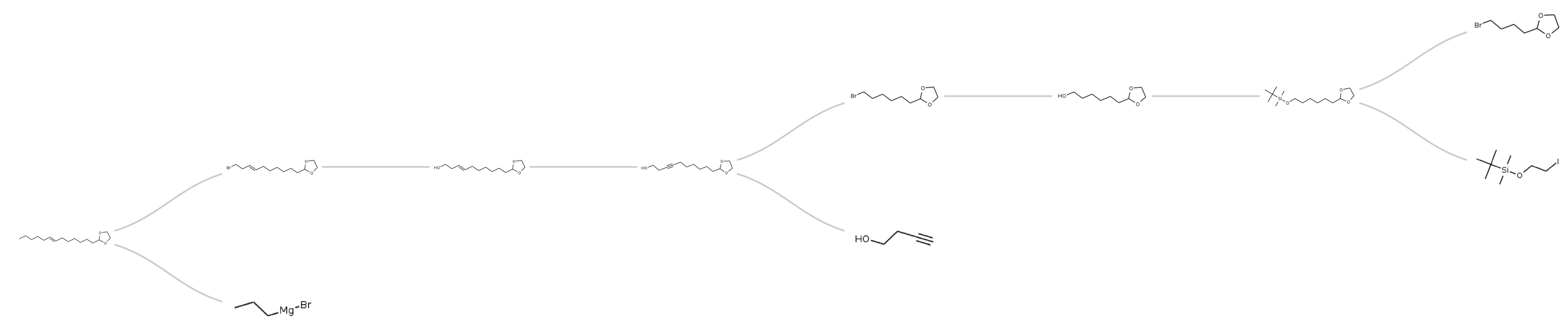

ASKCOS pathway, score: -19.959, ranking: 22, group: 5

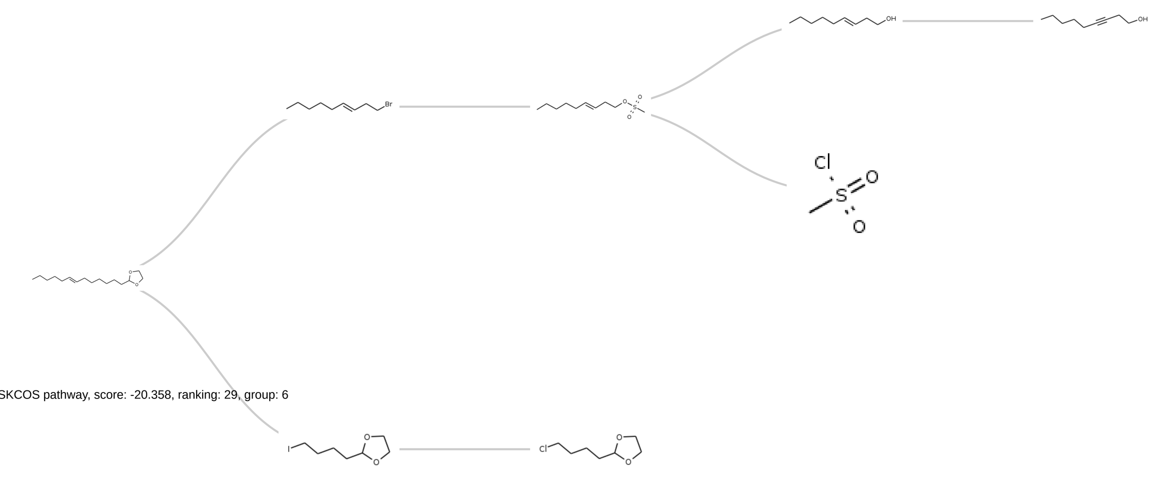

ASKCOS pathway, score: -20.358, ranking: 29, group: 6

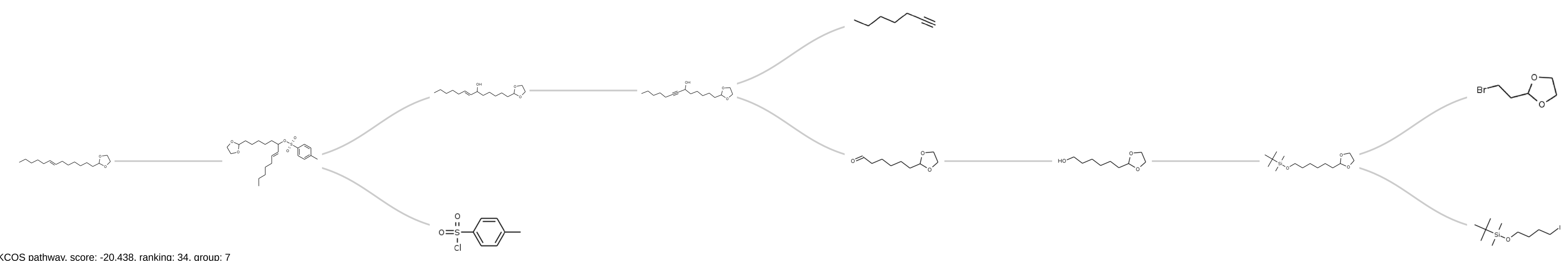

ASKCOS pathway, score: -20.438, ranking: 34, group: 7

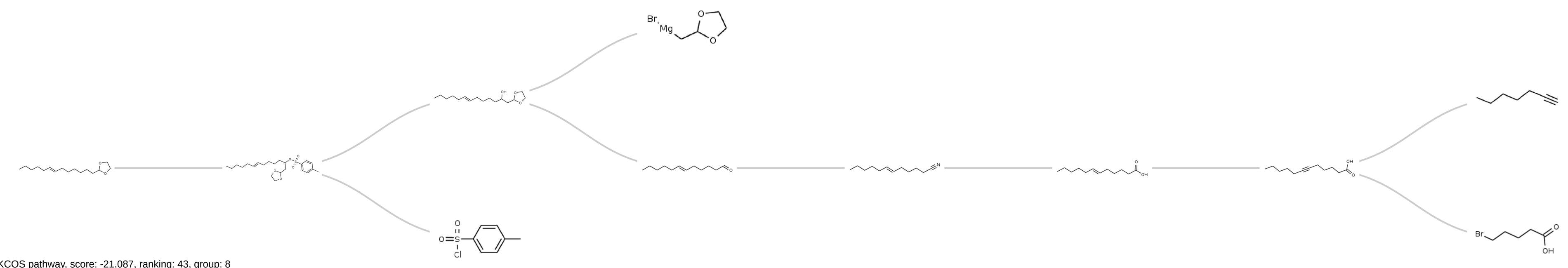

ASKCOS pathway, score: -21.087, ranking: 43, group: 8

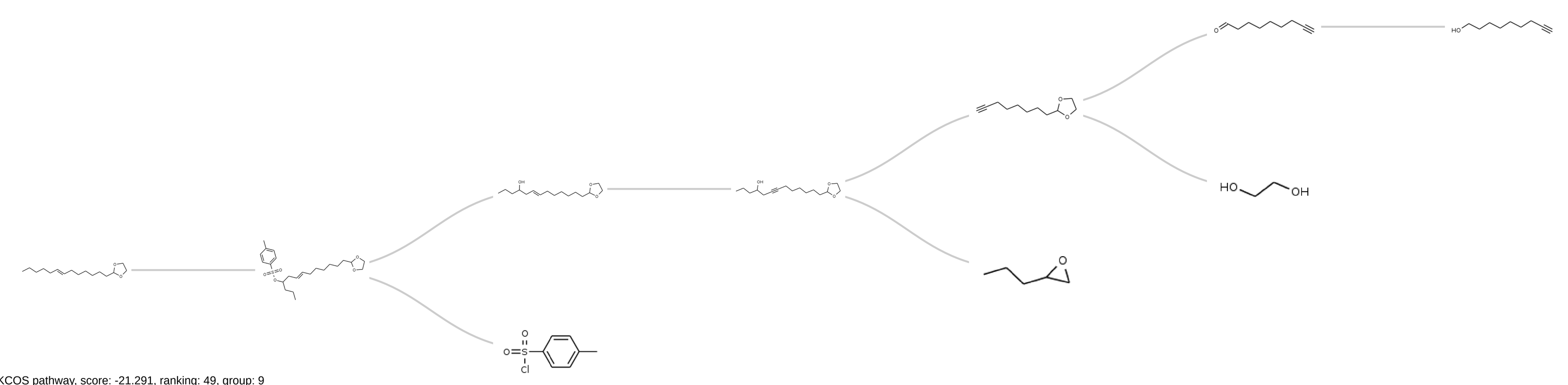

ASKCOS pathway, score: -21.291, ranking: 49, group: 9

Model ranks patent pathway as top-1: Example 2

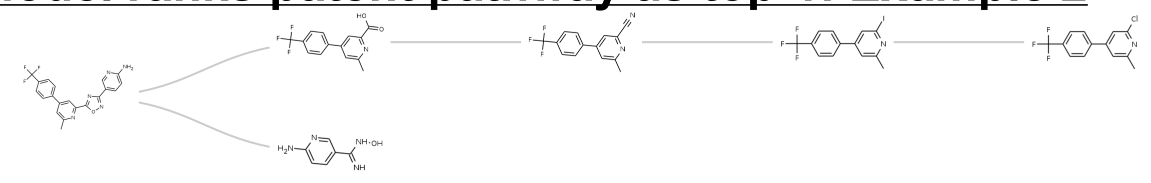

Piatachio pathway, score: -12.477, ranking: 1, Patent No: EP2468727A1

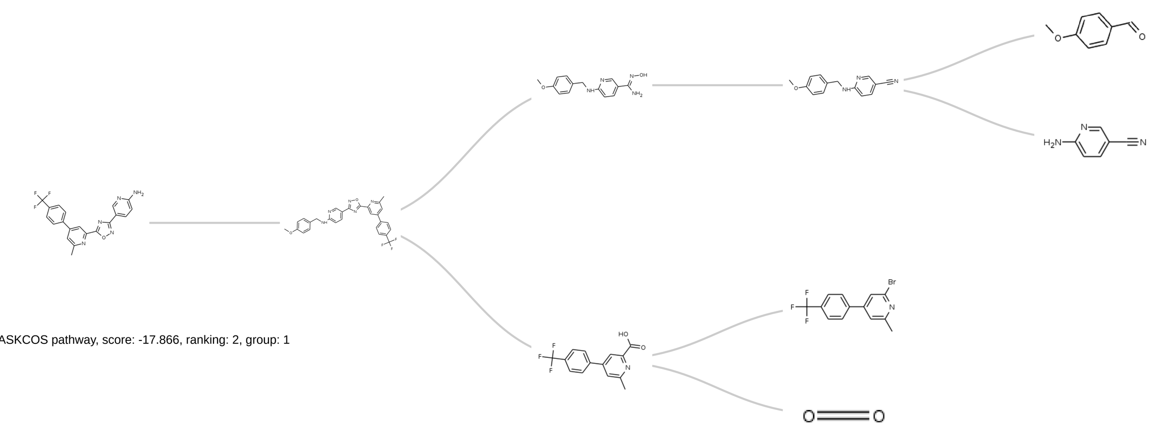

ASKCOS pathway, score: -17.866, ranking: 2, group: 1

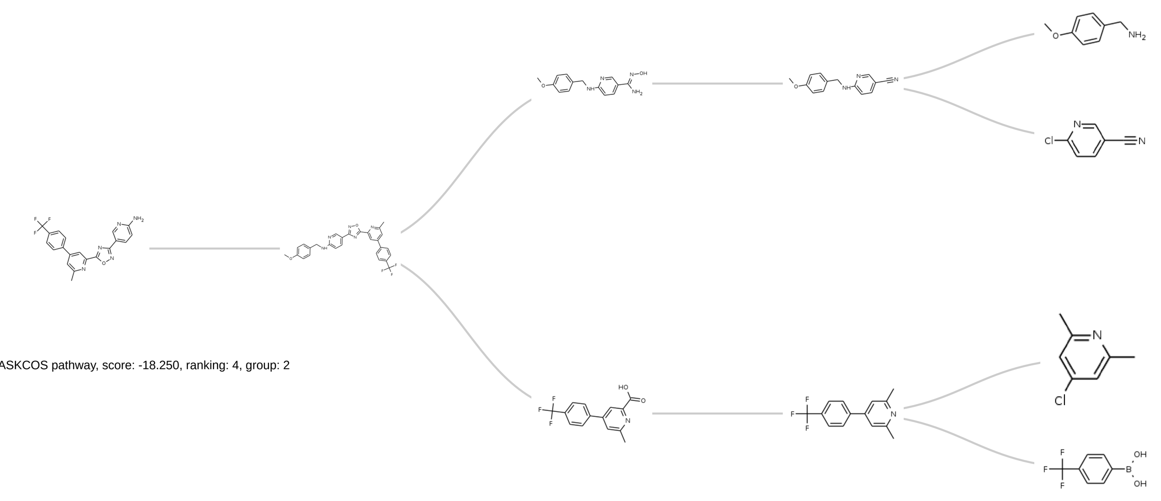

ASKCOS pathway, score: -18.250, ranking: 4, group: 2

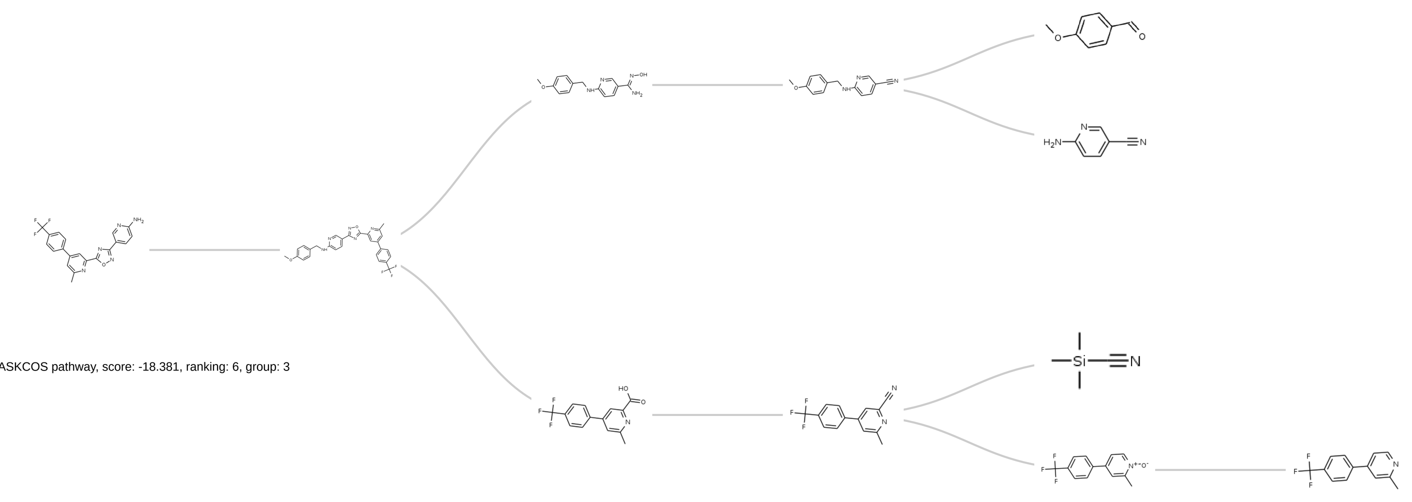

ASKCOS pathway, score: -18.381, ranking: 6, group: 3

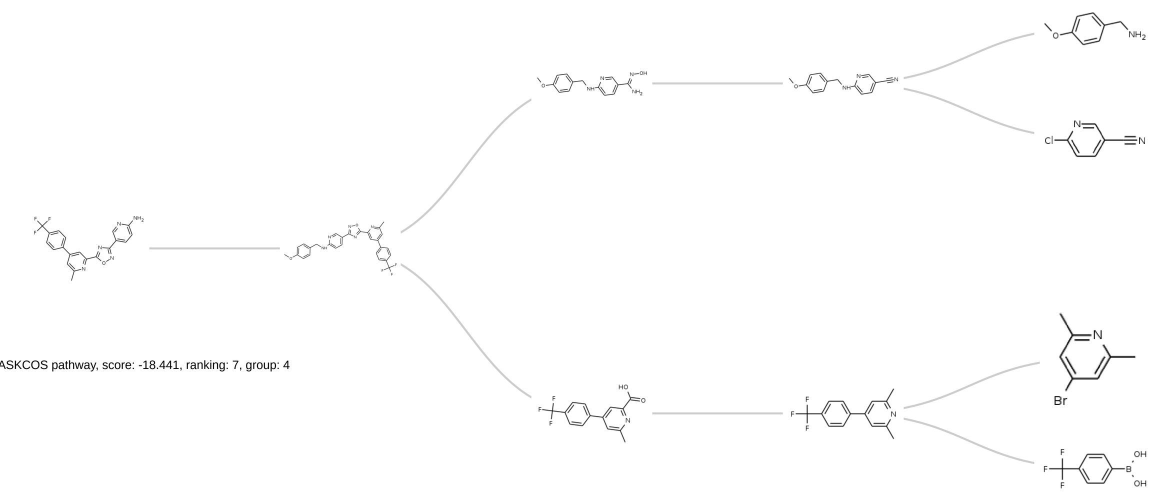

ASKCOS pathway, score: -18.441, ranking: 7, group: 4

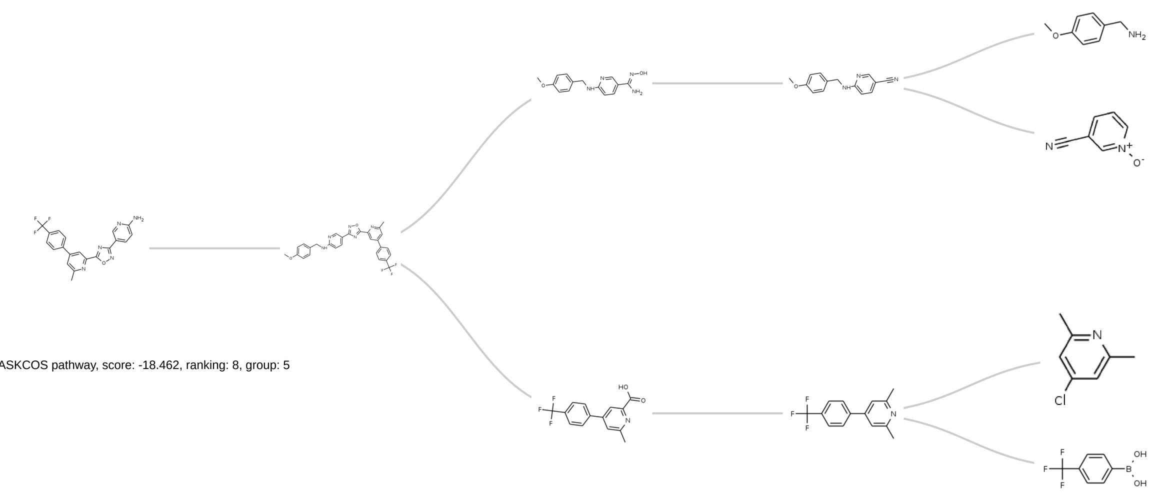

ASKCOS pathway, score: -18.462, ranking: 8, group: 5

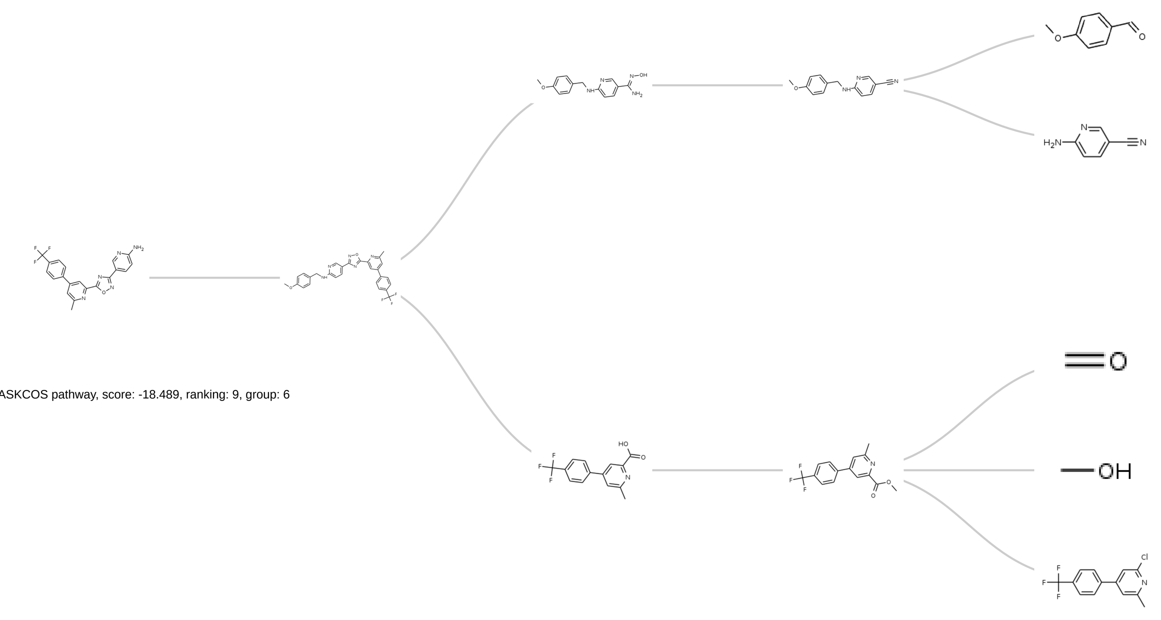

ASKCOS pathway, score: -18.489, ranking: 9, group: 6

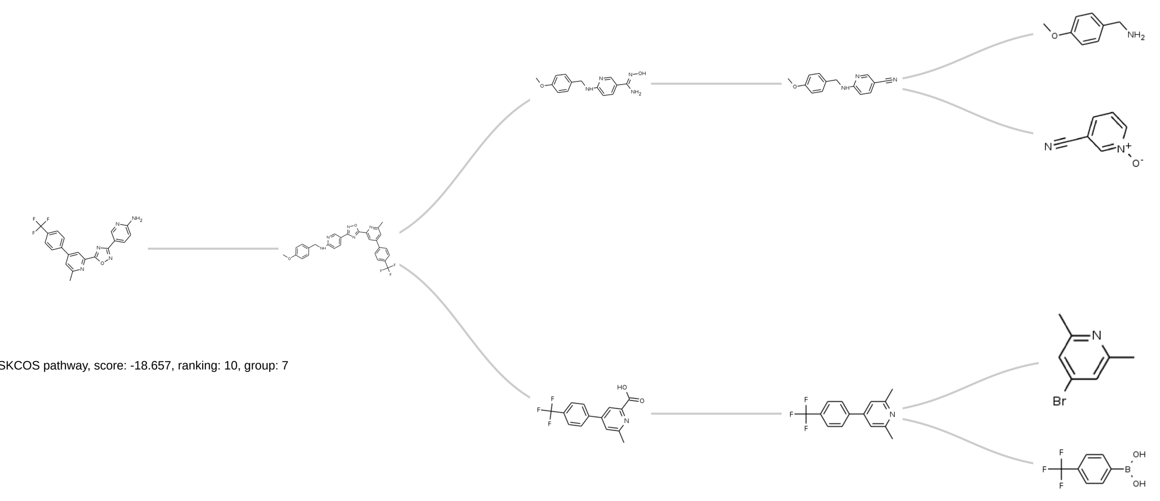

ASKCOS pathway, score: -18.657, ranking: 10, group: 7

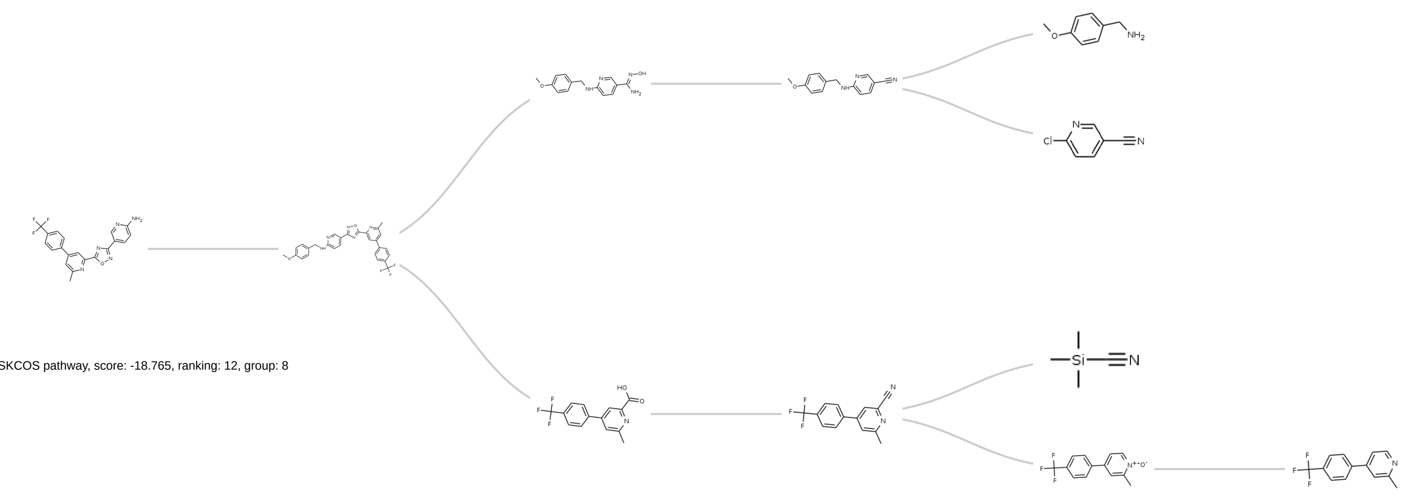

ASKCOS pathway, score: -18.765, ranking: 12, group: 8

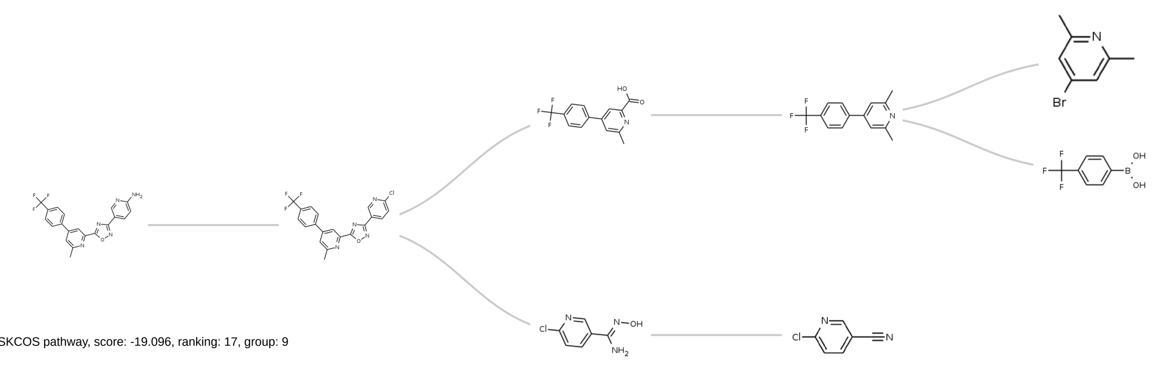

ASKCOS pathway, score: -19.096, ranking: 17, group: 9

Model ranks patent pathway as top-1: Example 3

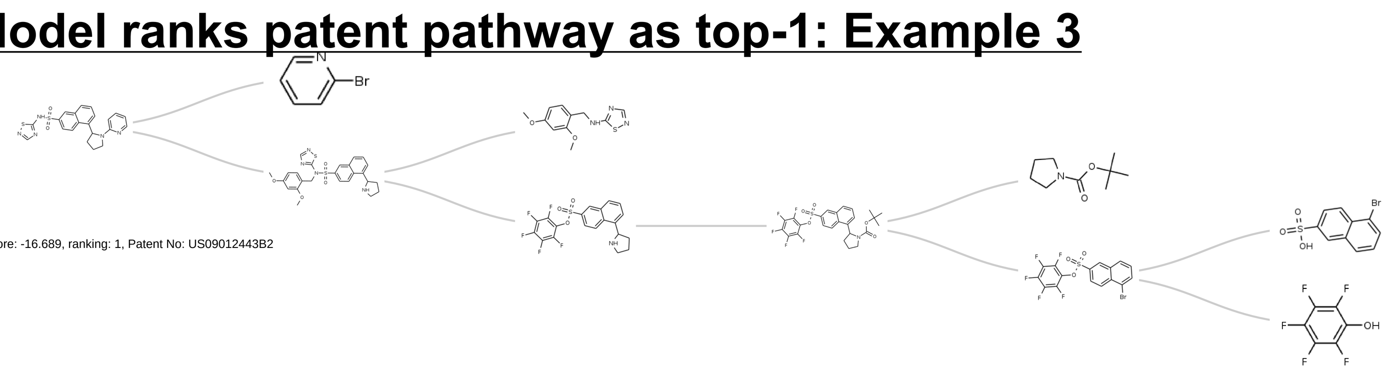

Piatachio pathway, score: -16.689, ranking: 1, Patent No: US09012443B2

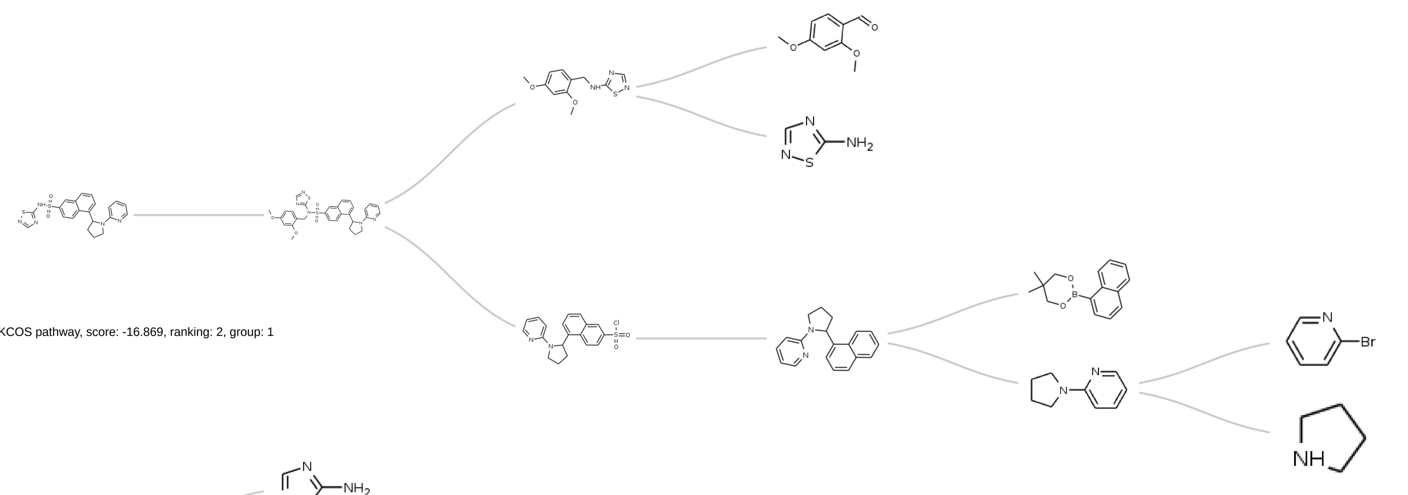

ASKCOS pathway, score: -16.869, ranking: 2, group: 1

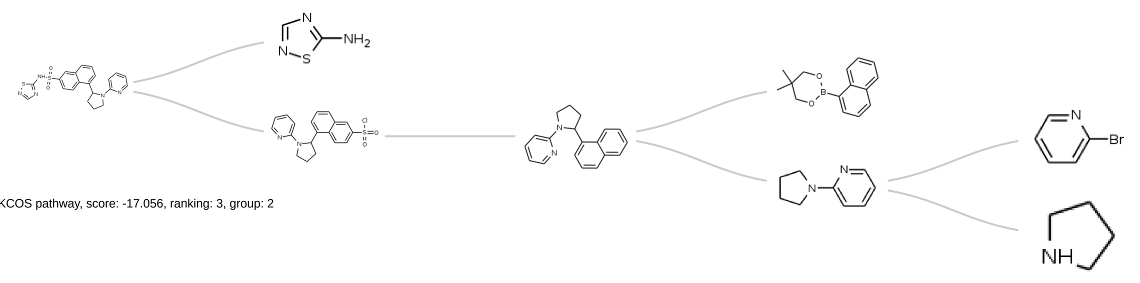

ASKCOS pathway, score: -17.056, ranking: 3, group: 2

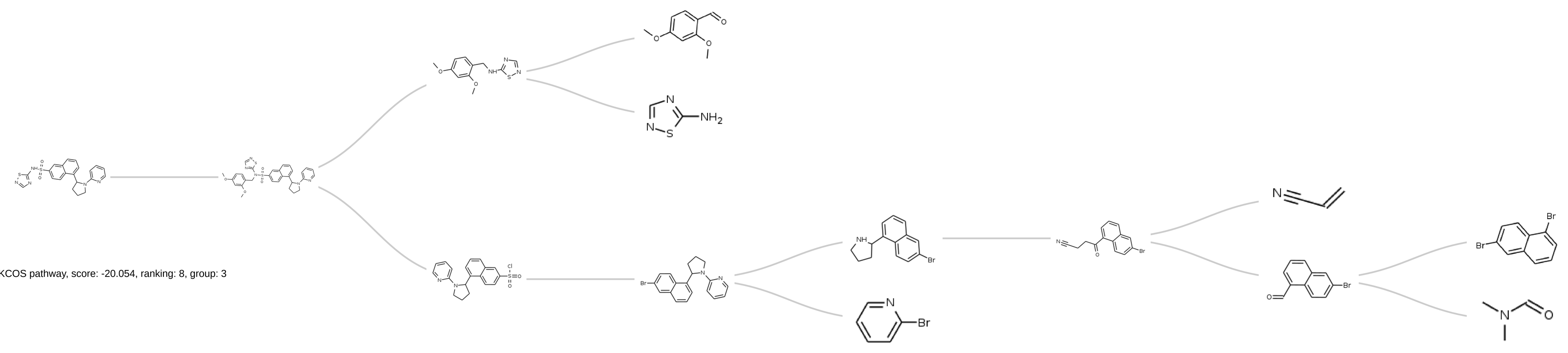

ASKCOS pathway, score: -20.054, ranking: 8, group: 3

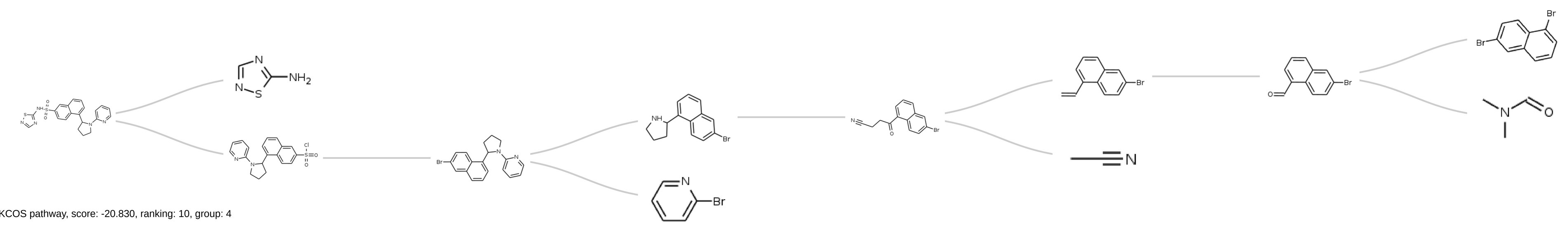

ASKCOS pathway, score: -20.830, ranking: 10, group: 4

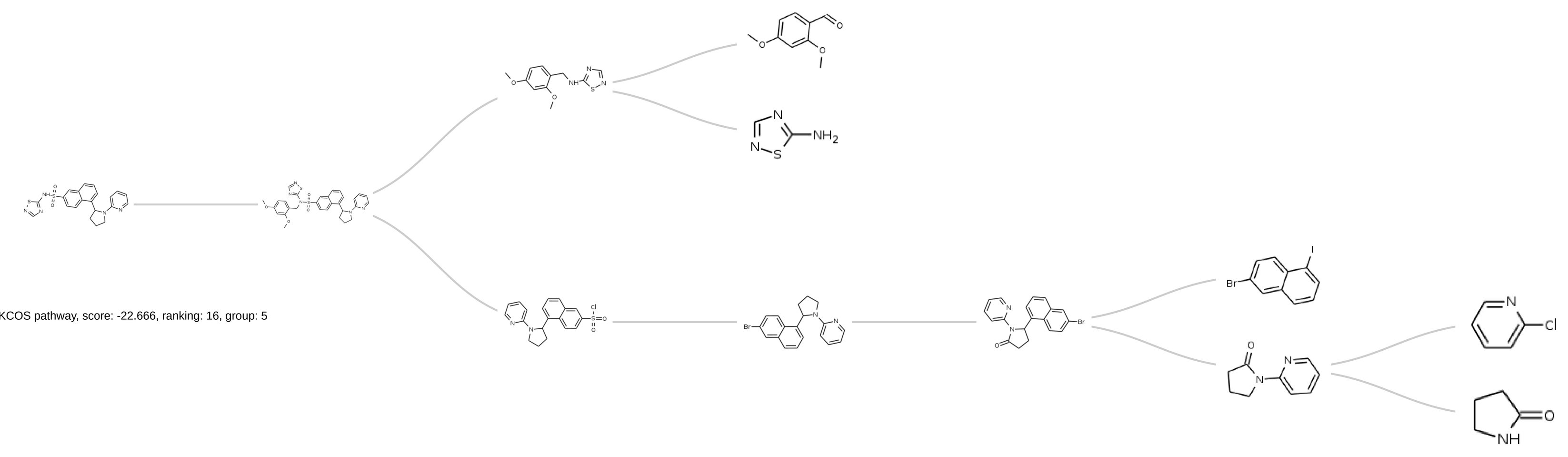

ASKCOS pathway, score: -22.666, ranking: 16, group: 5

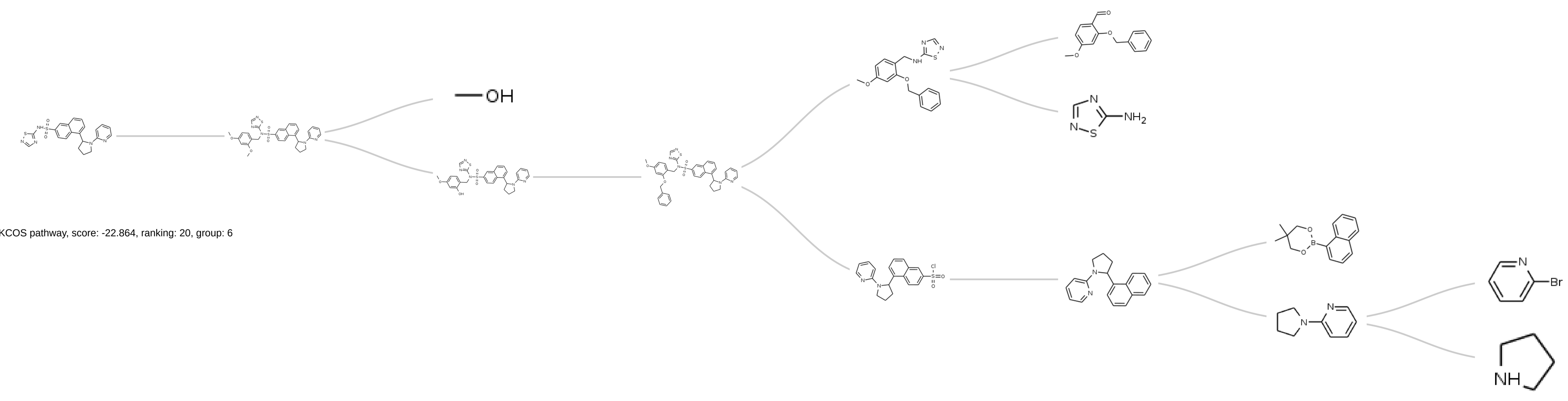

ASKCOS pathway, score: -22.864, ranking: 20, group: 6

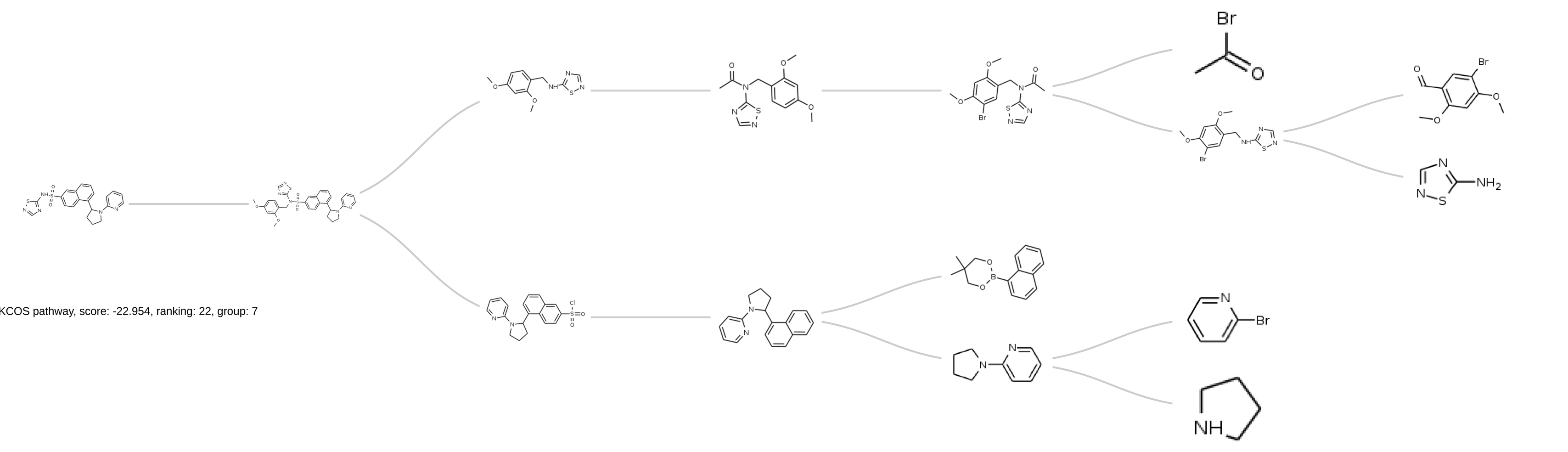

ASKCOS pathway, score: -22.954, ranking: 22, group: 7

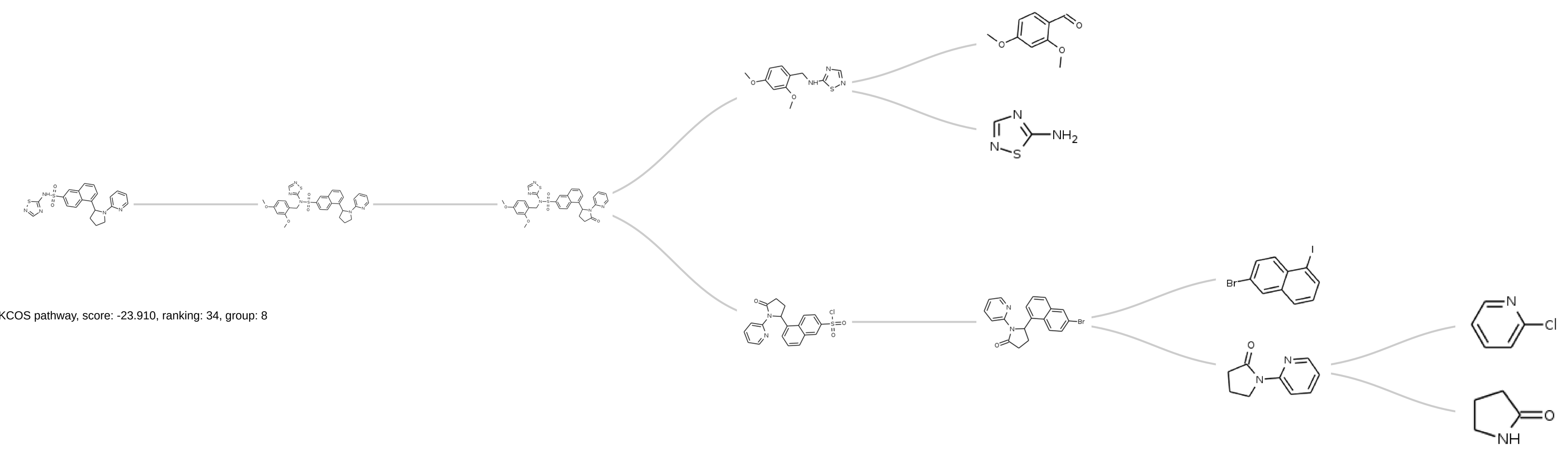

ASKCOS pathway, score: -23.910, ranking: 34, group: 8

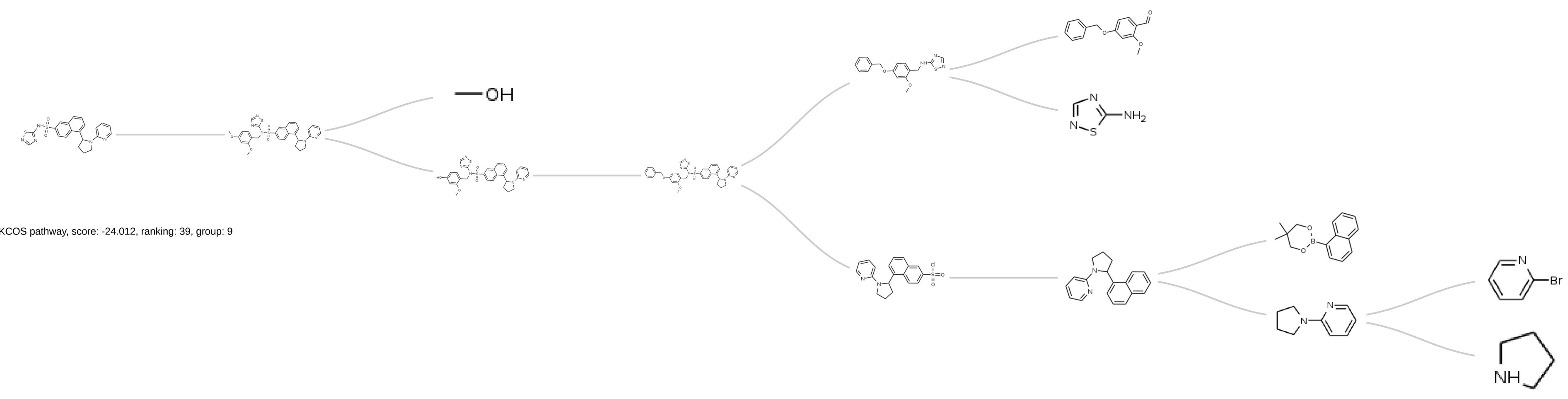

ASKCOS pathway, score: -24.012, ranking: 39, group: 9

Model ranks patent pathway as top-1: Example 4

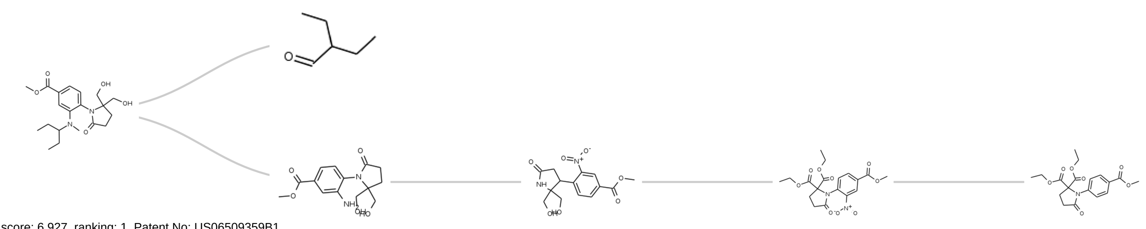

Piatachio pathway, score: 6.927, ranking: 1, Patent No: US06509359B1

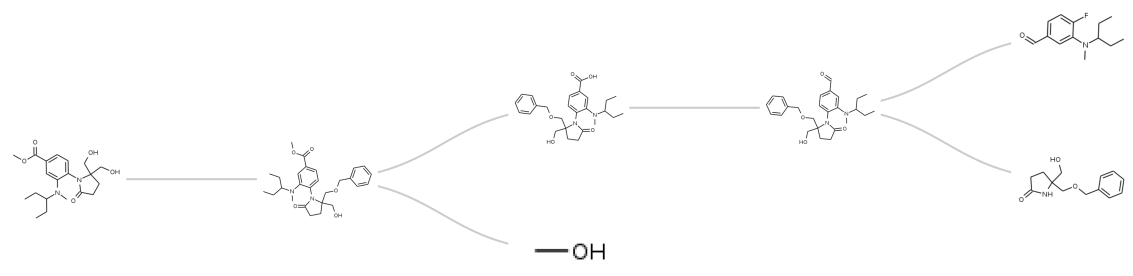

ASKCOS pathway, score: -17.138, ranking: 2, group: 1

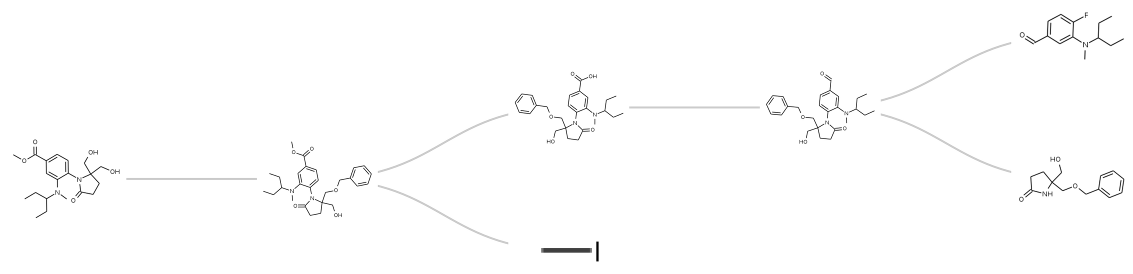

ASKCOS pathway, score: -18.026, ranking: 3, group: 2

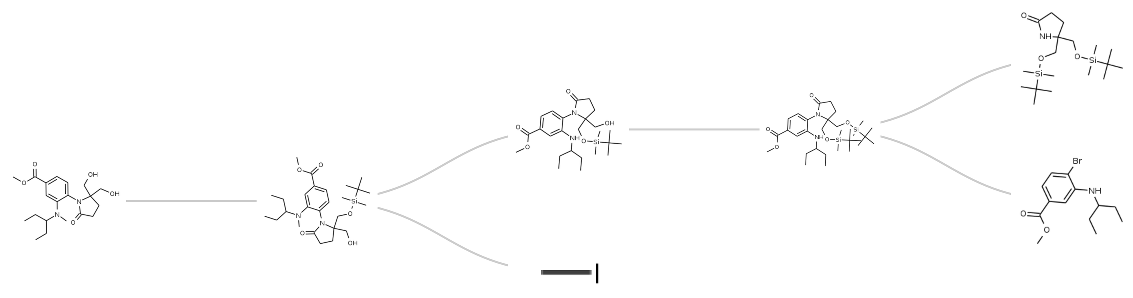

ASKCOS pathway, score: -18.795, ranking: 4, group: 3

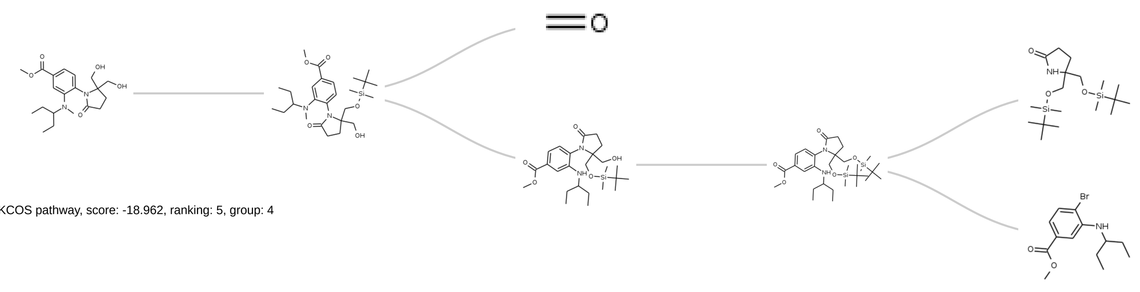

ASKCOS pathway, score: -18.962, ranking: 5, group: 4

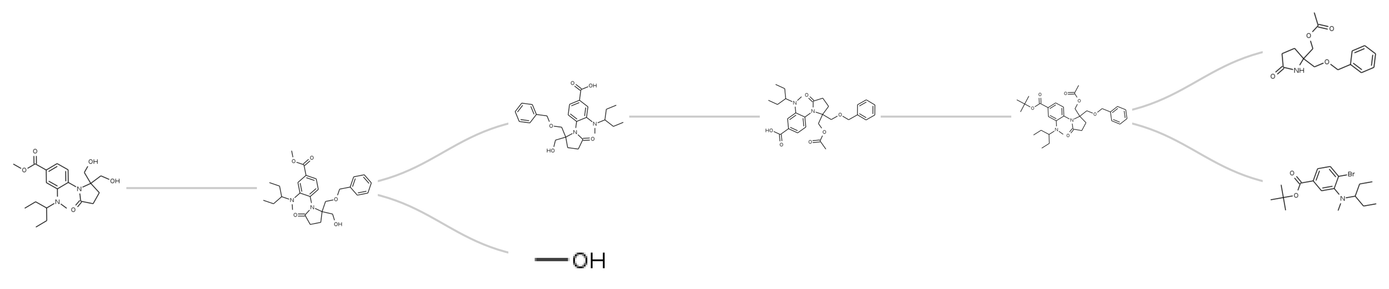

ASKCOS pathway, score: -19.487, ranking: 6, group: 5

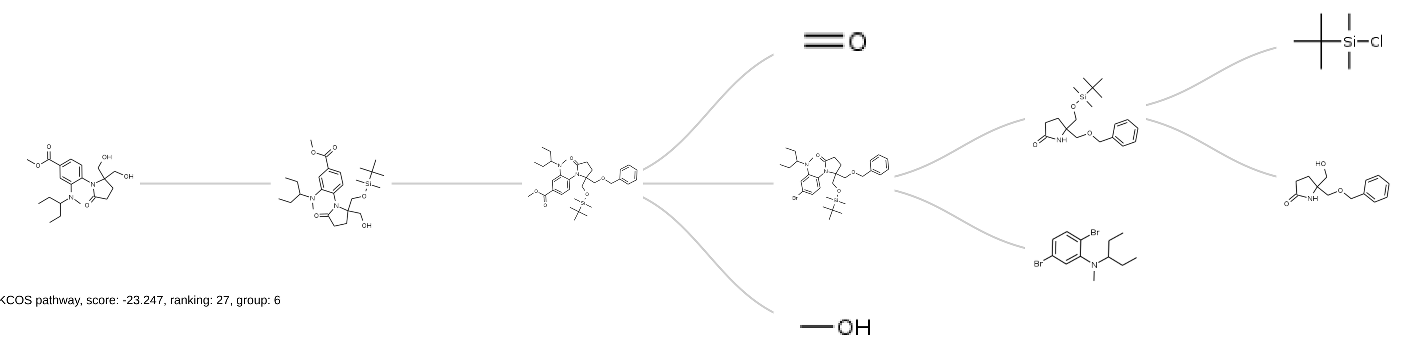

ASKCOS pathway, score: -23.247, ranking: 27, group: 6

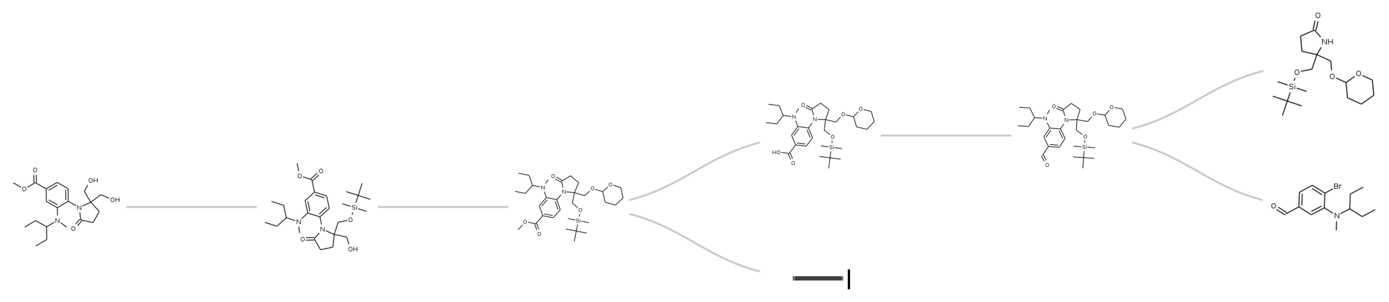

ASKCOS pathway, score: -24.957, ranking: 44, group: 7

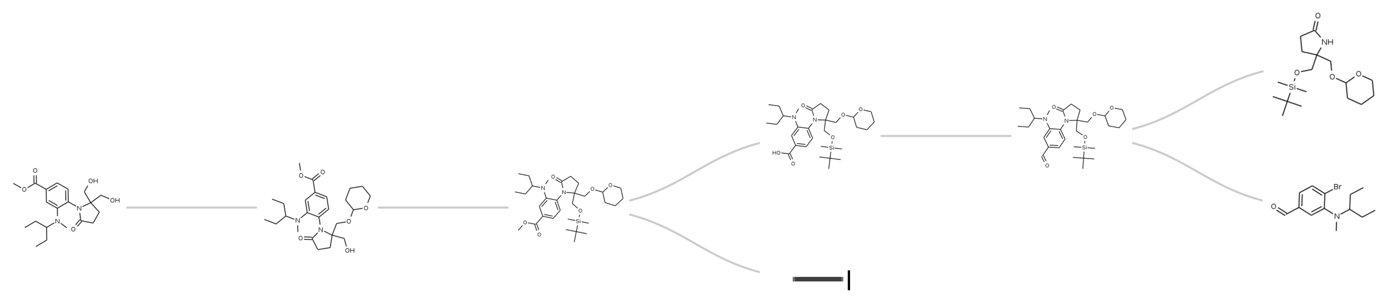

ASKCOS pathway, score: -25.993, ranking: 55, group: 8

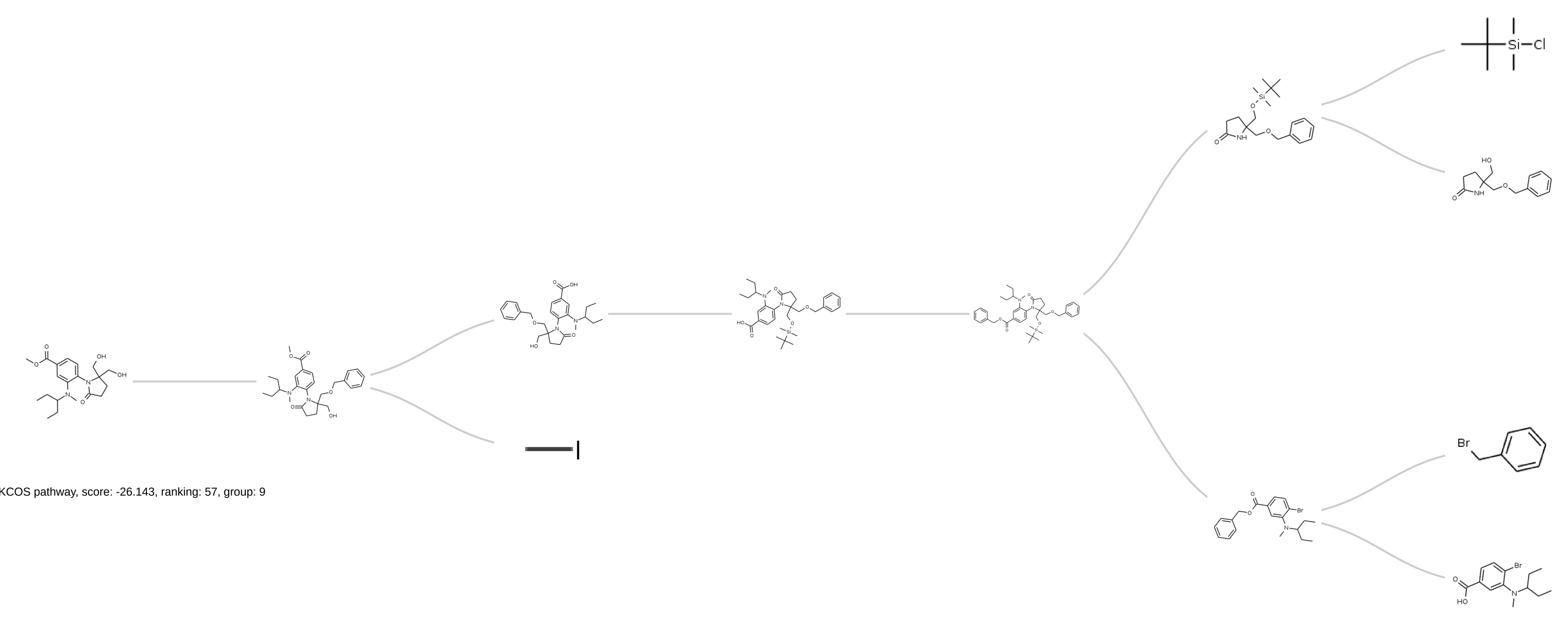

ASKCOS pathway, score: -26.143, ranking: 57, group: 9

Model ranks patent pathway as top-1: Example 5

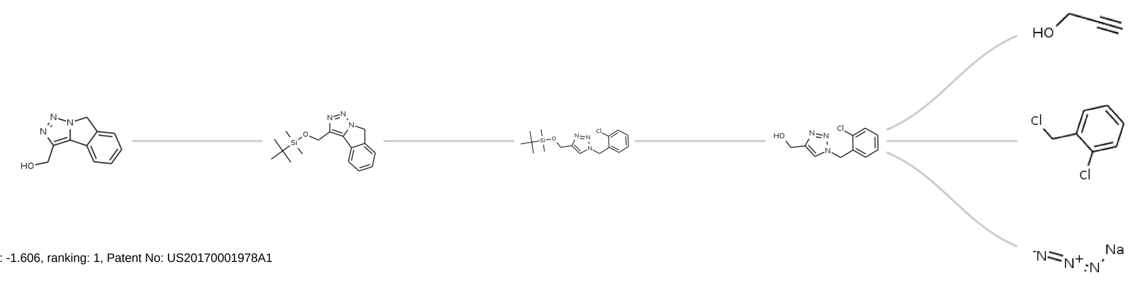

Piatachio pathway, score: -1.606, ranking: 1, Patent No: US20170001978A1

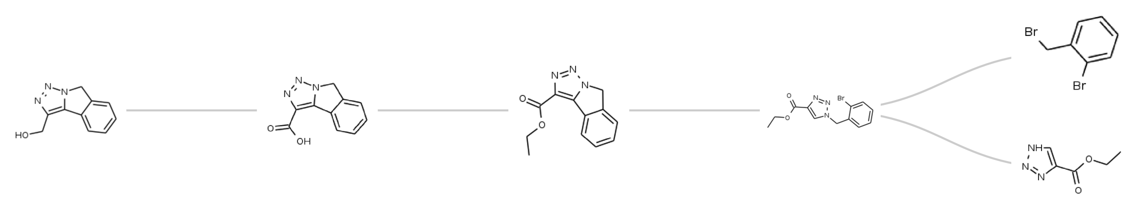

ASKCOS pathway, score: -12.987, ranking: 2, group: 1

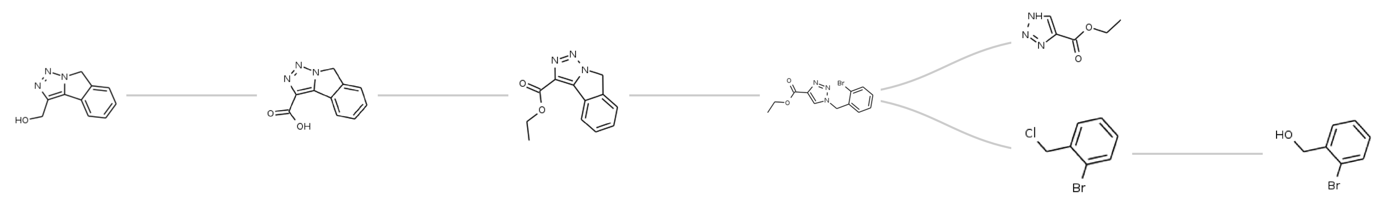

ASKCOS pathway, score: -13.977, ranking: 4, group: 2

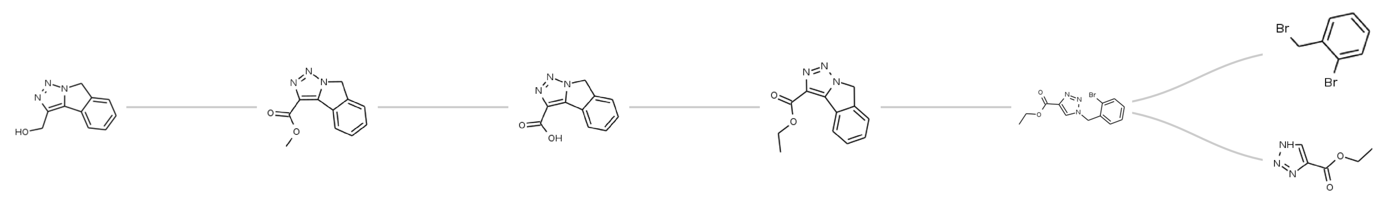

ASKCOS pathway, score: -15.317, ranking: 8, group: 3

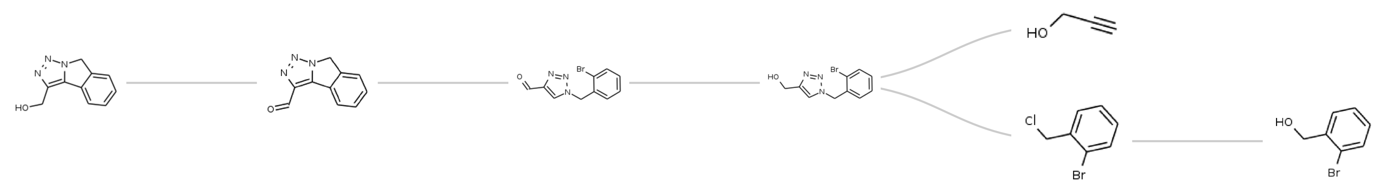

ASKCOS pathway, score: -16.381, ranking: 19, group: 4

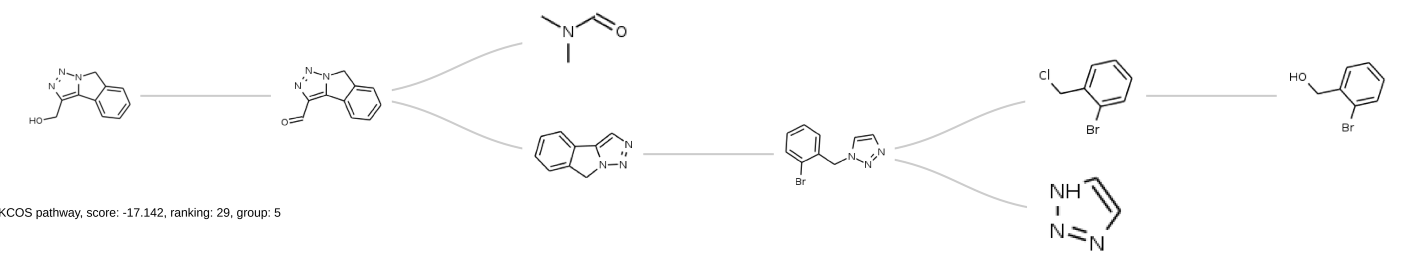

ASKCOS pathway, score: -17.142, ranking: 29, group: 5

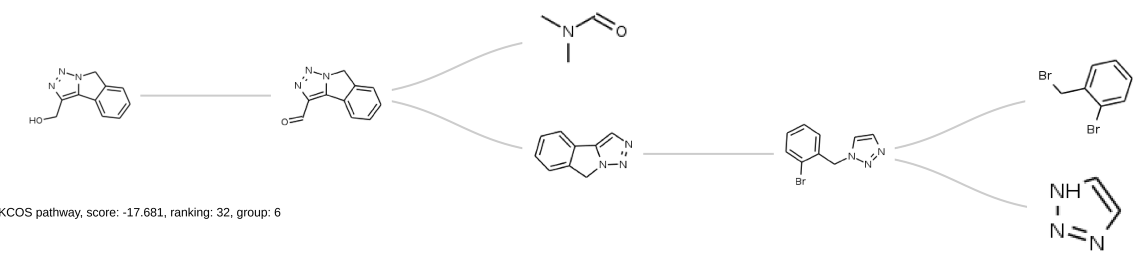

ASKCOS pathway, score: -17.681, ranking: 32, group: 6

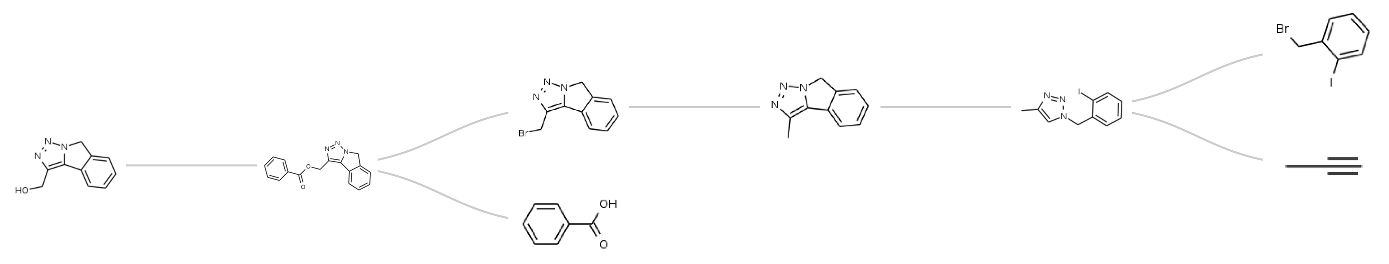

ASKCOS pathway, score: -19.981, ranking: 47, group: 7

Model ranks patent pathway as top-1: Example 6

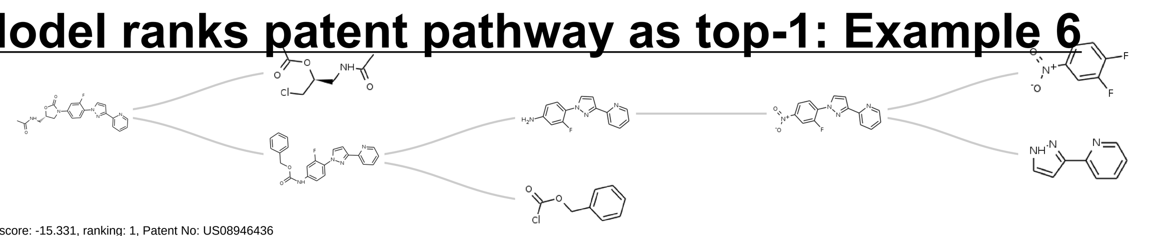

Piatachio pathway, score: -15.331, ranking: 1, Patent No: US08946436

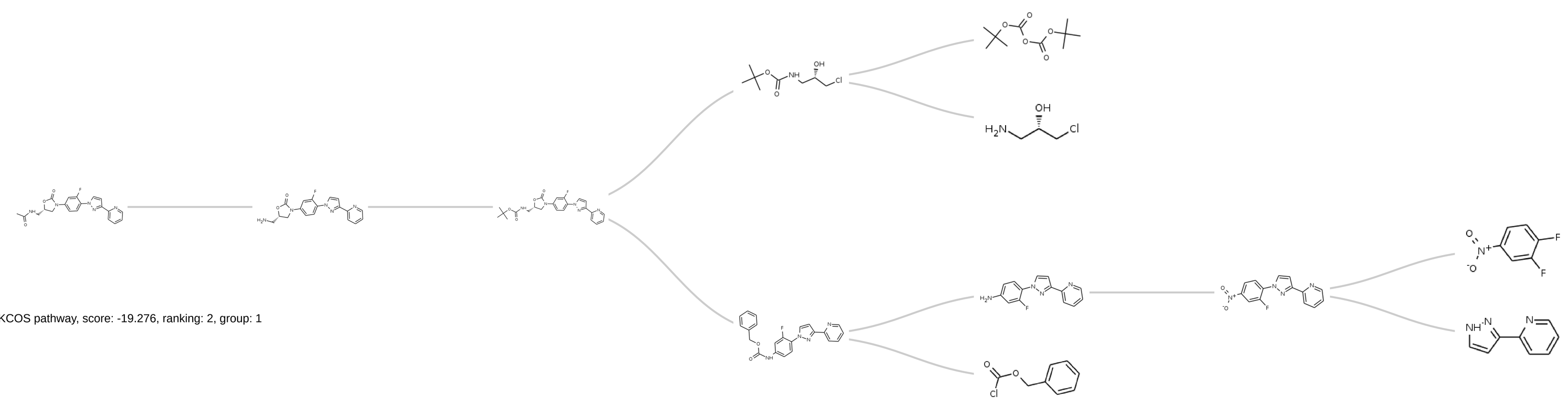

ASKCOS pathway, score: -19.276, ranking: 2, group: 1

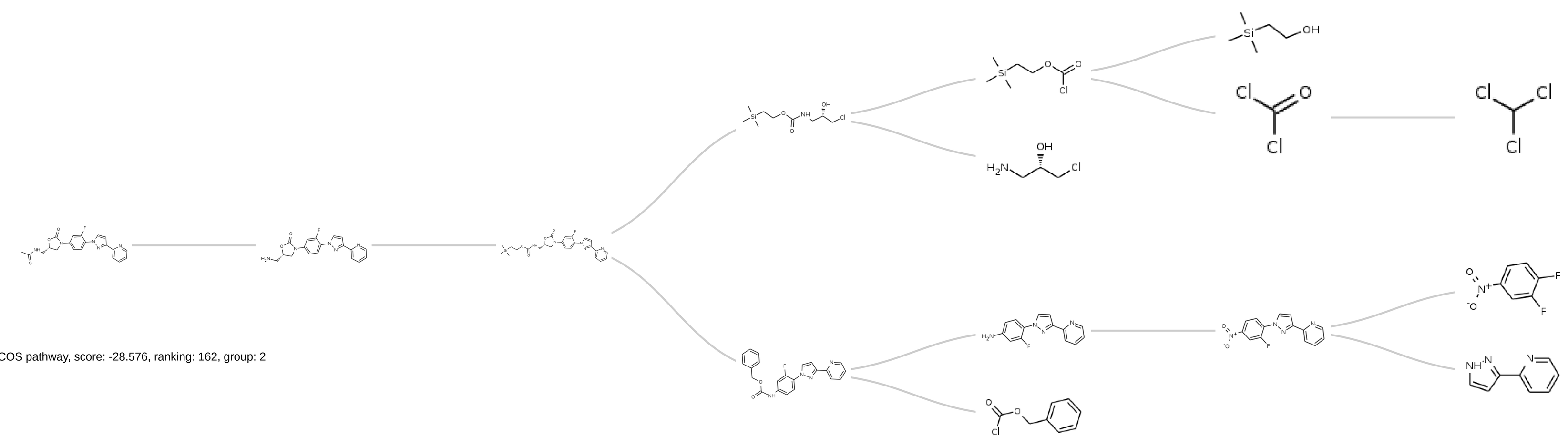

ASKCOS pathway, score: -28.576, ranking: 162, group: 2

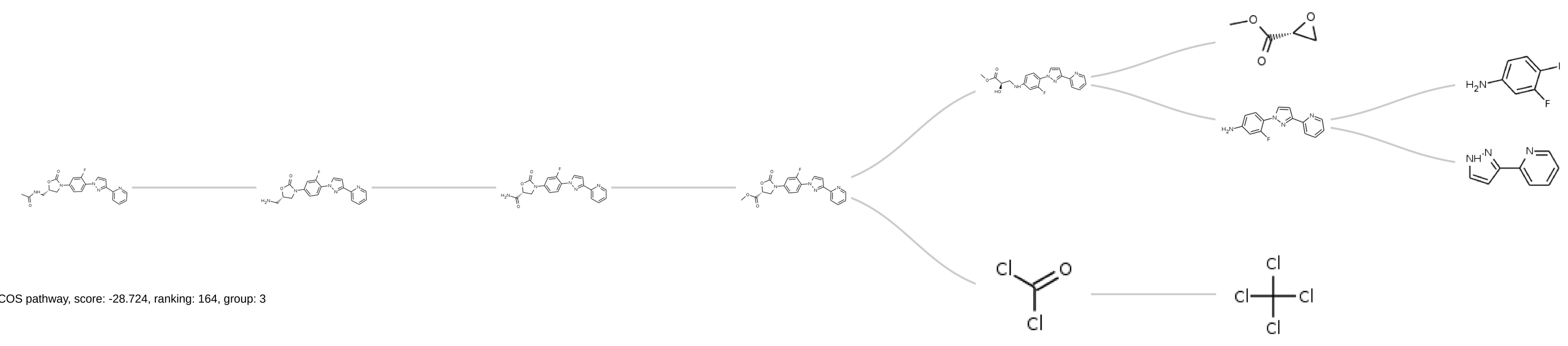

ASKCOS pathway, score: -28.724, ranking: 164, group: 3

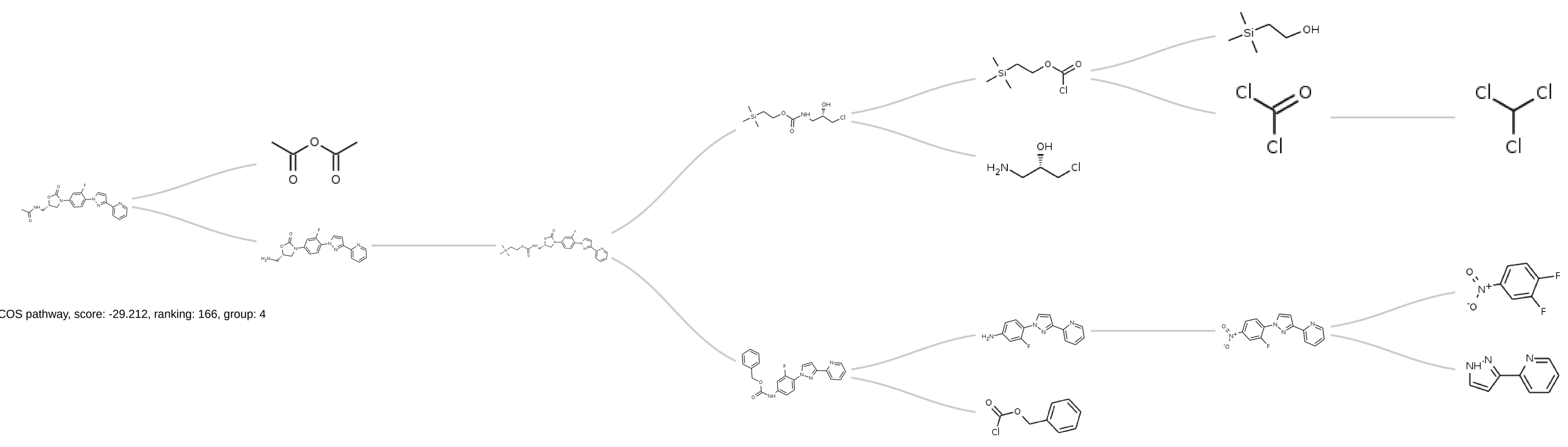

ASKCOS pathway, score: -29.212, ranking: 166, group: 4

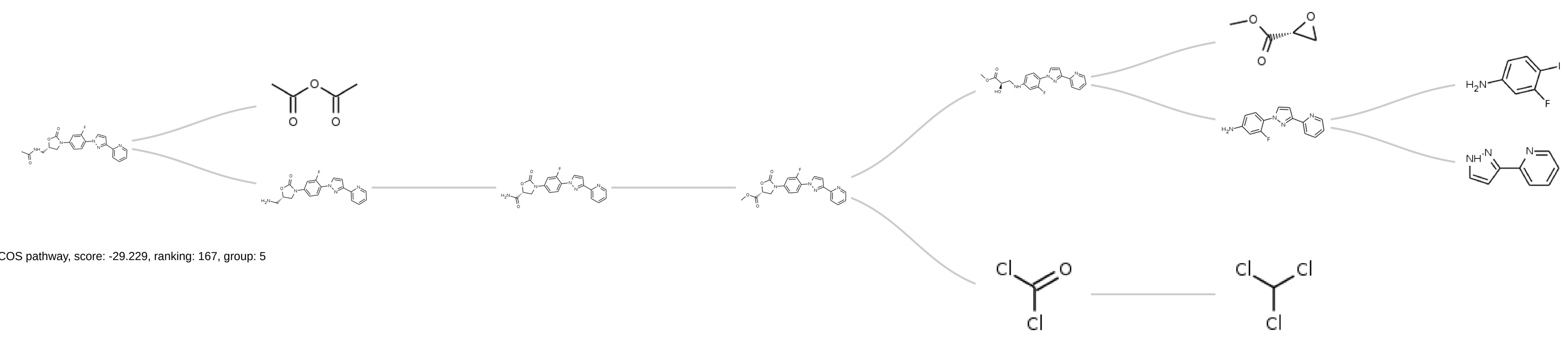

ASKCOS pathway, score: -29.229, ranking: 167, group: 5

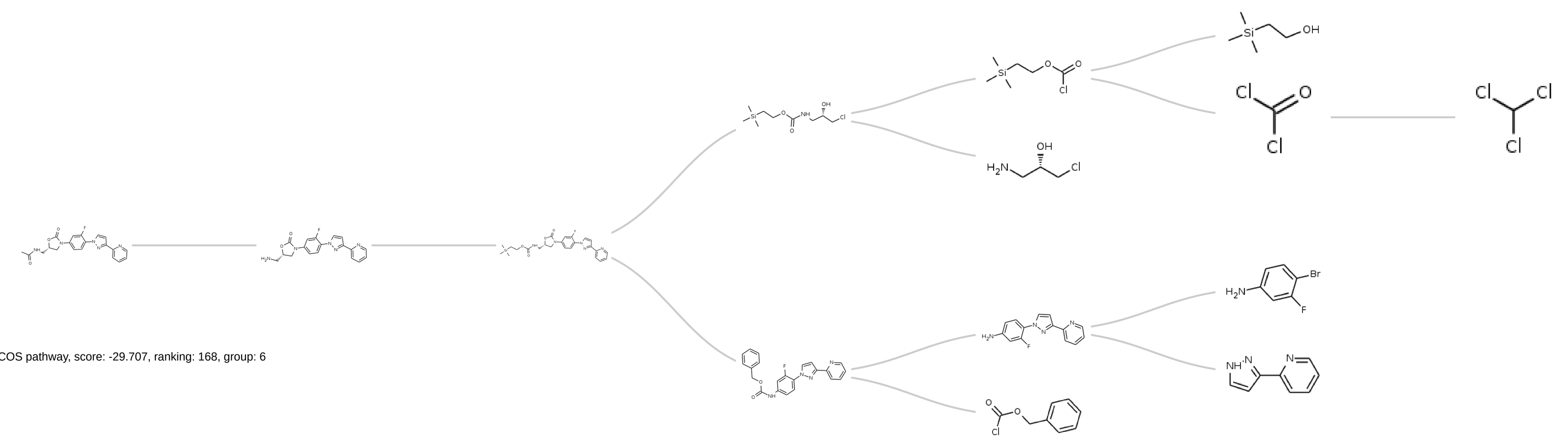

ASKCOS pathway, score: -29.707, ranking: 168, group: 6

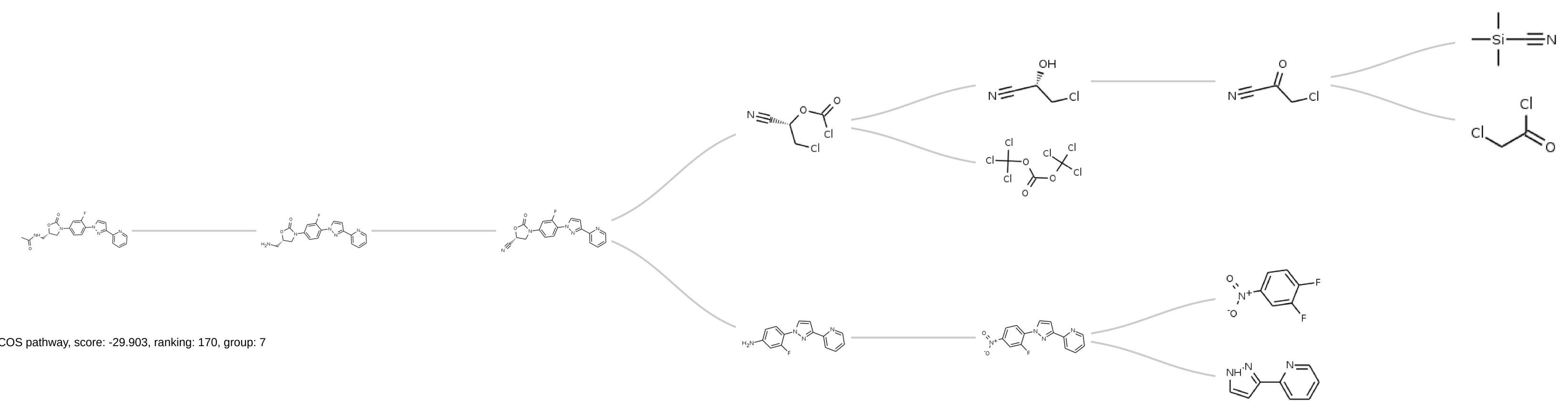

ASKCOS pathway, score: -29.903, ranking: 170, group: 7

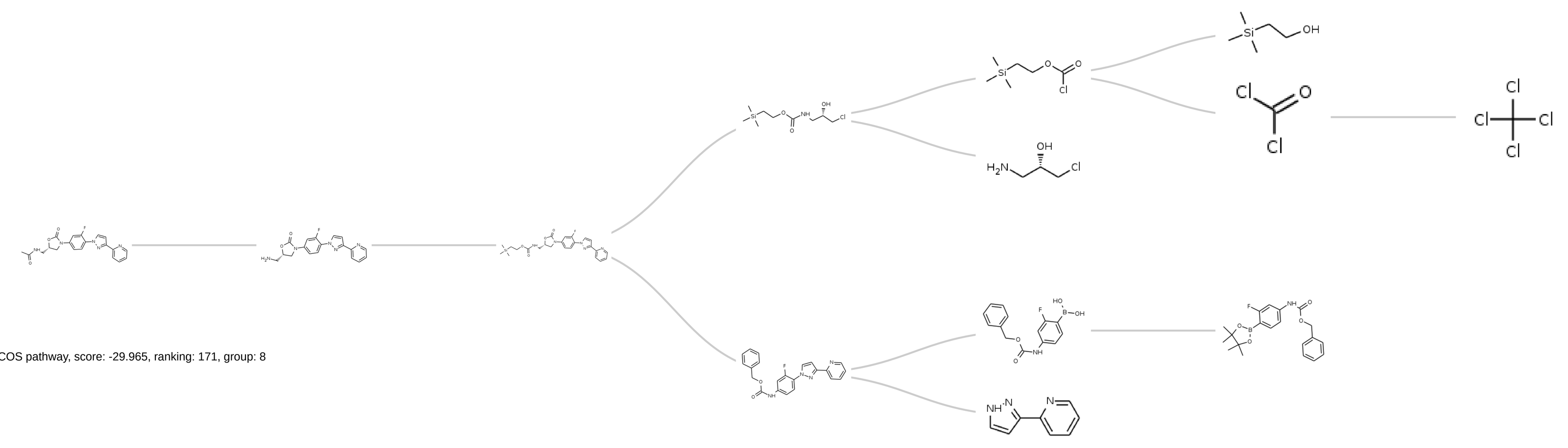

ASKCOS pathway, score: -29.965, ranking: 171, group: 8

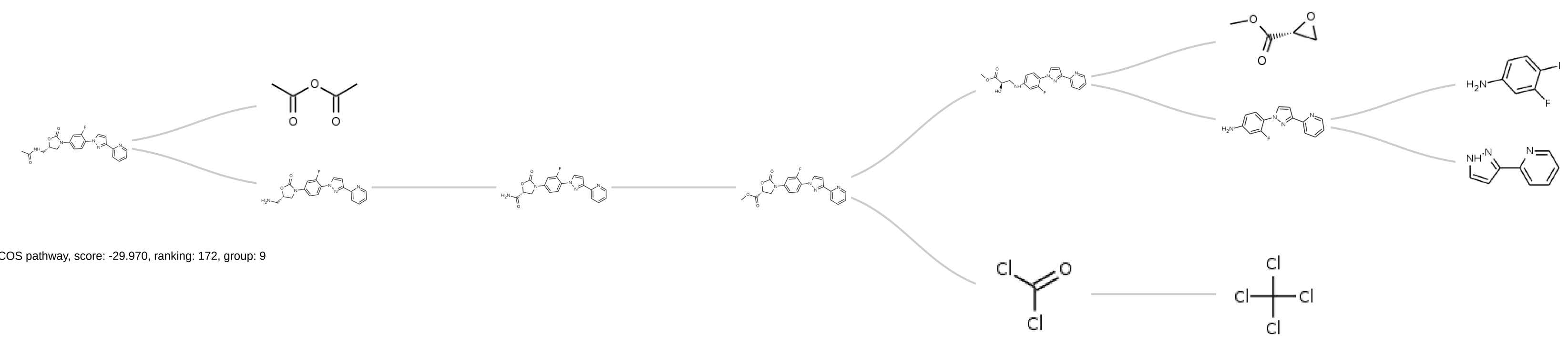

ASKCOS pathway, score: -29.970, ranking: 172, group: 9

Model ranks patent pathway as top-1: Example 7

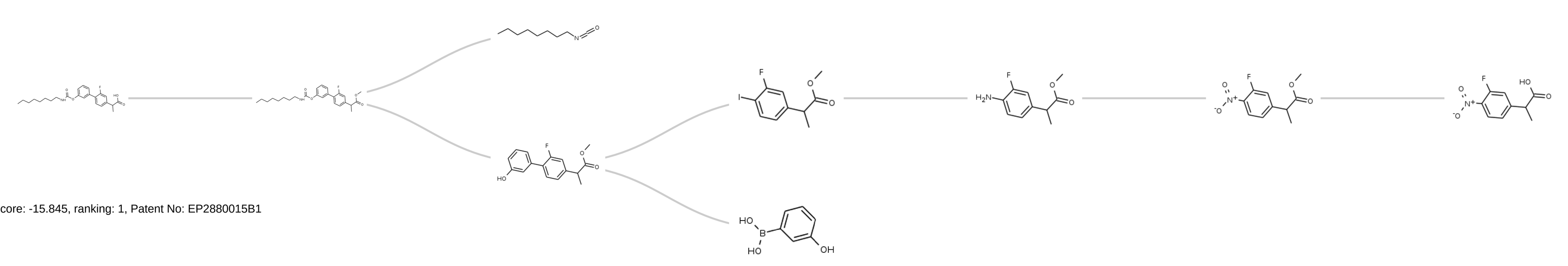

Piatachio pathway, score: -15.845, ranking: 1, Patent No: EP2880015B1

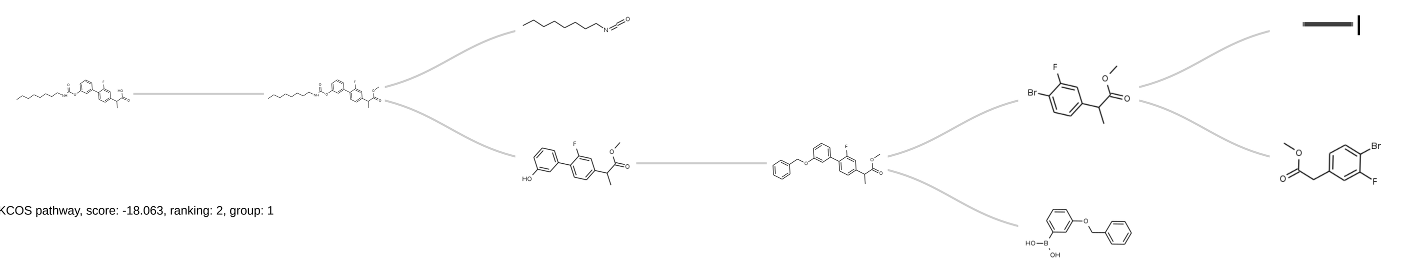

ASKCOS pathway, score: -18.063, ranking: 2, group: 1

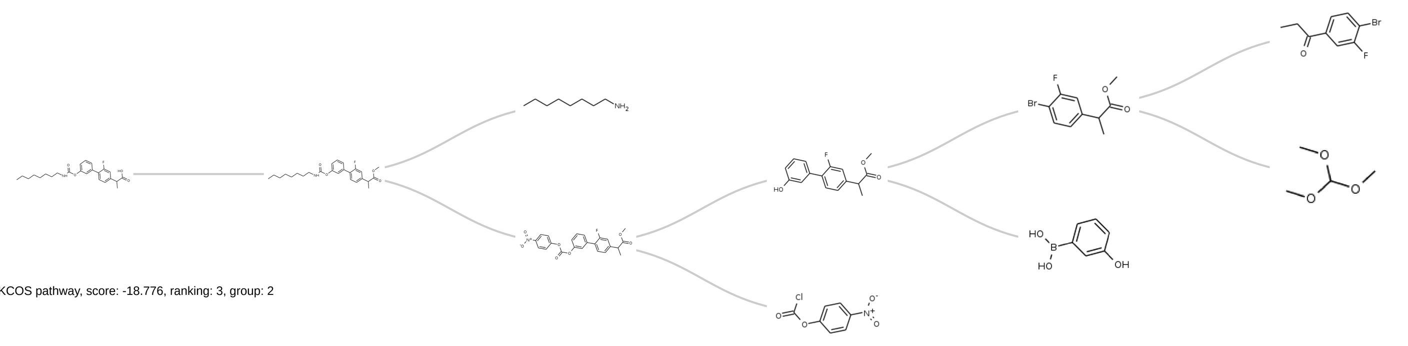

ASKCOS pathway, score: -18.776, ranking: 3, group: 2

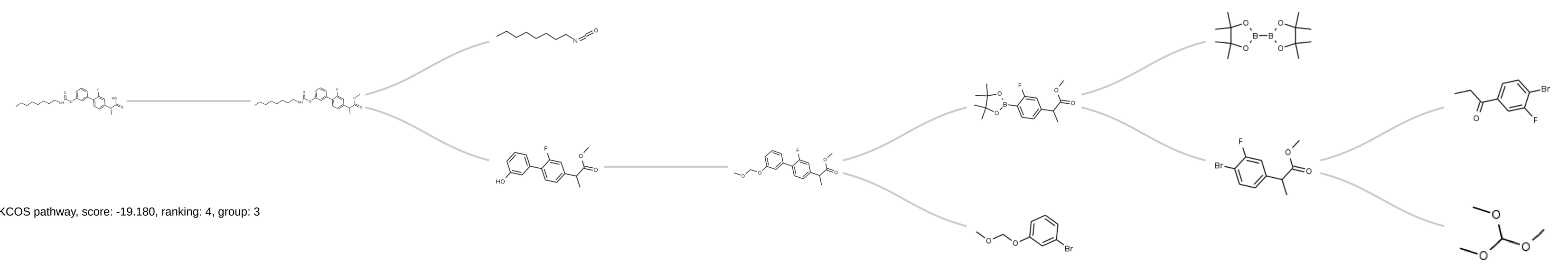

ASKCOS pathway, score: -19.180, ranking: 4, group: 3

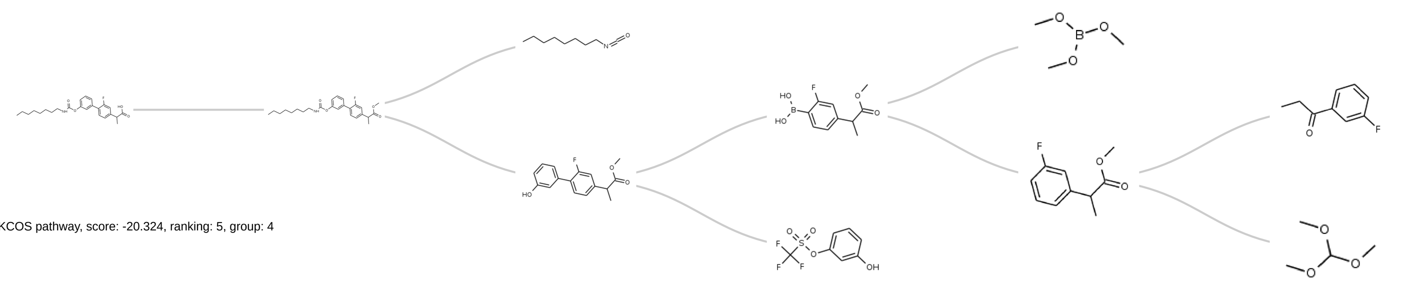

ASKCOS pathway, score: -20.324, ranking: 5, group: 4

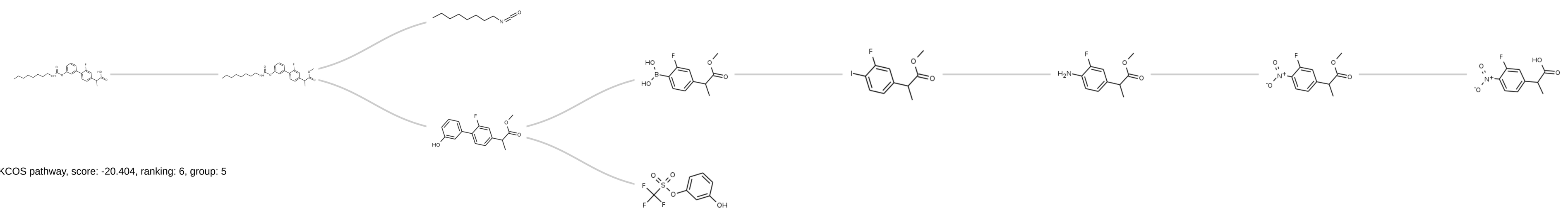

ASKCOS pathway, score: -20.404, ranking: 6, group: 5

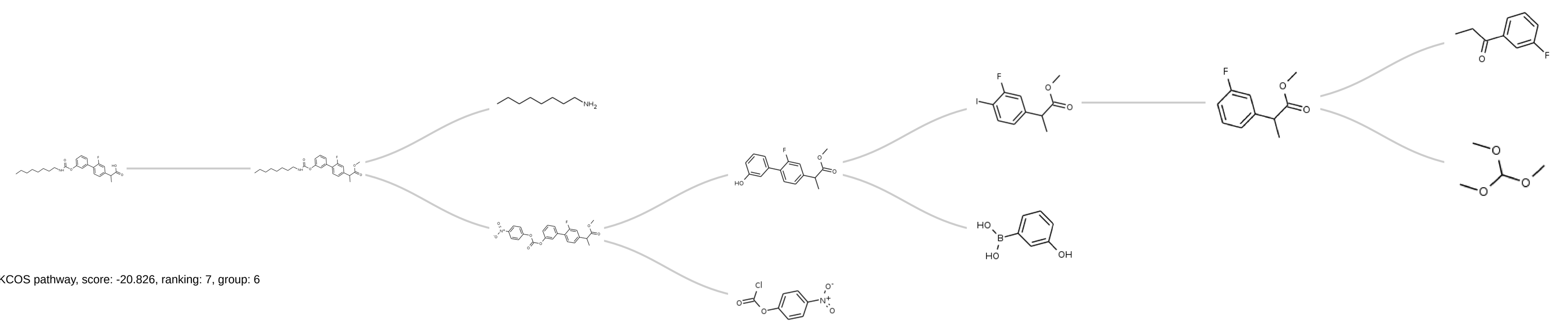

ASKCOS pathway, score: -20.826, ranking: 7, group: 6

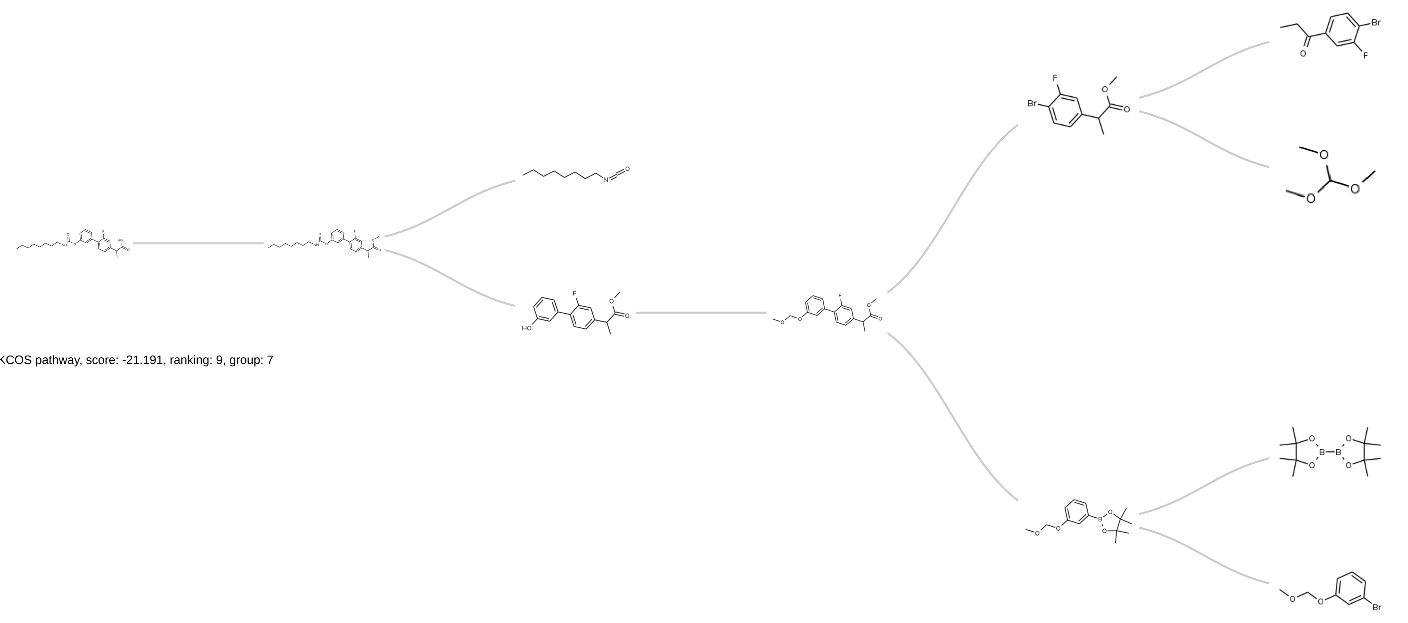

ASKCOS pathway, score: -21.191, ranking: 9, group: 7

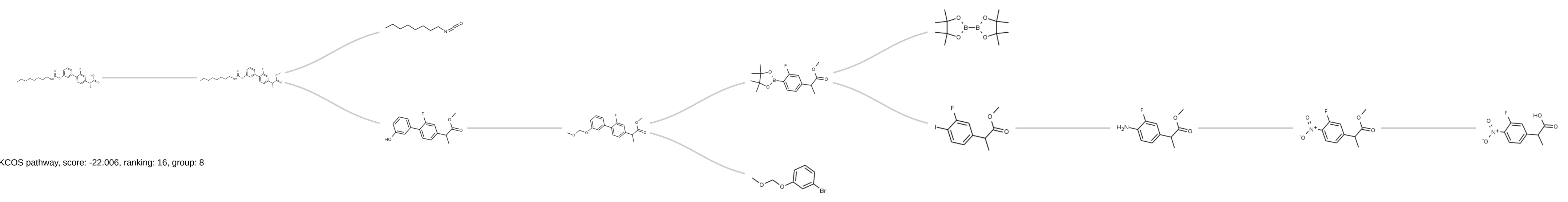

ASKCOS pathway, score: -22.006, ranking: 16, group: 8

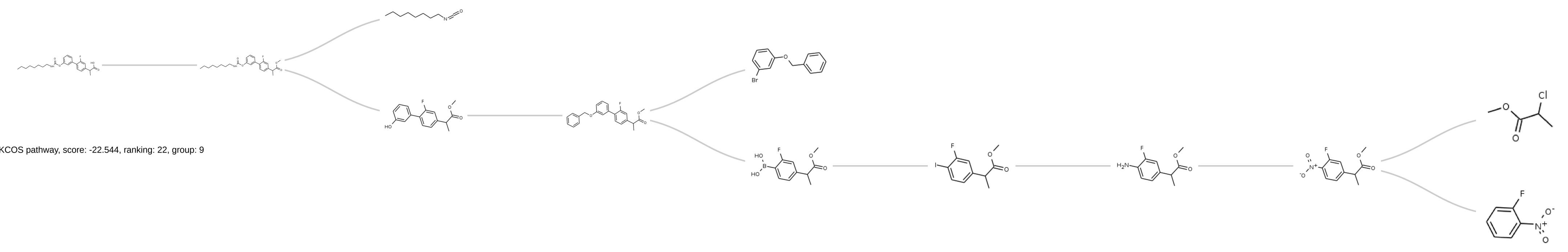

ASKCOS pathway, score: -22.544, ranking: 22, group: 9

Model ranks patent pathway as top-1: Example 8

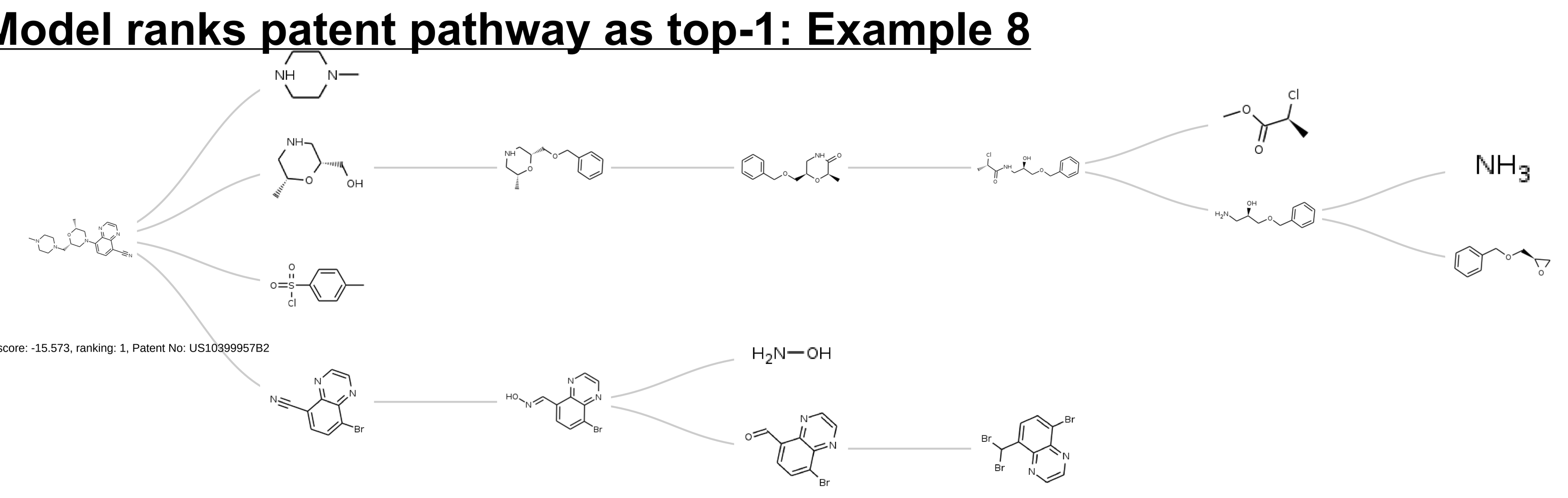

Piatachio pathway, score: -15.573, ranking: 1, Patent No: US10399957B2

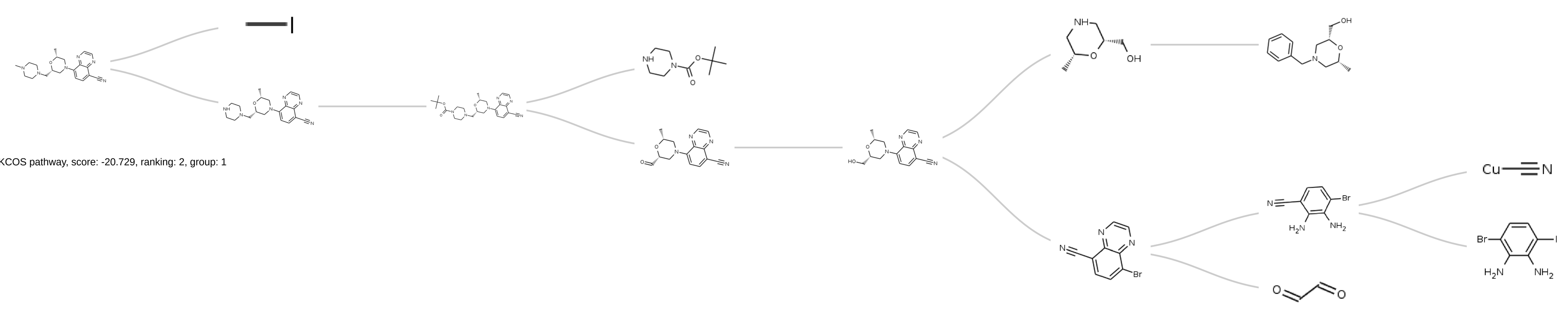

ASKCOS pathway, score: -20.729, ranking: 2, group: 1

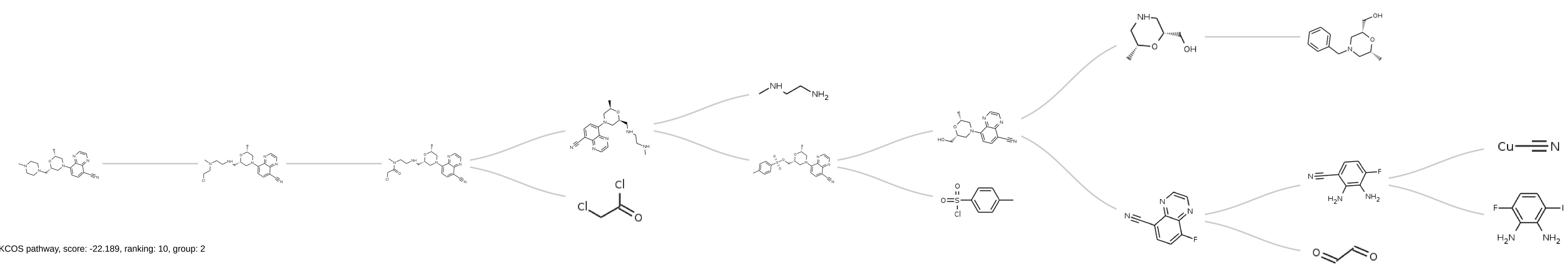

ASKCOS pathway, score: -22.189, ranking: 10, group: 2

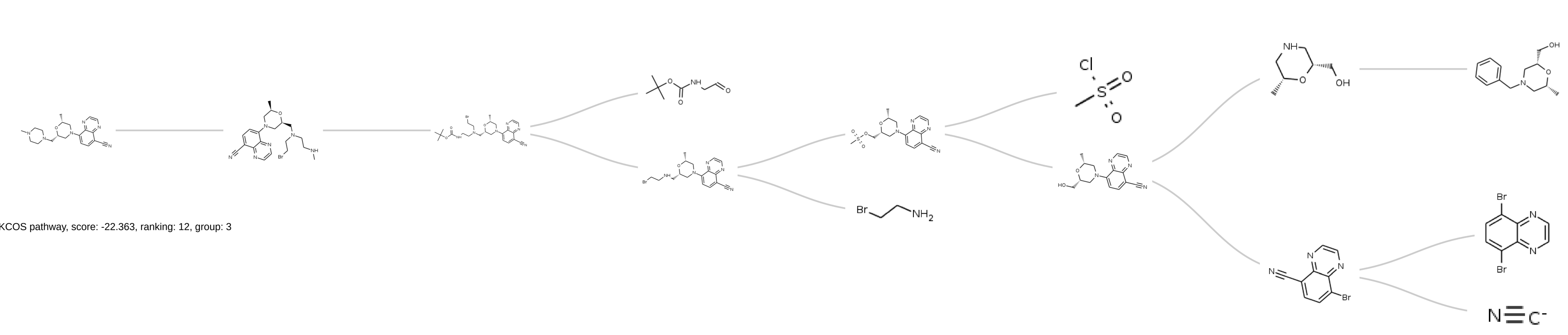

ASKCOS pathway, score: -22.363, ranking: 12, group: 3

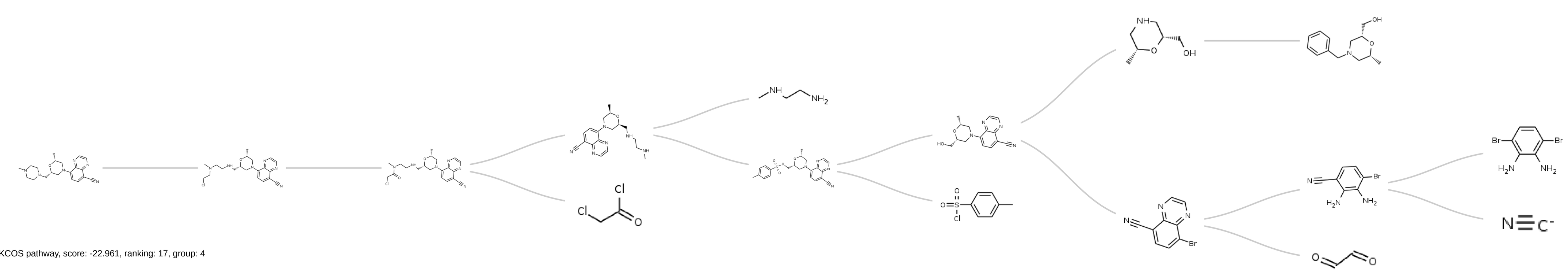

ASKCOS pathway, score: -22.961, ranking: 17, group: 4

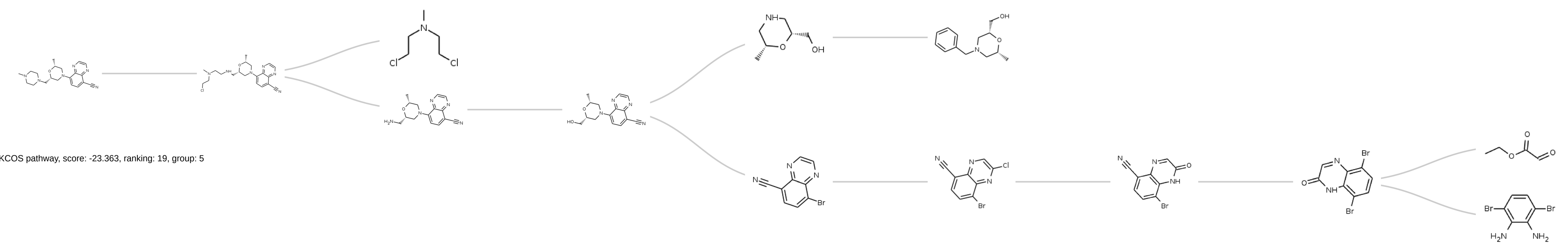

ASKCOS pathway, score: -23.363, ranking: 19, group: 5

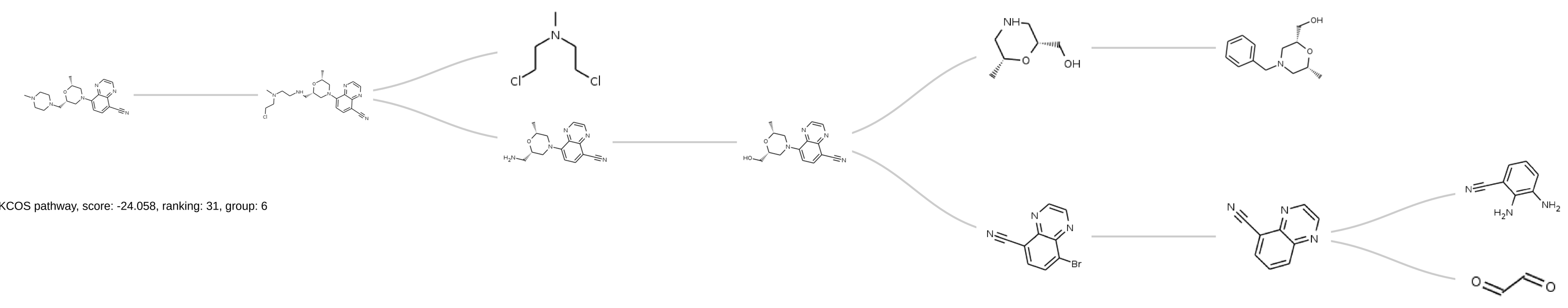

ASKCOS pathway, score: -24.058, ranking: 31, group: 6

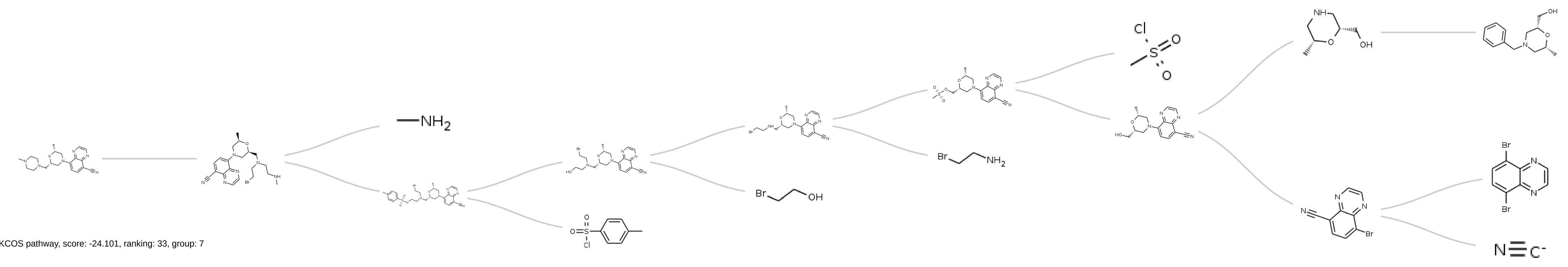

ASKCOS pathway, score: -24.101, ranking: 33, group: 7

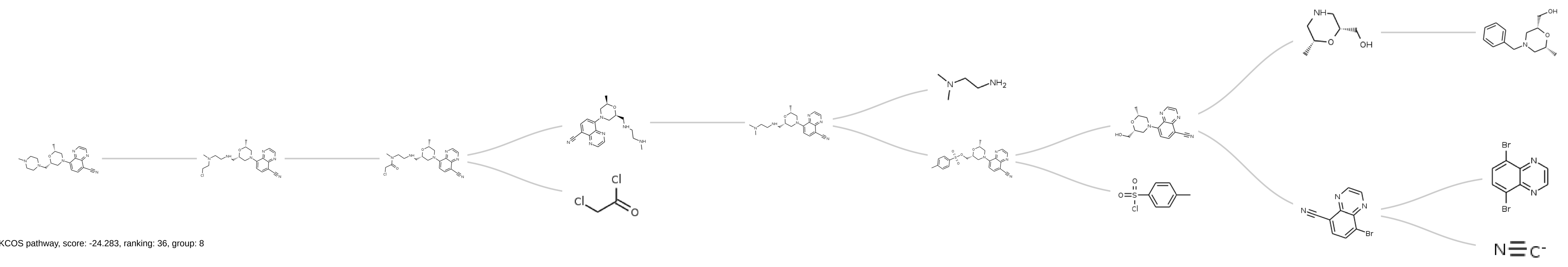

ASKCOS pathway, score: -24.283, ranking: 36, group: 8

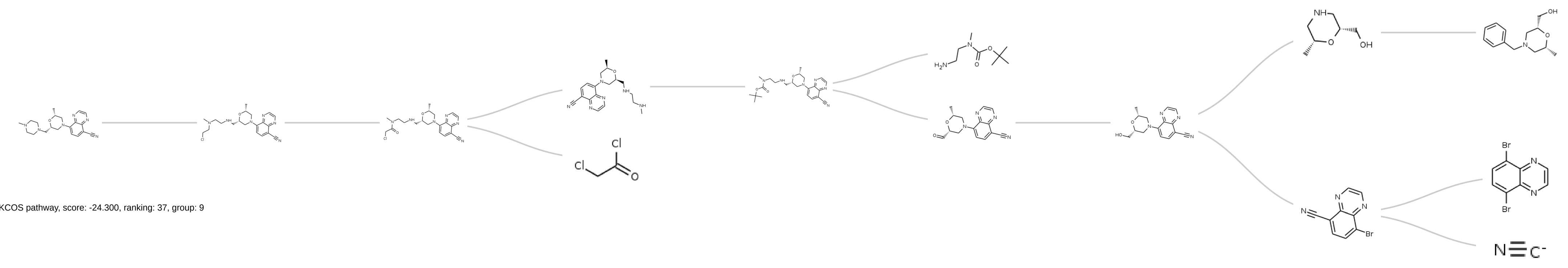

ASKCOS pathway, score: -24.300, ranking: 37, group: 9

Model ranks patent pathway as top-1: Example 9

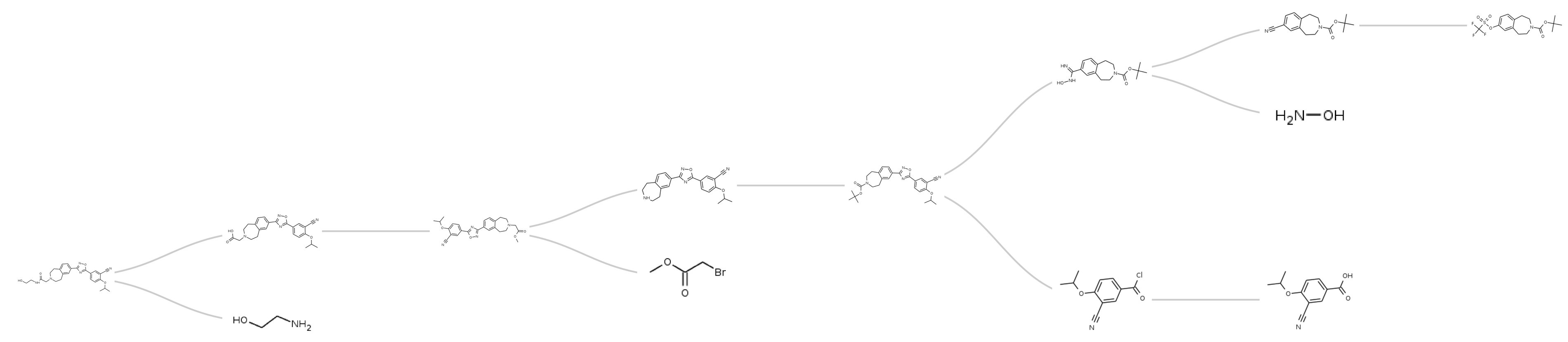

Piatachio pathway, score: -6.187, ranking: 1, Patent No: US20120101083A1

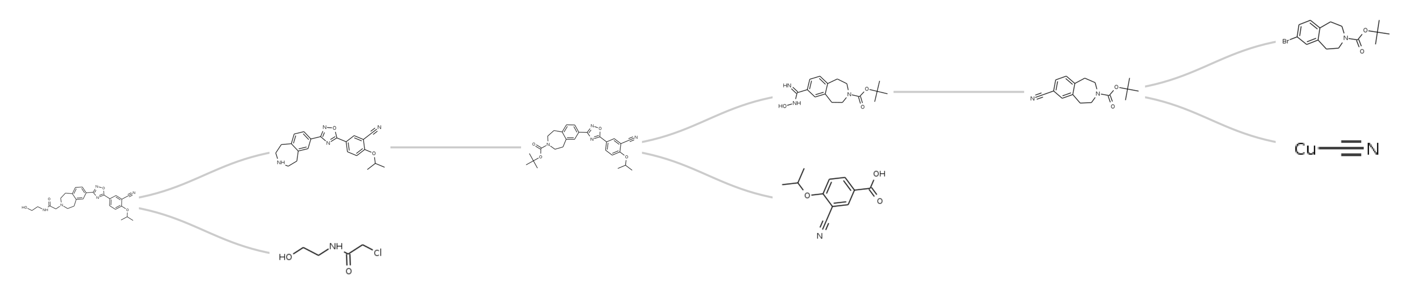

ASKCOS pathway, score: -11.898, ranking: 2, group: 1

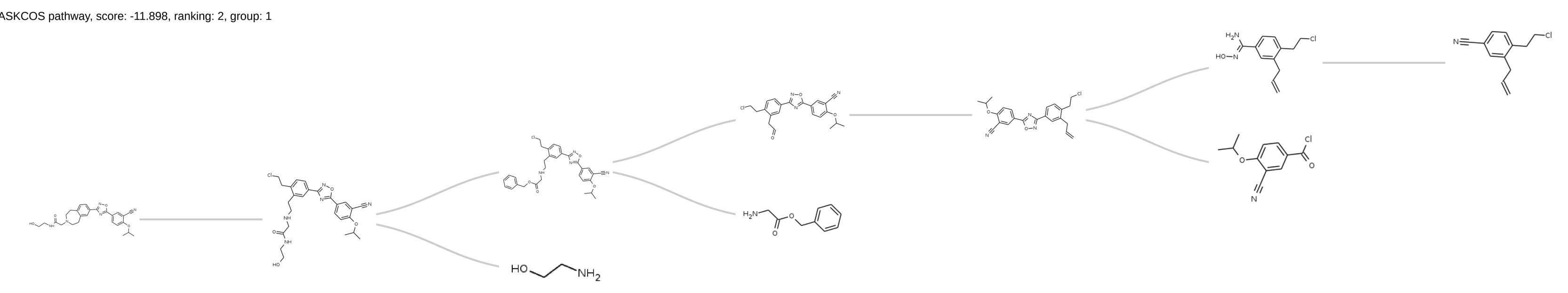

ASKCOS pathway, score: -25.233, ranking: 45, group: 2

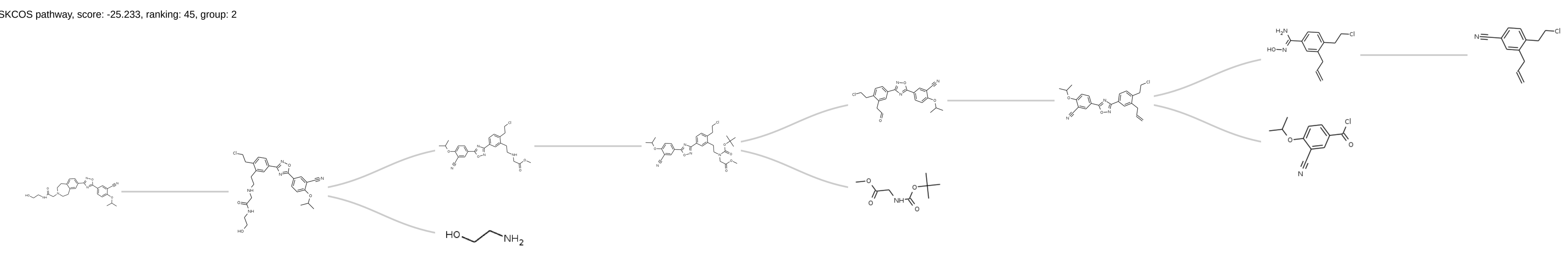

ASKCOS pathway, score: -26.371, ranking: 46, group: 3

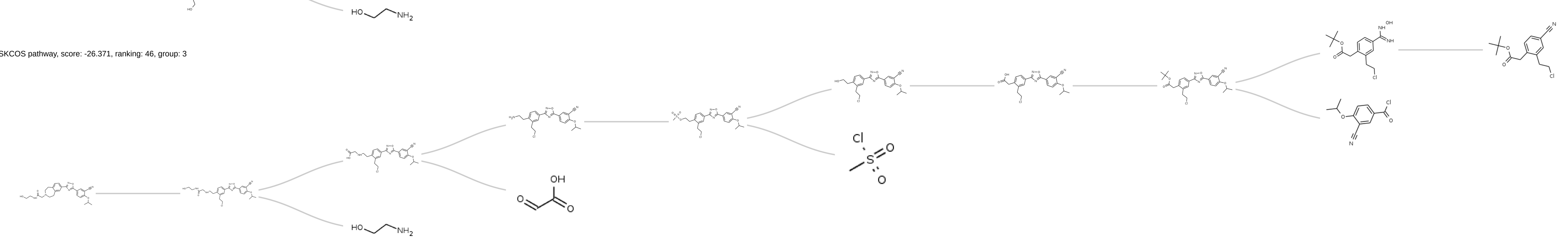

ASKCOS pathway, score: -31.696, ranking: 105, group: 4

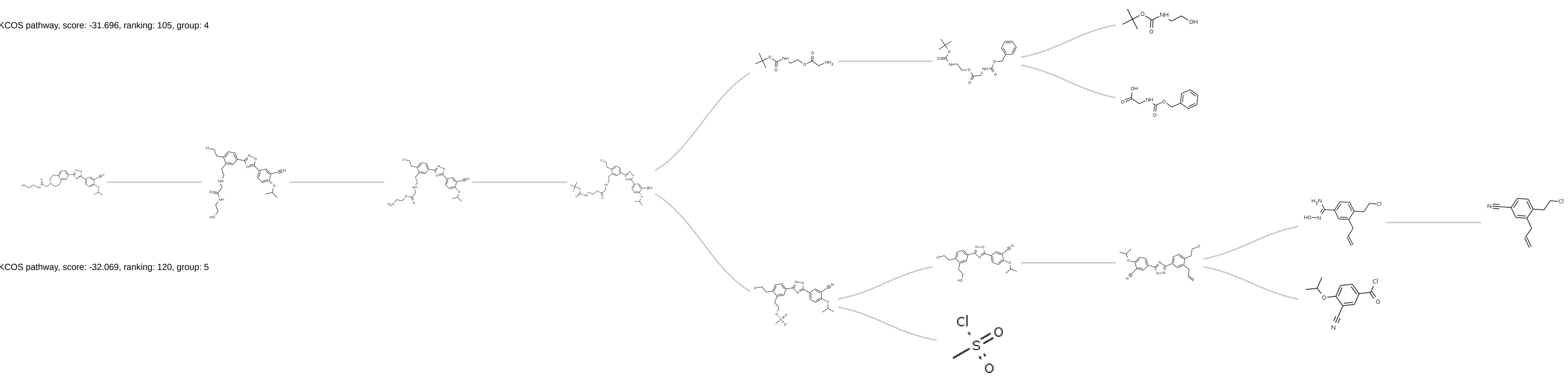

ASKCOS pathway, score: -32.069, ranking: 120, group: 5

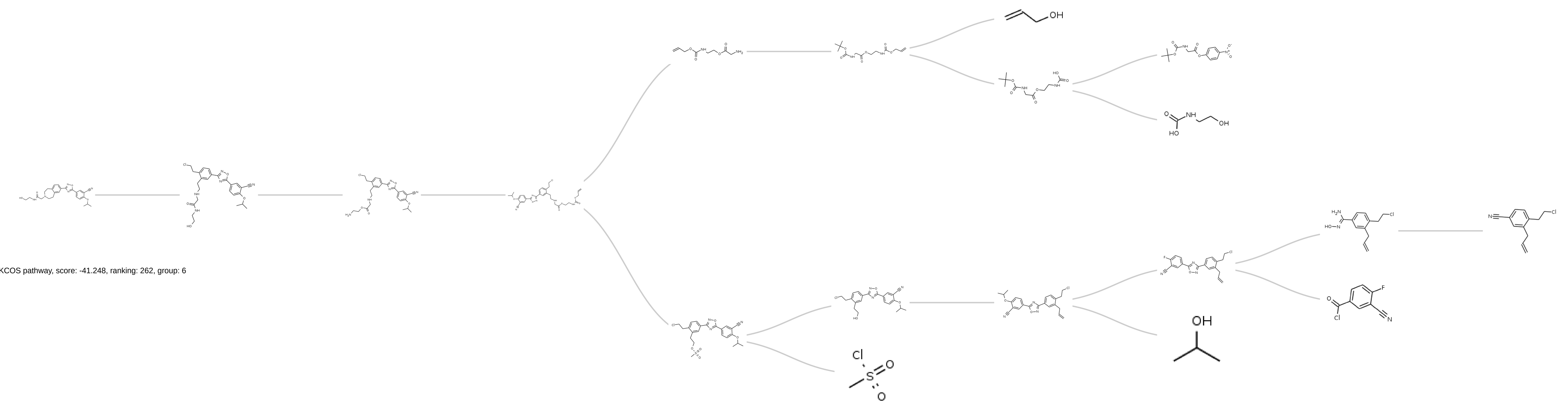

ASKCOS pathway, score: -41.248, ranking: 262, group: 6

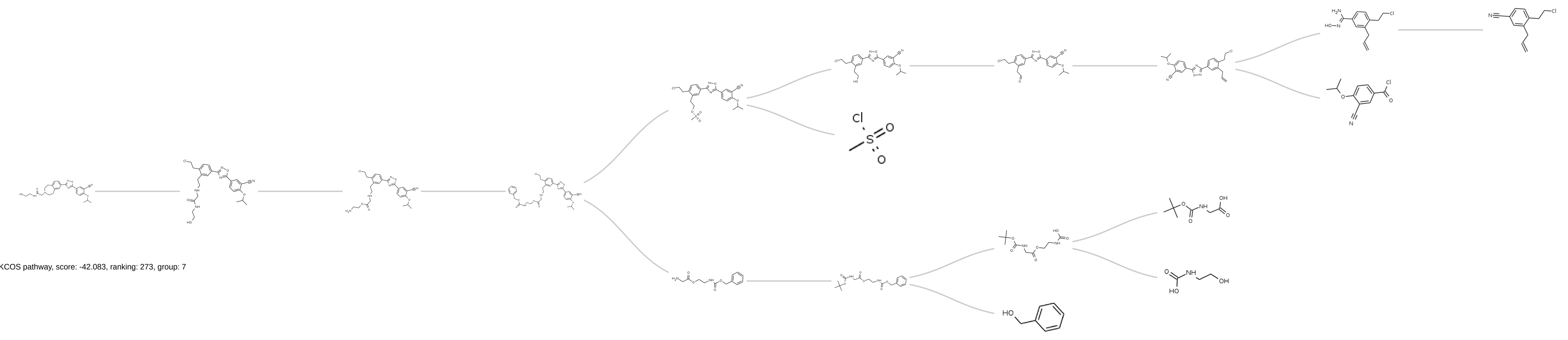

ASKCOS pathway, score: -42.083, ranking: 273, group: 7

Model ranks patent pathway as top-1: Example 10

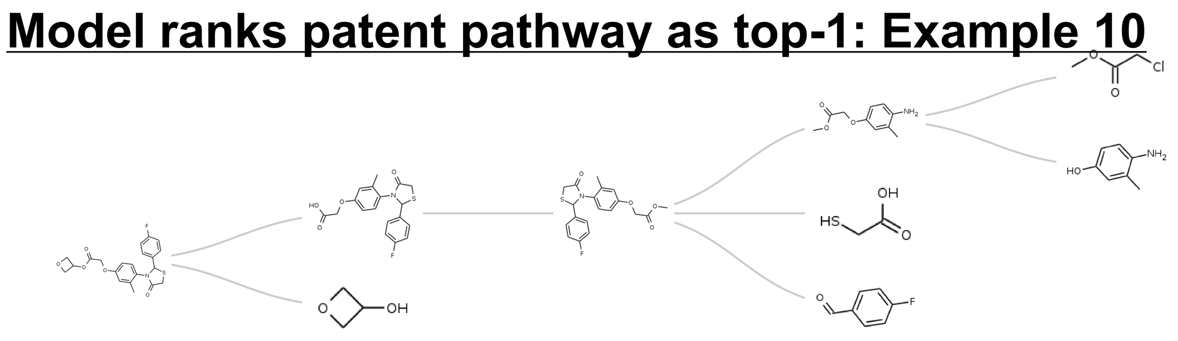

Piatachio pathway, score: -14.503, ranking: 1, Patent No: US20170253569A1

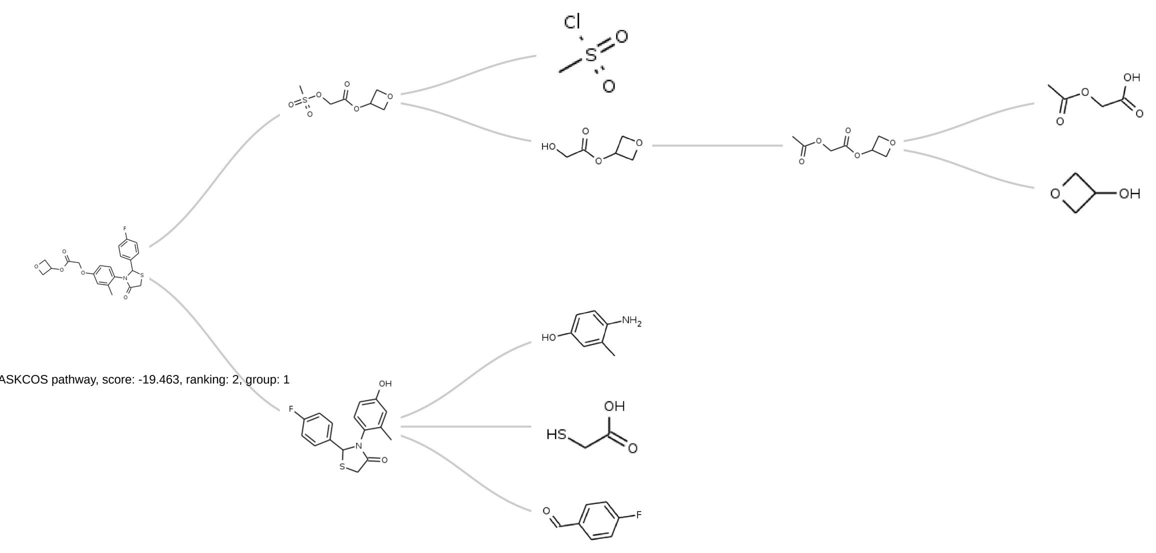

ASKCOS pathway, score: -19.463, ranking: 2, group: 1

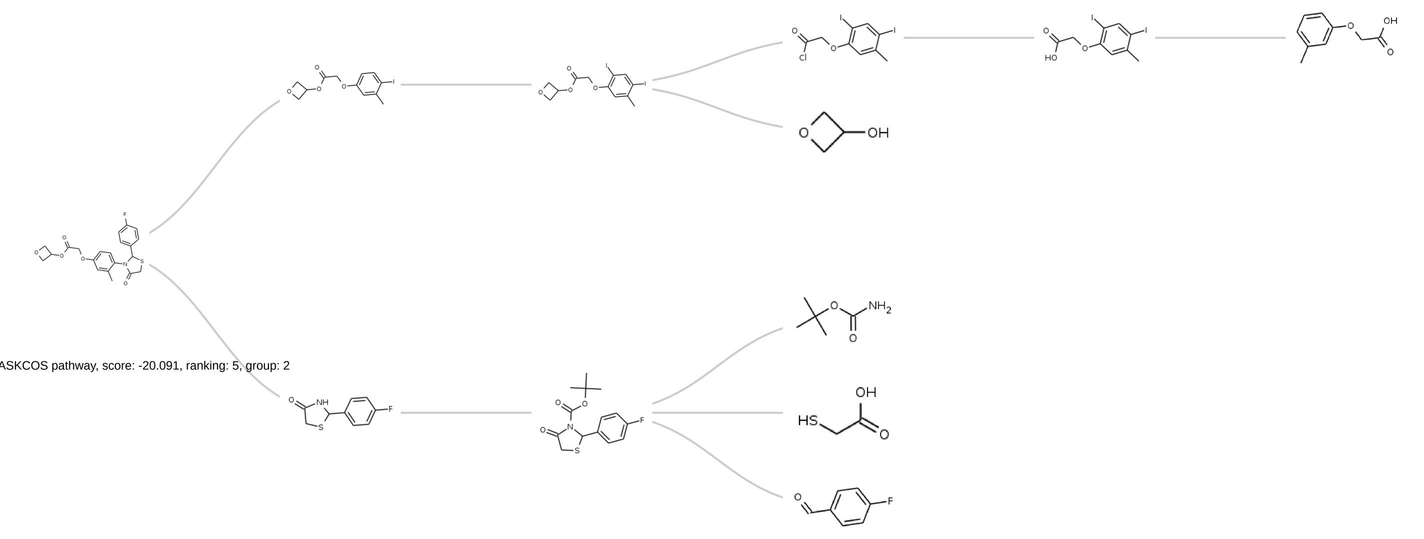

ASKCOS pathway, score: -20.091, ranking: 5, group: 2

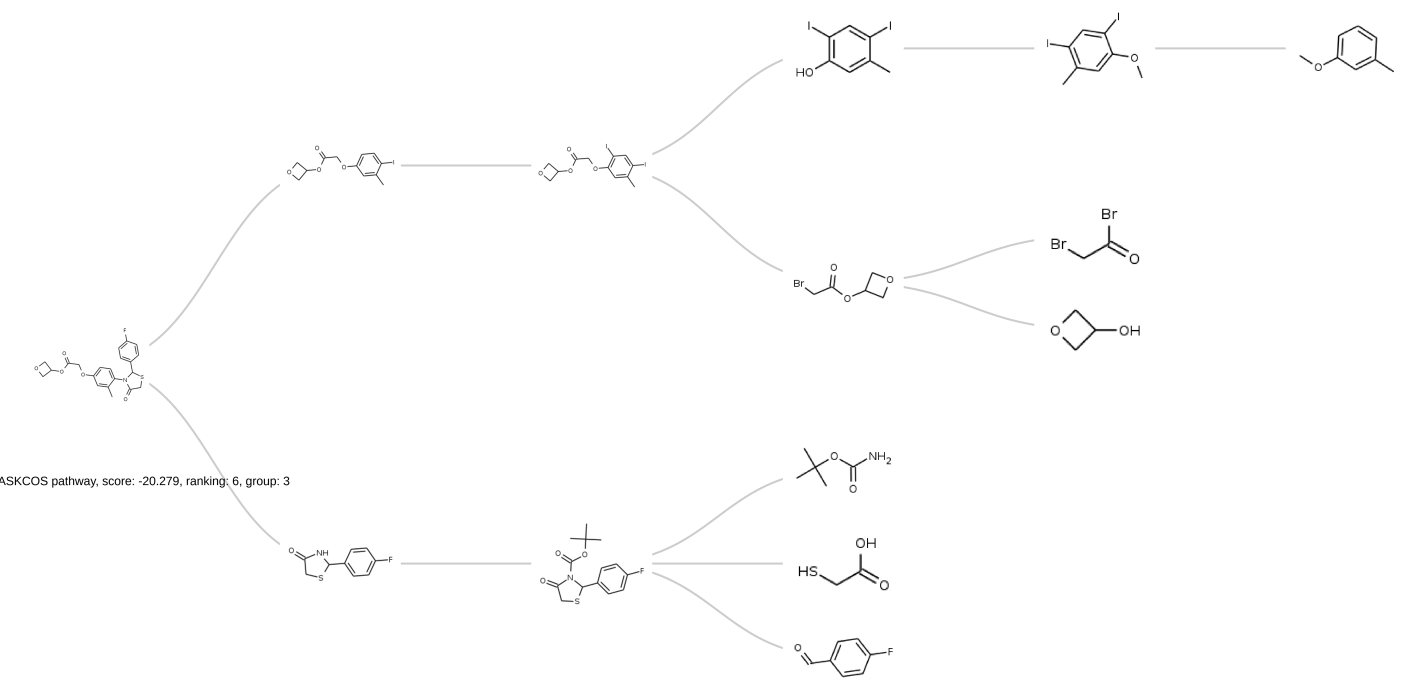

ASKCOS pathway, score: -20.279, ranking: 6, group: 3

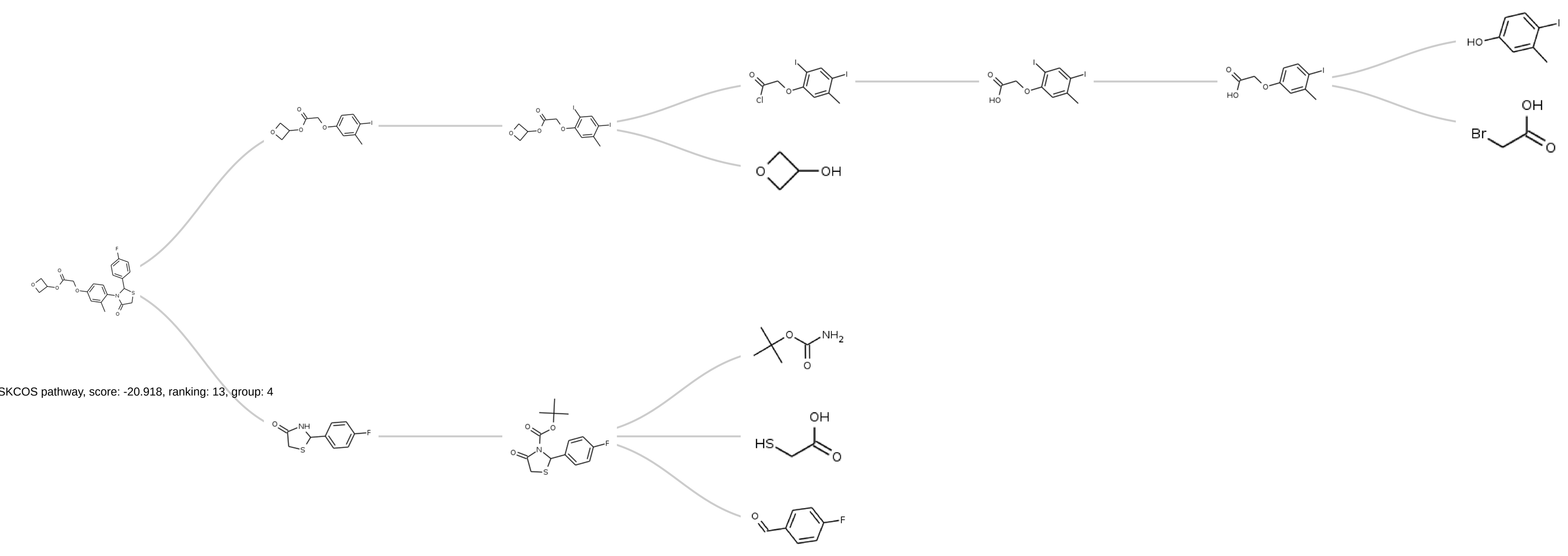

ASKCOS pathway, score: -20.918, ranking: 13, group: 4

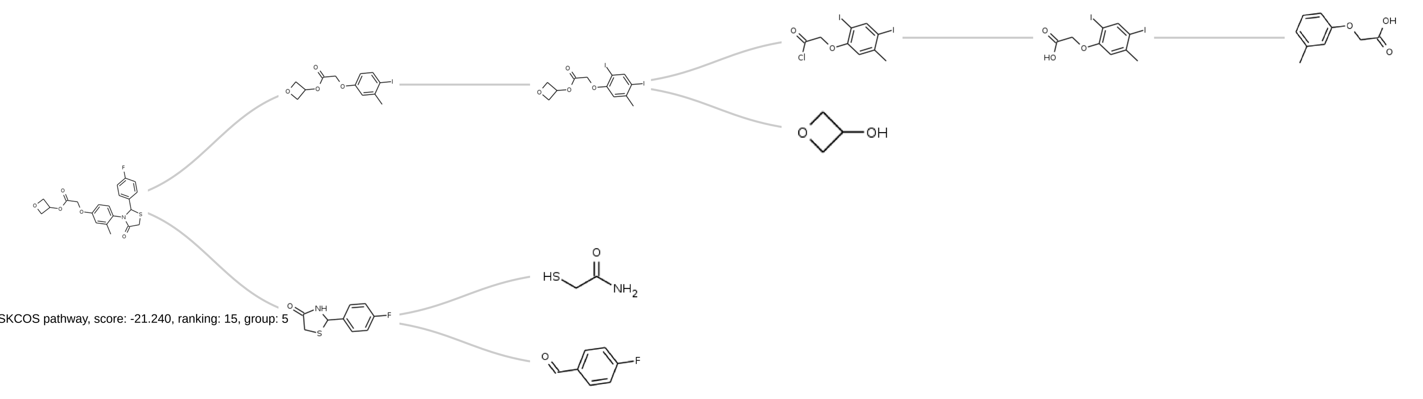

ASKCOS pathway, score: -21.240, ranking: 15, group: 5

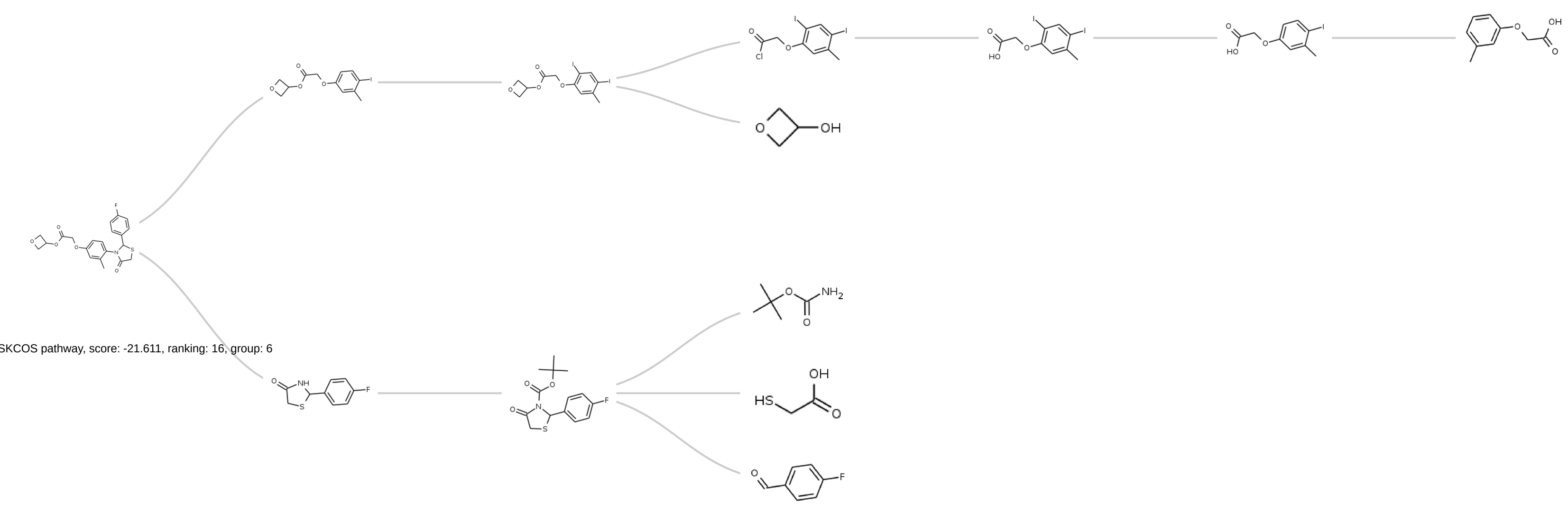

ASKCOS pathway, score: -21.611, ranking: 16, group: 6

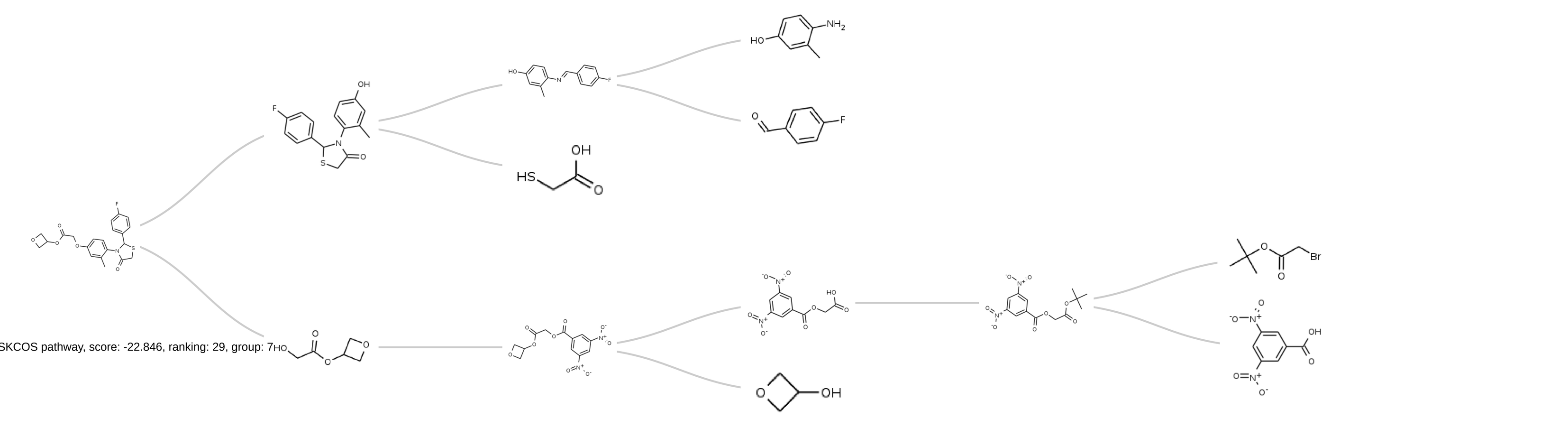

ASKCOS pathway, score: -22.846, ranking: 29, group: 7

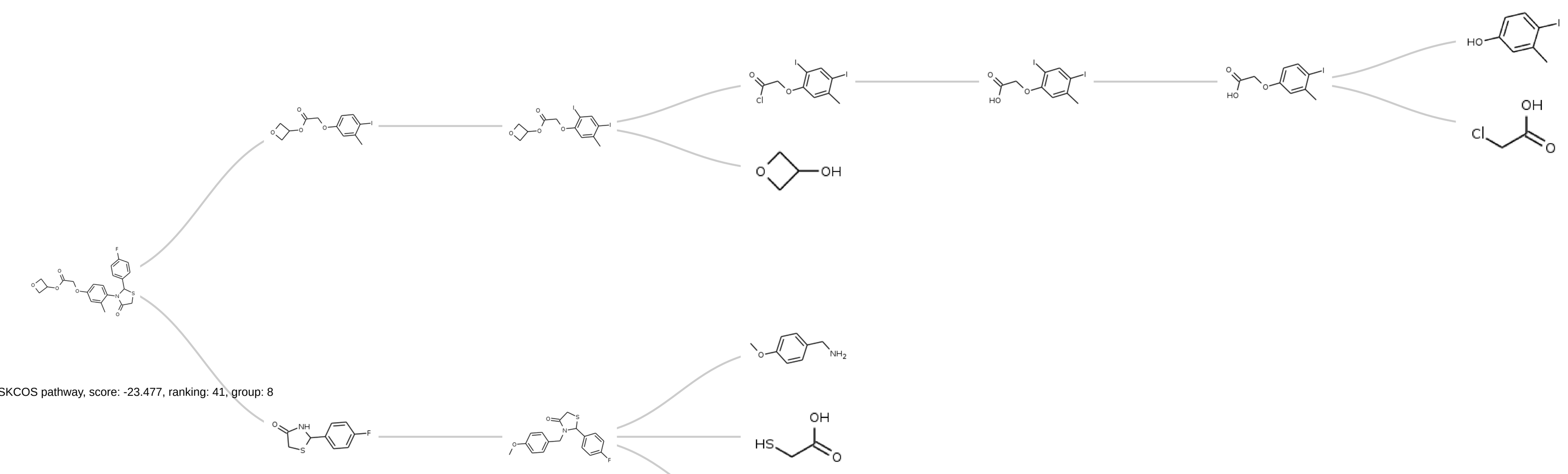

ASKCOS pathway, score: -23.477, ranking: 41, group: 8

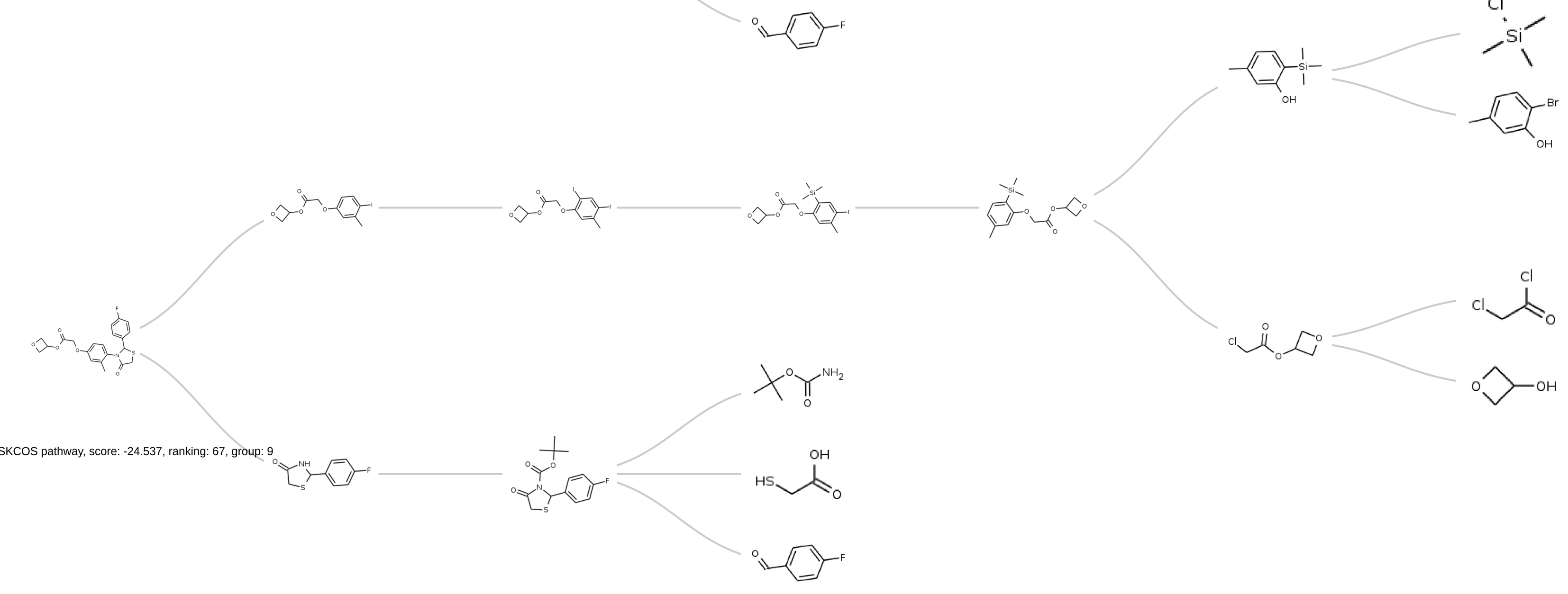

ASKCOS pathway, score: -24.537, ranking: 67, group: 9

Model ranks patent pathway as top-1: Example 11

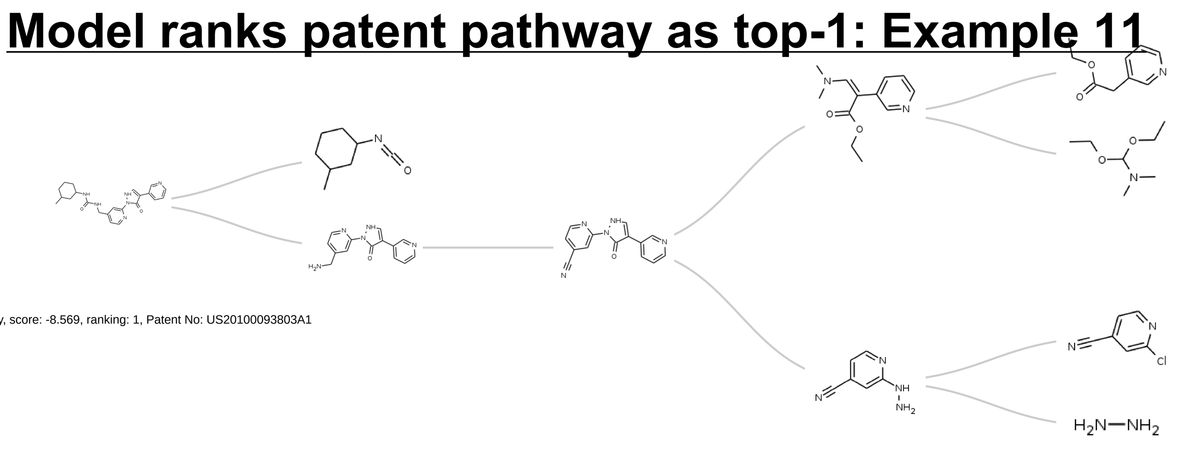

Piatachio pathway, score: -8.569, ranking: 1, Patent No: US20100093803A1

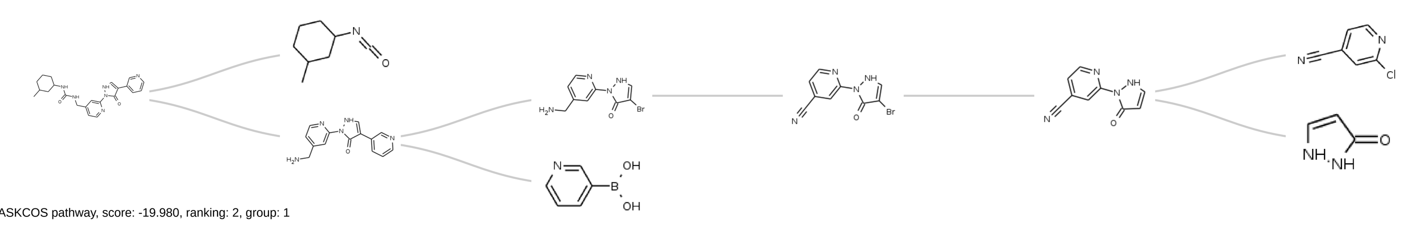

ASKCOS pathway, score: -19.980, ranking: 2, group: 1

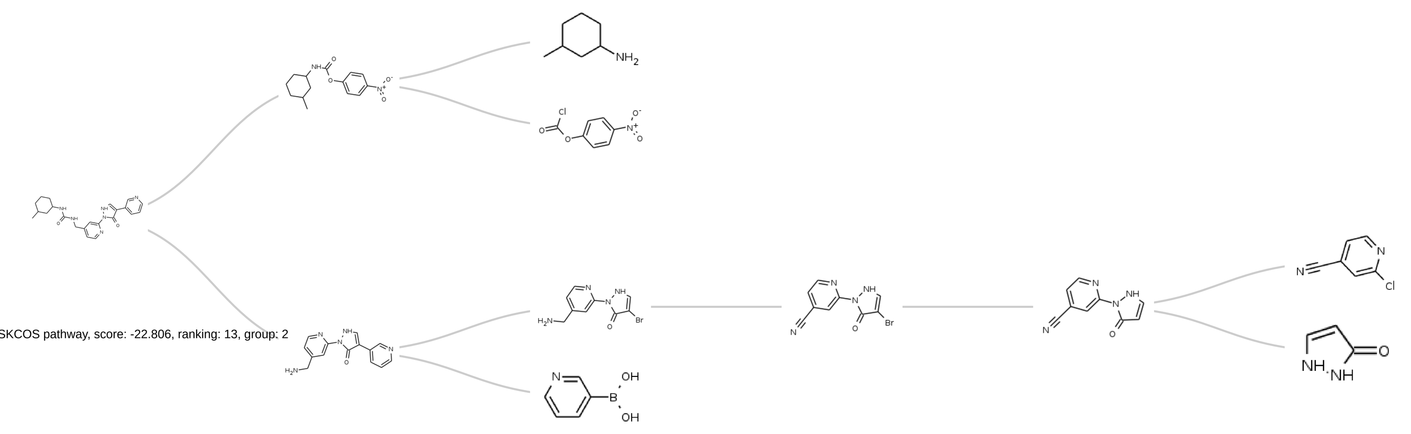

ASKCOS pathway, score: -22.806, ranking: 13, group: 2

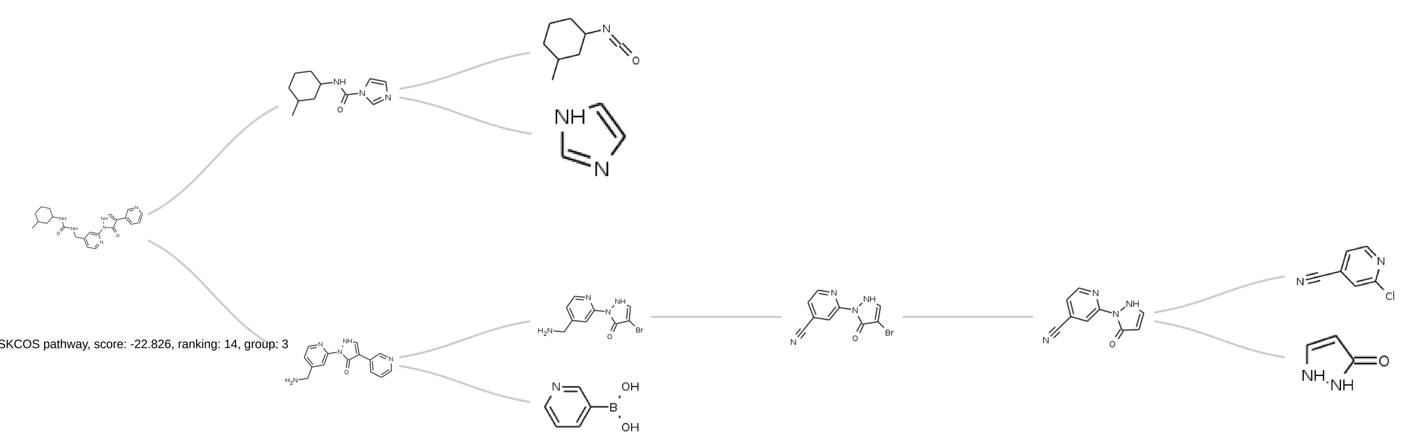

ASKCOS pathway, score: -22.826, ranking: 14, group: 3

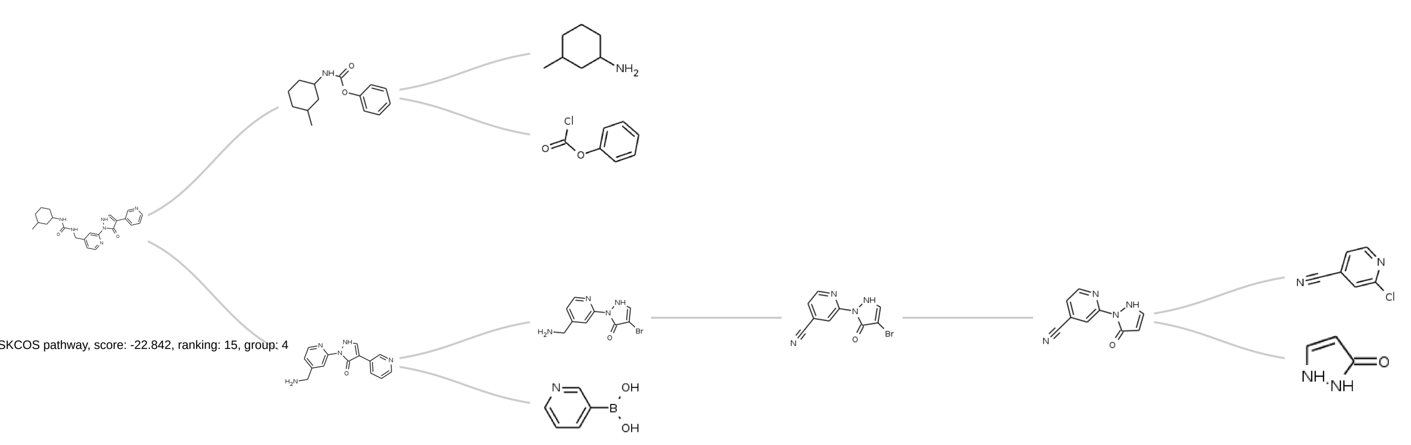

ASKCOS pathway, score: -22.842, ranking: 15, group: 4

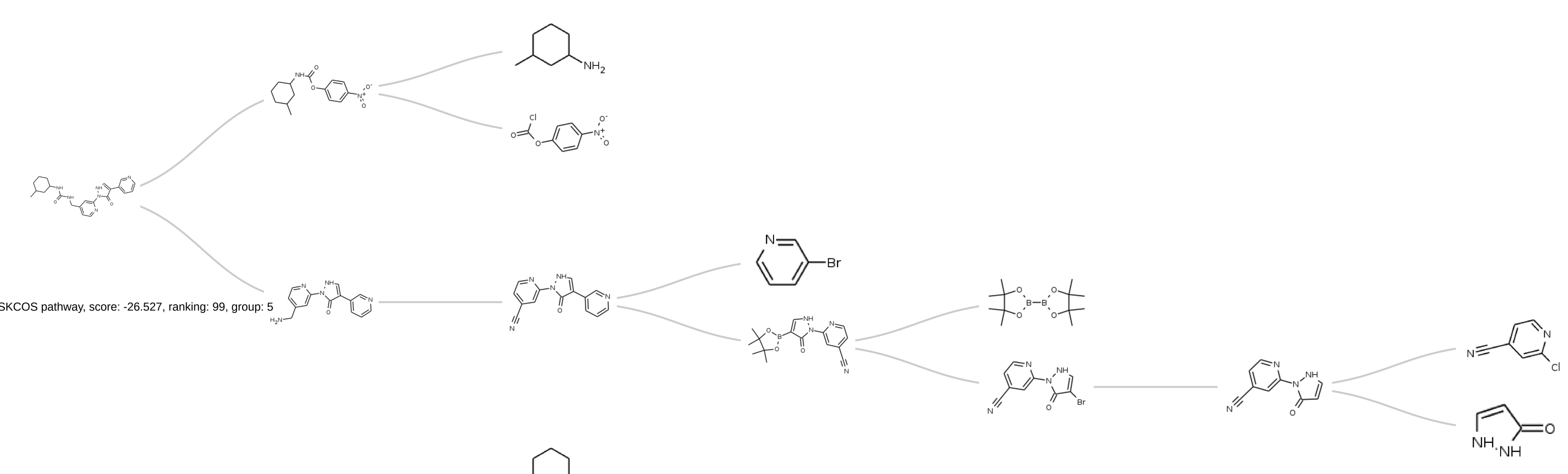

ASKCOS pathway, score: -26.527, ranking: 99, group: 5

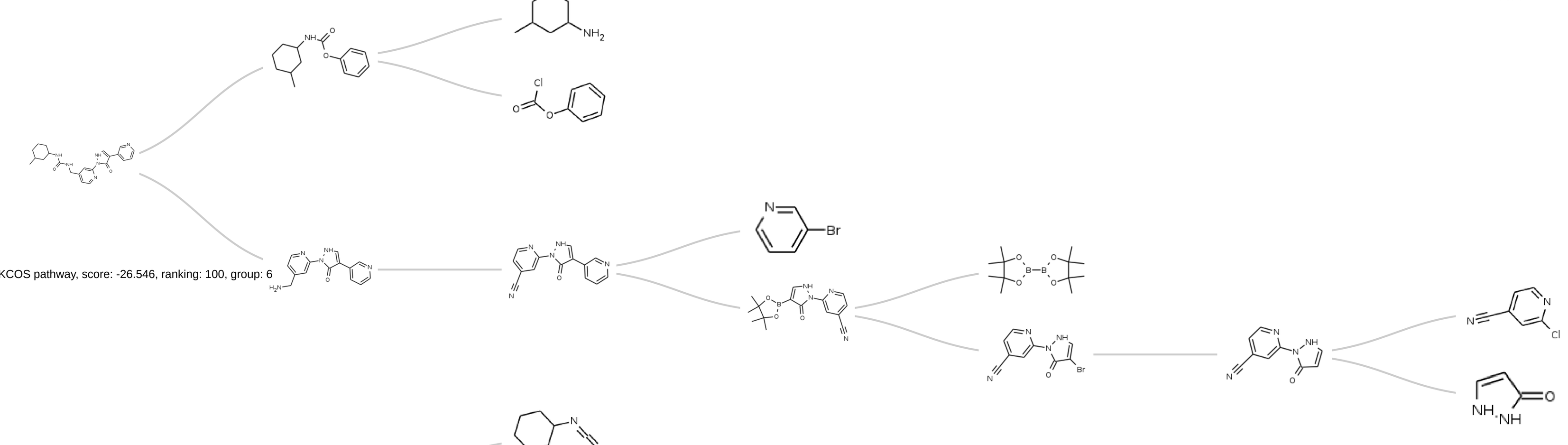

ASKCOS pathway, score: -26.546, ranking: 100, group: 6

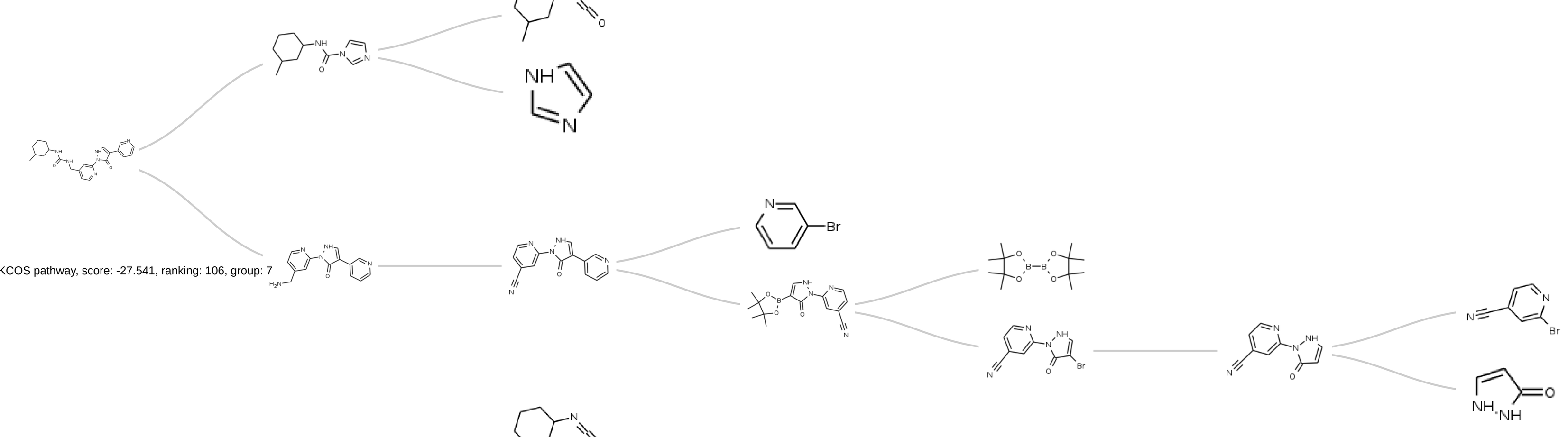

ASKCOS pathway, score: -27.541, ranking: 106, group: 7

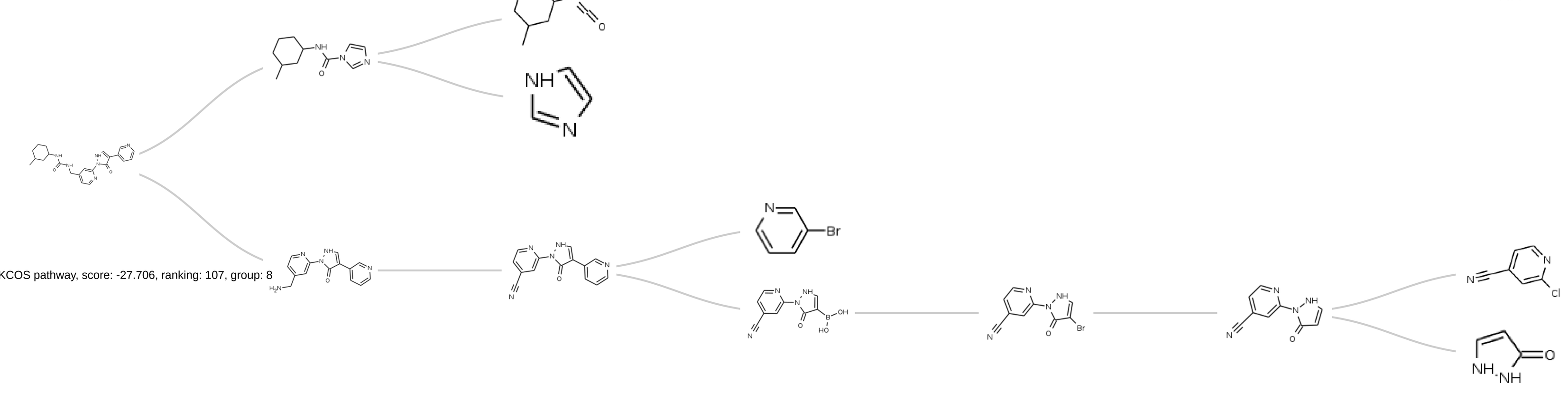

ASKCOS pathway, score: -27.706, ranking: 107, group: 8

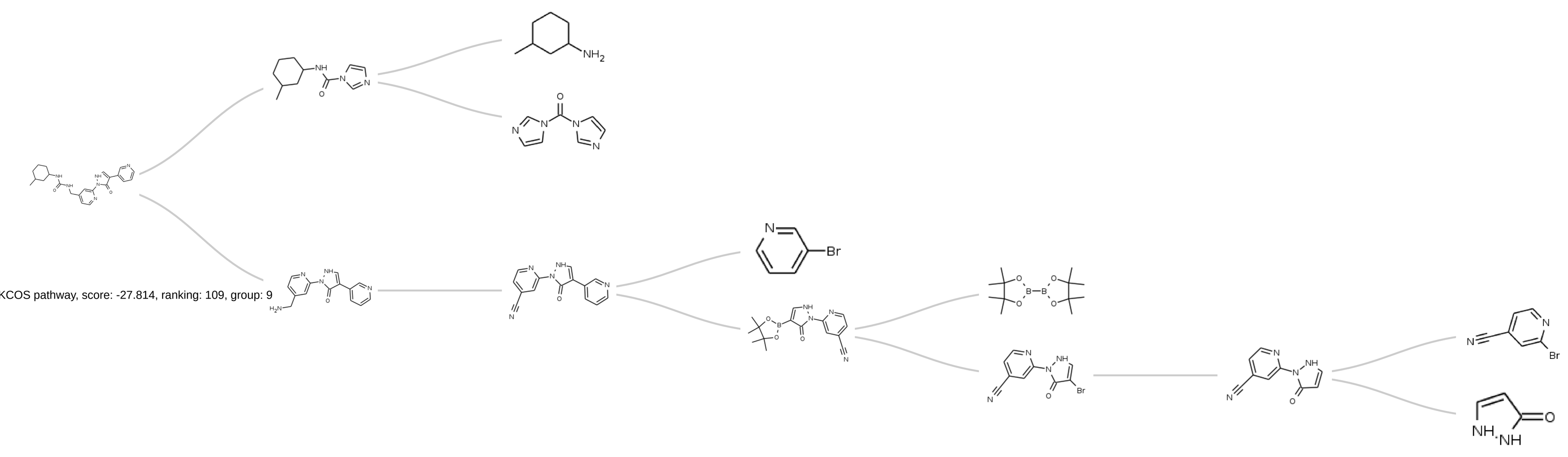

ASKCOS pathway, score: -27.814, ranking: 109, group: 9

Model ranks patent pathway as top-1: Example 12

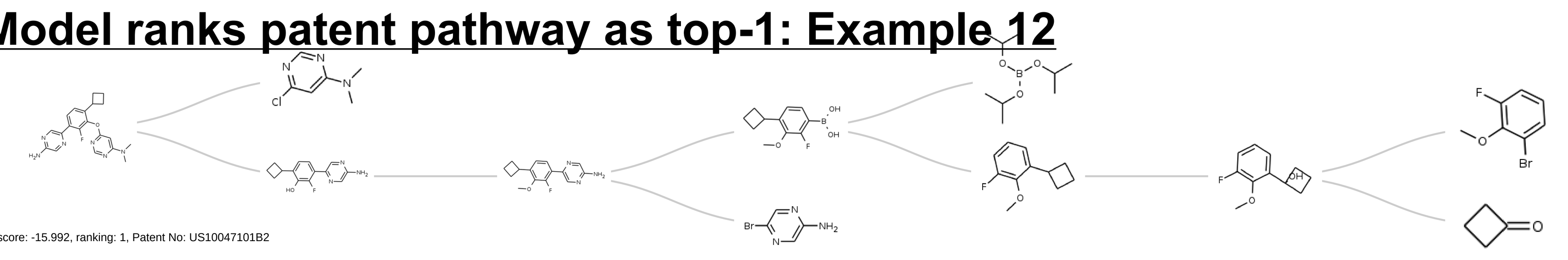

Piatachio pathway, score: -15.992, ranking: 1, Patent No: US10047101B2

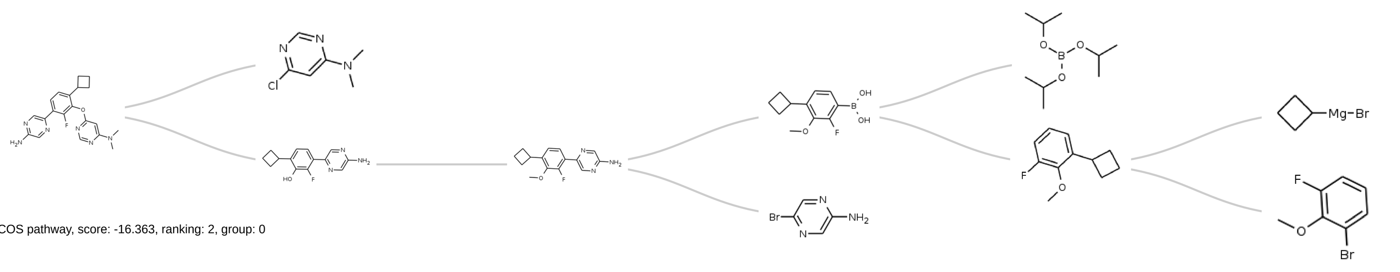

ASKCOS pathway, score: -16.363, ranking: 2, group: 0

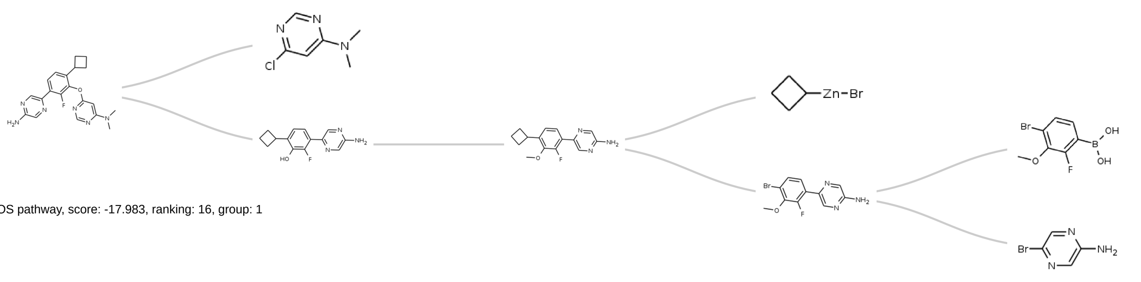

ASKCOS pathway, score: -17.983, ranking: 16, group: 1

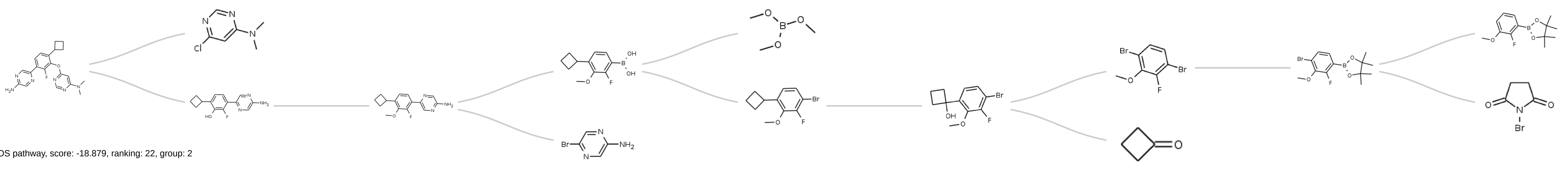

ASKCOS pathway, score: -18.879, ranking: 22, group: 2

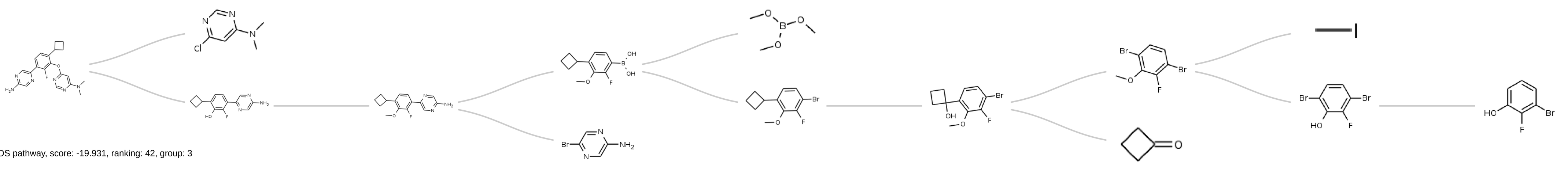

ASKCOS pathway, score: -19.931, ranking: 42, group: 3

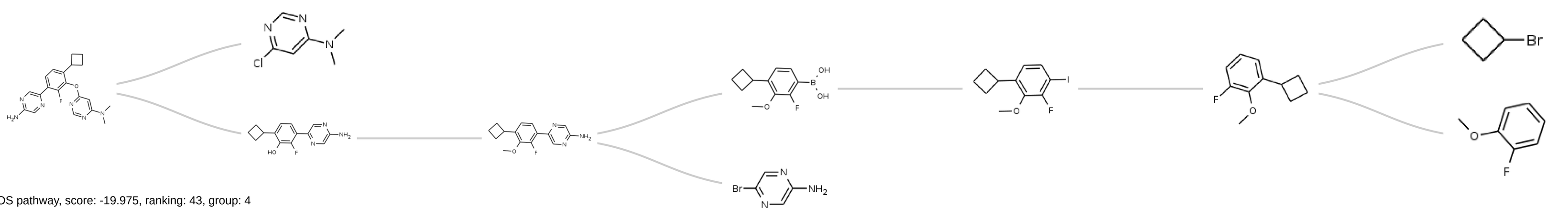

ASKCOS pathway, score: -19.975, ranking: 43, group: 4

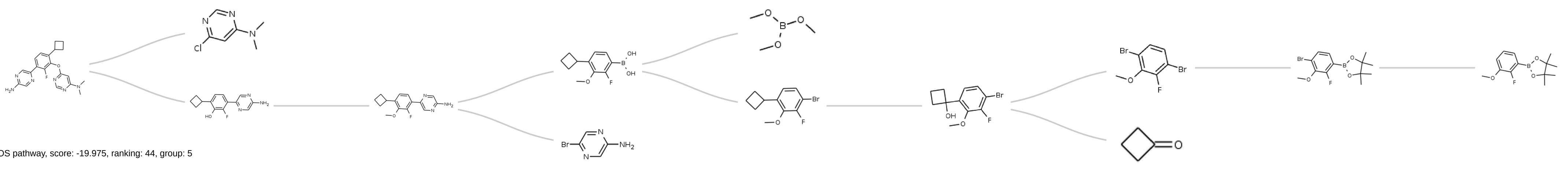

ASKCOS pathway, score: -19.975, ranking: 44, group: 5

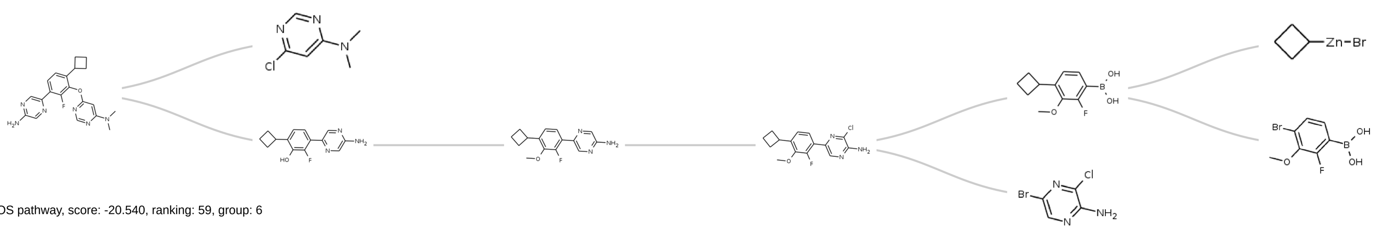

ASKCOS pathway, score: -20.540, ranking: 59, group: 6

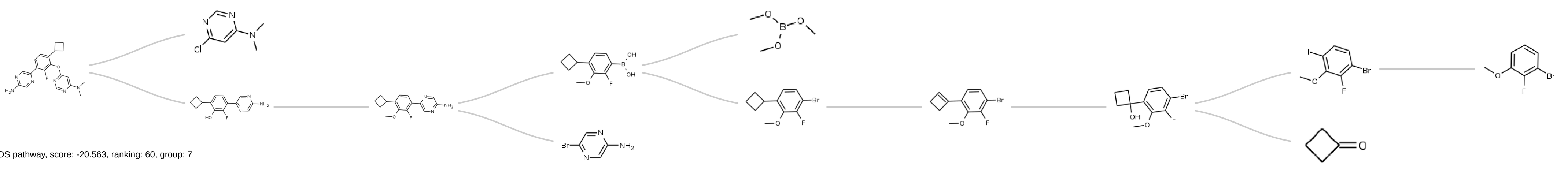

ASKCOS pathway, score: -20.563, ranking: 60, group: 7

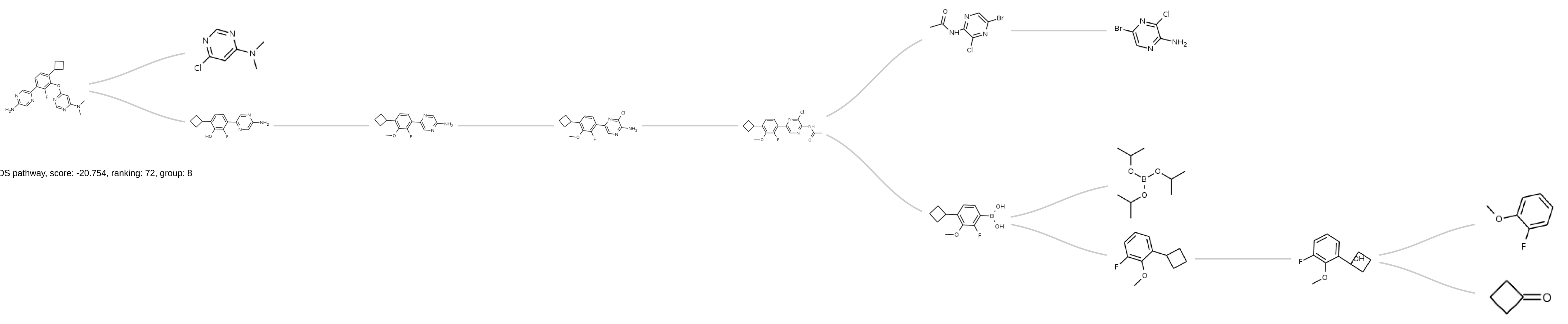

ASKCOS pathway, score: -20.754, ranking: 72, group: 8

Model ranks patent pathway as top-1: Example 13

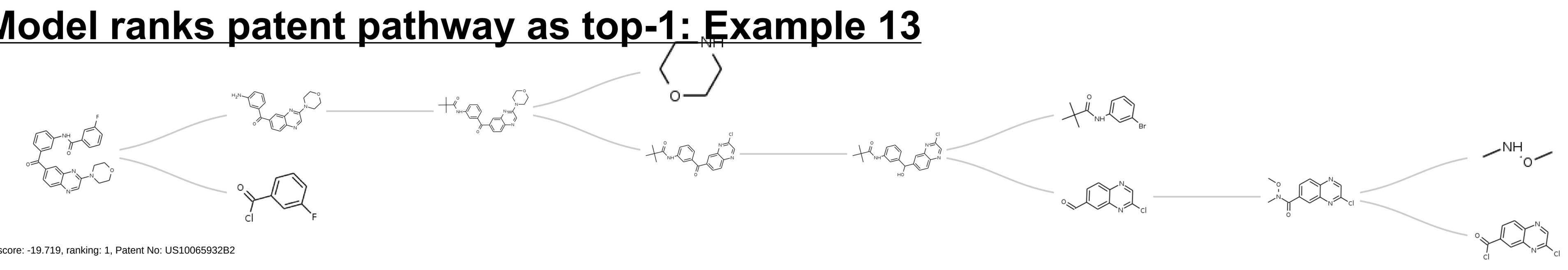

Piatachio pathway, score: -19.719, ranking: 1, Patent No: US10065932B2

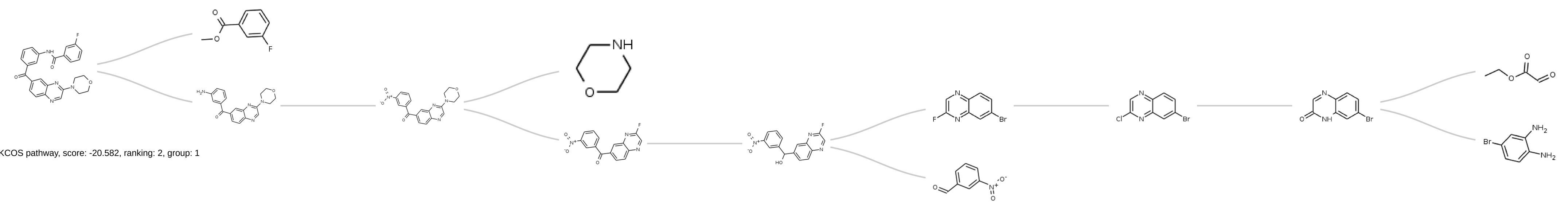

ASKCOS pathway, score: -20.582, ranking: 2, group: 1

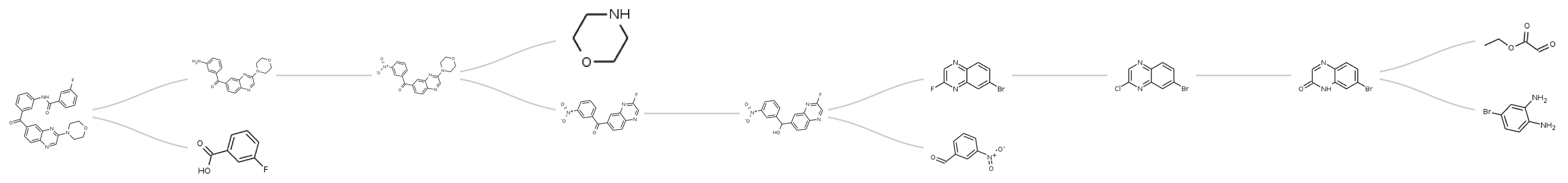

ASKCOS pathway, score: -21.239, ranking: 3, group: 2

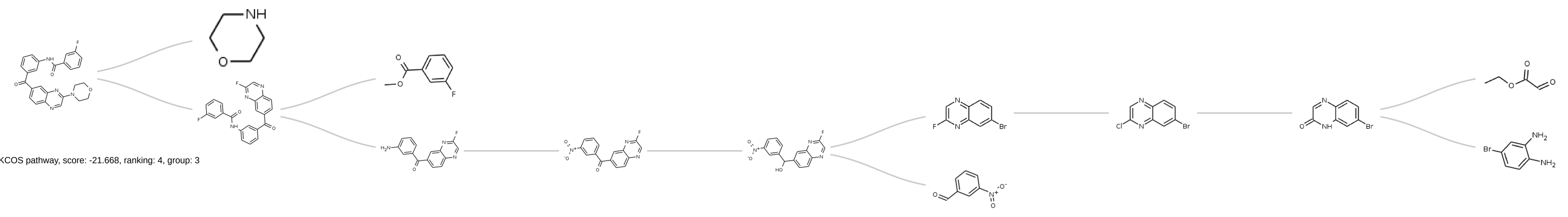

ASKCOS pathway, score: -21.668, ranking: 4, group: 3

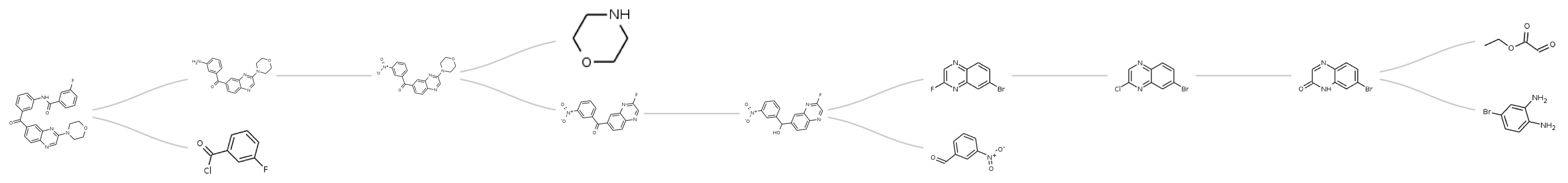

ASKCOS pathway, score: -21.738, ranking: 5, group: 4

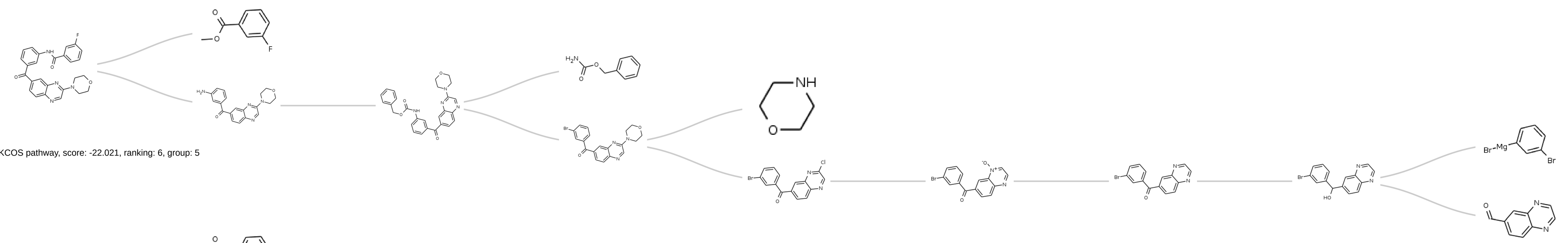

ASKCOS pathway, score: -22.021, ranking: 6, group: 5

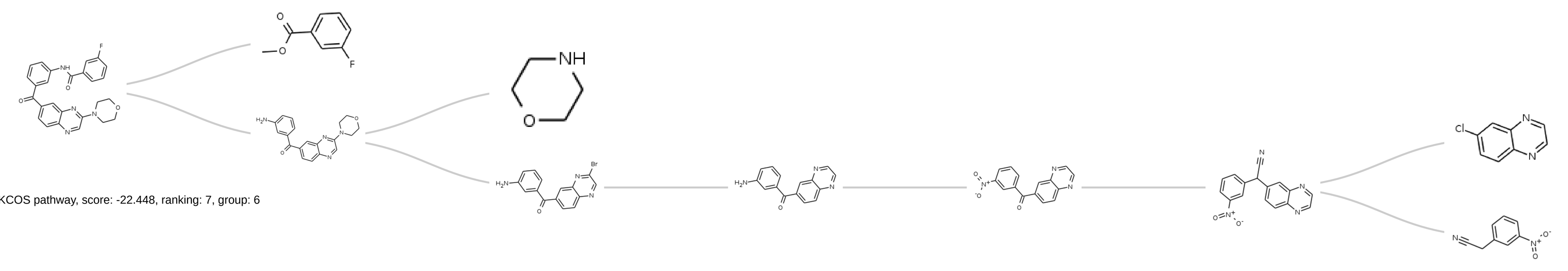

ASKCOS pathway, score: -22.448, ranking: 7, group: 6

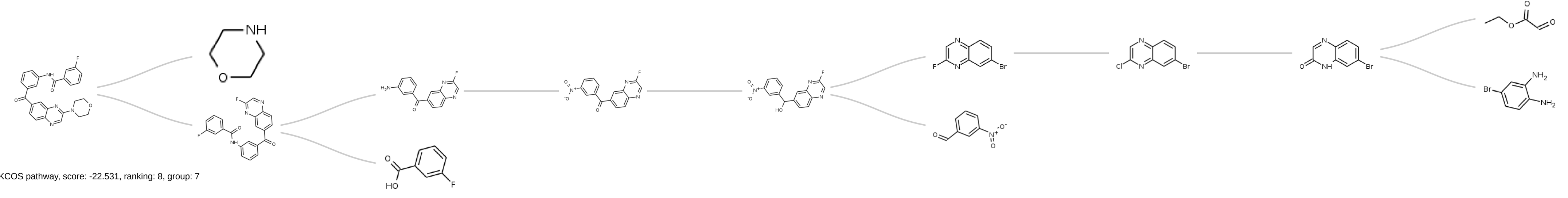

ASKCOS pathway, score: -22.531, ranking: 8, group: 7

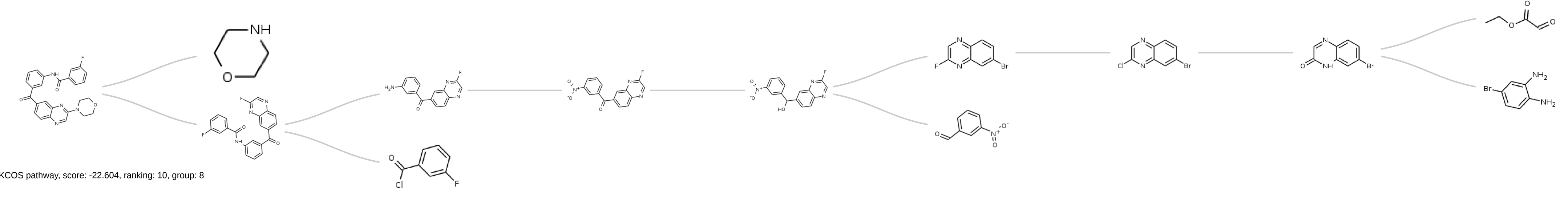

ASKCOS pathway, score: -22.604, ranking: 10, group: 8

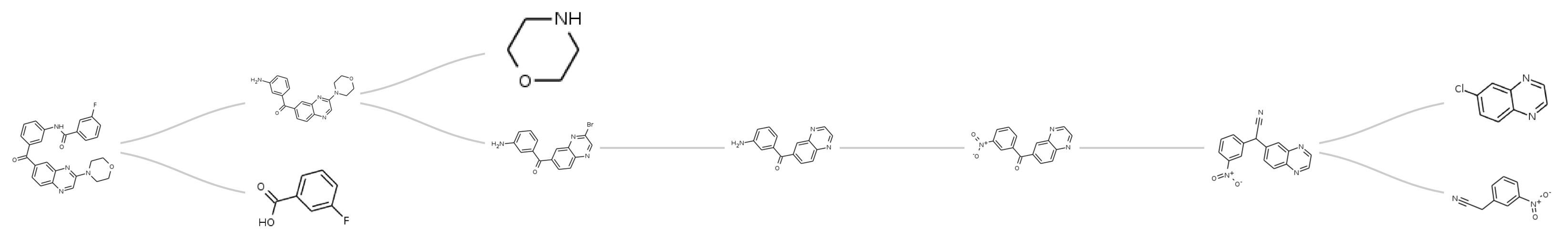

ASKCOS pathway, score: -23.035, ranking: 13, group: 9

Model ranks patent pathway as top-1: Example 14

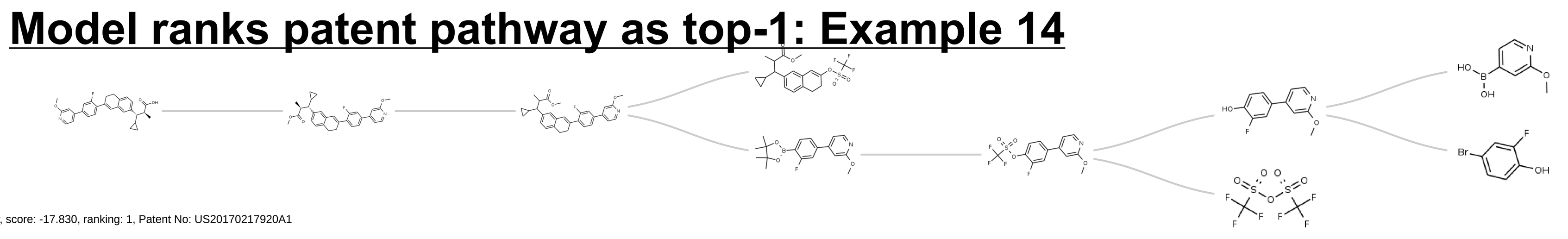

Piatachio pathway, score: -17.830, ranking: 1, Patent No: US20170217920A1

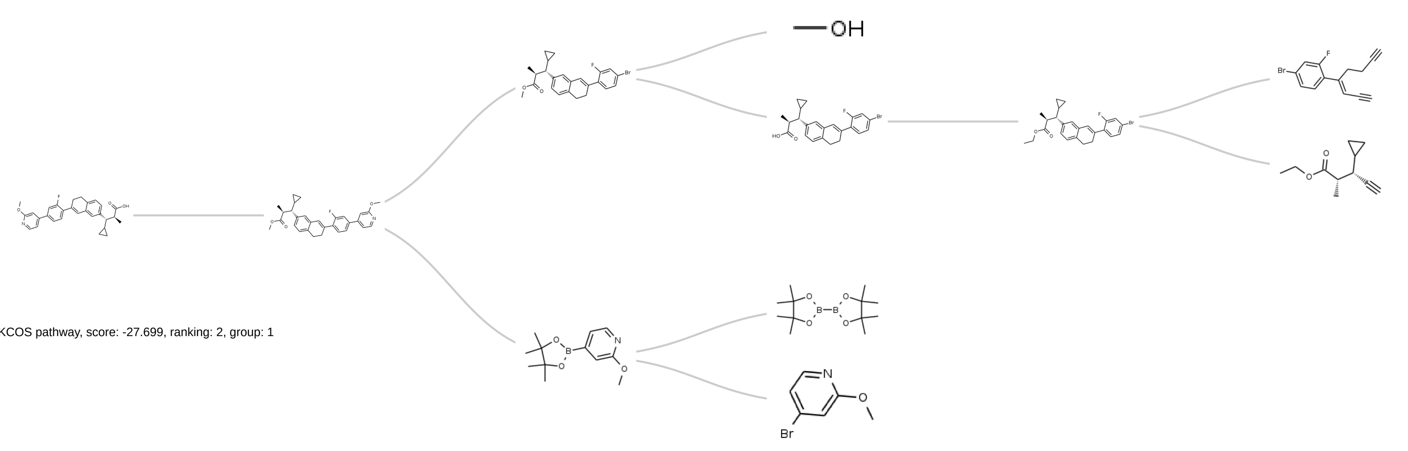

ASKCOS pathway, score: -27.699, ranking: 2, group: 1

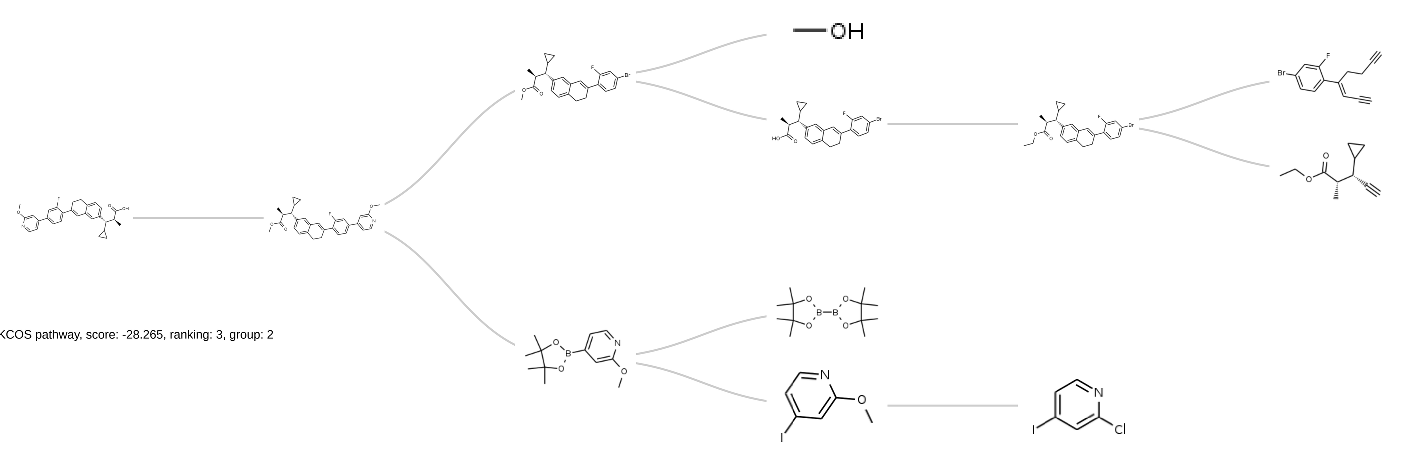

ASKCOS pathway, score: -28.265, ranking: 3, group: 2

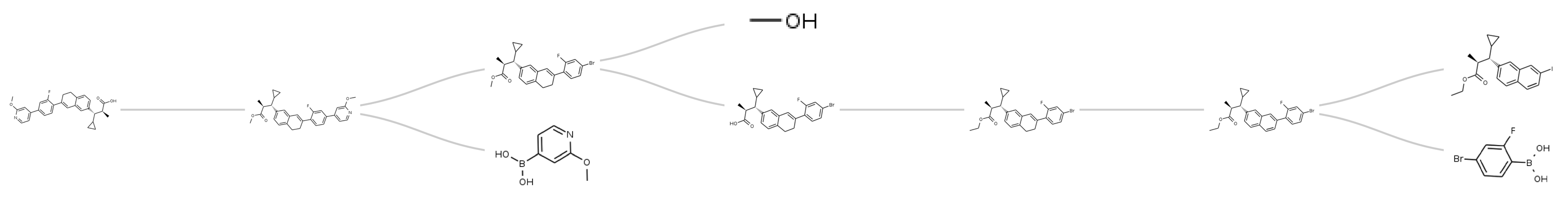

ASKCOS pathway, score: -28.316, ranking: 4, group: 3

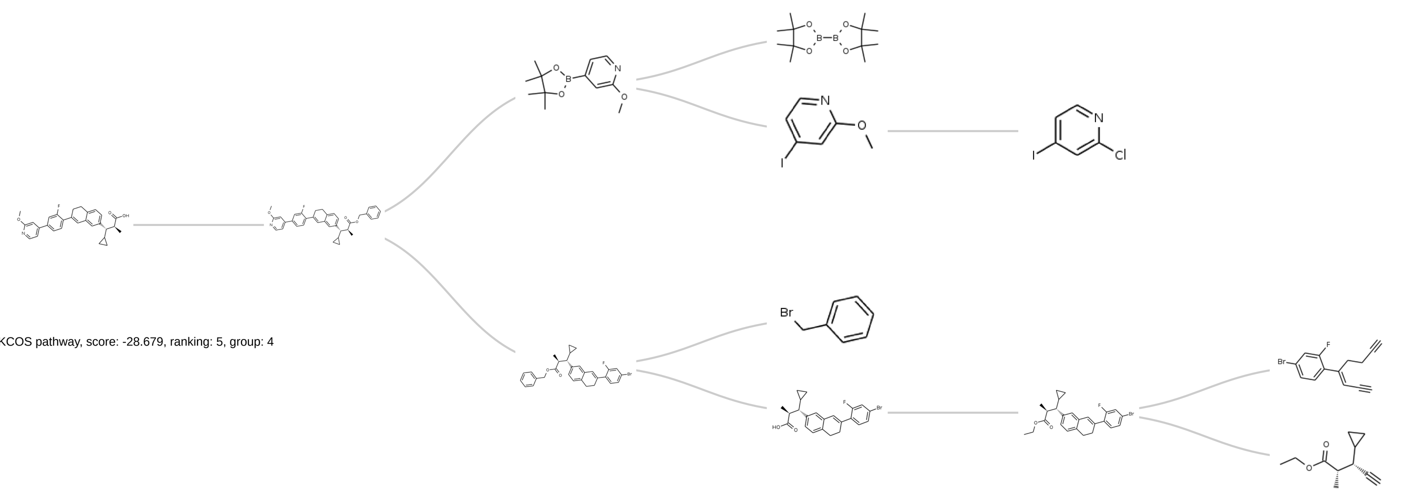

ASKCOS pathway, score: -28.679, ranking: 5, group: 4

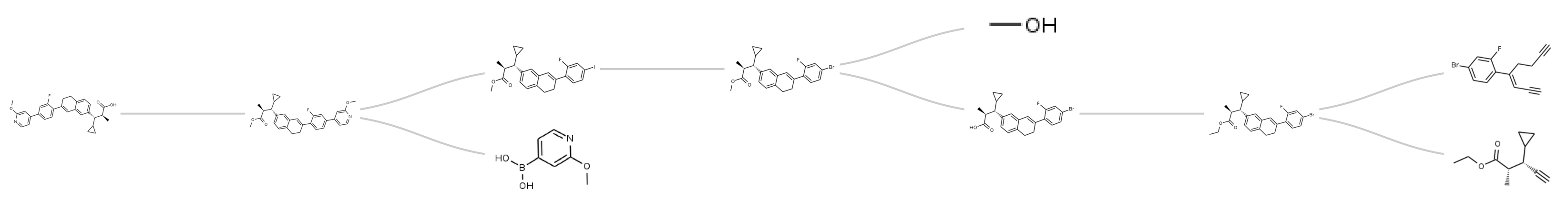

ASKCOS pathway, score: -28.929, ranking: 6, group: 5

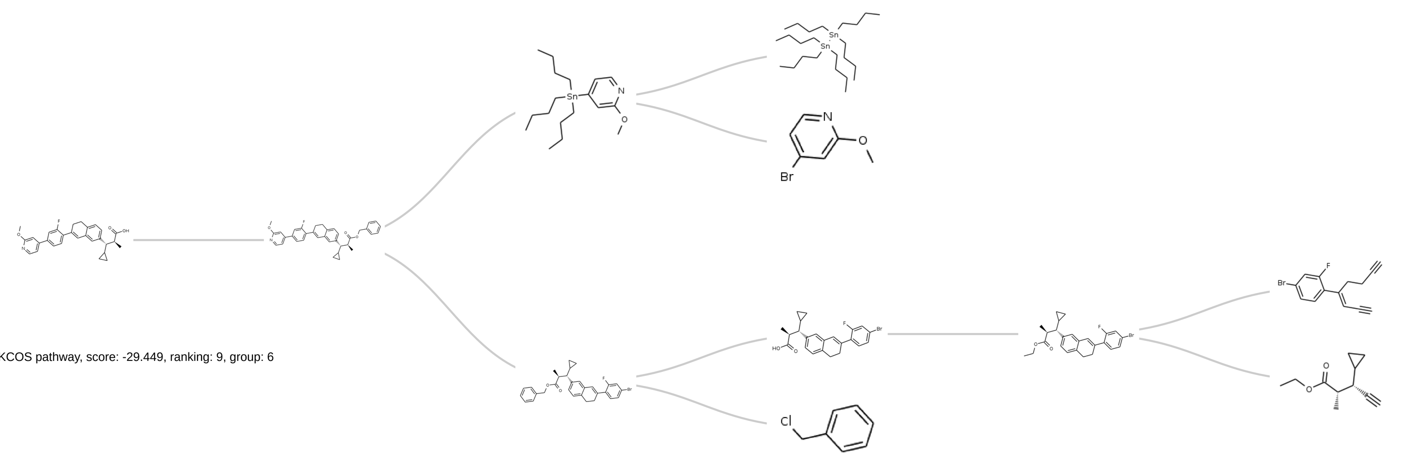

ASKCOS pathway, score: -29.449, ranking: 9, group: 6

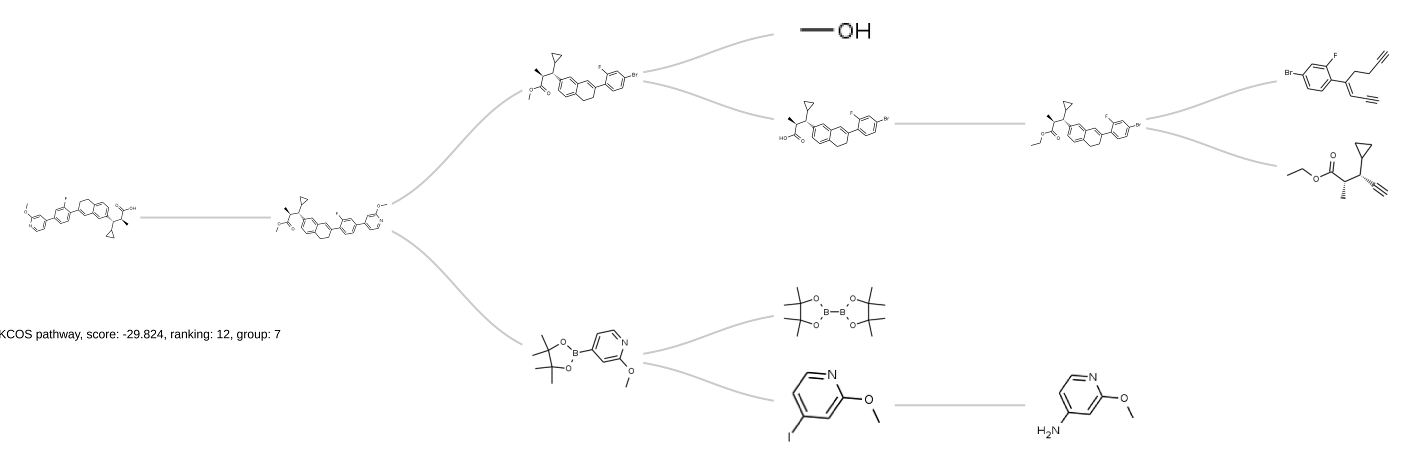

ASKCOS pathway, score: -29.824, ranking: 12, group: 7

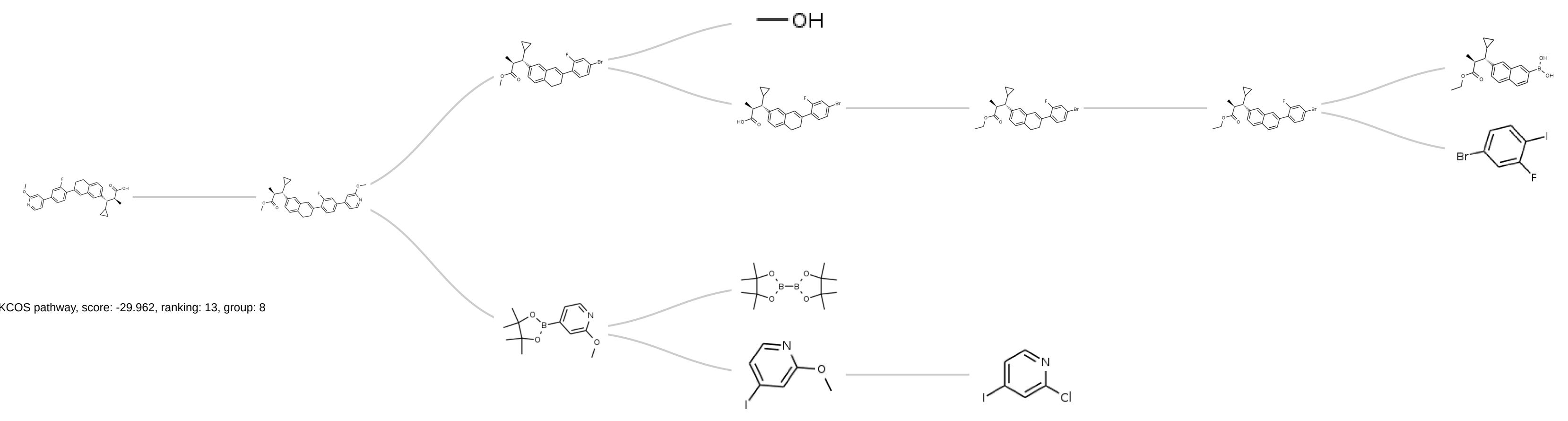

ASKCOS pathway, score: -29.962, ranking: 13, group: 8

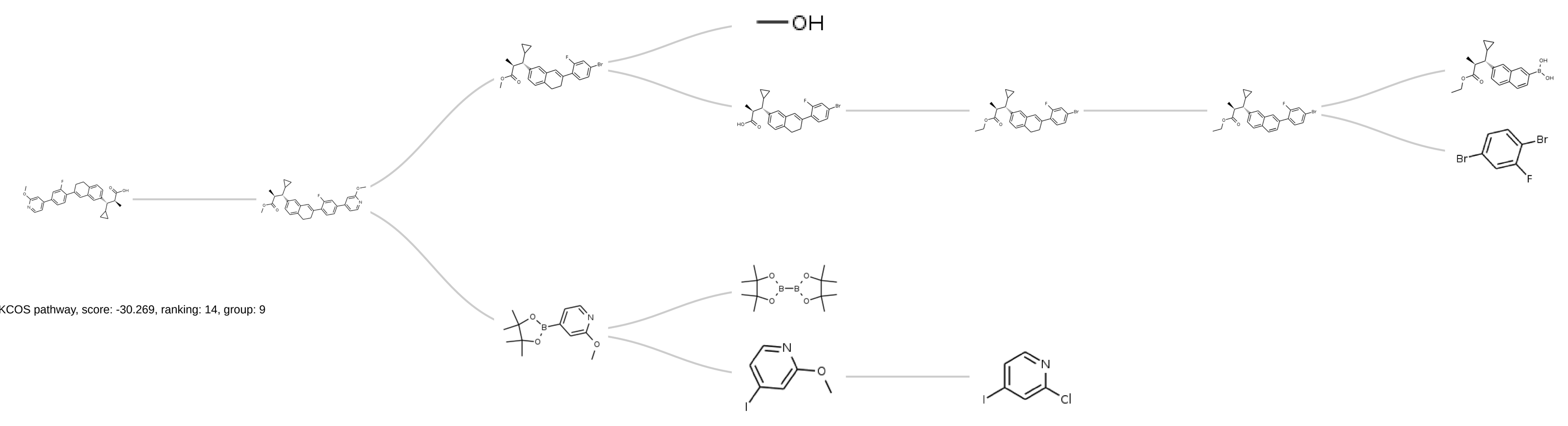

ASKCOS pathway, score: -30.269, ranking: 14, group: 9

Model ranks patent pathway as top-1: Example 15

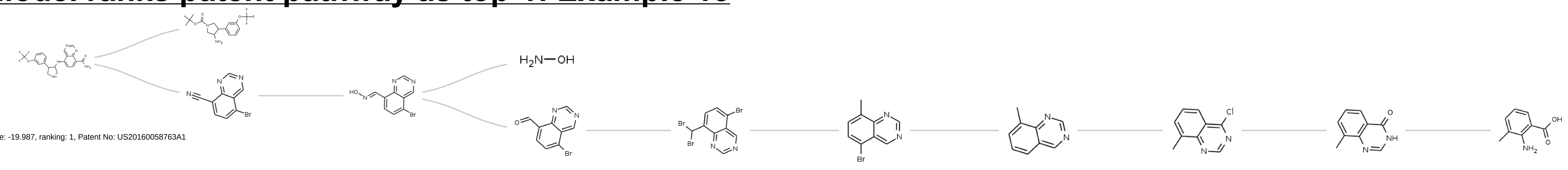

Piatachio pathway, score: -19.987, ranking: 1, Patent No: US20160058763A1

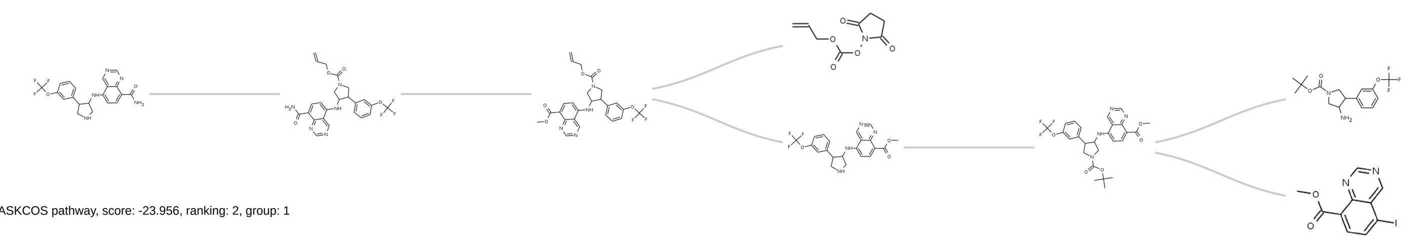

ASKCOS pathway, score: -23.956, ranking: 2, group: 1

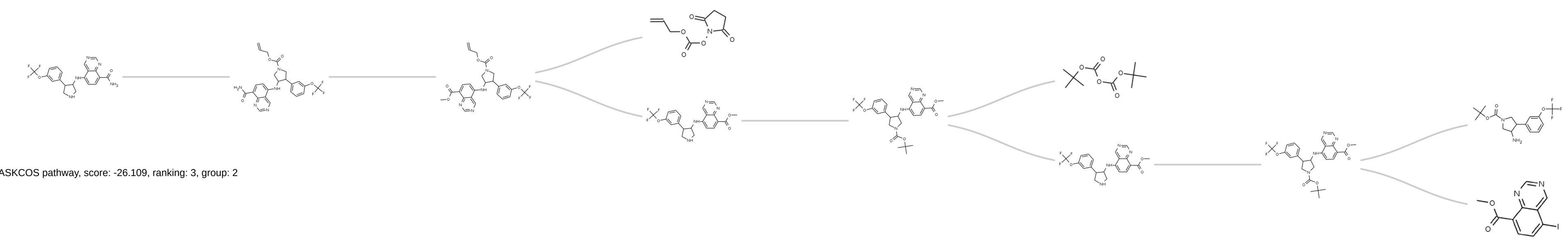

ASKCOS pathway, score: -26.109, ranking: 3, group: 2

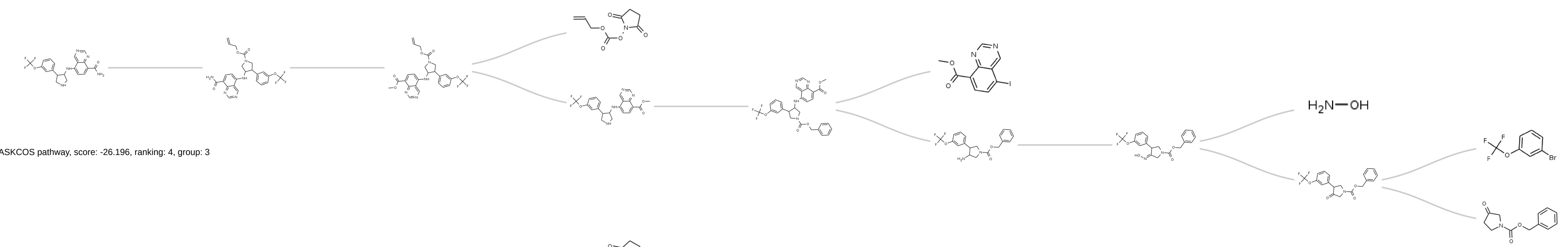

ASKCOS pathway, score: -26.196, ranking: 4, group: 3

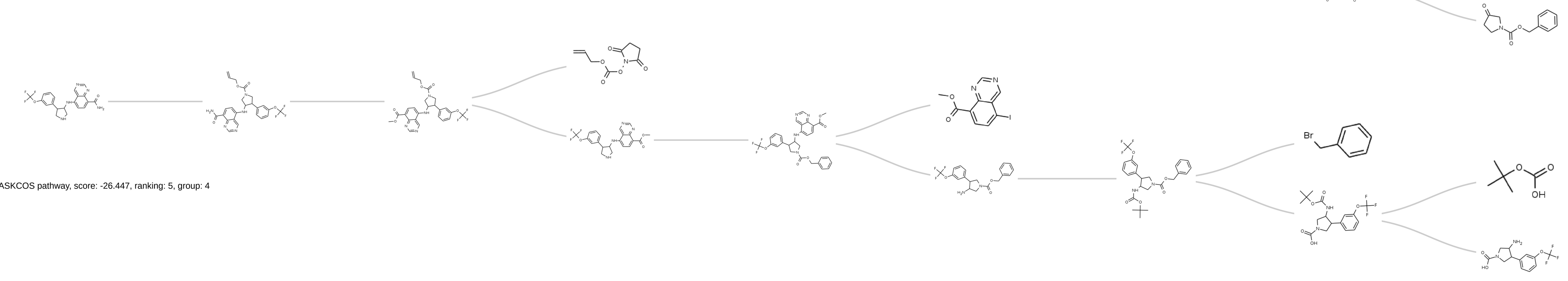

ASKCOS pathway, score: -26.447, ranking: 5, group: 4

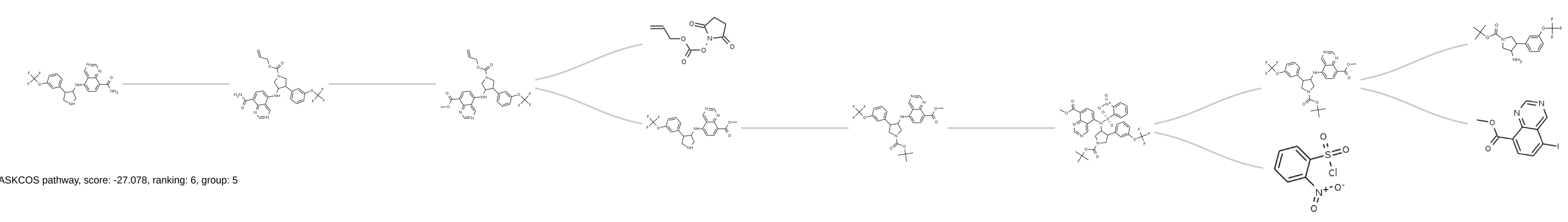

ASKCOS pathway, score: -27.078, ranking: 6, group: 5

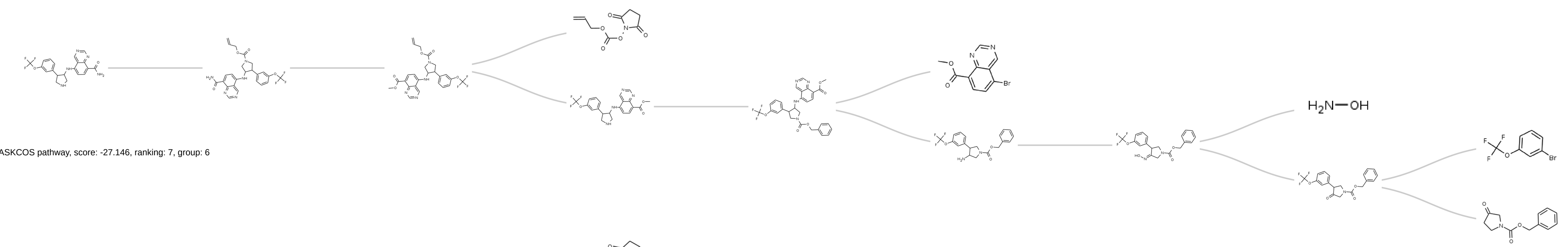

ASKCOS pathway, score: -27.146, ranking: 7, group: 6

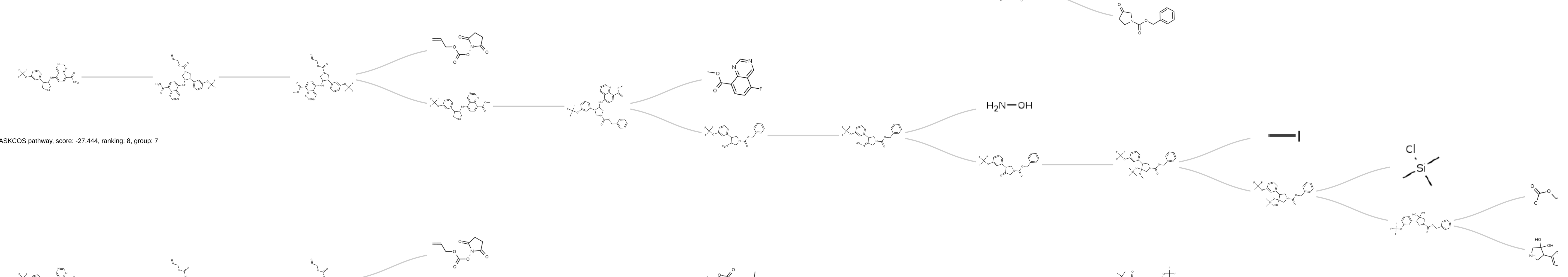

ASKCOS pathway, score: -27.444, ranking: 8, group: 7

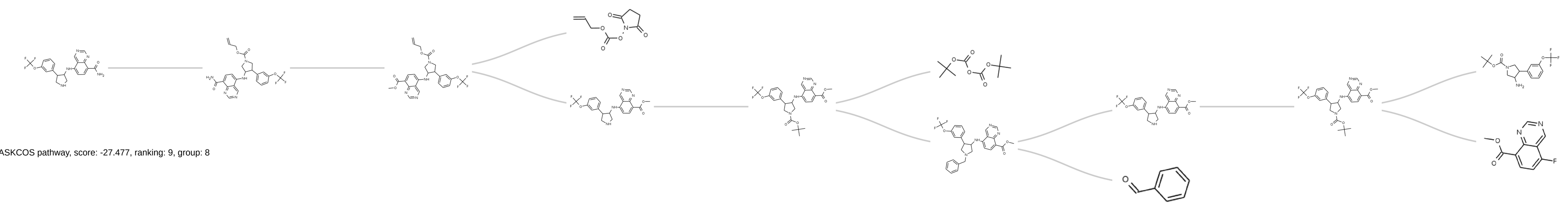

ASKCOS pathway, score: -27.477, ranking: 9, group: 8

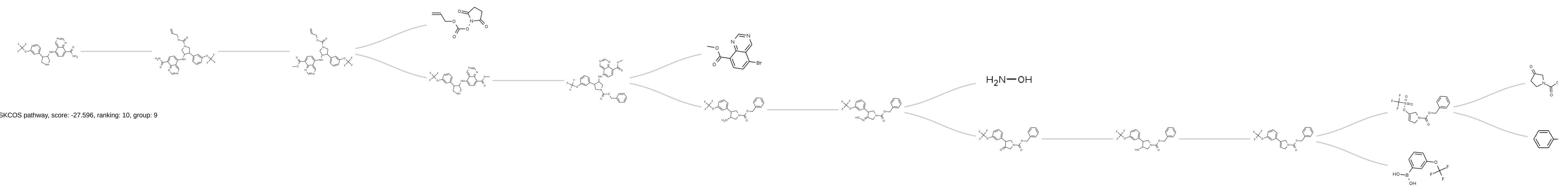

ASKCOS pathway, score: -27.596, ranking: 10, group: 9

Model ranks patent pathway as top-1: Example 16

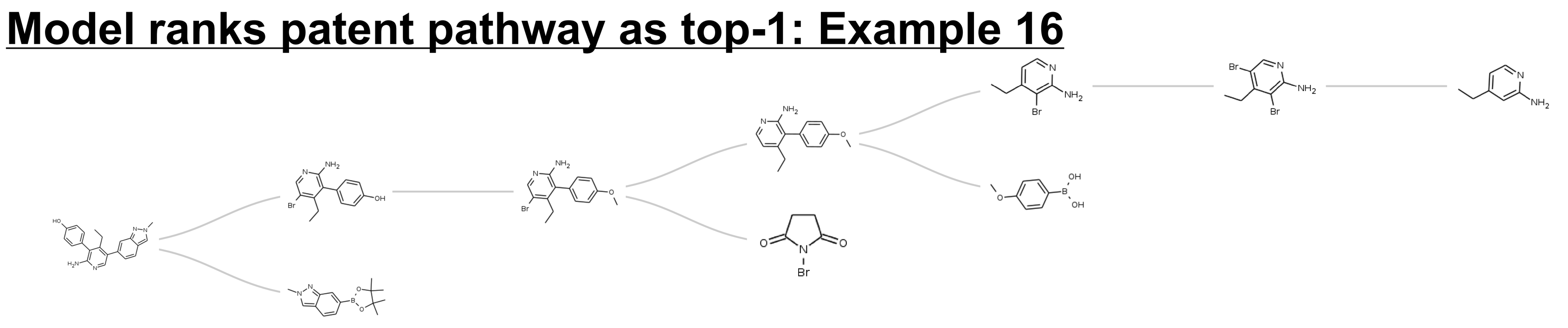

Piatachio pathway, score: -11.915, ranking: 1, Patent No: US09573899

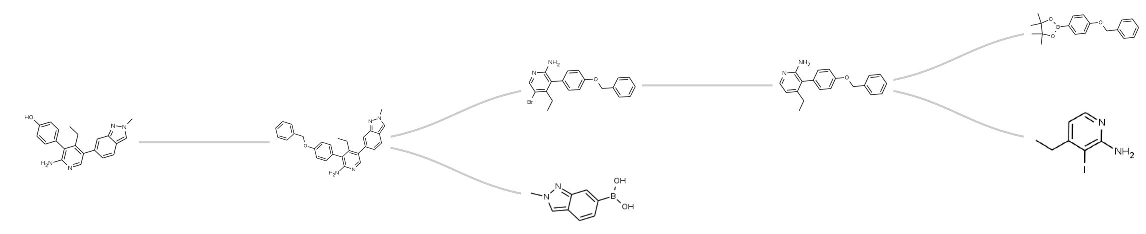

ASKCOS pathway, score: -17.347, ranking: 2, group: 1

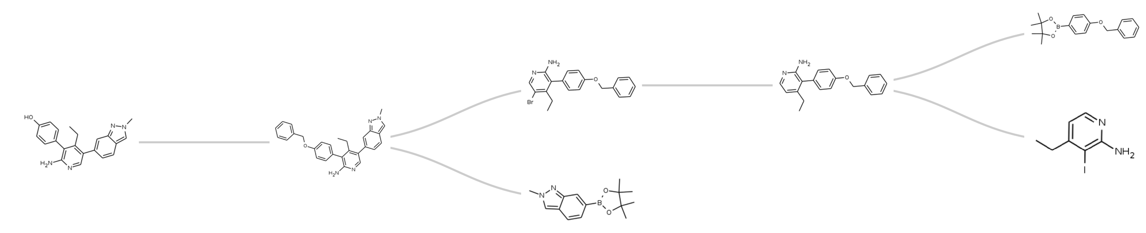

ASKCOS pathway, score: -18.007, ranking: 3, group: 2

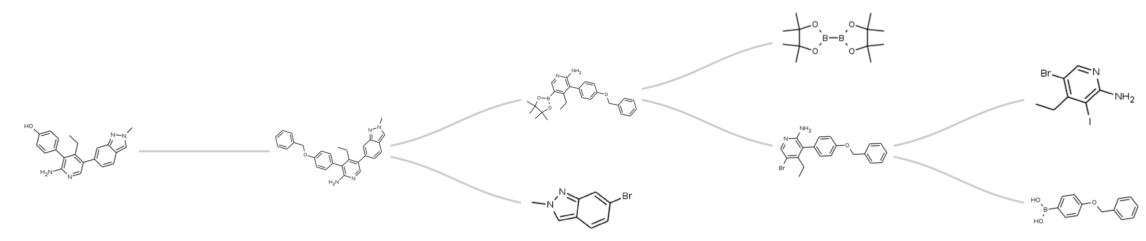

ASKCOS pathway, score: -18.584, ranking: 4, group: 3

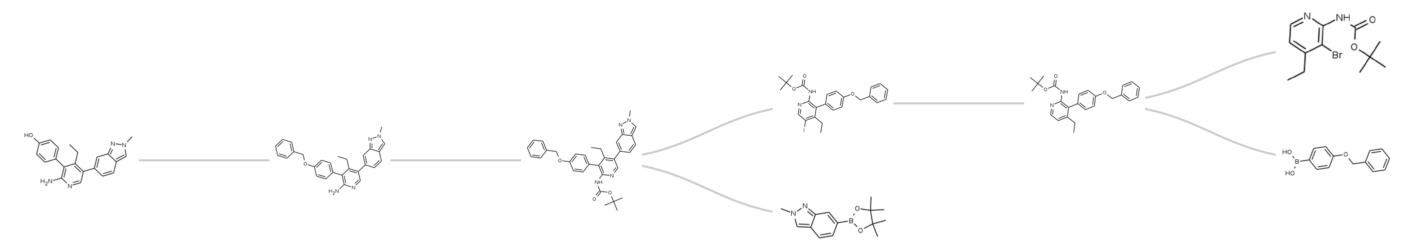

ASKCOS pathway, score: -19.499, ranking: 9, group: 4

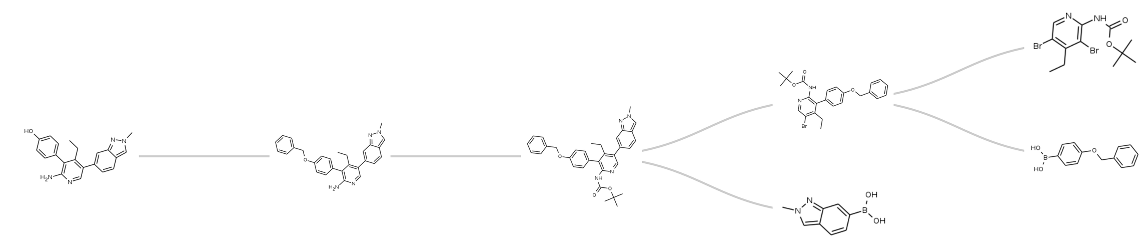

ASKCOS pathway, score: -19.536, ranking: 10, group: 5

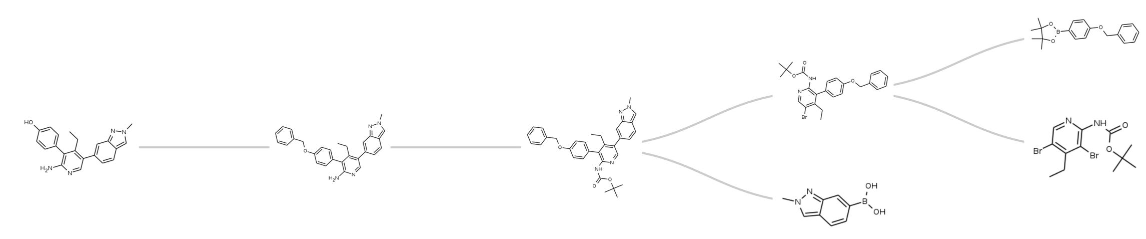

ASKCOS pathway, score: -20.171, ranking: 13, group: 6

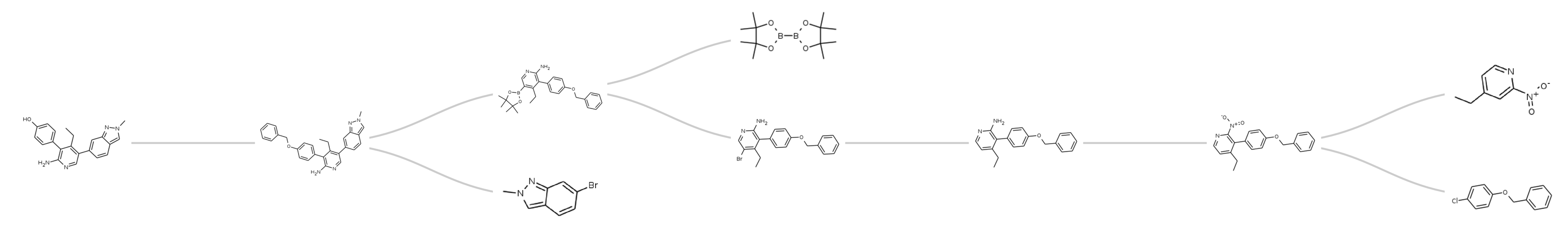

ASKCOS pathway, score: -20.520, ranking: 17, group: 7

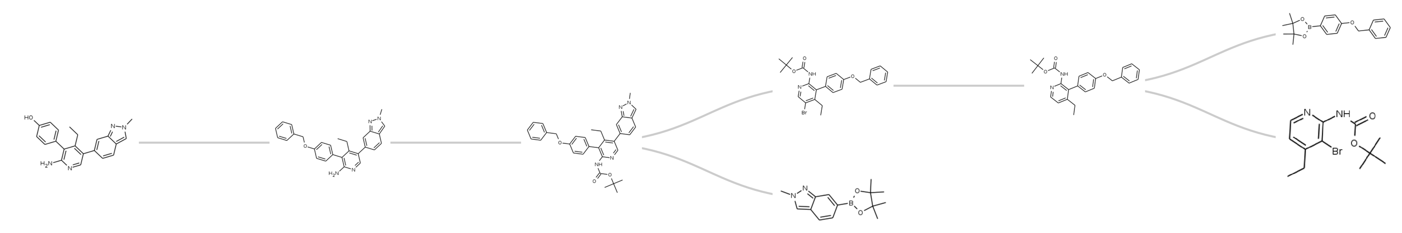

ASKCOS pathway, score: -20.759, ranking: 19, group: 8

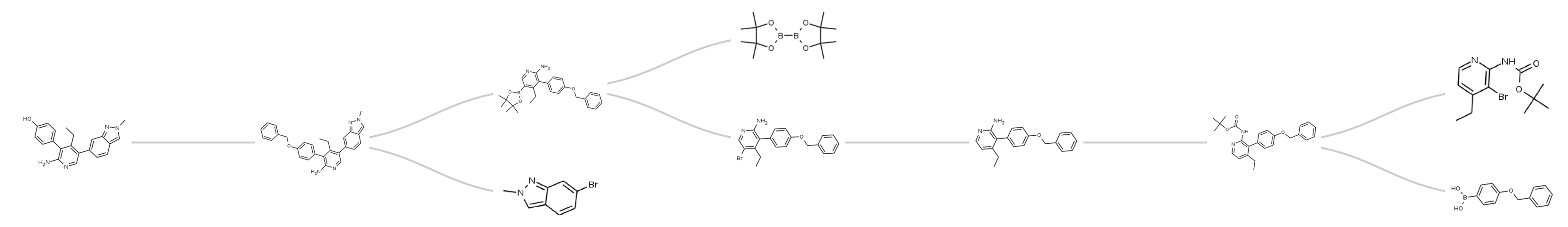

ASKCOS pathway, score: -20.827, ranking: 21, group: 9

Model ranks patent pathway as top-1: Example 17

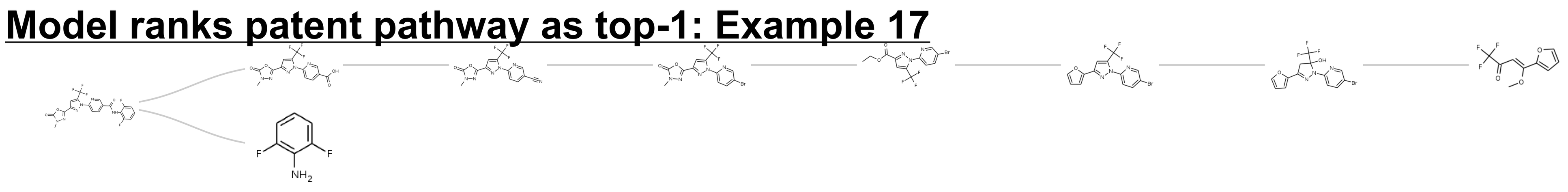

Piatachio pathway, score: -14.001, ranking: 1, Patent No: US20150111925A1

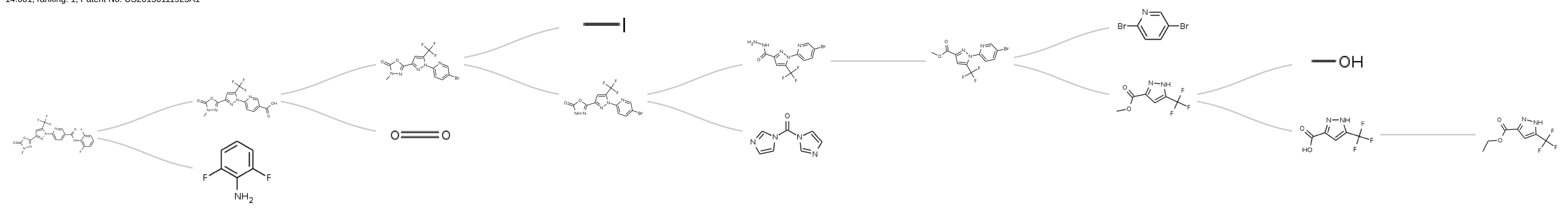

ASKCOS pathway, score: -23.066, ranking: 2, group: 1

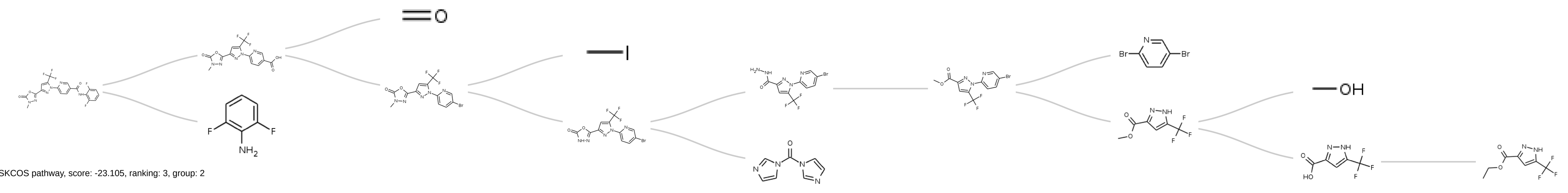

ASKCOS pathway, score: -23.105, ranking: 3, group: 2

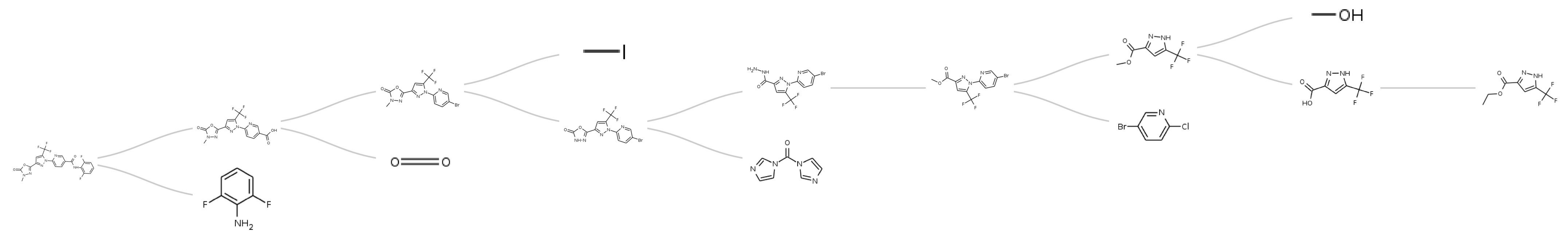

ASKCOS pathway, score: -23.890, ranking: 4, group: 3

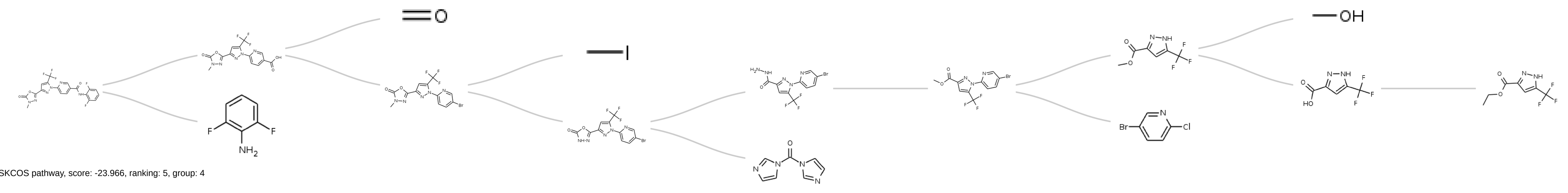

ASKCOS pathway, score: -23.966, ranking: 5, group: 4

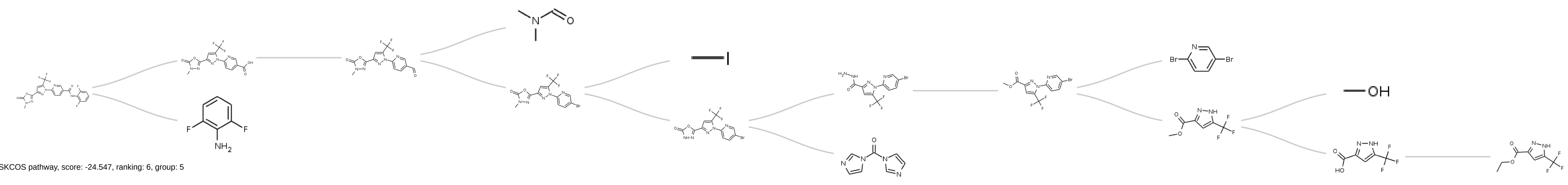

ASKCOS pathway, score: -24.547, ranking: 6, group: 5

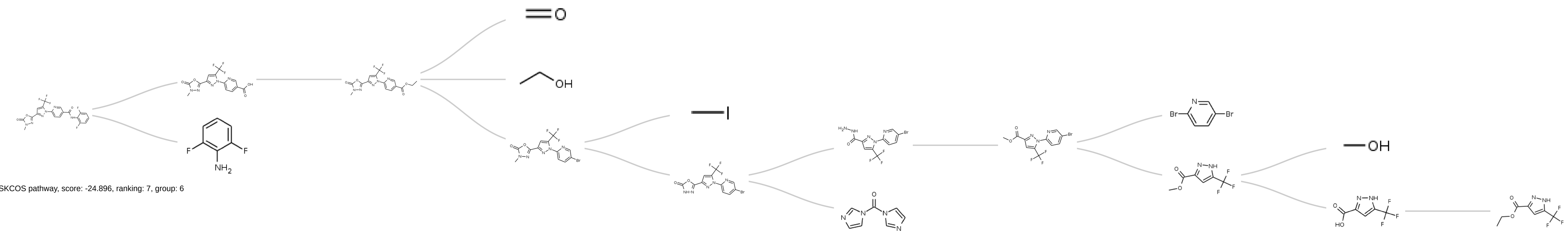

ASKCOS pathway, score: -24.896, ranking: 7, group: 6

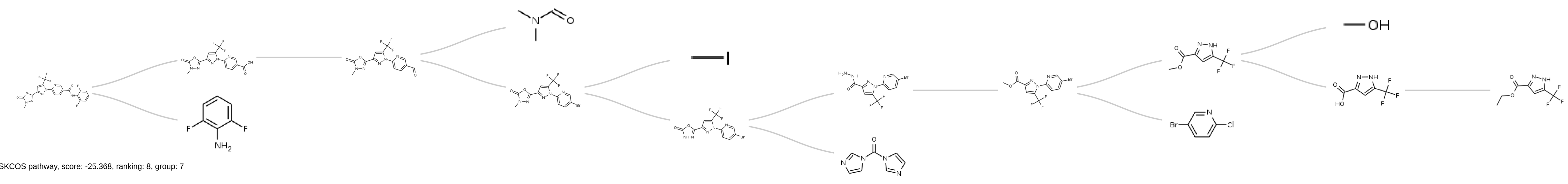

ASKCOS pathway, score: -25.368, ranking: 8, group: 7

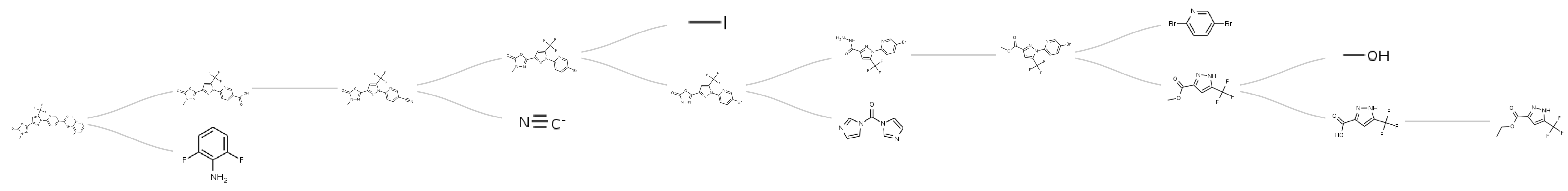

ASKCOS pathway, score: -25.376, ranking: 9, group: 8

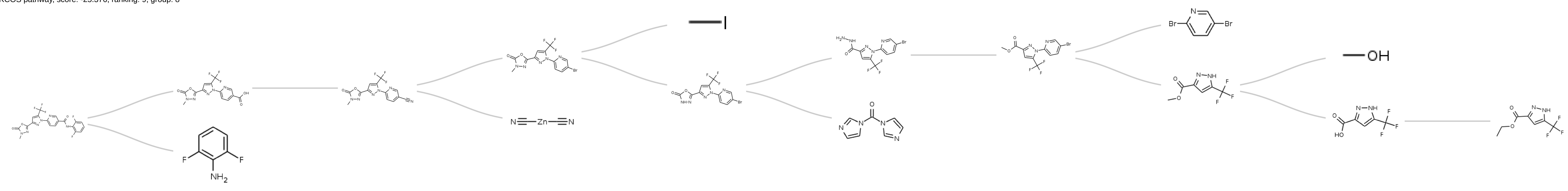

ASKCOS pathway, score: -25.378, ranking: 10, group: 9

Model ranks patent pathway as top-1: Example 18

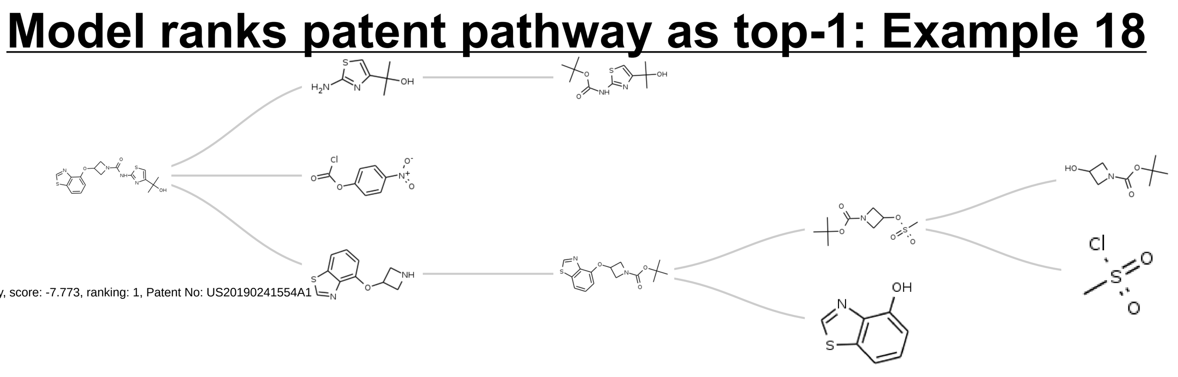

Piatachio pathway, score: -7.773, ranking: 1, Patent No: US20190241554A1

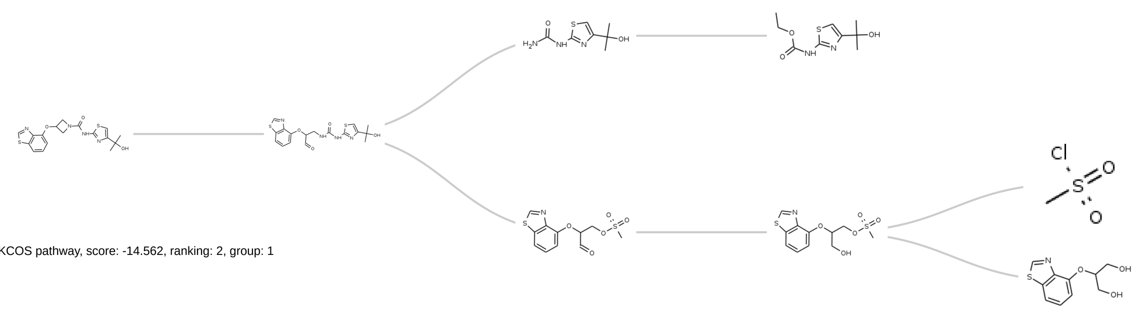

ASKCOS pathway, score: -14.562, ranking: 2, group: 1

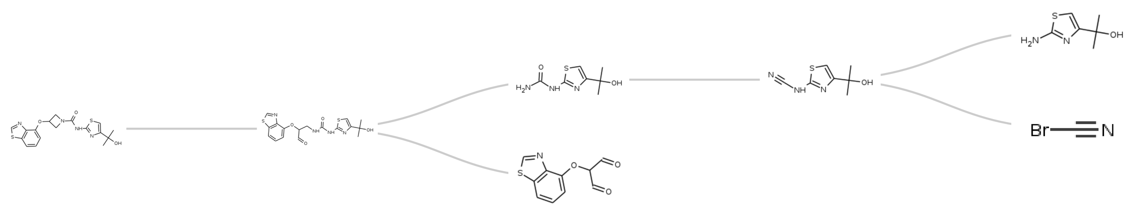

ASKCOS pathway, score: -17.652, ranking: 3, group: 2

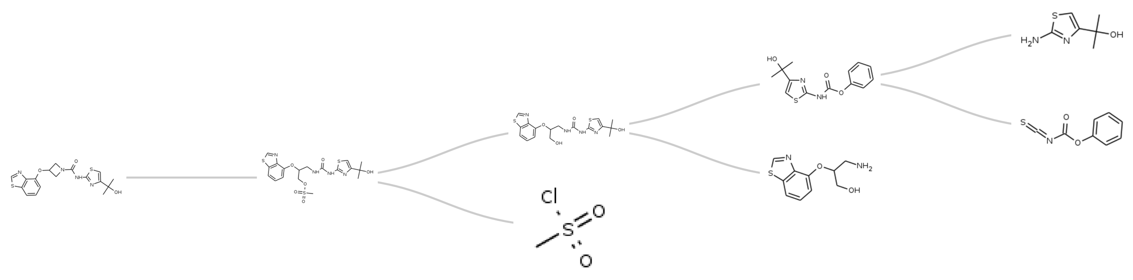

ASKCOS pathway, score: -18.016, ranking: 4, group: 3

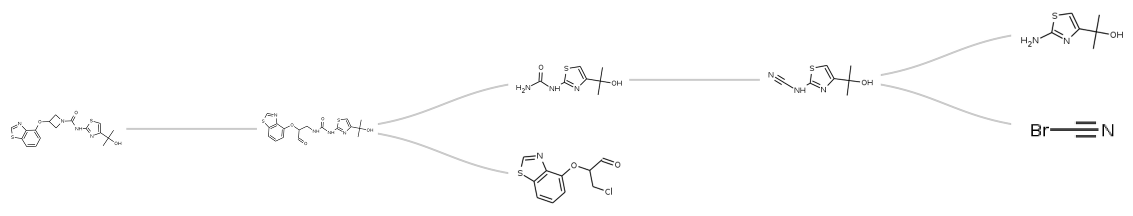

ASKCOS pathway, score: -18.040, ranking: 5, group: 4

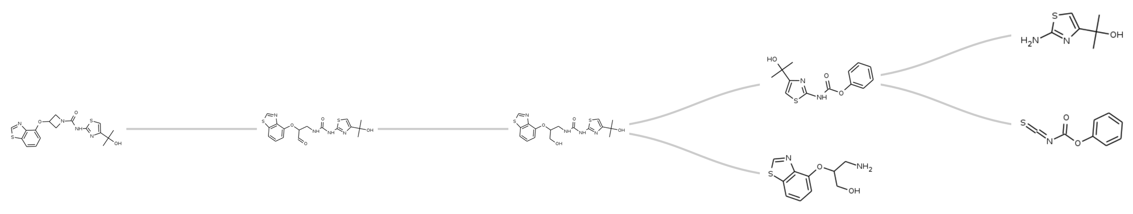

ASKCOS pathway, score: -18.143, ranking: 6, group: 5

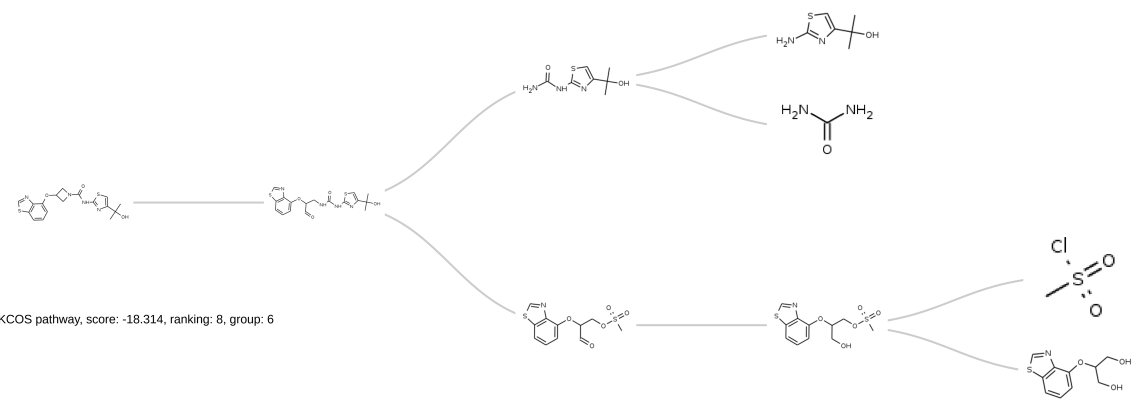

ASKCOS pathway, score: -18.314, ranking: 8, group: 6

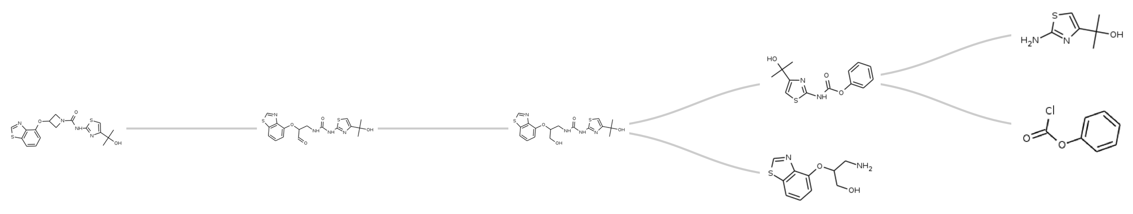

ASKCOS pathway, score: -18.543, ranking: 13, group: 7

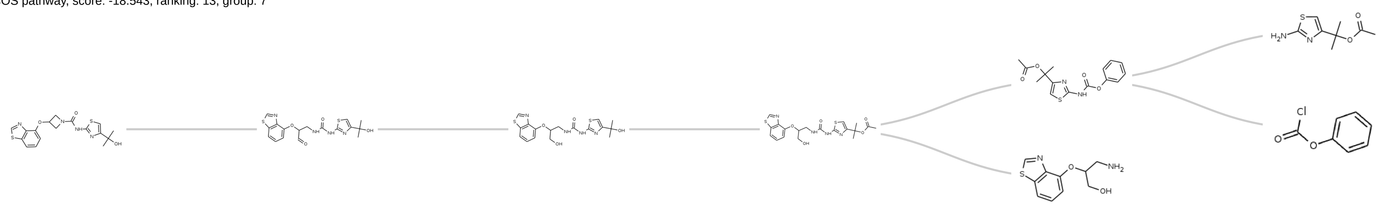

ASKCOS pathway, score: -18.584, ranking: 14, group: 8

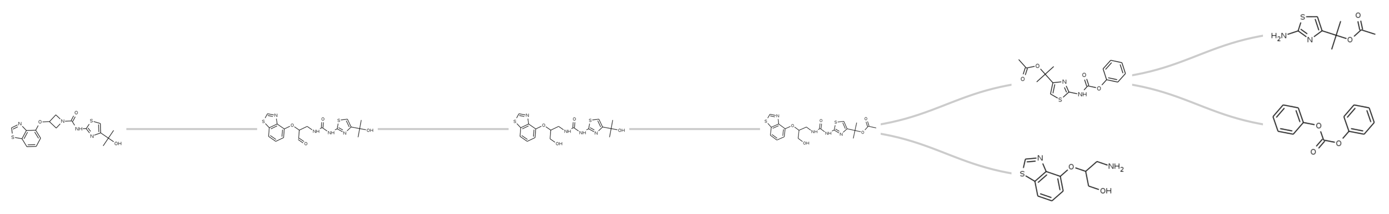

ASKCOS pathway, score: -18.696, ranking: 18, group: 9

Model ranks patent pathway as top-1: Example 19

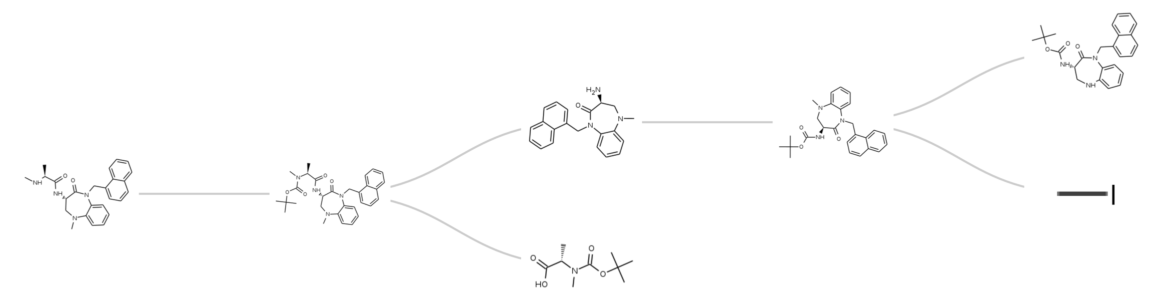

Piatachio pathway, score: -12.956, ranking: 1, Patent No: US20160272596A1

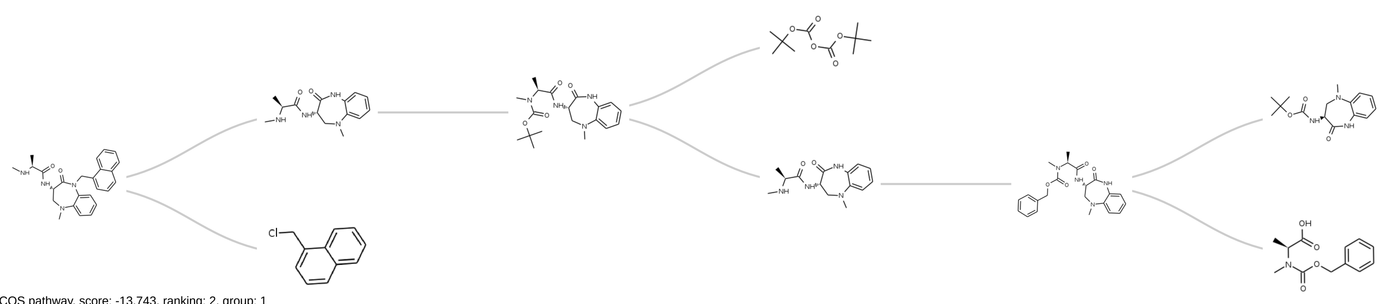

ASKCOS pathway, score: -13.743, ranking: 2, group: 1

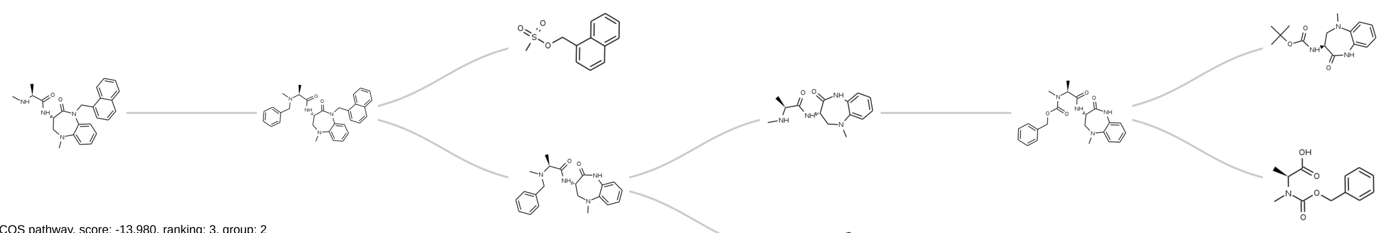

ASKCOS pathway, score: -13.980, ranking: 3, group: 2

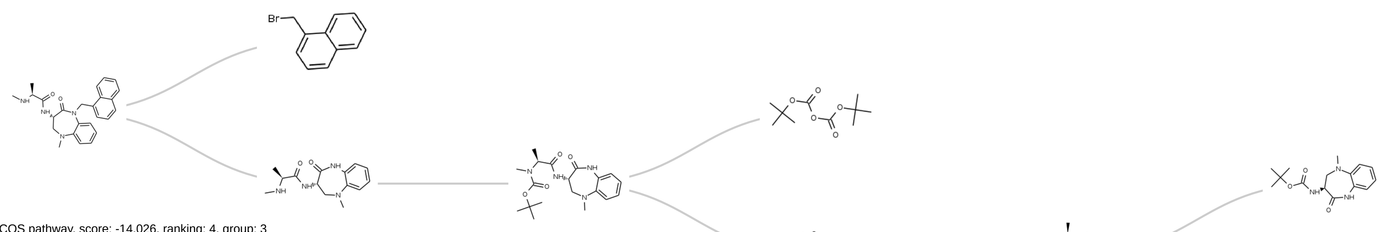

ASKCOS pathway, score: -14.026, ranking: 4, group: 3

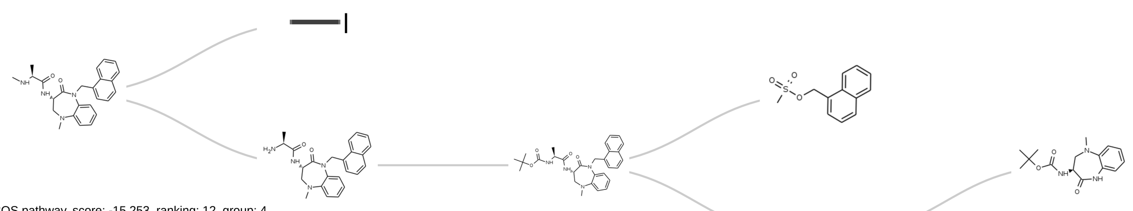

ASKCOS pathway, score: -15.253, ranking: 12, group: 4

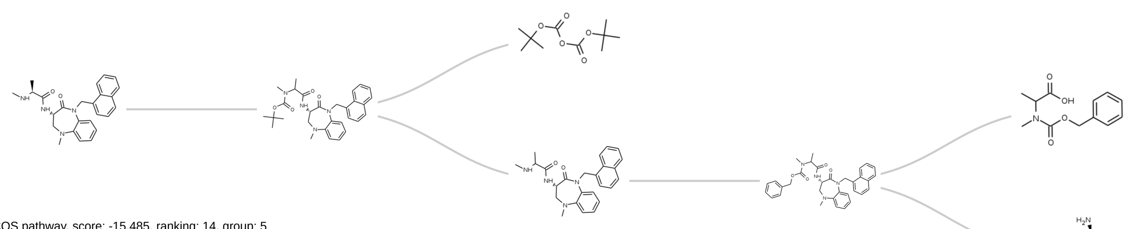

ASKCOS pathway, score: -15.485, ranking: 14, group: 5

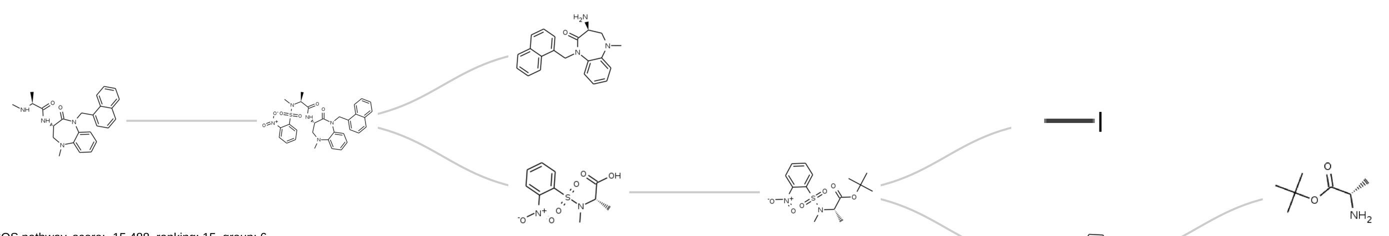

ASKCOS pathway, score: -15.488, ranking: 15, group: 6

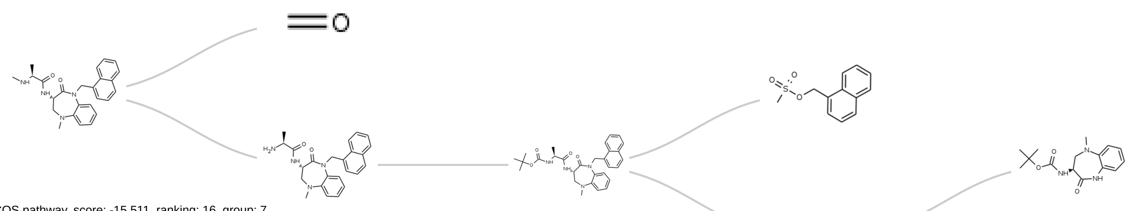

ASKCOS pathway, score: -15.511, ranking: 16, group: 7

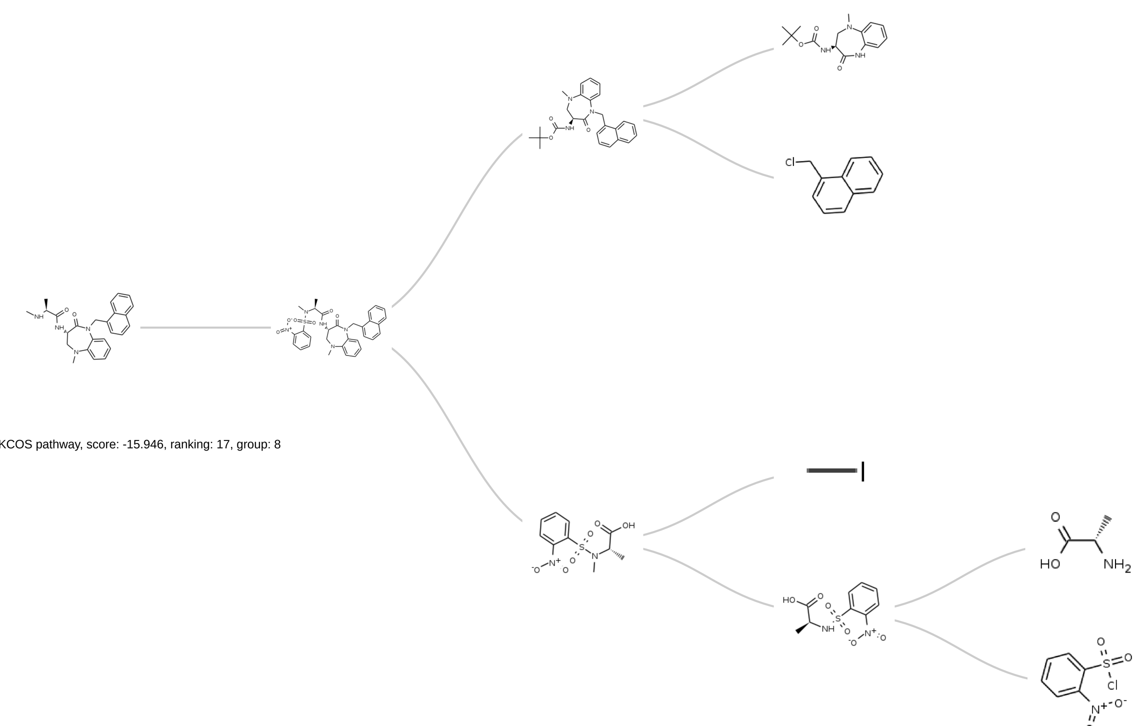

ASKCOS pathway, score: -15.946, ranking: 17, group: 8

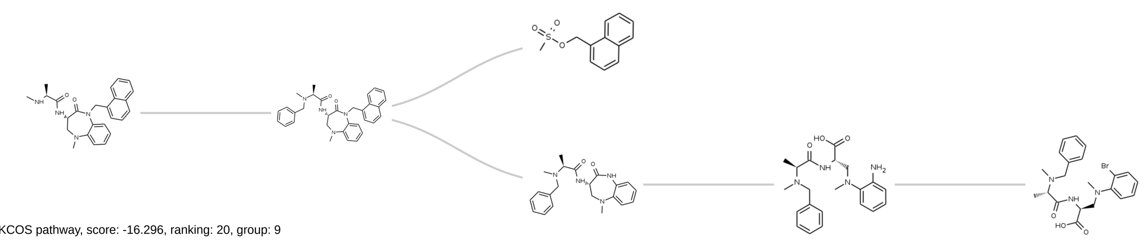

ASKCOS pathway, score: -16.296, ranking: 20, group: 9

Model ranks patent pathway as top-1: Example 20

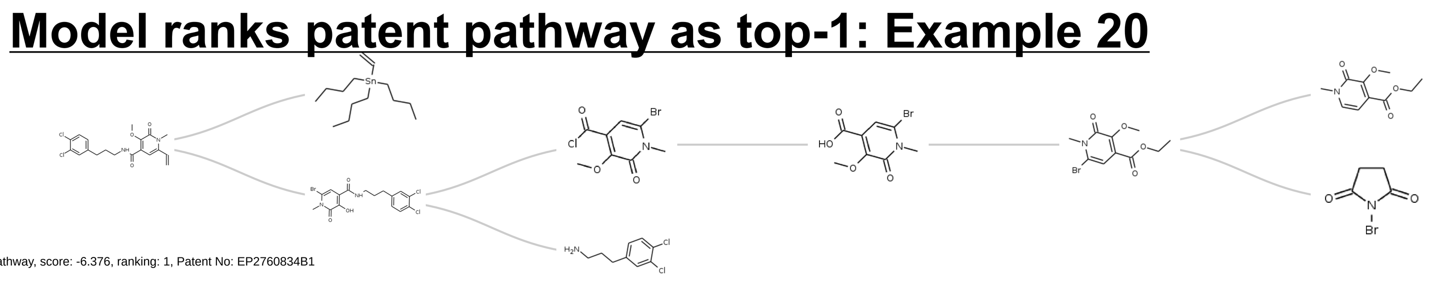

Piatachio pathway, score: -6.376, ranking: 1, Patent No: EP2760834B1

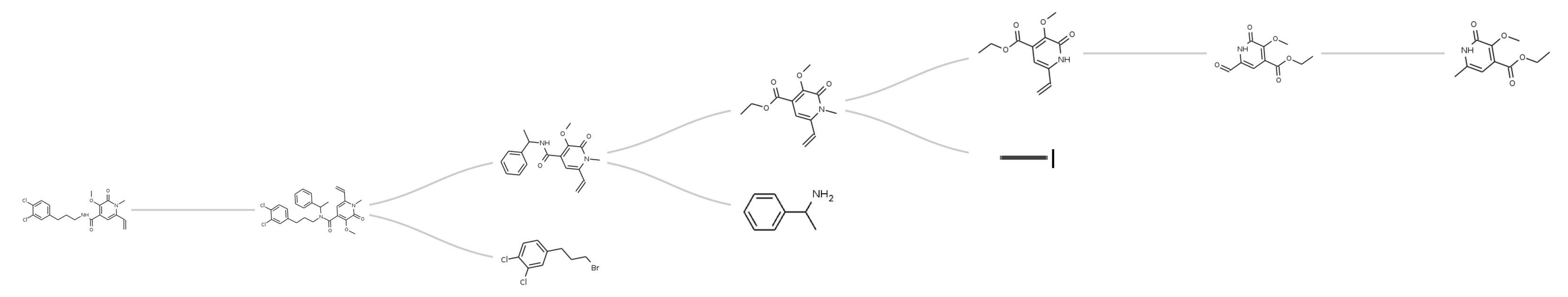

ASKCOS pathway, score: -15.831, ranking: 2, group: 1

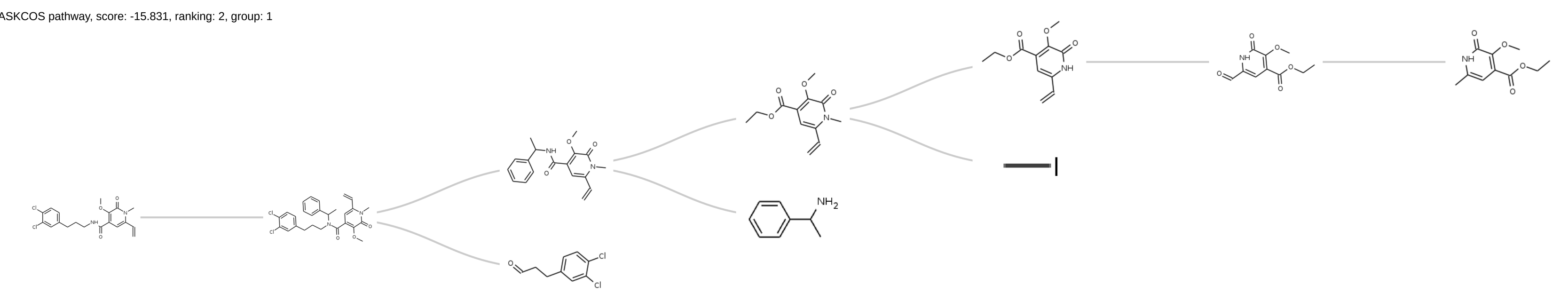

ASKCOS pathway, score: -15.842, ranking: 3, group: 2

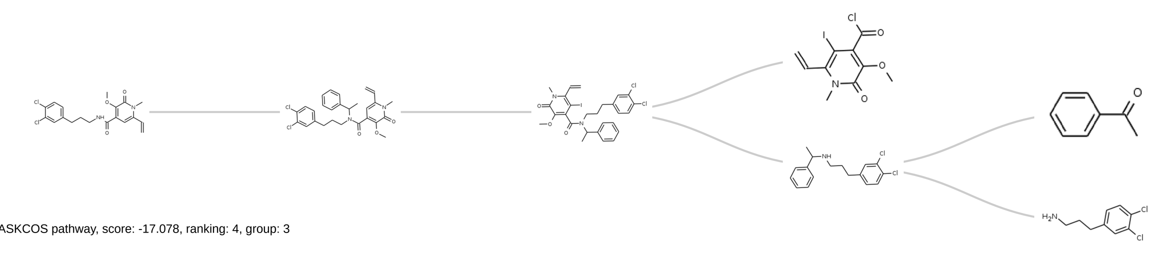

ASKCOS pathway, score: -17.078, ranking: 4, group: 3

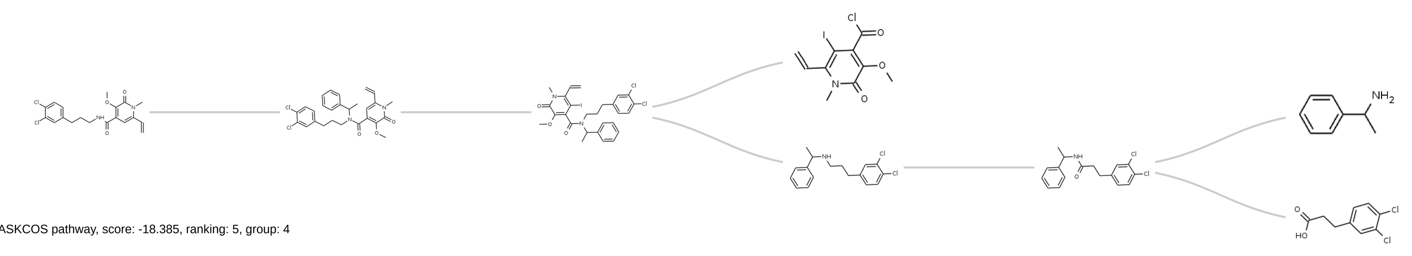

ASKCOS pathway, score: -18.385, ranking: 5, group: 4

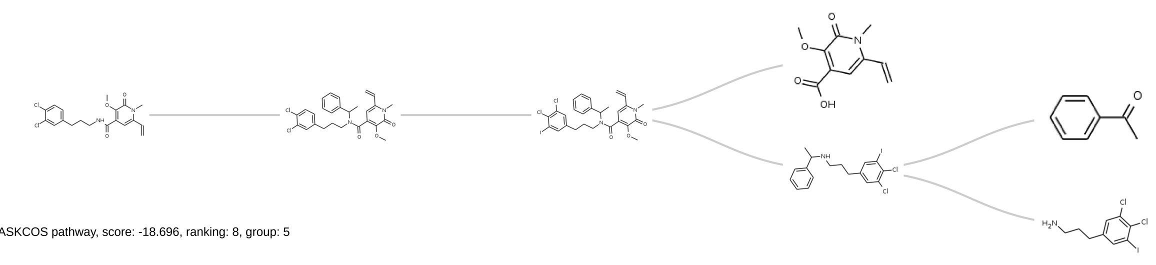

ASKCOS pathway, score: -18.696, ranking: 8, group: 5

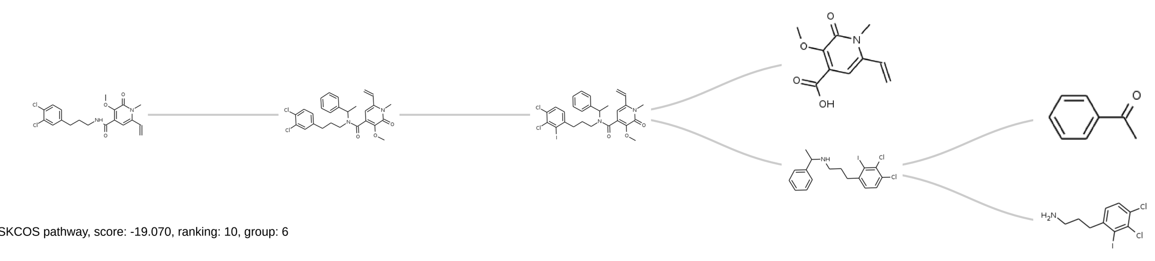

ASKCOS pathway, score: -19.070, ranking: 10, group: 6

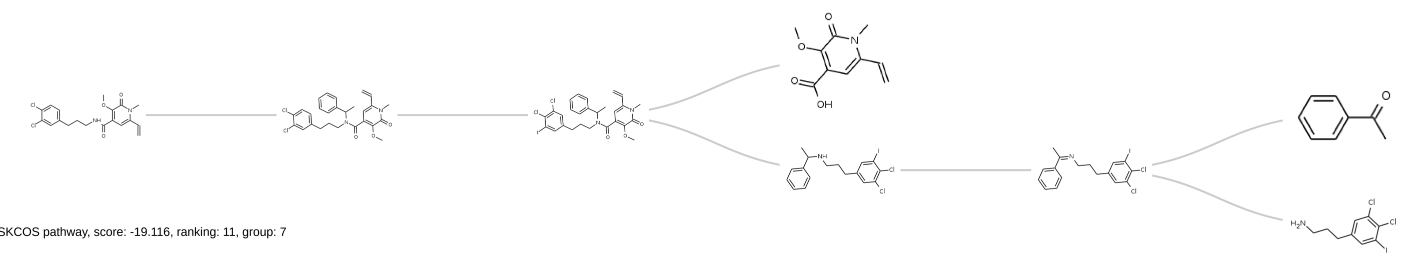

ASKCOS pathway, score: -19.116, ranking: 11, group: 7

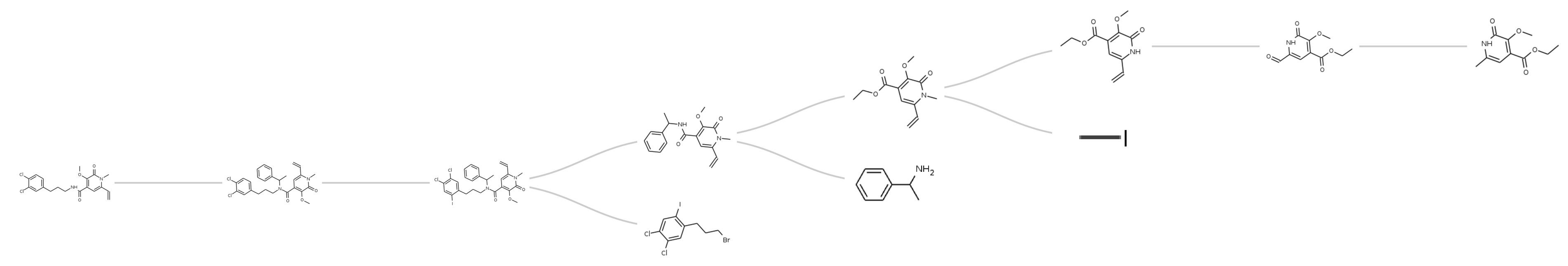

ASKCOS pathway, score: -19.286, ranking: 12, group: 8

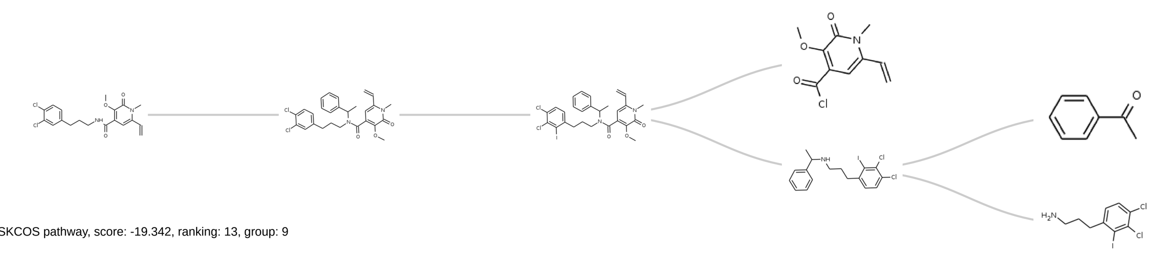

ASKCOS pathway, score: -19.342, ranking: 13, group: 9

Model ranks patent pathway as top-1: Example 21

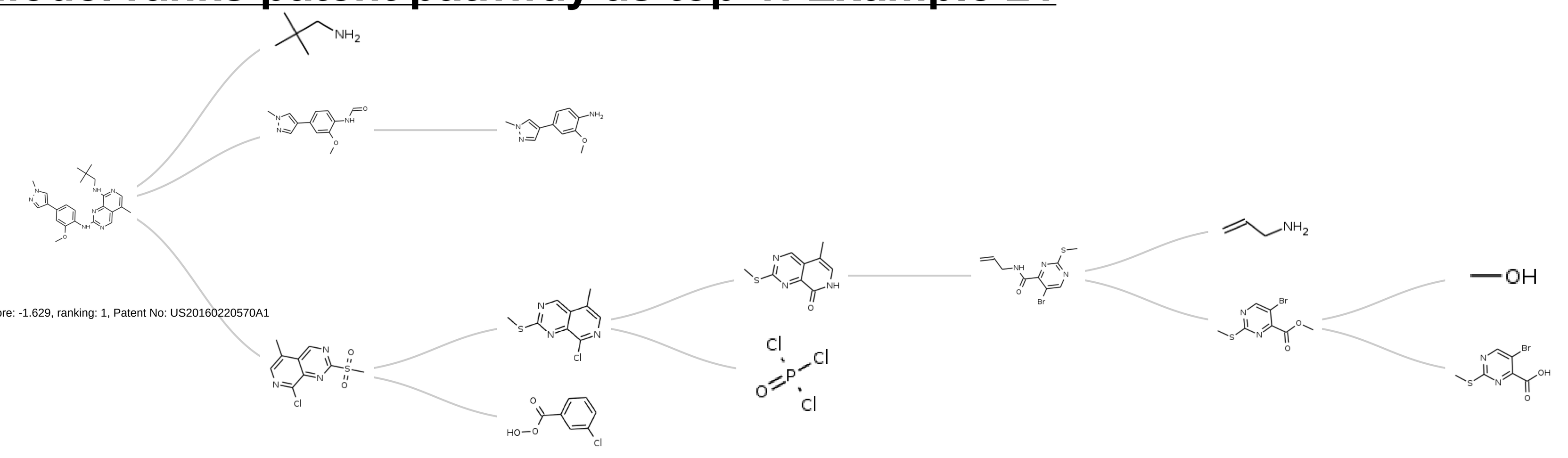

Piatachio pathway, score: -1.629, ranking: 1, Patent No: US20160220570A1

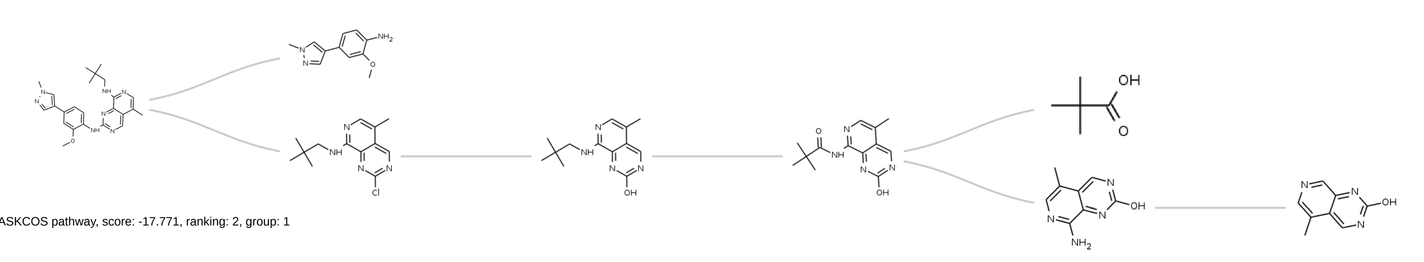

ASKCOS pathway, score: -17.771, ranking: 2, group: 1

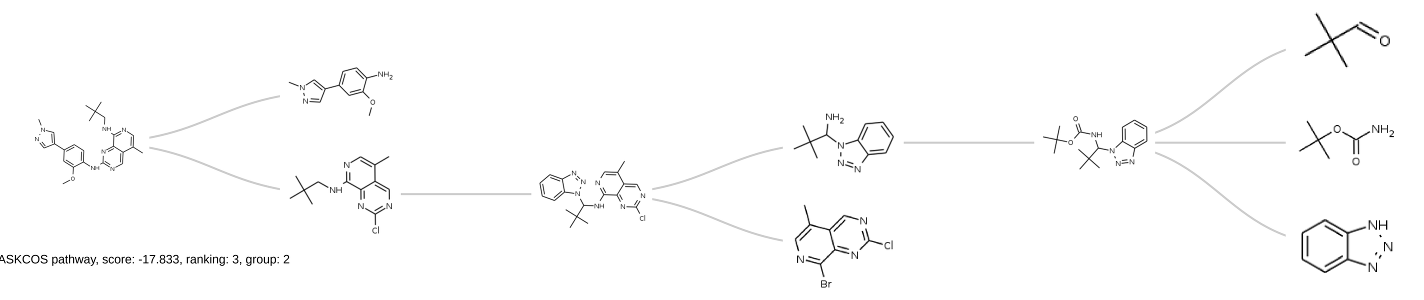

ASKCOS pathway, score: -17.833, ranking: 3, group: 2

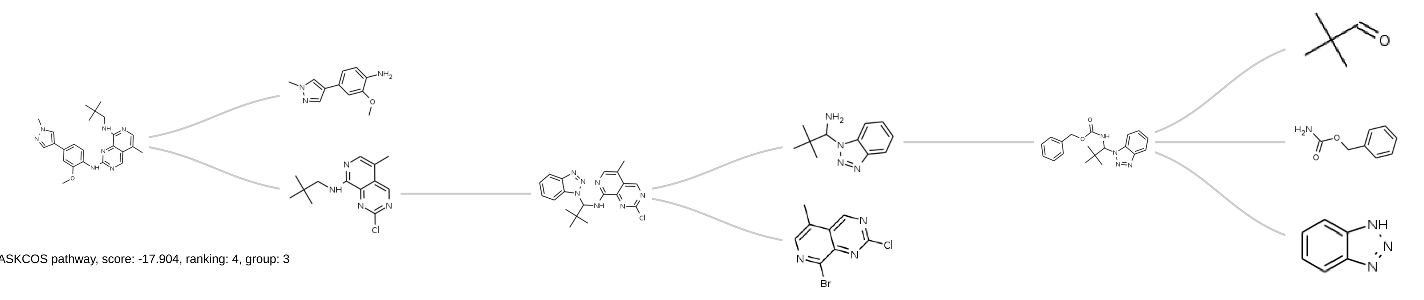

ASKCOS pathway, score: -17.904, ranking: 4, group: 3

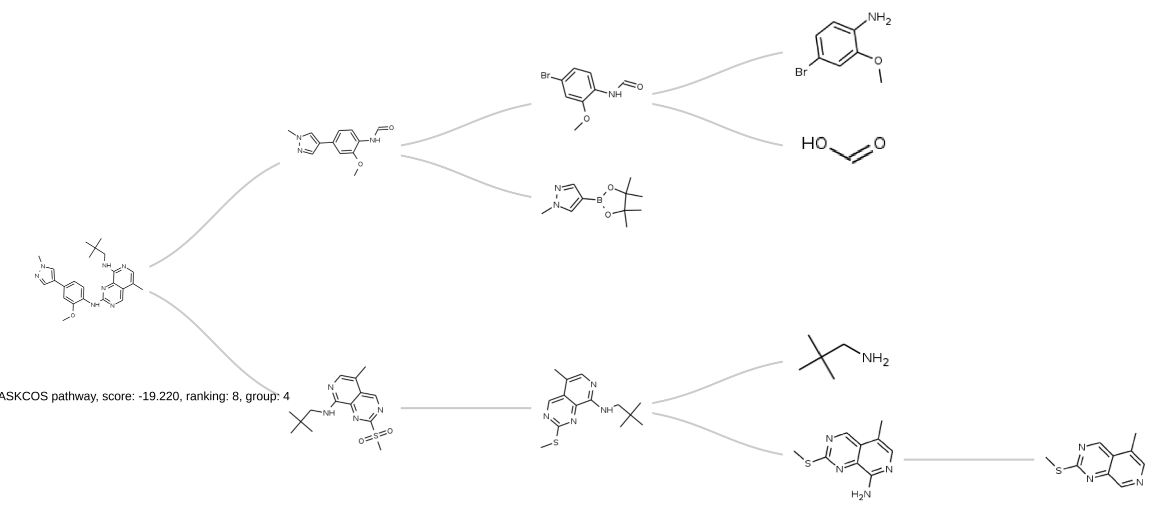

ASKCOS pathway, score: -19.220, ranking: 8, group: 4

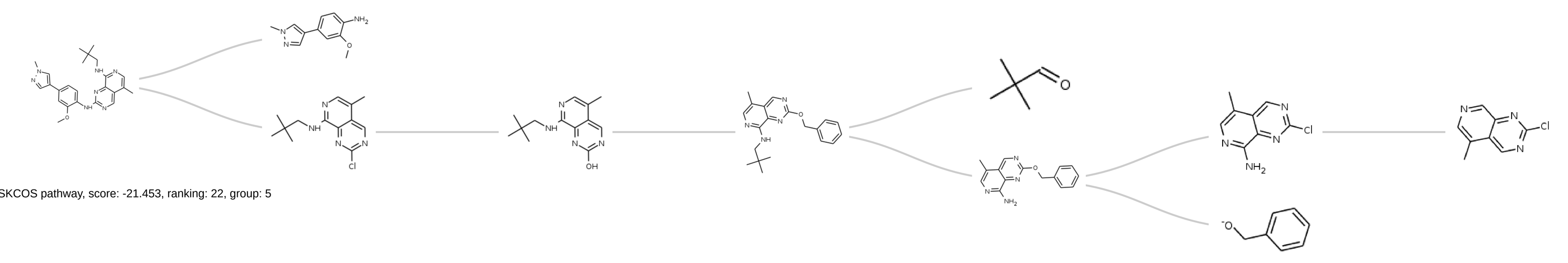

ASKCOS pathway, score: -21.453, ranking: 22, group: 5

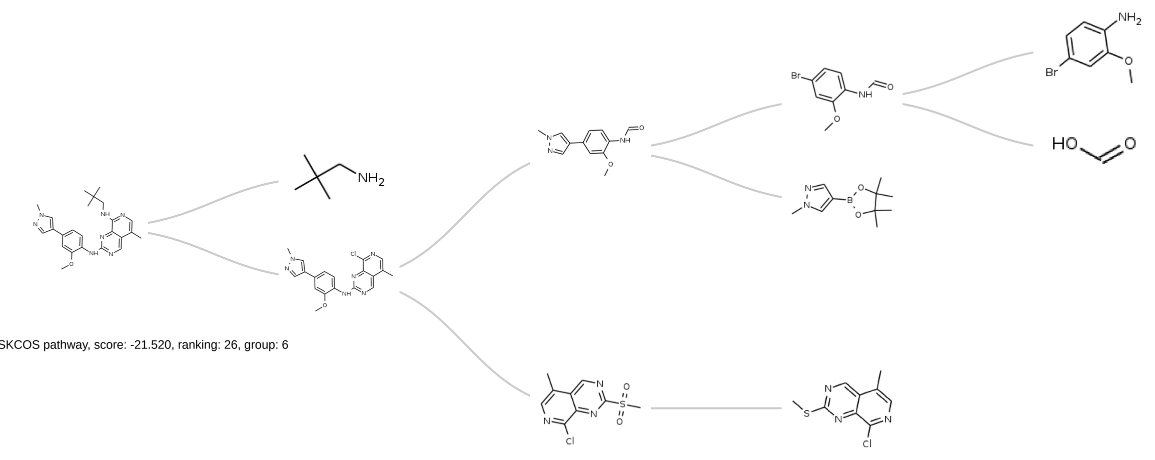

ASKCOS pathway, score: -21.520, ranking: 26, group: 6

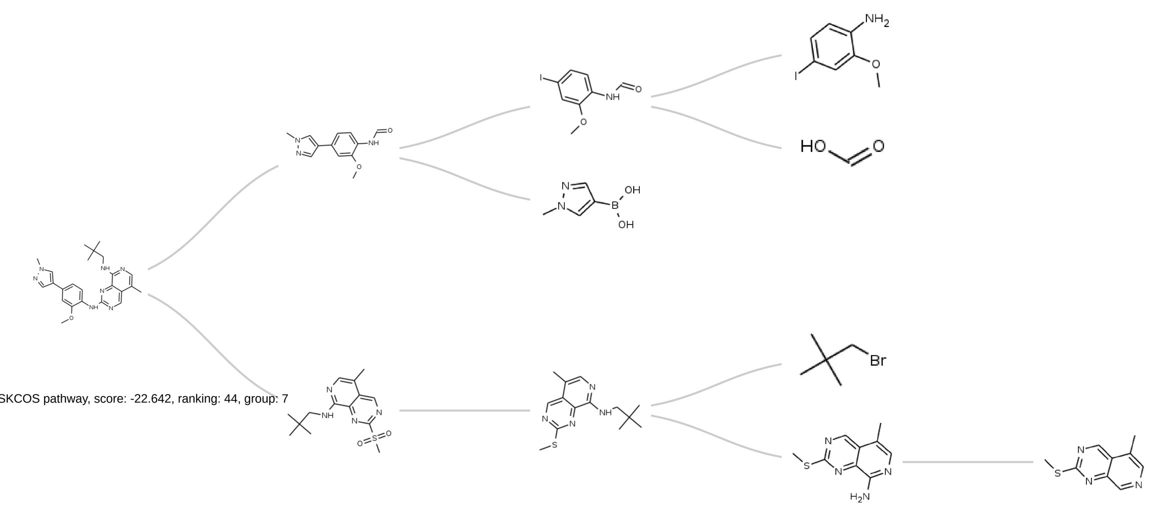

ASKCOS pathway, score: -22.642, ranking: 44, group: 7

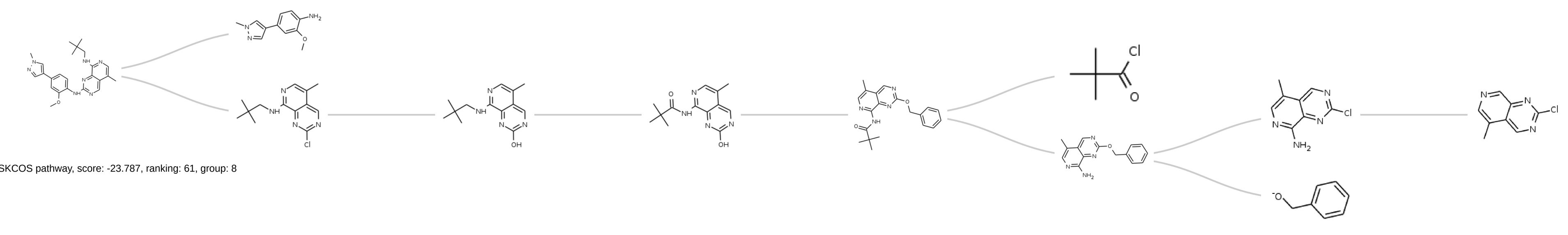

ASKCOS pathway, score: -23.787, ranking: 61, group: 8

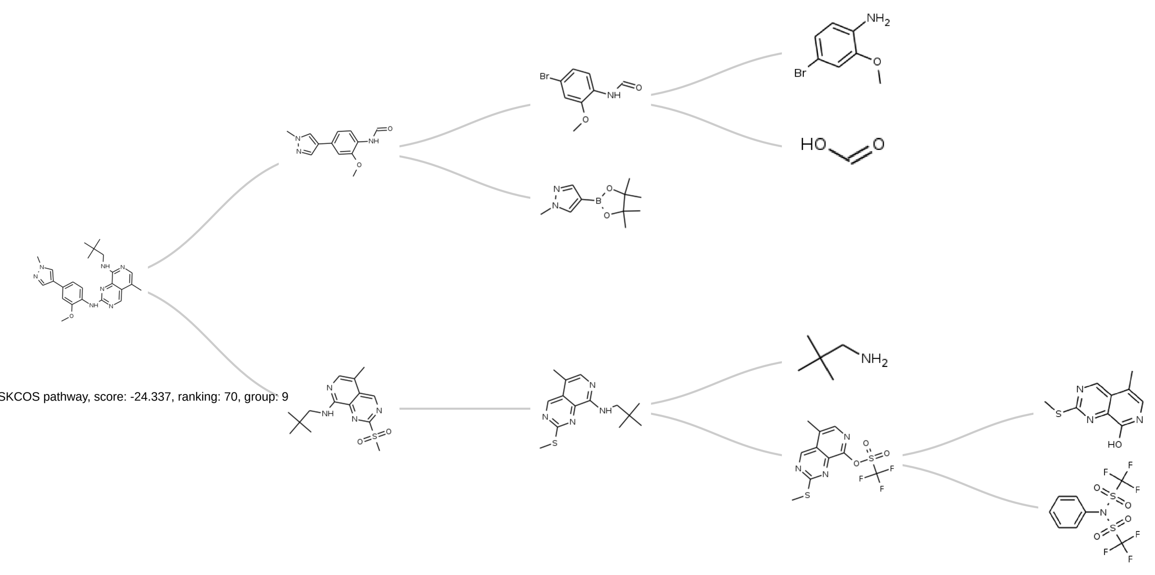

ASKCOS pathway, score: -24.337, ranking: 70, group: 9

Model ranks patent pathway as top-1: Example 22

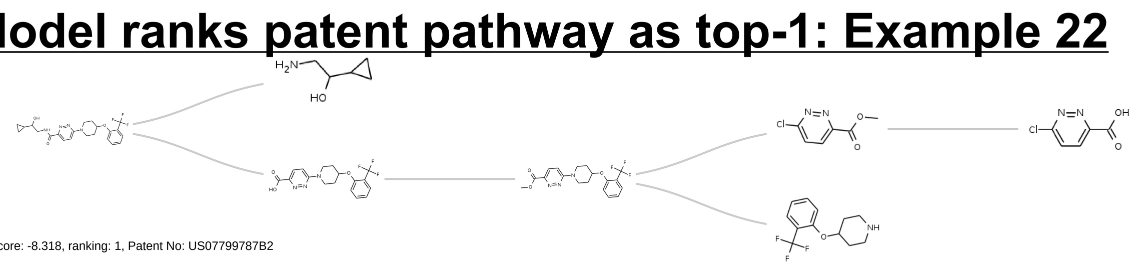

Piatachio pathway, score: -8.318, ranking: 1, Patent No: US07799787B2

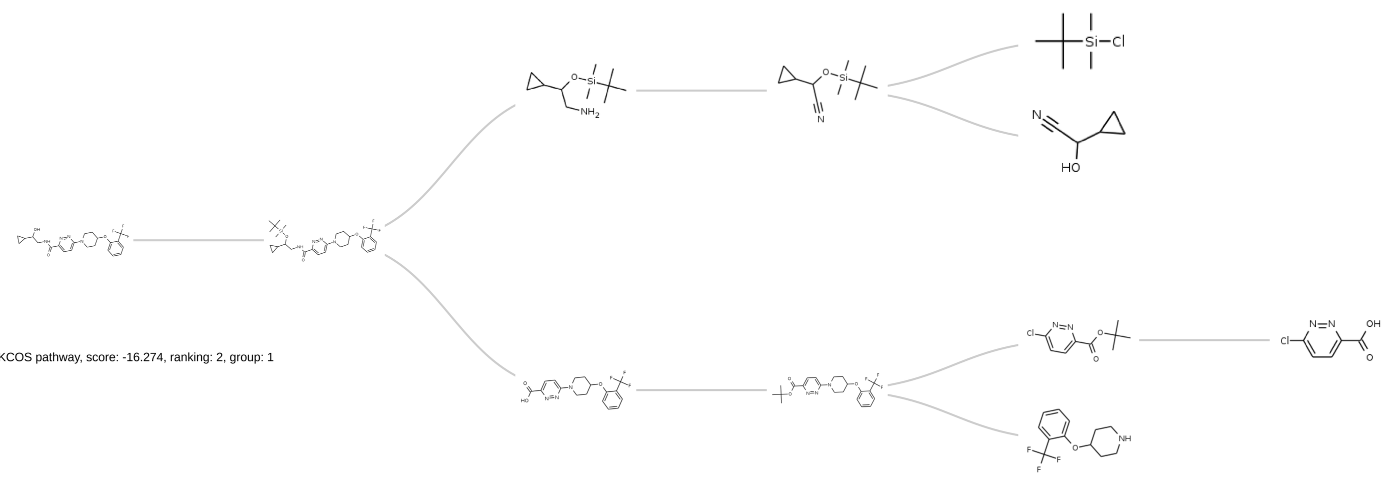

ASKCOS pathway, score: -16.274, ranking: 2, group: 1

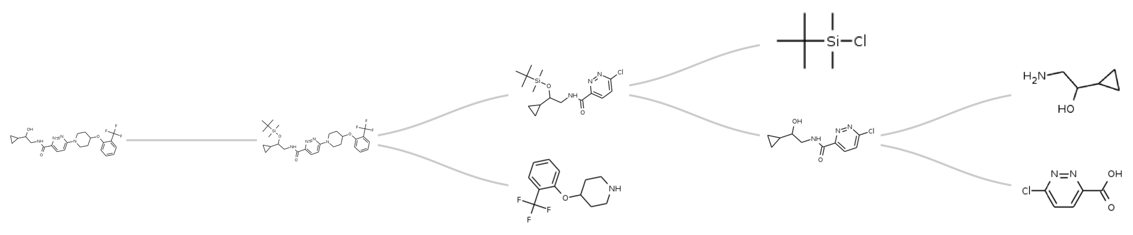

ASKCOS pathway, score: -16.790, ranking: 3, group: 2

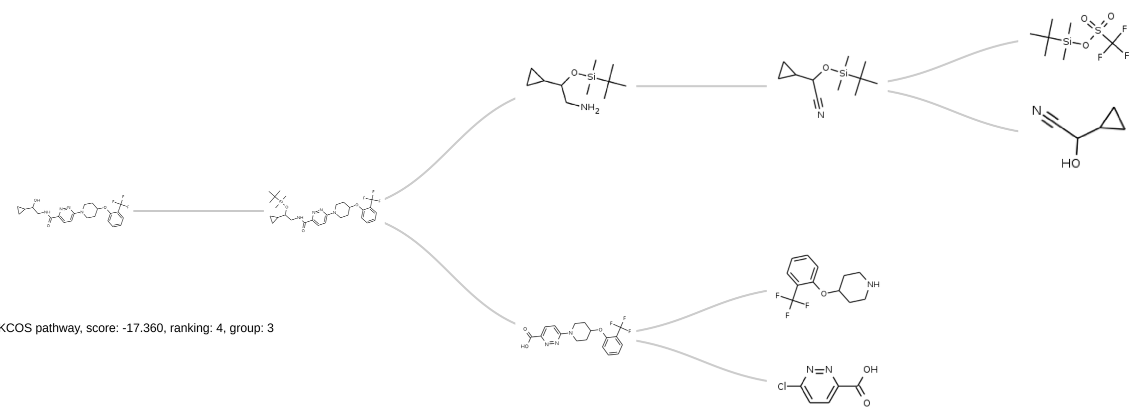

ASKCOS pathway, score: -17.360, ranking: 4, group: 3

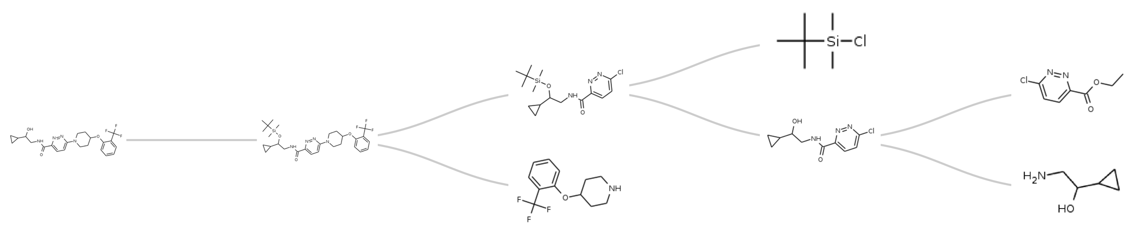

ASKCOS pathway, score: -18.005, ranking: 5, group: 4

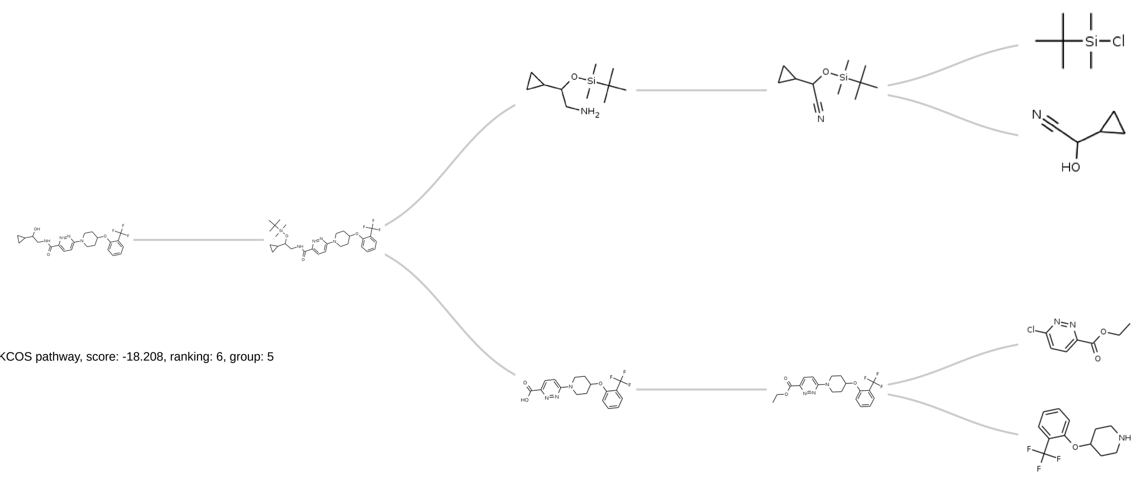

ASKCOS pathway, score: -18.208, ranking: 6, group: 5

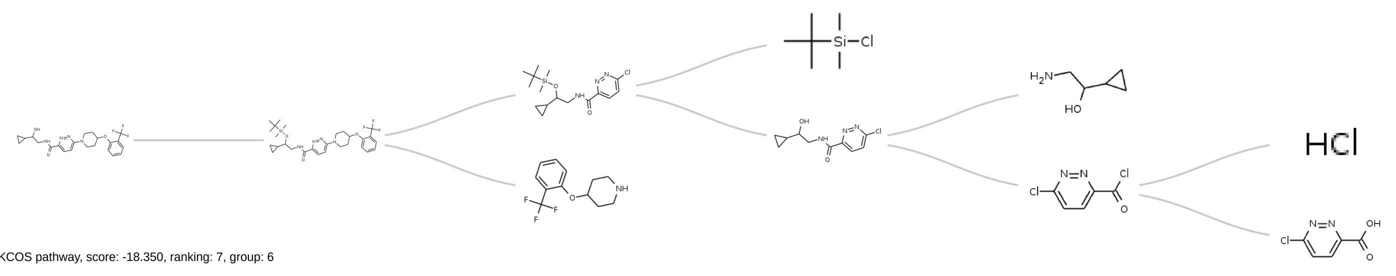

ASKCOS pathway, score: -18.350, ranking: 7, group: 6

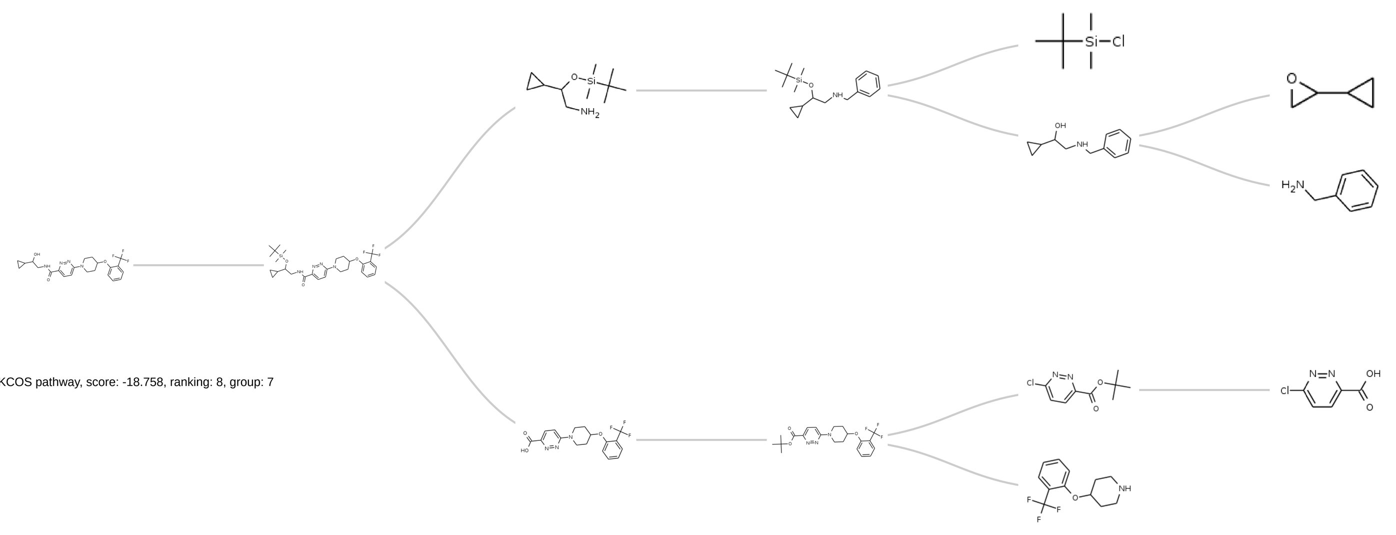

ASKCOS pathway, score: -18.758, ranking: 8, group: 7

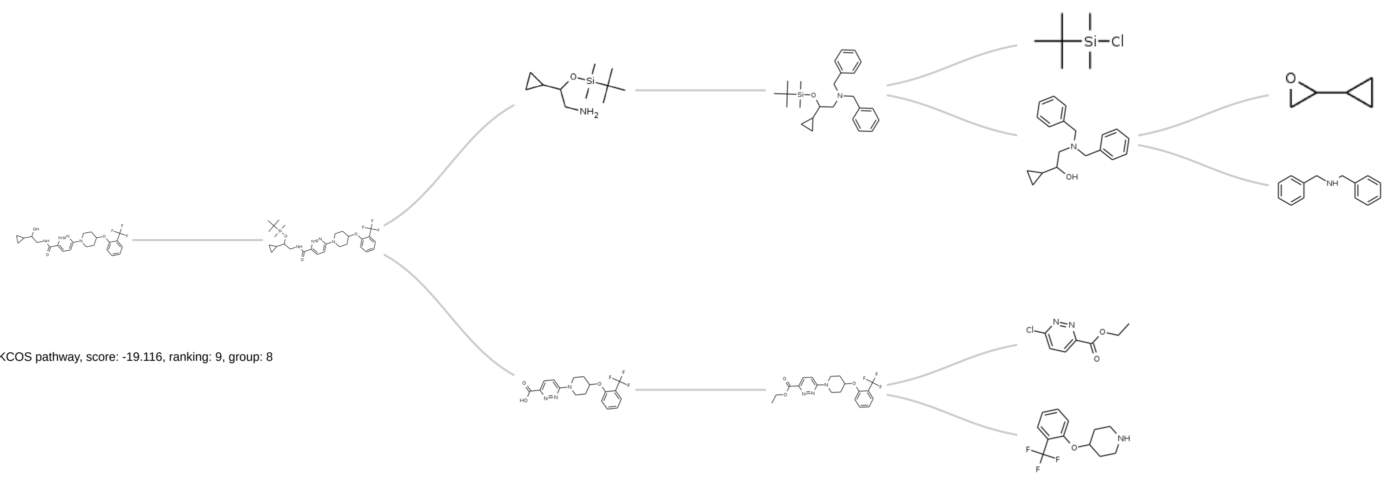

ASKCOS pathway, score: -19.116, ranking: 9, group: 8

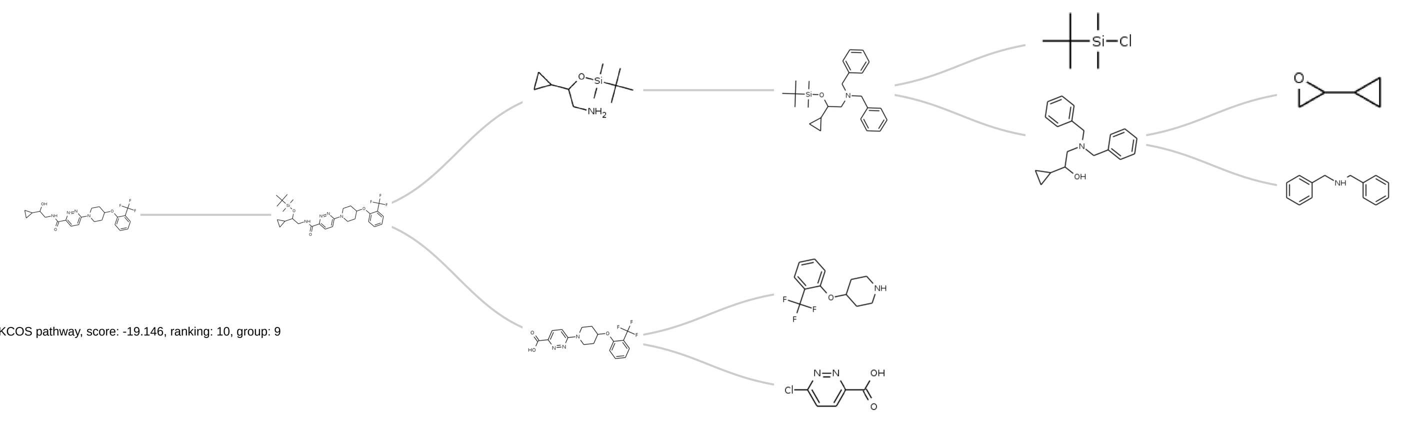

ASKCOS pathway, score: -19.146, ranking: 10, group: 9

Model ranks patent pathway as top-1: Example 23

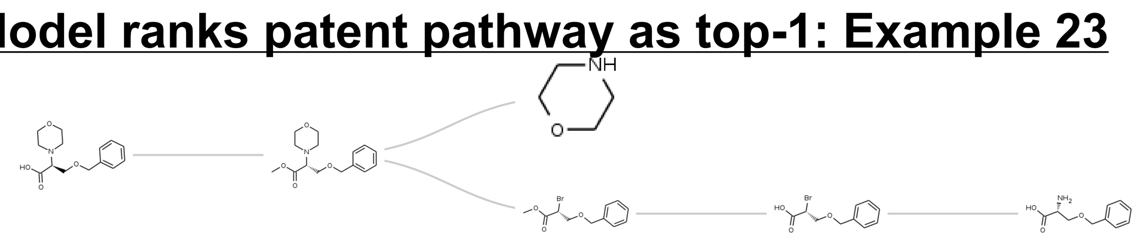

Piatachio pathway, score: -8.714, ranking: 1, Patent No: US20180037557A1

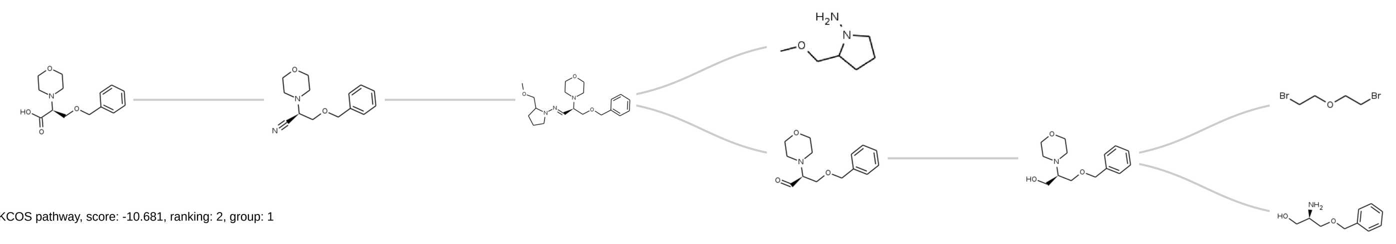

ASKCOS pathway, score: -10.681, ranking: 2, group: 1

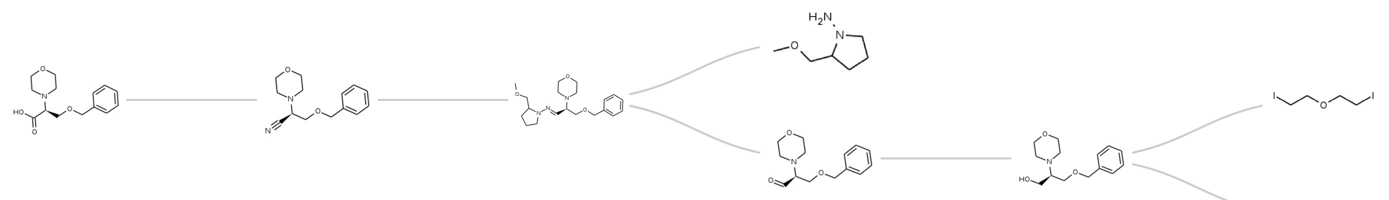

ASKCOS pathway, score: -11.230, ranking: 3, group: 2

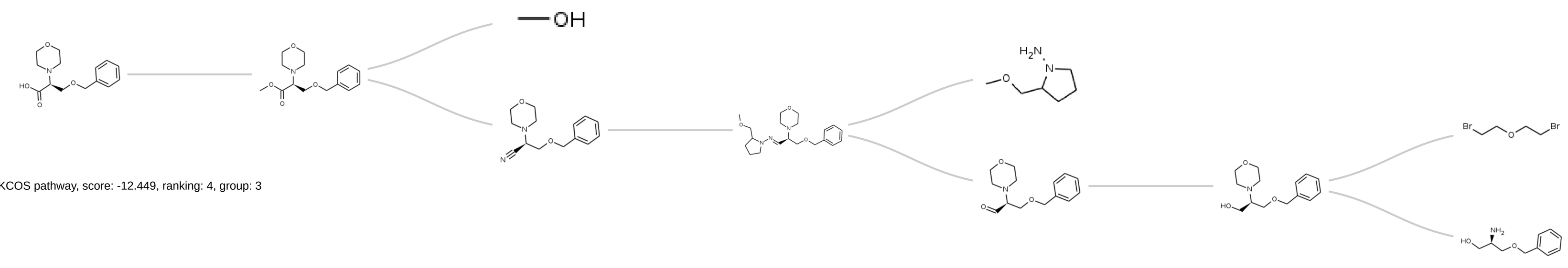

ASKCOS pathway, score: -12.449, ranking: 4, group: 3

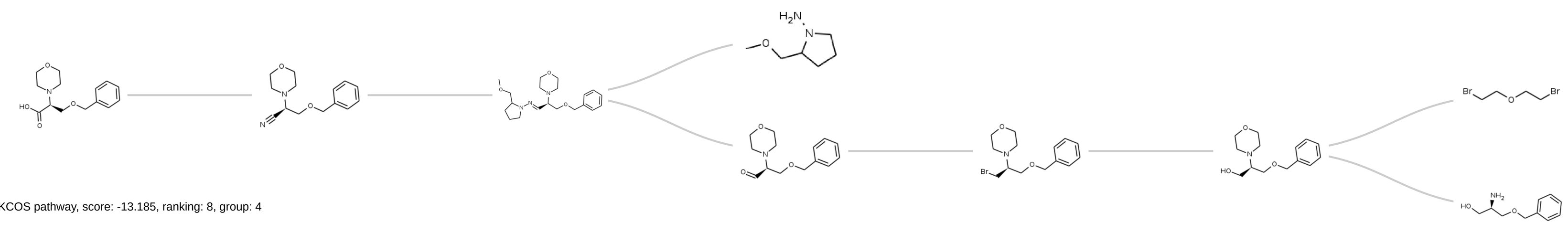

ASKCOS pathway, score: -13.185, ranking: 8, group: 4

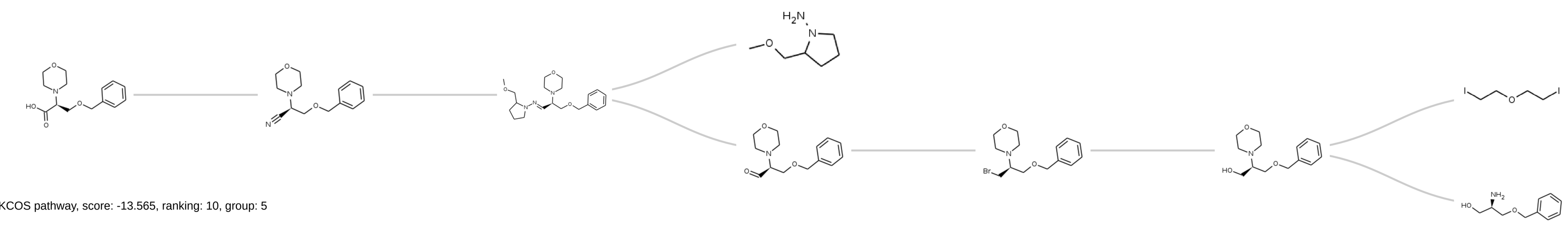

ASKCOS pathway, score: -13.565, ranking: 10, group: 5

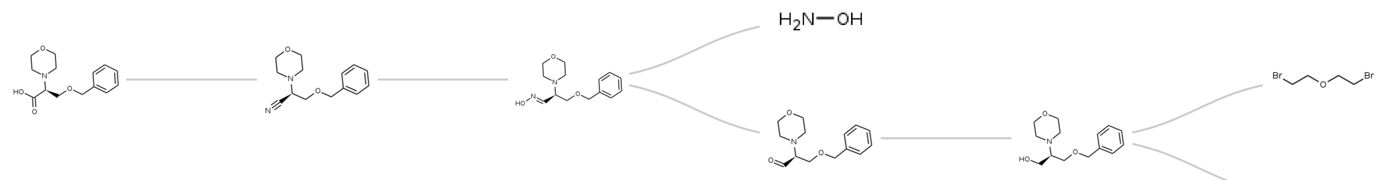

ASKCOS pathway, score: -14.068, ranking: 12, group: 6

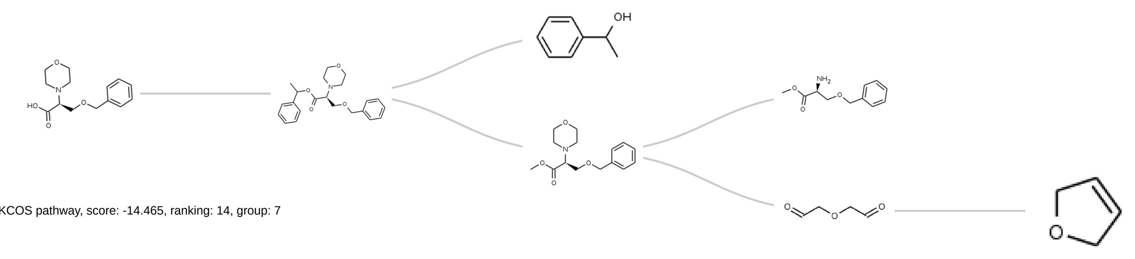

ASKCOS pathway, score: -14.465, ranking: 14, group: 7

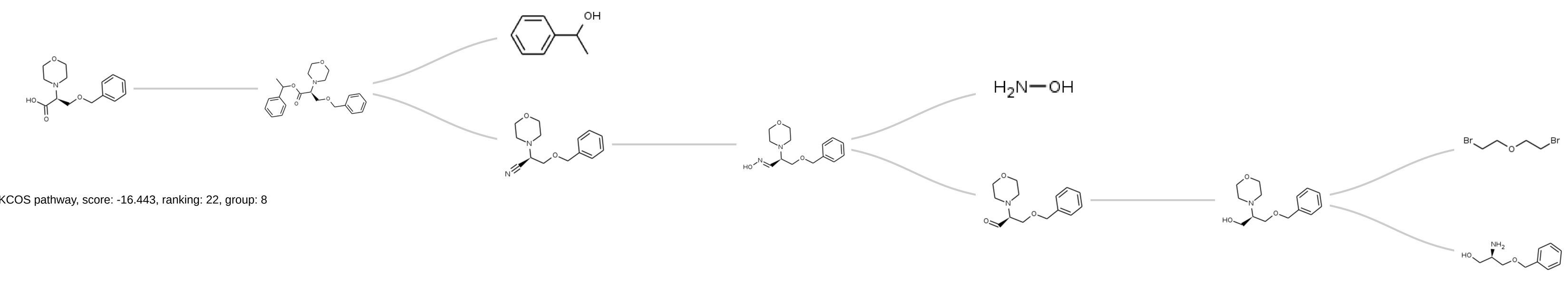

ASKCOS pathway, score: -16.443, ranking: 22, group: 8

Model ranks patent pathway as top-1: Example 24

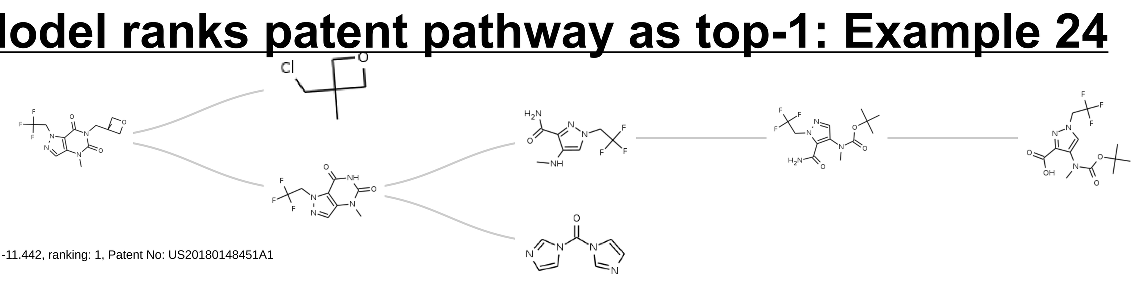

Piatachio pathway, score: -11.442, ranking: 1, Patent No: US20180148451A1

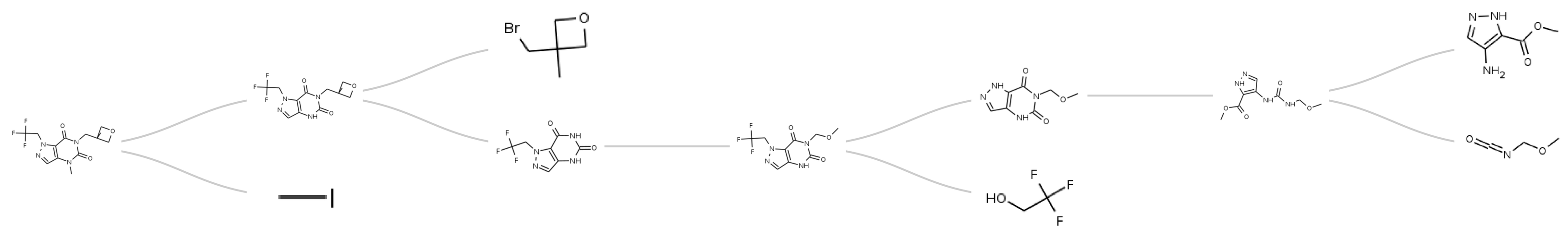

ASKCOS pathway, score: -16.806, ranking: 2, group: 1

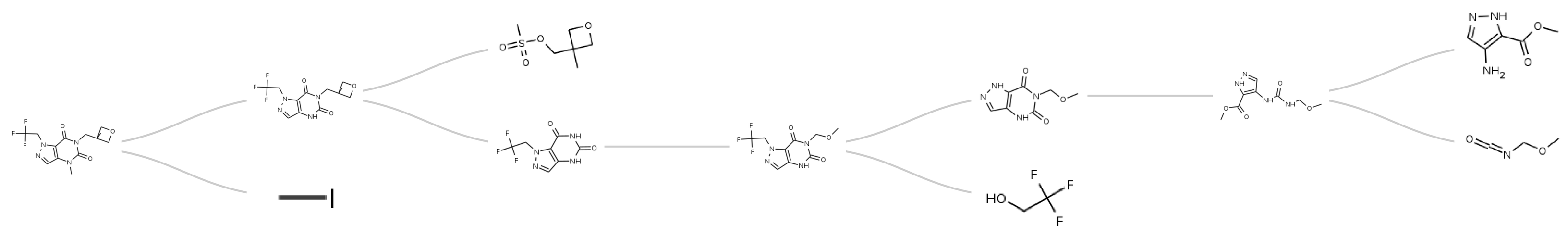

ASKCOS pathway, score: -17.307, ranking: 3, group: 2

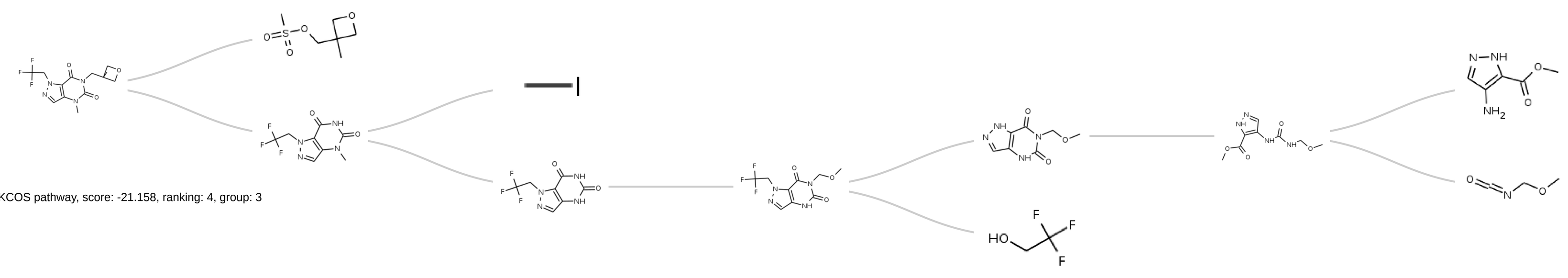

ASKCOS pathway, score: -21.158, ranking: 4, group: 3

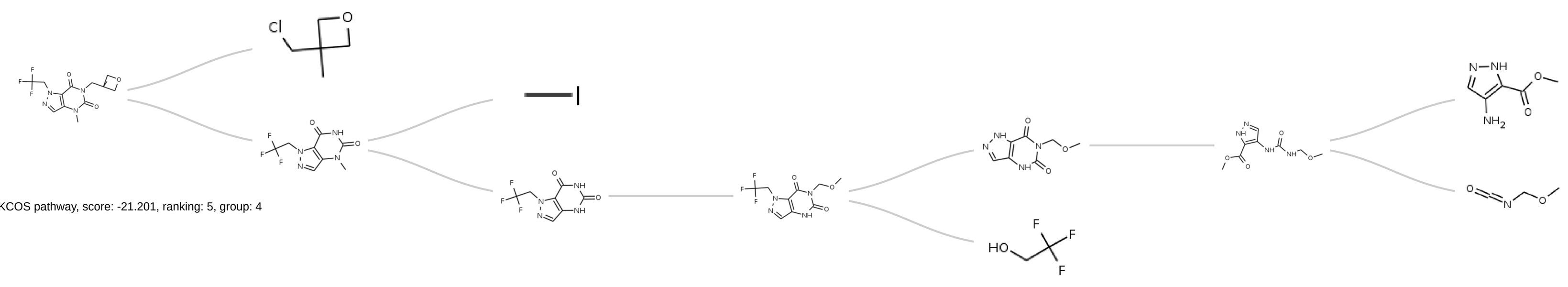

ASKCOS pathway, score: -21.201, ranking: 5, group: 4

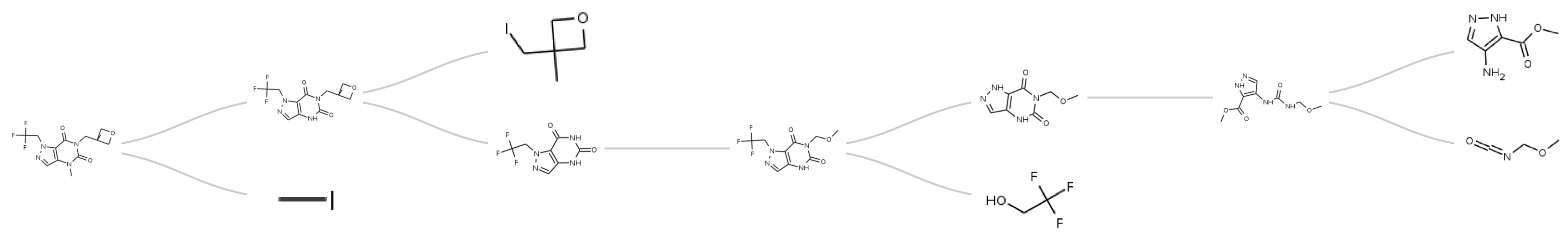

ASKCOS pathway, score: -21.570, ranking: 6, group: 5

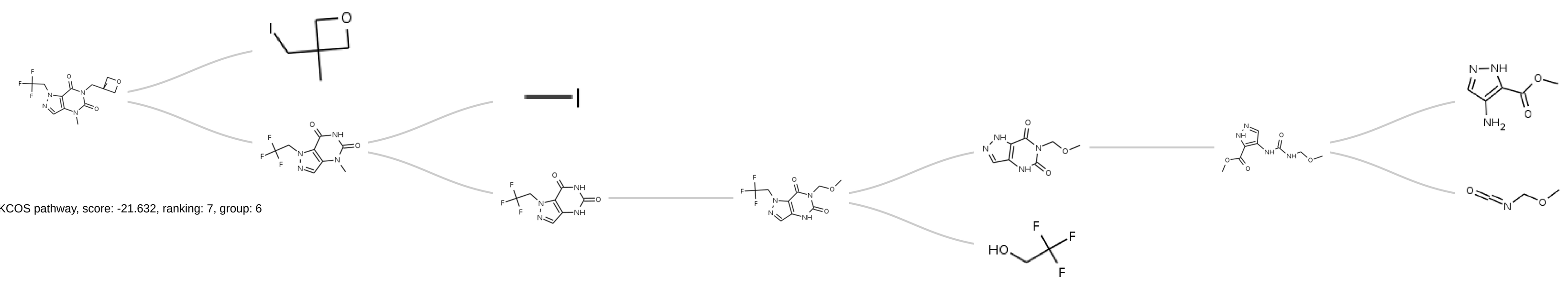

ASKCOS pathway, score: -21.632, ranking: 7, group: 6

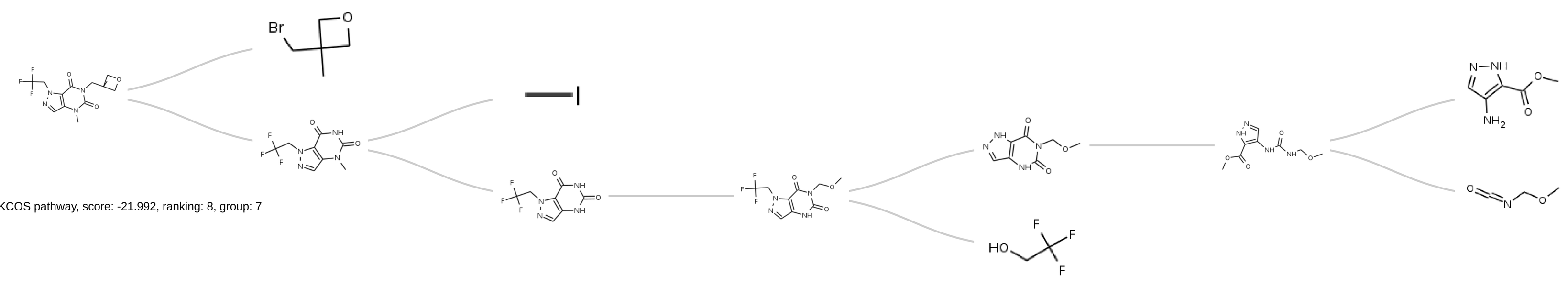

ASKCOS pathway, score: -21.992, ranking: 8, group: 7

Model ranks patent pathway as top-1: Example 25

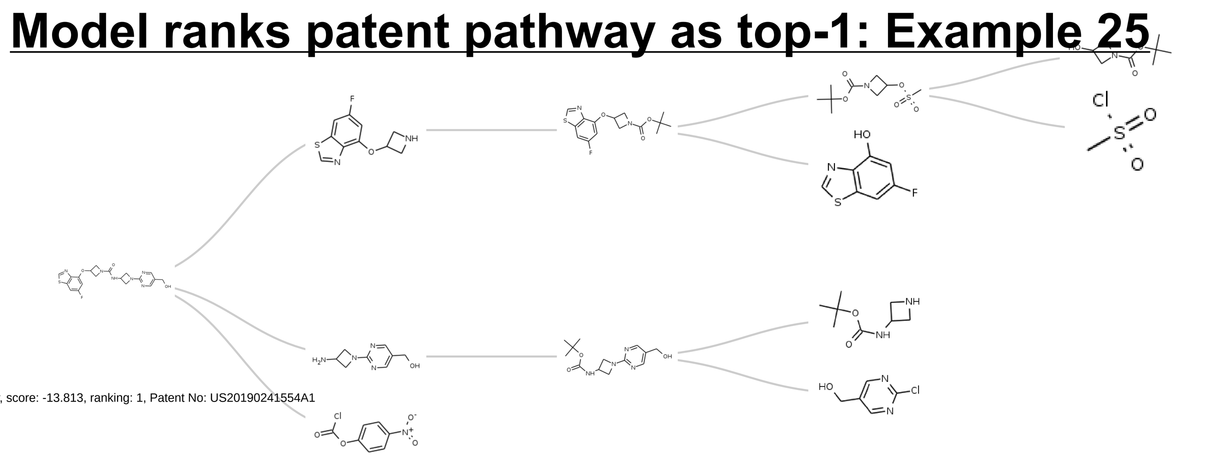

Piatachio pathway, score: -13.813, ranking: 1, Patent No: US20190241554A1

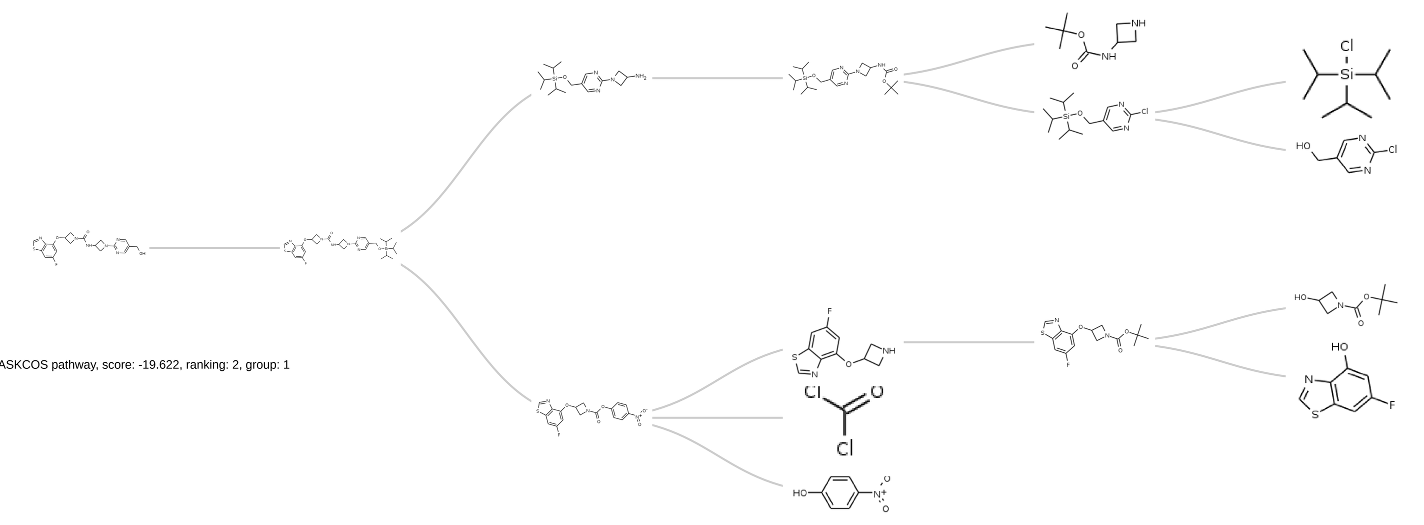

ASKCOS pathway, score: -19.622, ranking: 2, group: 1

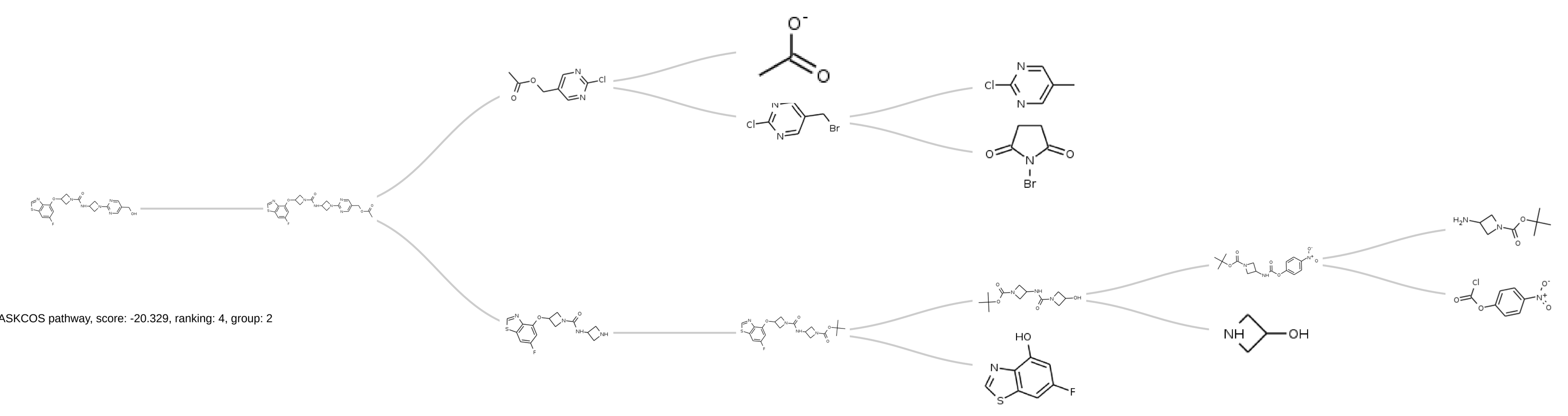

ASKCOS pathway, score: -20.329, ranking: 4, group: 2

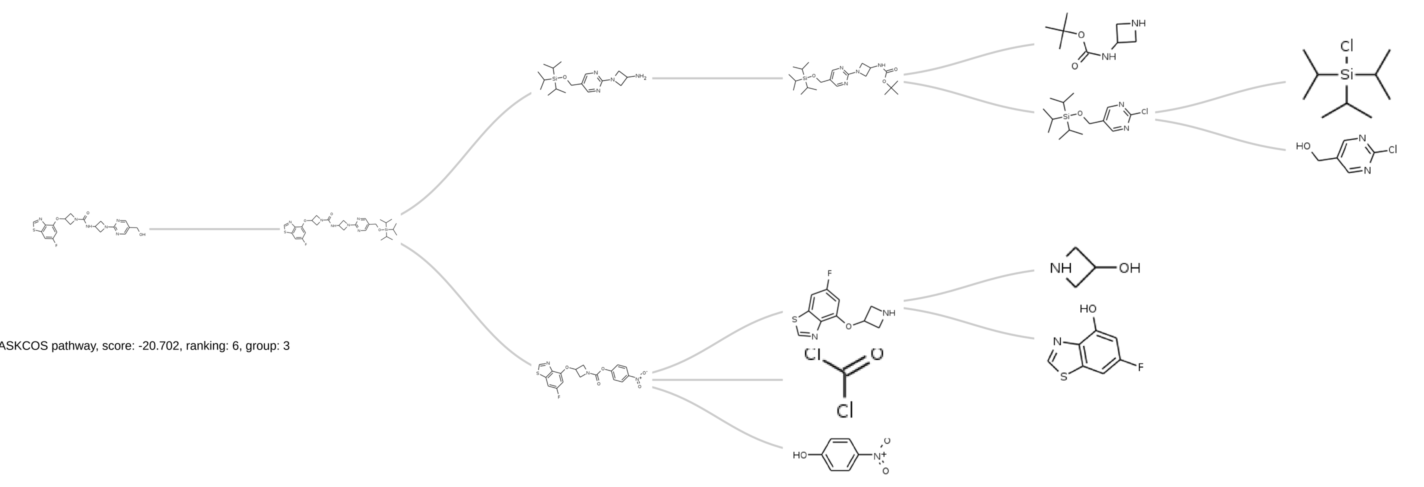

ASKCOS pathway, score: -20.702, ranking: 6, group: 3

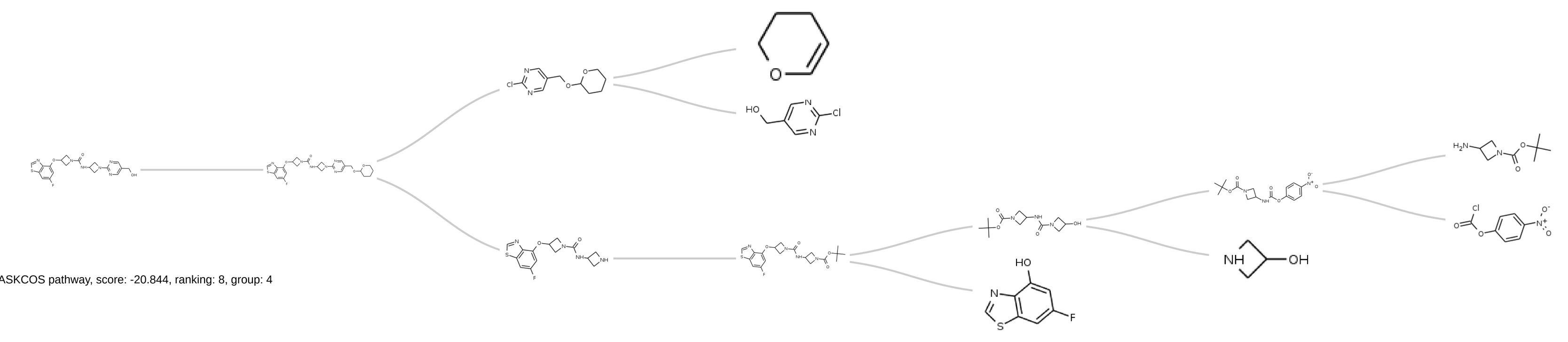

ASKCOS pathway, score: -20.844, ranking: 8, group: 4

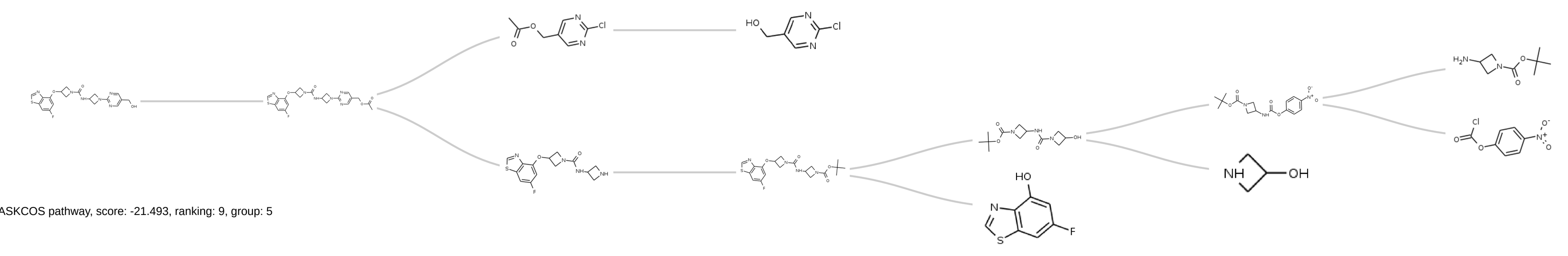

ASKCOS pathway, score: -21.493, ranking: 9, group: 5

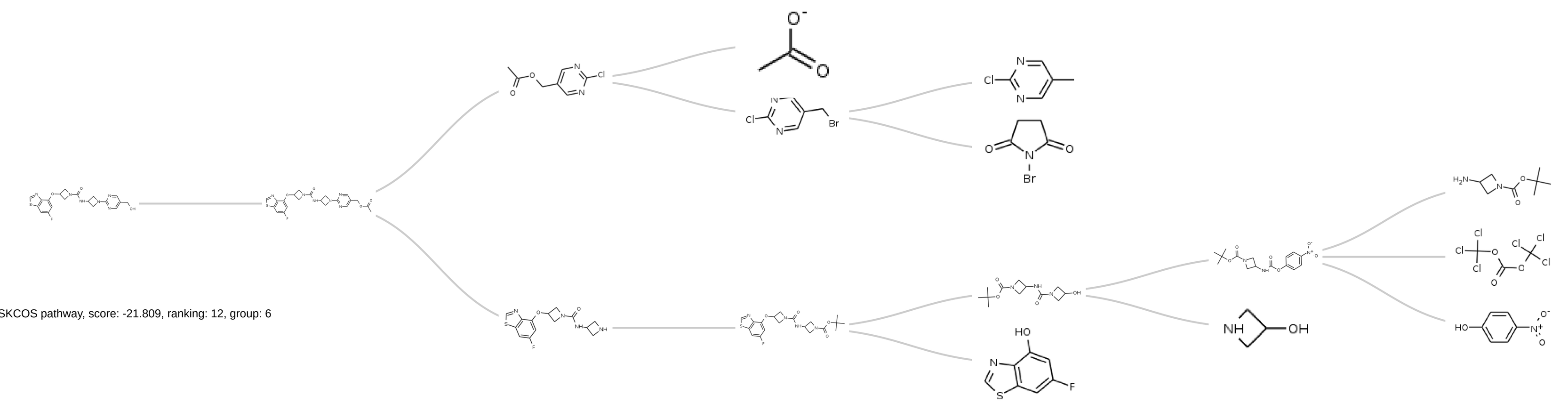

ASKCOS pathway, score: -21.809, ranking: 12, group: 6

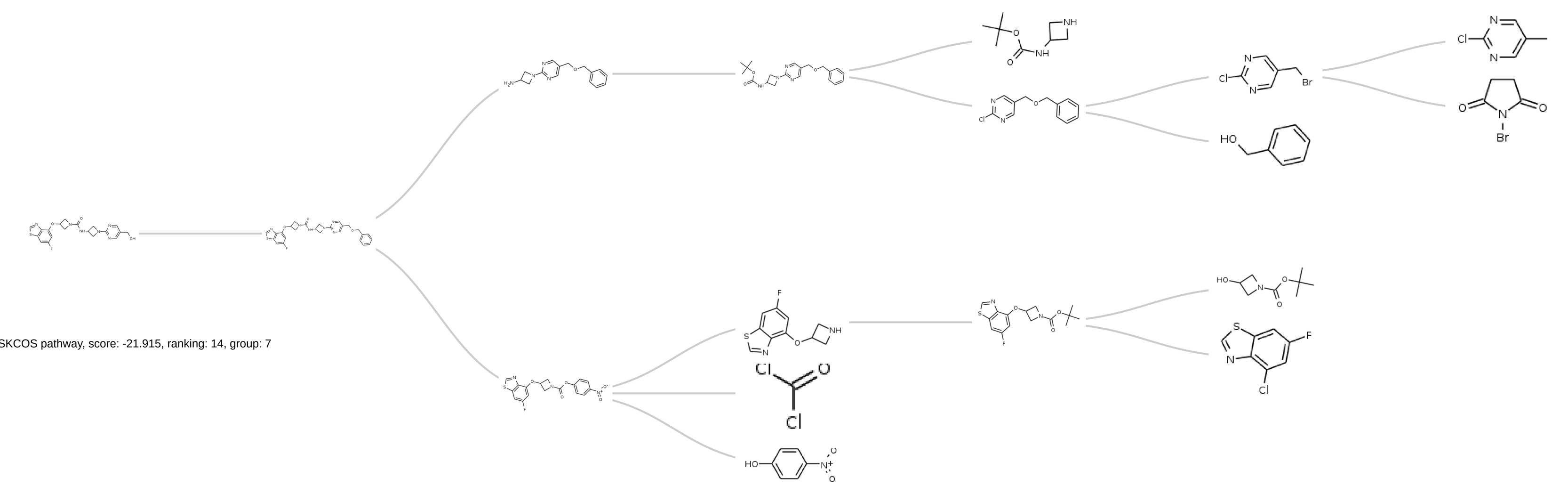

ASKCOS pathway, score: -21.915, ranking: 14, group: 7

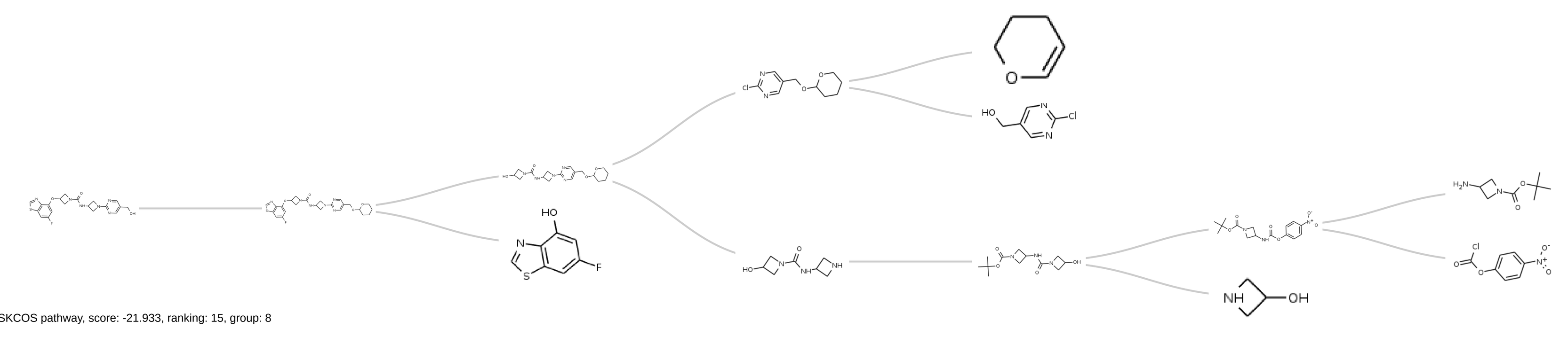

ASKCOS pathway, score: -21.933, ranking: 15, group: 8

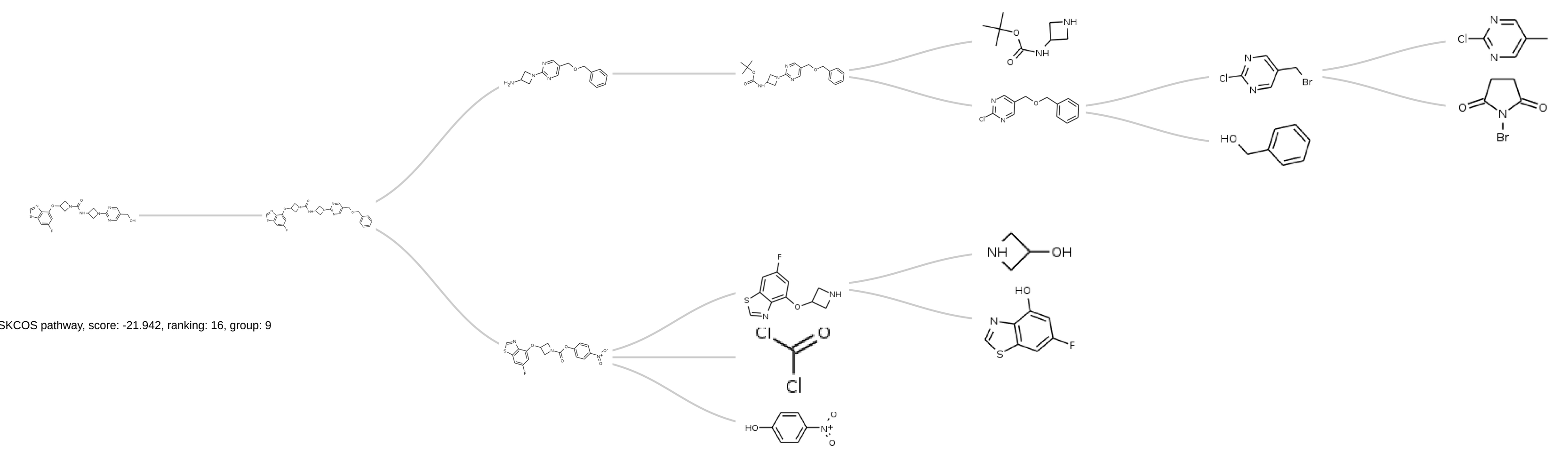

ASKCOS pathway, score: -21.942, ranking: 16, group: 9

Model ranks patent pathway as top-1: Example 26

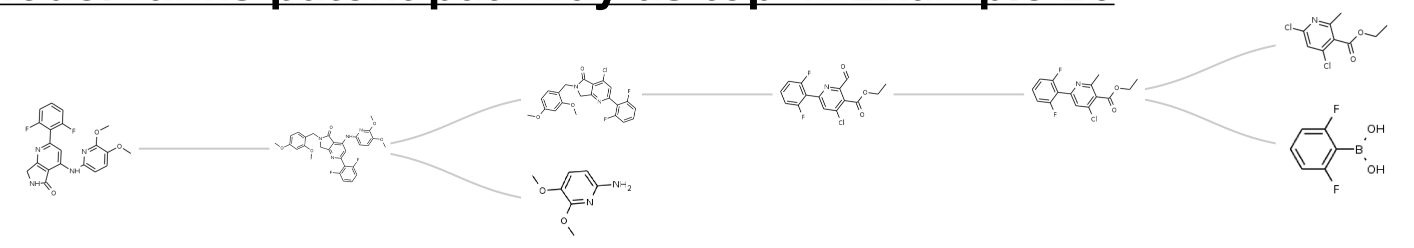

Piatachio pathway, score: -14.842, ranking: 1, Patent No: US20150266875A1

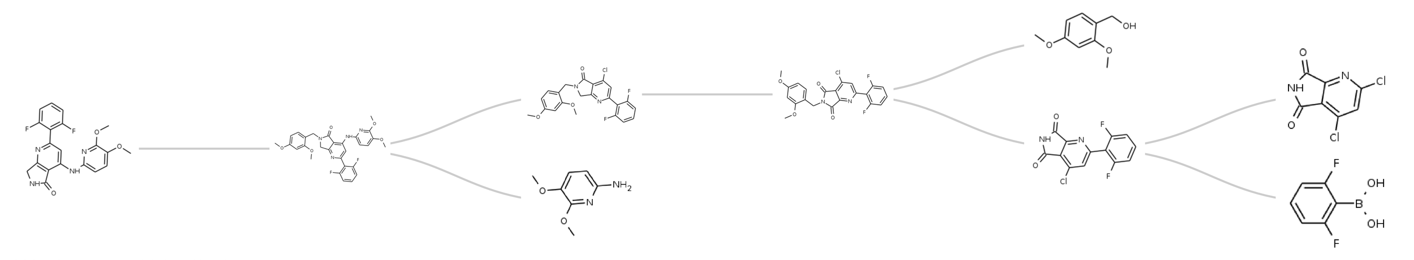

ASKCOS pathway, score: -16.356, ranking: 2, group: 1

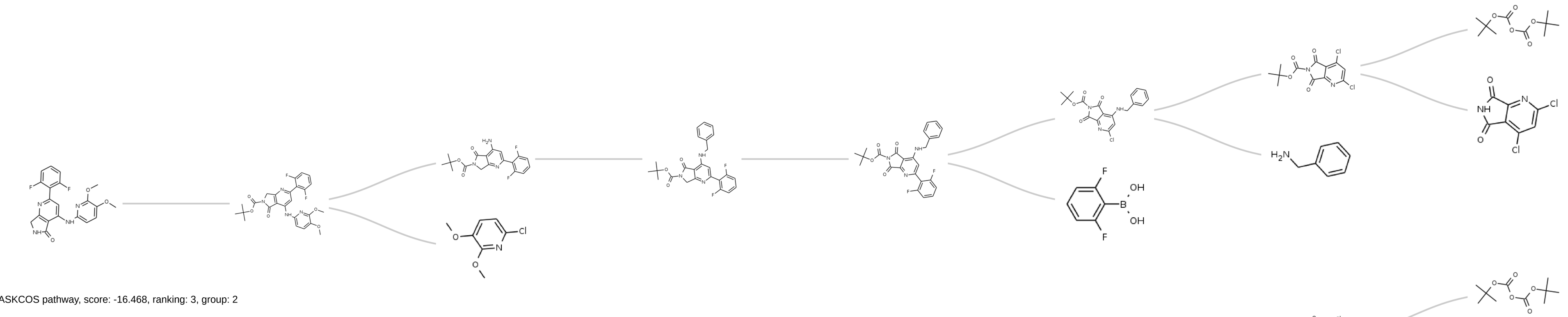

ASKCOS pathway, score: -16.468, ranking: 3, group: 2

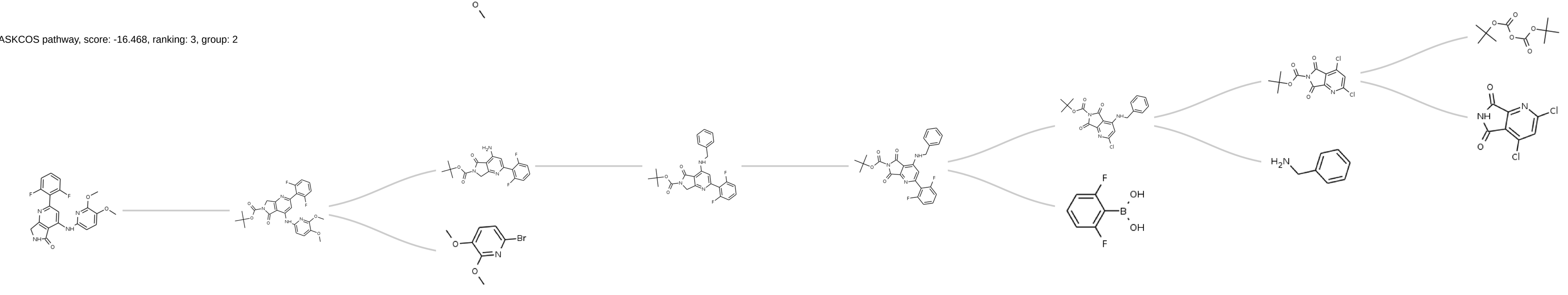

ASKCOS pathway, score: -17.779, ranking: 4, group: 3

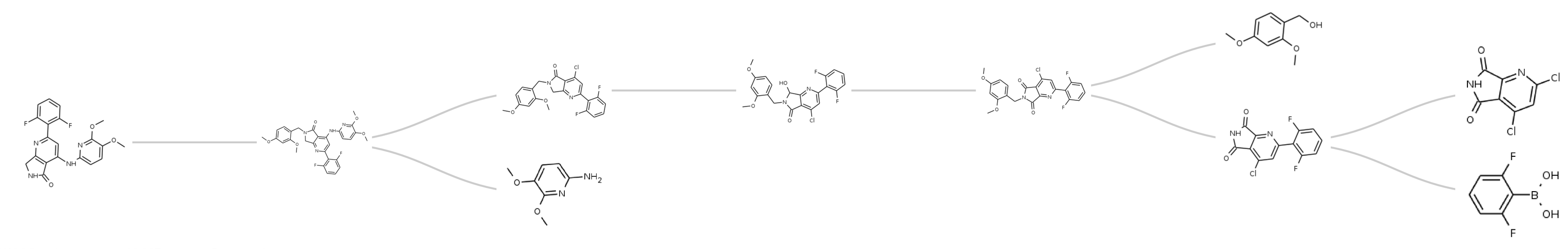

ASKCOS pathway, score: -18.897, ranking: 5, group: 4

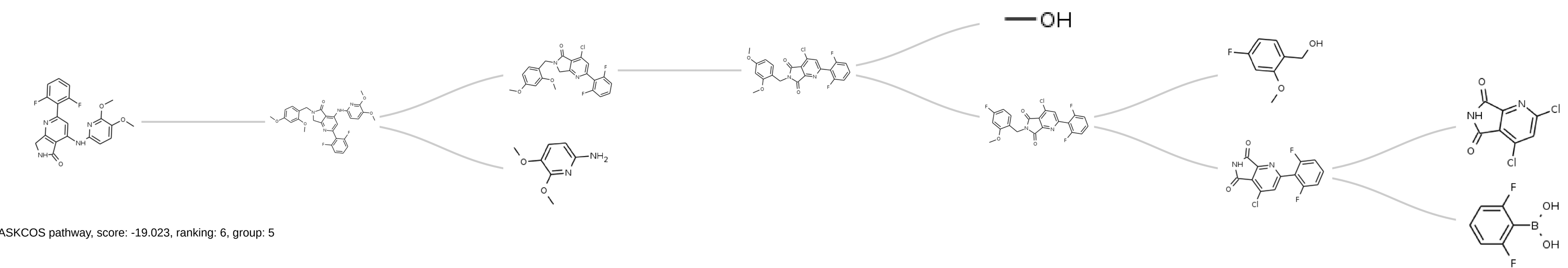

ASKCOS pathway, score: -19.023, ranking: 6, group: 5

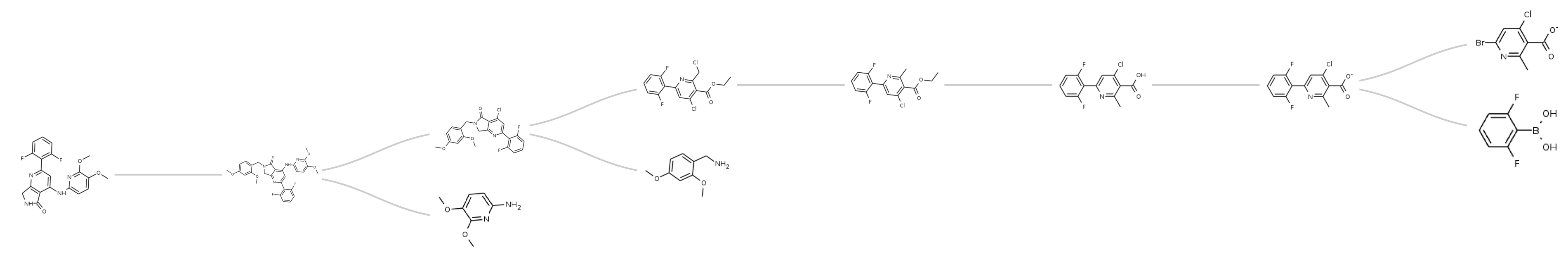

ASKCOS pathway, score: -19.479, ranking: 7, group: 6

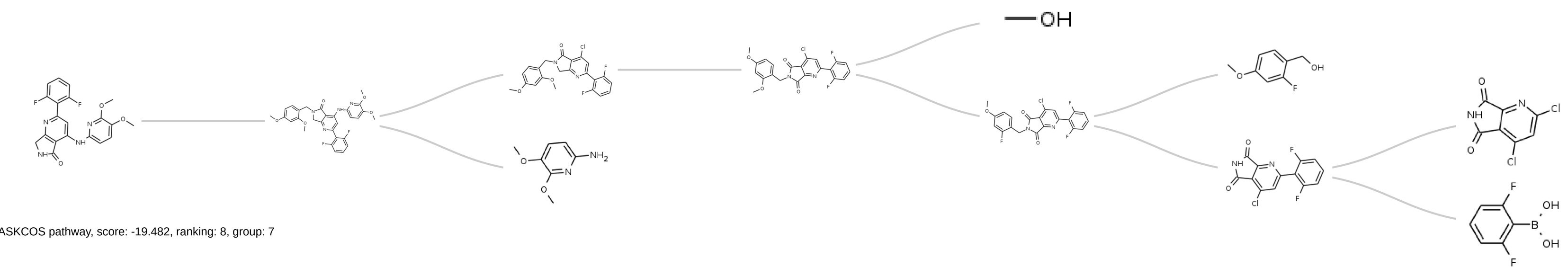

ASKCOS pathway, score: -19.482, ranking: 8, group: 7

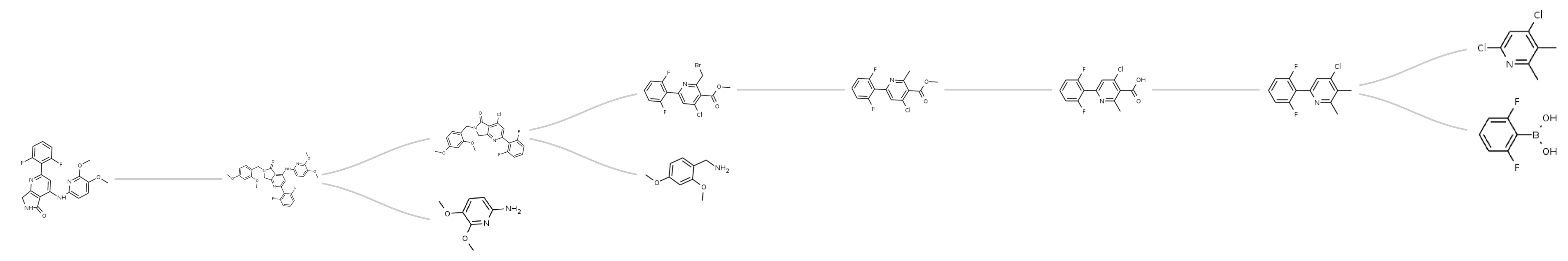

ASKCOS pathway, score: -20.894, ranking: 16, group: 8

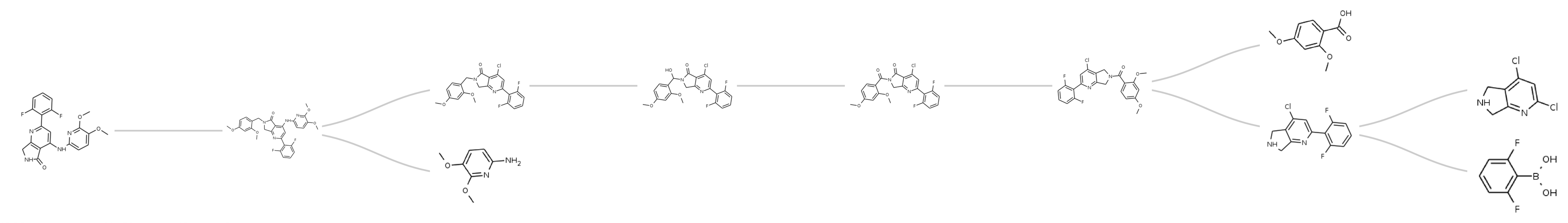

ASKCOS pathway, score: -21.178, ranking: 19, group: 9

Model ranks patent pathway as top-1: Example 27

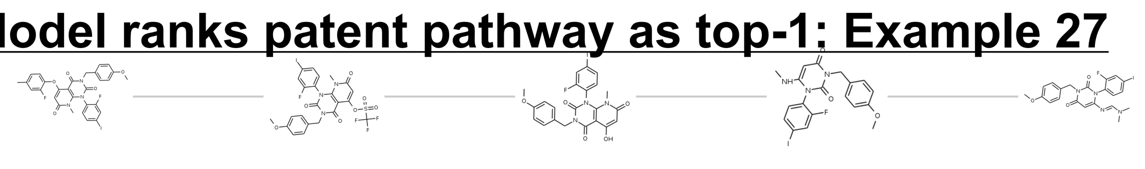

Piatachio pathway, score: 1.503, ranking: 1, Patent No: US10064848

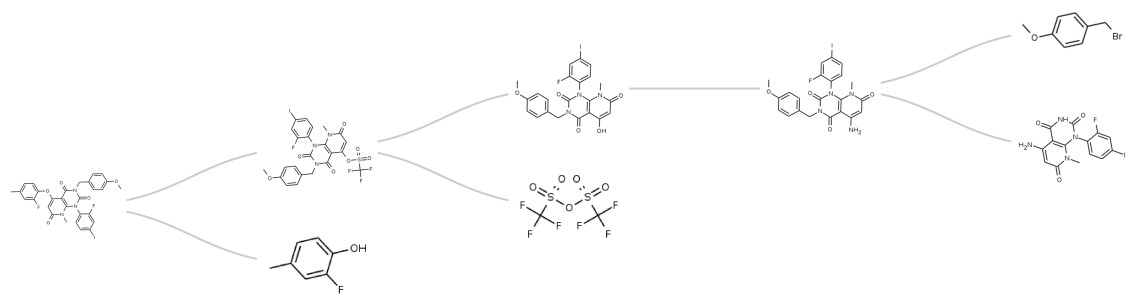

ASKCOS pathway, score: -17.367, ranking: 2, group: 1

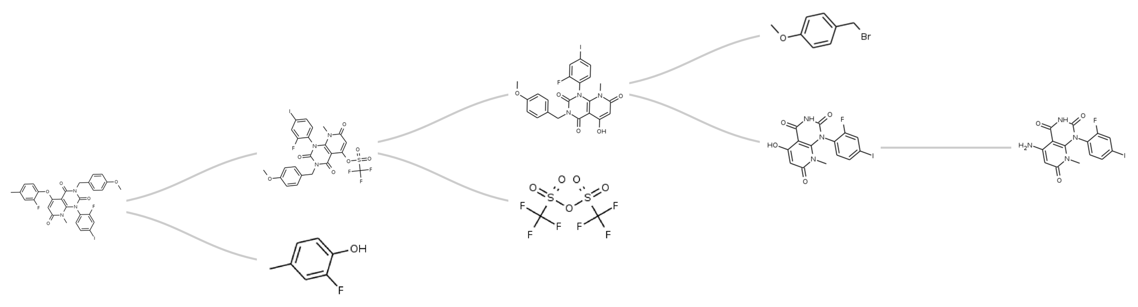

ASKCOS pathway, score: -18.055, ranking: 3, group: 2

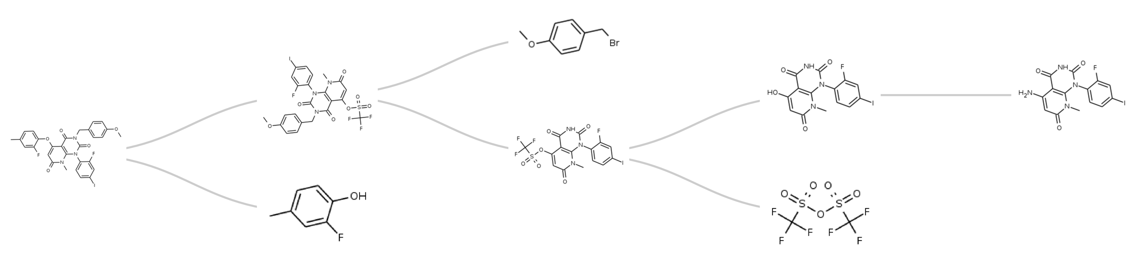

ASKCOS pathway, score: -18.070, ranking: 4, group: 3

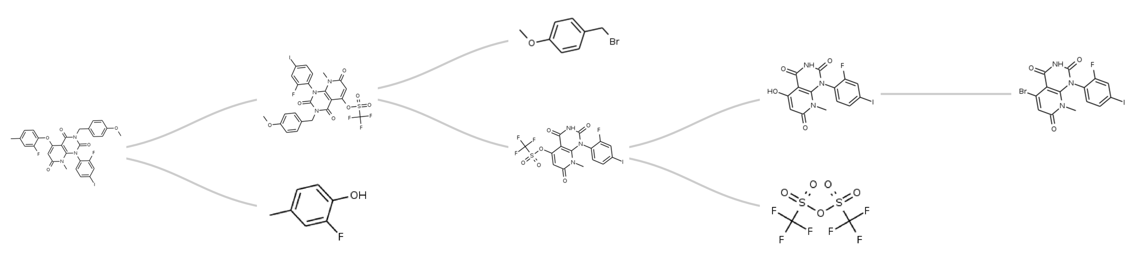

ASKCOS pathway, score: -18.211, ranking: 5, group: 4

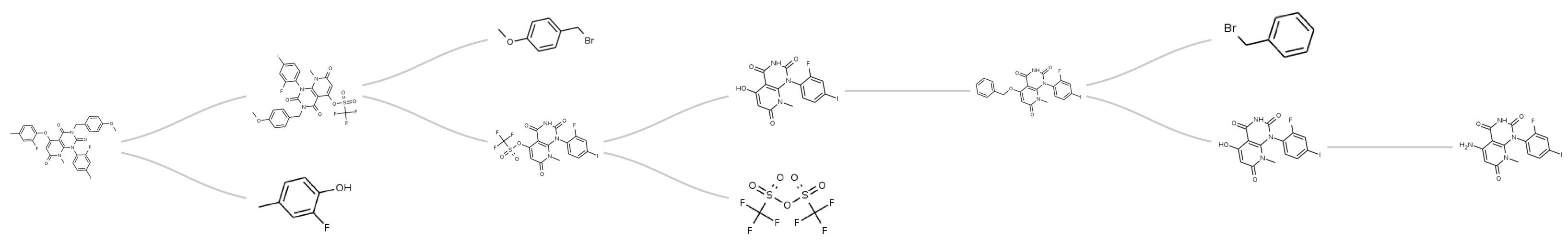

ASKCOS pathway, score: -20.907, ranking: 16, group: 5

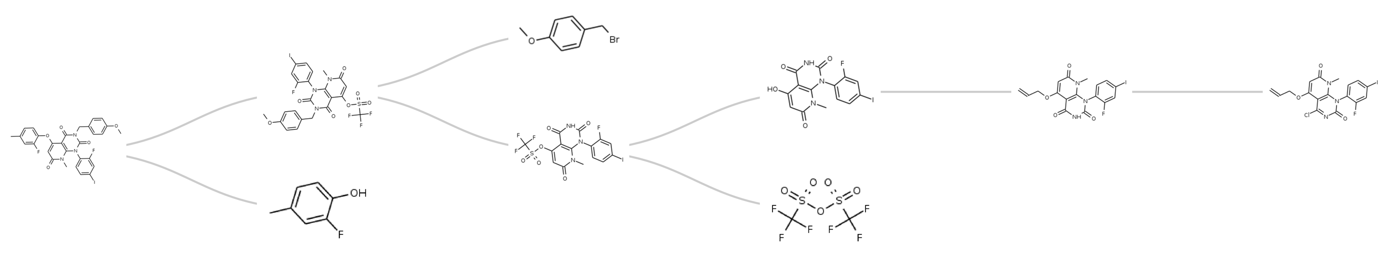

ASKCOS pathway, score: -20.919, ranking: 17, group: 6

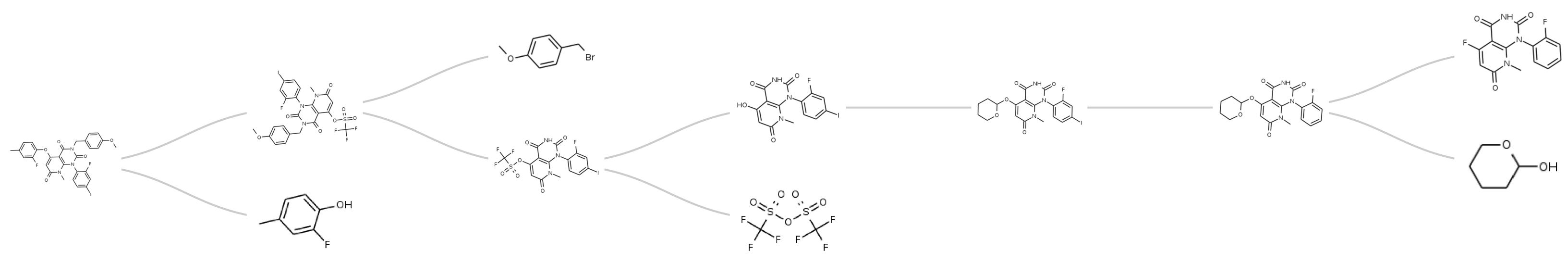

ASKCOS pathway, score: -21.134, ranking: 19, group: 7

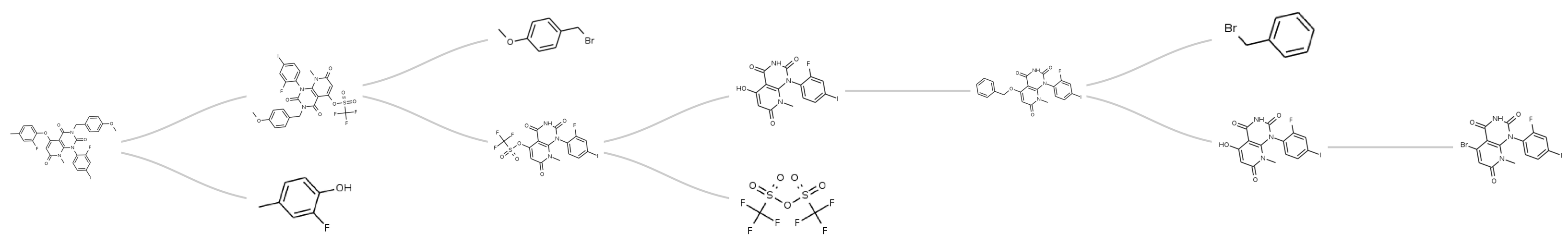

ASKCOS pathway, score: -21.148, ranking: 20, group: 8

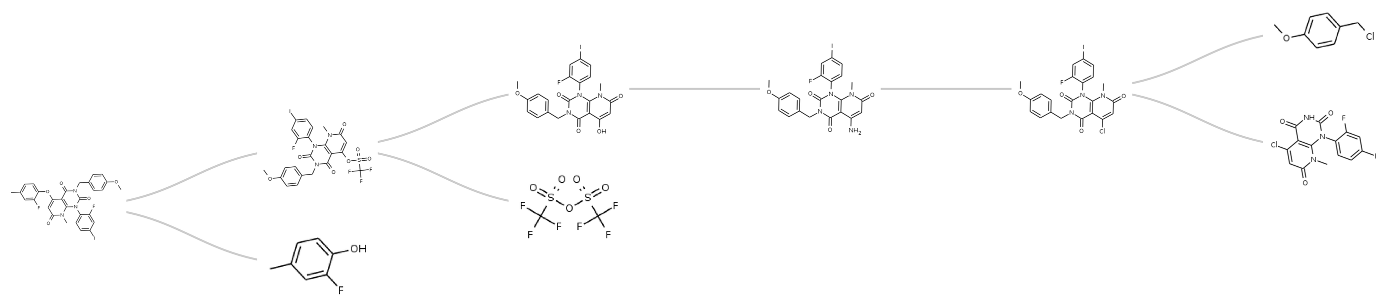

ASKCOS pathway, score: -21.272, ranking: 21, group: 9

Model ranks patent pathway as top-1: Example 28

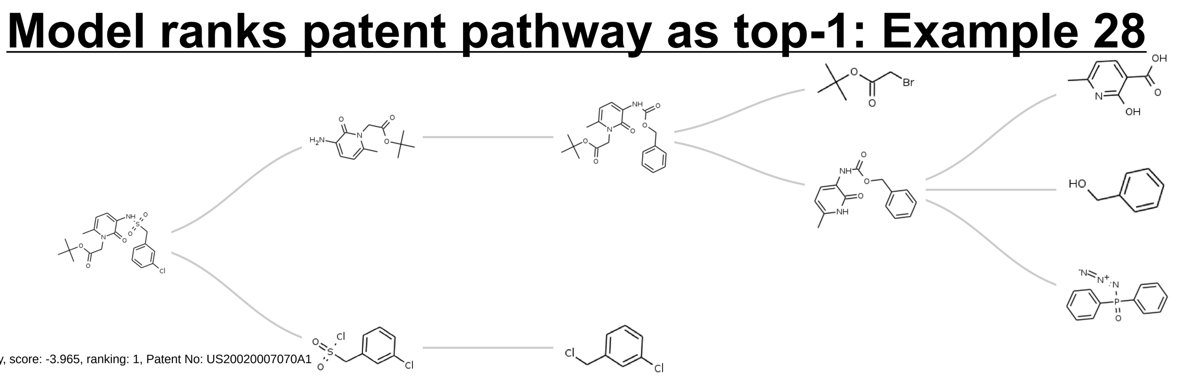

Piatachio pathway, score: -3.965, ranking: 1, Patent No: US20020007070A1

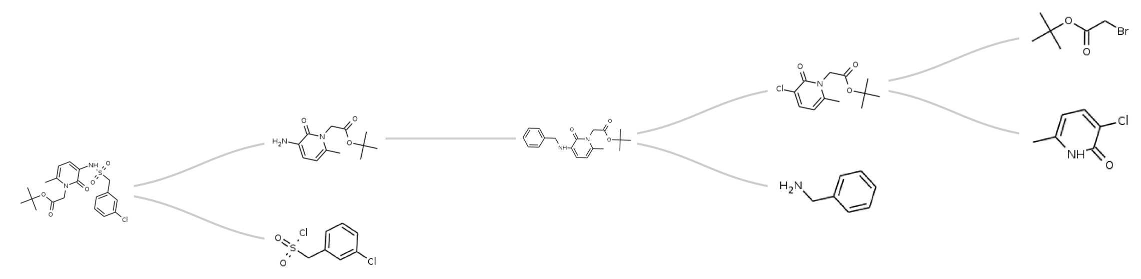

ASKCOS pathway, score: -9.148, ranking: 2, group: 1

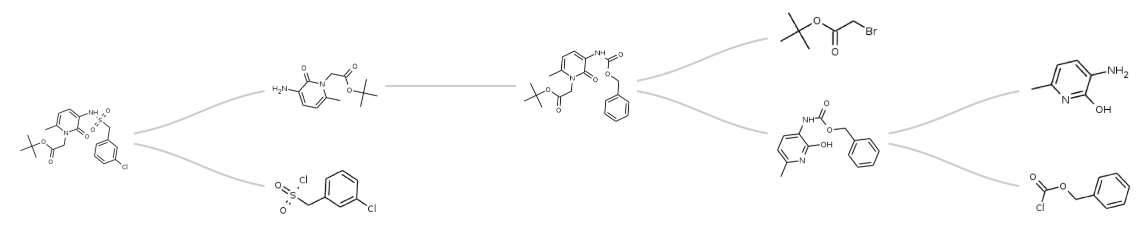

ASKCOS pathway, score: -9.278, ranking: 3, group: 2

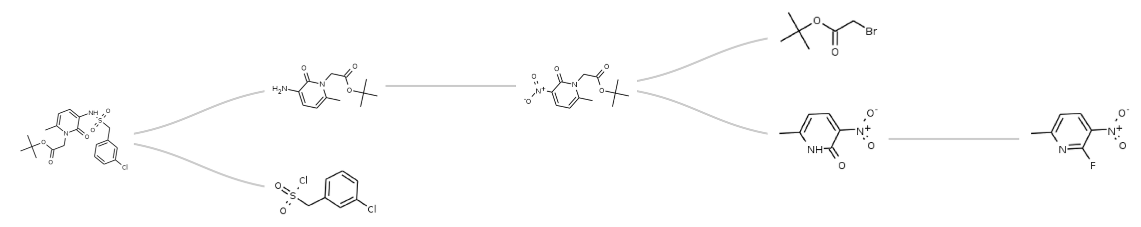

ASKCOS pathway, score: -10.478, ranking: 4, group: 3

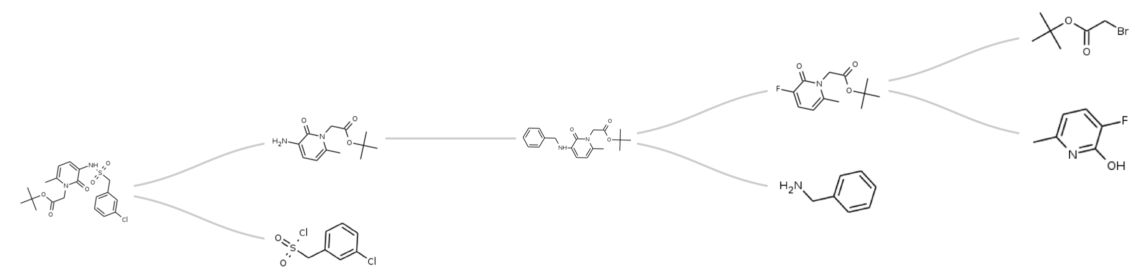

ASKCOS pathway, score: -10.581, ranking: 5, group: 4

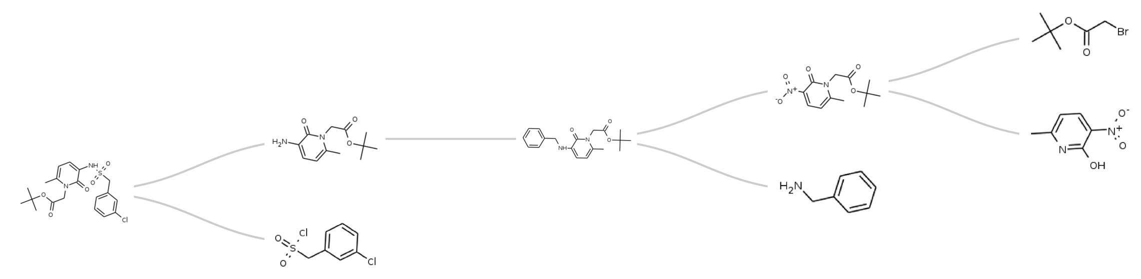

ASKCOS pathway, score: -10.684, ranking: 6, group: 5

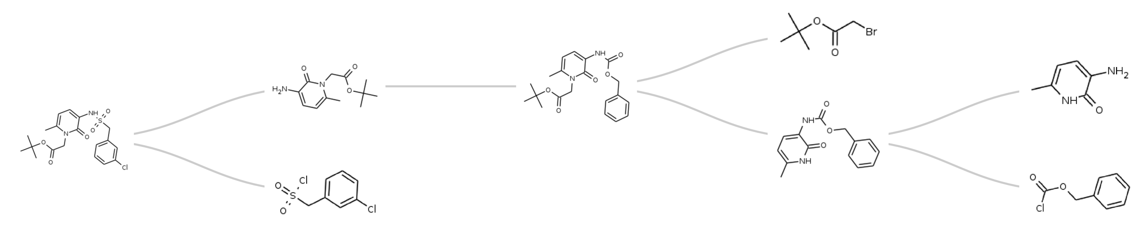

ASKCOS pathway, score: -11.363, ranking: 7, group: 6

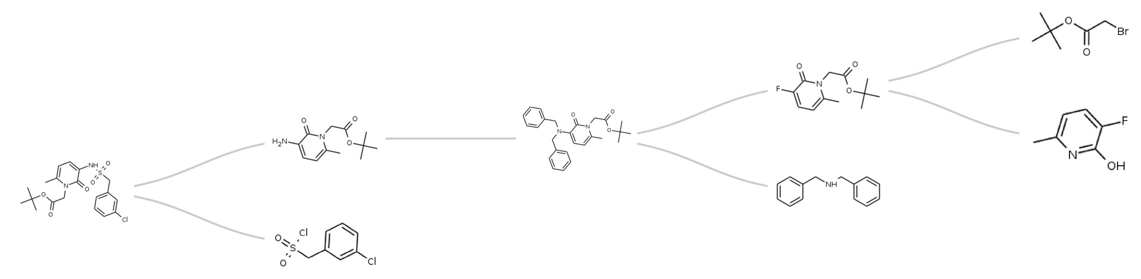

ASKCOS pathway, score: -11.565, ranking: 8, group: 7

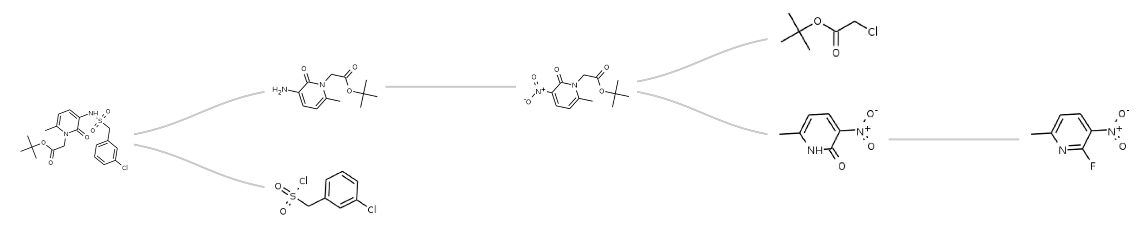

ASKCOS pathway, score: -11.680, ranking: 9, group: 8

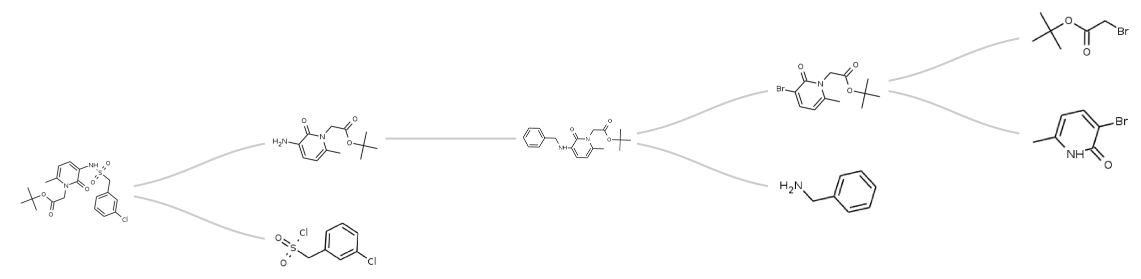

ASKCOS pathway, score: -11.750, ranking: 10, group: 9

Model ranks patent pathway as top-1: Example 29

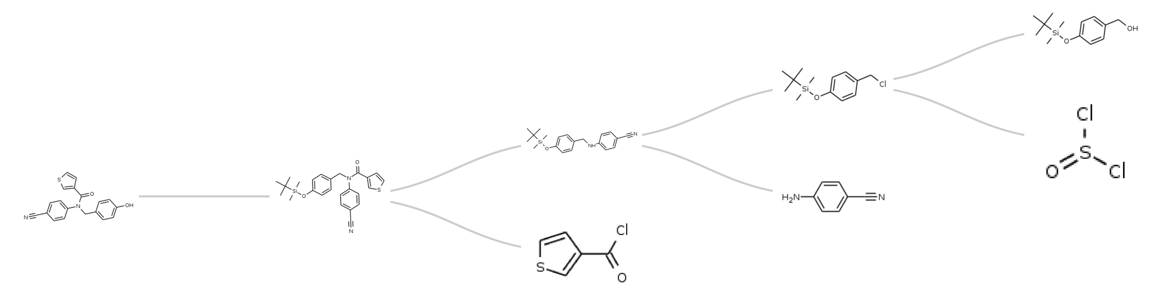

Piatachio pathway, score: -1.763, ranking: 1, Patent No: US06762205B1

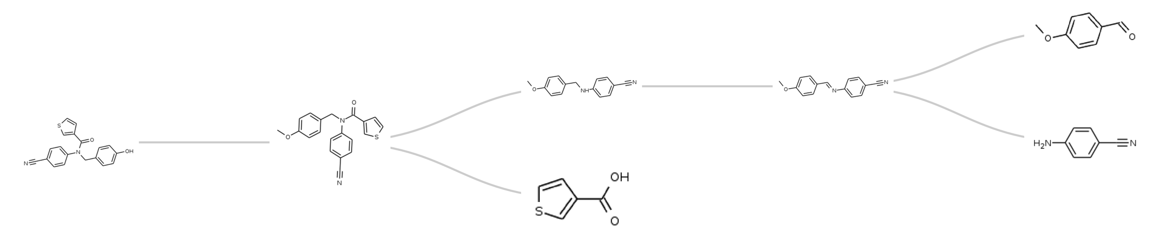

ASKCOS pathway, score: -12.198, ranking: 2, group: 1

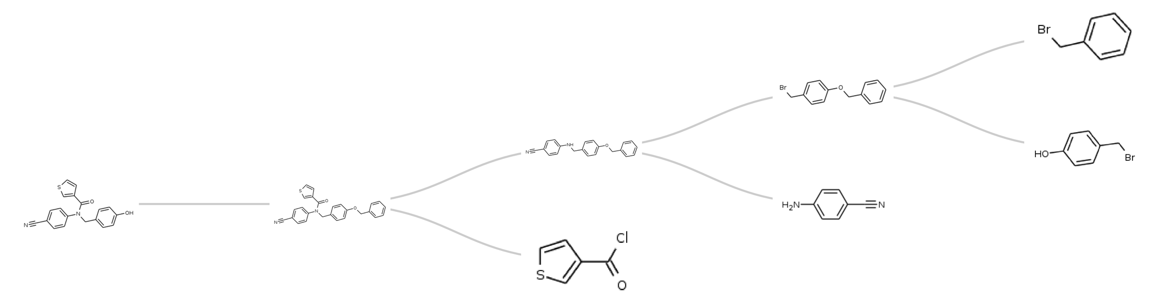

ASKCOS pathway, score: -13.710, ranking: 3, group: 2

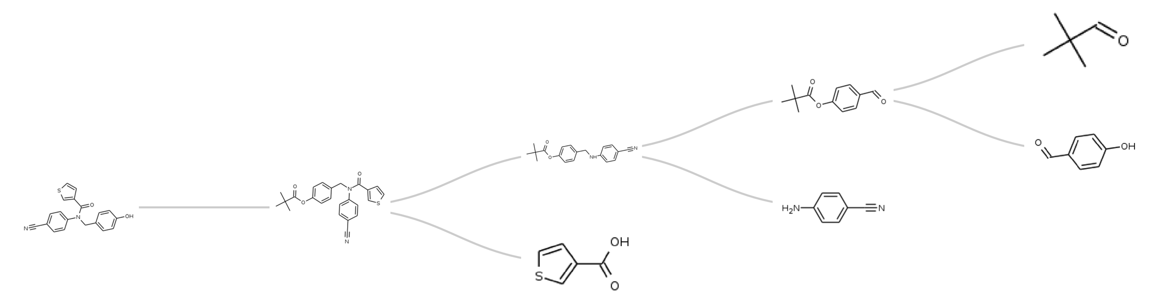

ASKCOS pathway, score: -13.942, ranking: 5, group: 3

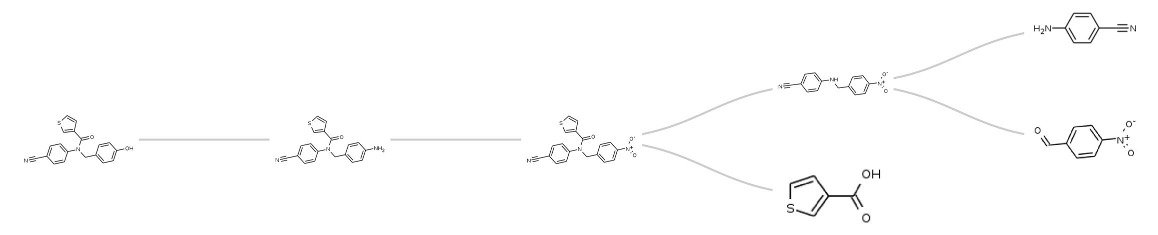

ASKCOS pathway, score: -14.397, ranking: 10, group: 4

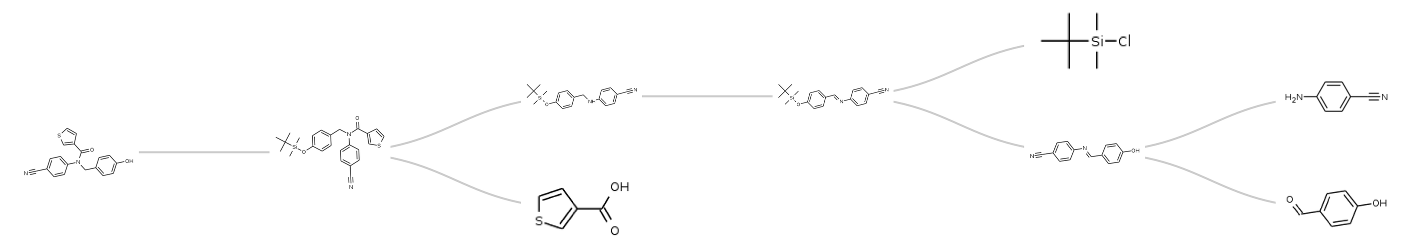

ASKCOS pathway, score: -15.287, ranking: 17, group: 5

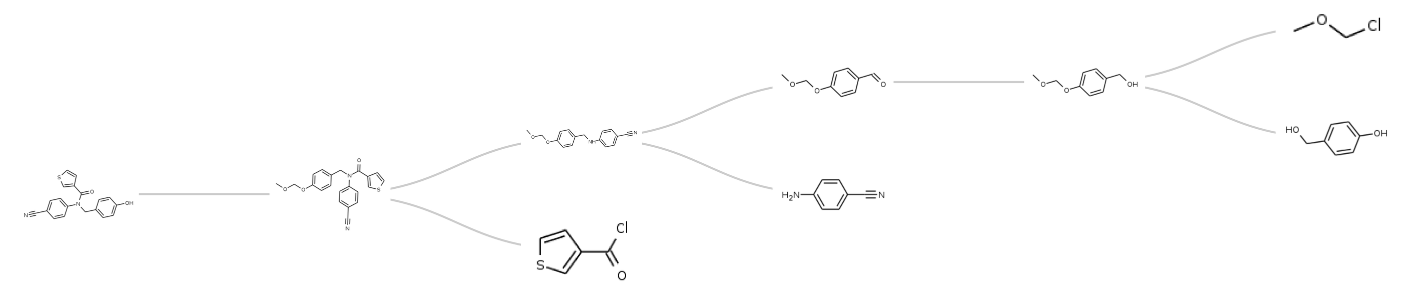

ASKCOS pathway, score: -15.865, ranking: 24, group: 6

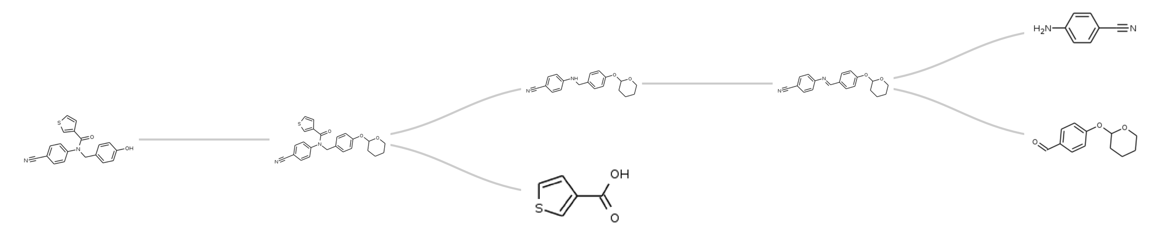

ASKCOS pathway, score: -16.348, ranking: 29, group: 7

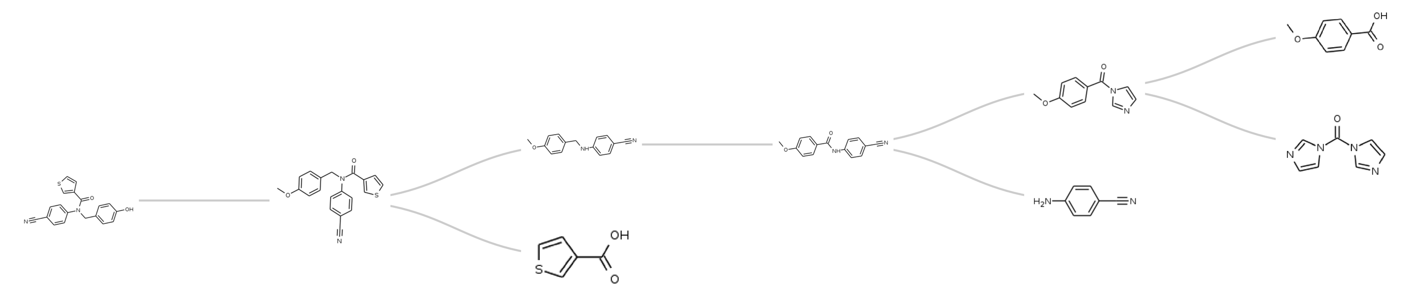

ASKCOS pathway, score: -17.345, ranking: 52, group: 8

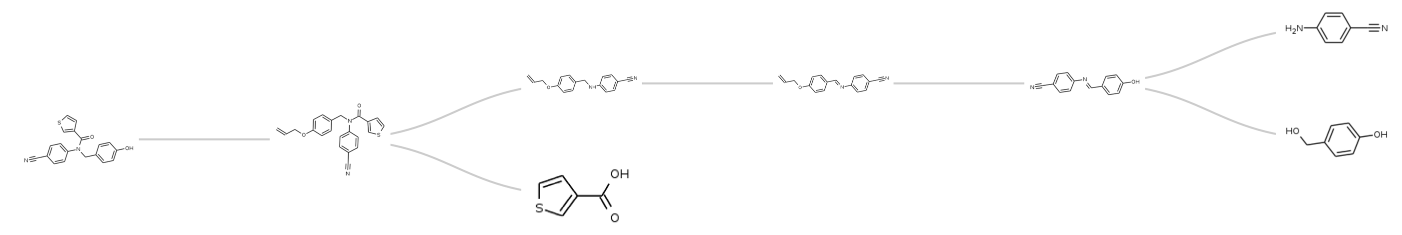

ASKCOS pathway, score: -17.495, ranking: 56, group: 9

Model ranks patent pathway as top-1: Example 30

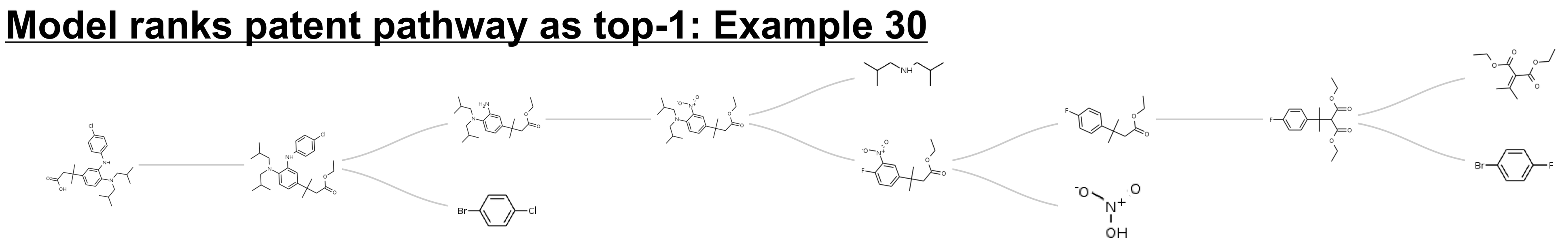

Piatachio pathway, score: -1.035, ranking: 1, Patent No: US20170231999A1

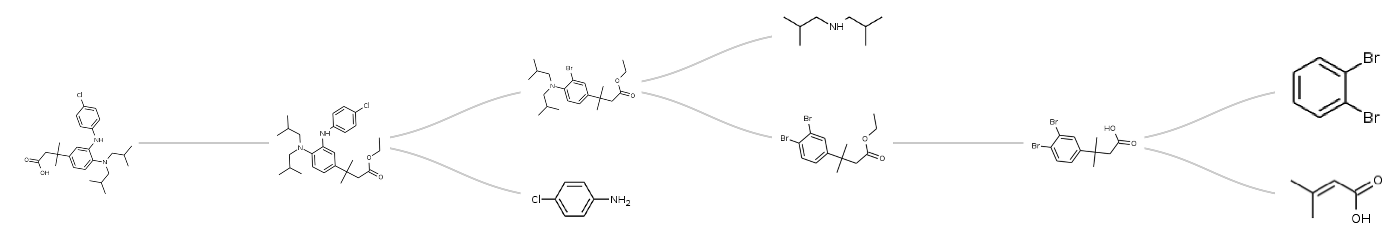

ASKCOS pathway, score: -13.350, ranking: 2, group: 1

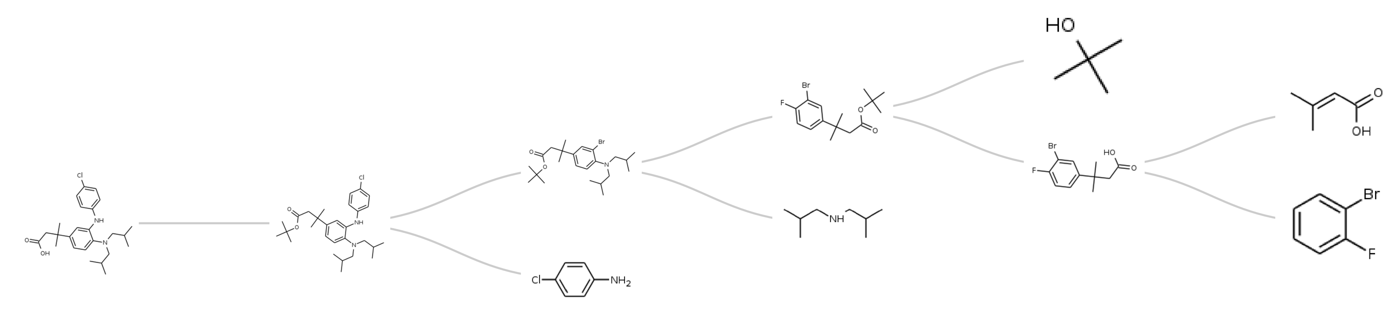

ASKCOS pathway, score: -14.401, ranking: 4, group: 2

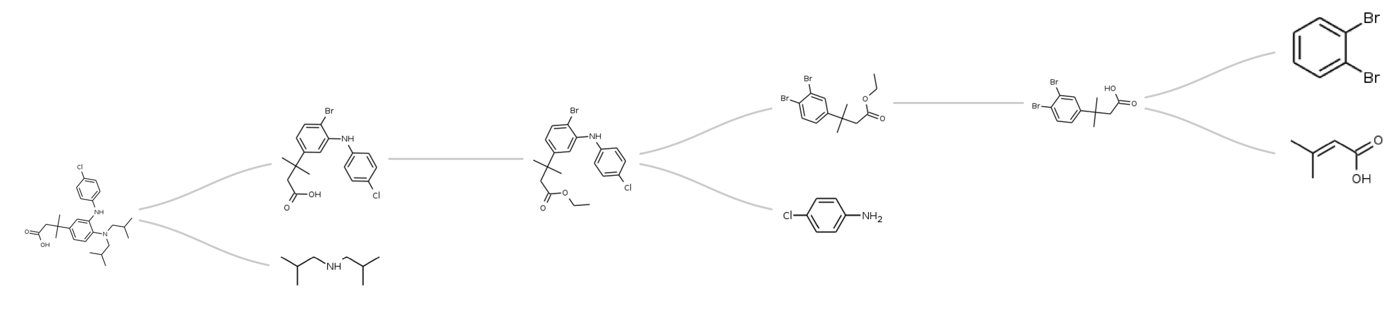

ASKCOS pathway, score: -14.583, ranking: 6, group: 3

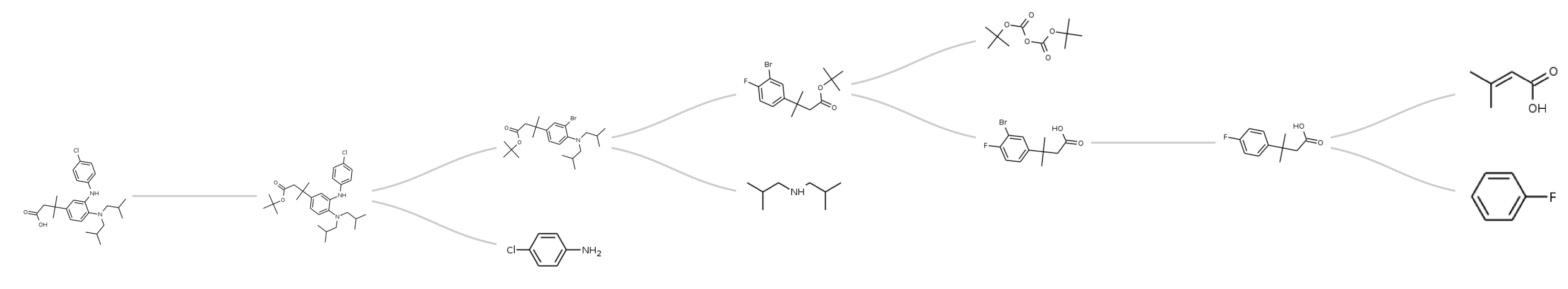

ASKCOS pathway, score: -16.446, ranking: 10, group: 4

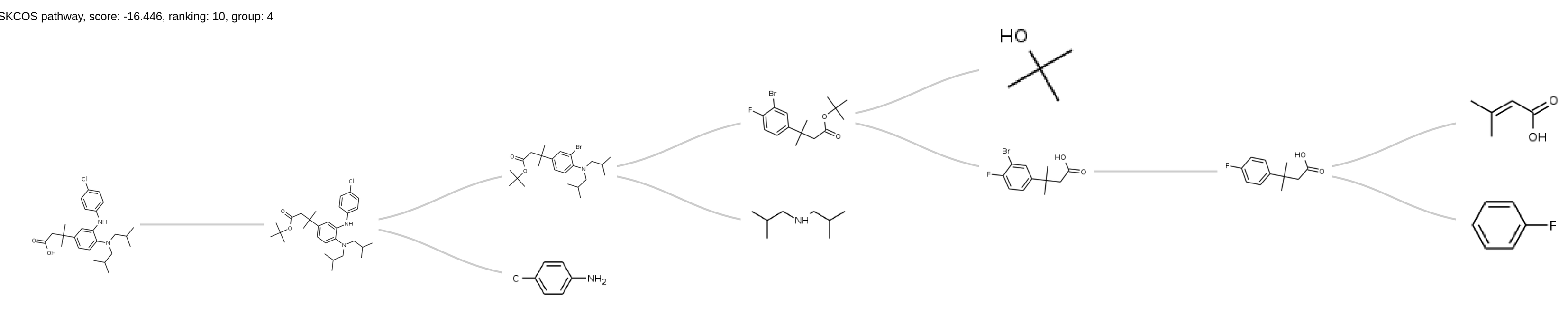

ASKCOS pathway, score: -16.858, ranking: 12, group: 5

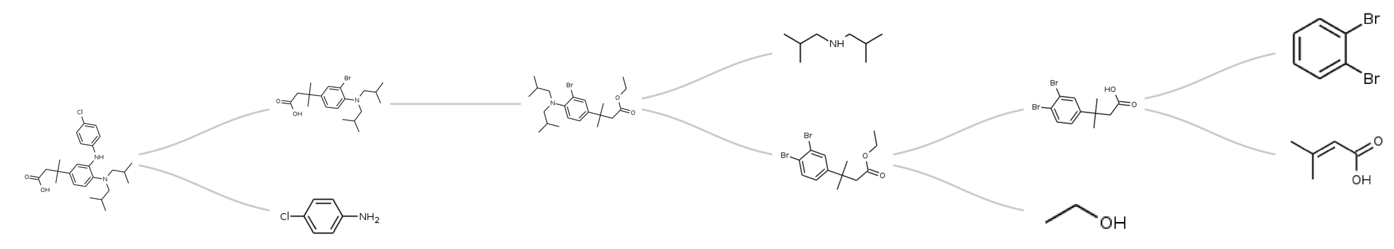

ASKCOS pathway, score: -17.205, ranking: 15, group: 6

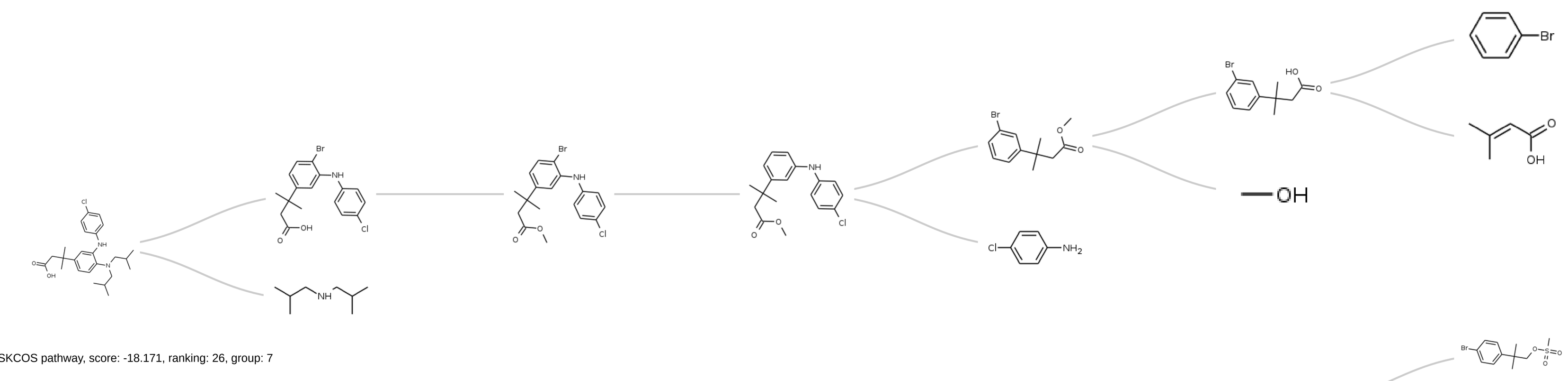

ASKCOS pathway, score: -18.171, ranking: 26, group: 7

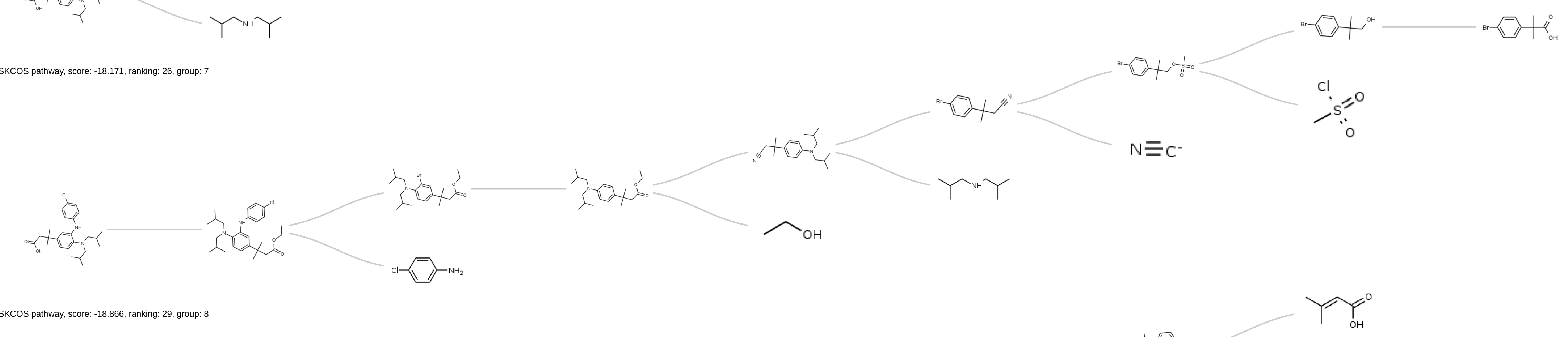

ASKCOS pathway, score: -18.866, ranking: 29, group: 8

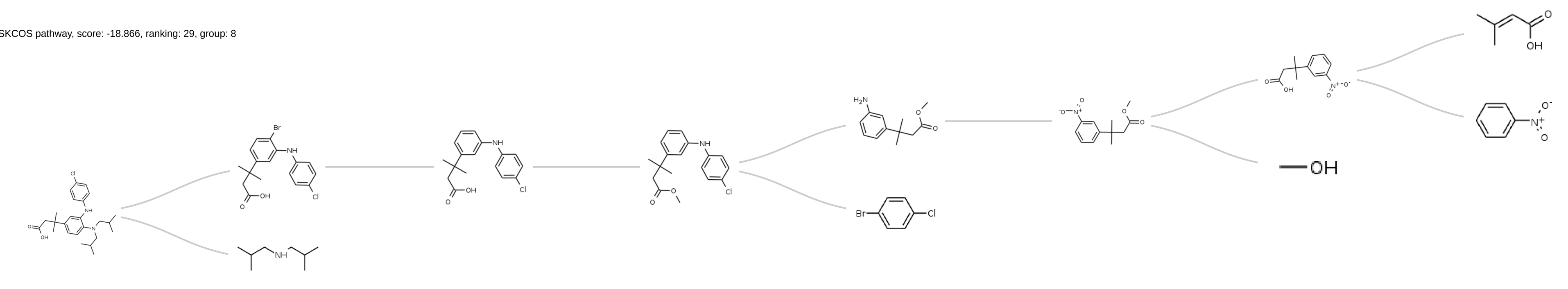

ASKCOS pathway, score: -19.002, ranking: 31, group: 9

Model ranks patent pathway as top-1: Example 31

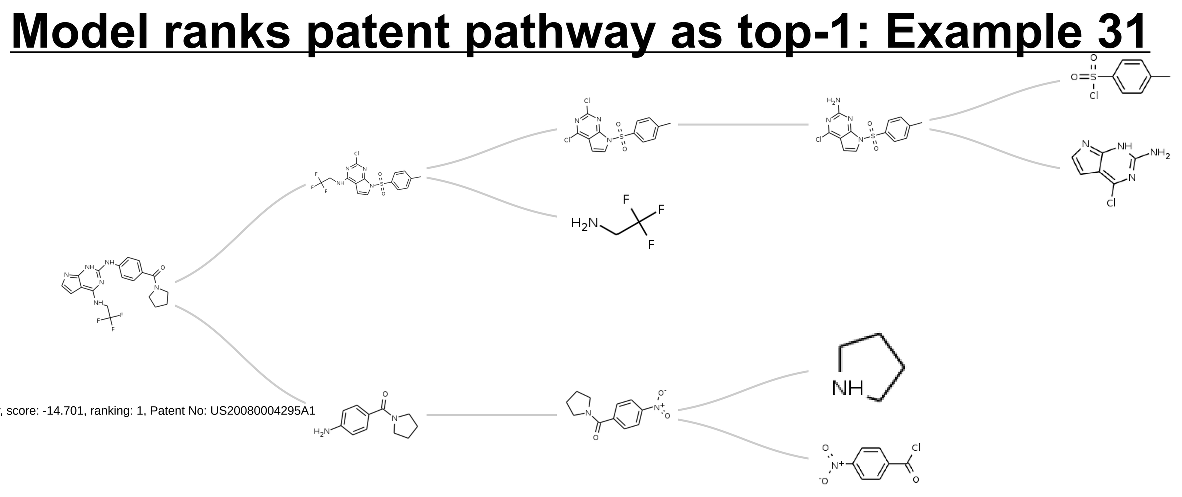

Piatachio pathway, score: -14.701, ranking: 1, Patent No: US20080004295A1

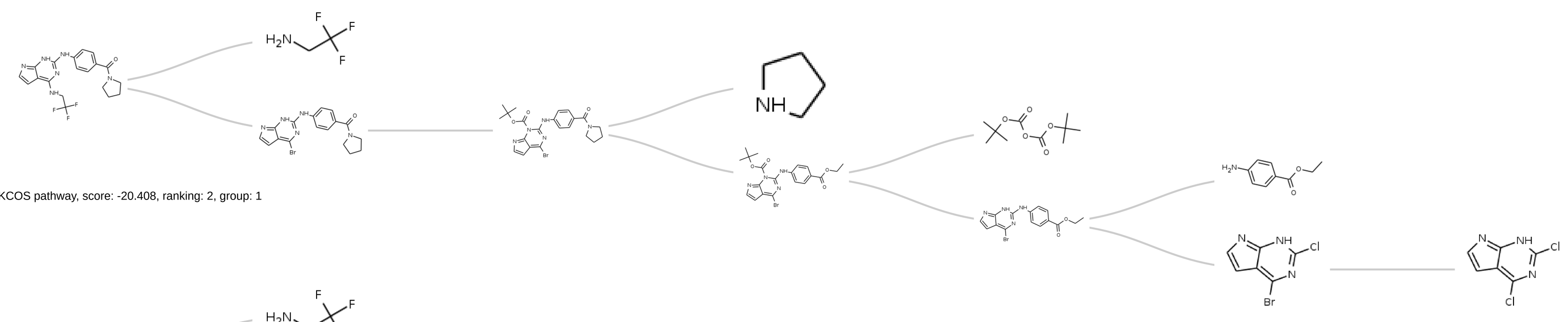

ASKCOS pathway, score: -20.408, ranking: 2, group: 1

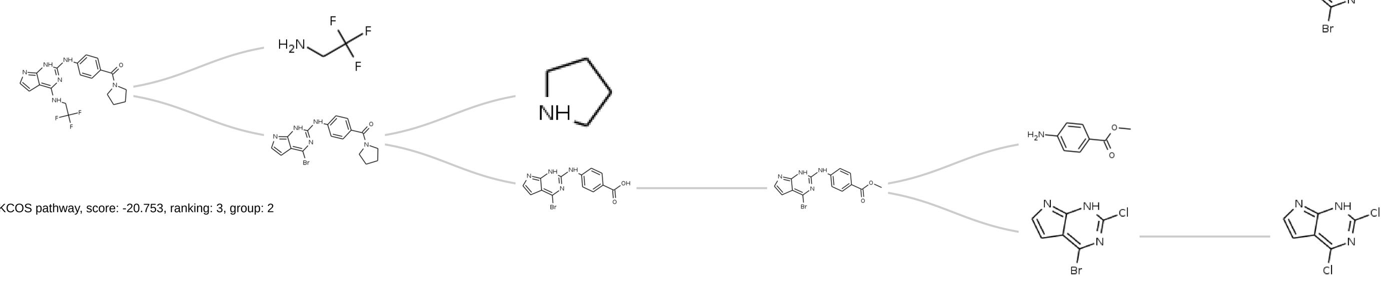

ASKCOS pathway, score: -20.753, ranking: 3, group: 2

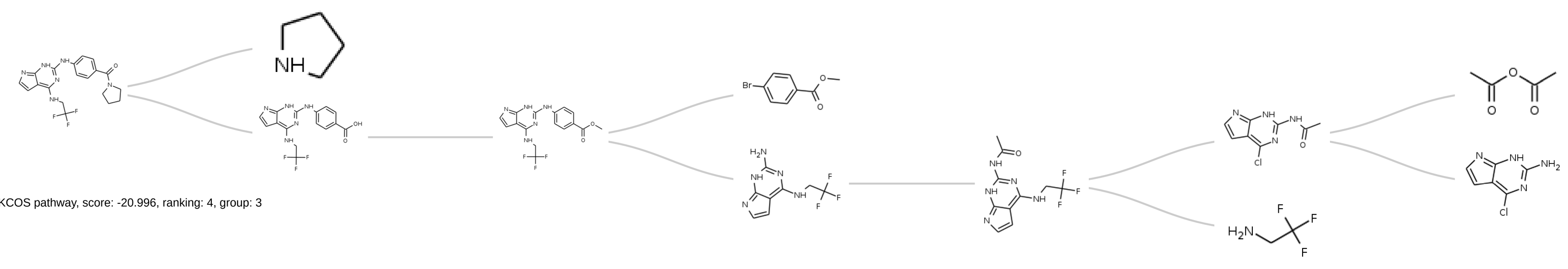

ASKCOS pathway, score: -20.996, ranking: 4, group: 3

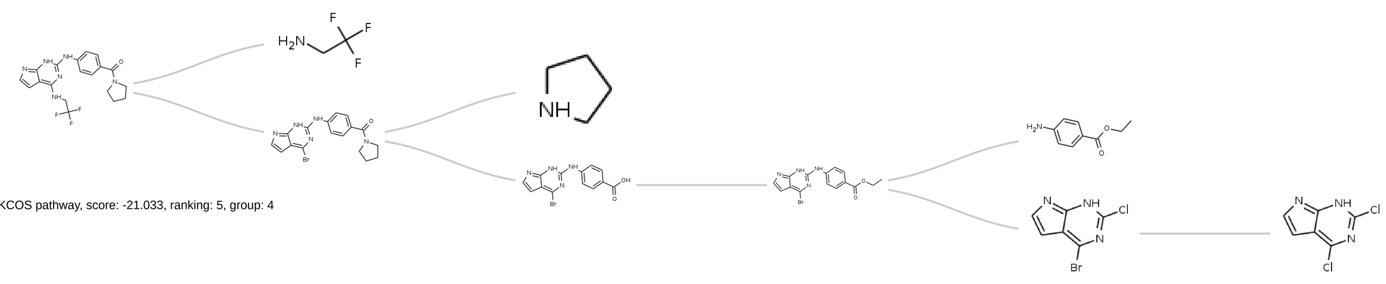

ASKCOS pathway, score: -21.033, ranking: 5, group: 4

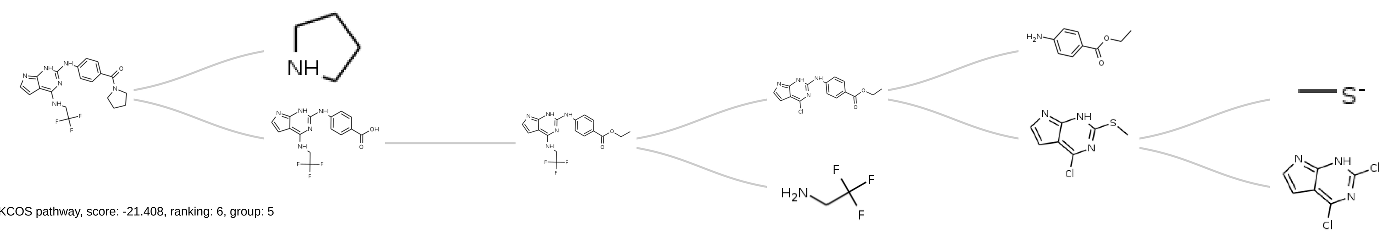

ASKCOS pathway, score: -21.408, ranking: 6, group: 5

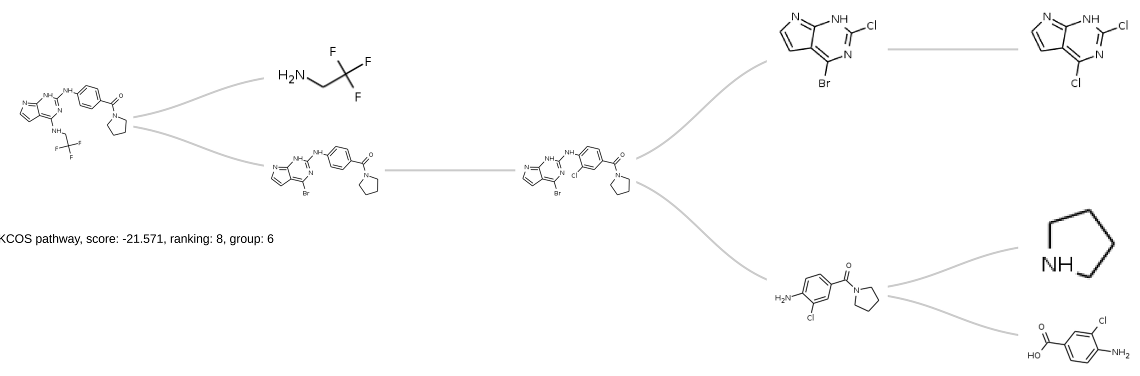

ASKCOS pathway, score: -21.571, ranking: 8, group: 6

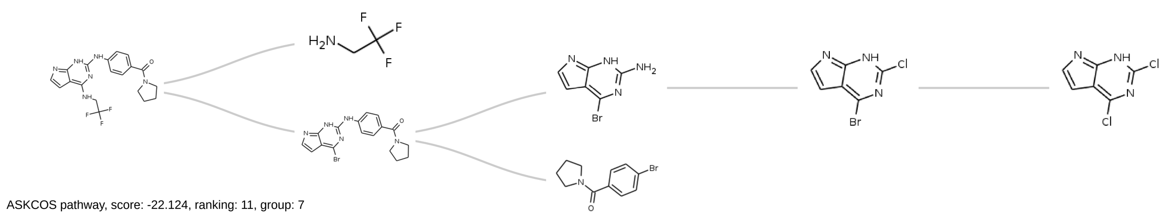

ASKCOS pathway, score: -22.124, ranking: 11, group: 7

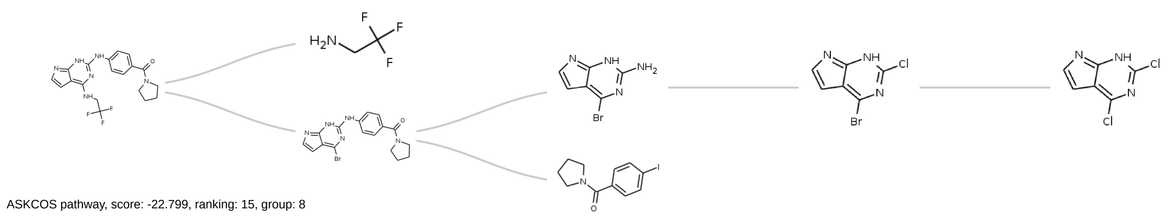

ASKCOS pathway, score: -22.799, ranking: 15, group: 8

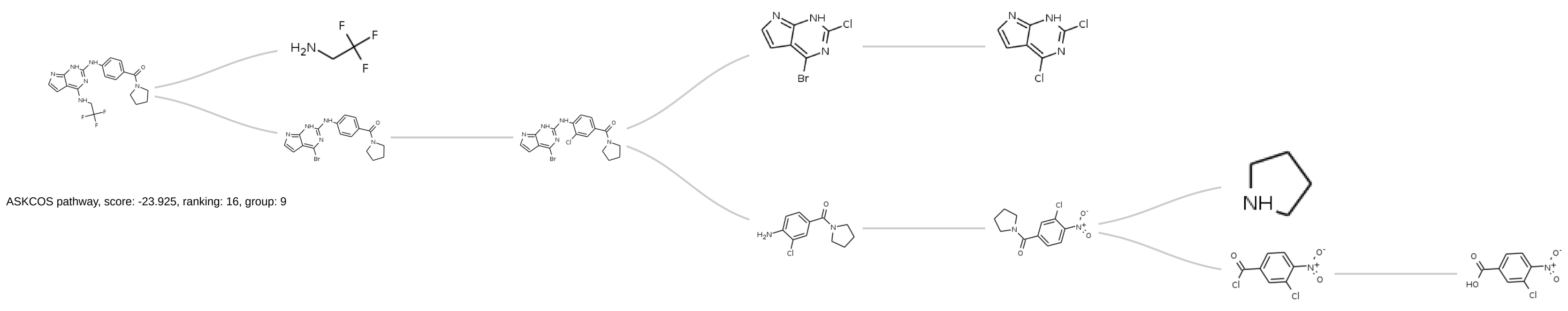

ASKCOS pathway, score: -23.925, ranking: 16, group: 9

Model ranks patent pathway as top-1: Example 32

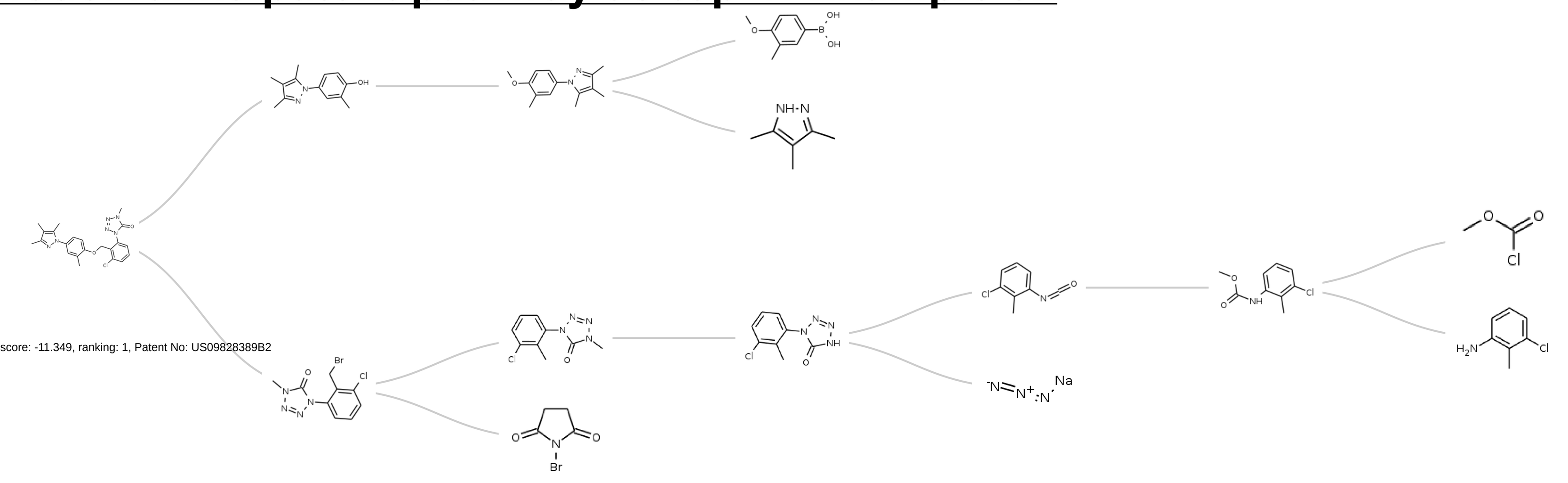

Piatachio pathway, score: -11.349, ranking: 1, Patent No: US09828389B2

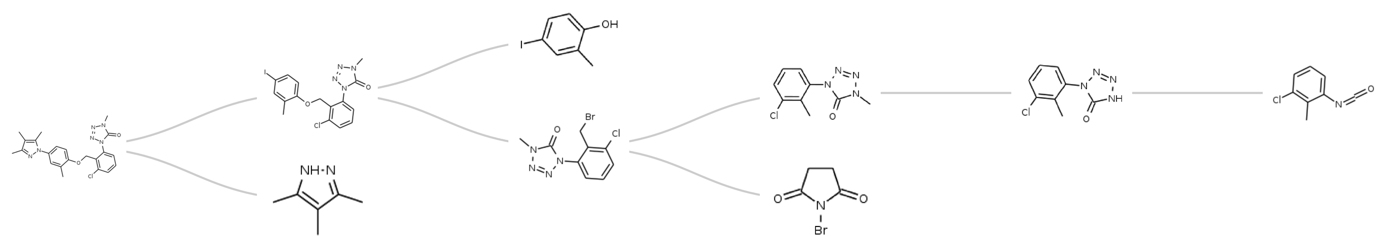

ASKCOS pathway, score: -14.441, ranking: 2, group: 1

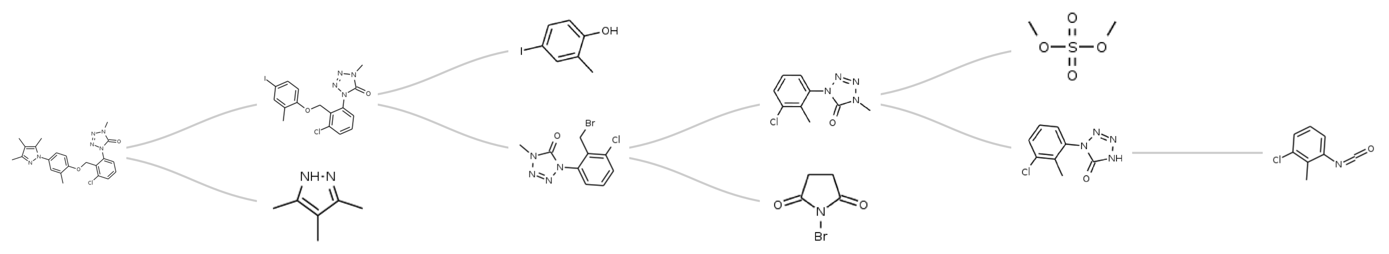

ASKCOS pathway, score: -16.547, ranking: 3, group: 2

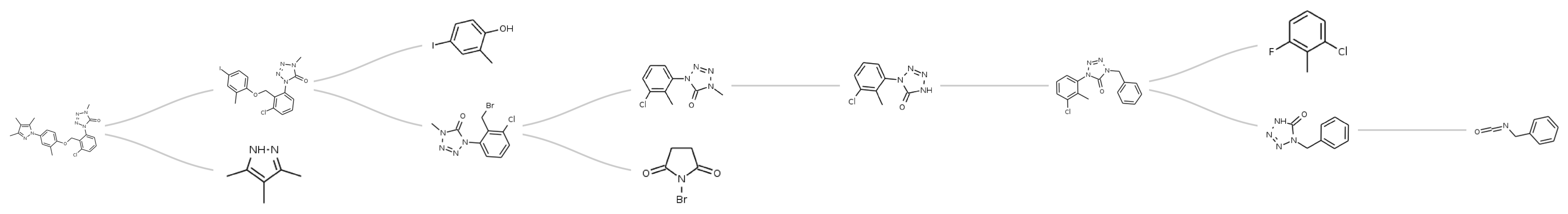

ASKCOS pathway, score: -17.599, ranking: 4, group: 3

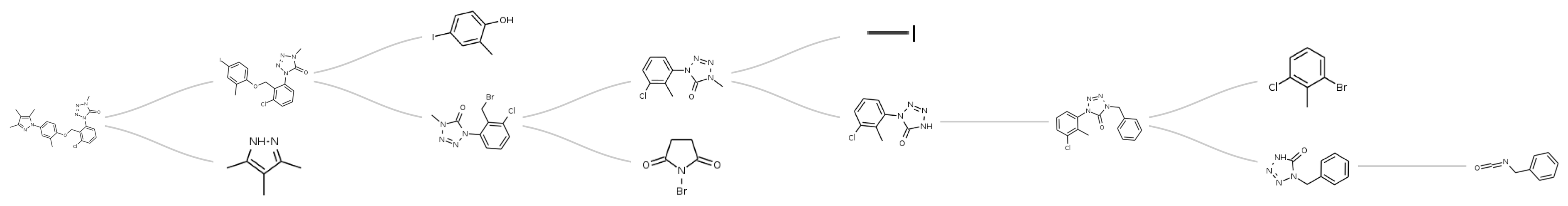

ASKCOS pathway, score: -17.903, ranking: 5, group: 4

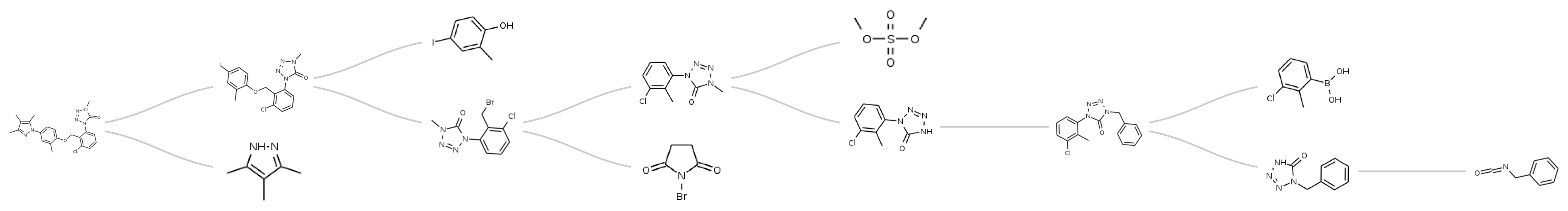

ASKCOS pathway, score: -19.042, ranking: 6, group: 5

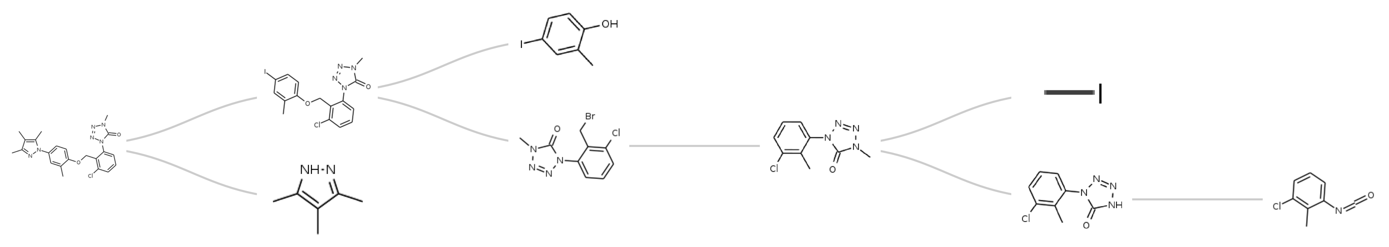

ASKCOS pathway, score: -19.066, ranking: 7, group: 6

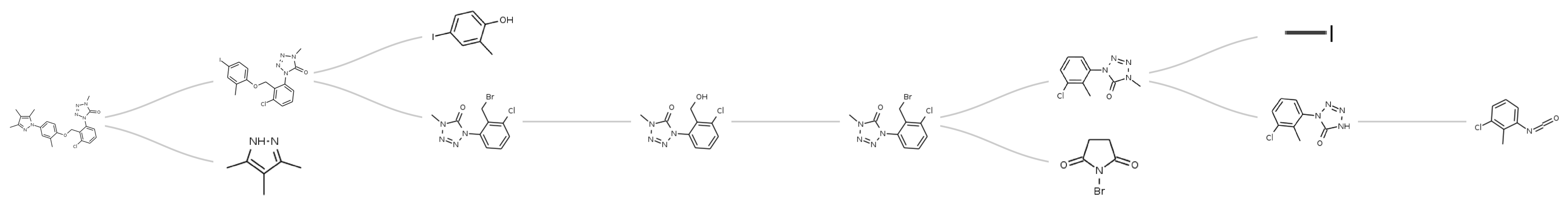

ASKCOS pathway, score: -19.330, ranking: 8, group: 7

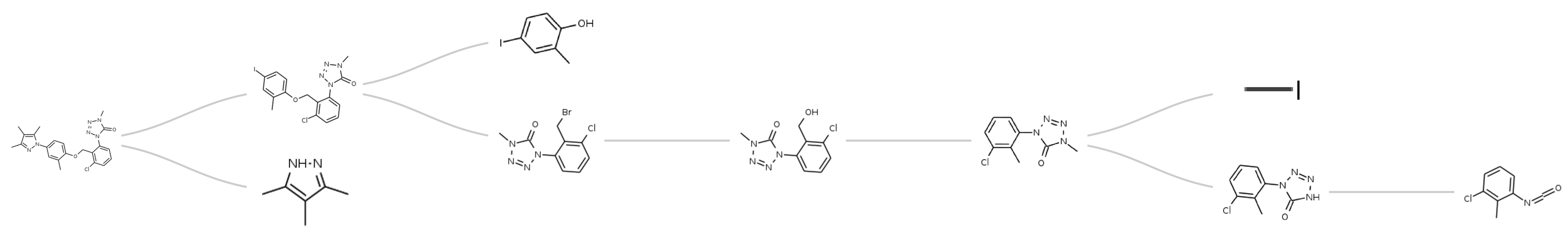

ASKCOS pathway, score: -20.754, ranking: 13, group: 8

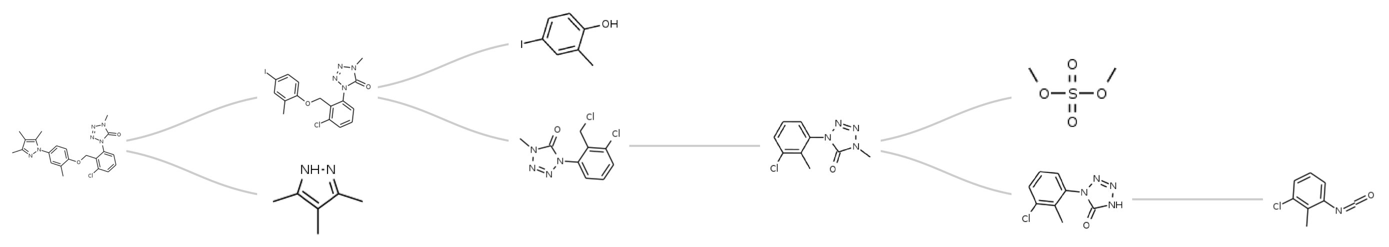

ASKCOS pathway, score: -20.808, ranking: 14, group: 9

Model ranks patent pathway as top-1: Example 33

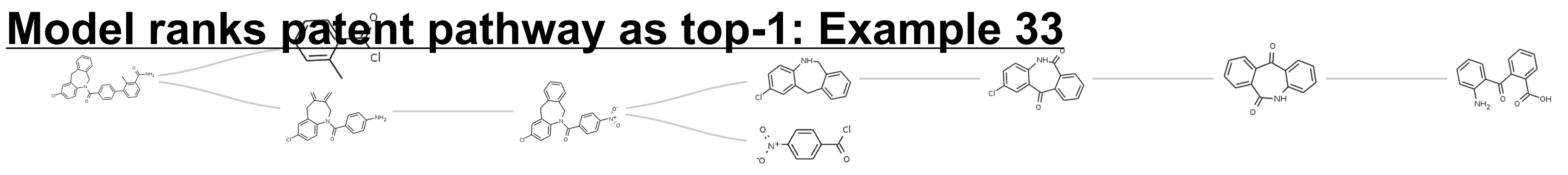

Piatachio pathway, score: -12.247, ranking: 1, Patent No: US05747487A

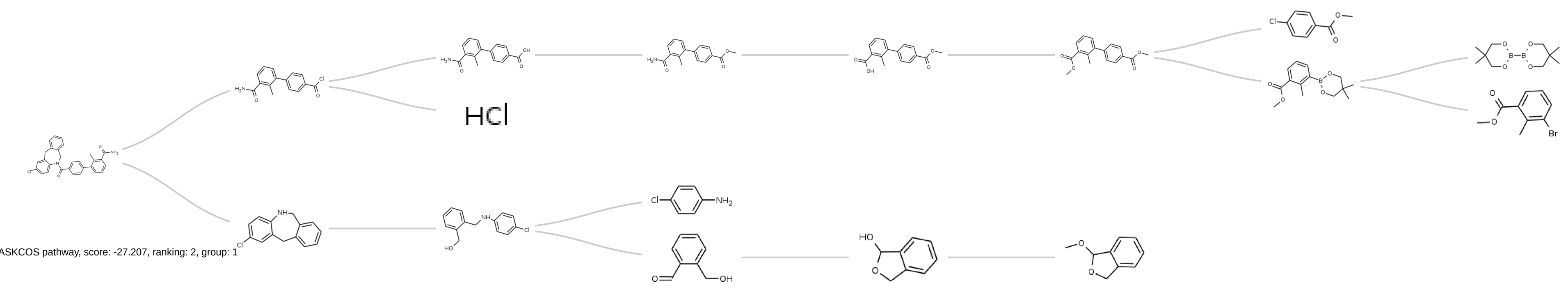

ASKCOS pathway, score: -27.207, ranking: 2, group: 1

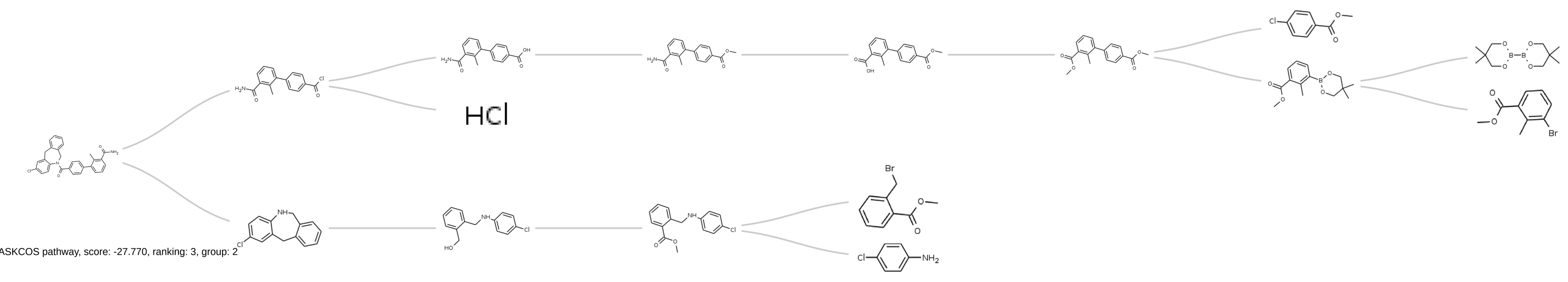

ASKCOS pathway, score: -27.770, ranking: 3, group: 2

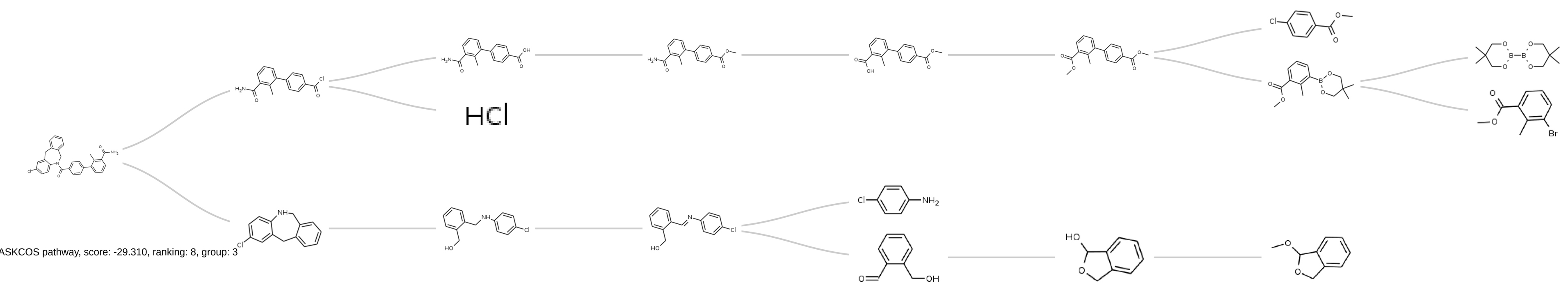

ASKCOS pathway, score: -29.310, ranking: 8, group: 3

Model ranks patent pathway as top-1: Example 34

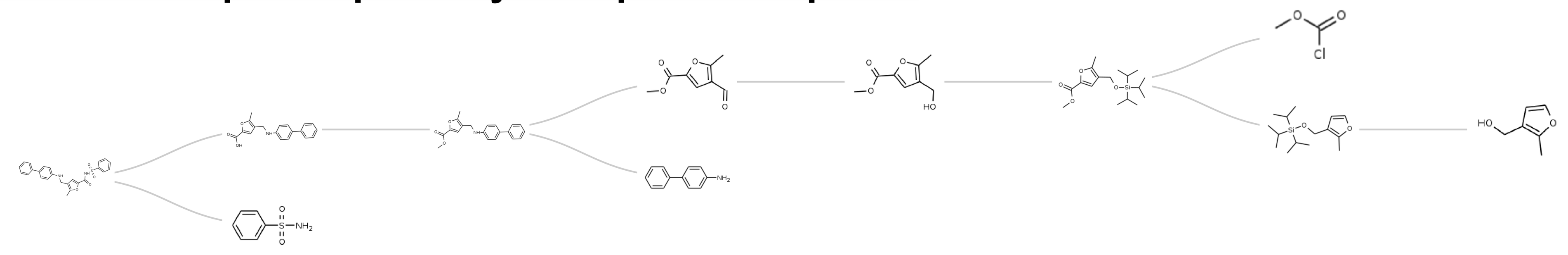

Piatachio pathway, score: -6.173, ranking: 1, Patent No: US07507754B2

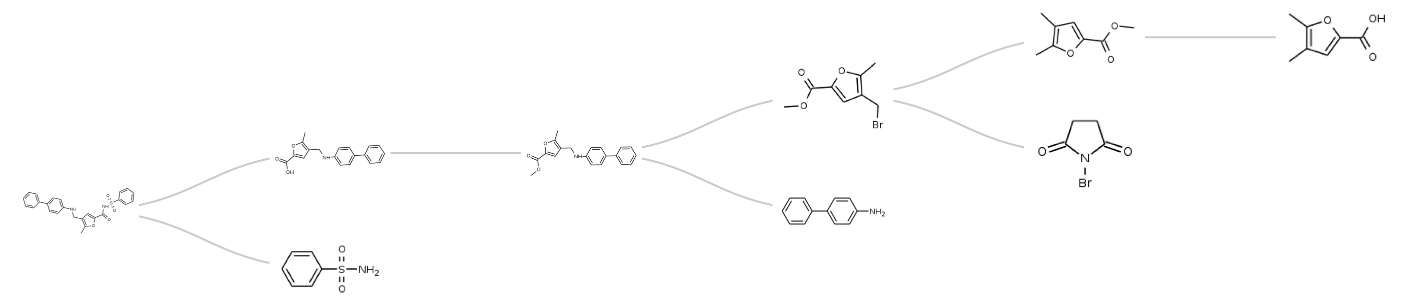

ASKCOS pathway, score: -8.748, ranking: 2, group: 1

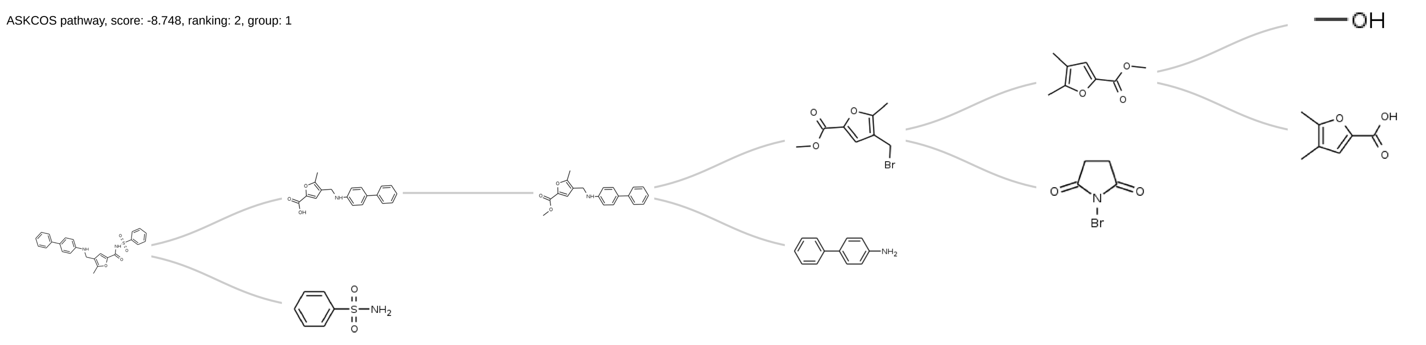

ASKCOS pathway, score: -9.596, ranking: 3, group: 2

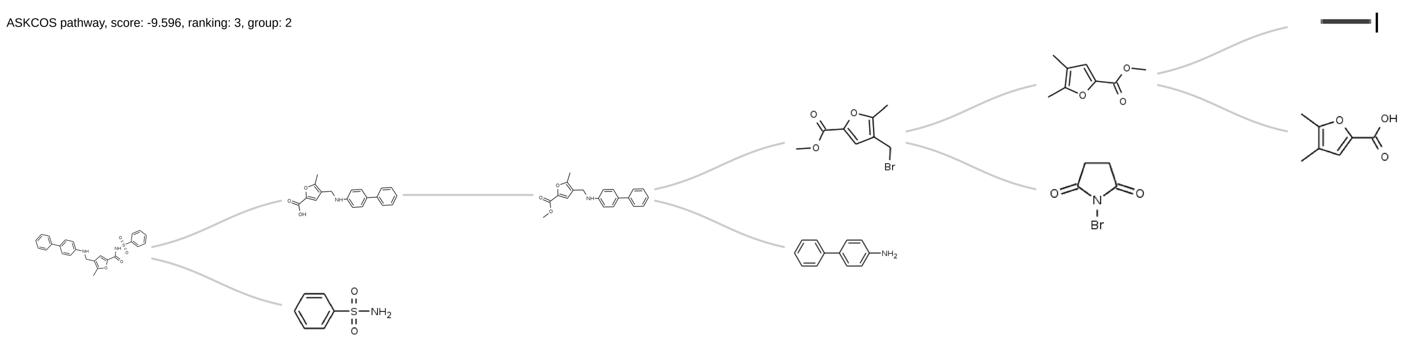

ASKCOS pathway, score: -10.605, ranking: 4, group: 3

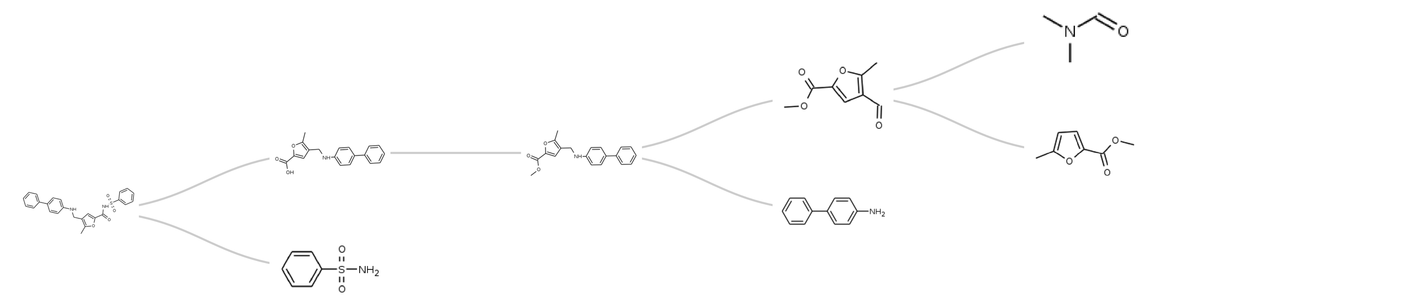

ASKCOS pathway, score: -13.409, ranking: 5, group: 4

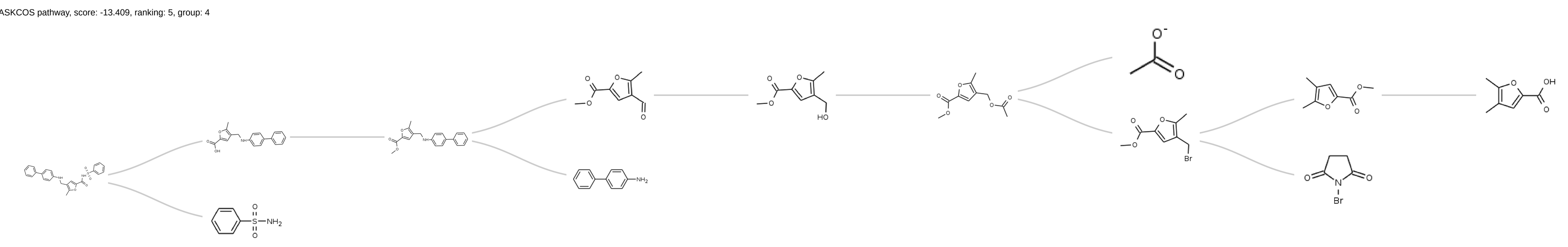

ASKCOS pathway, score: -13.921, ranking: 6, group: 5

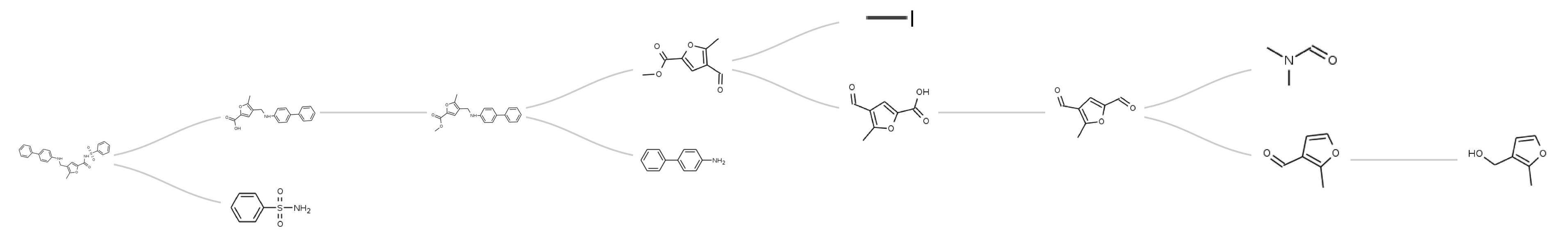

ASKCOS pathway, score: -14.392, ranking: 8, group: 6

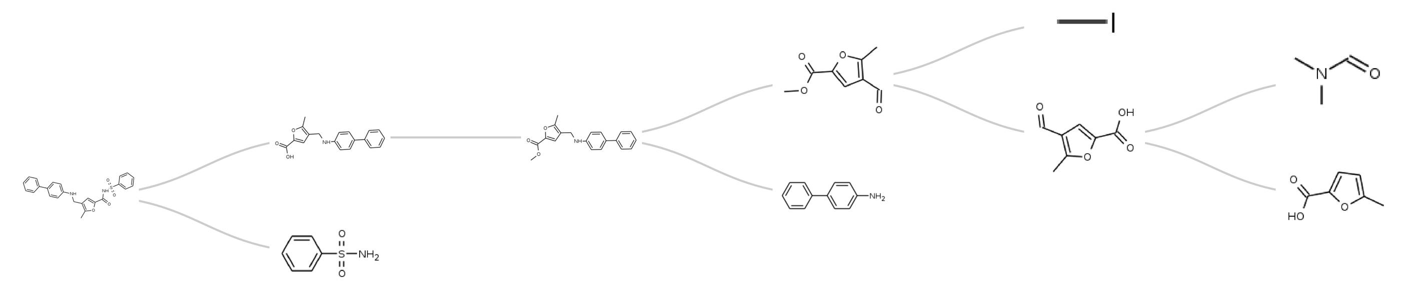

ASKCOS pathway, score: -14.522, ranking: 9, group: 7

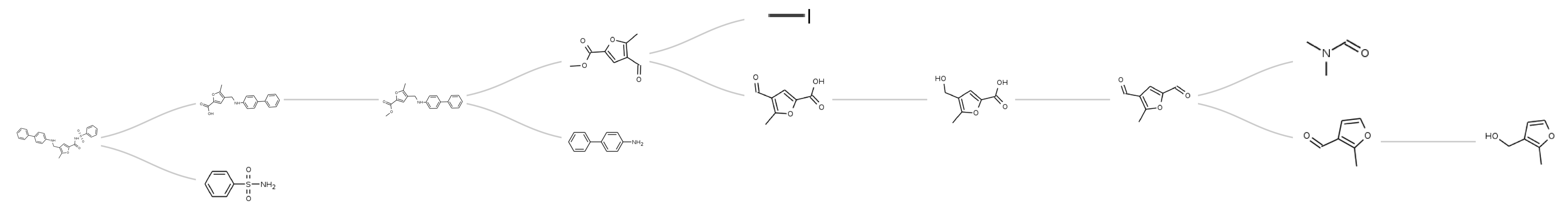

ASKCOS pathway, score: -15.644, ranking: 21, group: 8

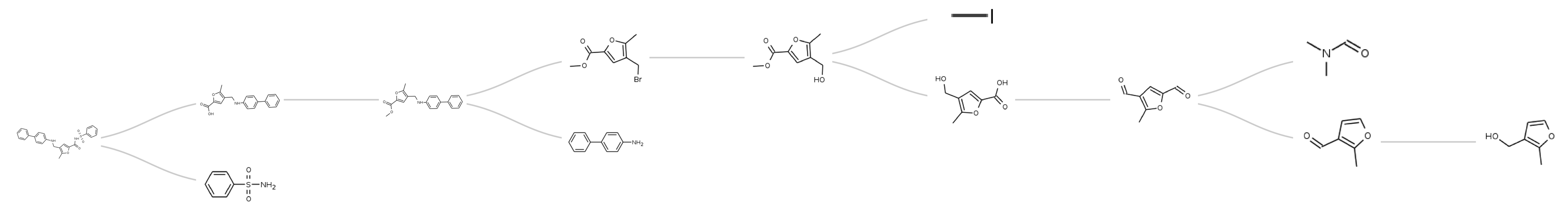

ASKCOS pathway, score: -16.866, ranking: 38, group: 9

Model ranks patent pathway as top-1: Example 35

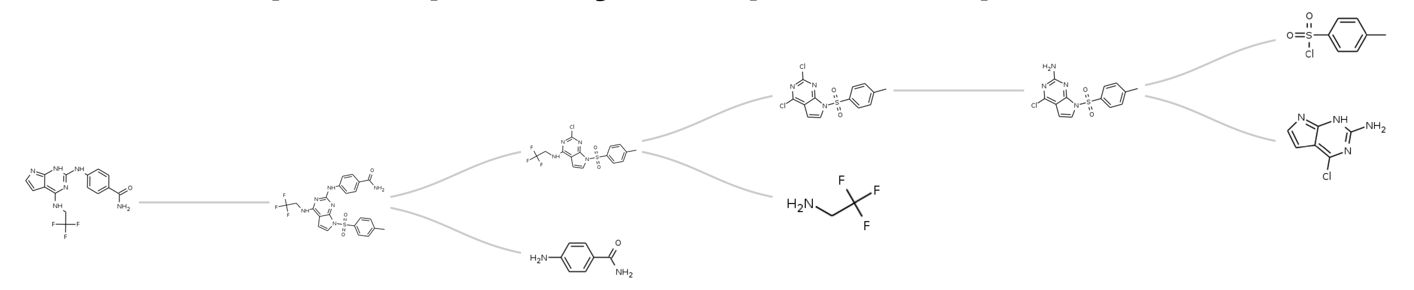

Piatachio pathway, score: -14.450, ranking: 1, Patent No: US20080004295A1

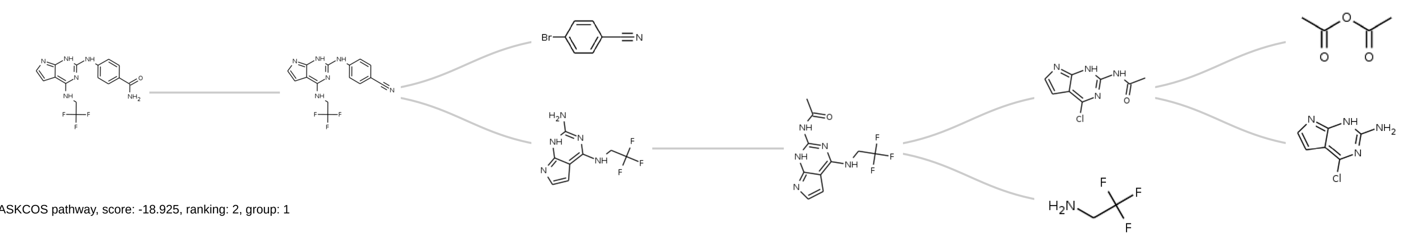

ASKCOS pathway, score: -18.925, ranking: 2, group: 1

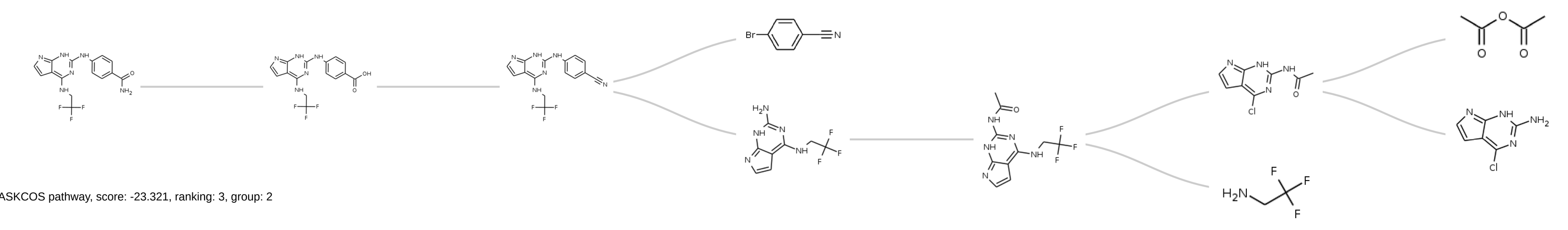

ASKCOS pathway, score: -23.321, ranking: 3, group: 2

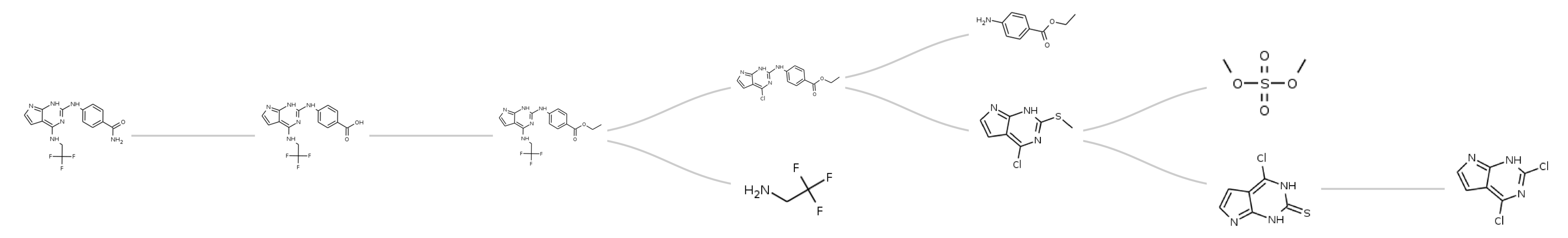

ASKCOS pathway, score: -27.163, ranking: 4, group: 3

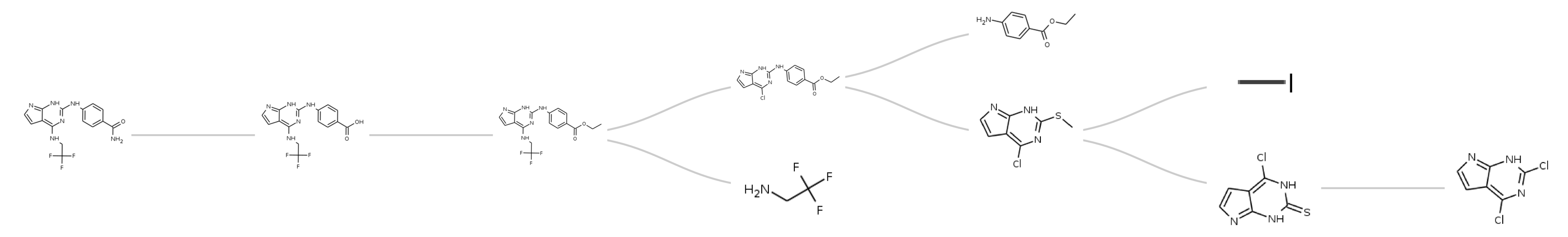

ASKCOS pathway, score: -27.191, ranking: 5, group: 4

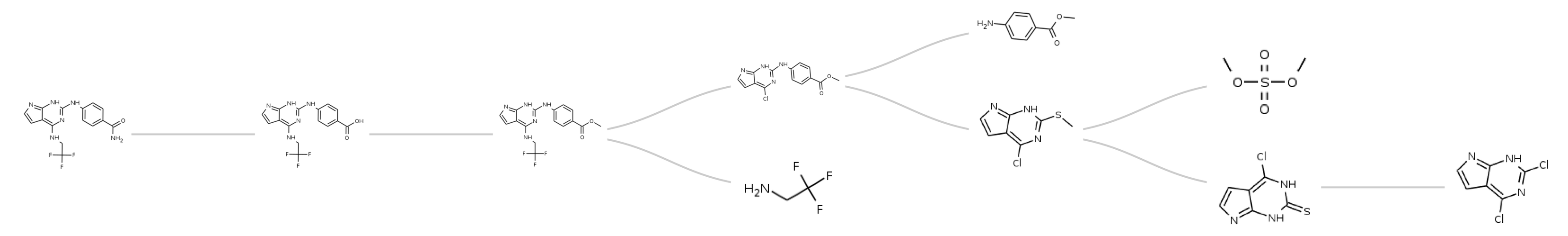

ASKCOS pathway, score: -27.532, ranking: 6, group: 5

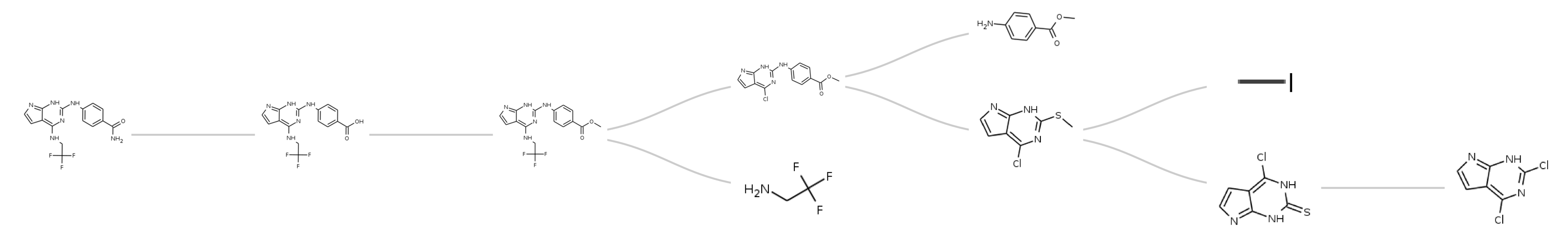

ASKCOS pathway, score: -27.636, ranking: 7, group: 6

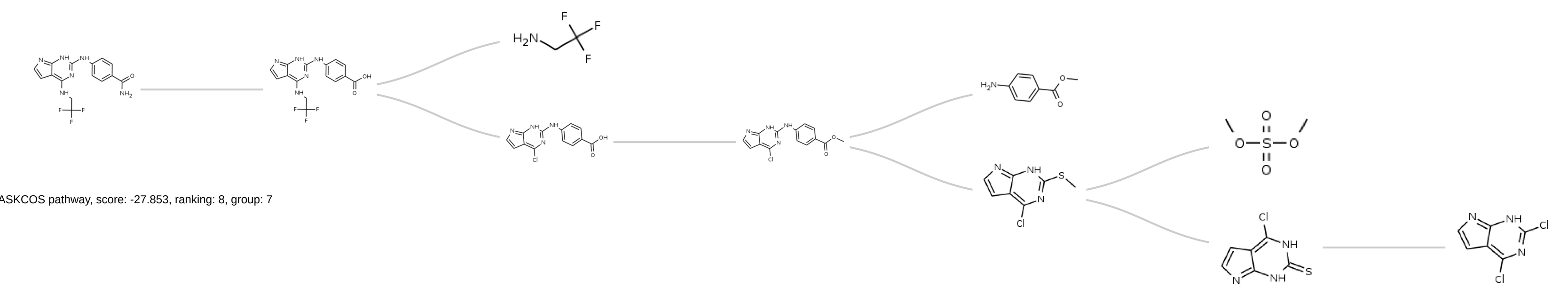

ASKCOS pathway, score: -27.853, ranking: 8, group: 7

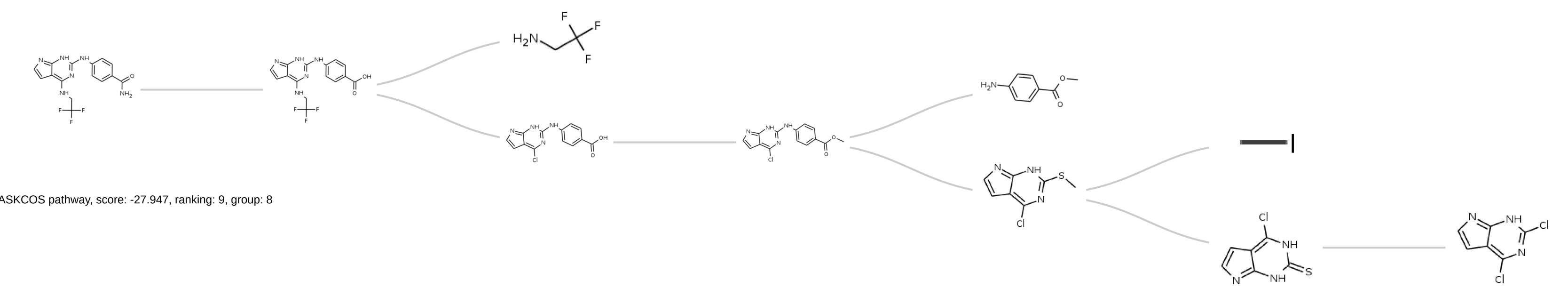

ASKCOS pathway, score: -27.947, ranking: 9, group: 8

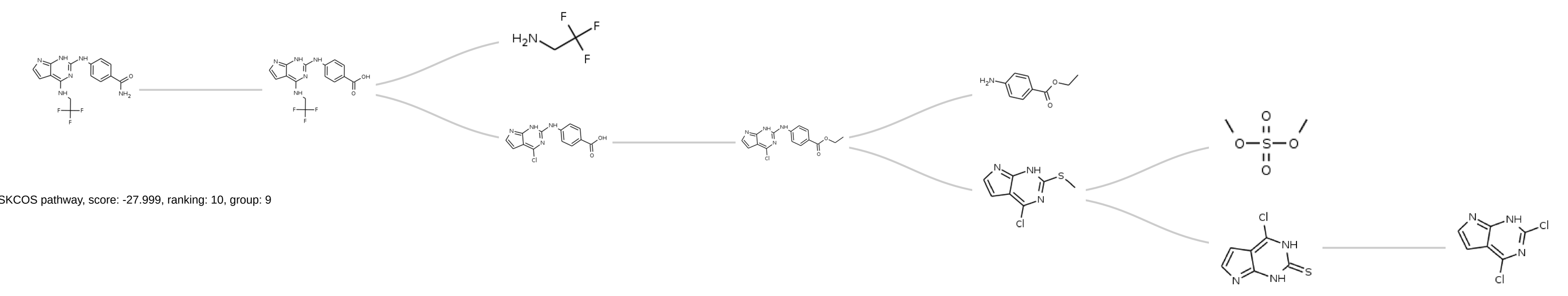

ASKCOS pathway, score: -27.999, ranking: 10, group: 9

Model ranks patent pathway as top-1: Example 36

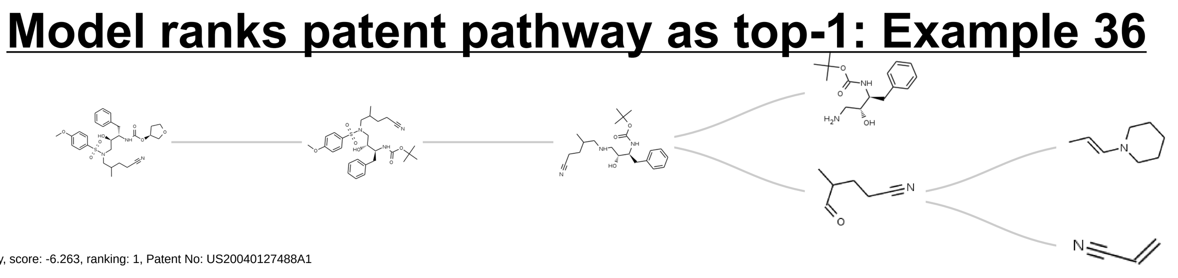

Piatachio pathway, score: -6.263, ranking: 1, Patent No: US20040127488A1

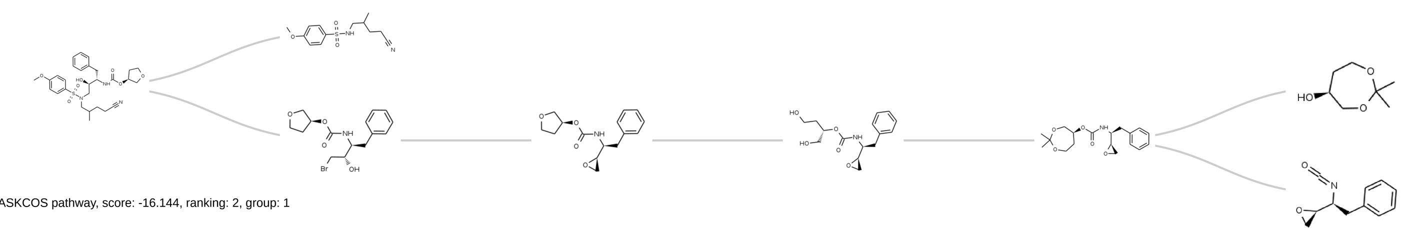

ASKCOS pathway, score: -16.144, ranking: 2, group: 1

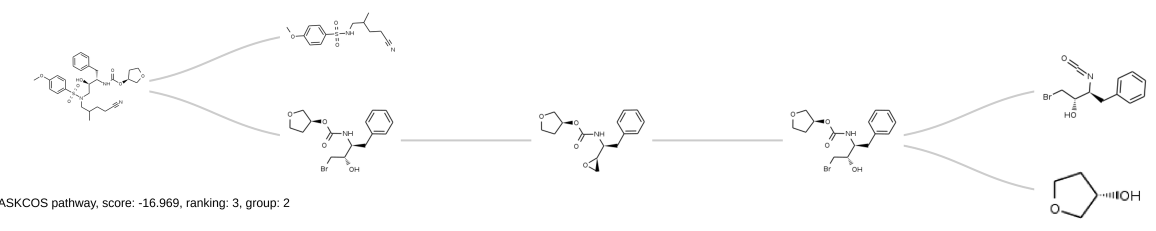

ASKCOS pathway, score: -16.969, ranking: 3, group: 2

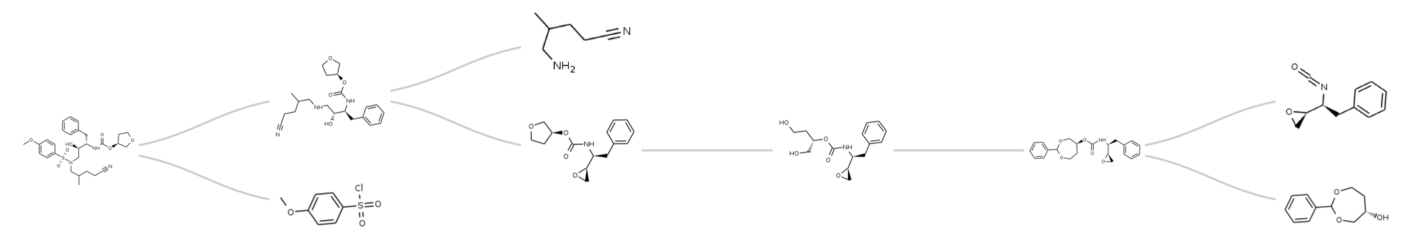

ASKCOS pathway, score: -17.381, ranking: 4, group: 3

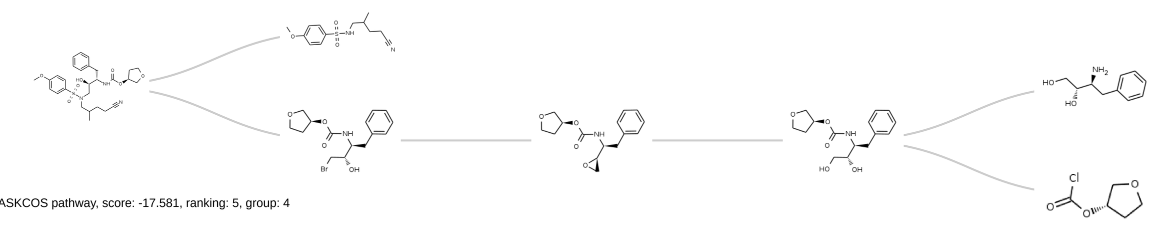

ASKCOS pathway, score: -17.581, ranking: 5, group: 4

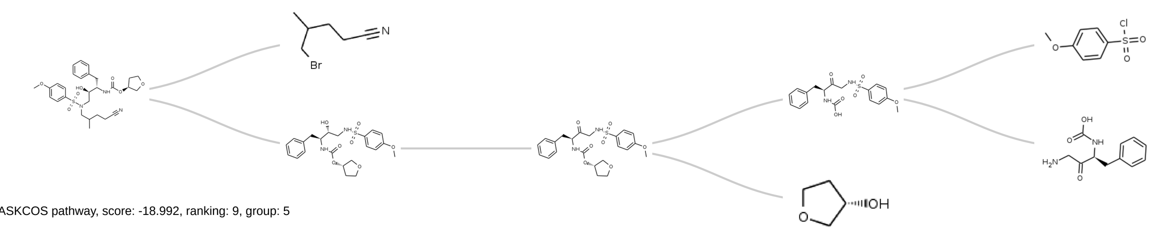

ASKCOS pathway, score: -18.992, ranking: 9, group: 5

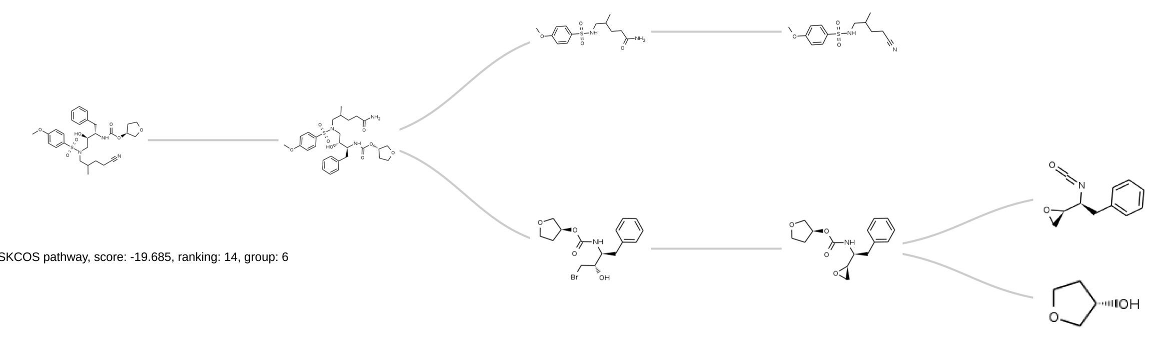

ASKCOS pathway, score: -19.685, ranking: 14, group: 6

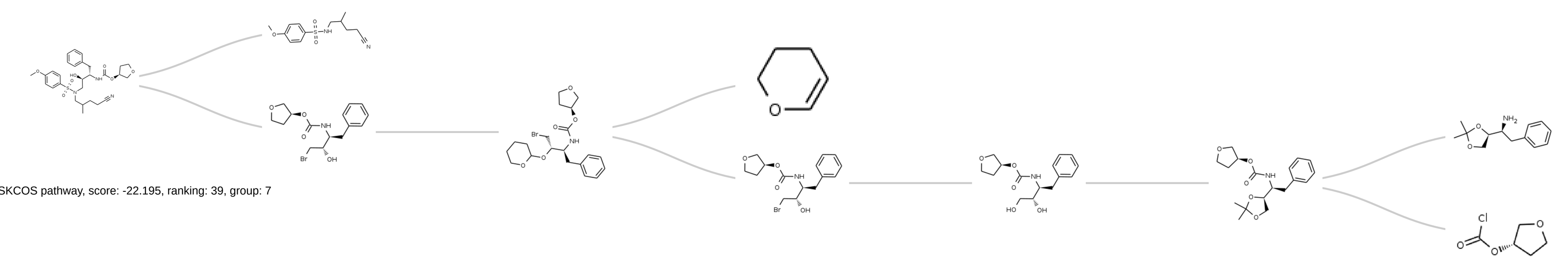

ASKCOS pathway, score: -22.195, ranking: 39, group: 7

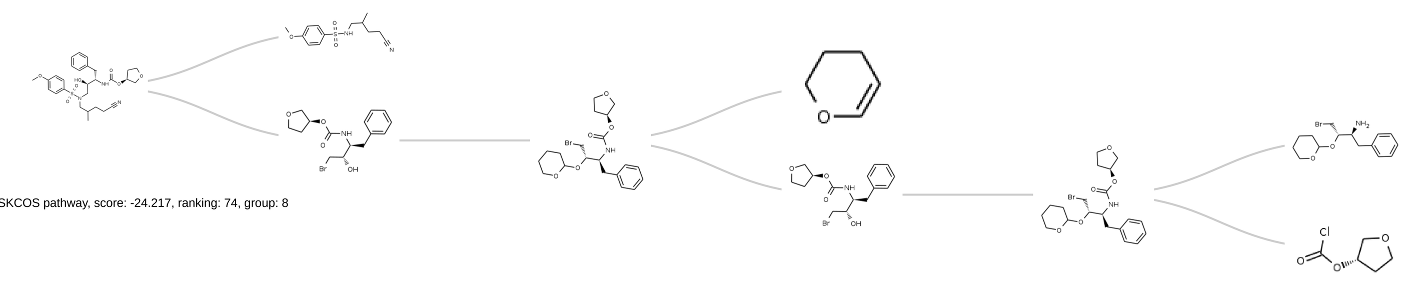

ASKCOS pathway, score: -24.217, ranking: 74, group: 8

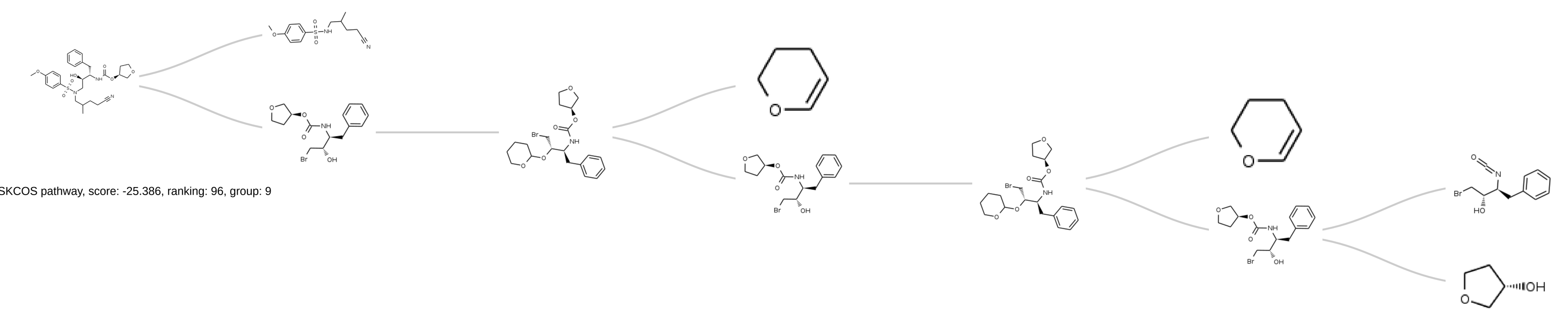

ASKCOS pathway, score: -25.386, ranking: 96, group: 9

Model ranks patent pathway as top-1: Example 37

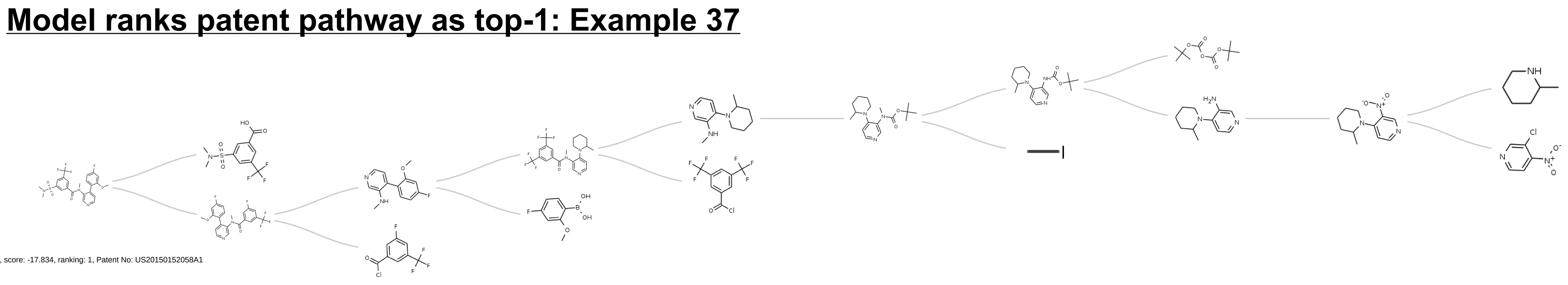

Piatachio pathway, score: -17.834, ranking: 1, Patent No: US20150152058A1

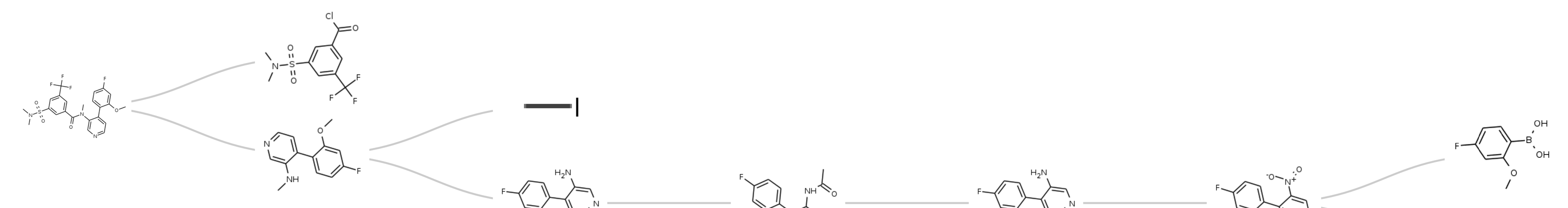

ASKCOS pathway, score: -21.733, ranking: 2, group: 1

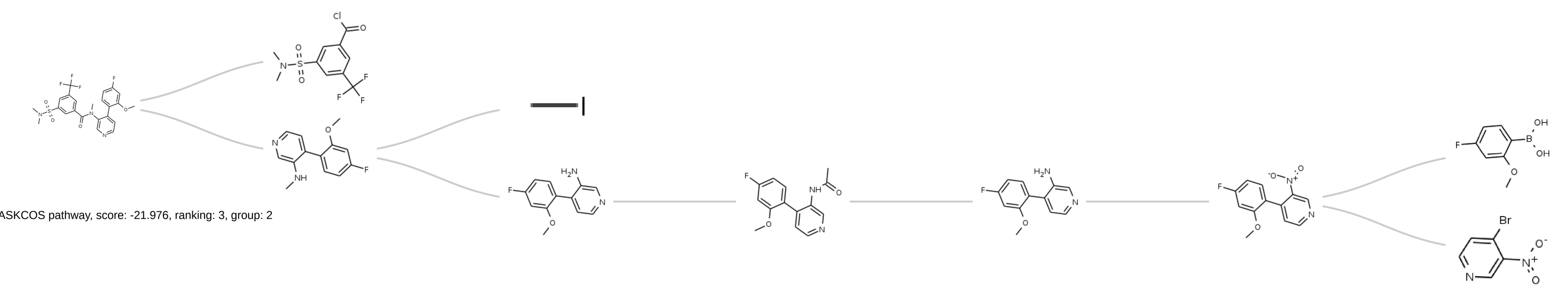

ASKCOS pathway, score: -21.976, ranking: 3, group: 2

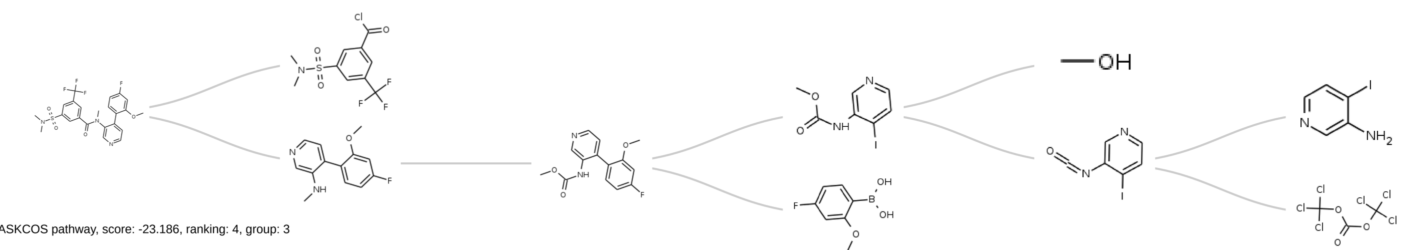

ASKCOS pathway, score: -23.186, ranking: 4, group: 3

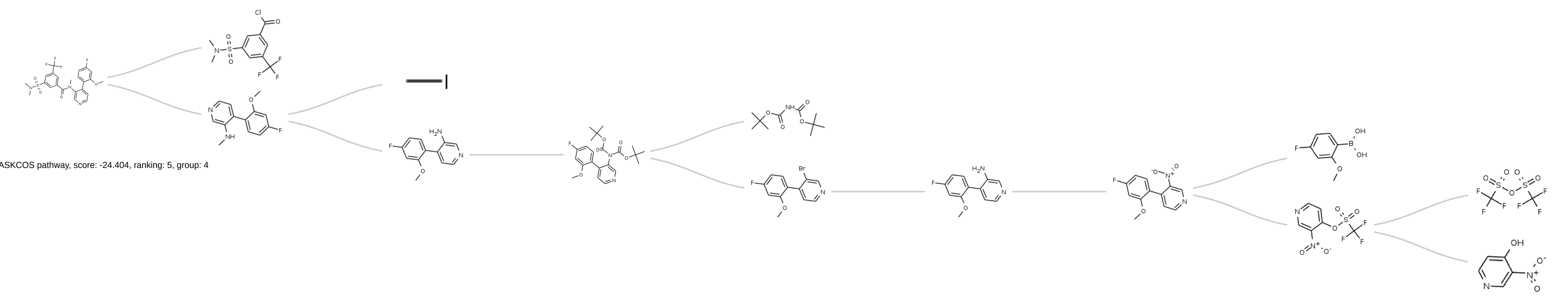

ASKCOS pathway, score: -24.404, ranking: 5, group: 4

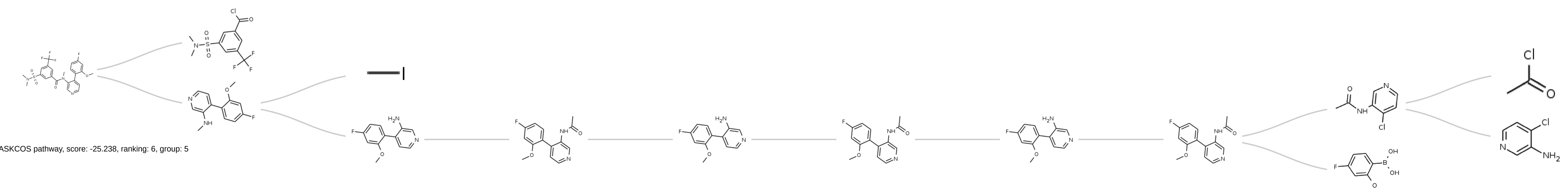

ASKCOS pathway, score: -25.238, ranking: 6, group: 5

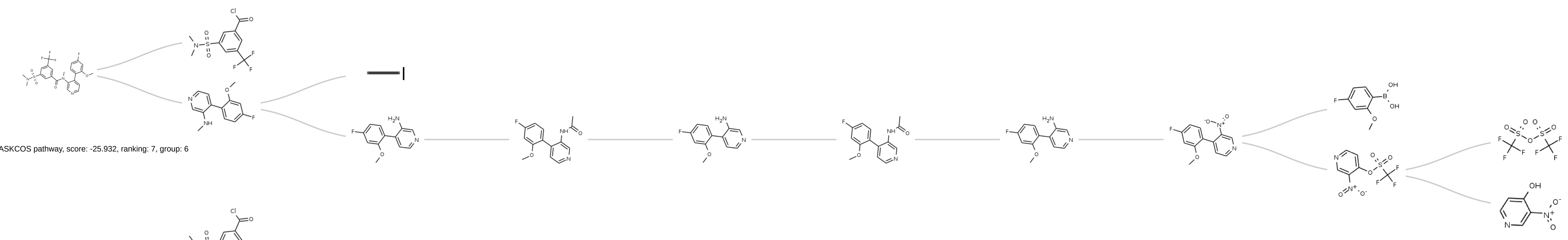

ASKCOS pathway, score: -25.832, ranking: 7, group: 6

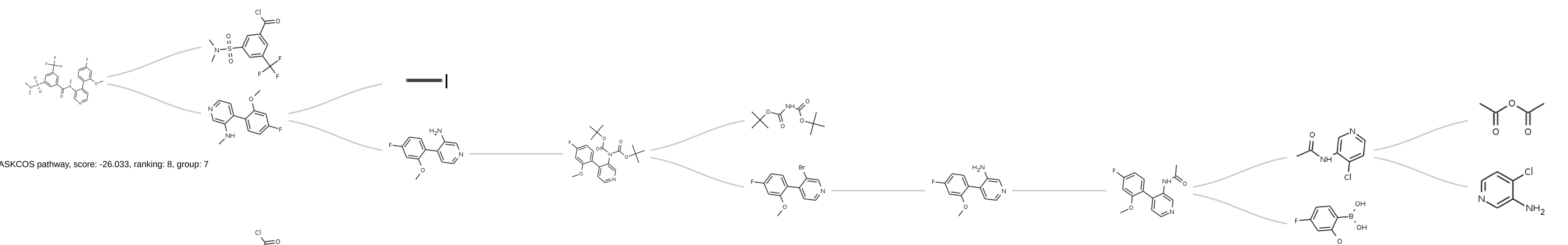

ASKCOS pathway, score: -26.033, ranking: 8, group: 7

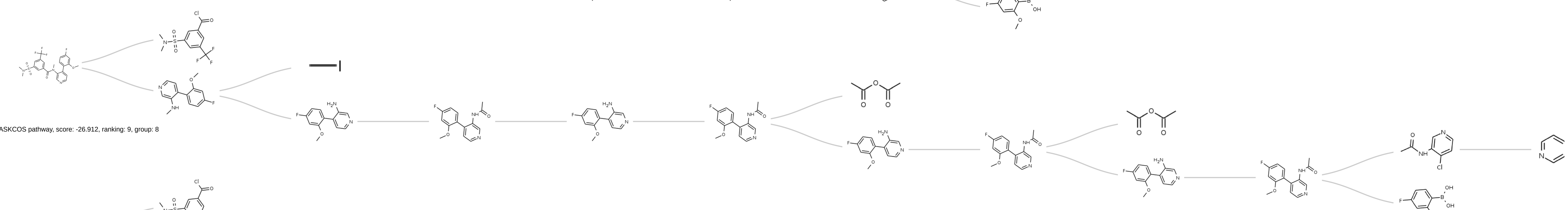

ASKCOS pathway, score: -26.912, ranking: 9, group: 8

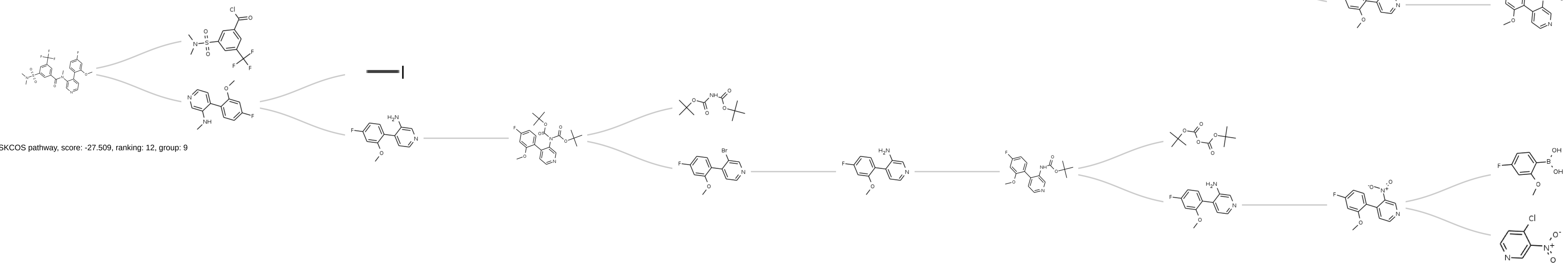

ASKCOS pathway, score: -27.509, ranking: 12, group: 9

Model ranks patent pathway as top-1: Example 38

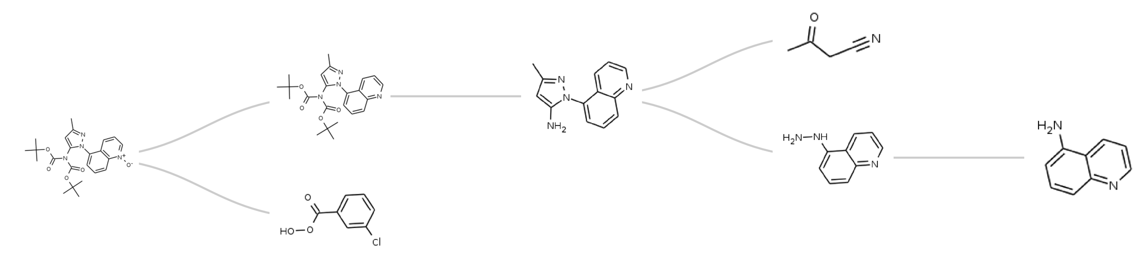

Piatachio pathway, score: -1.413, ranking: 1, Patent No: US20180009797A1

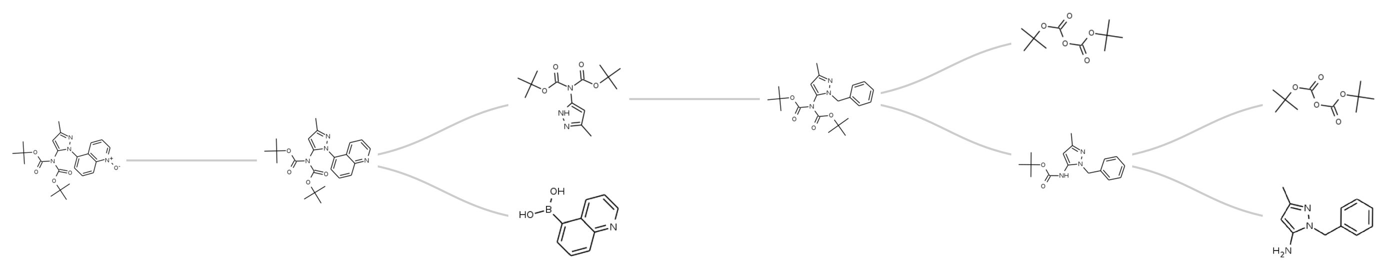

ASKCOS pathway, score: -14.916, ranking: 2, group: 1

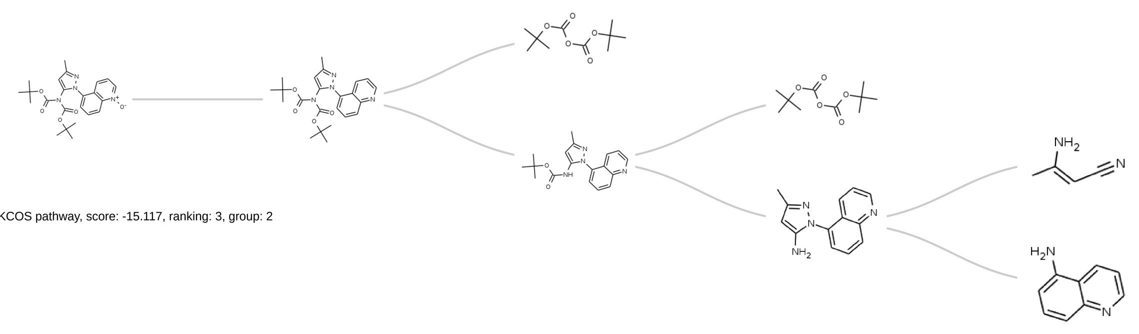

ASKCOS pathway, score: -15.117, ranking: 3, group: 2

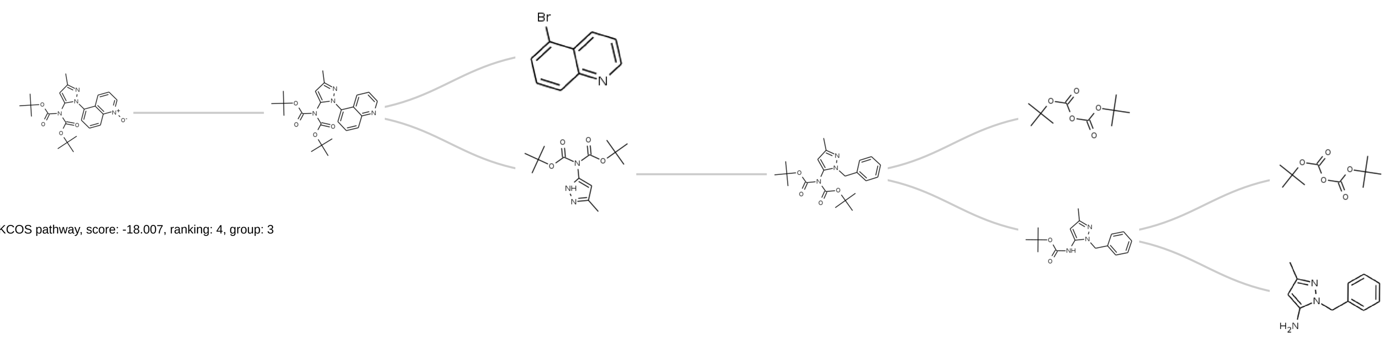

ASKCOS pathway, score: -18.007, ranking: 4, group: 3

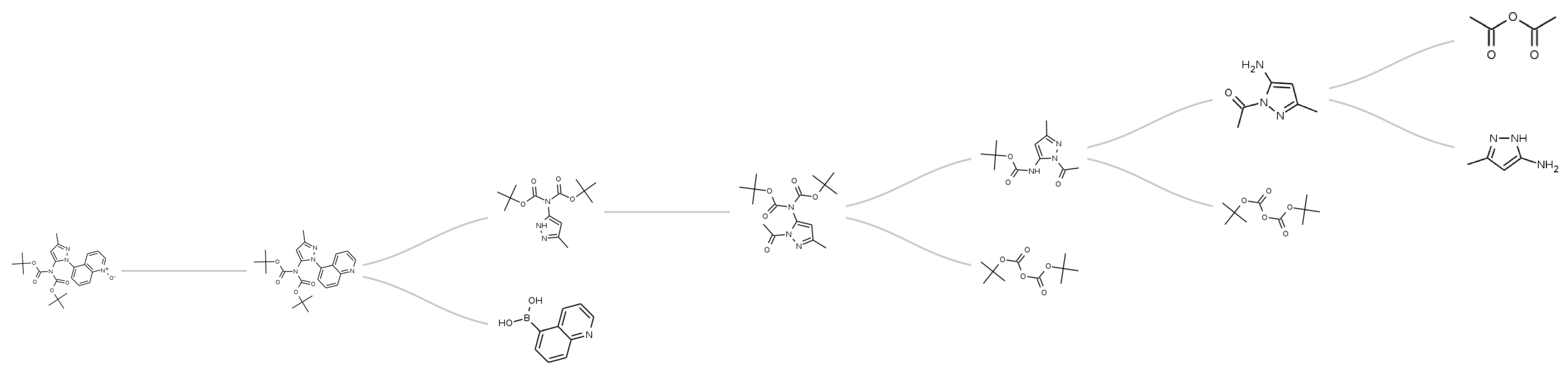

ASKCOS pathway, score: -18.241, ranking: 5, group: 4

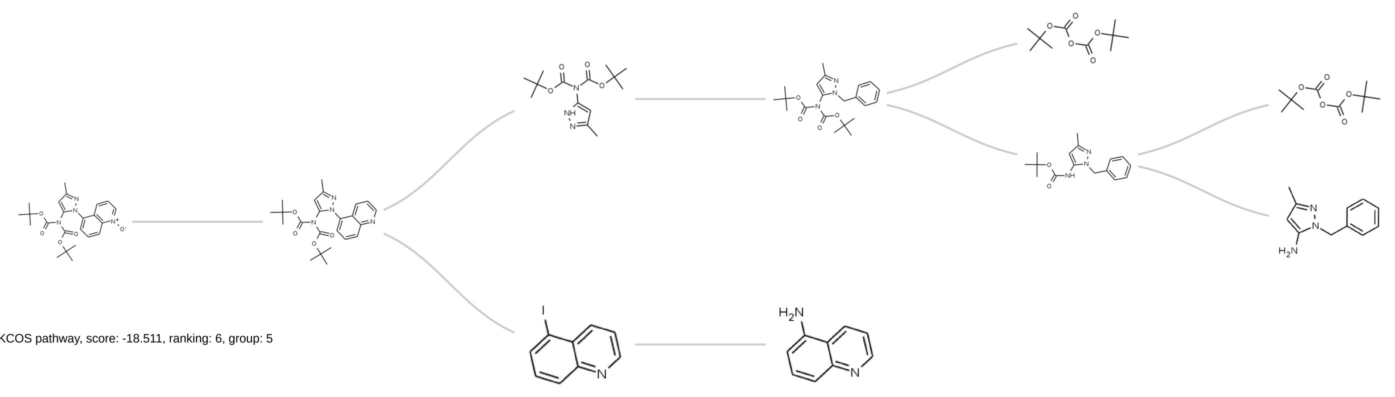

ASKCOS pathway, score: -18.511, ranking: 6, group: 5

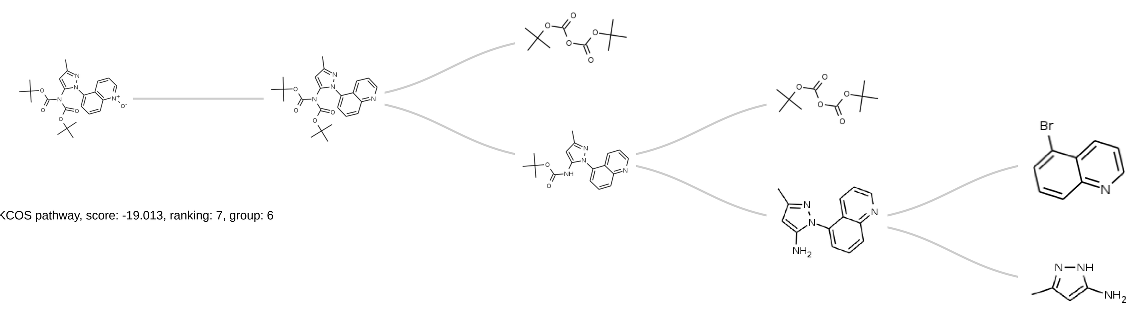

ASKCOS pathway, score: -19.013, ranking: 7, group: 6

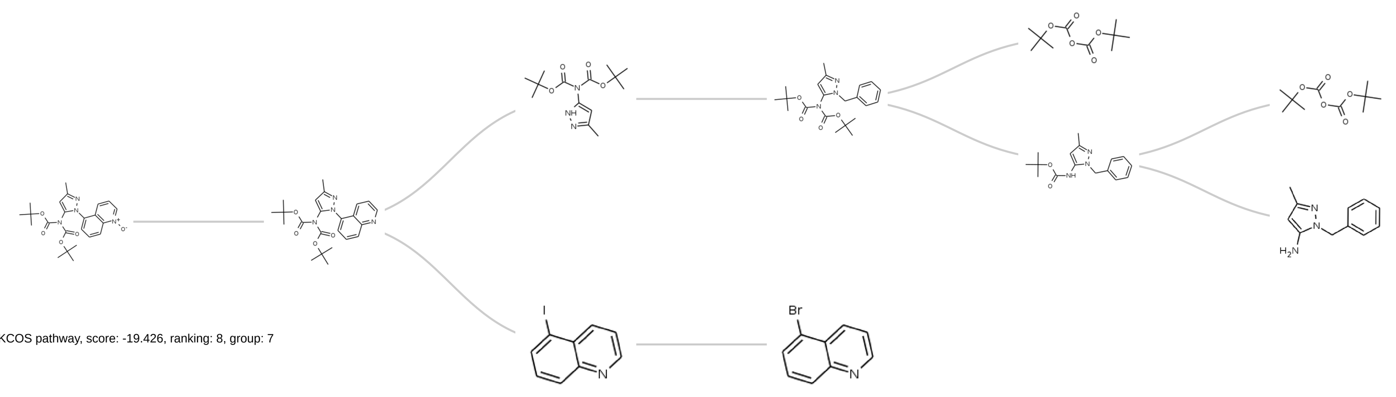

ASKCOS pathway, score: -19.426, ranking: 8, group: 7

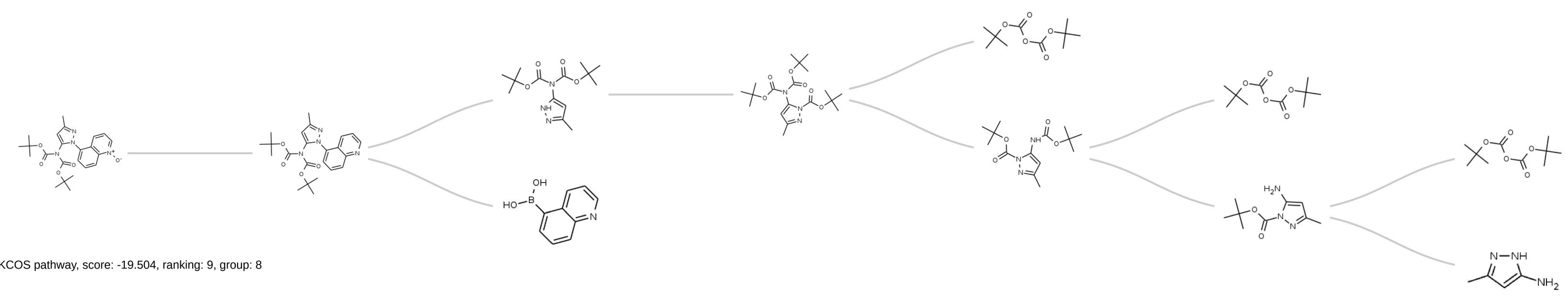

ASKCOS pathway, score: -19.504, ranking: 9, group: 8

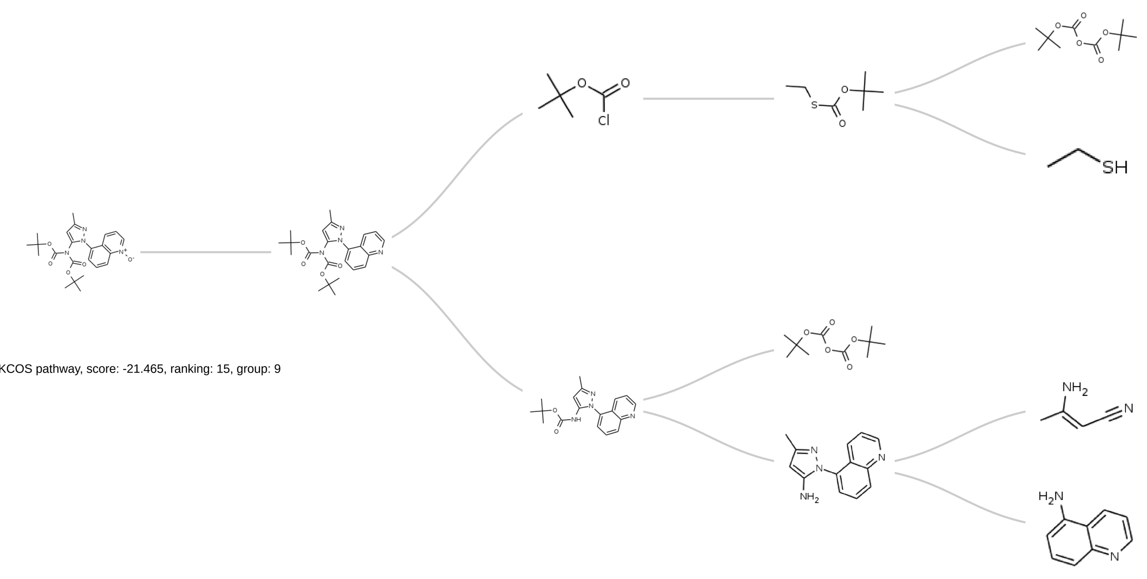

ASKCOS pathway, score: -21.465, ranking: 15, group: 9

Model ranks patent pathway as top-1: Example 39

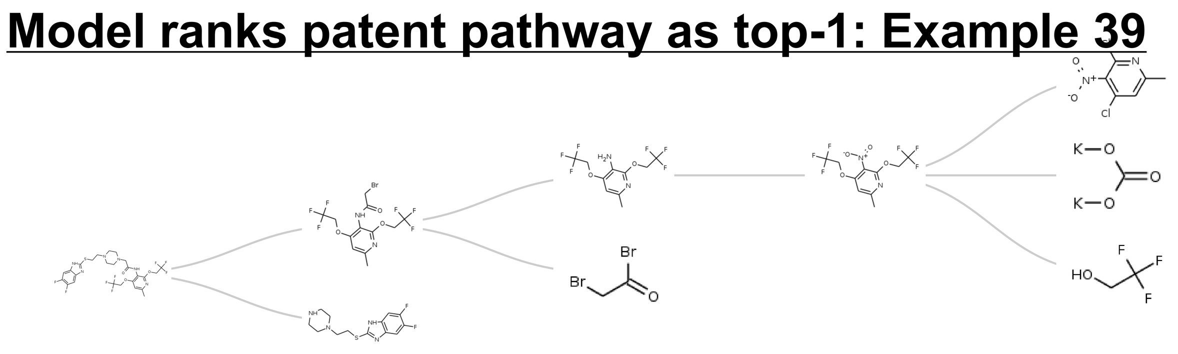

Piatachio pathway, score: -10.069, ranking: 1, Patent No: US20050020606A1

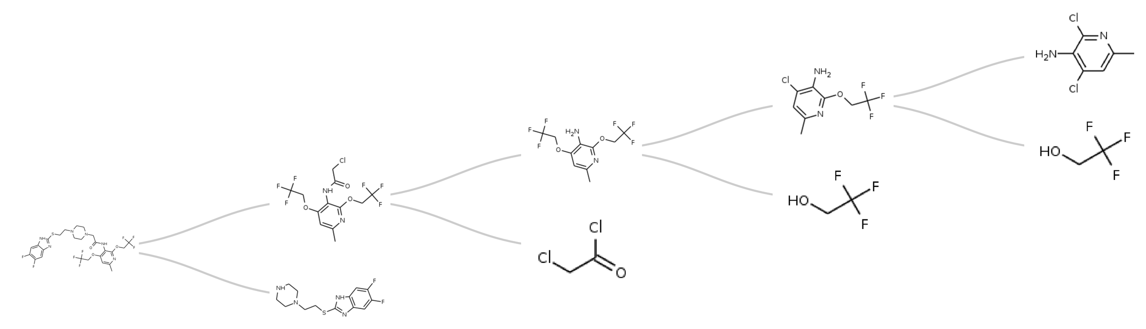

ASKCOS pathway, score: -15.964, ranking: 2, group: 1

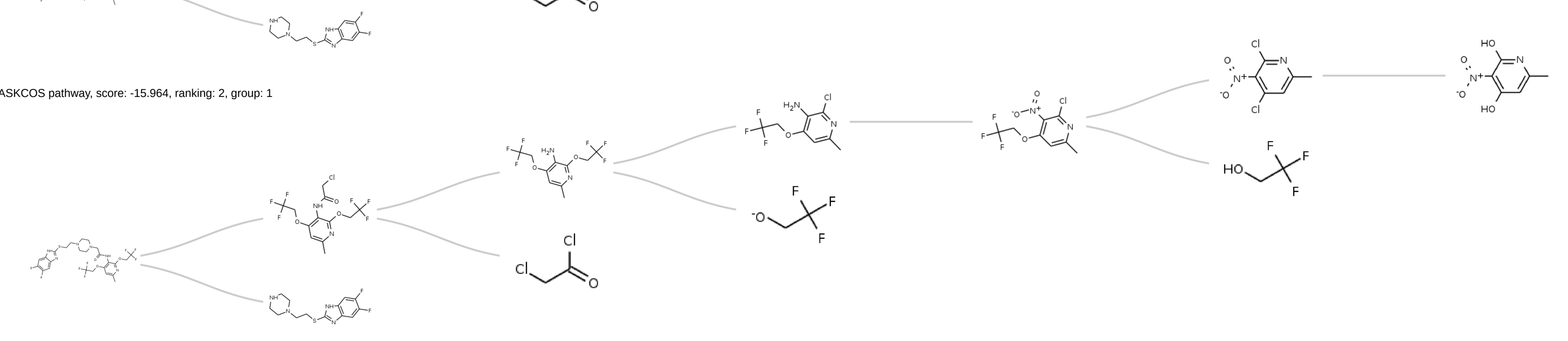

ASKCOS pathway, score: -16.027, ranking: 3, group: 2

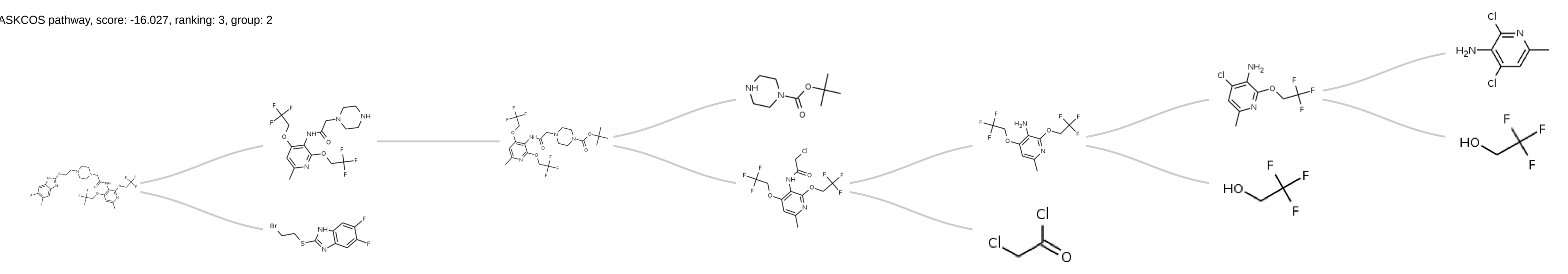

ASKCOS pathway, score: -16.680, ranking: 7, group: 3

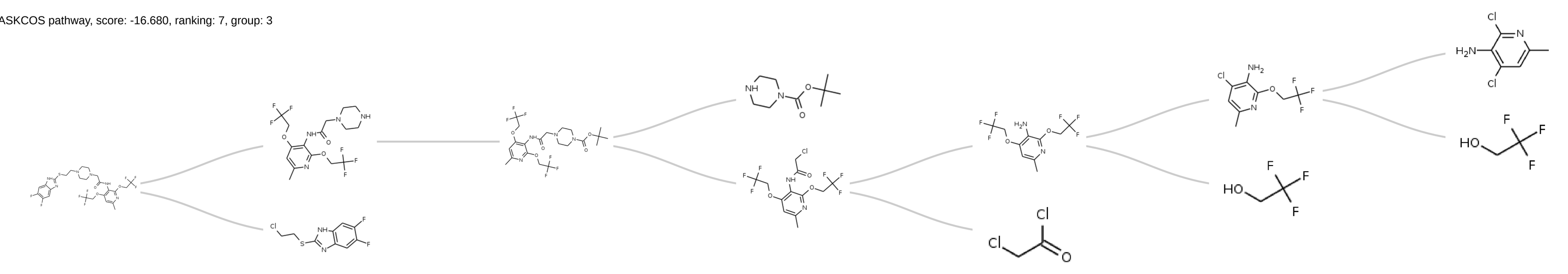

ASKCOS pathway, score: -16.862, ranking: 10, group: 4

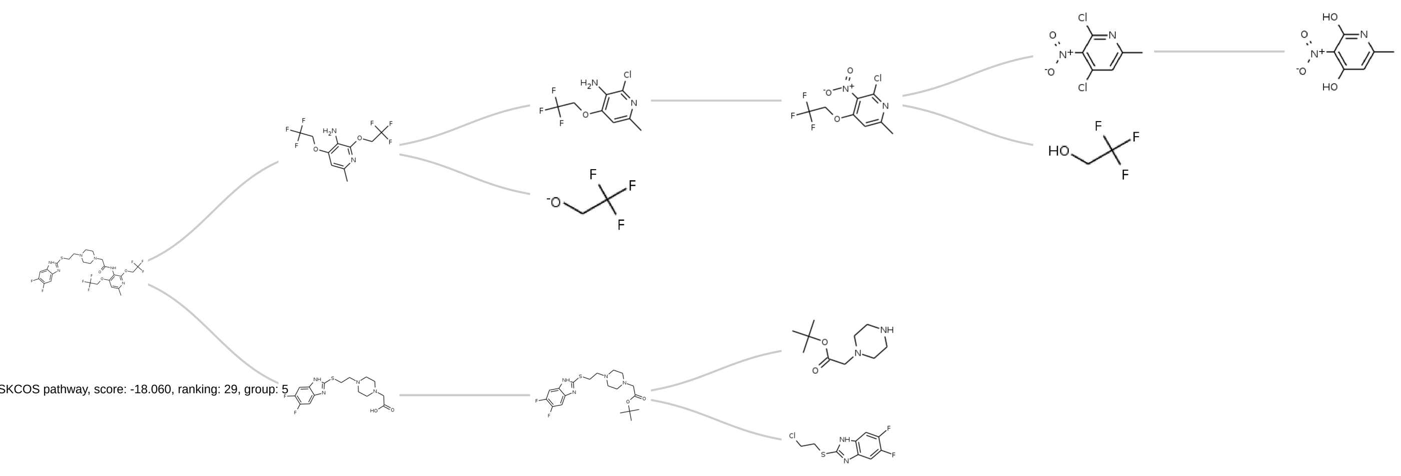

ASKCOS pathway, score: -18.060, ranking: 29, group: 5

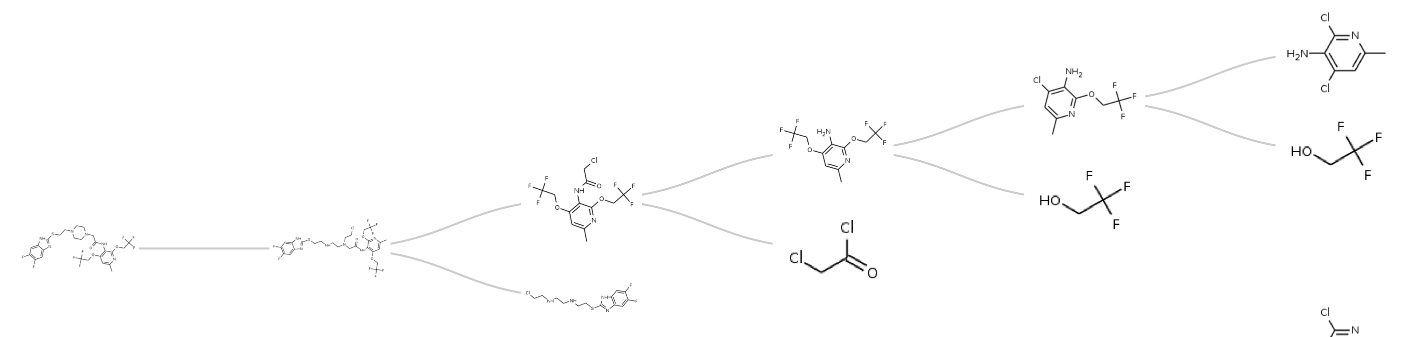

ASKCOS pathway, score: -19.216, ranking: 50, group: 6

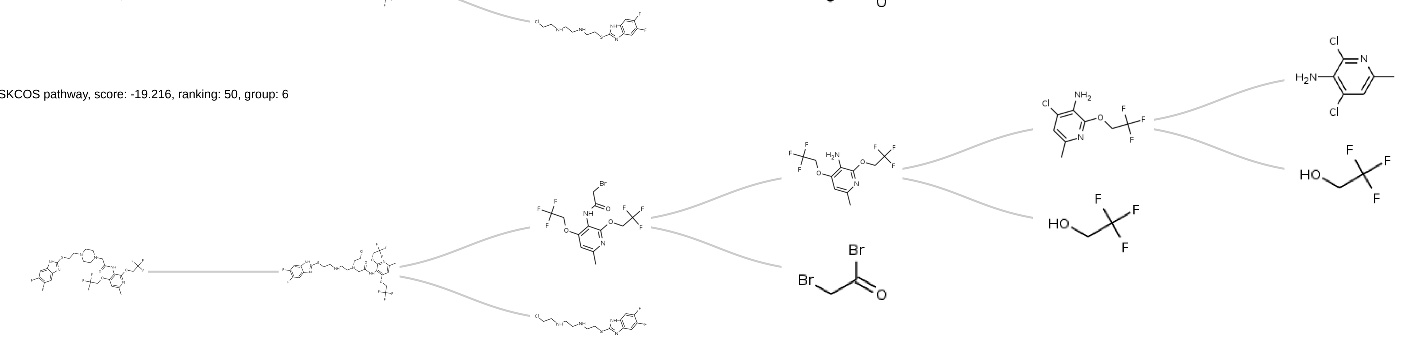

ASKCOS pathway, score: -19.427, ranking: 56, group: 7

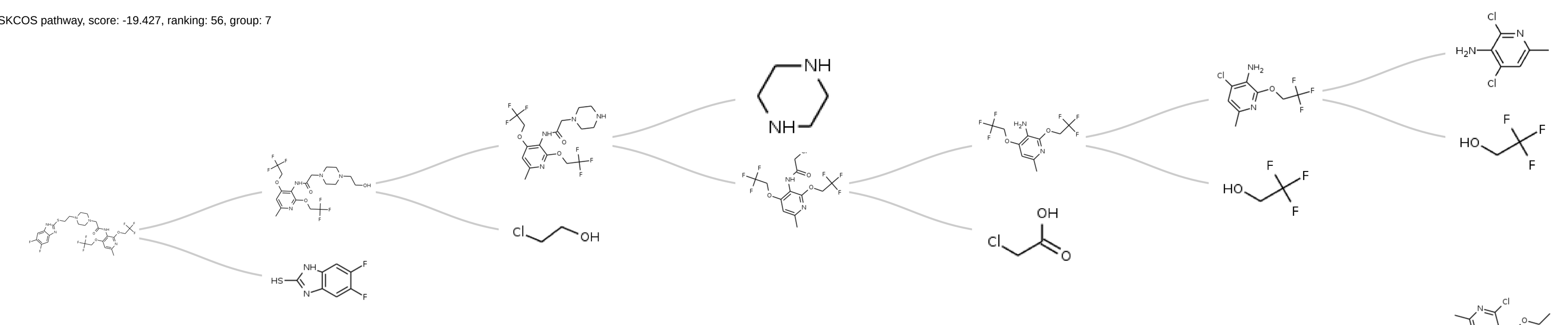

ASKCOS pathway, score: -20.247, ranking: 69, group: 8

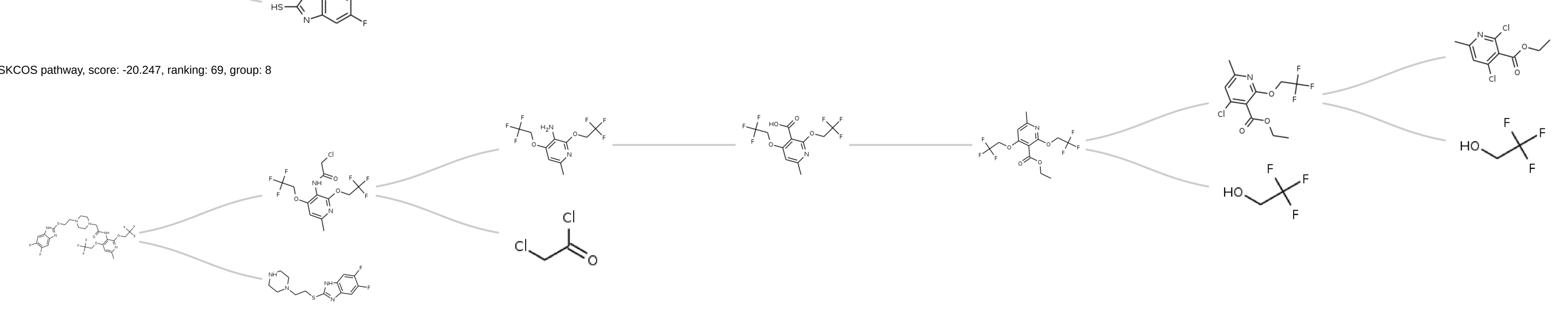

ASKCOS pathway, score: -20.847, ranking: 81, group: 9

Model ranks patent pathway as top-1: Example 40

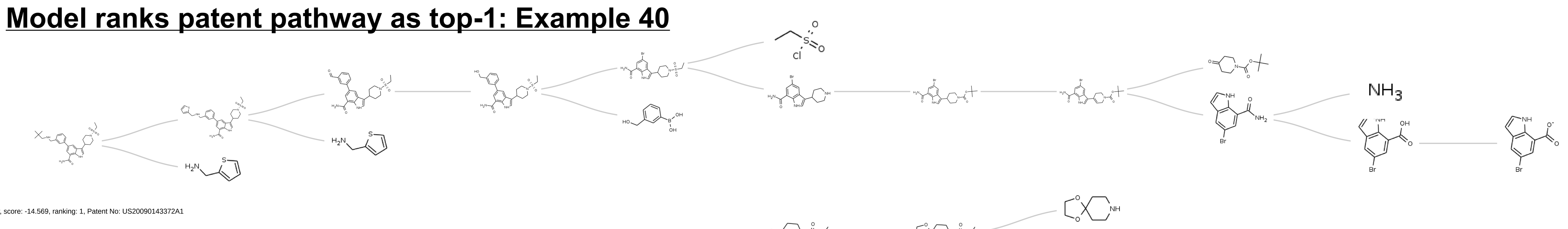

Piatachio pathway, score: -14.569, ranking: 1, Patent No: US20090143372A1

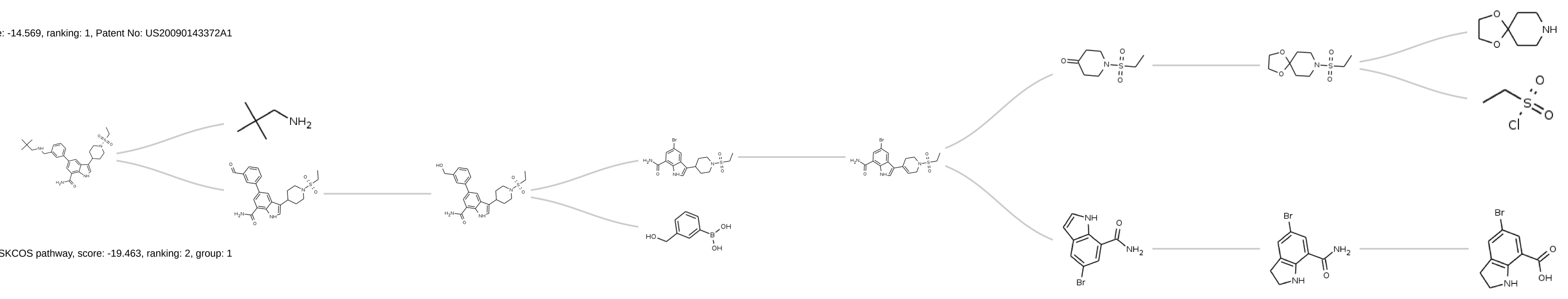

ASKCOS pathway, score: -19.463, ranking: 2, group: 1

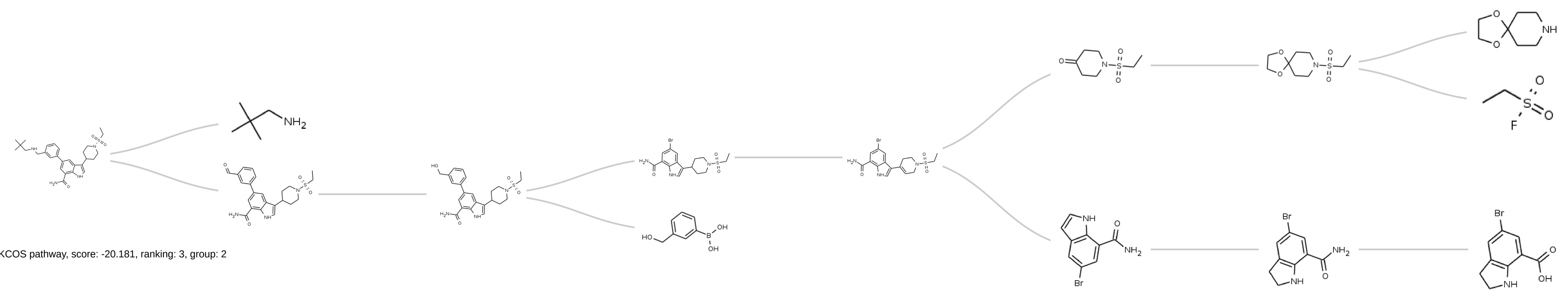

ASKCOS pathway, score: -20.181, ranking: 3, group: 2

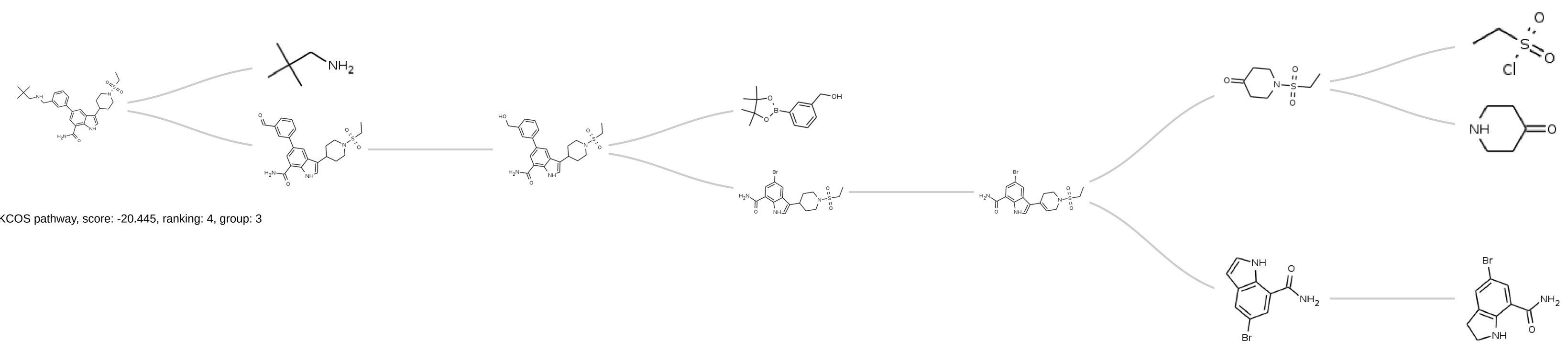

ASKCOS pathway, score: -20.445, ranking: 4, group: 3

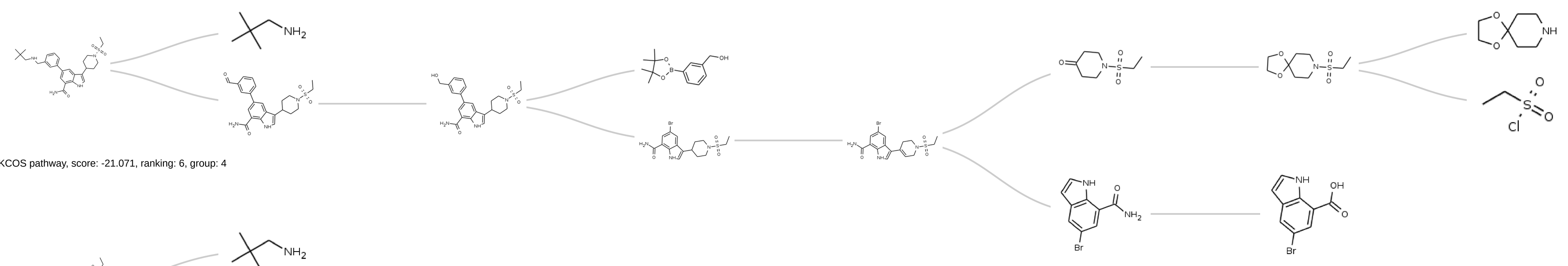

ASKCOS pathway, score: -21.071, ranking: 6, group: 4

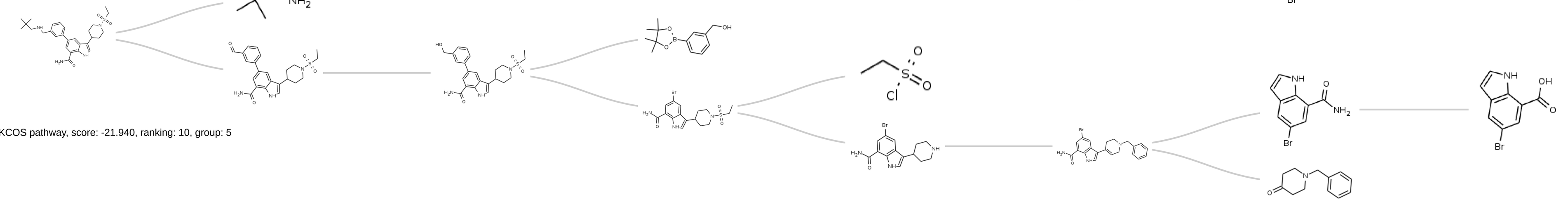

ASKCOS pathway, score: -21.940, ranking: 10, group: 5

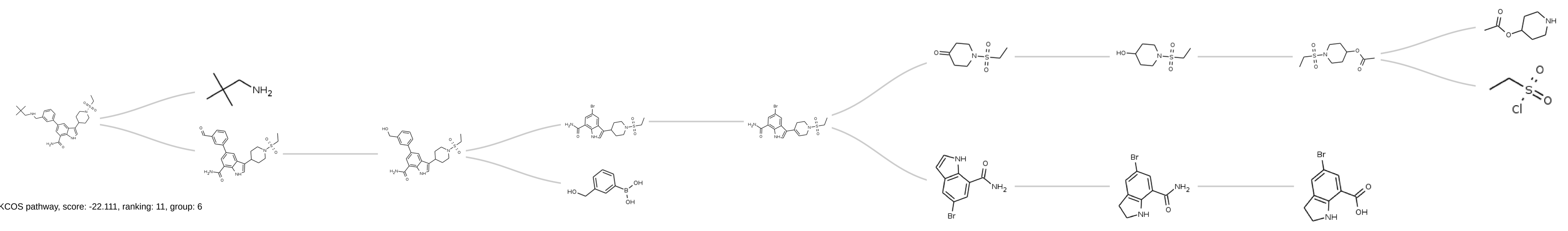

ASKCOS pathway, score: -22.111, ranking: 11, group: 6

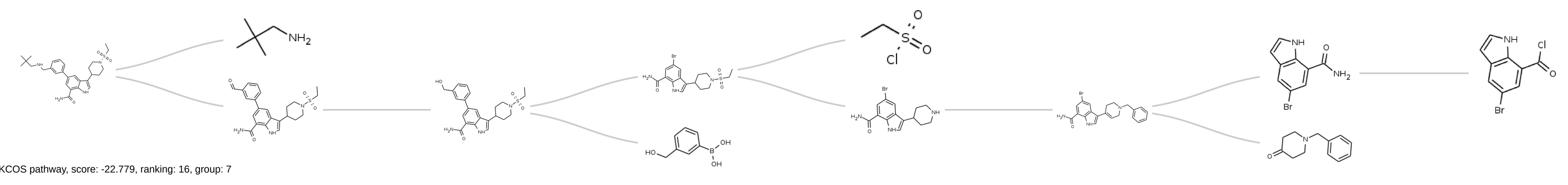

ASKCOS pathway, score: -22.779, ranking: 16, group: 7

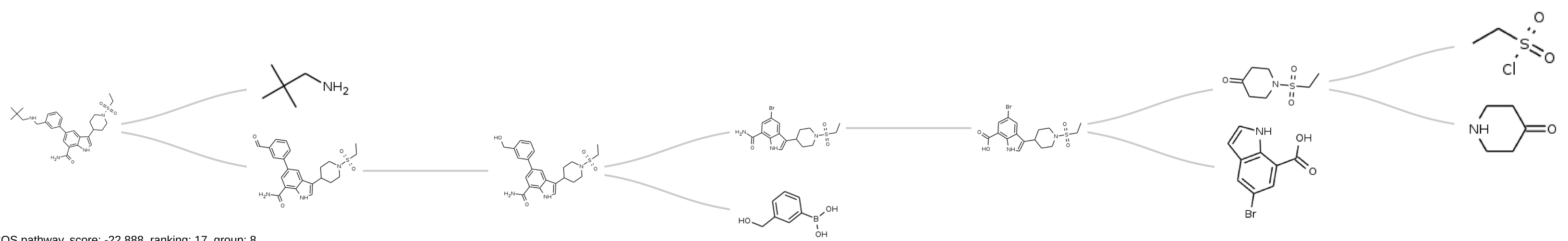

ASKCOS pathway, score: -22.888, ranking: 17, group: 8

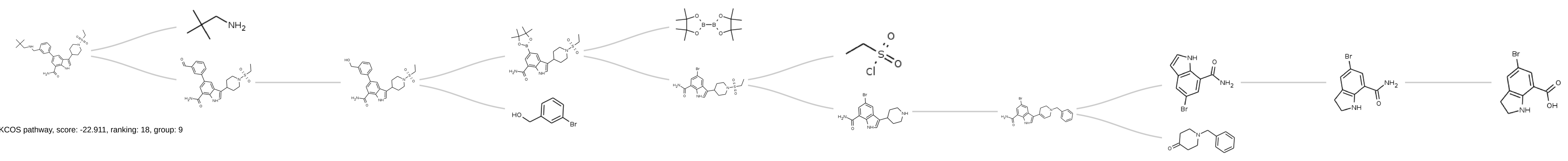

ASKCOS pathway, score: -22.911, ranking: 18, group: 9

Model ranks patent pathway as top-1: Example 41

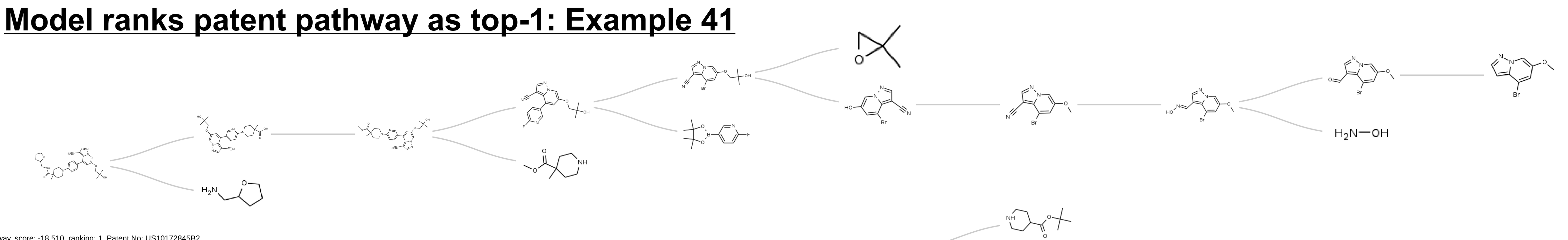

Piatachio pathway, score: -18.510, ranking: 1, Patent No: US10172845B2

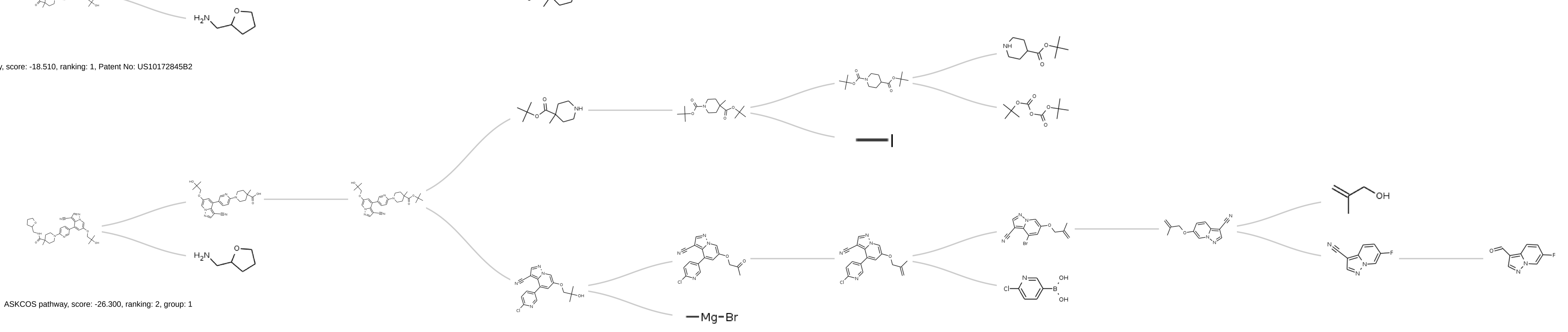

ASKCOS pathway, score: -26.300, ranking: 2, group: 1

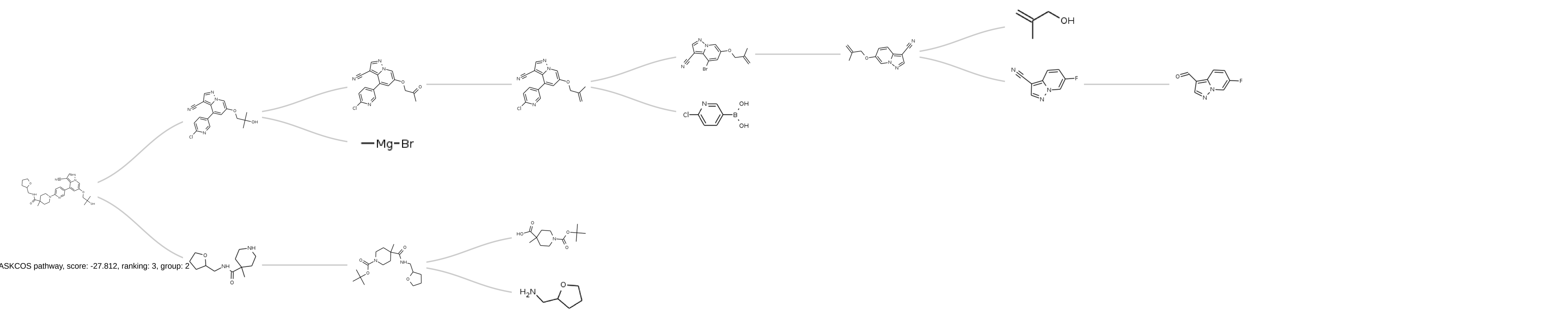

ASKCOS pathway, score: -27.812, ranking: 3, group: 2

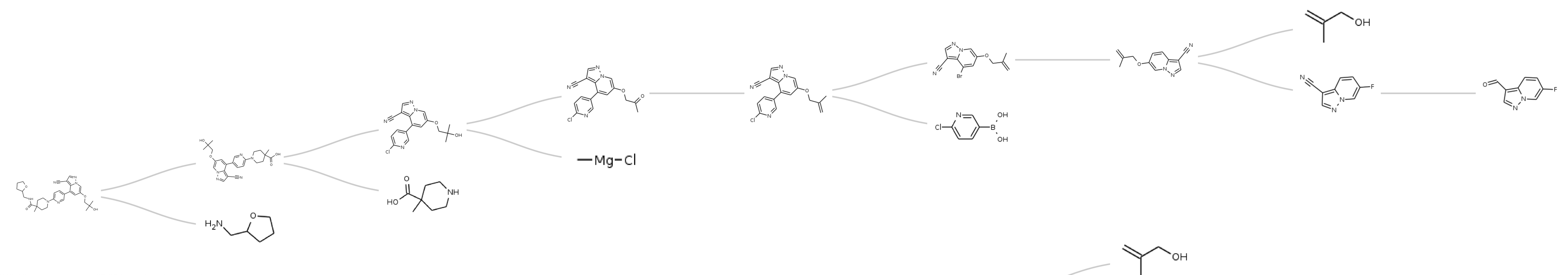

ASKCOS pathway, score: -27.854, ranking: 4, group: 3

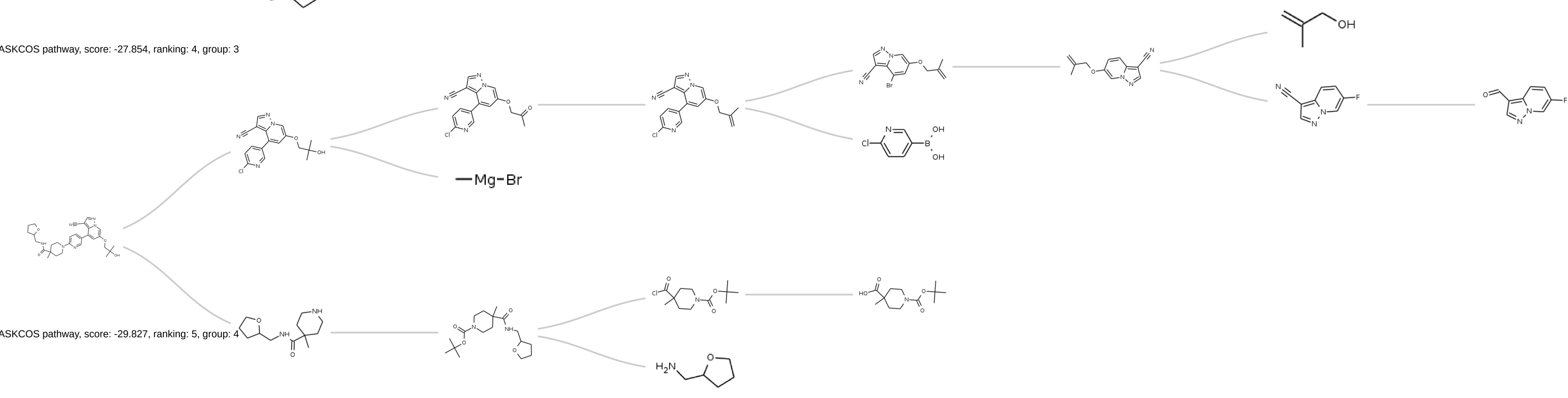

ASKCOS pathway, score: -29.827, ranking: 5, group: 4

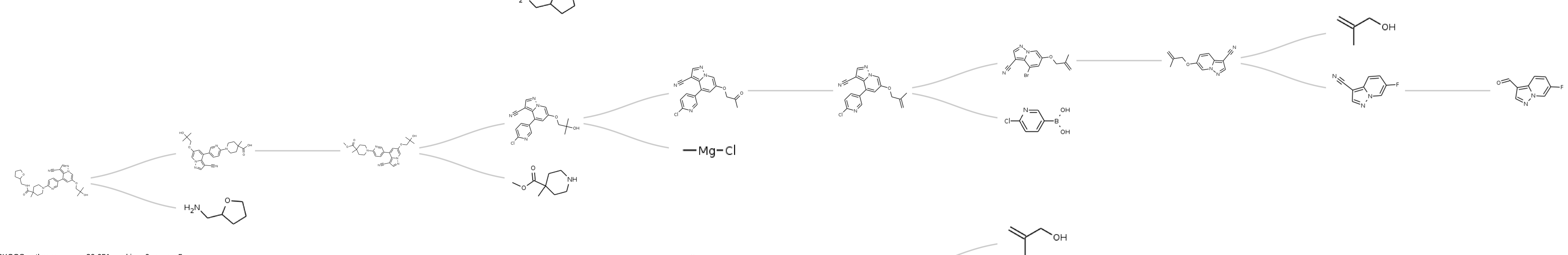

ASKCOS pathway, score: -30.651, ranking: 6, group: 5

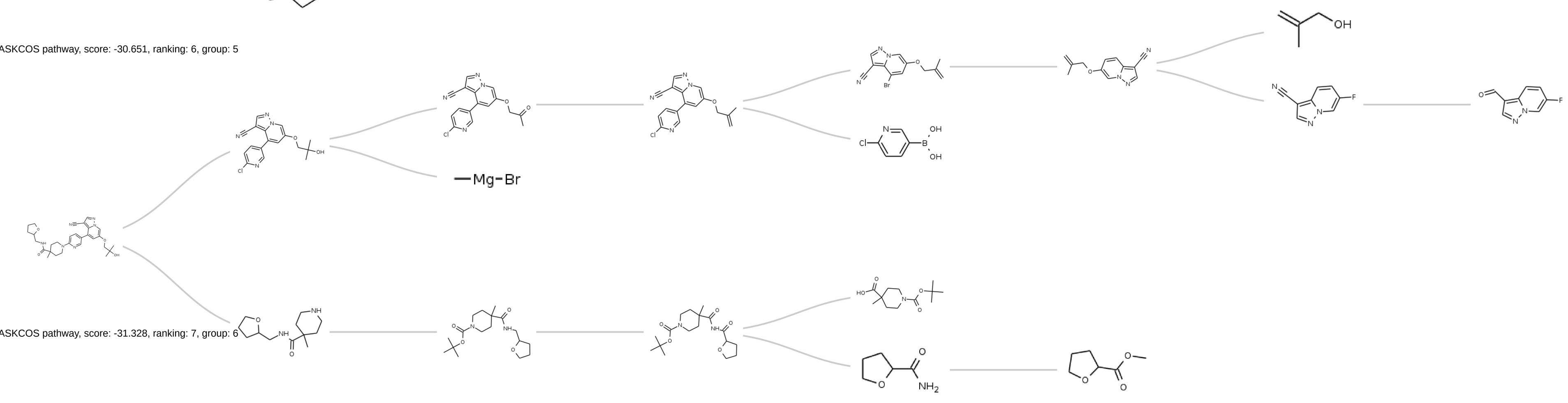

ASKCOS pathway, score: -31.328, ranking: 7, group: 6

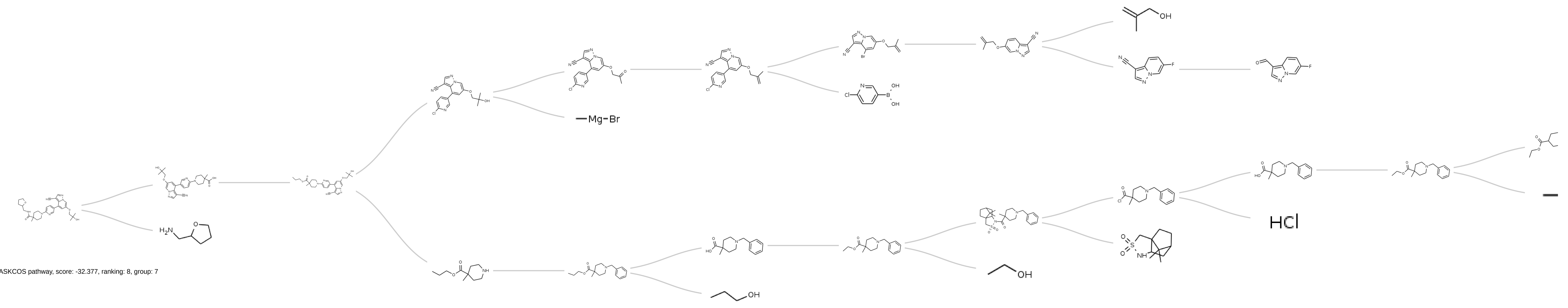

ASKCOS pathway, score: -32.377, ranking: 8, group: 7

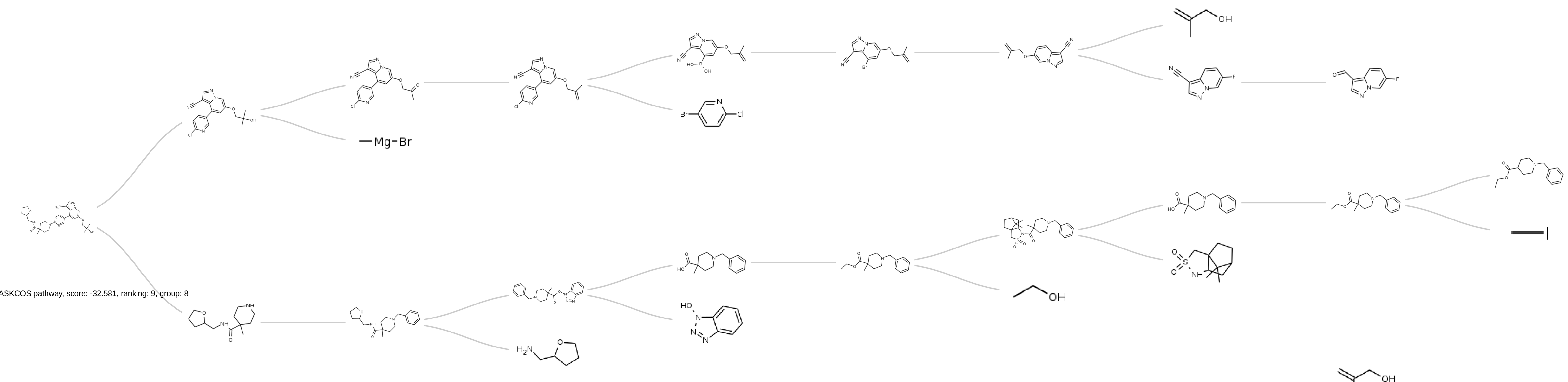

ASKCOS pathway, score: -32.581, ranking: 9, group: 8

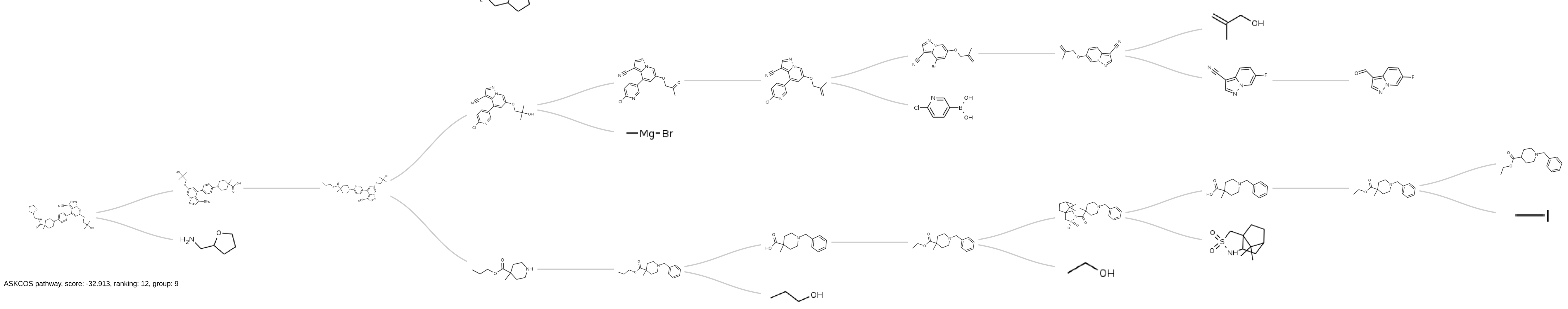

ASKCOS pathway, score: -32.913, ranking: 12, group: 9

Model ranks patent pathway as top-1: Example 42

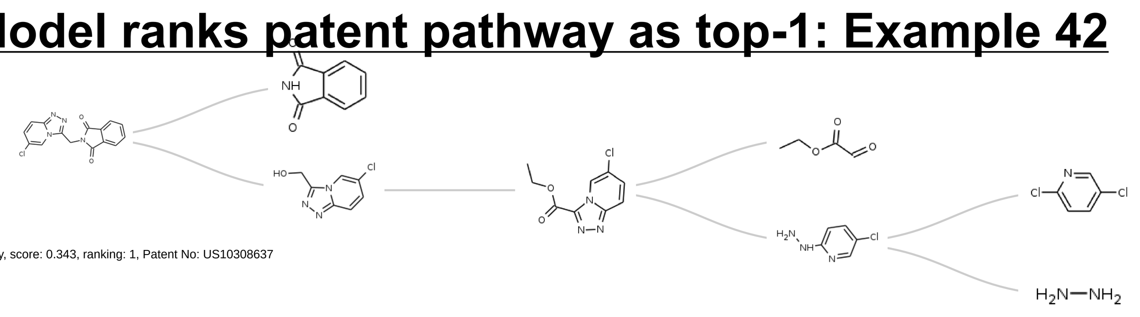

Piatachio pathway, score: 0.343, ranking: 1, Patent No: US10308637

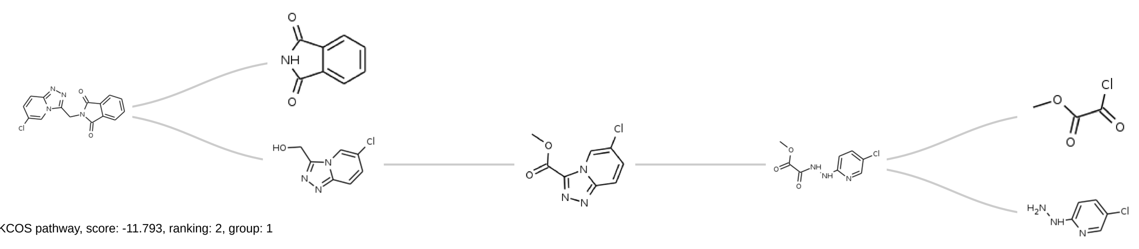

ASKCOS pathway, score: -11.793, ranking: 2, group: 1

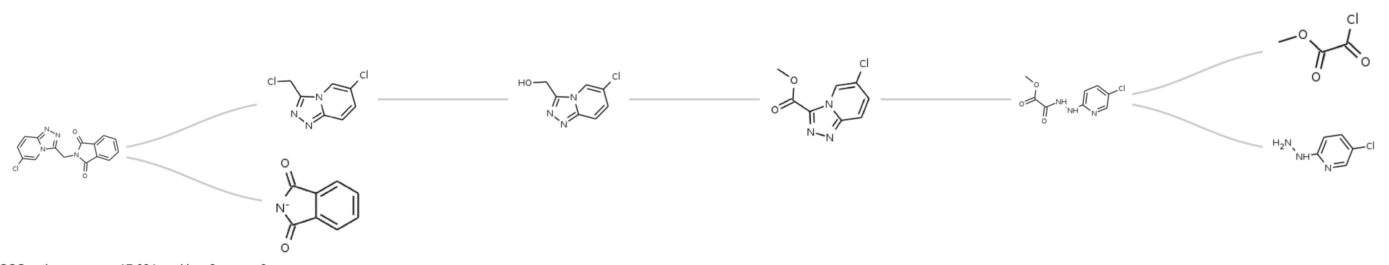

ASKCOS pathway, score: -17.624, ranking: 3, group: 2

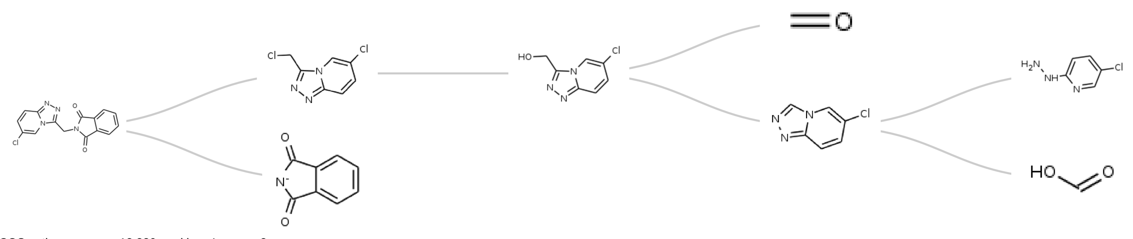

ASKCOS pathway, score: -18.380, ranking: 4, group: 3

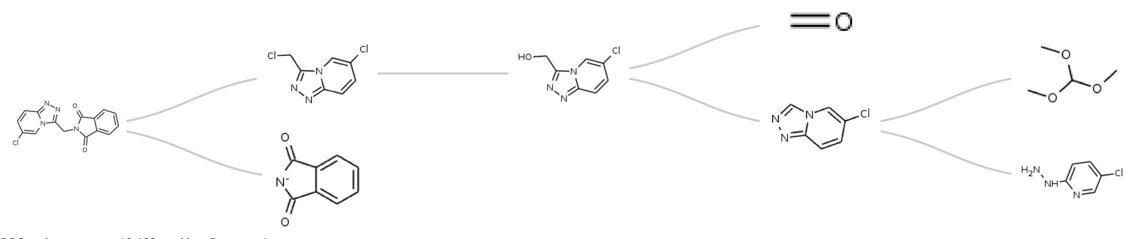

ASKCOS pathway, score: -19.138, ranking: 5, group: 4

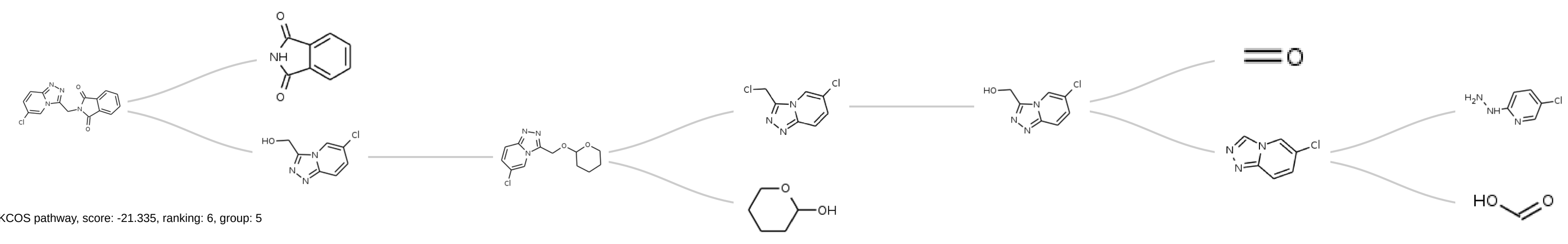

ASKCOS pathway, score: -21.335, ranking: 6, group: 5

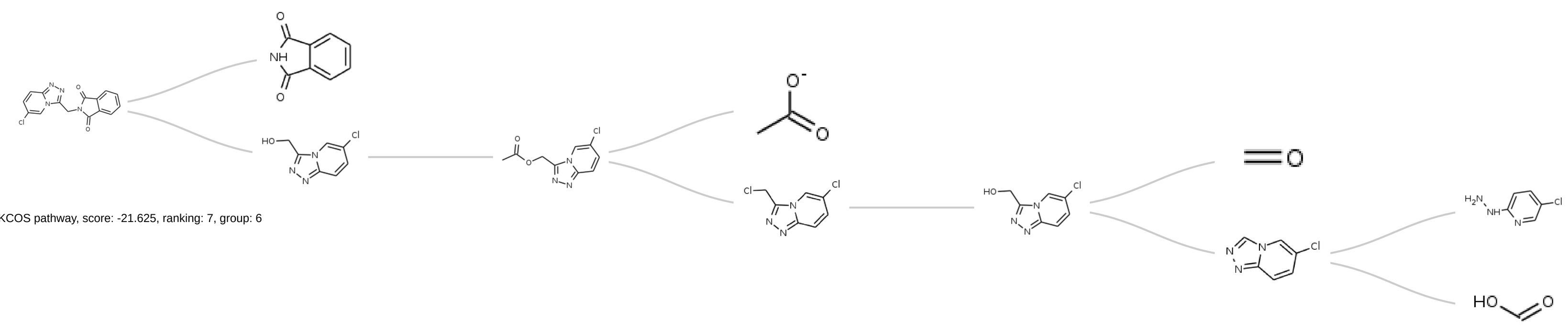

ASKCOS pathway, score: -21.625, ranking: 7, group: 6

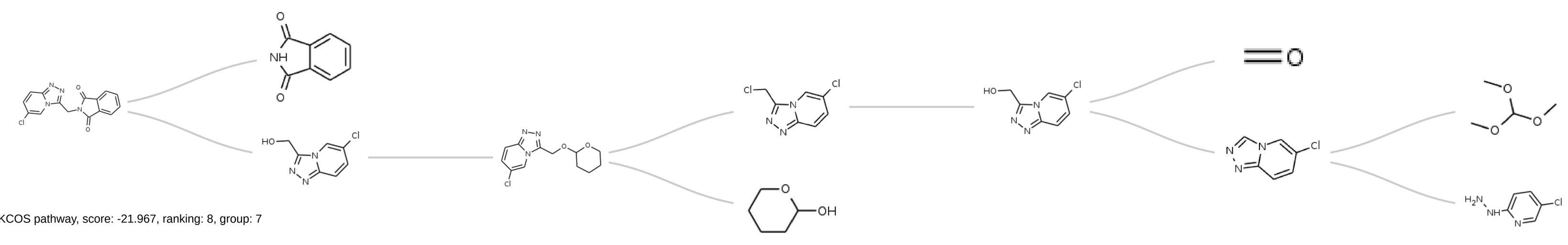

ASKCOS pathway, score: -21.967, ranking: 8, group: 7

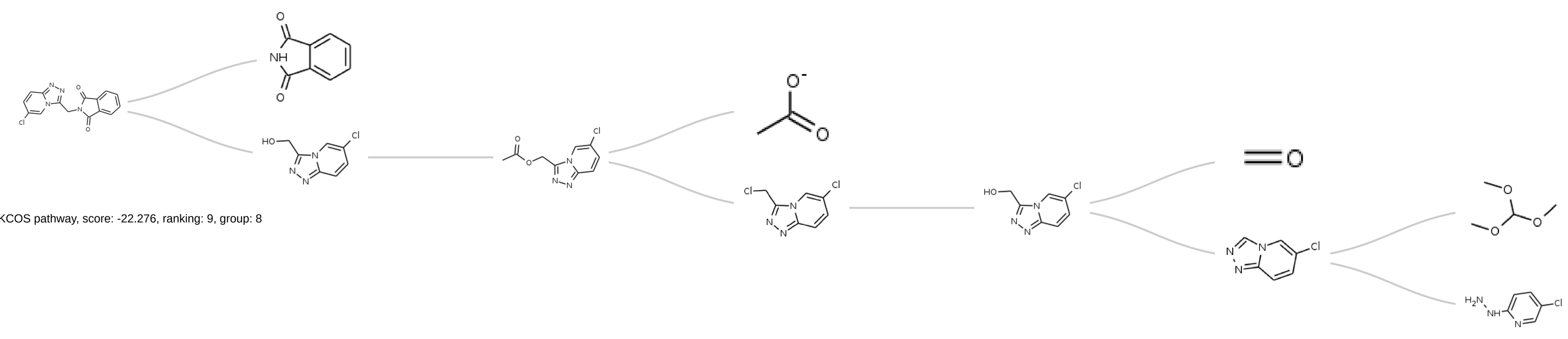

ASKCOS pathway, score: -22.276, ranking: 9, group: 8

Model ranks patent pathway as top-1: Example 43

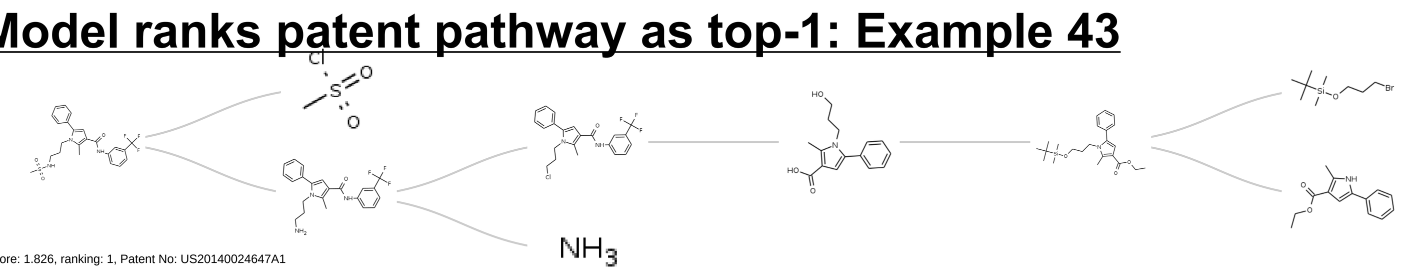

Piatachio pathway, score: 1.826, ranking: 1, Patent No: US20140024647A1

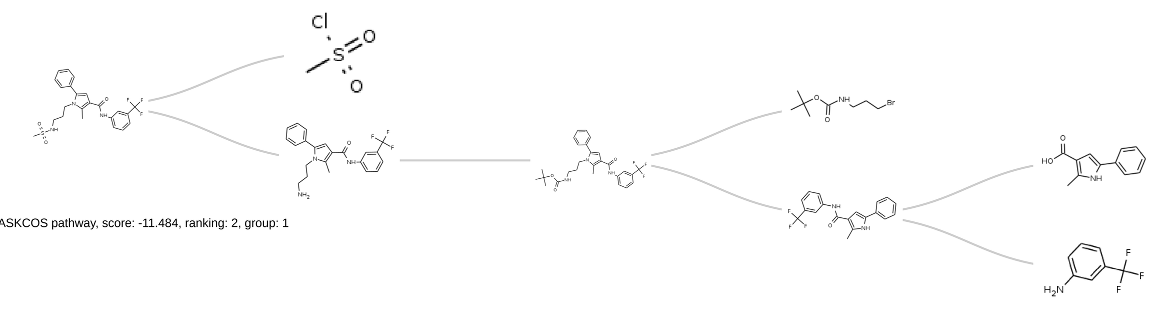

ASKCOS pathway, score: -11.484, ranking: 2, group: 1

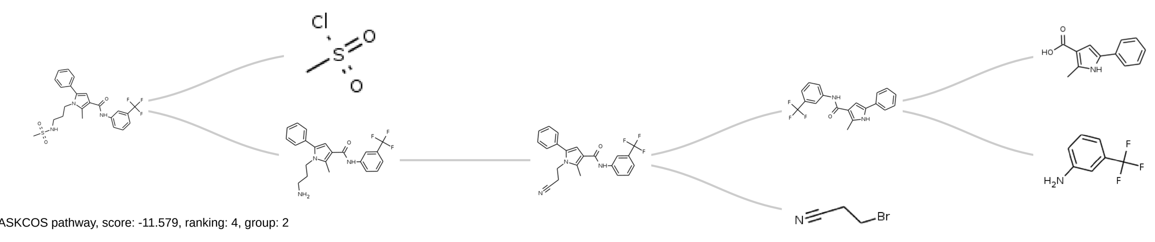

ASKCOS pathway, score: -11.579, ranking: 4, group: 2

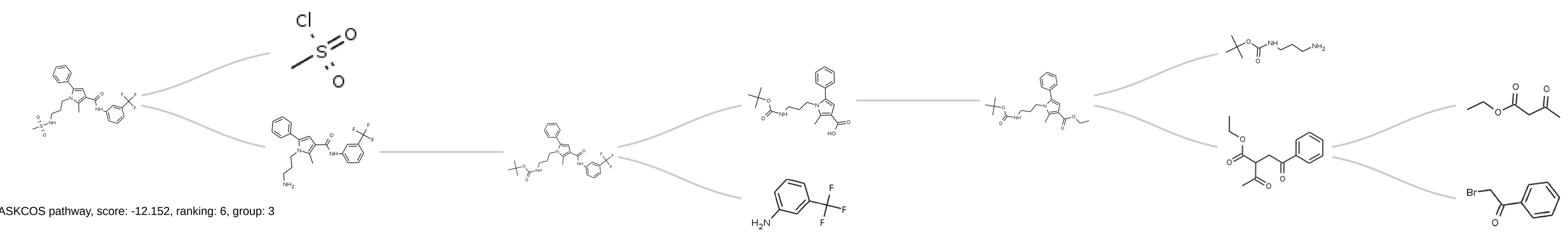

ASKCOS pathway, score: -12.152, ranking: 6, group: 3

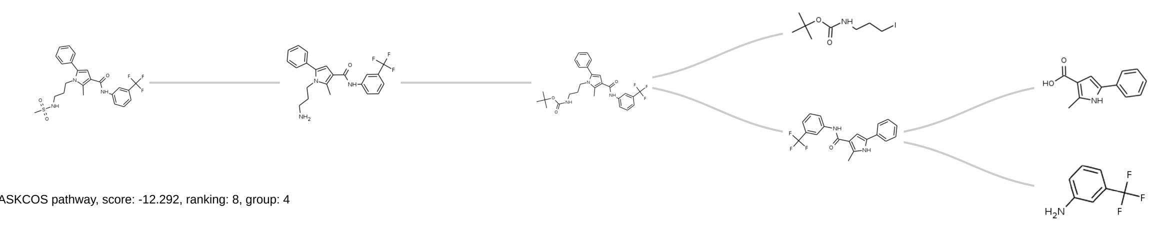

ASKCOS pathway, score: -12.292, ranking: 8, group: 4

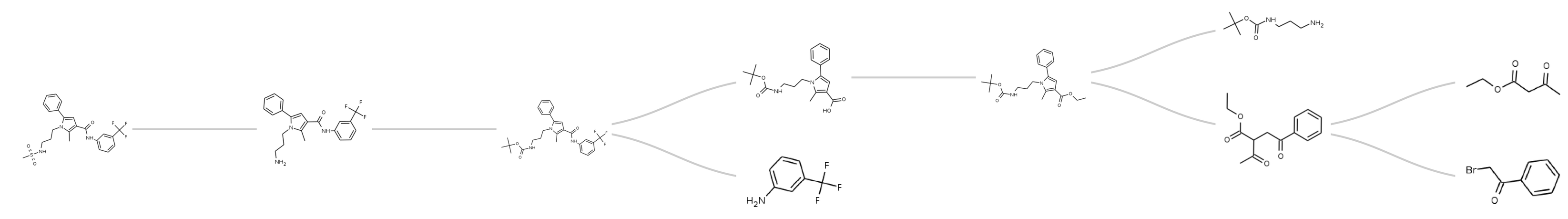

ASKCOS pathway, score: -12.662, ranking: 10, group: 5

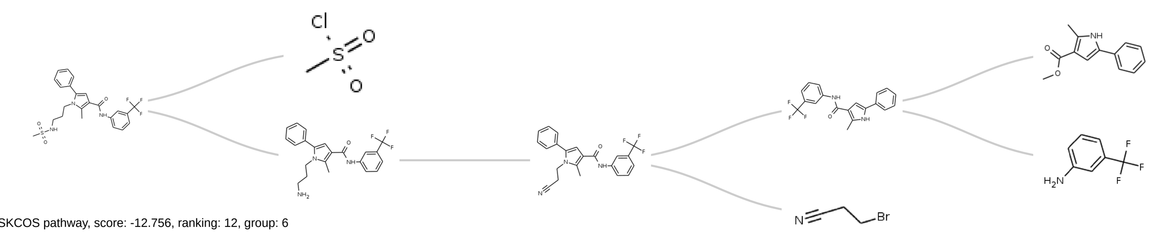

ASKCOS pathway, score: -12.756, ranking: 12, group: 6

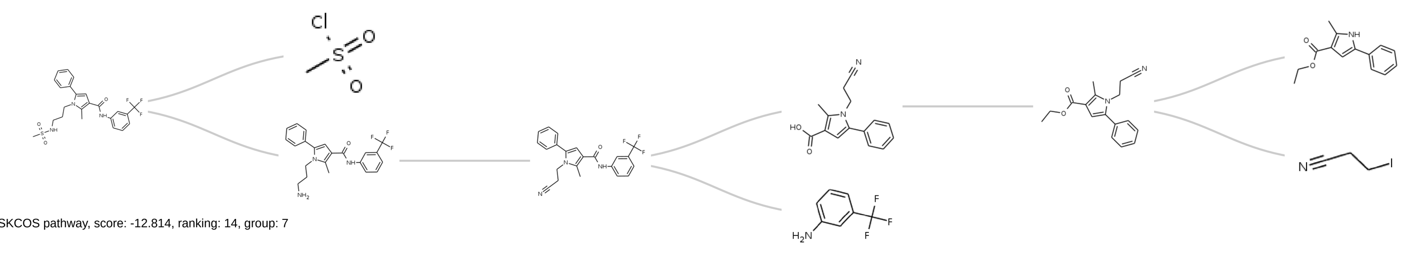

ASKCOS pathway, score: -12.814, ranking: 14, group: 7

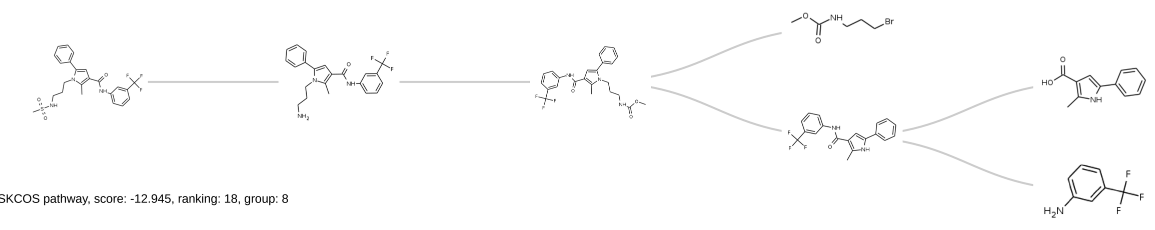

ASKCOS pathway, score: -12.945, ranking: 18, group: 8

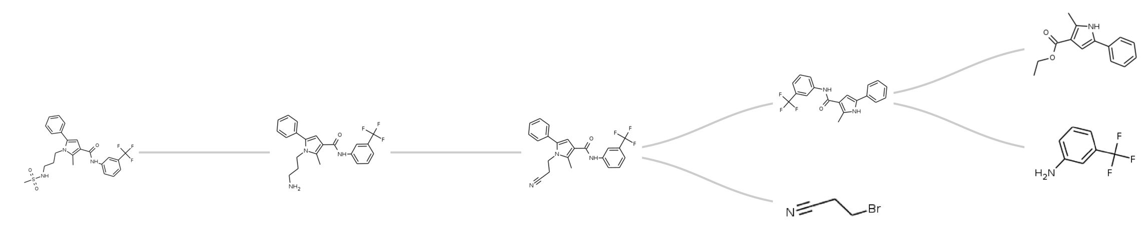

ASKCOS pathway, score: -12.964, ranking: 19, group: 9

Model ranks patent pathway as top-1: Example 44

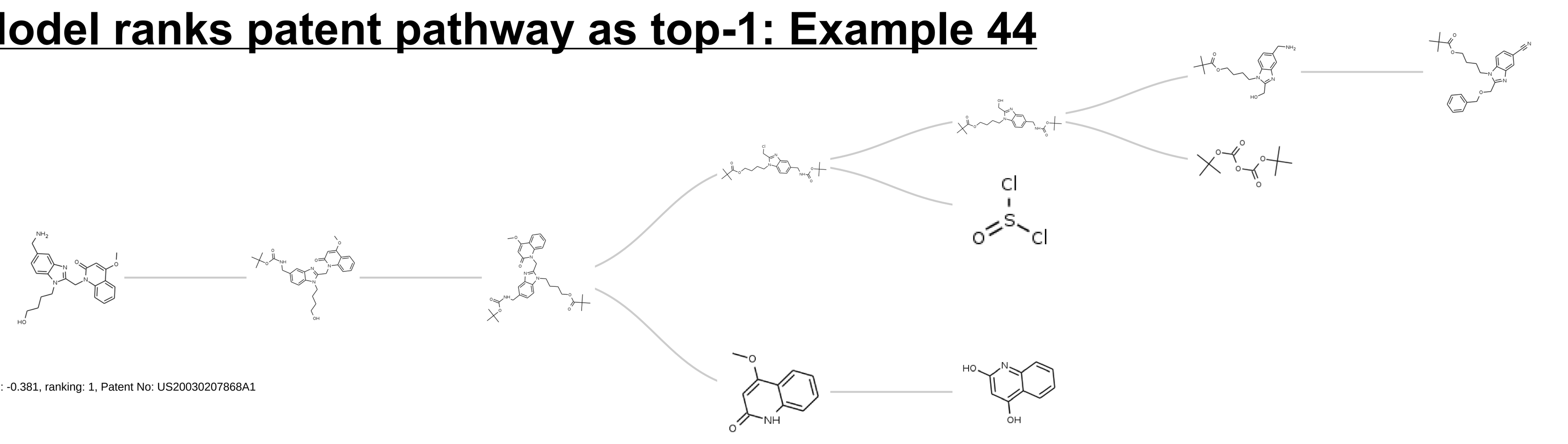

Piatachio pathway, score: -0.381, ranking: 1, Patent No: US20030207868A1

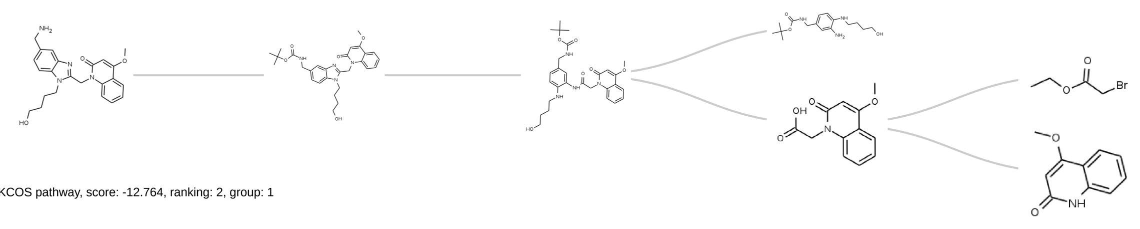

ASKCOS pathway, score: -12.764, ranking: 2, group: 1

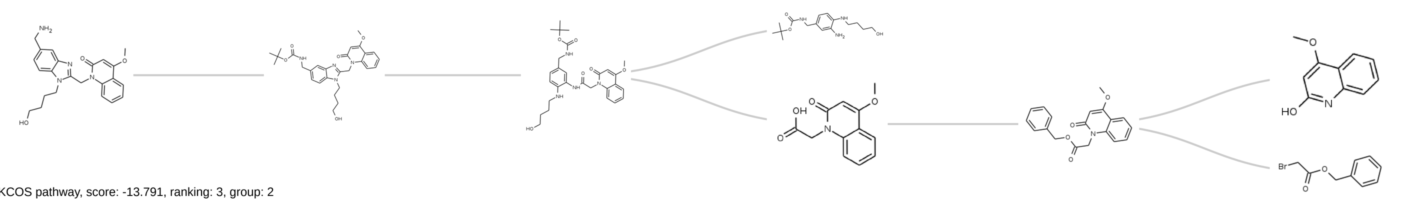

ASKCOS pathway, score: -13.791, ranking: 3, group: 2

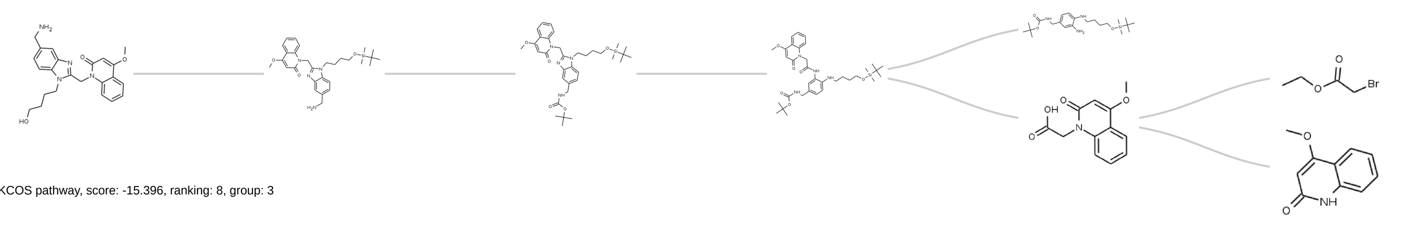

ASKCOS pathway, score: -15.396, ranking: 8, group: 3

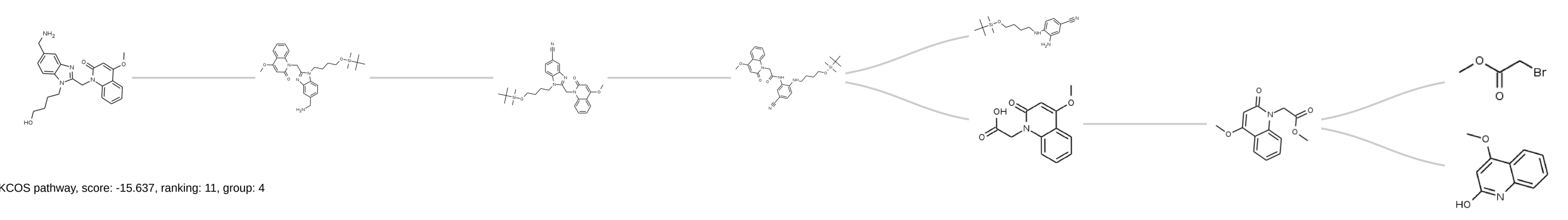

ASKCOS pathway, score: -15.637, ranking: 11, group: 4

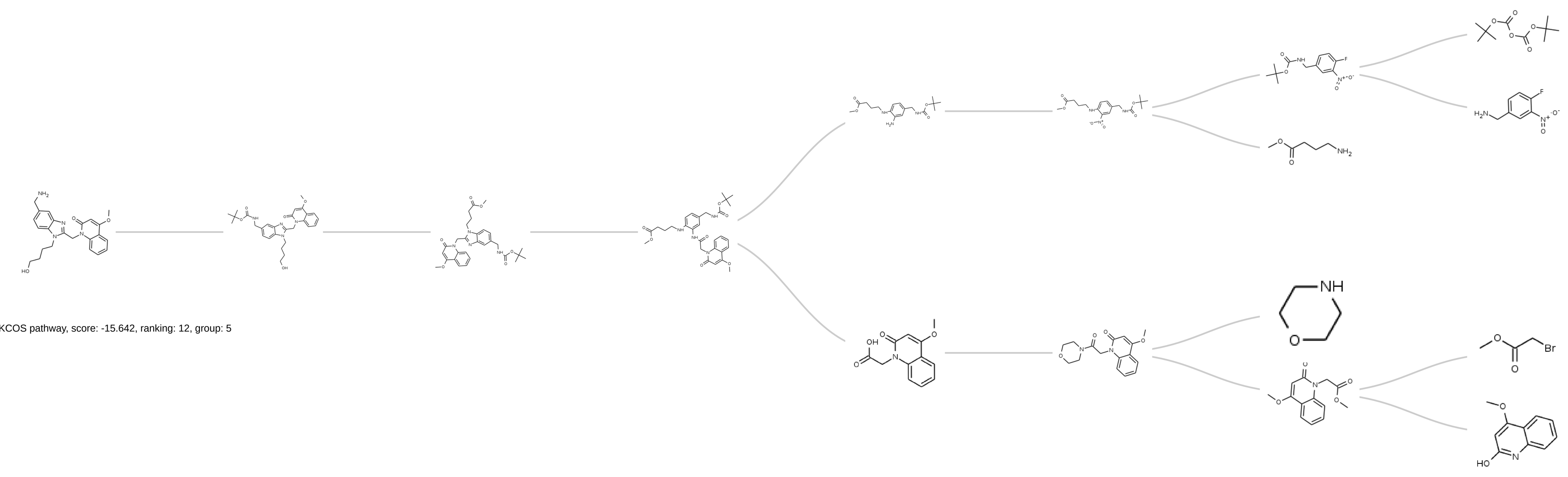

ASKCOS pathway, score: -15.642, ranking: 12, group: 5

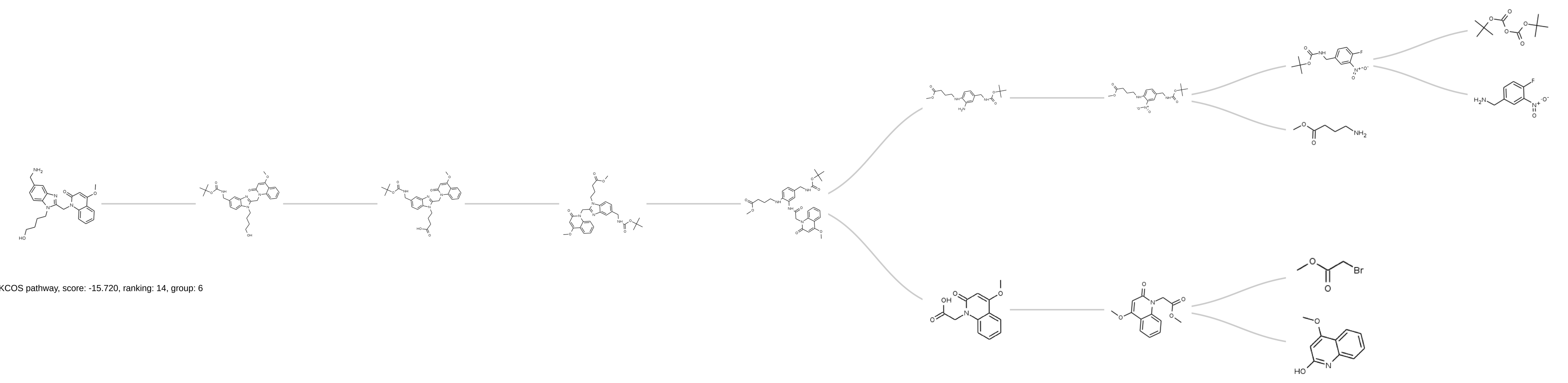

ASKCOS pathway, score: -15.720, ranking: 14, group: 6

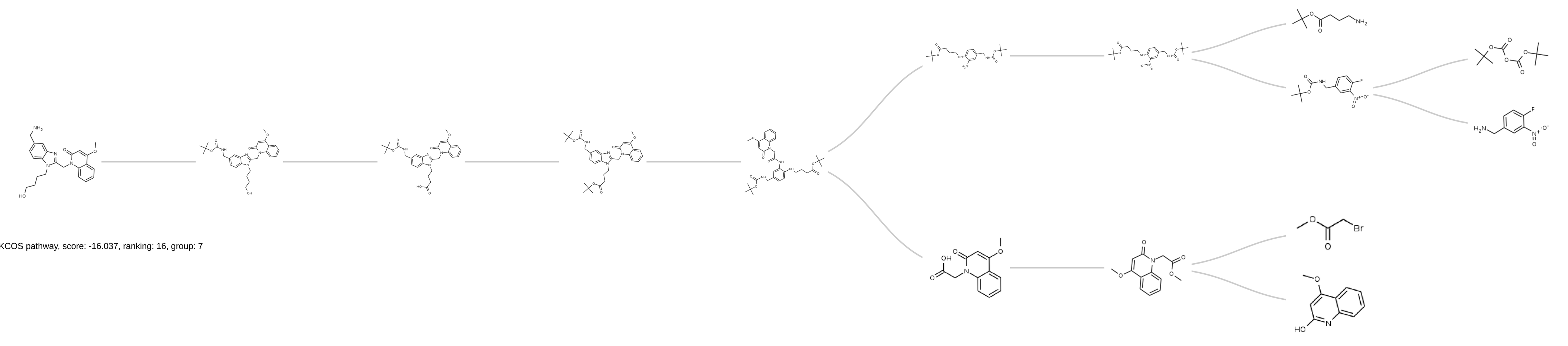

ASKCOS pathway, score: -16.037, ranking: 16, group: 7

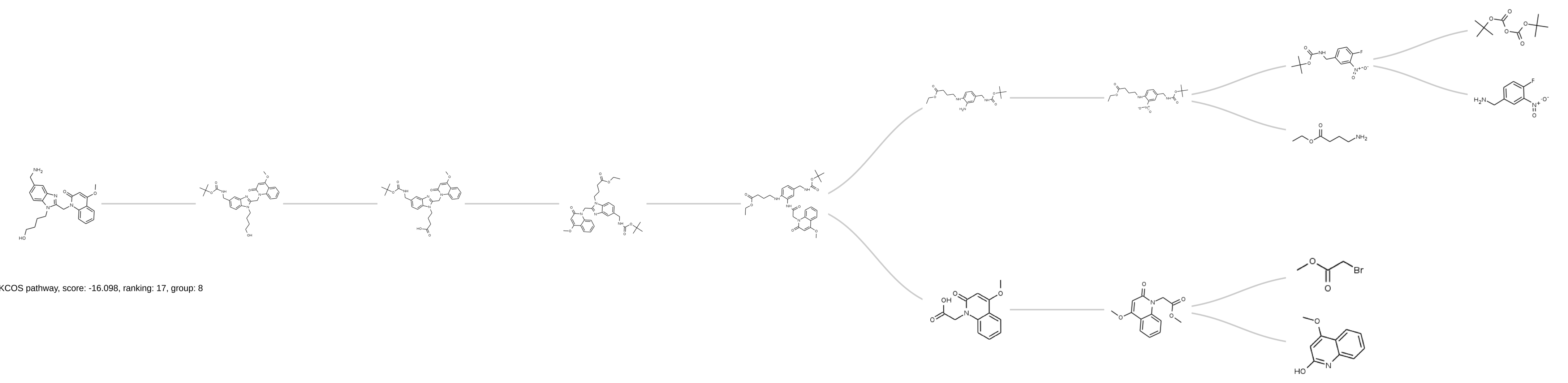

ASKCOS pathway, score: -16.098, ranking: 17, group: 8

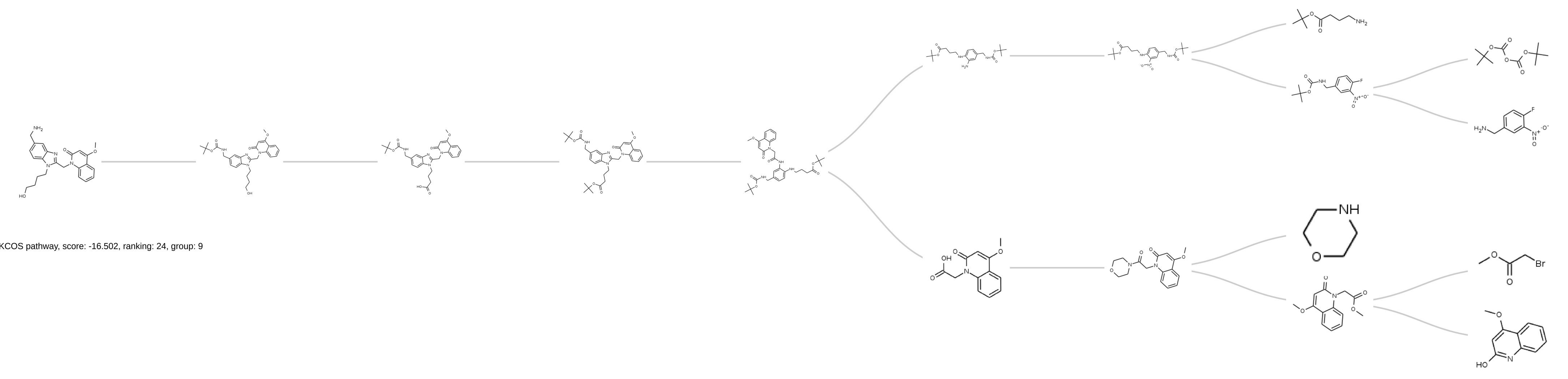

ASKCOS pathway, score: -16.502, ranking: 24, group: 9

Model ranks patent pathway as top-1: Example 45

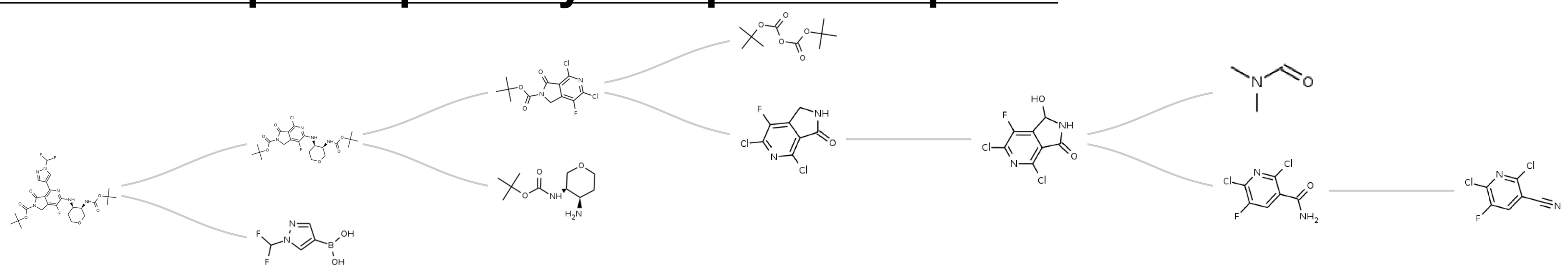

Piatachio pathway, score: -12.616, ranking: 1, Patent No: US20110152273A1

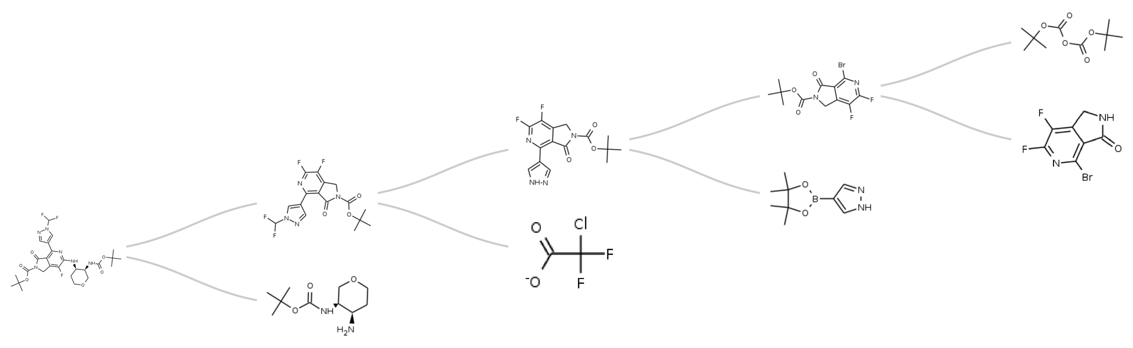

ASKCOS pathway, score: -18.338, ranking: 2, group: 1

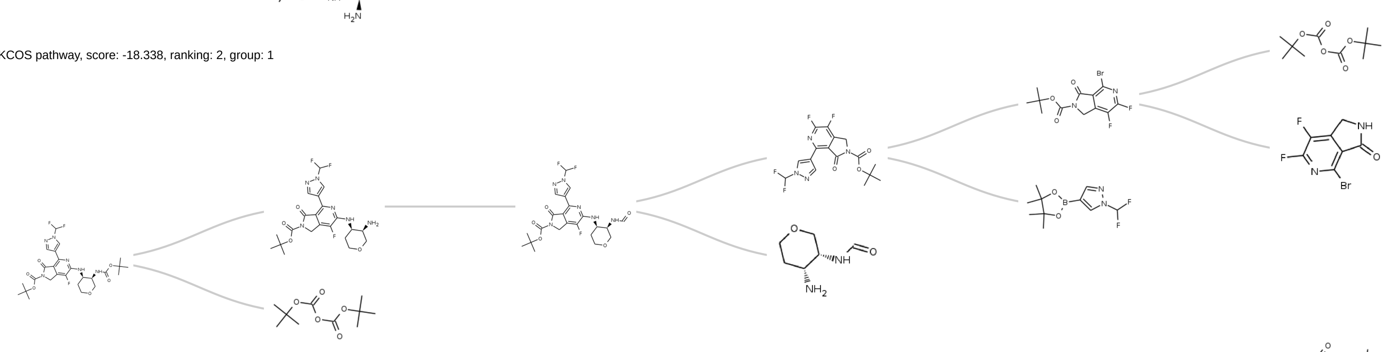

ASKCOS pathway, score: -23.986, ranking: 3, group: 2

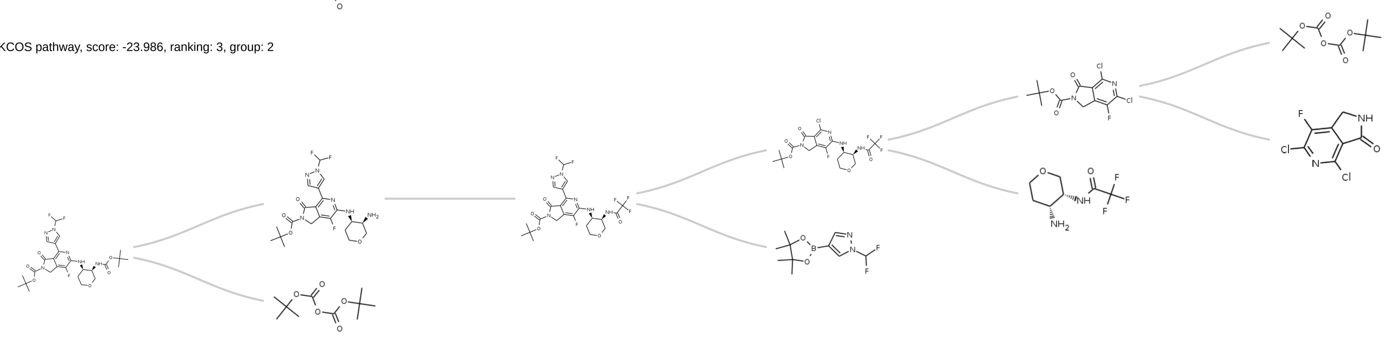

ASKCOS pathway, score: -24.753, ranking: 6, group: 3

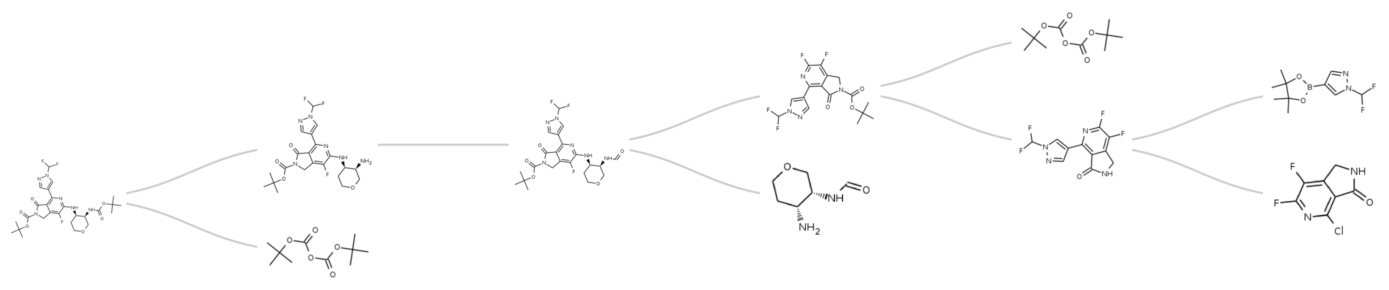

ASKCOS pathway, score: -27.769, ranking: 19, group: 4

Model ranks patent pathway as top-1: Example 46

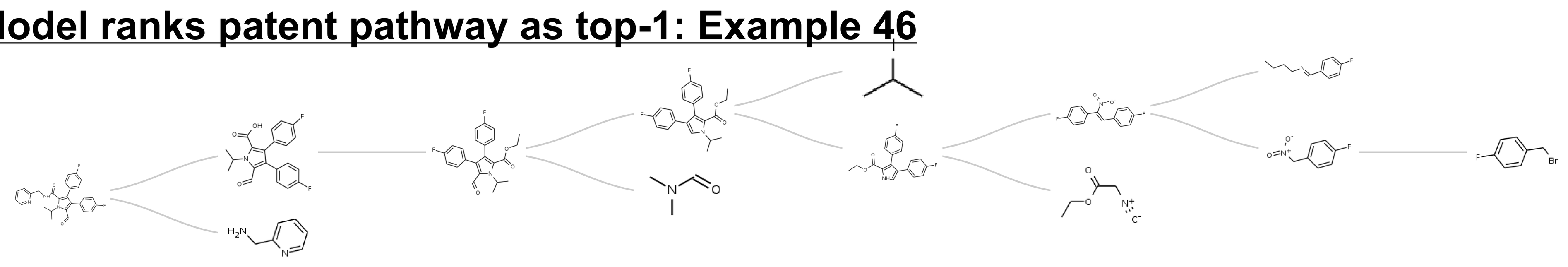

Piatachio pathway, score: -12.469, ranking: 1, Patent No: US20050154042A1

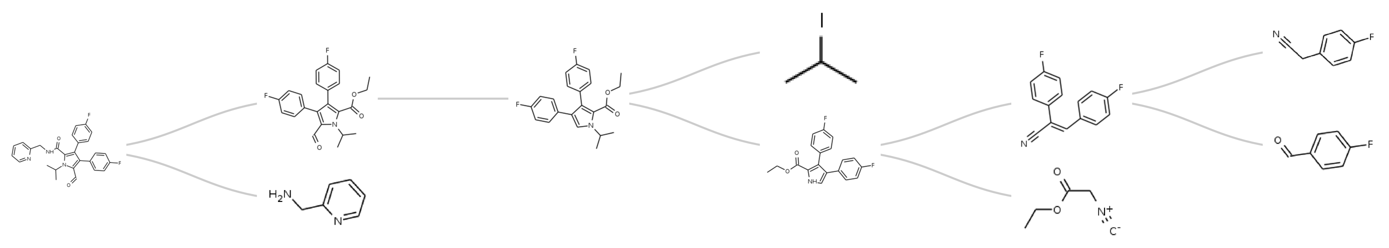

ASKCOS pathway, score: -13.435, ranking: 2, group: 1

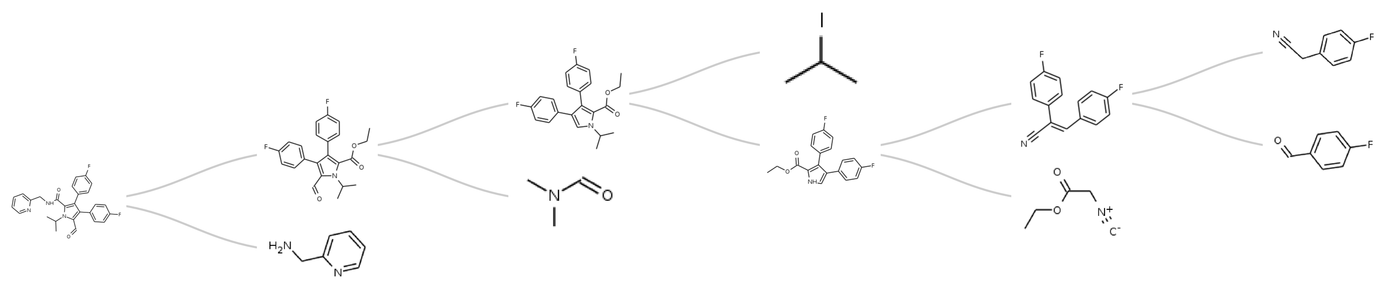

ASKCOS pathway, score: -13.554, ranking: 3, group: 2

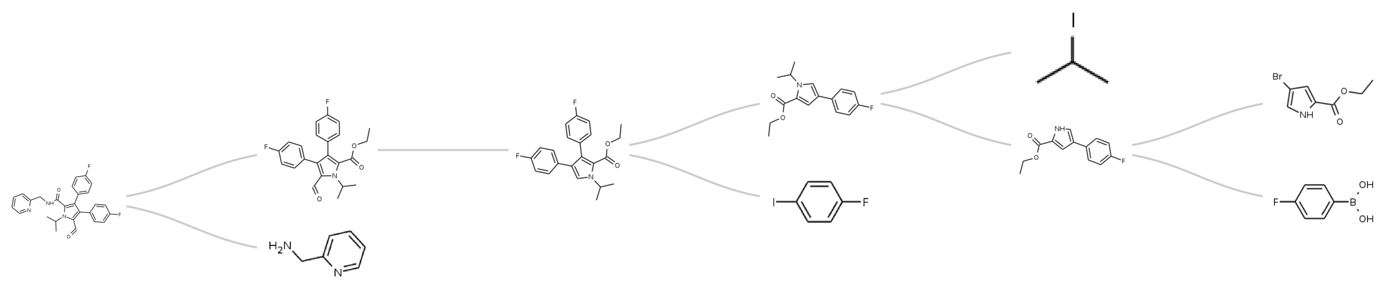

ASKCOS pathway, score: -18.741, ranking: 17, group: 3

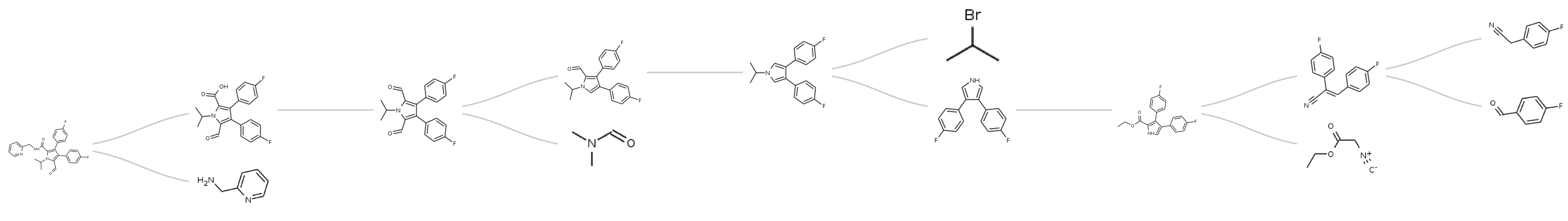

ASKCOS pathway, score: -19.026, ranking: 20, group: 4

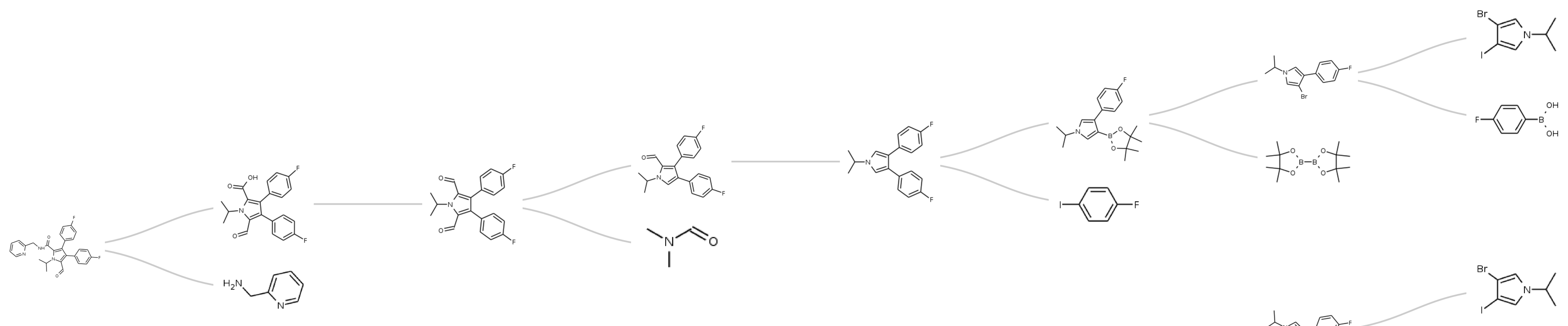

ASKCOS pathway, score: -20.423, ranking: 36, group: 5

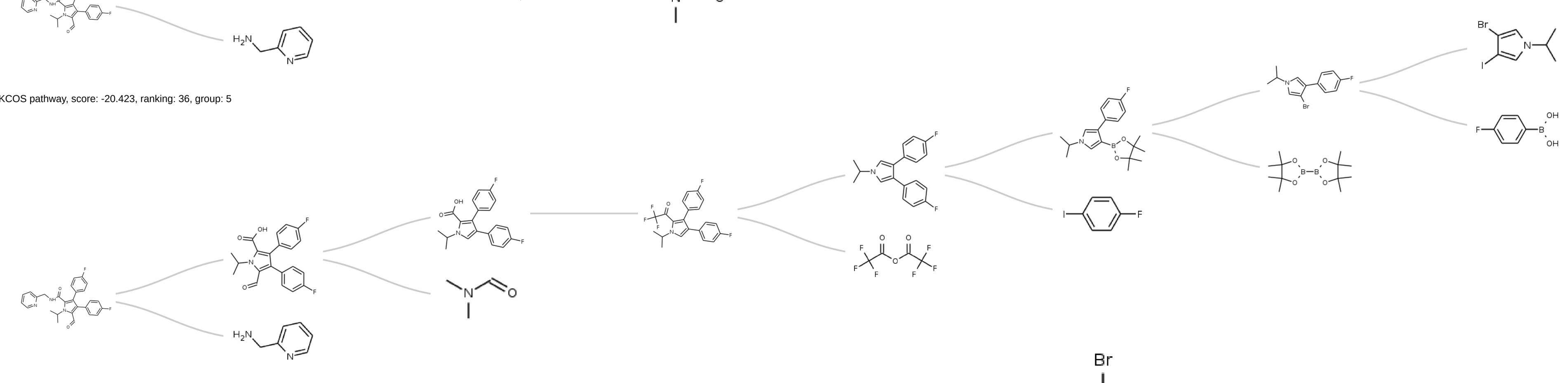

ASKCOS pathway, score: -21.339, ranking: 47, group: 6

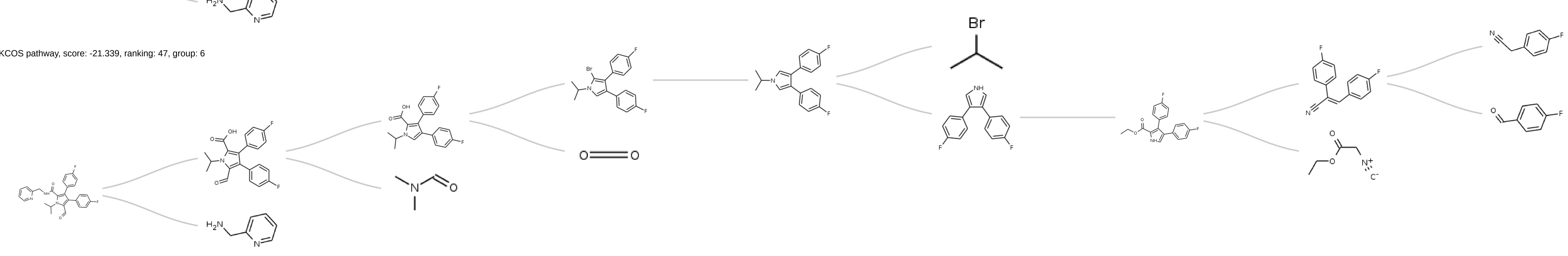

ASKCOS pathway, score: -21.863, ranking: 57, group: 7

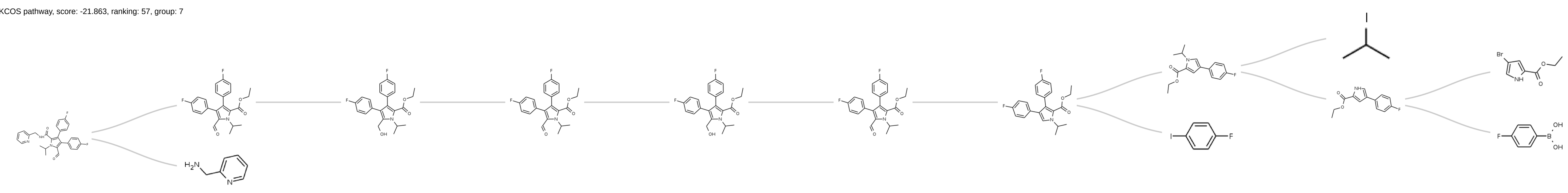

ASKCOS pathway, score: -22.229, ranking: 62, group: 8

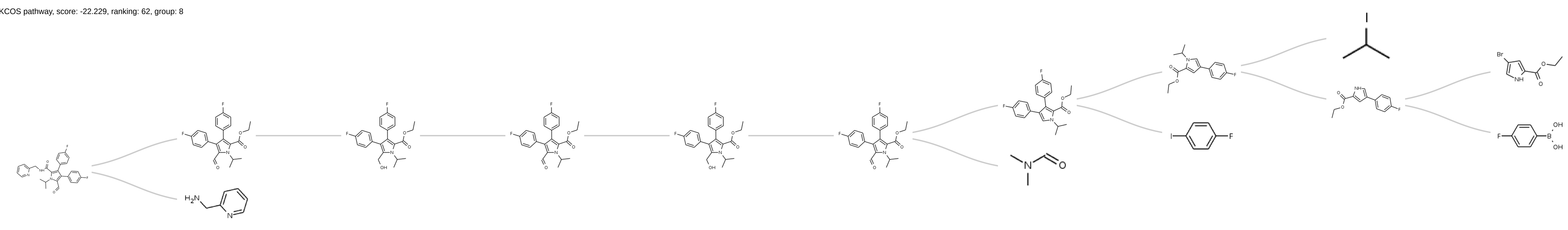

ASKCOS pathway, score: -22.348, ranking: 63, group: 9

Model ranks patent pathway as top-1: Example 47

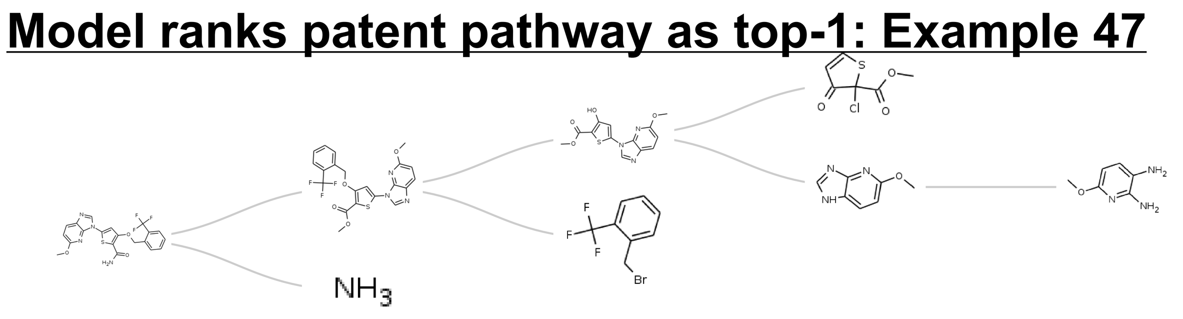

Piatachio pathway, score: -12.786, ranking: 1, Patent No: US20100278833A1

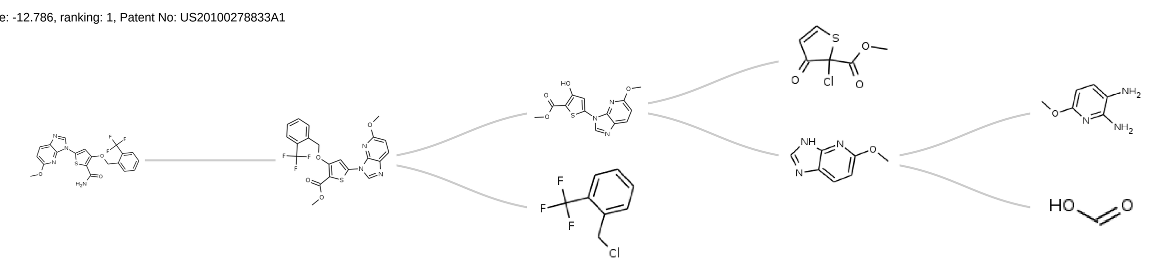

ASKCOS pathway, score: -16.343, ranking: 2, group: 1

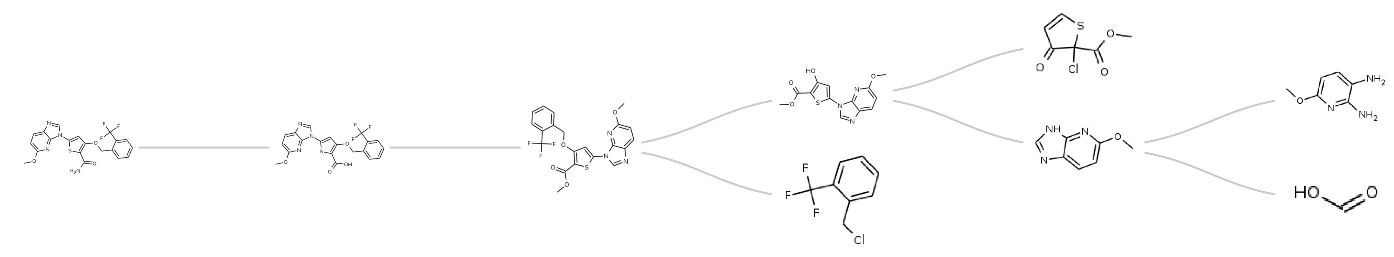

ASKCOS pathway, score: -18.583, ranking: 10, group: 2

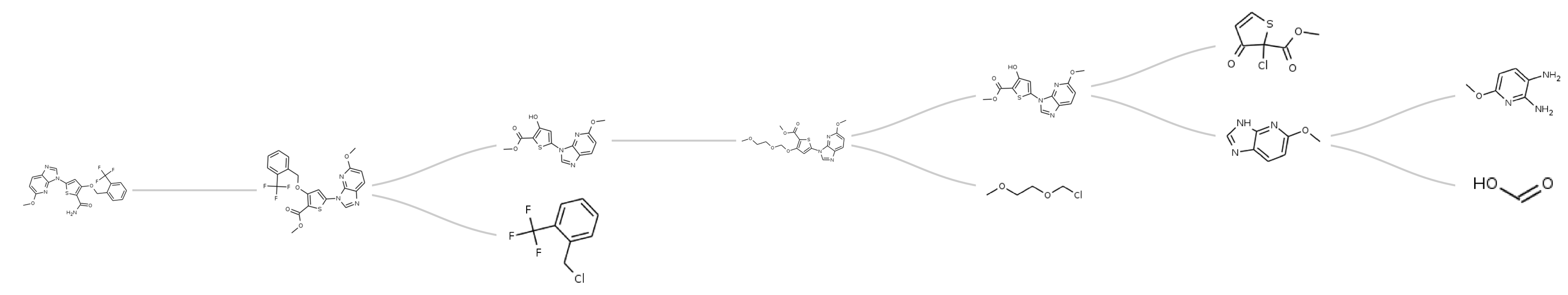

ASKCOS pathway, score: -19.437, ranking: 16, group: 3

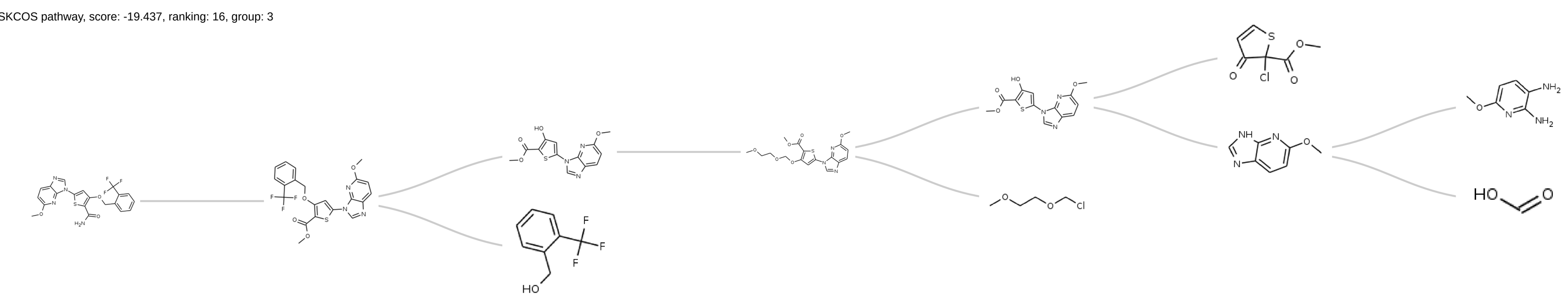

ASKCOS pathway, score: -19.881, ranking: 19, group: 4

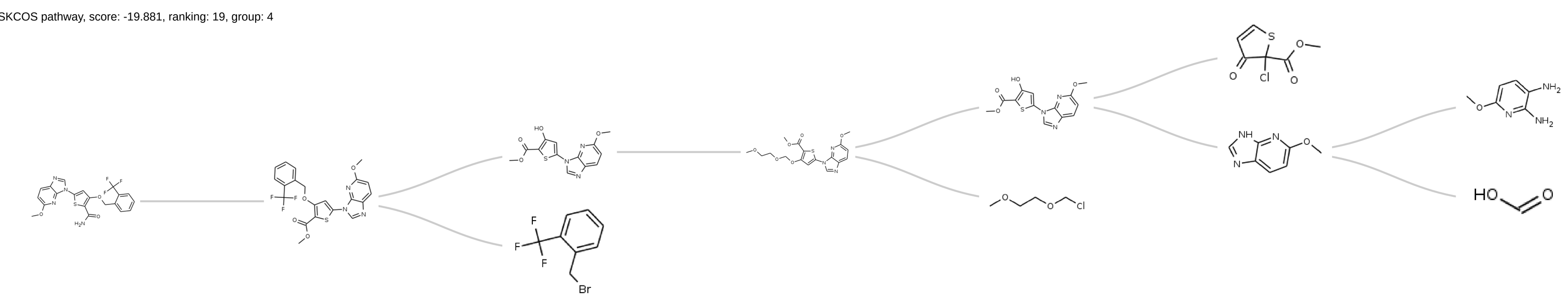

ASKCOS pathway, score: -20.037, ranking: 20, group: 5

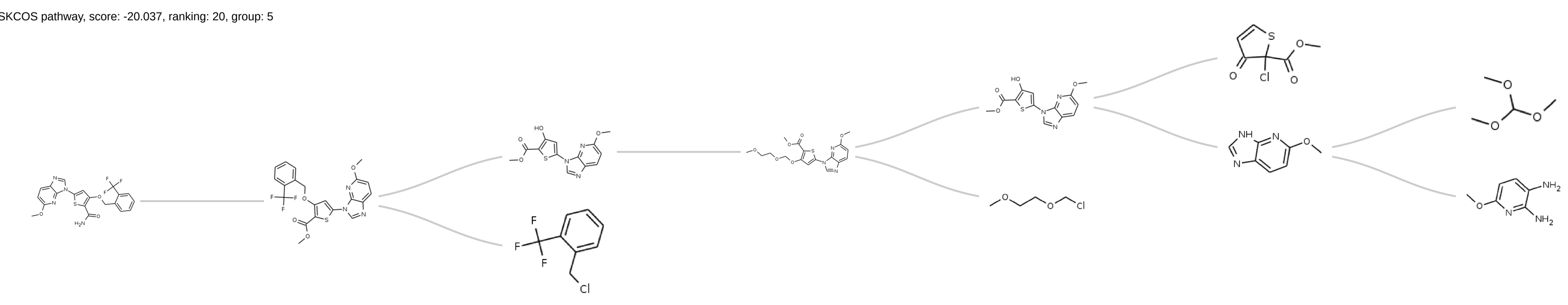

ASKCOS pathway, score: -20.352, ranking: 24, group: 6

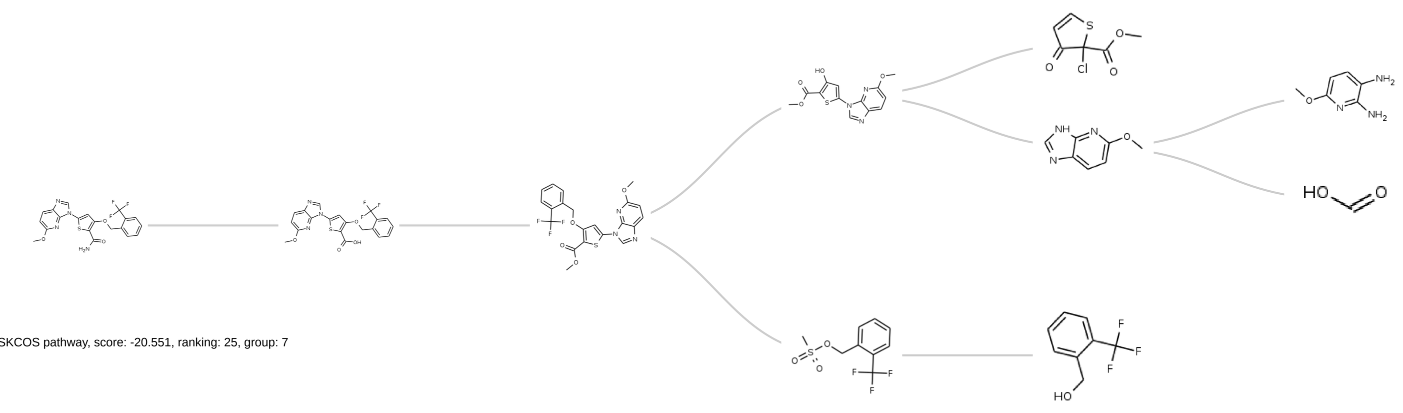

ASKCOS pathway, score: -20.551, ranking: 25, group: 7

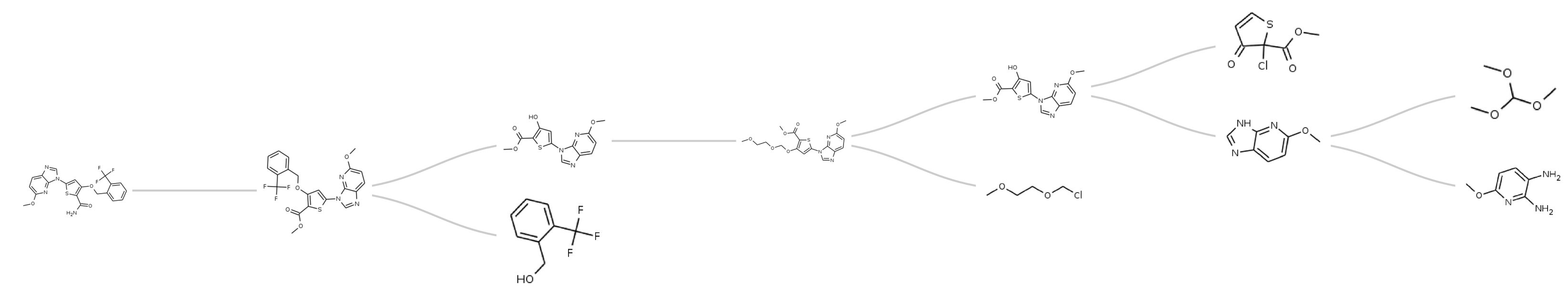

ASKCOS pathway, score: -20.795, ranking: 27, group: 8

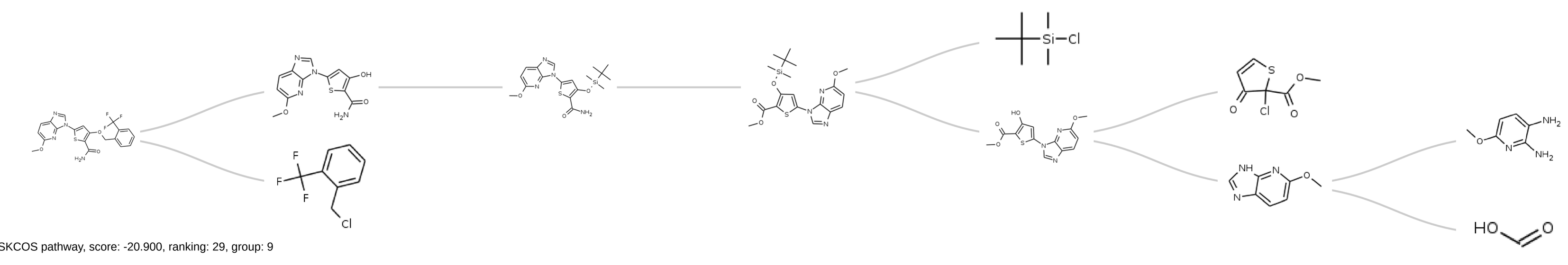

ASKCOS pathway, score: -20.900, ranking: 29, group: 9

Model ranks patent pathway as top-1: Example 48

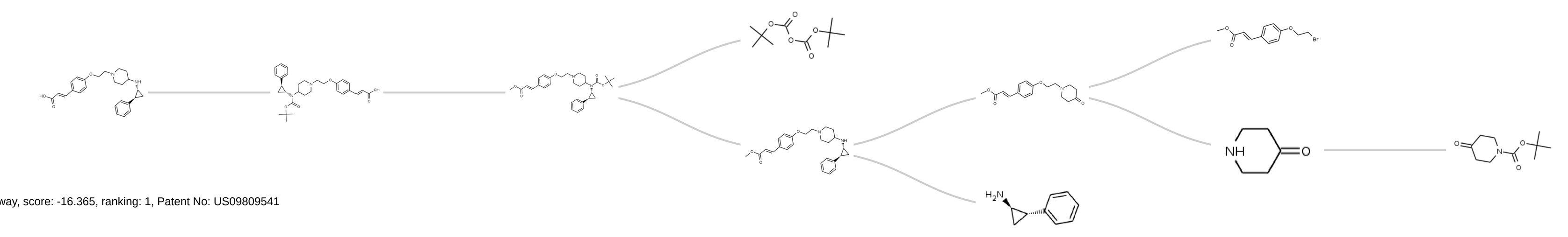

Piatachio pathway, score: -16.365, ranking: 1, Patent No: US09809541

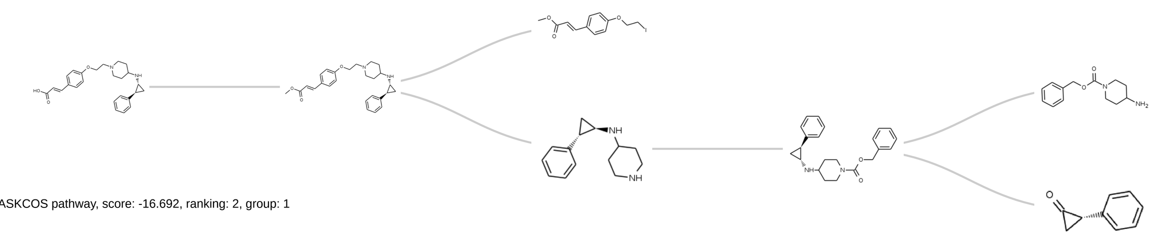

ASKCOS pathway, score: -16.692, ranking: 2, group: 1

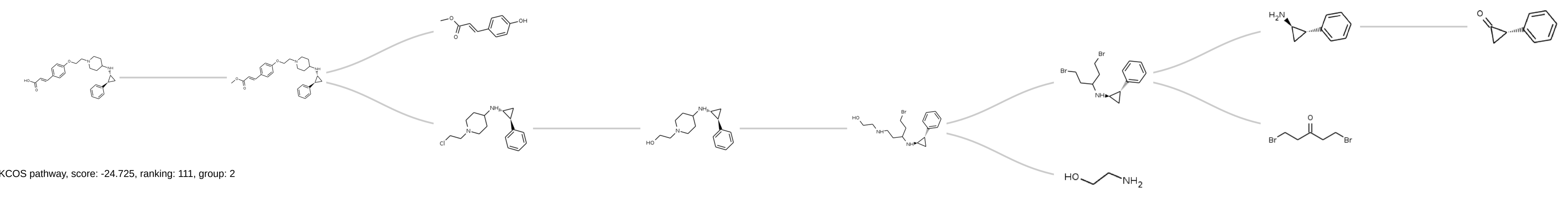

ASKCOS pathway, score: -24.725, ranking: 111, group: 2

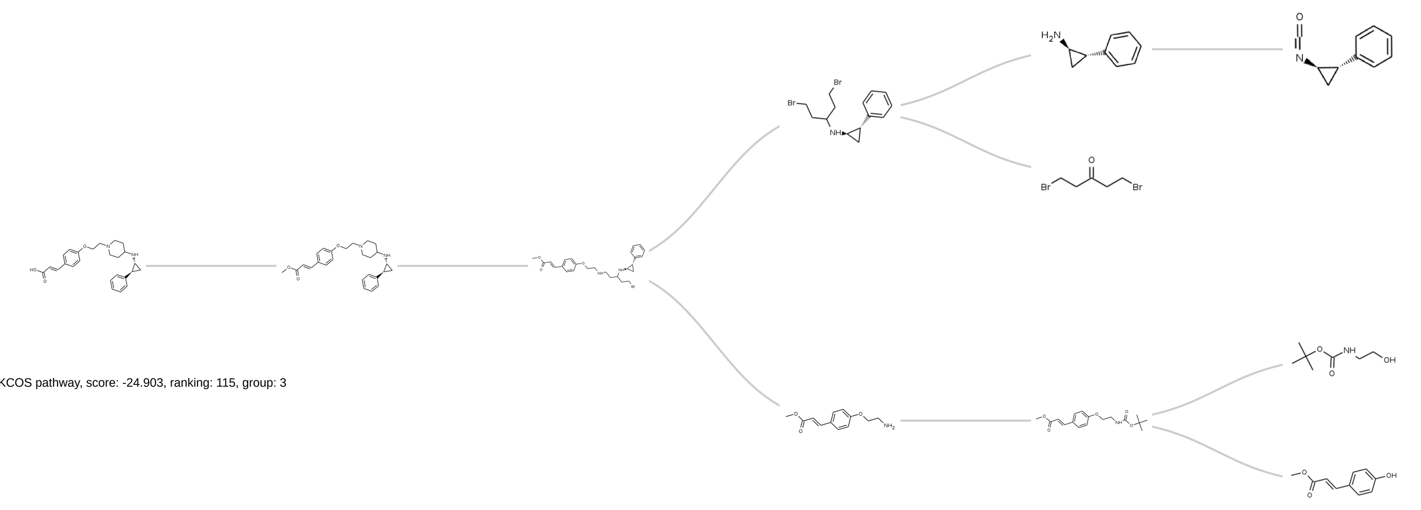

ASKCOS pathway, score: -24.903, ranking: 115, group: 3

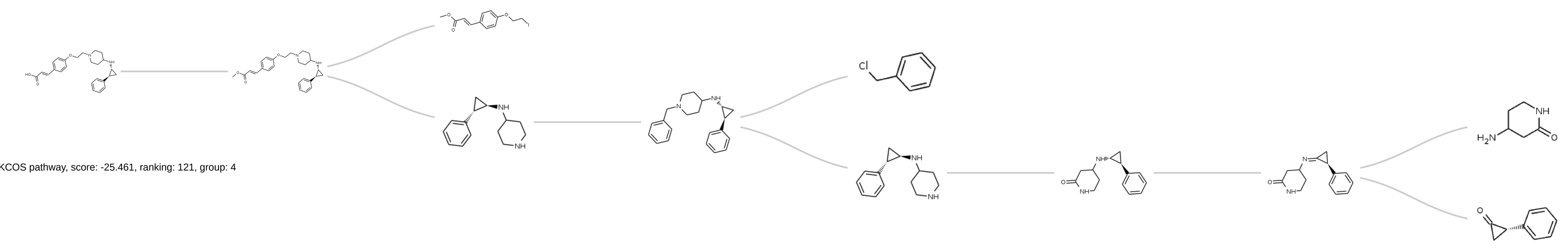

ASKCOS pathway, score: -25.461, ranking: 121, group: 4

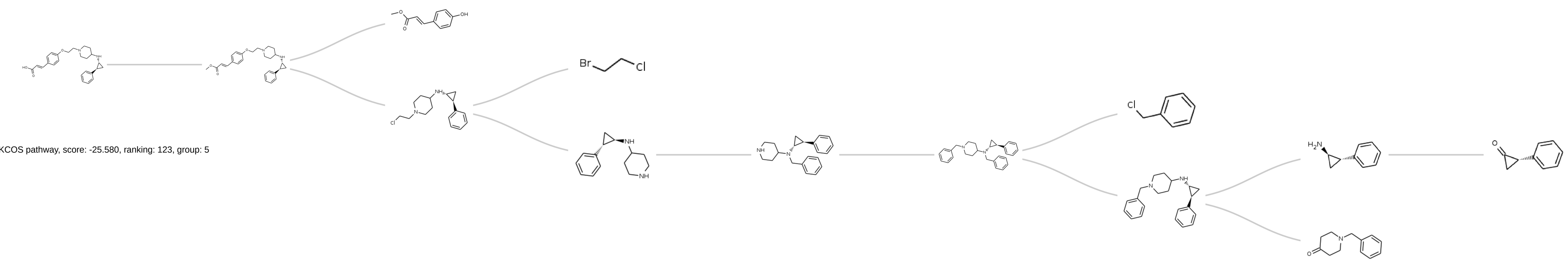

ASKCOS pathway, score: -25.580, ranking: 123, group: 5

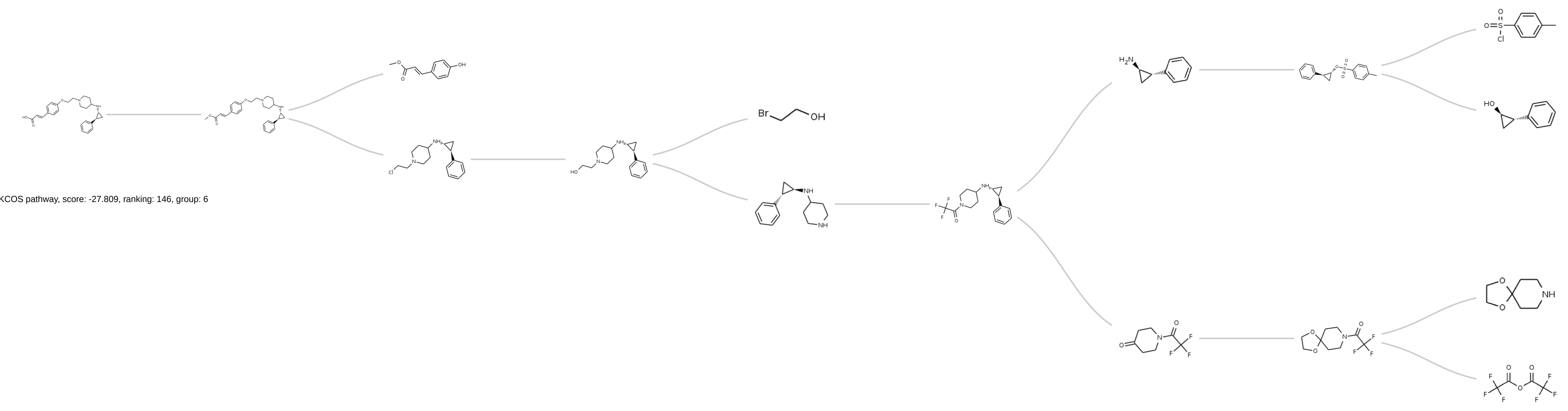

ASKCOS pathway, score: -27.809, ranking: 146, group: 6

Model ranks patent pathway as top-1: Example 49

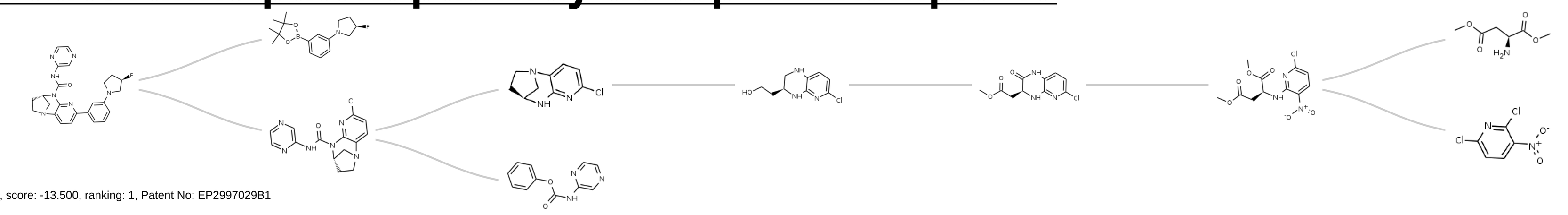

Piatachio pathway, score: -13.500, ranking: 1, Patent No: EP2997029B1

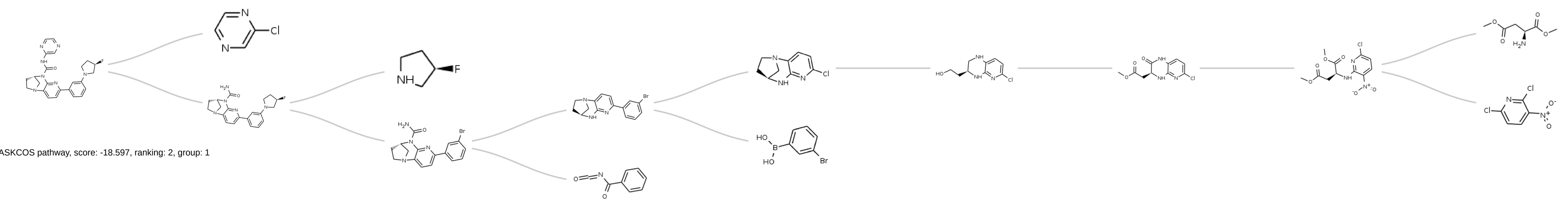

ASKCOS pathway, score: -18.597, ranking: 2, group: 1

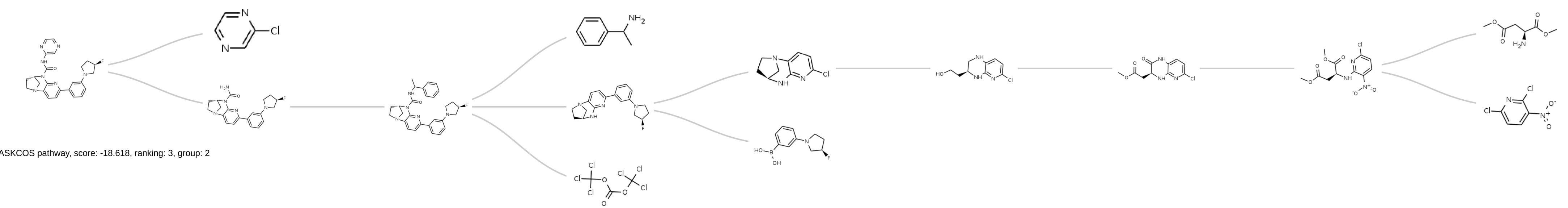

ASKCOS pathway, score: -18.618, ranking: 3, group: 2

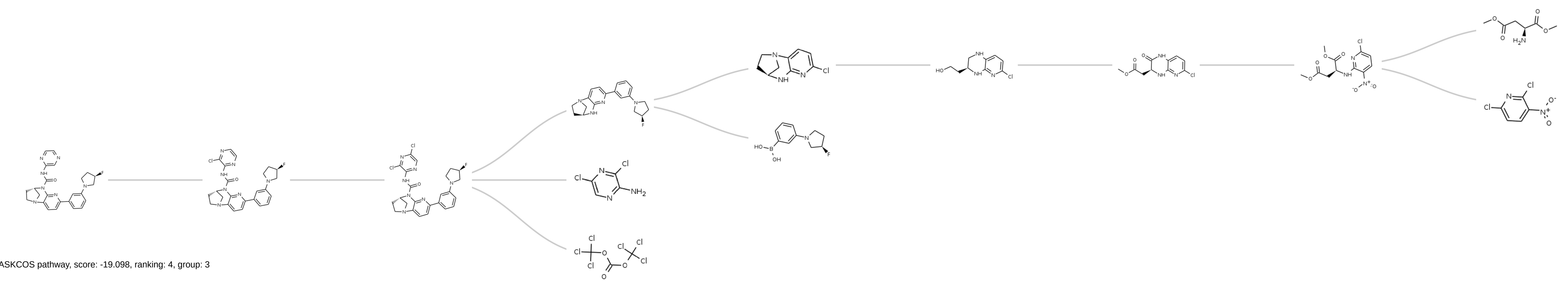

ASKCOS pathway, score: -19.098, ranking: 4, group: 3

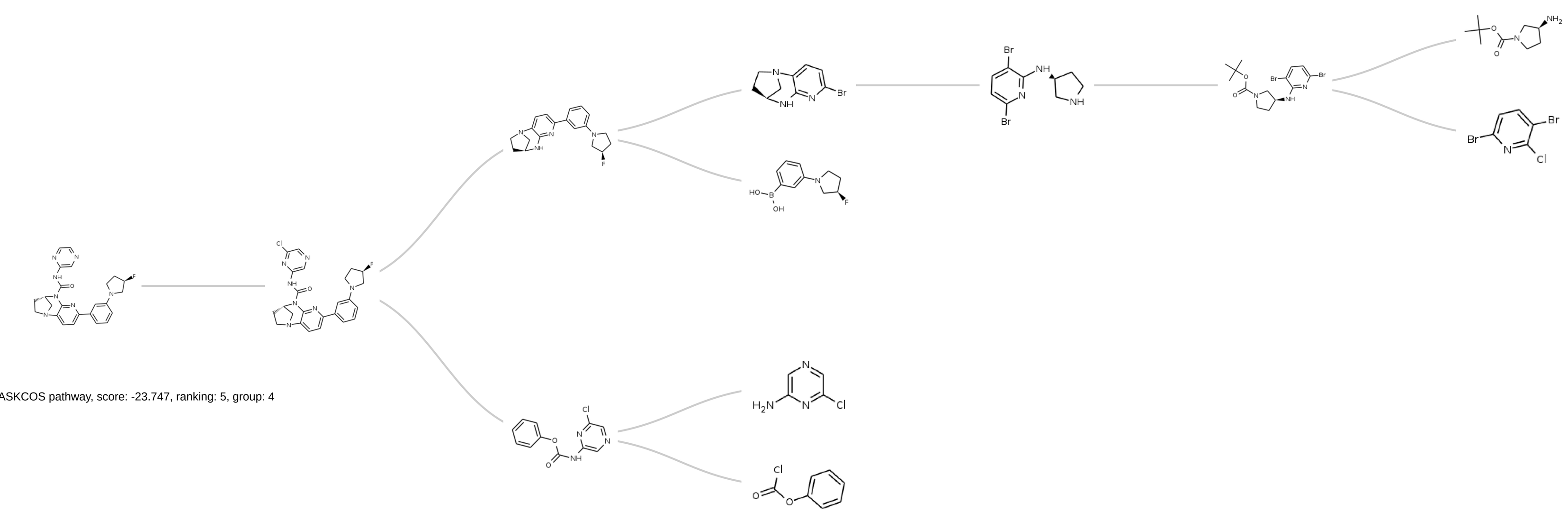

ASKCOS pathway, score: -23.747, ranking: 5, group: 4

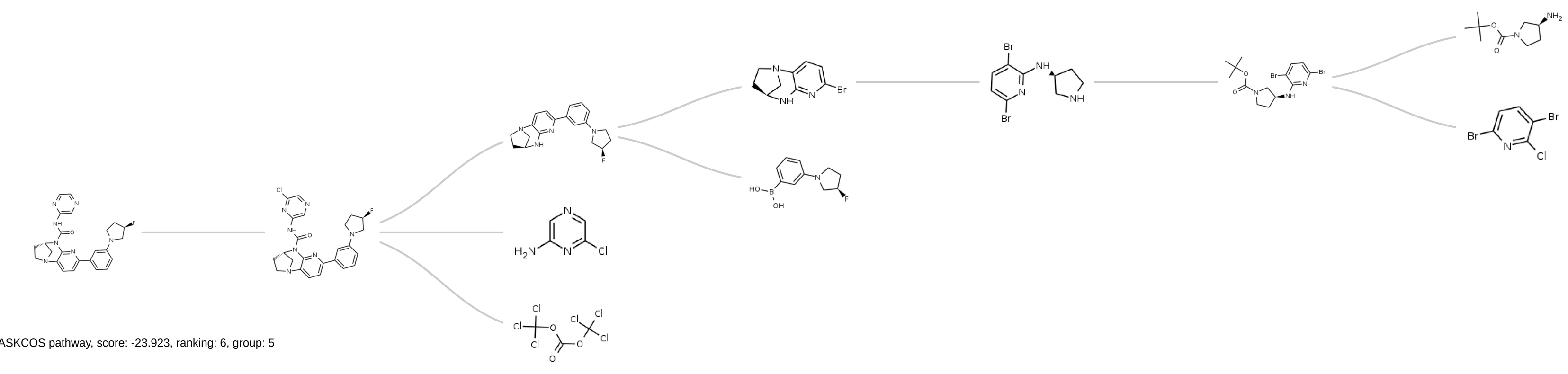

ASKCOS pathway, score: -23.923, ranking: 6, group: 5

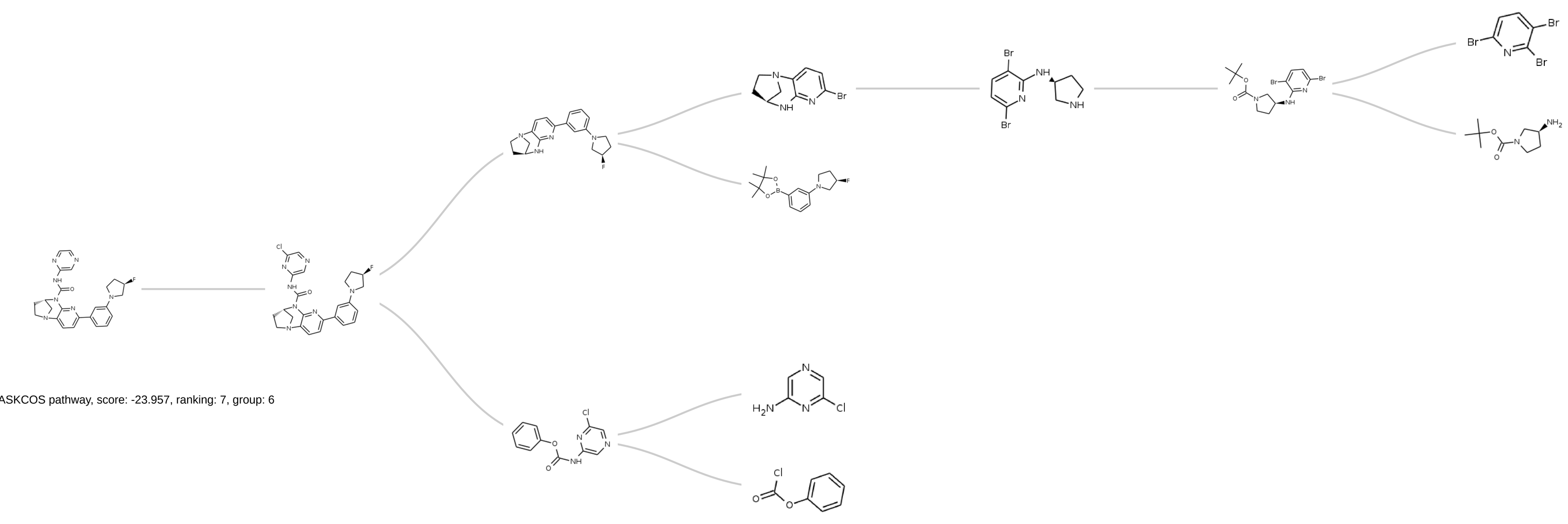

ASKCOS pathway, score: -23.957, ranking: 7, group: 6

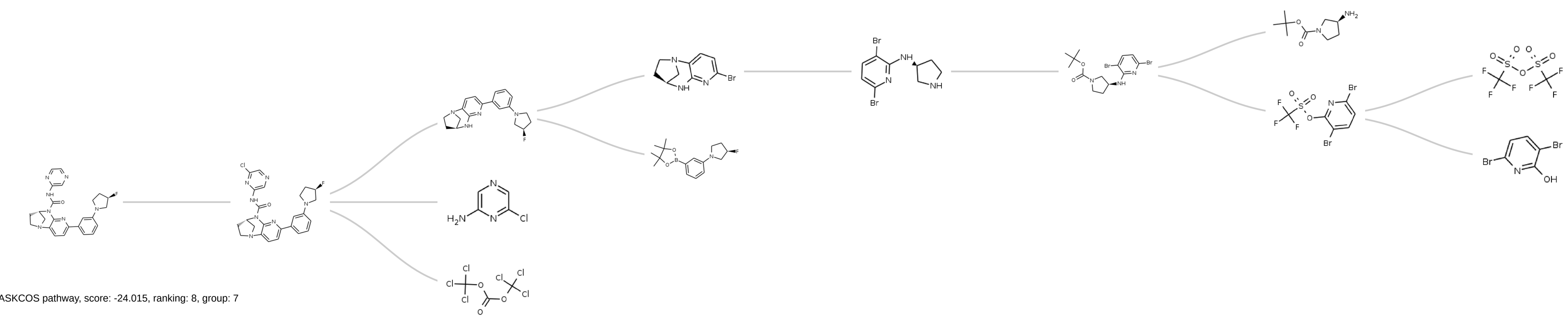

ASKCOS pathway, score: -24.015, ranking: 8, group: 7

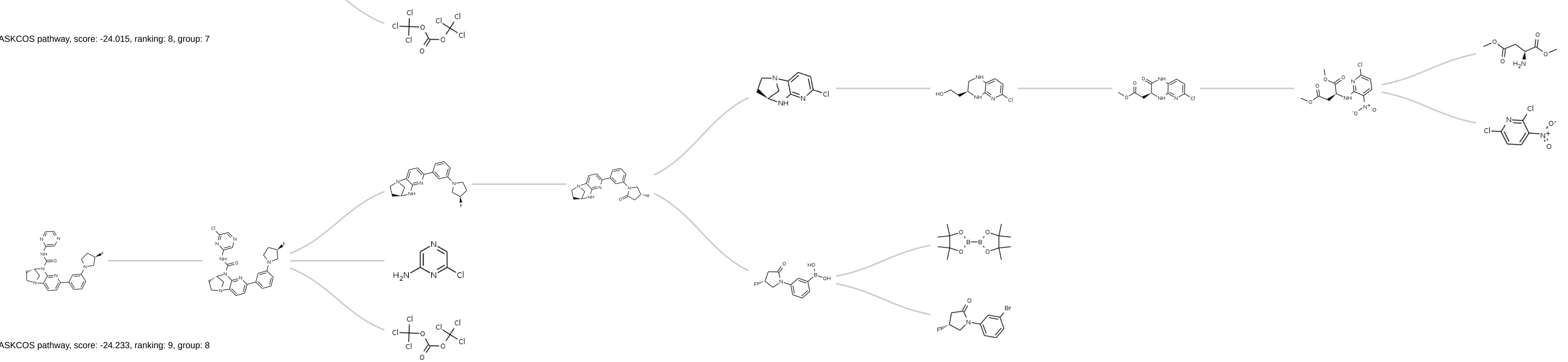

ASKCOS pathway, score: -24.233, ranking: 9, group: 8

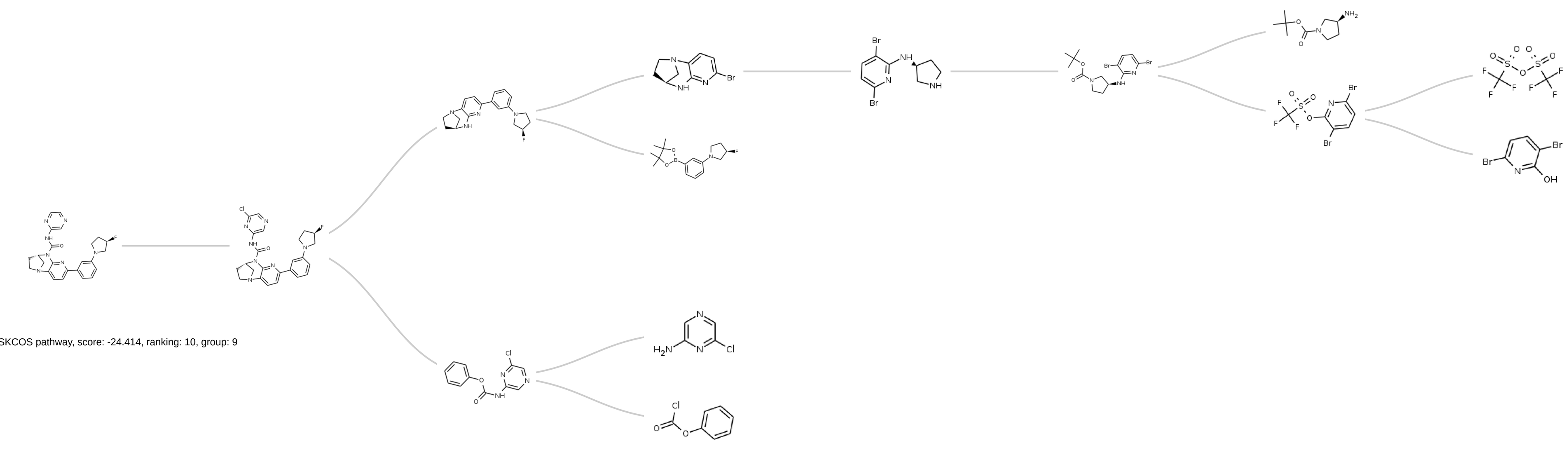

ASKCOS pathway, score: -24.414, ranking: 10, group: 9

Model ranks patent pathway as top-1: Example 50

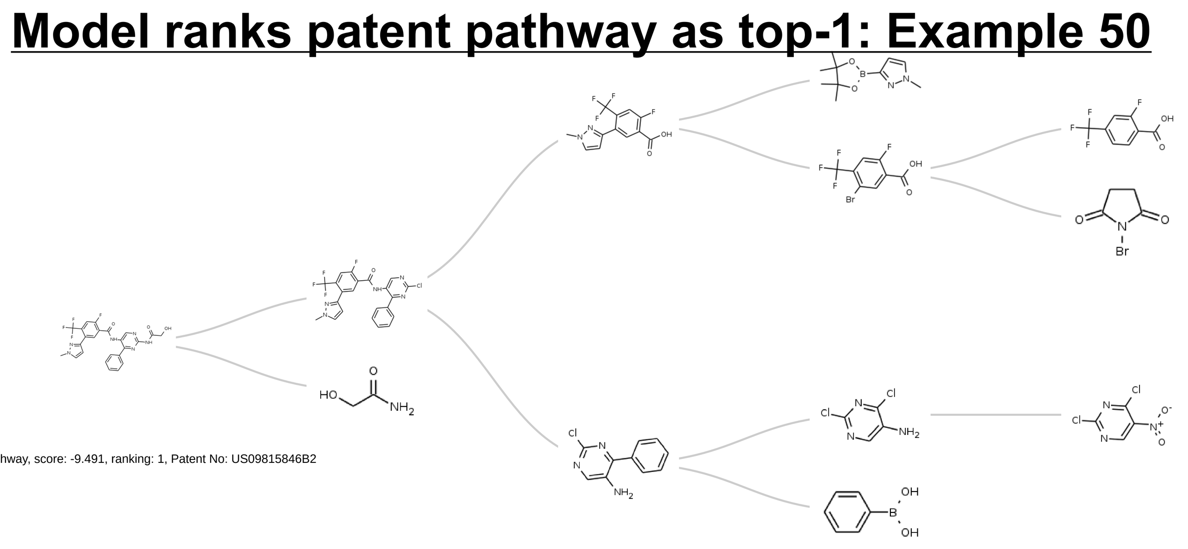

Piatachio pathway, score: -9.491, ranking: 1, Patent No: US09815846B2

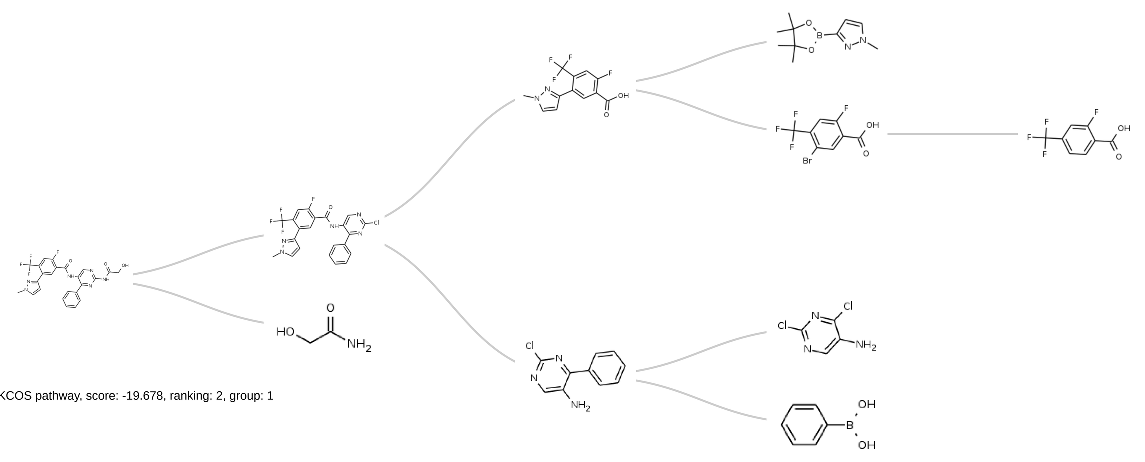

ASKCOS pathway, score: -19.678, ranking: 2, group: 1

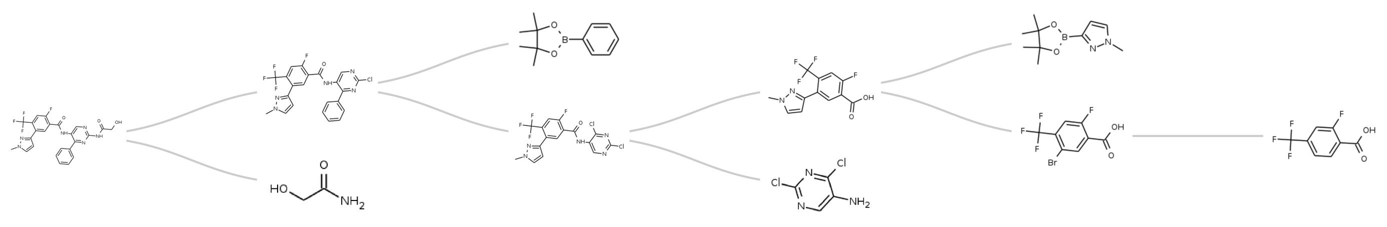

ASKCOS pathway, score: -21.041, ranking: 3, group: 2

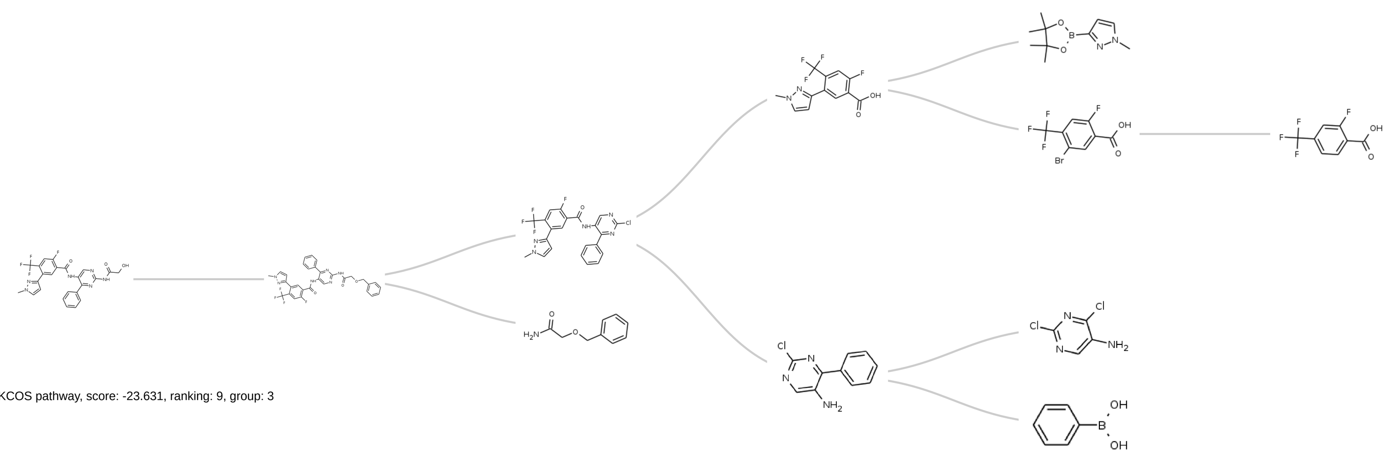

ASKCOS pathway, score: -23.631, ranking: 9, group: 3

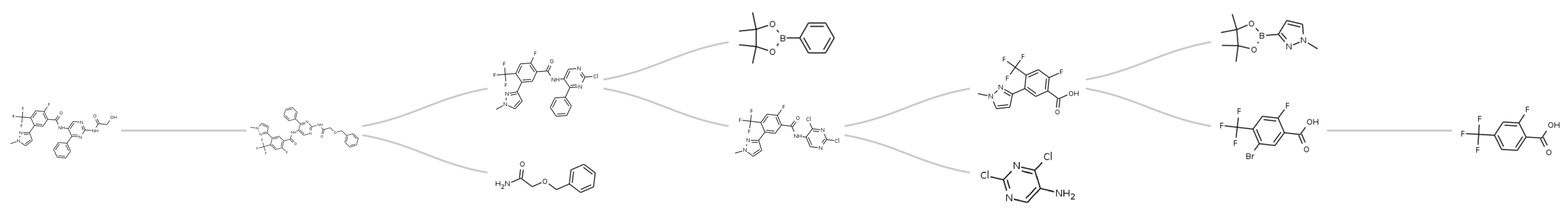

ASKCOS pathway, score: -24.042, ranking: 13, group: 4

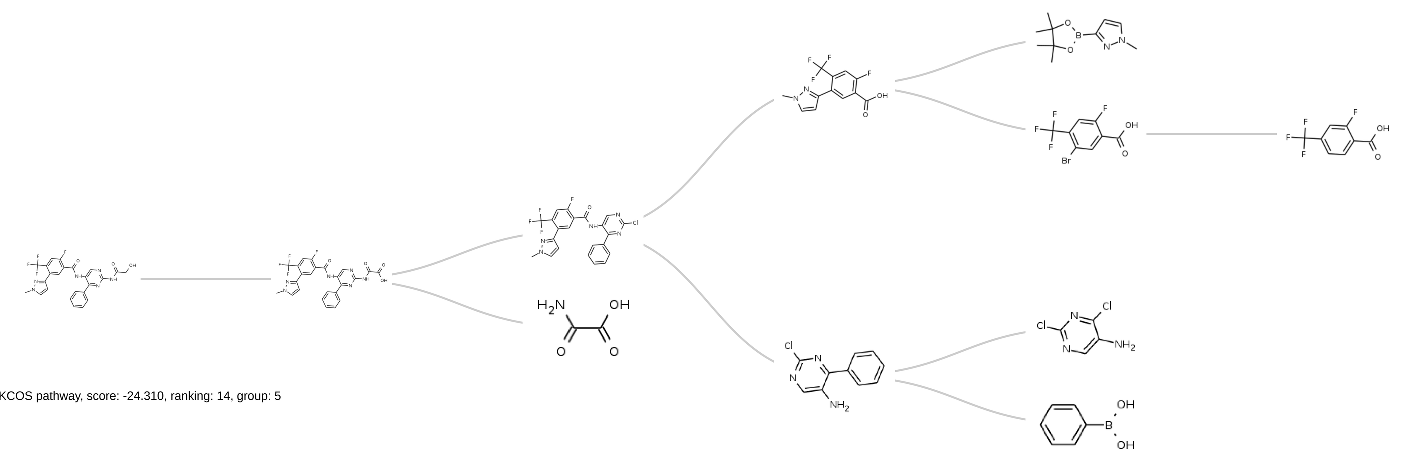

ASKCOS pathway, score: -24.310, ranking: 14, group: 5

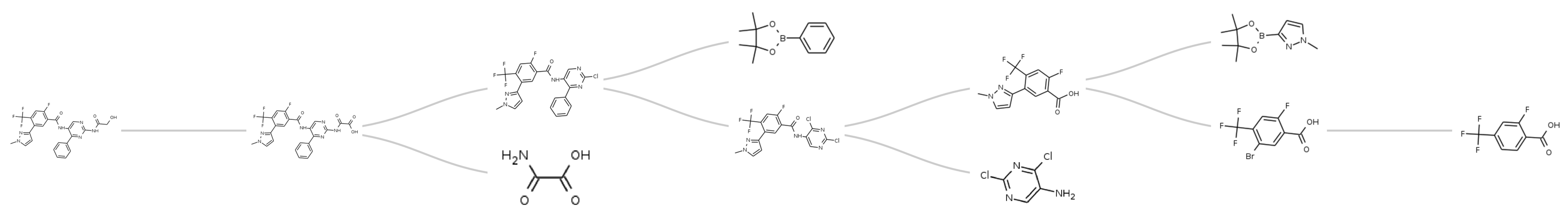

ASKCOS pathway, score: -24.531, ranking: 17, group: 6

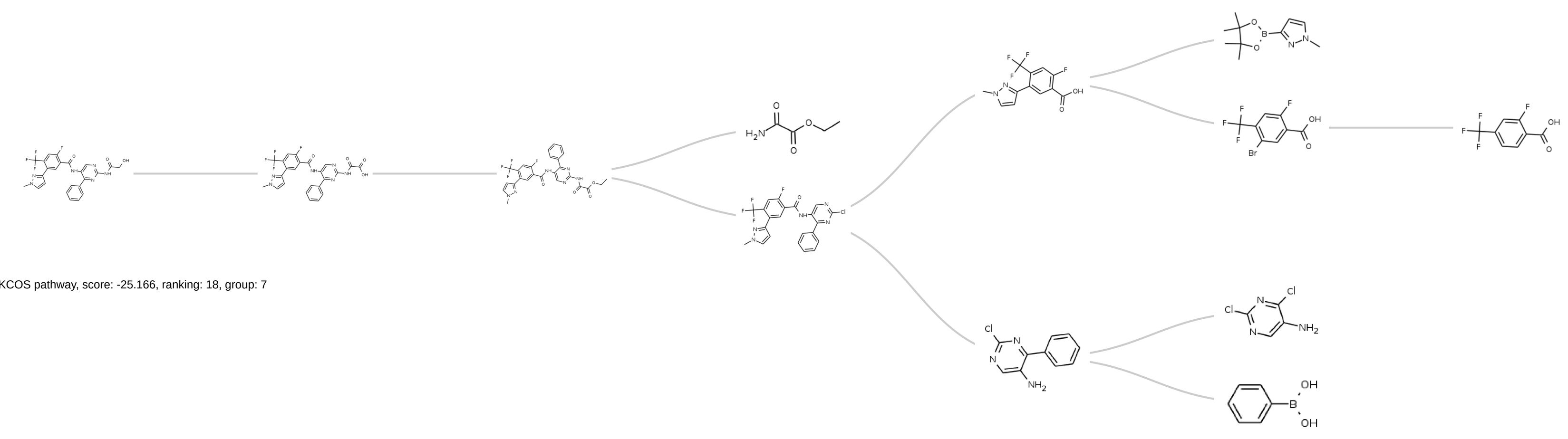

ASKCOS pathway, score: -25.166, ranking: 18, group: 7

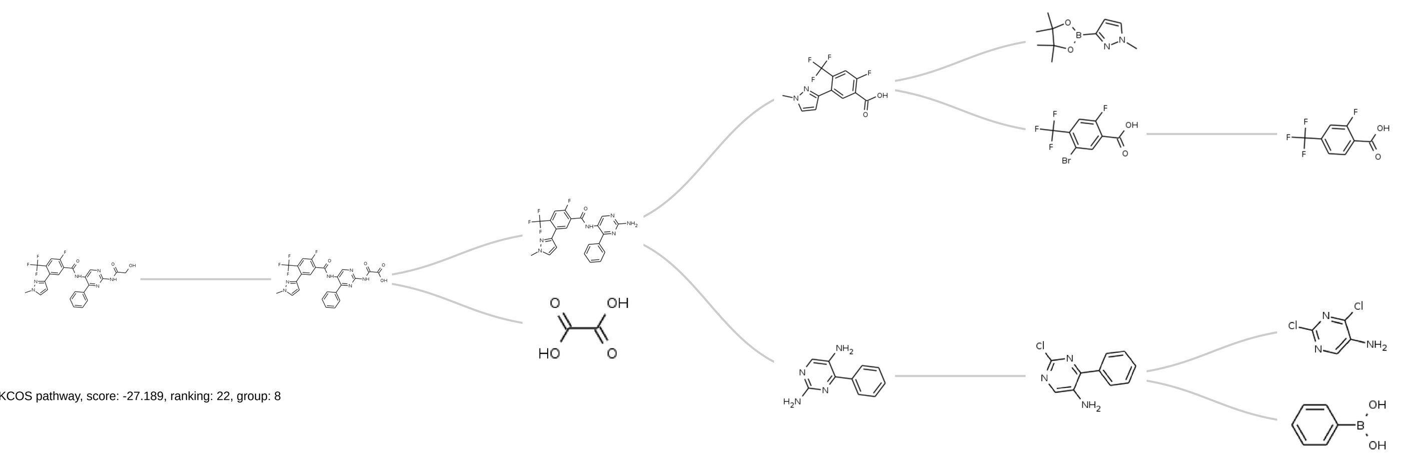

ASKCOS pathway, score: -27.189, ranking: 22, group: 8

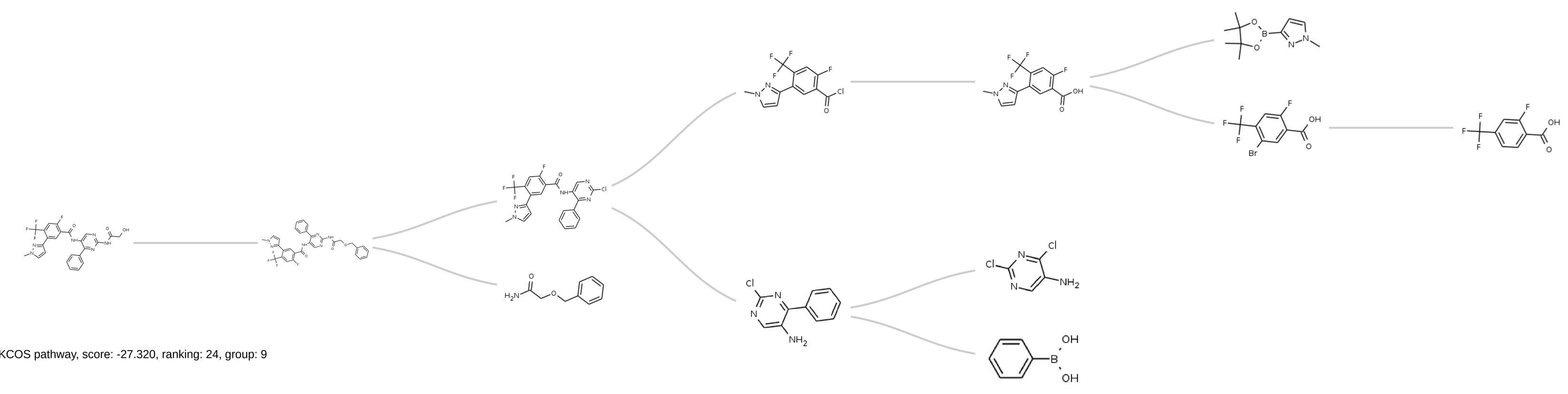

ASKCOS pathway, score: -27.320, ranking: 24, group: 9

Model ranks patent pathway not as top-1: Example 1

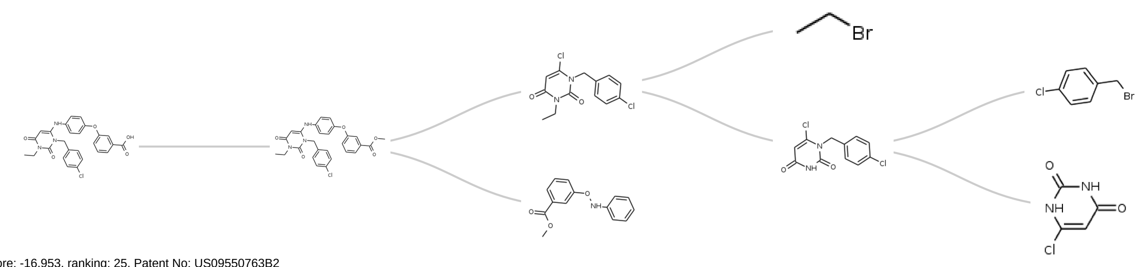

Piatachio pathway, score: -16.953, ranking: 25, Patent No: US09550763B2

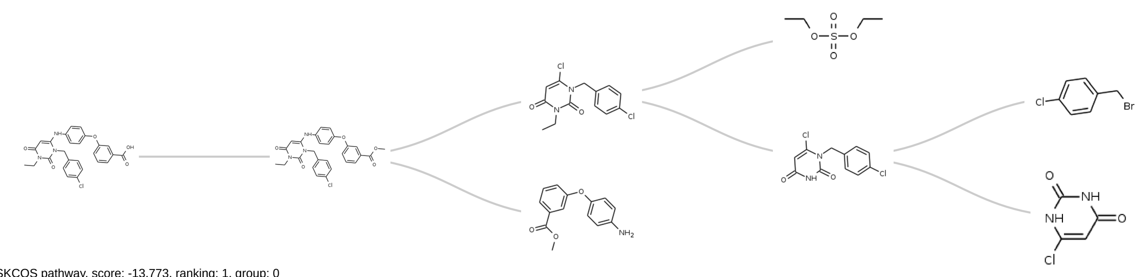

ASKCOS pathway, score: -13.773, ranking: 1, group: 0

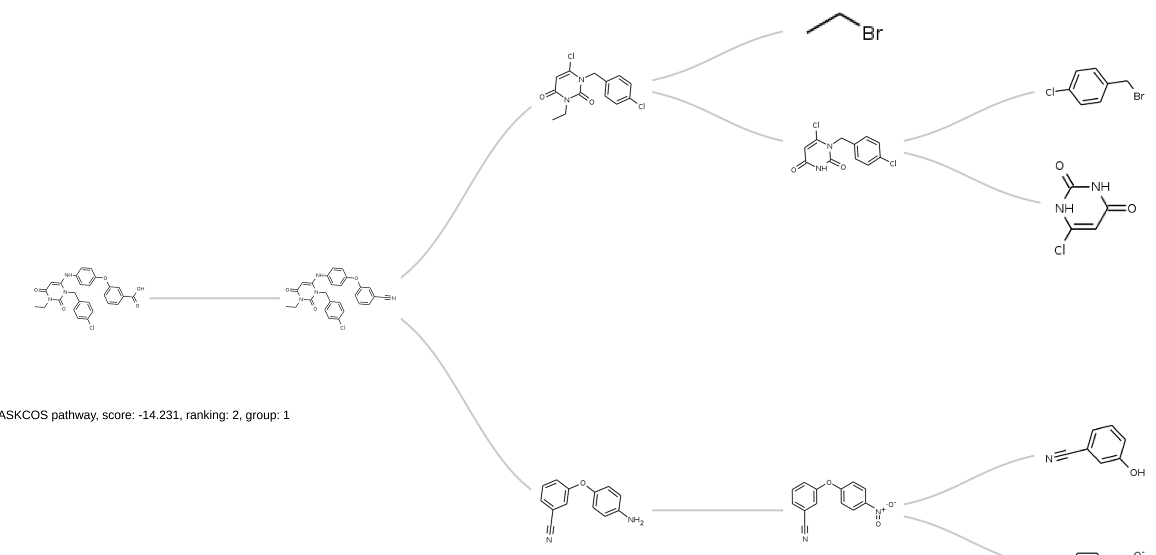

ASKCOS pathway, score: -14.231, ranking: 2, group: 1

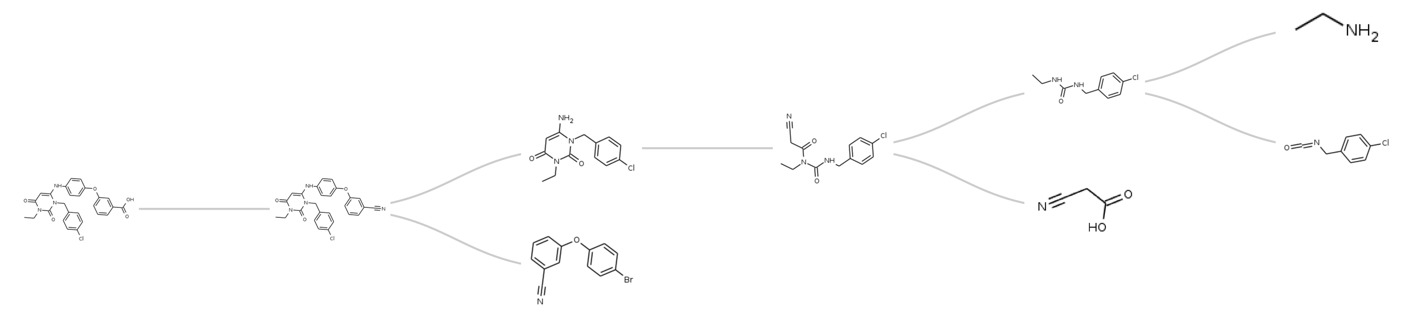

ASKCOS pathway, score: -14.984, ranking: 6, group: 2

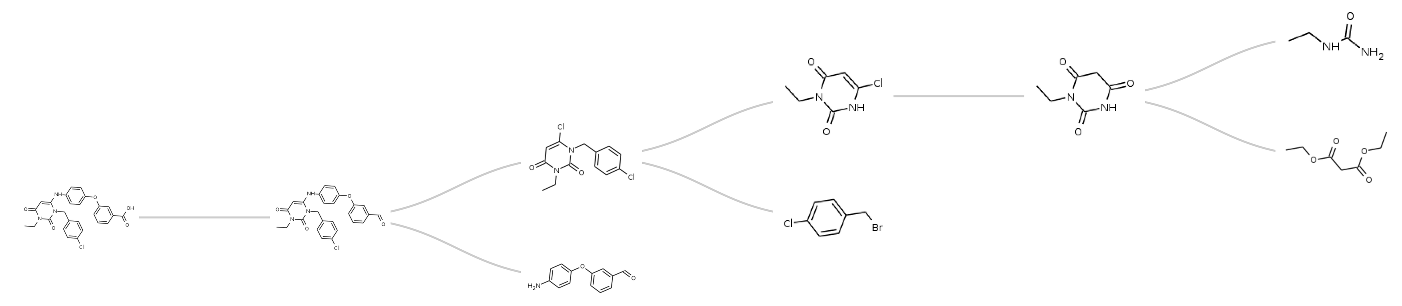

ASKCOS pathway, score: -15.251, ranking: 10, group: 3

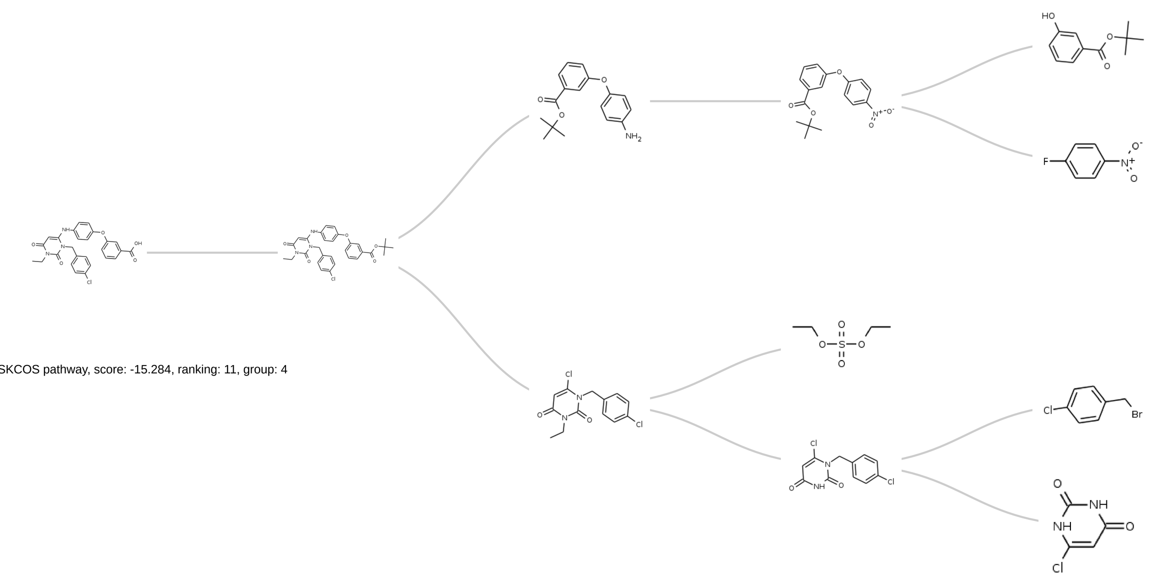

ASKCOS pathway, score: -15.284, ranking: 11, group: 4

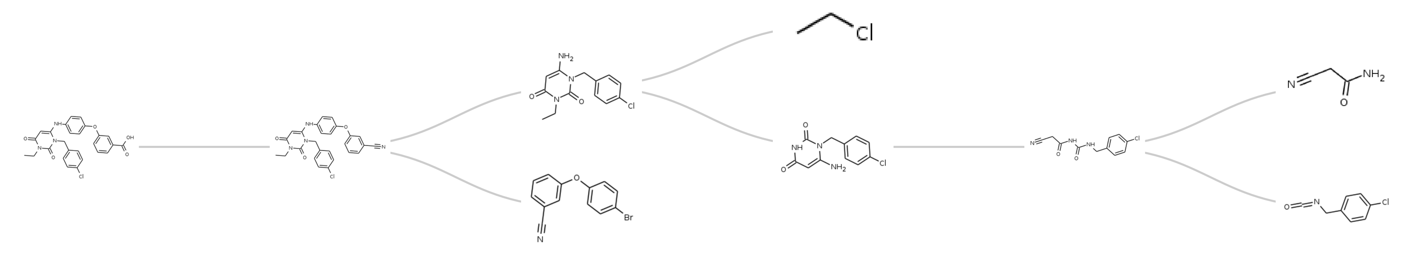

ASKCOS pathway, score: -15.882, ranking: 15, group: 5

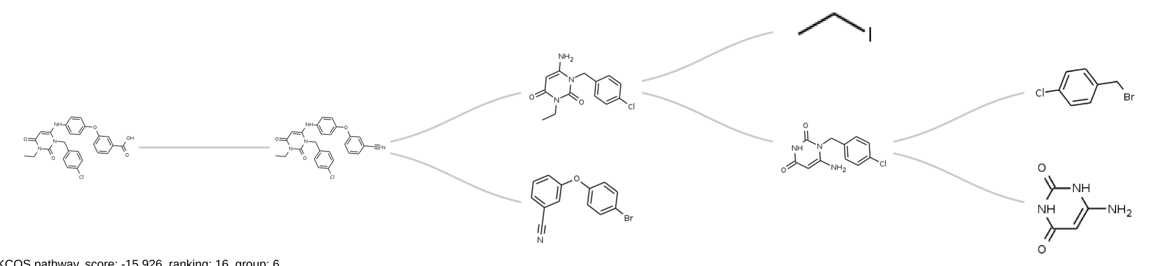

ASKCOS pathway, score: -15.926, ranking: 16, group: 6

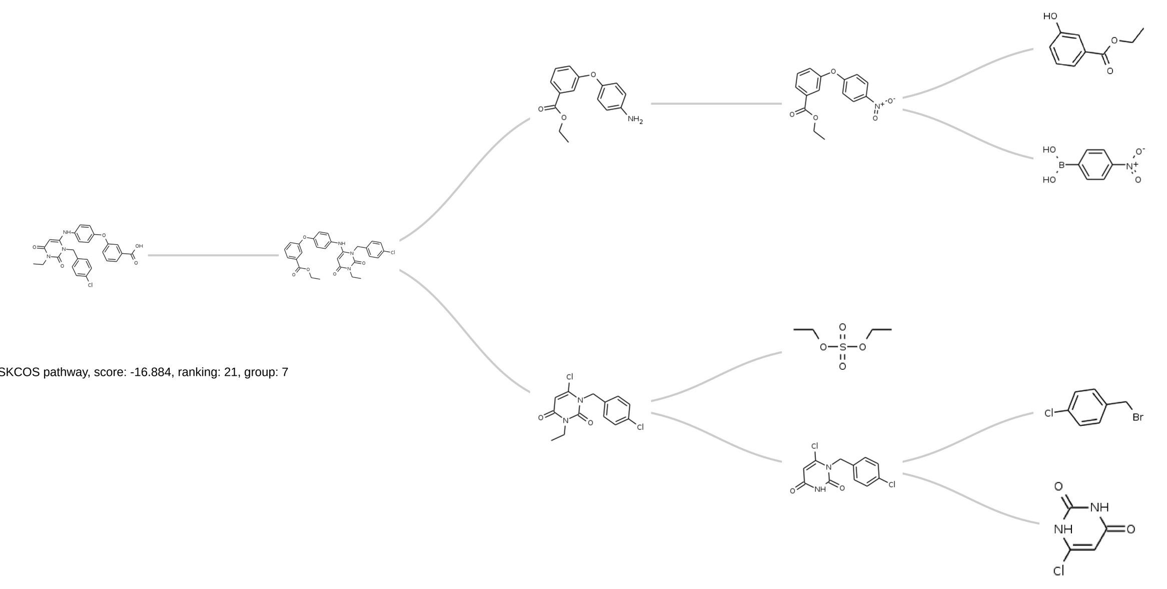

ASKCOS pathway, score: -16.884, ranking: 21, group: 7

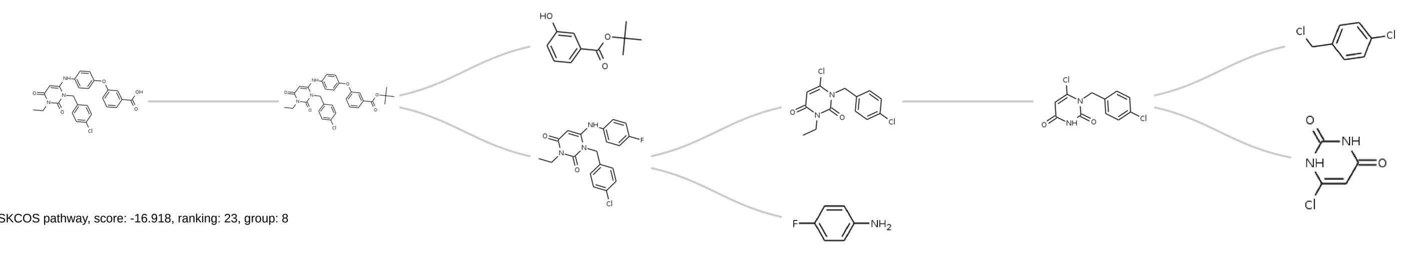

ASKCOS pathway, score: -16.918, ranking: 23, group: 8

Model ranks patent pathway not as top-1: Example 2

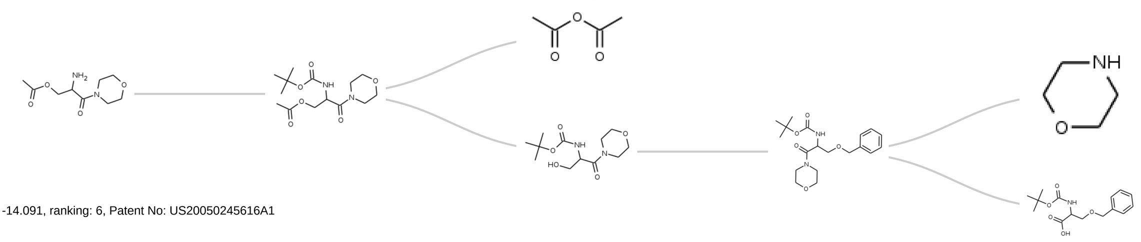

Piatachio pathway, score: -14.091, ranking: 6, Patent No: US20050245616A1

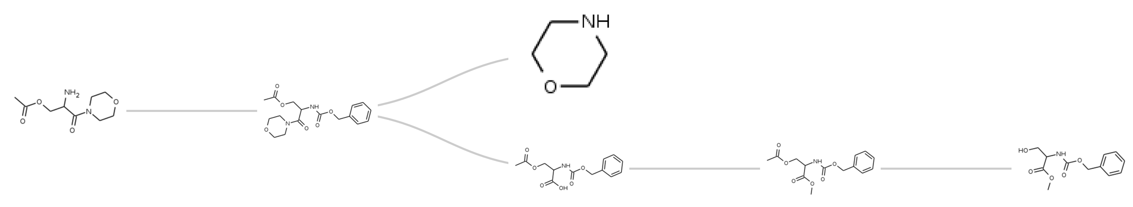

ASKCOS pathway, score: -12.507, ranking: 1, group: 0

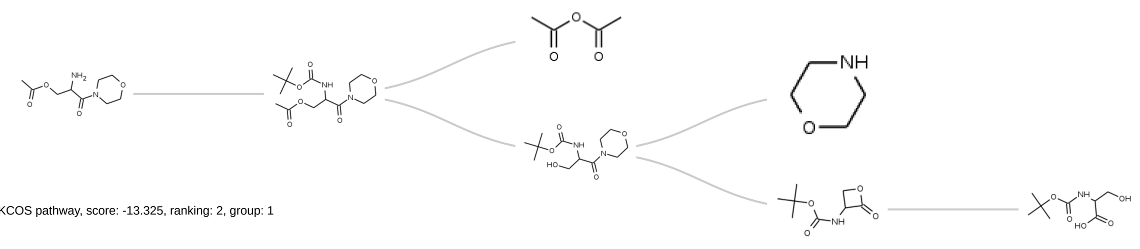

ASKCOS pathway, score: -13.325, ranking: 2, group: 1

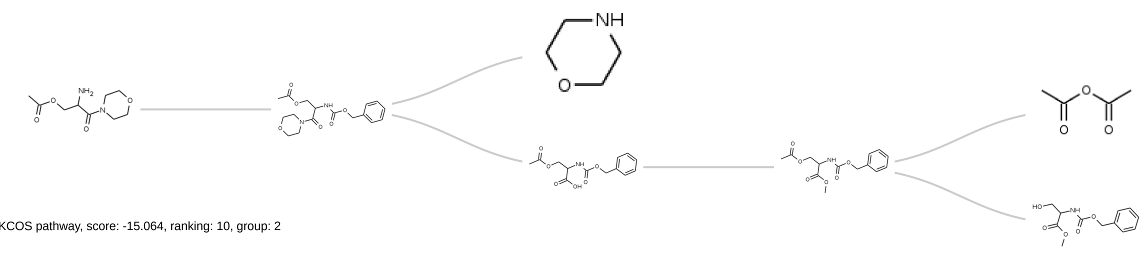

ASKCOS pathway, score: -15.064, ranking: 10, group: 2

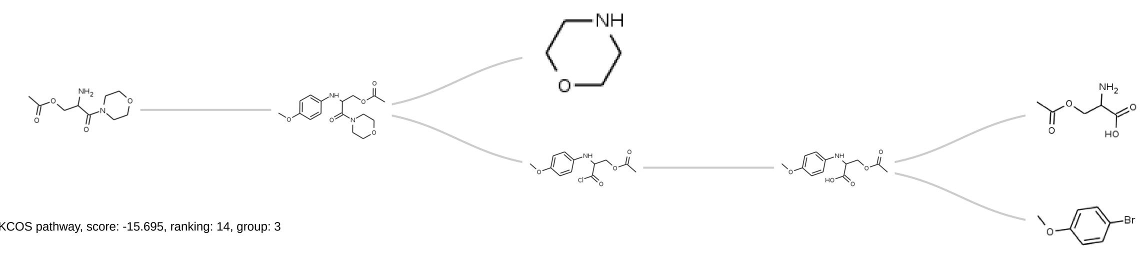

ASKCOS pathway, score: -15.695, ranking: 14, group: 3

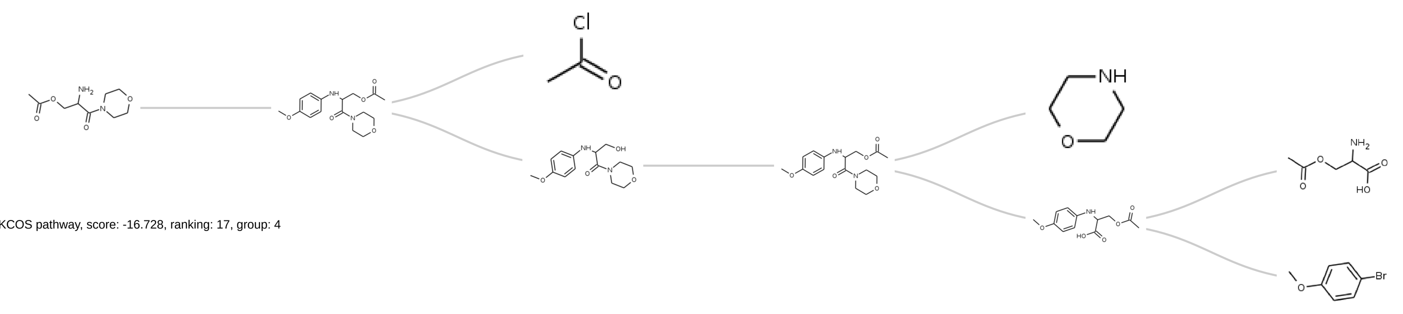

ASKCOS pathway, score: -16.728, ranking: 17, group: 4

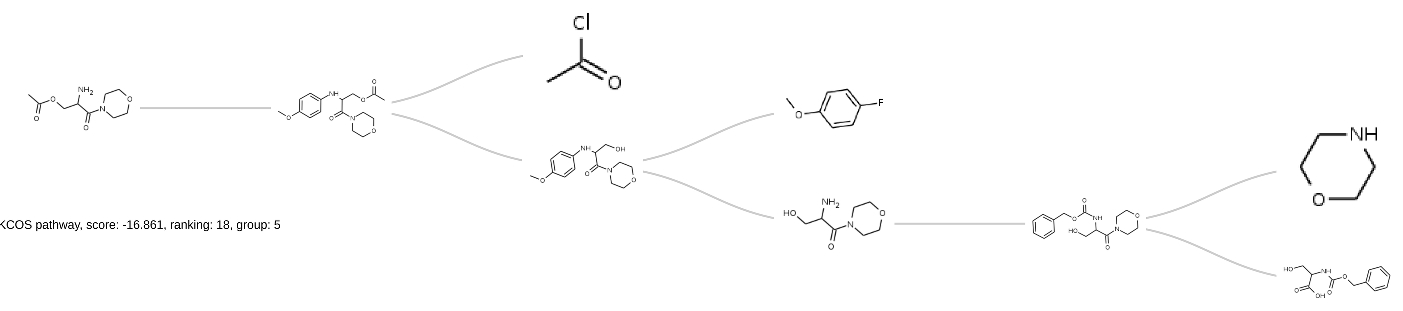

ASKCOS pathway, score: -16.861, ranking: 18, group: 5

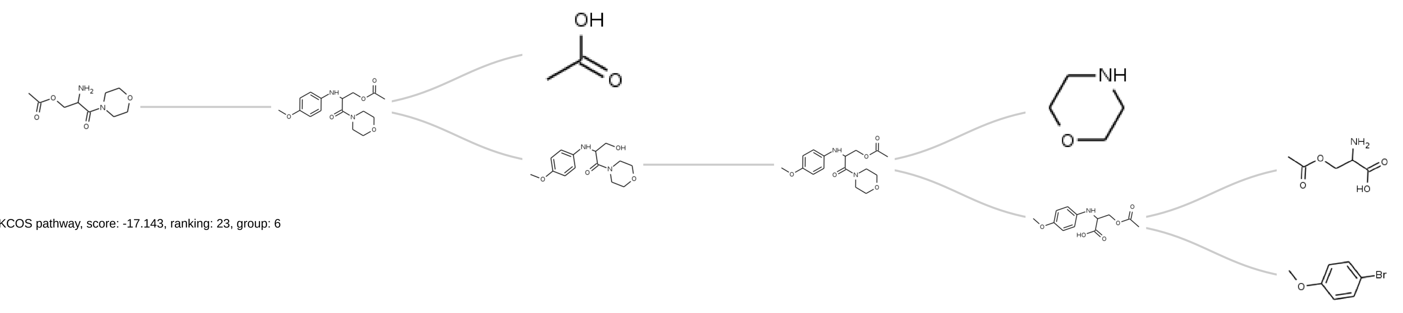

ASKCOS pathway, score: -17.143, ranking: 23, group: 6

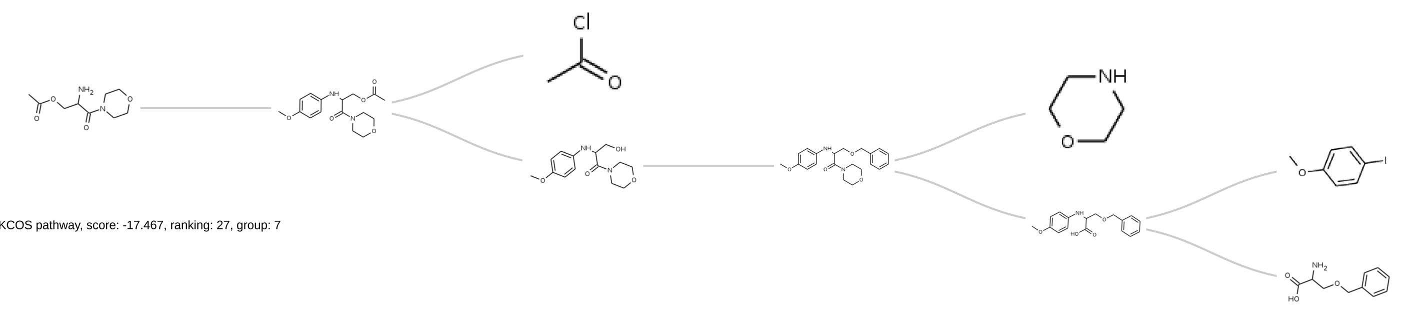

ASKCOS pathway, score: -17.467, ranking: 27, group: 7

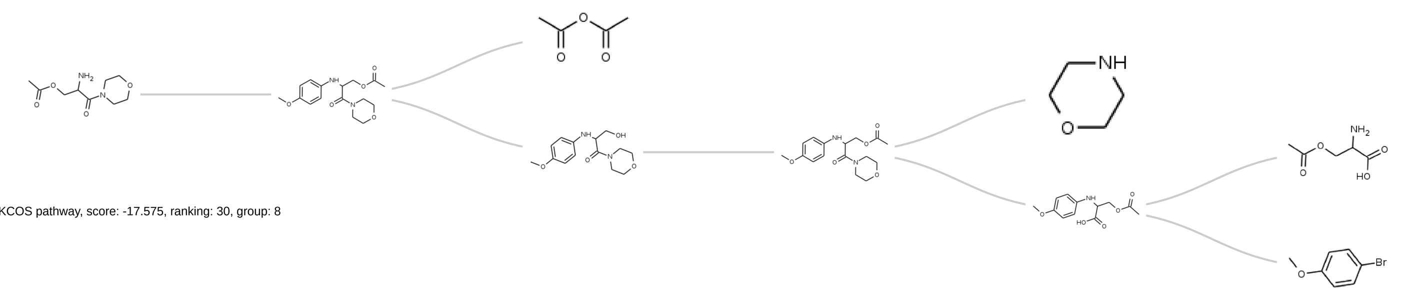

ASKCOS pathway, score: -17.575, ranking: 30, group: 8

Model ranks patent pathway not as top-1: Example 3

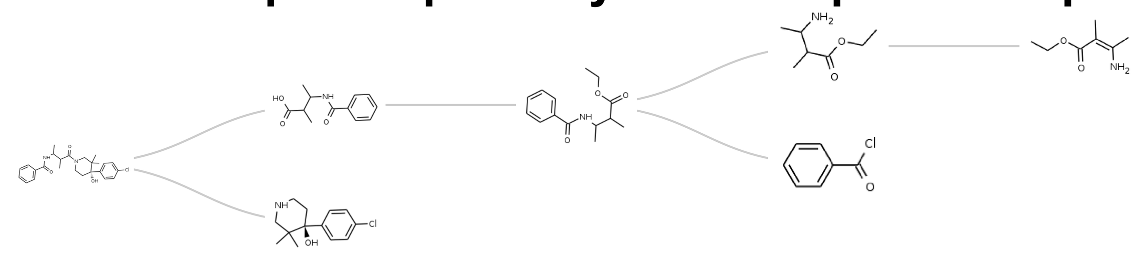

Piatachio pathway, score: -11.511, ranking: 2, Patent No: US08501782B2

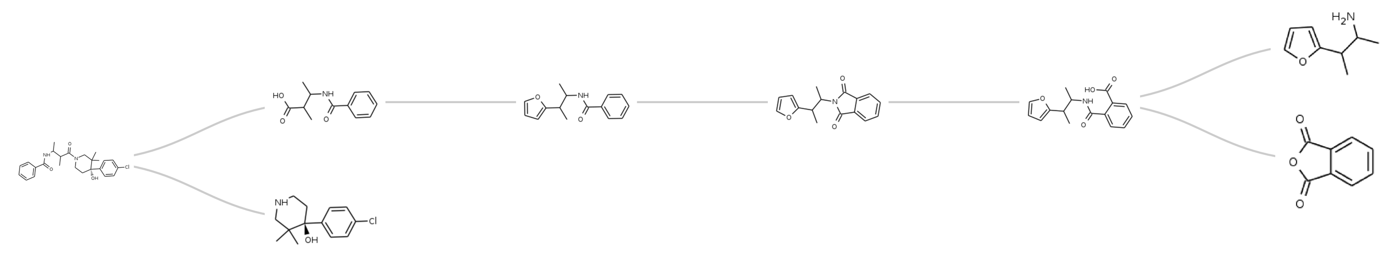

ASKCOS pathway, score: -8.654, ranking: 1, group: 0

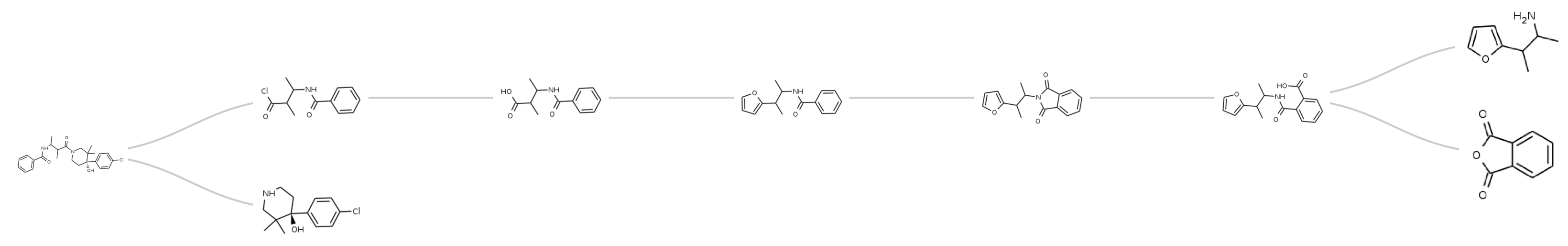

ASKCOS pathway, score: -11.728, ranking: 3, group: 2

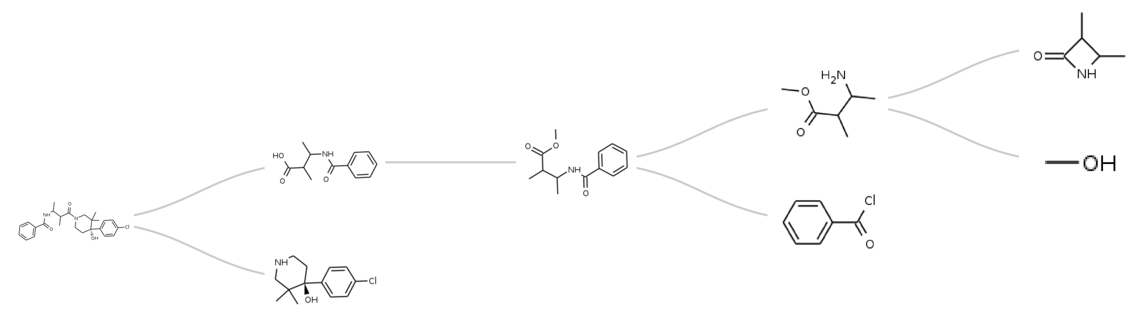

ASKCOS pathway, score: -11.986, ranking: 4, group: 3

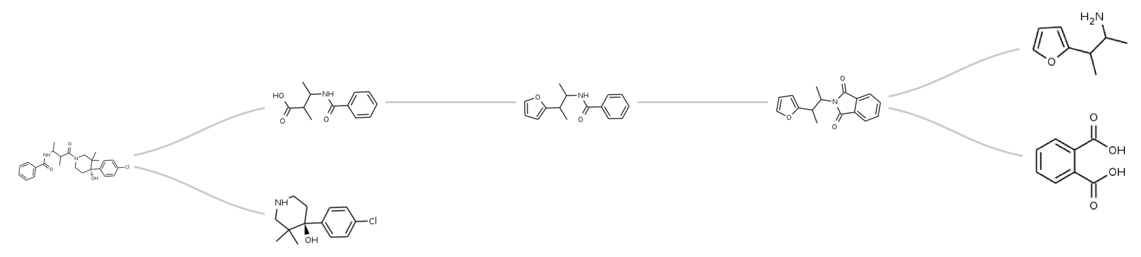

ASKCOS pathway, score: -12.242, ranking: 5, group: 4

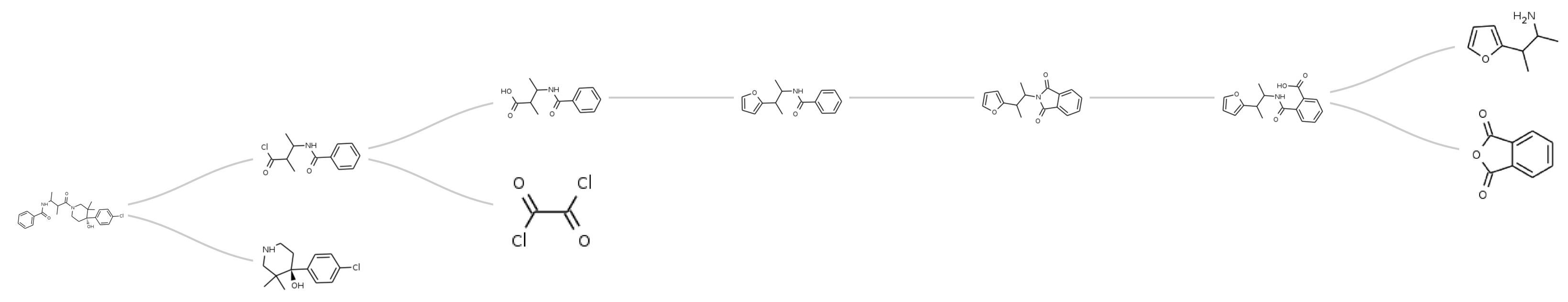

ASKCOS pathway, score: -12.853, ranking: 6, group: 5

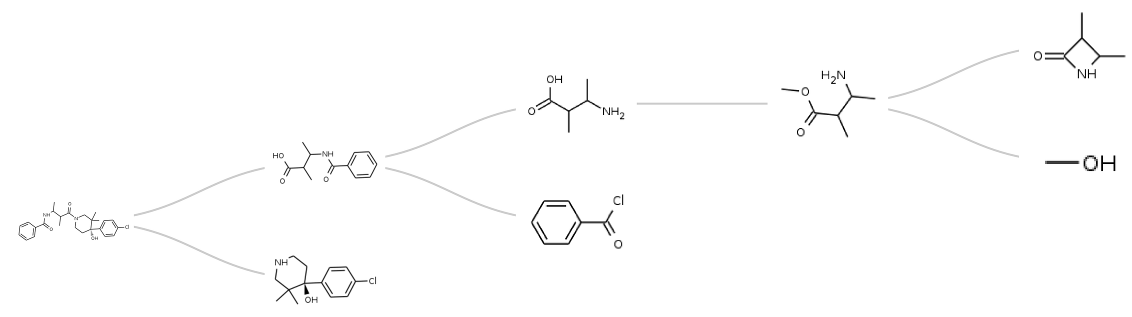

ASKCOS pathway, score: -14.289, ranking: 9, group: 6

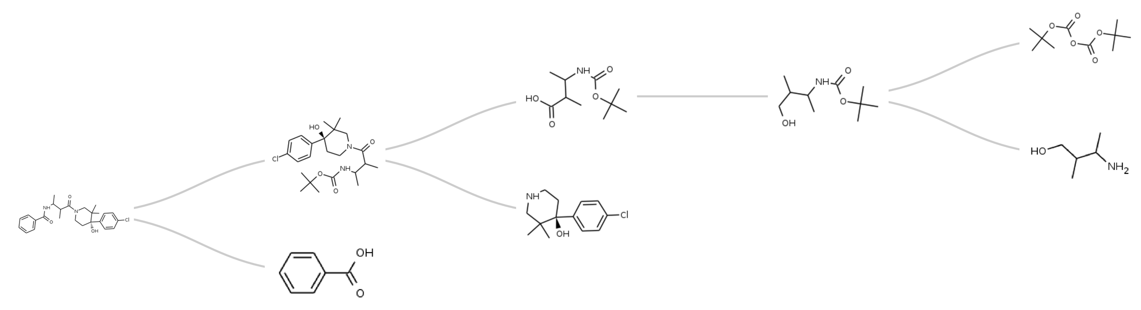

ASKCOS pathway, score: -14.487, ranking: 11, group: 7

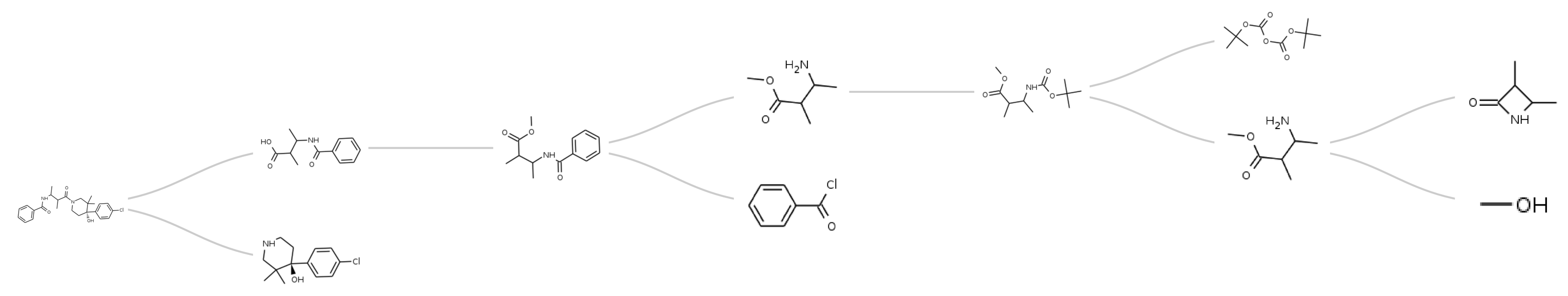

ASKCOS pathway, score: -15.795, ranking: 23, group: 8

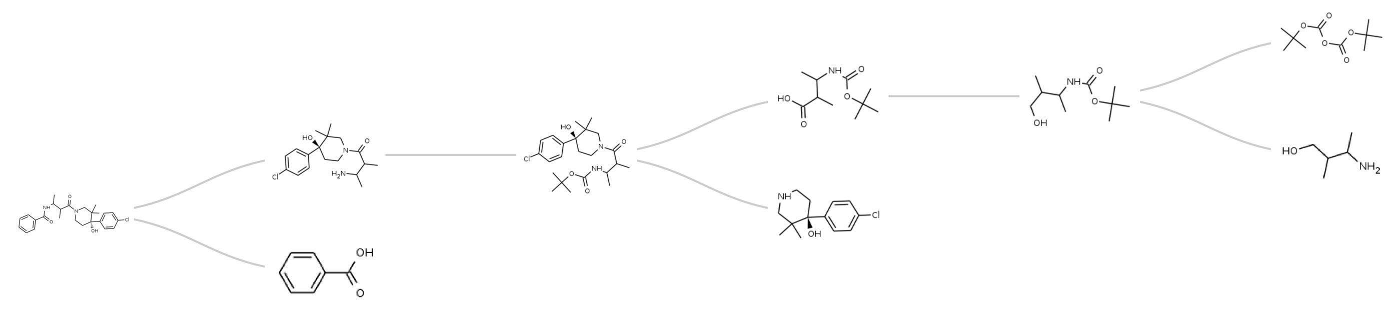

ASKCOS pathway, score: -16.159, ranking: 32, group: 9

Model ranks patent pathway not as top-1: Example 4

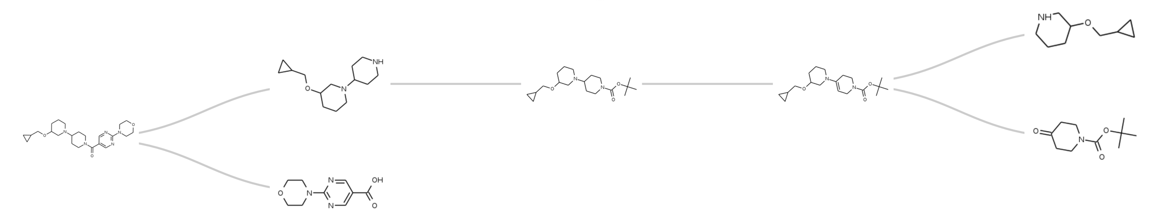

Piatachio pathway, score: -14.512, ranking: 21, Patent No: US09624199B2

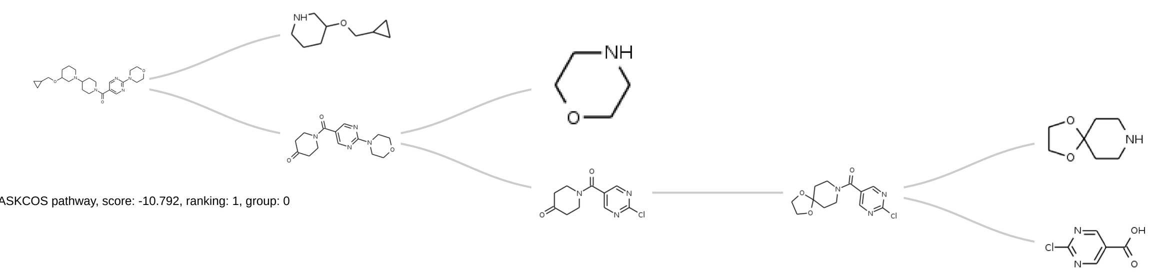

ASKCOS pathway, score: -10.792, ranking: 1, group: 0

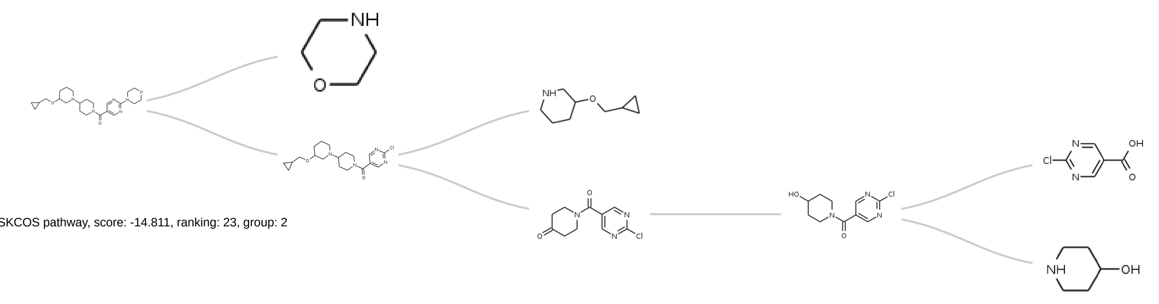

ASKCOS pathway, score: -14.811, ranking: 23, group: 2

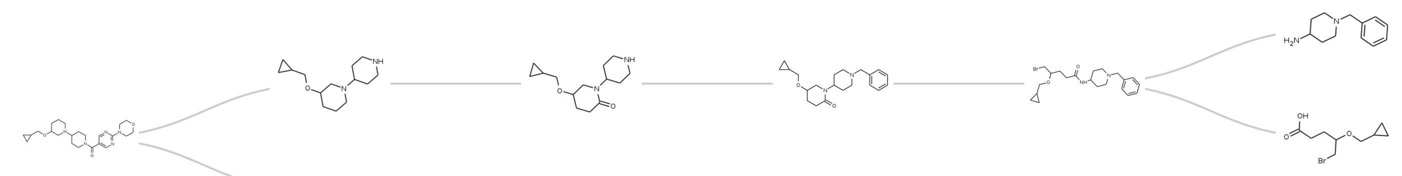

ASKCOS pathway, score: -15.423, ranking: 26, group: 3

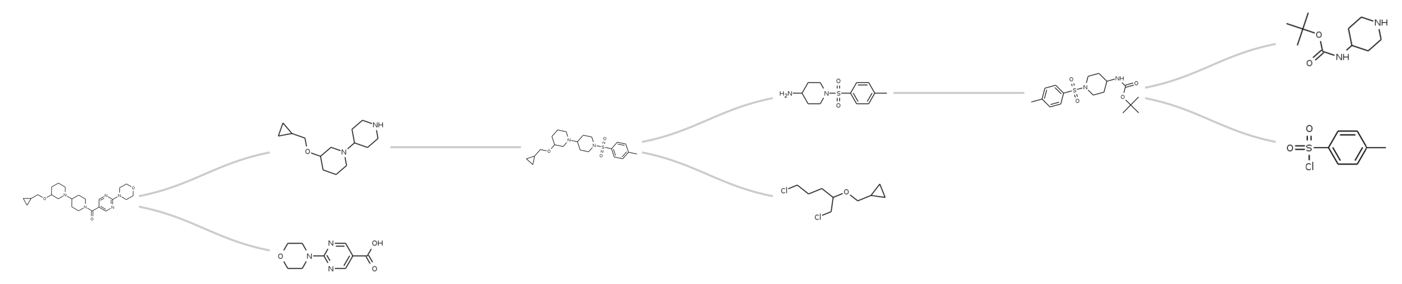

ASKCOS pathway, score: -15.627, ranking: 27, group: 4

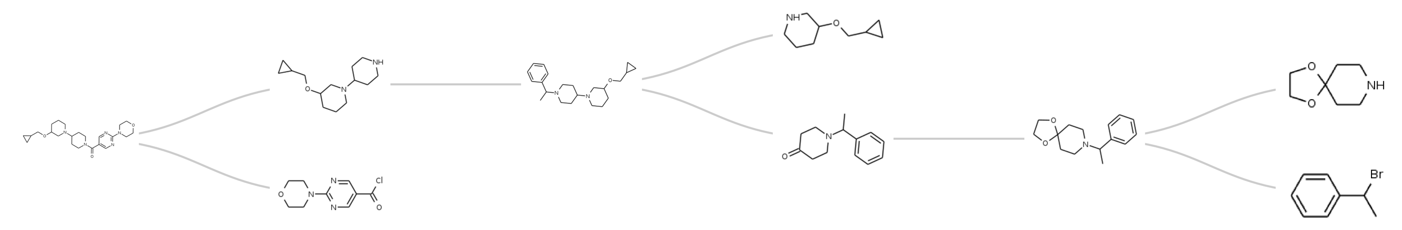

ASKCOS pathway, score: -16.227, ranking: 30, group: 5

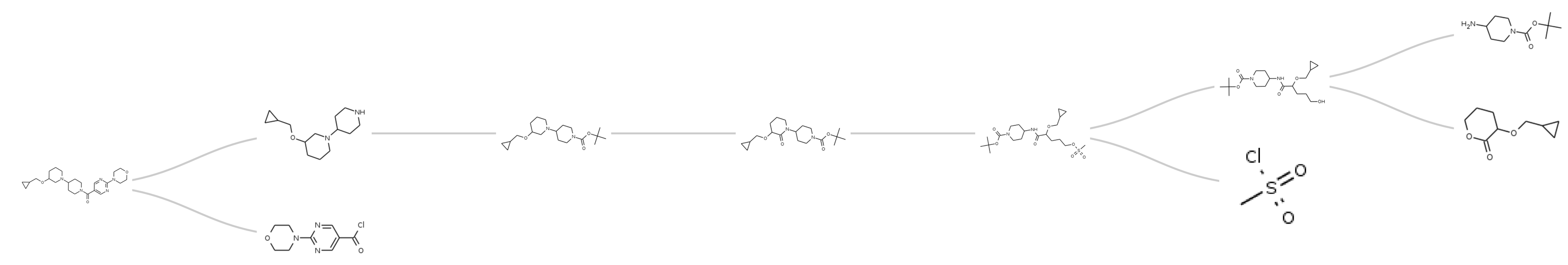

ASKCOS pathway, score: -16.512, ranking: 31, group: 6

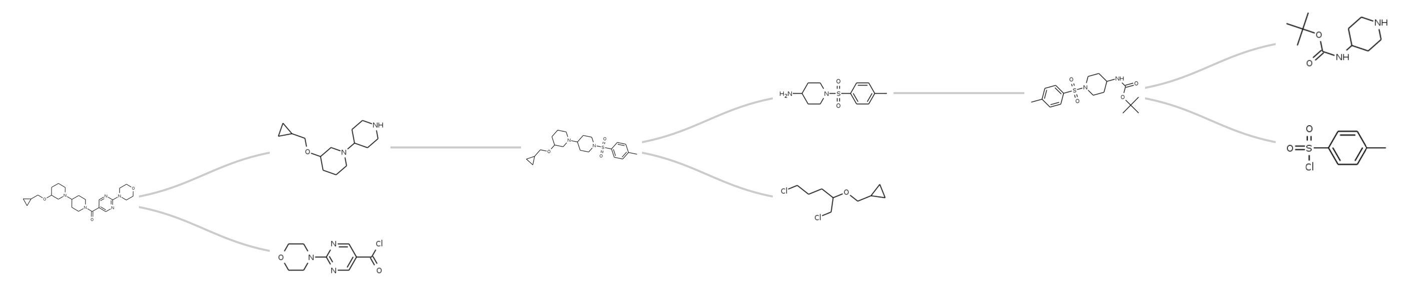

ASKCOS pathway, score: -16.710, ranking: 33, group: 7

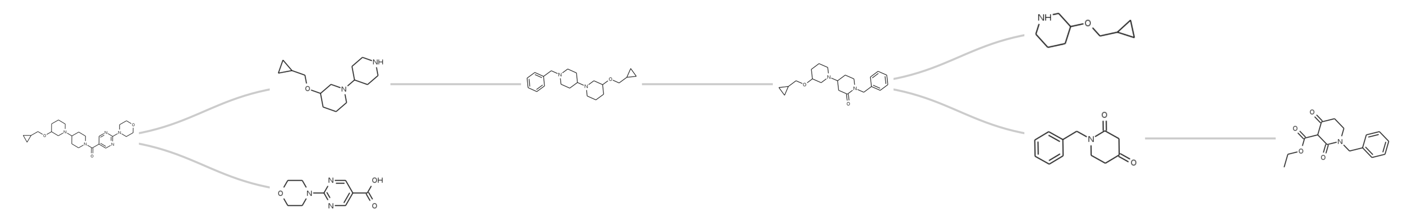

ASKCOS pathway, score: -17.024, ranking: 40, group: 8

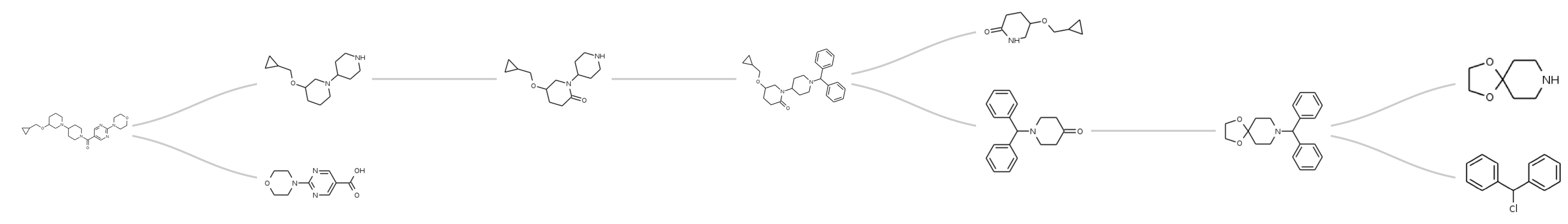

ASKCOS pathway, score: -17.975, ranking: 51, group: 9

Model ranks patent pathway not as top-1: Example 5

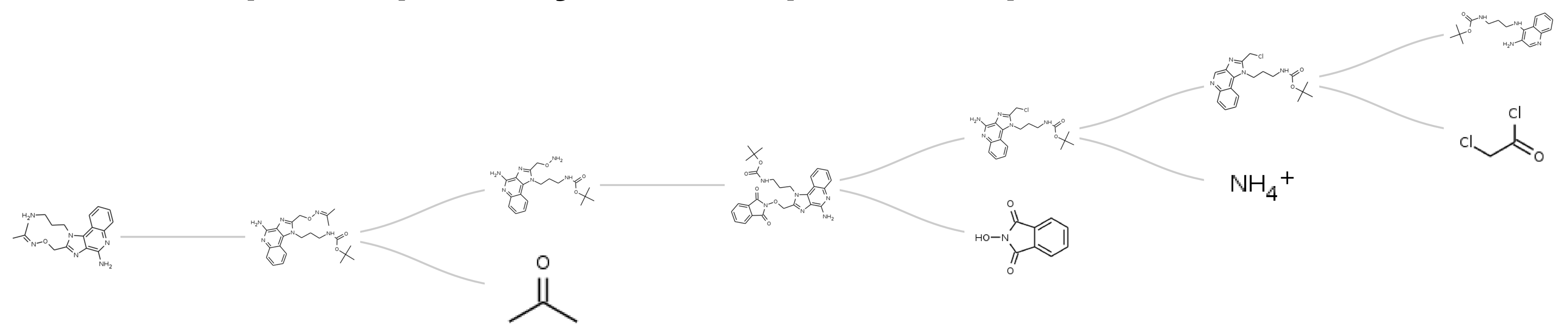

Piatachio pathway, score: -16.024, ranking: 2, Patent No: US20090042925A1

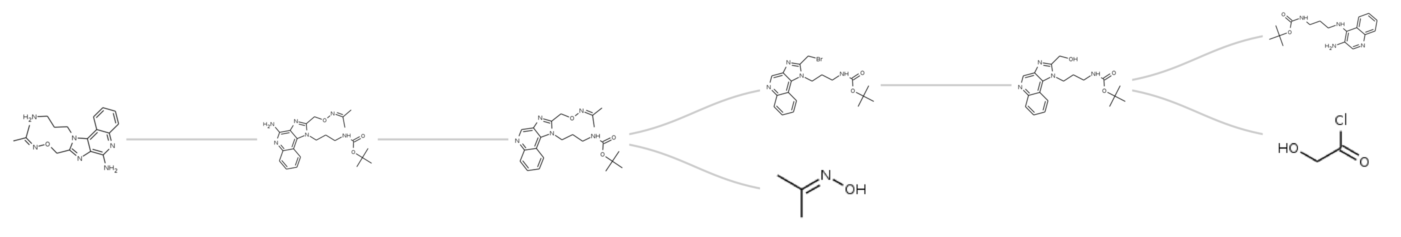

ASKCOS pathway, score: -15.201, ranking: 1, group: 0

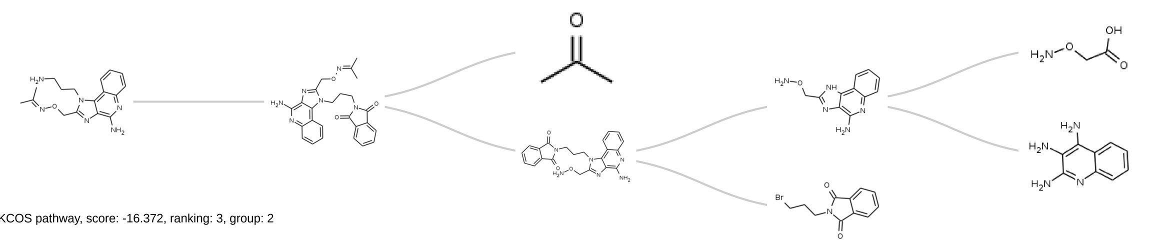

ASKCOS pathway, score: -16.372, ranking: 3, group: 2

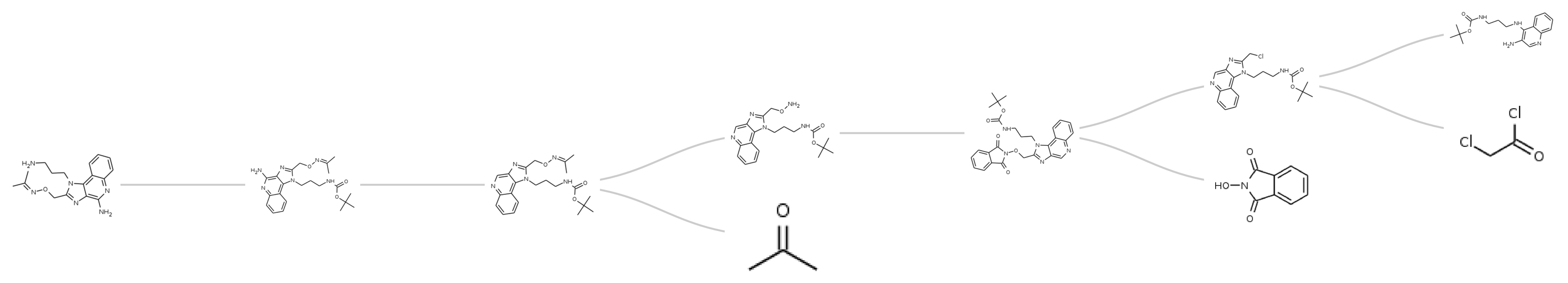

ASKCOS pathway, score: -16.584, ranking: 4, group: 3

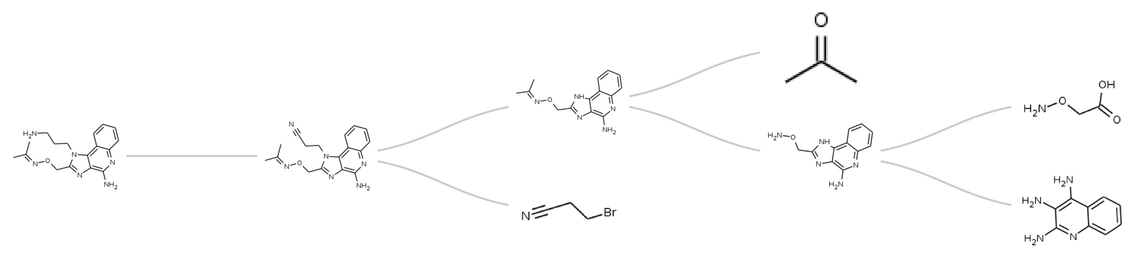

ASKCOS pathway, score: -17.659, ranking: 6, group: 4

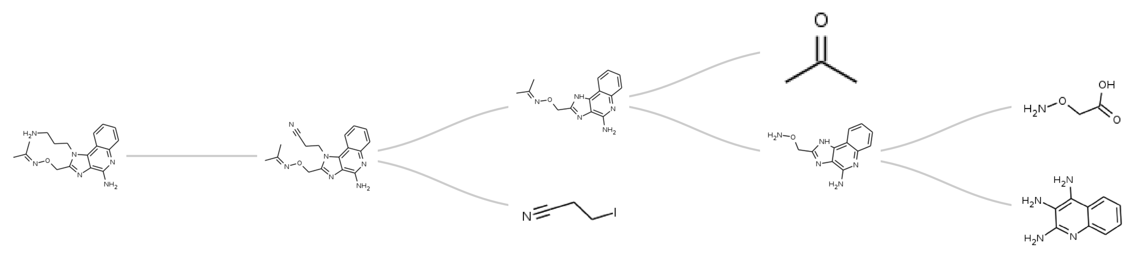

ASKCOS pathway, score: -17.856, ranking: 7, group: 5

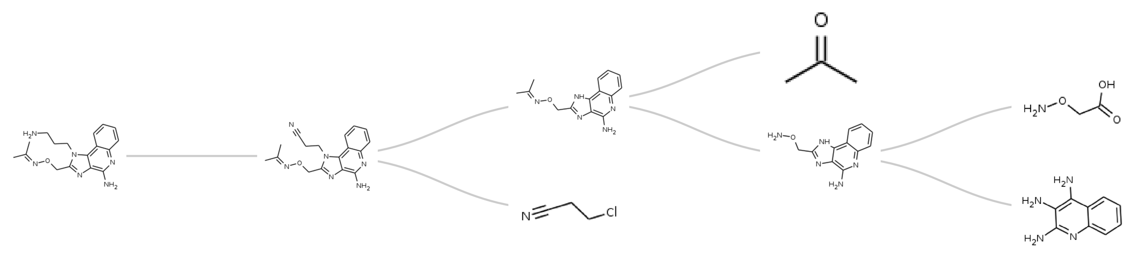

ASKCOS pathway, score: -17.990, ranking: 8, group: 6

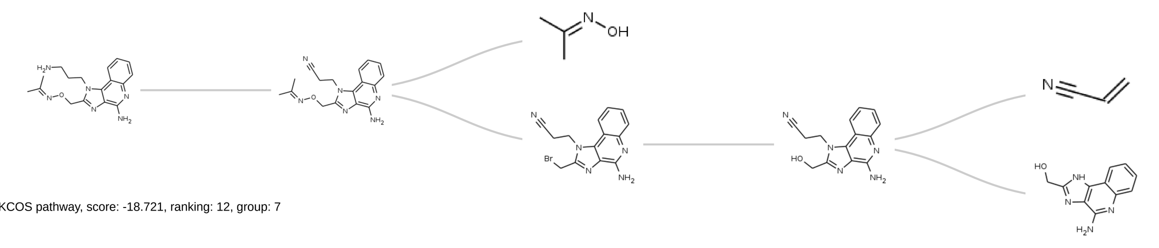

ASKCOS pathway, score: -18.721, ranking: 12, group: 7

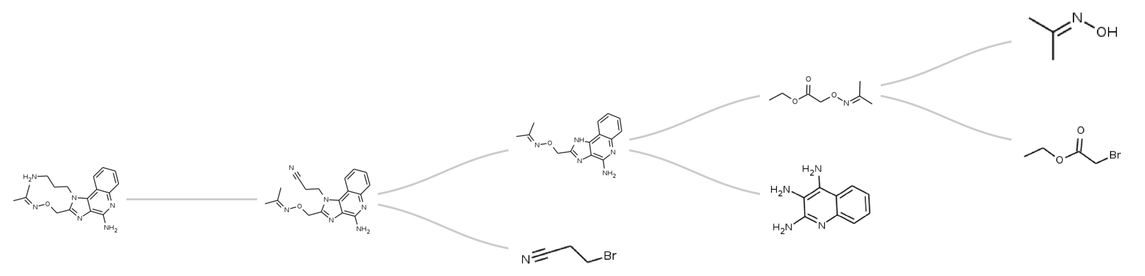

ASKCOS pathway, score: -18.798, ranking: 13, group: 8

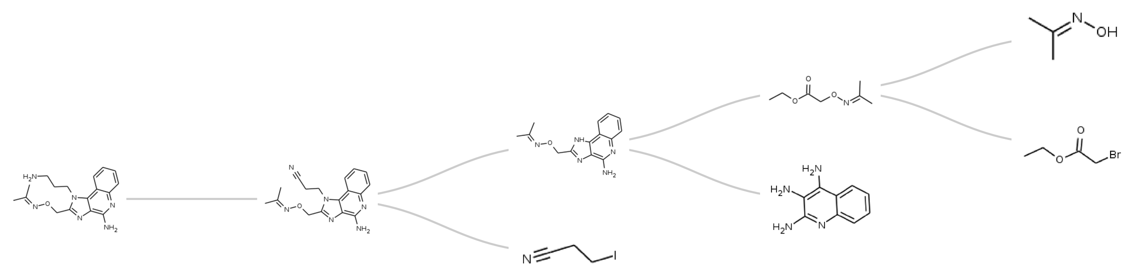

ASKCOS pathway, score: -18.977, ranking: 14, group: 9

Model ranks patent pathway not as top-1: Example 6

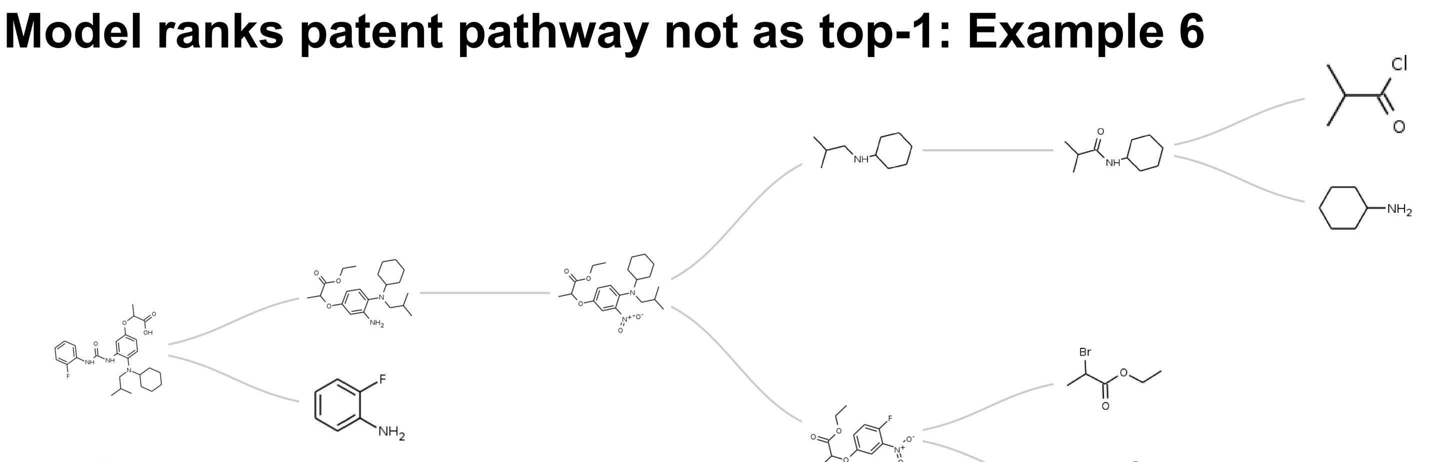

Piatachio pathway, score: -17.728, ranking: 2, Patent No: US20190002402A1

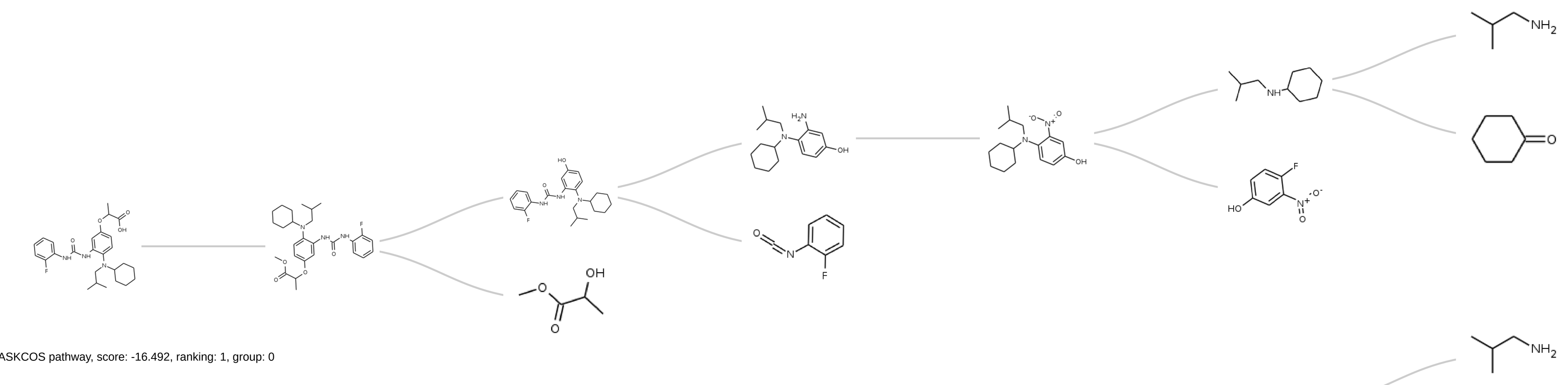

ASKCOS pathway, score: -16.492, ranking: 1, group: 0

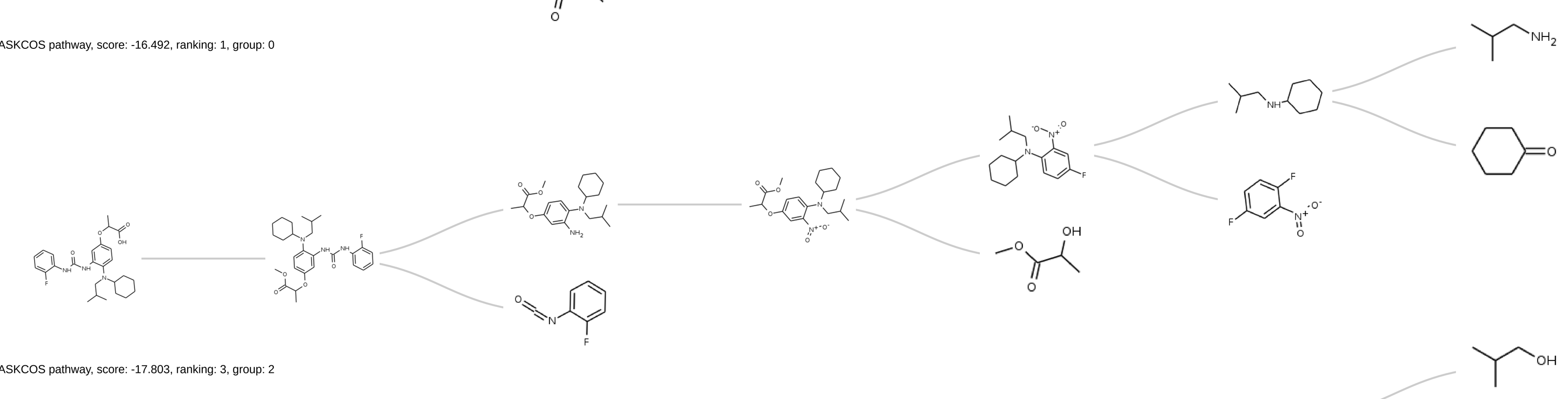

ASKCOS pathway, score: -17.803, ranking: 3, group: 2

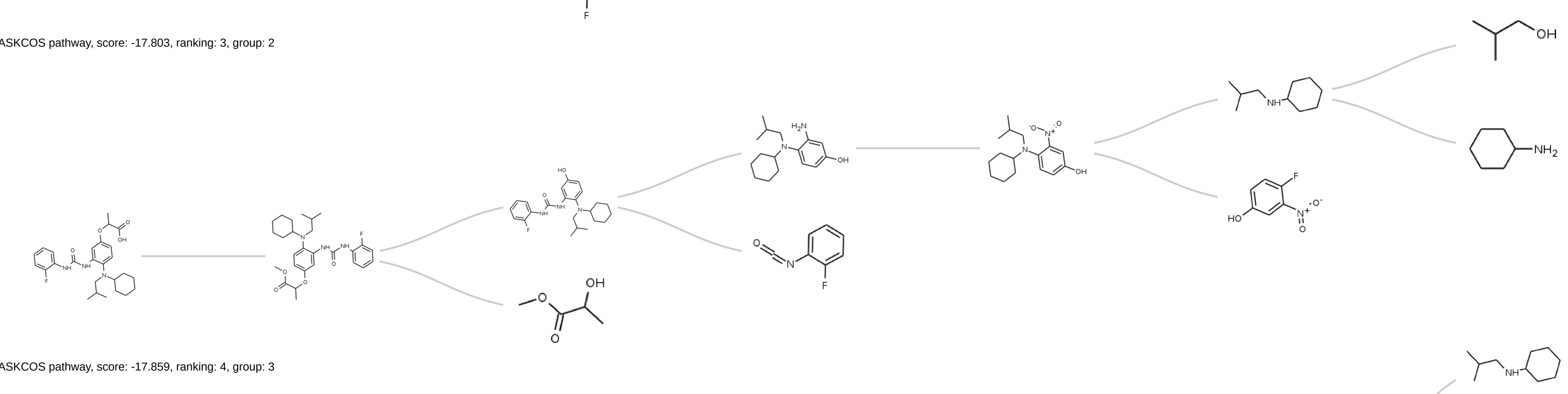

ASKCOS pathway, score: -17.859, ranking: 4, group: 3

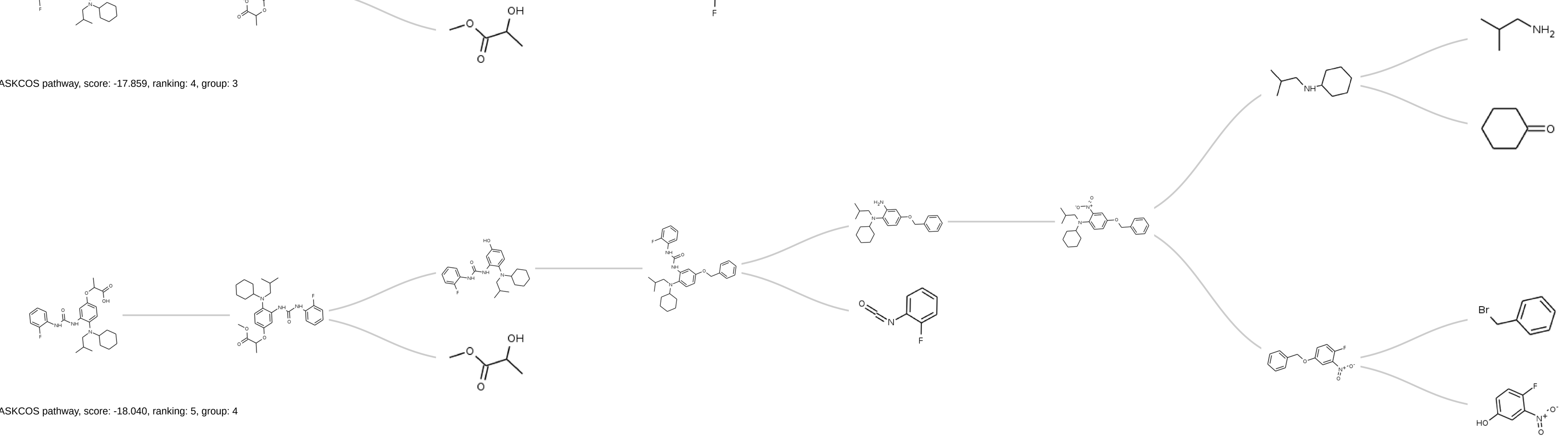

ASKCOS pathway, score: -18.040, ranking: 5, group: 4

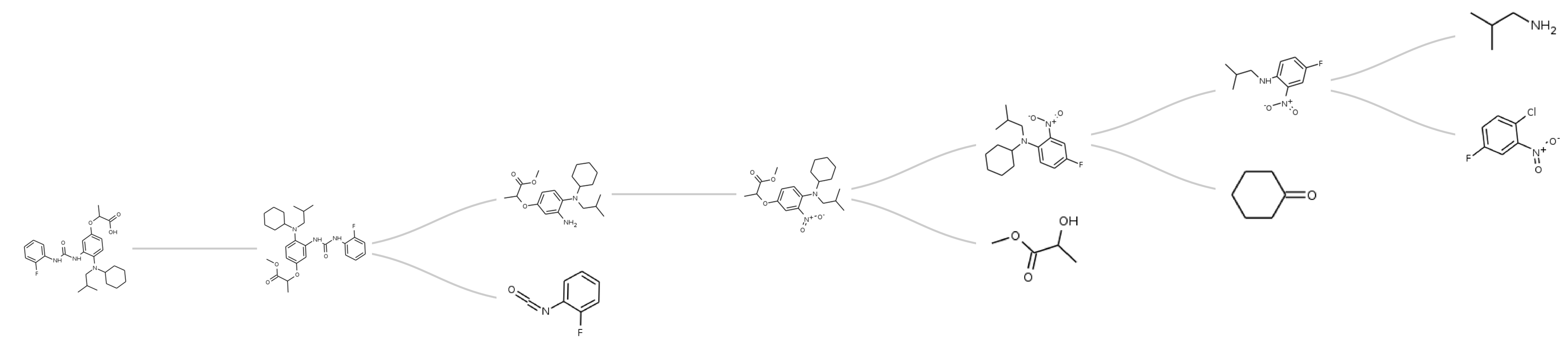

ASKCOS pathway, score: -18.646, ranking: 9, group: 5

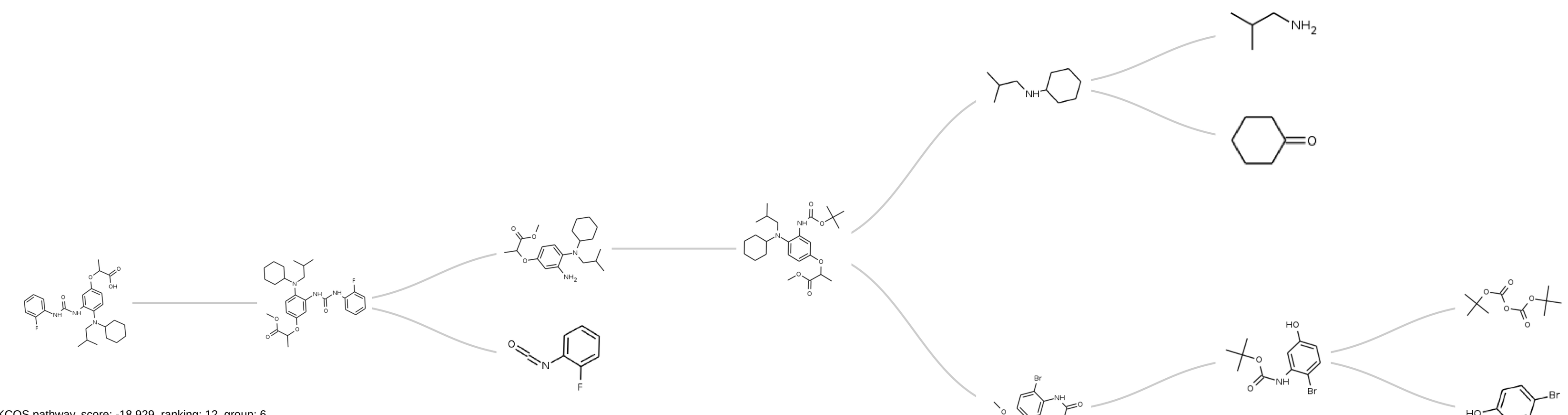

ASKCOS pathway, score: -18.929, ranking: 12, group: 6

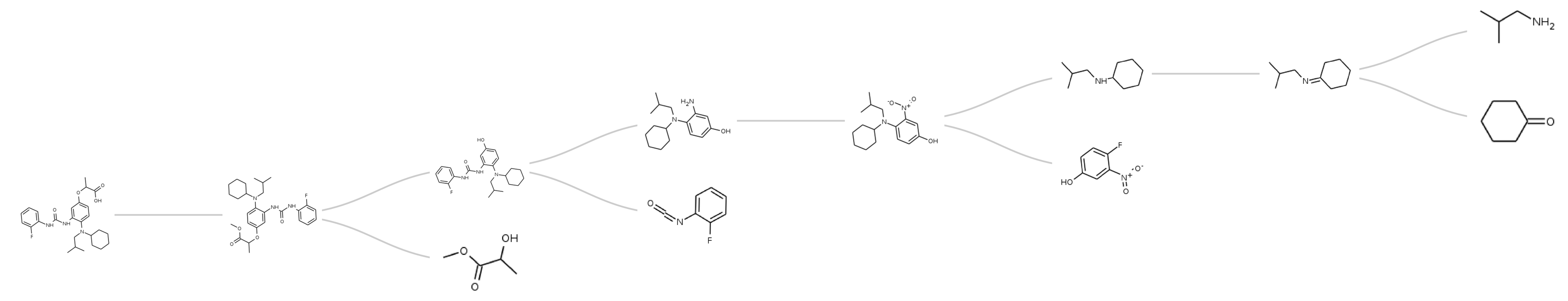

ASKCOS pathway, score: -19.192, ranking: 15, group: 7

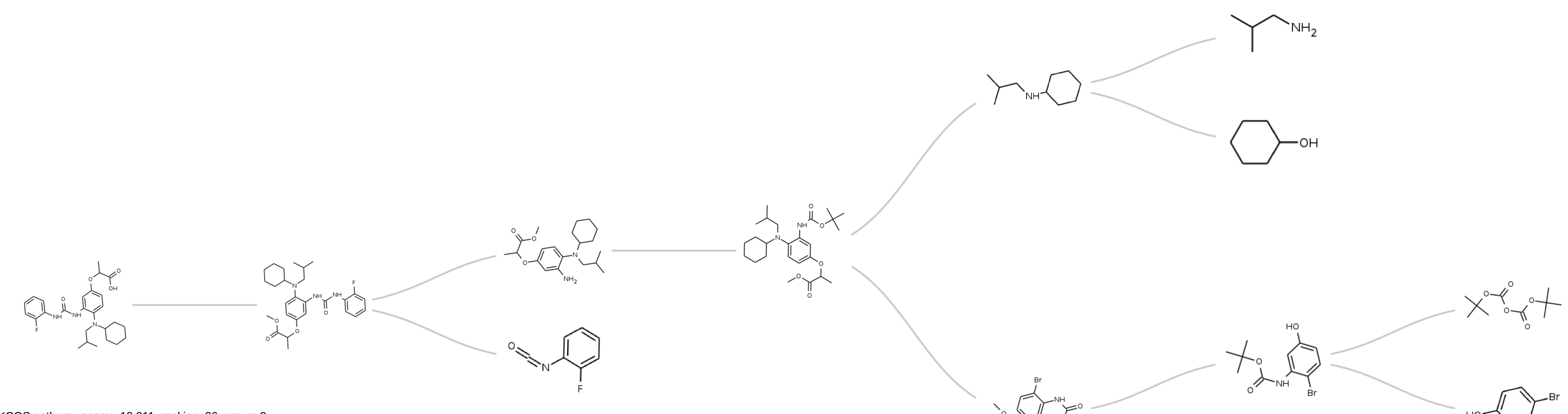

ASKCOS pathway, score: -19.911, ranking: 26, group: 8

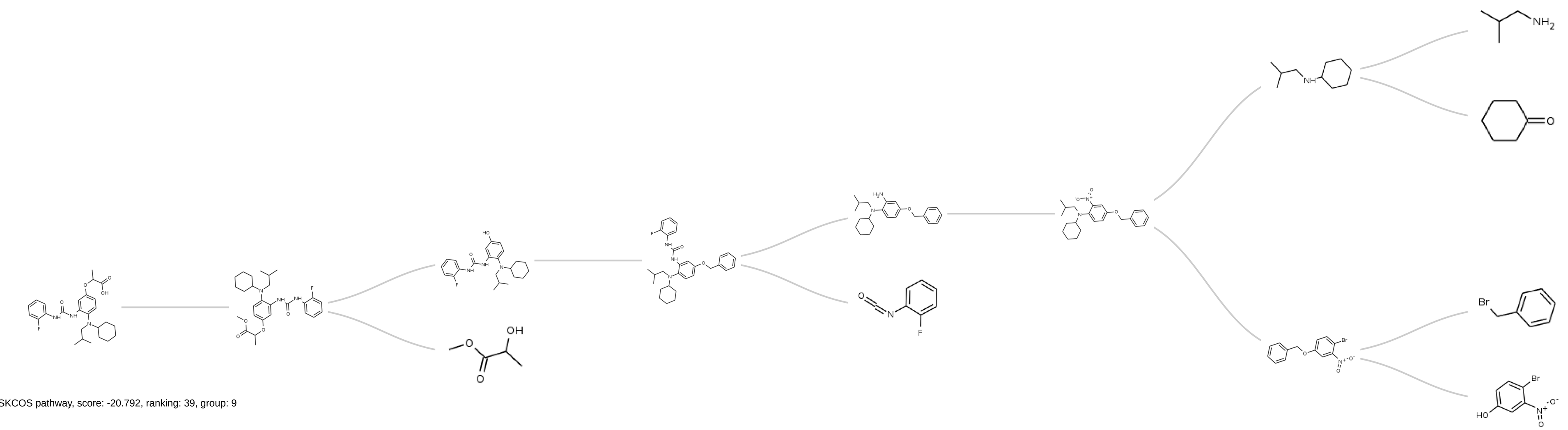

ASKCOS pathway, score: -20.792, ranking: 39, group: 9

Model ranks patent pathway not as top-1: Example 7

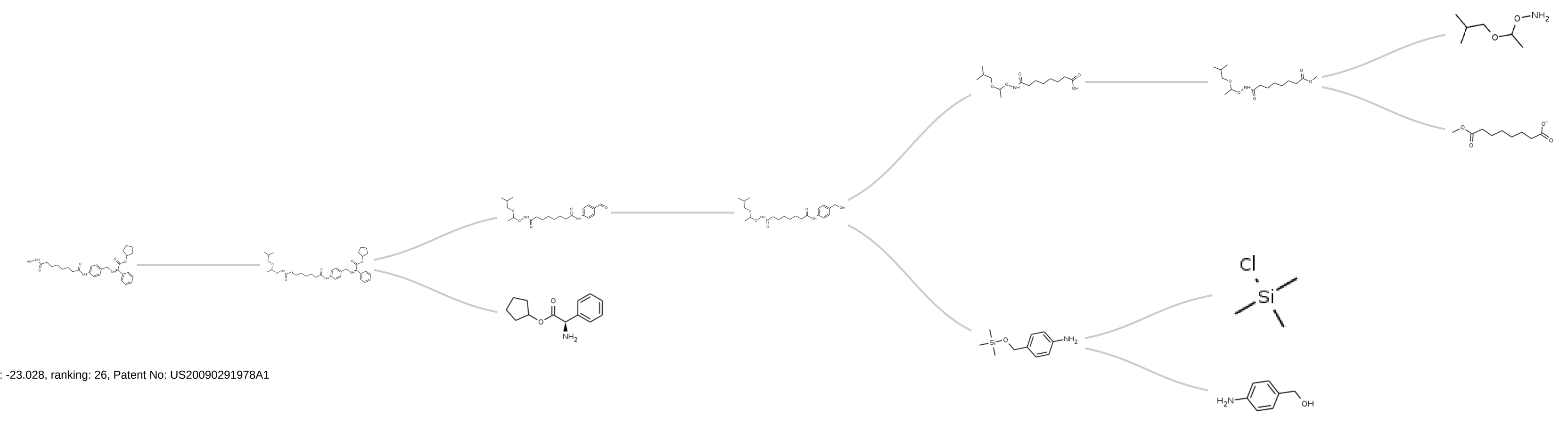

Piatachio pathway, score: -23.028, ranking: 26, Patent No: US20090291978A1

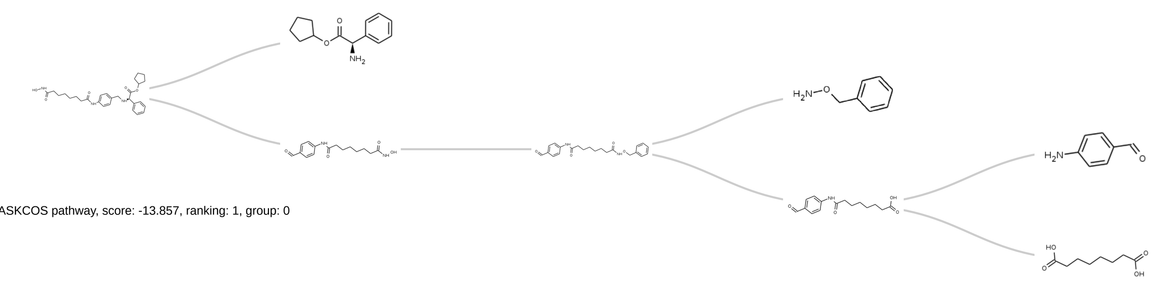

ASKCOS pathway, score: -13.857, ranking: 1, group: 0

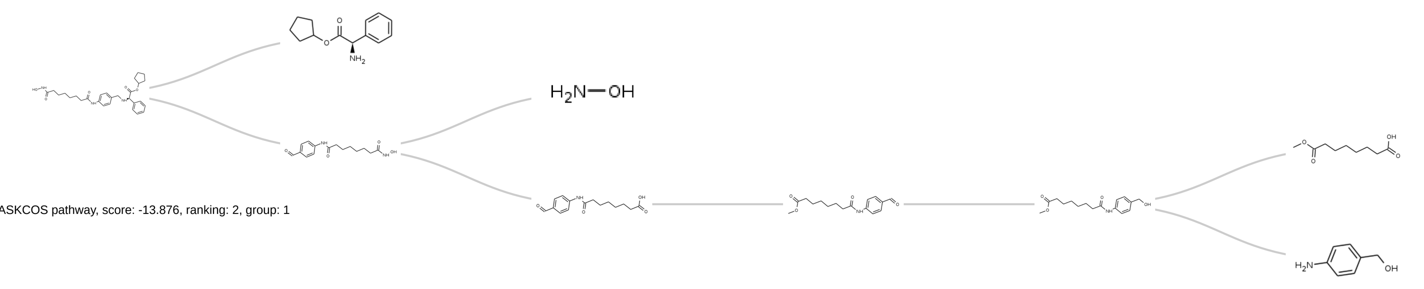

ASKCOS pathway, score: -13.876, ranking: 2, group: 1

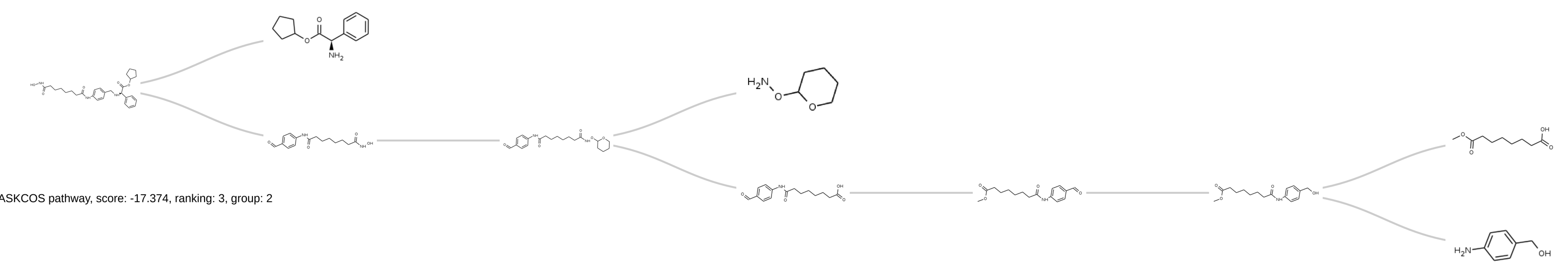

ASKCOS pathway, score: -17.374, ranking: 3, group: 2

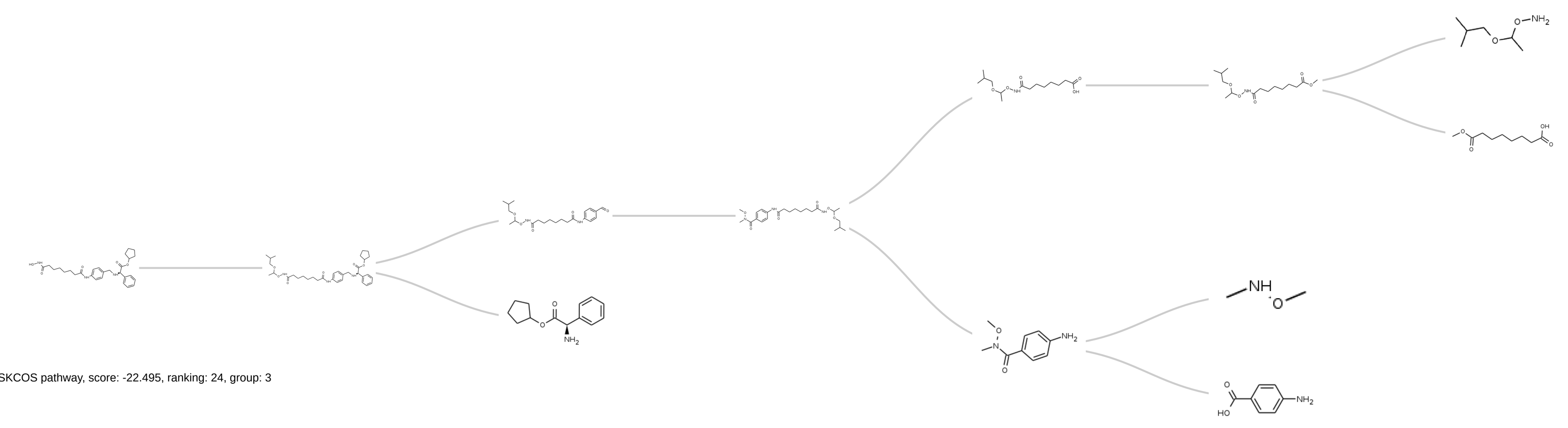

ASKCOS pathway, score: -22.495, ranking: 24, group: 3

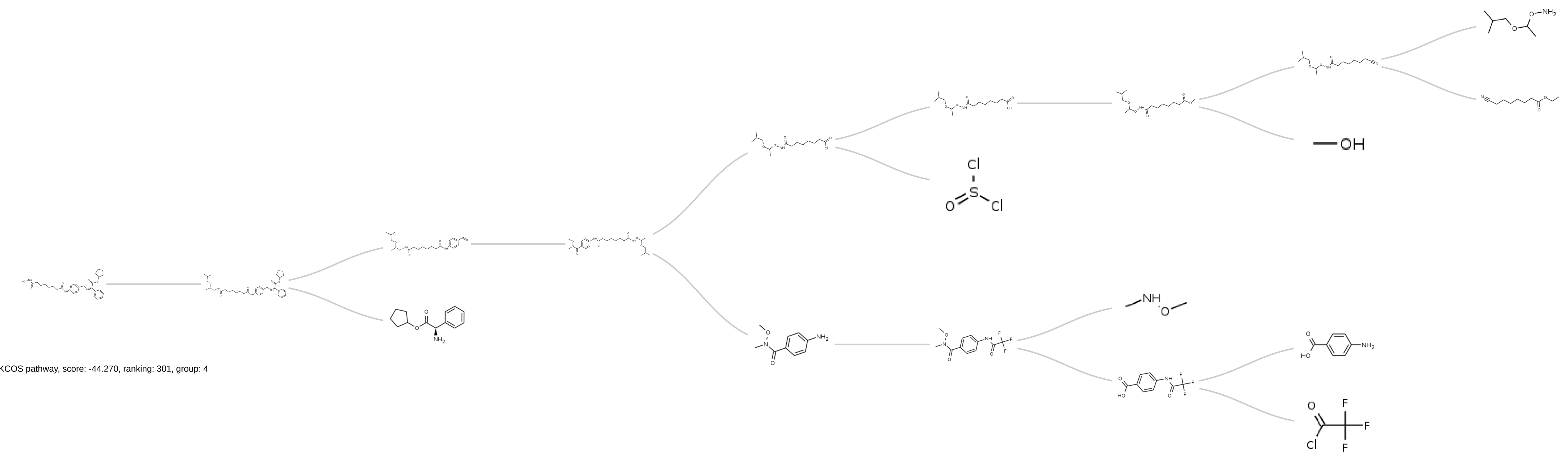

ASKCOS pathway, score: -44.270, ranking: 301, group: 4

Model ranks patent pathway not as top-1: Example 8

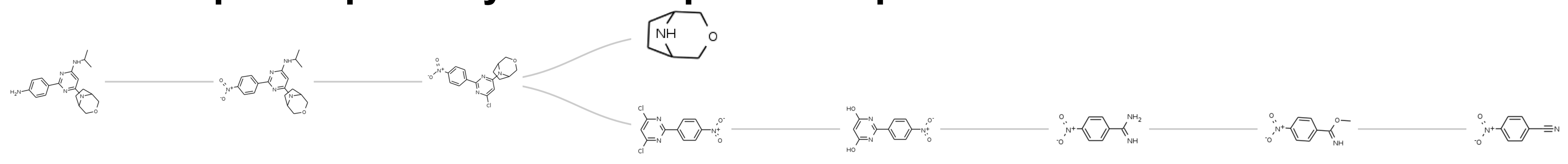

Piatachio pathway, score: -14.032, ranking: 3, Patent No: US20120134959A1

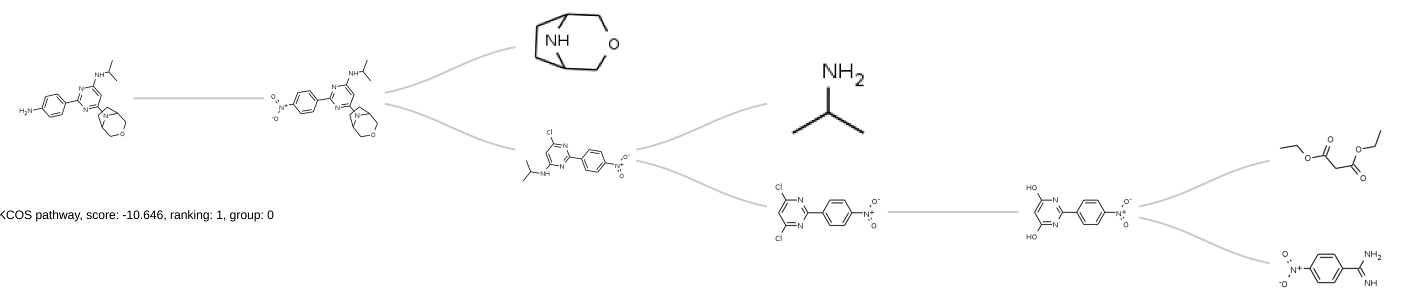

ASKCOS pathway, score: -10.646, ranking: 1, group: 0

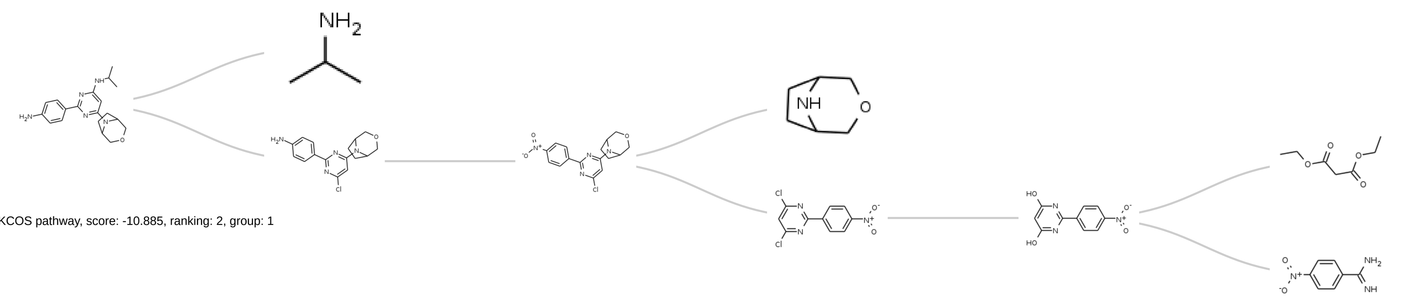

ASKCOS pathway, score: -10.885, ranking: 2, group: 1

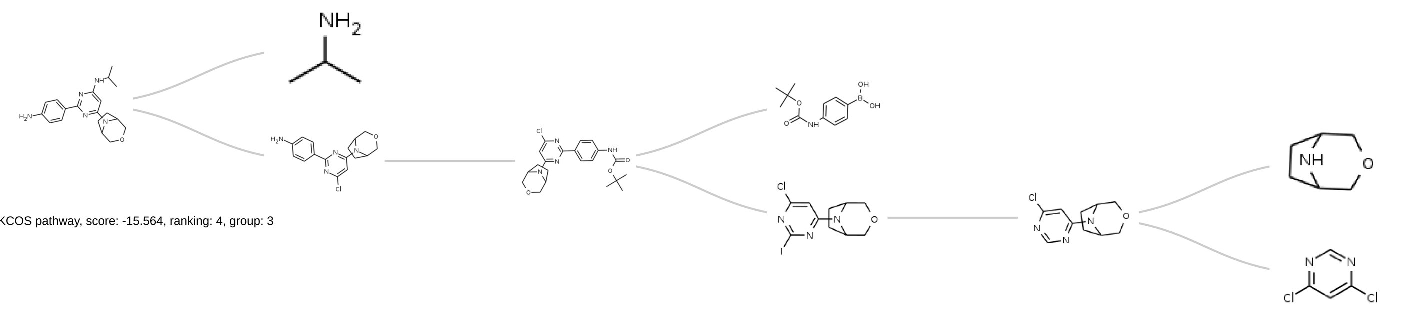

ASKCOS pathway, score: -15.564, ranking: 4, group: 3

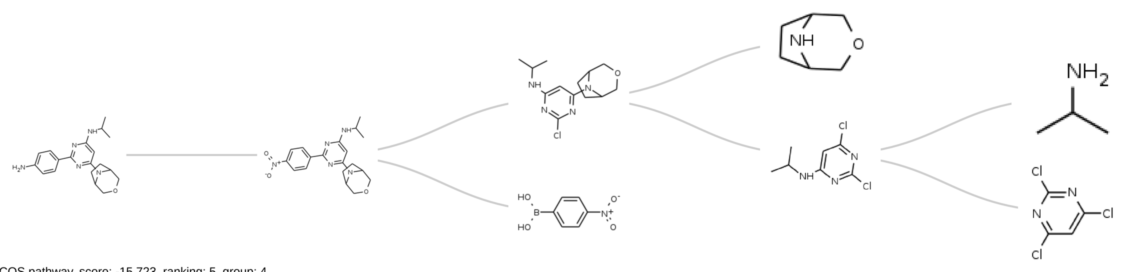

ASKCOS pathway, score: -15.723, ranking: 5, group: 4

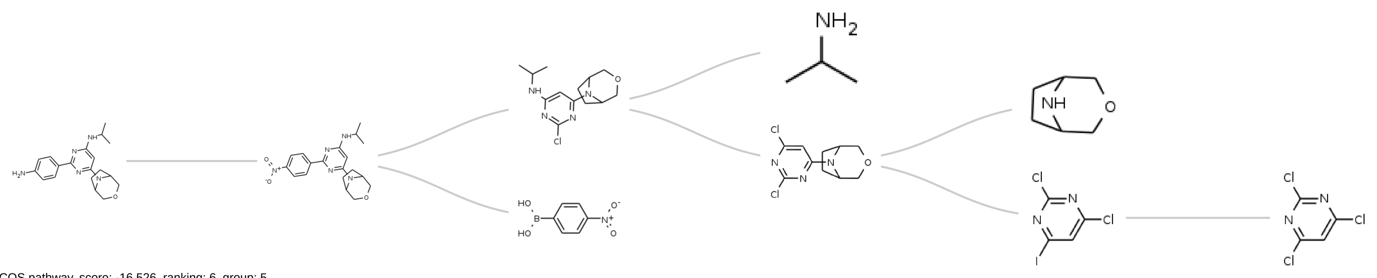

ASKCOS pathway, score: -16.526, ranking: 6, group: 5

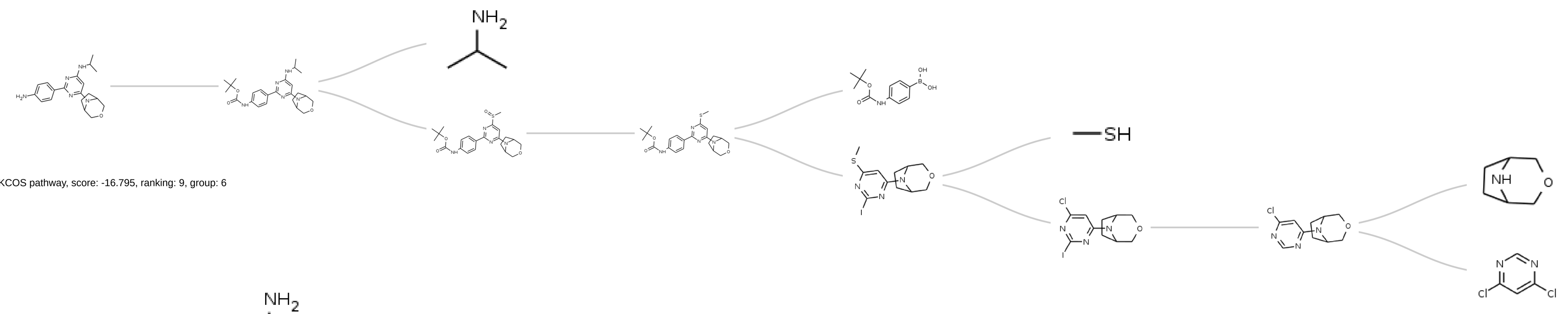

ASKCOS pathway, score: -16.795, ranking: 9, group: 6

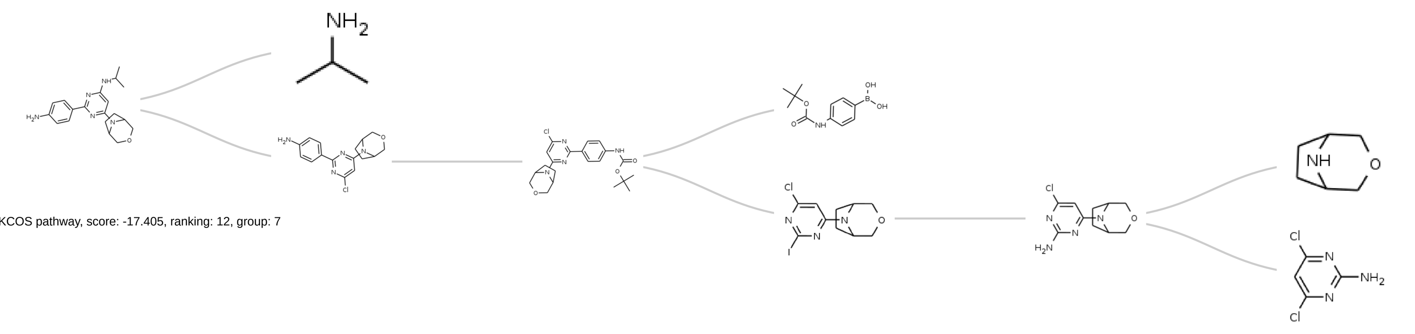

ASKCOS pathway, score: -17.405, ranking: 12, group: 7

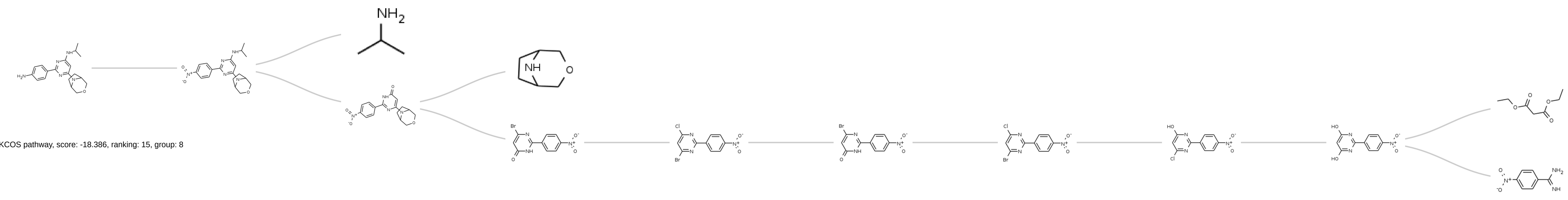

ASKCOS pathway, score: -18.386, ranking: 15, group: 8

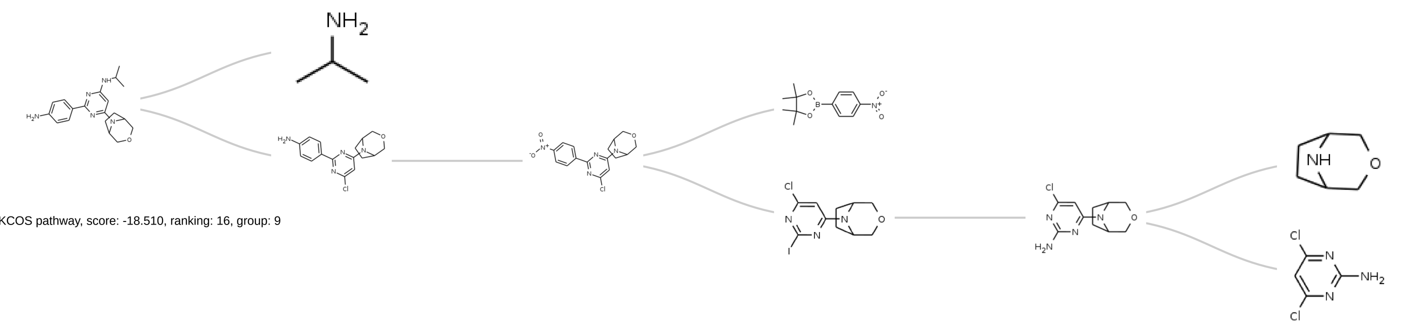

ASKCOS pathway, score: -18.510, ranking: 16, group: 9

Model ranks patent pathway not as top-1: Example 9

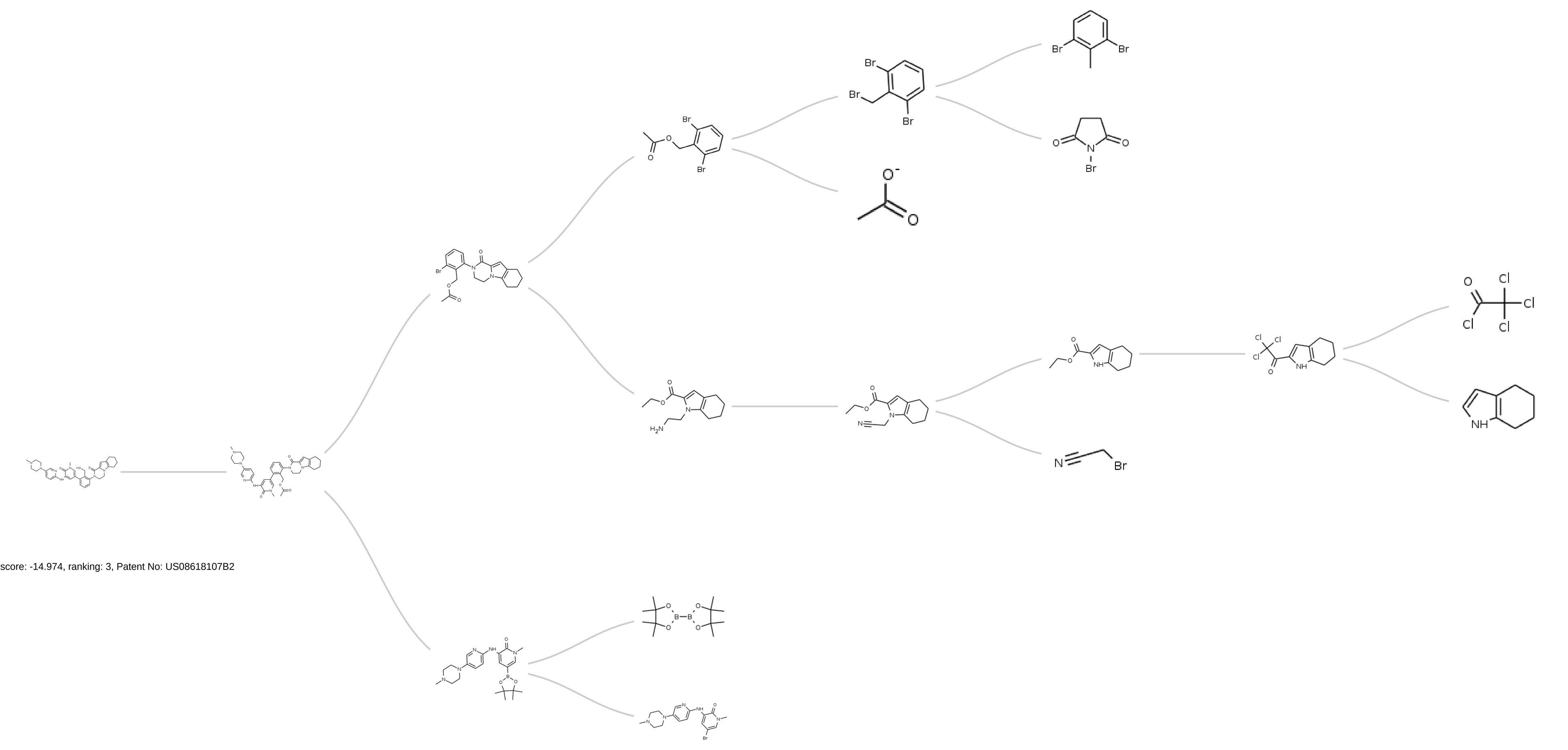

Piatachio pathway, score: -14.974, ranking: 3, Patent No: US08618107B2

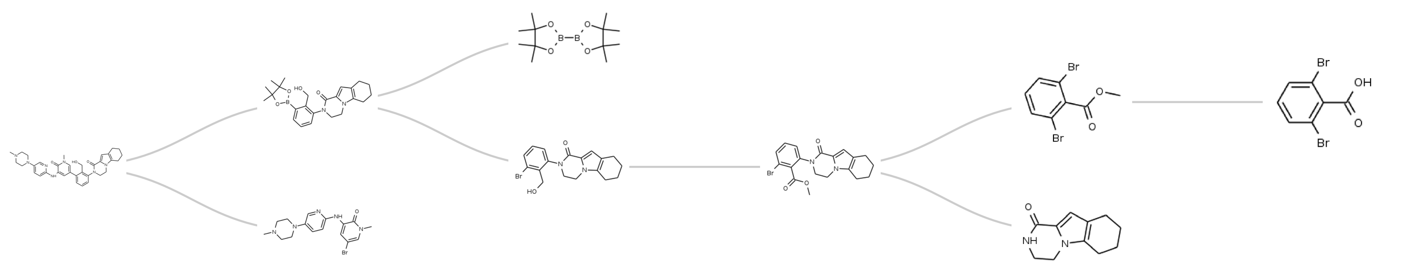

ASKCOS pathway, score: -13.110, ranking: 1, group: 0

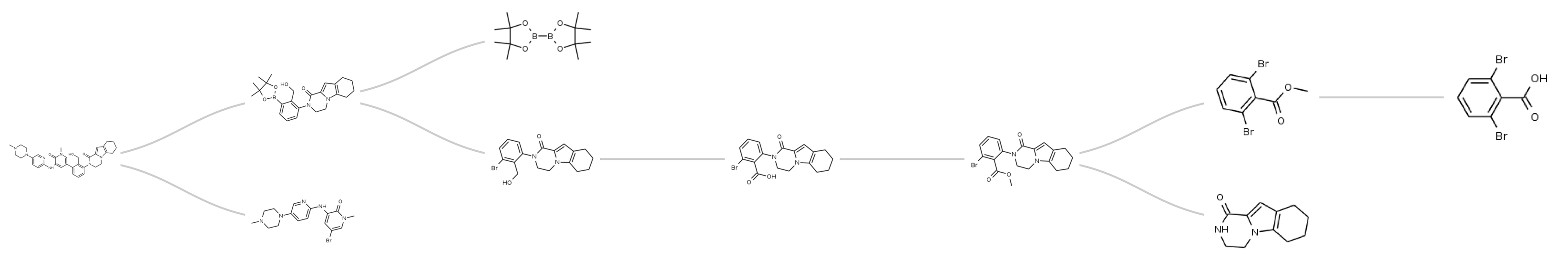

ASKCOS pathway, score: -14.090, ranking: 2, group: 1

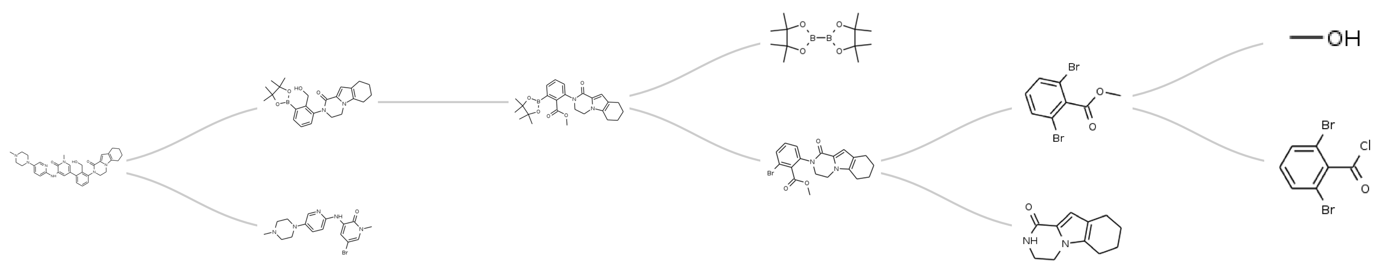

ASKCOS pathway, score: -15.758, ranking: 4, group: 3

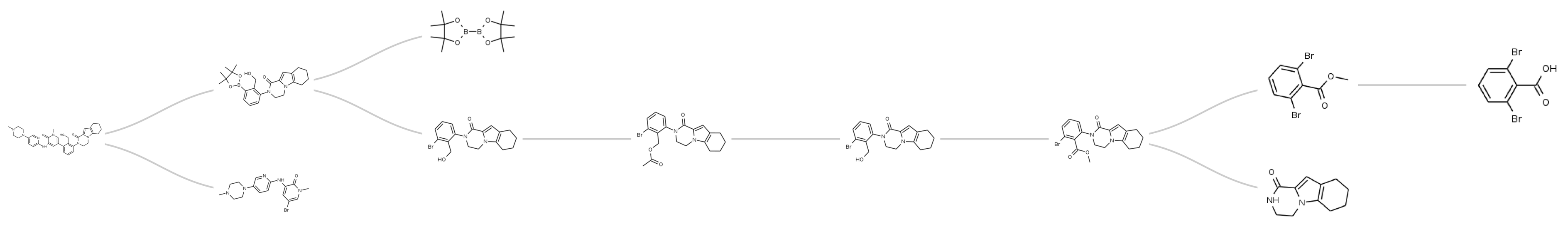

ASKCOS pathway, score: -16.591, ranking: 5, group: 4

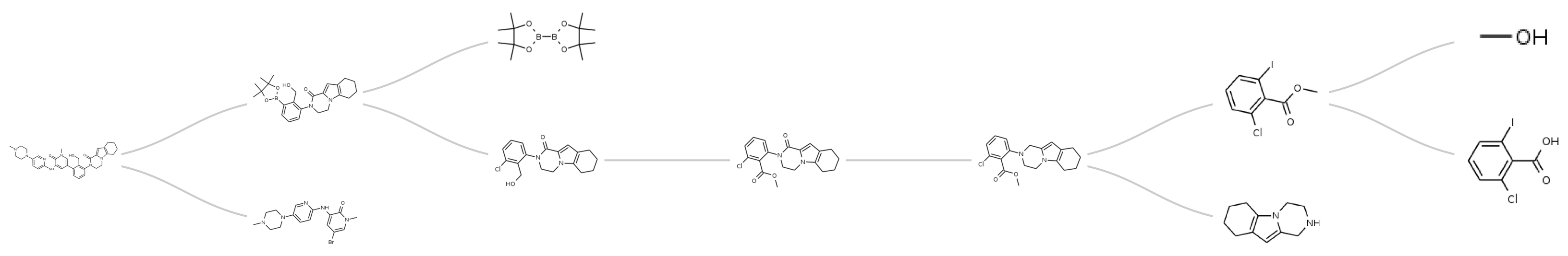

ASKCOS pathway, score: -17.228, ranking: 6, group: 5

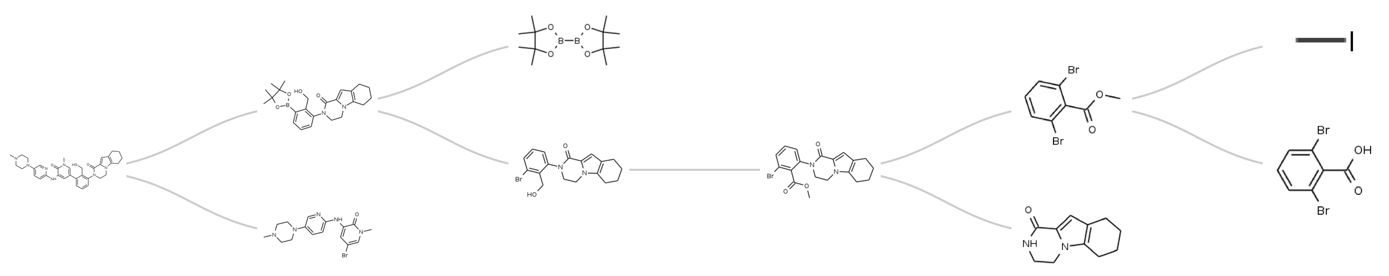

ASKCOS pathway, score: -17.338, ranking: 7, group: 6

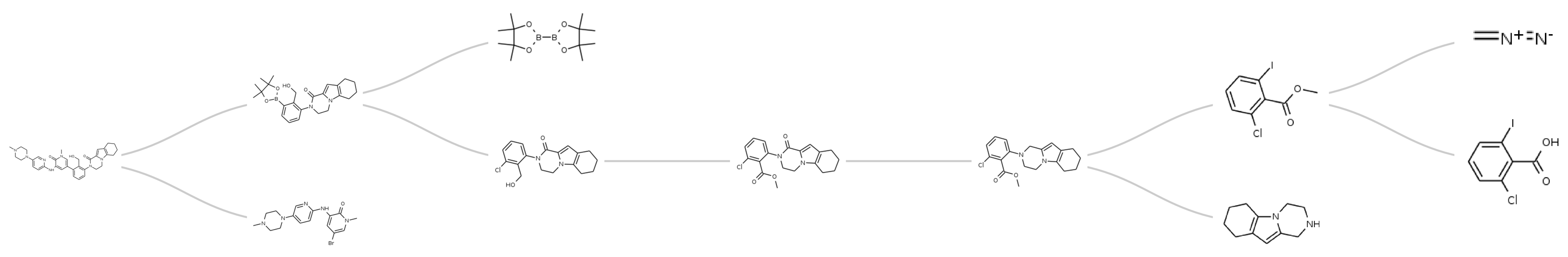

ASKCOS pathway, score: -18.376, ranking: 14, group: 7

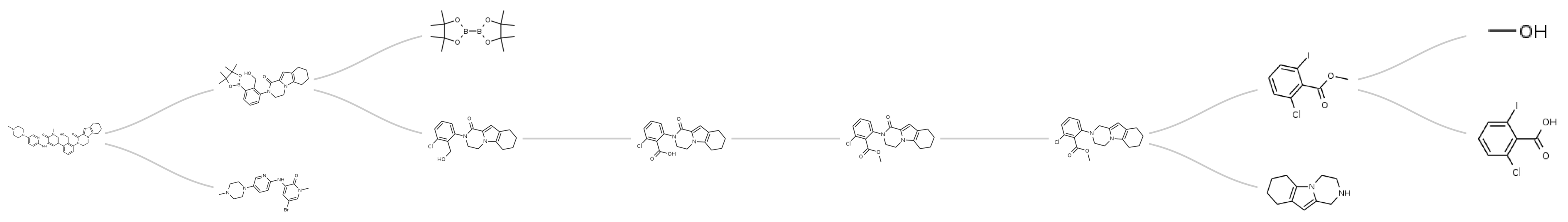

ASKCOS pathway, score: -18.414, ranking: 15, group: 8

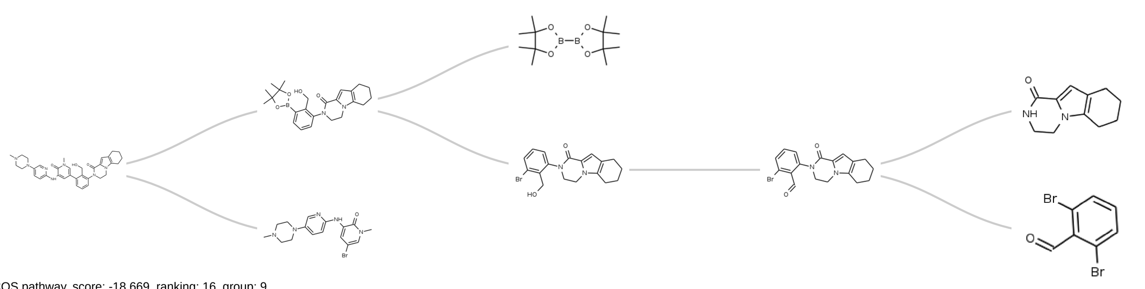

ASKCOS pathway, score: -18.669, ranking: 16, group: 9

Model ranks patent pathway not as top-1: Example 10

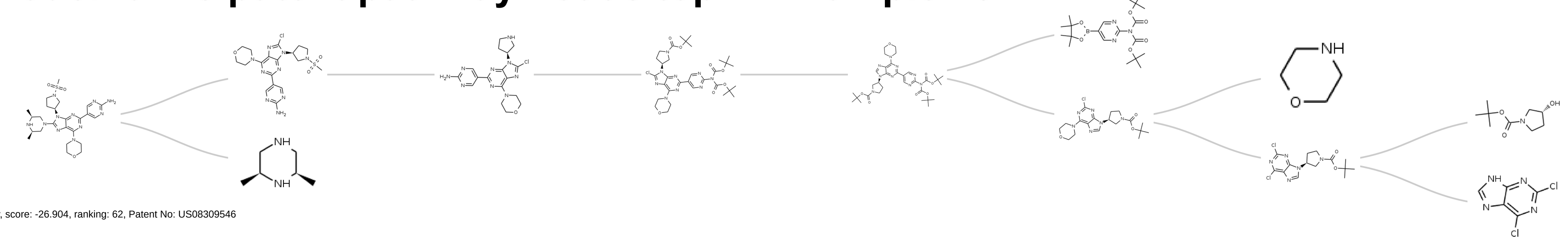

Piatachio pathway, score: -26.904, ranking: 62, Patent No: US08309546

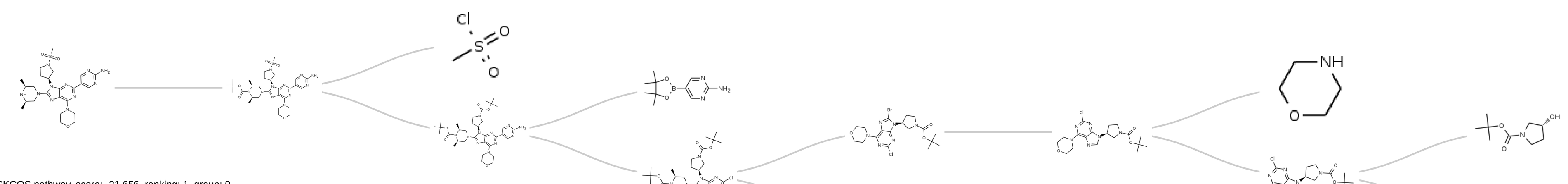

ASKCOS pathway, score: -21.656, ranking: 1, group: 0

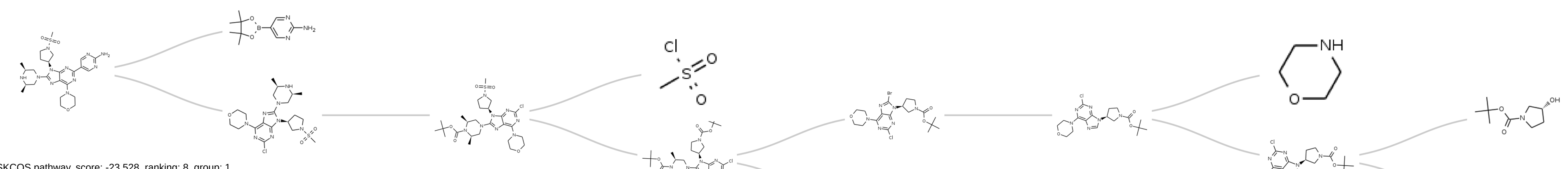

ASKCOS pathway, score: -23.528, ranking: 8, group: 1

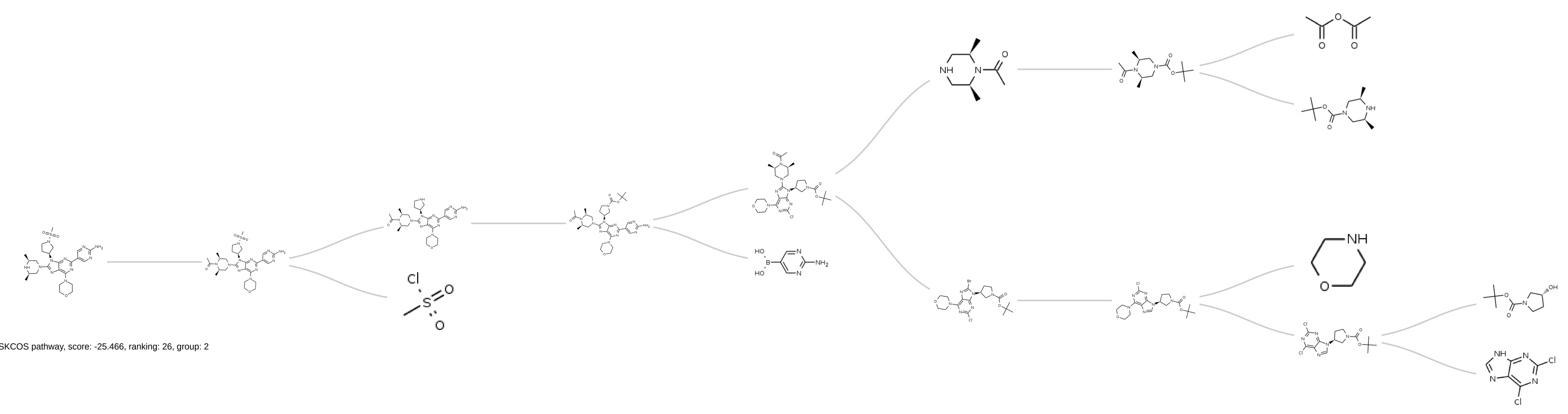

ASKCOS pathway, score: -25.466, ranking: 26, group: 2

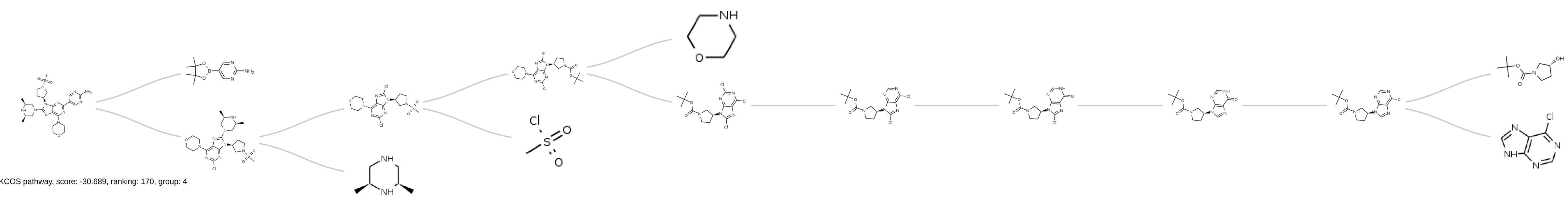

ASKCOS pathway, score: -30.689, ranking: 170, group: 4

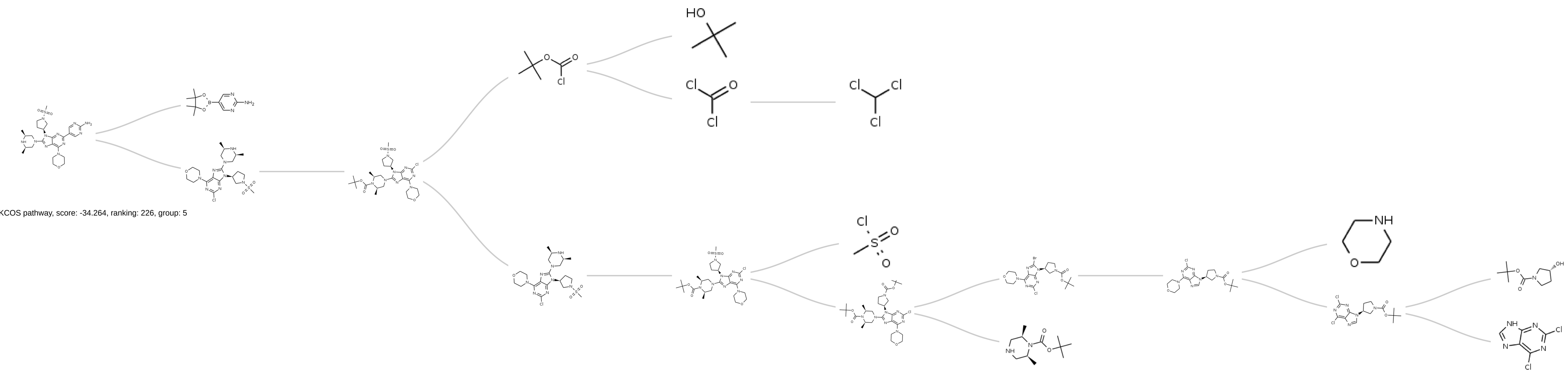

ASKCOS pathway, score: -34.264, ranking: 226, group: 5

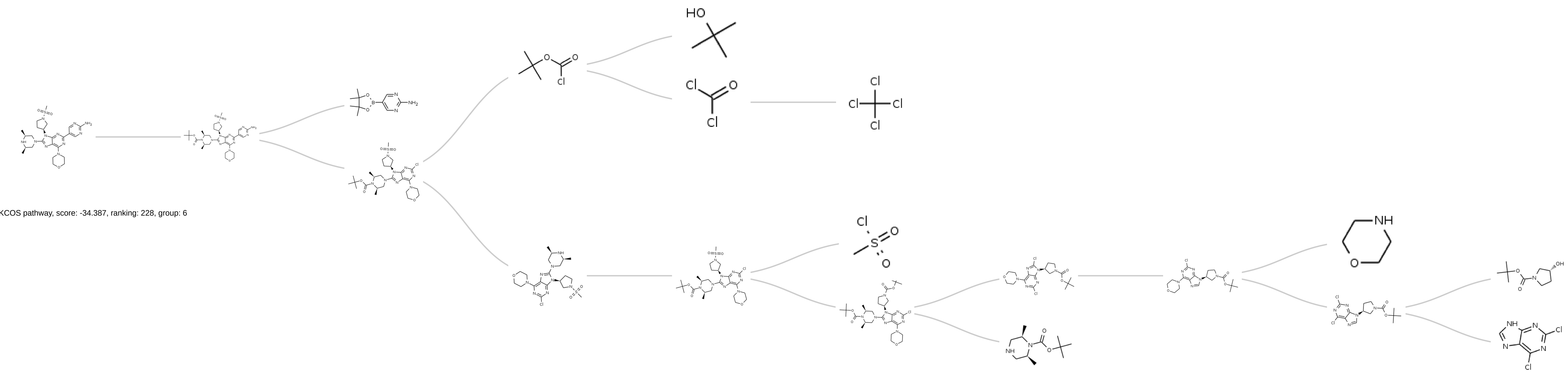

ASKCOS pathway, score: -34.387, ranking: 229, group: 6

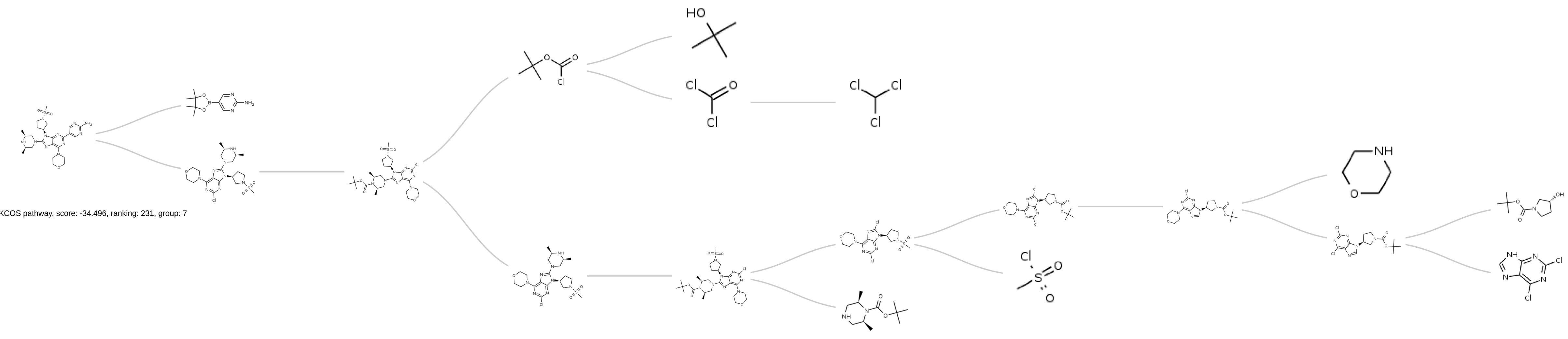

ASKCOS pathway, score: -34.496, ranking: 231, group: 7

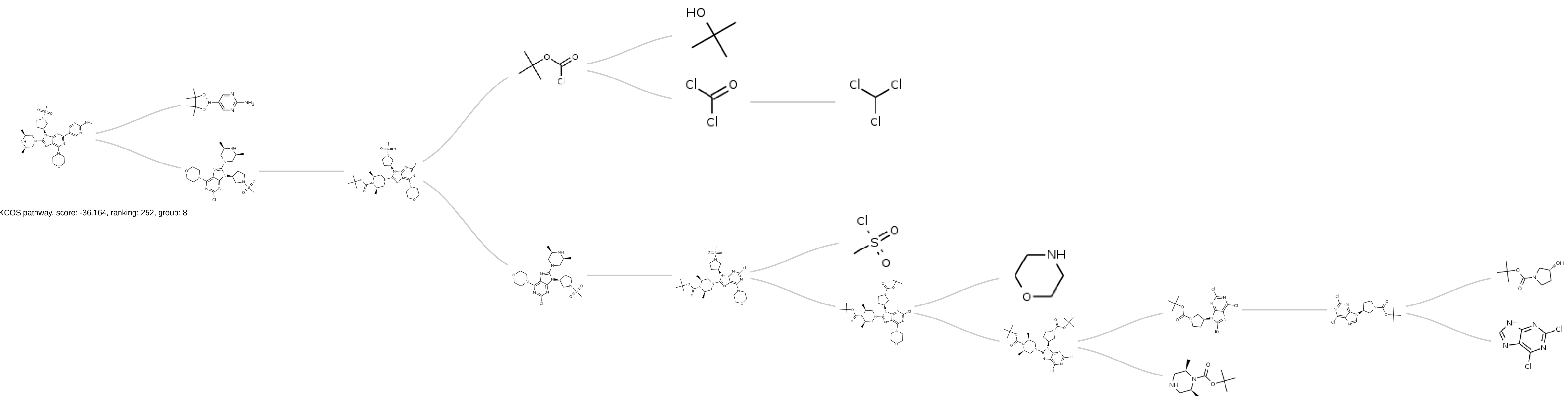

ASKCOS pathway, score: -36.164, ranking: 252, group: 8

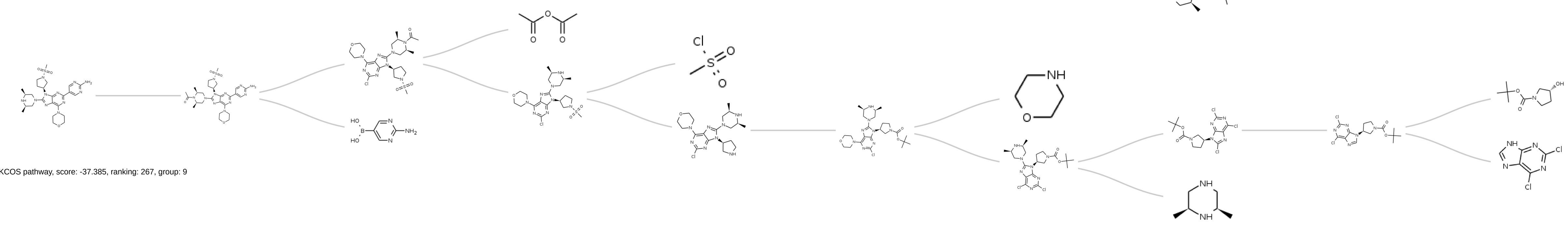

ASKCOS pathway, score: -37.385, ranking: 267, group: 9
